# 10-year opportunistic mammographic screening scenario in Brazil and its impact on breast cancer early detection: a nationwide population-based study

**DOI:** 10.7189/jogh.12.04061

**Published:** 2022-10-14

**Authors:** Isabela Campeti Cuoghi, Mariana Furlani da Silva Soares, Gustavo Motta Cabello dos Santos, Francisco José Candido dos-Reis, Omero Benedicto Poli-Neto, Jurandyr Moreira de Andrade, Priscila Longhin Bosquesi, Leonardo Fleury Orlandini, Daniel Guimarães Tiezzi

**Affiliations:** 1 CEPAM – Centro de Pesquisa Avançada em Medicina da UNILAGO, Faculdade de Medicina UNILAGO, União das Faculdades dos Grandes Lagos, São José do Rio Preto, São Paulo, Brazil; 2 Faculdade de Medicina de Ribeirão Preto FMRP – USP, Ribeirão Preto, São Paulo, Brazil; 3 Laboratory for Translational Data Science - University of São Paulo, São Paulo, Brazil; 4 Faculdade de Ciências Farmacêuticas UNESP, Araraquara, São Paulo, Brazil

## Abstract

**Background:**

Mammographic screening has been used to reduce breast cancer mortality worldwide and remains the main modality for the early detection of this disease. Women from low- and middle-income countries still lack access to periodic mammograms and efficient health care. This cross-sectional study aimed to explore opportunistic mammographic coverage in Brazil, while considering the privately insured population and its association with early breast cancer (EBC) detection.

**Methods:**

Data on population, gross domestic product (GDP), number of mammograms performed under the Sistema Único de Saúde (SUS) public health system or private system, and women diagnosed with early-stage breast cancer from 2010 to 2019 were retrieved from publicly available databases.

**Results:**

A total of 39 555 636 mammograms with an average of 3 955 564 ± 395 704 mammograms were obtained per year from 2010 to 2019 in Brazil. Most examinations (58.6%) were performed in the target population (50-69 years old), while 32% were performed in women aged 40-49, and 9.4% were performed in women <40 years or >70 years of age. The 10-year mammogram coverage was 30.6% in the target population and 24.8% in the population aged 40-49 years, with significant variation across states and municipalities. The overall EBC detection rates in Brazil were 30.6% in populations aged 50-70 and 24.8% in those aged 40-50 years. We observed a positive correlation between coverage and EBC detection rate (r = 0.68; *P* = 0.0001 (50-70 years) and r = 0.75; *P* < 0.0001 (40-50 years)). According to the GDP, the municipalities with higher GDP *per capita* had higher mammogram coverage (*P* < 0.0001).

**Conclusions:**

The coverage of mammographic screening for women under the SUS is far below the international guidelines. Additionally, a significant number of mammograms have been performed in non-target populations. This scenario reflects the problematic screening programs in developing countries and reflects low rates of EBC diagnosis. As Brazil is a continental country with heterogeneous socioeconomic indicators, we observed significant variations in the number of mammograms performed by age groups when separated by states and municipalities. Even when considering supplemental health system coverage, municipalities with higher GDP *per capita* were associated with higher mammogram coverage.

Breast cancer is the leading type of cancer in women and accounts for approximately 30% of female malignant tumours, except for skin cancers [1]. Despite molecular heterogeneity, breast cancer can be cured in more than 80% of cases if detected in its early stages [2]. Mammography remains the main modality for screening asymptomatic breast cancer in average-risk women worldwide. Studies have demonstrated that countries with organized screening programs have achieved up to a 40% mortality reduction for women who participated in screening [3,4].

Recommendations for mammography screening for women with an average risk have been controversial among agencies and medical societies, balancing the benefits and harms of screening. For example, the US Preventive Services Task Force recommends biennial mammography for women aged 50-74 years, with individualized decisions for women aged 40-49 years [5]; the American College of Radiology recommends annual mammography screening starting at age 40, with no upper age limit [6]; and the European Commission Initiative on Breast Cancer recommends mammography every 2 years for women aged 50-69 years and every 2-3 years for women aged 45-49 years and 70-74 years [7].

Women from low- and middle-income countries do not have access to an organized screening program for breast cancer prevention, mainly due to limited budget for public health, geographical distribution and quality of mammographic equipment, limited human and consumable resources, and lack of awareness and acceptance of the screening programs among women [8,9].

In Brazil, mammographic screening is an opportunistic procedure. While the Brazilian Ministry of Health recommends mammography every 2 years for women aged 50-69 years, it recommends against mammography for asymptomatic women aged <50 and >70 years [10]. It is estimated that more than 60% of Brazilian women are treated only by the Brazilian public health system (Sistema Único de Saúde, SUS) [11]. Women who have private insurance or pay for their exams mostly follow the recommendations of medical societies (for example, Mastology Brazilian Society and Brazilian College of Radiology), which recommend annual mammography starting at 40 years of age, with an upper limit depending on life expectancy [12].

A recent study addressed breast cancer screening coverage provided under Brazilian public insurance from 2008 to 2017 and showed insufficient mammography coverage and a lack of uniformity between different Brazilian states and regions [13]. However, the study did not consider mammograms performed in the private health system, which can exceed 30% of the coverage in some locations in Brazil. The early breast cancer detection rate and number of mammograms performed outside the age range of 50-69 years recommended by the Brazilian Ministry of Health were not evaluated in previous studies. Therefore, this study aimed to evaluate the opportunistic mammographic coverage in Brazil, taking the privately insured population and gross domestic product (GDP) and its association with early breast cancer (EBC) detection into consideration.

## METHODS

### Database and data preprocessing

We downloaded data from online sources to estimate mammogram coverage and EBC detection rates at state and municipality resolutions. The population estimates for each municipality and state from 2010 to 2019 were downloaded from the Instituto Brasileiro de Geografia e Estatística (IBGE) website [14,15]. The population in 2010 and its distribution by gender and age were based on the 2010 Brazilian Census [16]. Complete population estimates from 2010 to 2019 were obtained from 5549 municipalities and population density was estimated by dividing the average population estimates from 2010 to 2019 by the municipality area per km^2^ [17]. The GDP information for each municipality in Brazil from 2010 to 2019 was downloaded from the IBGE repository [18]. We used the mean GDP value from 2010 to 2019.

The total number of mammogram screenings per county/state from 2010 to 2019 was obtained from the SUS outpatient information system (DATASUS-SIA-PA) database. All database files from the SIA-PA were downloaded from the DATASUS repository [19] using the OpenMP API written in C [20]. The files were read in the R environment and all the screening mammogram reports were retrieved based on the “204030188” procedure code (bilateral screening mammography). Screening examinations for men were excluded from the study.

Breast cancer data were obtained from the Brazilian National Cancer Institute (INCA) [21] and the Oncocentro Foundation of São Paulo (FOSP) [22]. We filtered all breast cancer reports based on code C50 from the International Classification of Diseases (ICD-10) [23]. In total, 288 975 and 80 772 reports were found in the INCA and FOSP databases, respectively. Patients with an undefined stage (93 598 in INCA and 1 908 in FOSP databases) and male (2245 in INCA and 552 in FOSP databases) breast cancer were excluded. Additionally, 10 233 reports in the INCA database did not contain information about the year of diagnosis and 13 did not contain information about the birthplace or place of residence and were thus excluded. A total of 261 198 reports were used for the analysis, and we defined EBC as those patients diagnosed with stage 0 (in situ carcinoma) and stage I. The rate of early-stage detection was estimated by the ratio of the number of EBC cases to the total number of breast cancer cases reported.

### Statistical analyses

Mammography screening coverage was estimated based on biennial mammography examinations. The mean 10-year mammography coverage was then estimated by the total mammograms performed between 2010 and 2019 per five times the mean population estimates for women aged 40-49 (40-50 group) and 50-69 (50-70 group) years and expressed as a percentage of the target population. The target populations were adjusted by the percentage of women covered by the supplemental health system, as reported by the Agência Nacional de Saúde Suplementar [24]. The mammogram coverage above 100% indicates that the number of performed mammograms was higher than necessary and the values were adjusted to 100% for statistical analyses.

For descriptive statistics, we used the standard deviation (SD) to represent the variation around the mean (or average) for normally distributed variables. The range (minimum and maximum values) or interquartile range (IQR) was used to represent the spread of the middle half (median) of the distribution in cases of non-normal distributed variables. The Mann-Kendall trend test was used to analyse trends in annual coverage (“Kendall” package in R). Differences in coverage and EBC detection rates across different states and municipalities were estimated using the χ^2^ test. The r correlation index was estimated using Pearson correlation test. GDP *per capita* values in Brazilian Real (R$) and mammogram coverage were categorized by quartiles named Q1, Q2, Q3, and Q4, and the χ^2^ test was used to analyse the distribution of the number of municipalities in the contingency table. All analyses were performed in the R environment (version 4.1.2, R Core Team, Austria). A *P*-value <0.05 was used as a cut-off for the significance of the statistical tests.

### Supplemental data and additional tools

All supplemental data, interactive maps, and plots can be accessed online at http://cancermamabrasil.com.br.

## RESULTS

### Mammogram coverage

**Figure 1:**
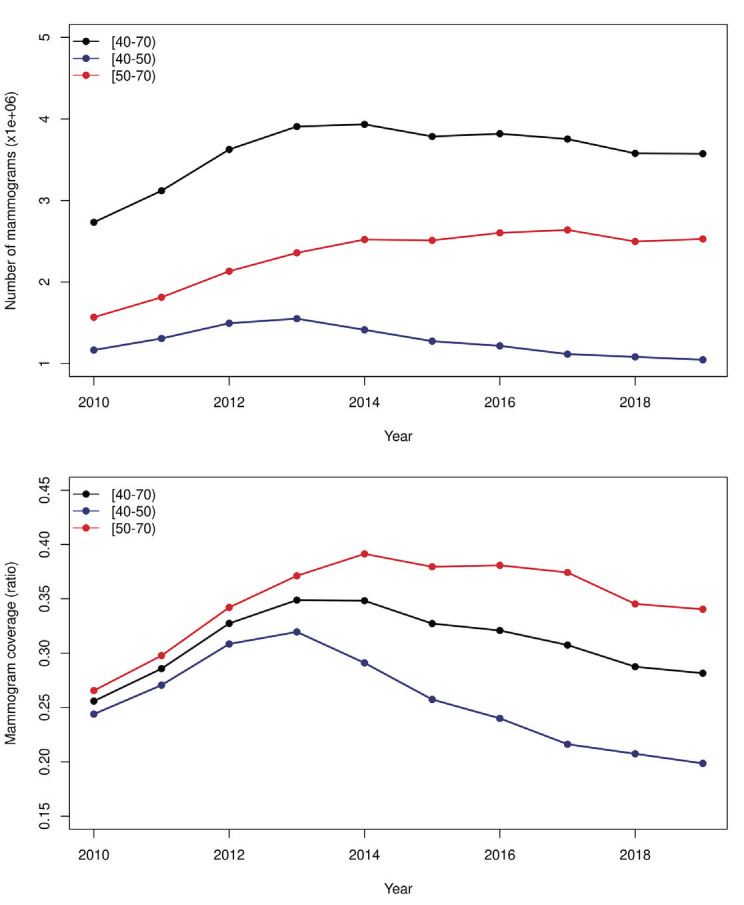
Total number of mammograms (**Panel A**) and mammogram coverage (**Panel B**) from 2010 to 2019 in Brazil in the female population aged 40 to 69 years.

A total of 39 555 636 mammograms with an average of 3 955 564 ± 395 704 were recorded per year from 2010 to 2019 in Brazil. This represented a total cost of 1.77 billion Brazilian reals. The total number of mammograms performed per year increased from 2010 to 2013, followed by a slight reduction (*P* = 0.8). As seen in **Figure 1**, the main reduction was in the number of mammograms performed in the population aged 40-50 years. When adjusted to the population estimates, the coverage significantly dropped in the population aged 40-50 years (*P* = 0.02) and was stable in the population aged 50-70 years (*P* = 0.4). Since the value paid per screening mammogram did not change from 2010 to 2019 (R$45.00/US$9.51), the investment was proportional to the total number of mammograms performed.

**Figure 2:**
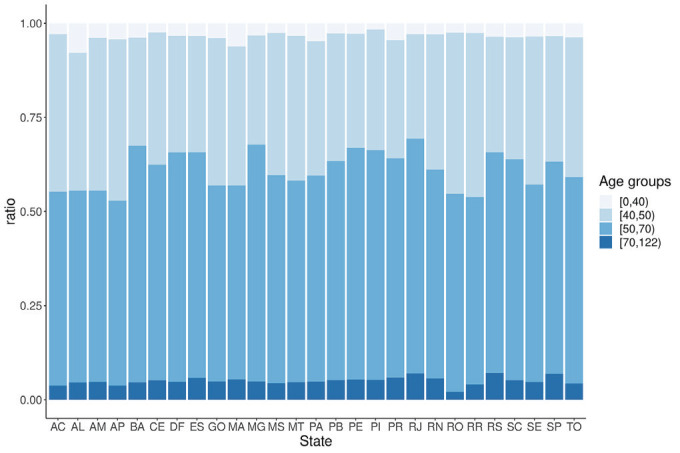
Distribution of screening mammograms according to age groups for every state in Brazil. AC – Acre, AL – Alagoas, AM – Amazonas, AP – Amapá, BA – Bahia, CE – Ceará, DF – Distrito Federal, ES – Espírito Santo, GO – Goiás, MA – Maranhão, MT – Mato Grosso, MS – Mato Grosso do Sul, MG – Minas Gerais, PA – Pará, PB – Paraíba, PR – Paraná, PE – Pernambuco, PI – Piauí, RJ – Rio de Janeiro, RN – Rio Grande do Norte, RO – Rondônia, RR – Roraima, RS – Rio Grande do Sul, SC – Santa Catarina, SE – Sergipe, SP – São Paulo, TO – Tocantins

The median age of the women who underwent a screening mammogram was 53 years (IQR = 13). Most examinations (58.6%) were performed in women aged 50-69 years, while 32% were performed in women aged 40-49 years, and 9.4% were performed in women aged <40 or >70 years. At the state level, the median percentage of mammograms performed in the population aged 50-70 years was 55.6% and ranged from 49.2% to 62.9%. This represents significant variation across the 27 states (*P* < 0.0001). All seven states from the south and southeast regions exceeded the national median in the number of mammograms performed in the population aged 50-70 years. Minas Gerais (62.9%), Bahia (62.9%), and Rio de Janeiro (62.4%) were the top-ranked states, and Amapá (49.2%), Roraima (49.8%), and Amazonas (50.8%) were the lowest-ranked states. **Figure 2** shows the distribution of the percentage of screening mammograms performed in every state according to age group.

The overall Brazilian mammogram coverage was 35% for women aged 50–69 years and 25.4% for women aged 40-50 years. We also observed significant variation in the mean mammogram coverage in the populations aged 40-50 and 50-70 years across the states (*P* < 0.0001). The highest mean mammogram coverage in the populations aged 50-70 and 40-50 years was observed in São Paulo (52.5% and 44.7%, respectively), Paraná (46.6% and 34%, respectively), and Minas Gerais (44.5% and 28.9%, respectively), and the lowest mean was observed in Amapá (2.8% and 2.1%, respectively), Pará (10.8% and 7.6%, respectively), and Maranhão (10.9% and 9.6%, respectively). Table S1 in the **Online Supplementary Document** summarizes the number and percentage of screening mammograms for each state.

### Detection rate of early breast cancer

**Figure 3:**
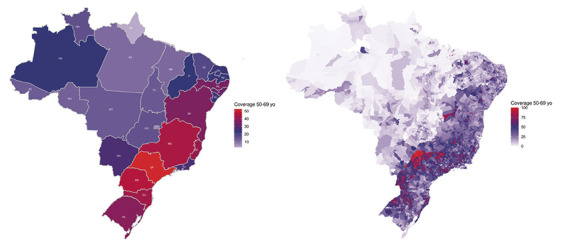
Distribution of mammogram coverage in women from 50 to 69 years old; **Panel A** – across all states in Brazil, **Panel B** – municipalities in Brazil.

The overall EBC detection rates in Brazil were 30.8% among populations aged 50-70 years and 25.4% among those aged 50-70. The highest EBC detection rates among the Brazilian states in the populations aged 50-70 and 40-50 years were in Santa Catarina (36.3% and 31.3%, respectively), São Paulo (36% and 30.4%, respectively), and Minas Gerais (34.9% and 28.6%, respectively), and the lowest EBC detection rates were in Pará (11.4% and 7%, respectively), Tocantins (12% and 8.2%, respectively), and Acre (13.7% and 9.6%, respectively). We observed a positive correlation between the coverage and the EBC detection rate in the populations aged 50-70 (r = 0.67; 95% confidence interval (CI) = 0.39-0.83, *P* = 0.0001) and 40-50 years (r = 0.67; 95% CI = 0.39-0.83, *P* < 0.0001) across the Brazilian states. **Figure 3** illustrates the distribution of mammogram coverage in the population aged 50-69 years among states a) municipalities b) in Brazil.

### Mammogram coverage and EBC detection rate at the municipality level

**Figure 4:**
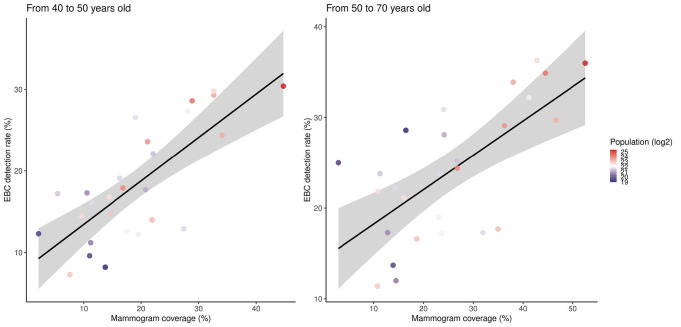
Correlation between mammogram coverage and the early breast cancer (EBC) detection rate across all Brazilian states.

**Figure 5:**
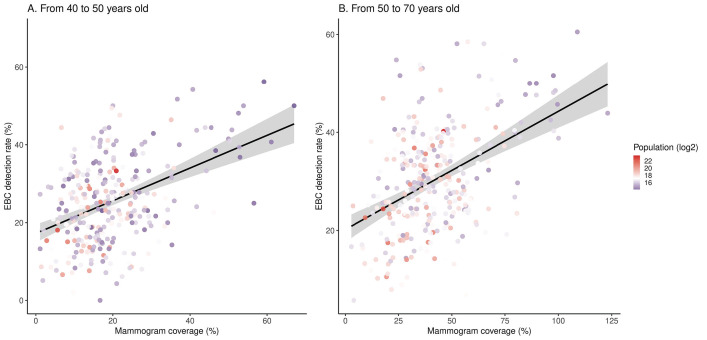
Correlation between mammogram coverage and the early breast cancer (EBC) detection rate across Brazilian counties.

We estimated mammogram coverage for 5549 municipalities in Brazil. The coverage ranged from 0.1% to 207.2% in the population aged 50-70 years, and from 0% to 111.5% in the population aged 40-50 years. There were 124 municipalities with an estimated coverage higher than 100% in the population aged 50-70 years. All these municipalities had a small population (median = 3004, range = 827.3-48,394). A total of 435 municipalities had over 70% coverage in the population aged 50-70 years. Most municipalities (n = 424) were located in the country’s south and southeast regions, and 214 were located in the state of São Paulo. **Figure 4** and **Figure 5** show the relationship between coverage and the EBC detection rate.

The EBC detection ratio was estimated in many municipalities, with at least 30 reported breast cancer cases diagnosed in women aged 50-69 years in 301 municipalities and women aged 40-49 years in 204 municipalities during the 10-year observation time. The median EBC detection ratio was 29.7% (IQR = 14) in the population aged 50-70 years and 25% (IQR = 14.7) in the population aged 40-50 years. Positive correlations between coverage and the EBC detection rate across these 591 municipalities were observed in the populations aged 50-70 (r = 0.45; 95% CI 95% = 0.35-0.53, *P* < 0.0001) and 40-50 years (r = 0.42; 95% CI = 0.32-0.51, *P* < 0.0001). Higher coverage and EBC detection ratios were present in municipalities with smaller populations.

### Gross domestic product (GDP), population density, and mammogram coverage

**Figure 6:**
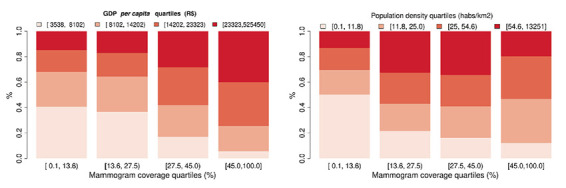
Gross domestic product (GDP) *per capita* quartile (**Panel A**) and population density quartile (**Panel B**) distribution according to the mammogram coverage quartiles.

We investigated the association between a municipality's GDP *per capita* and mammogram coverage. The median municipality GDP *per capita* in Brazil was R$14 202.00/US$3001.14 (IQR = 15 218.96). We observed a strong positive association between GDP *per capita* and mammogram coverage (*P* < 0.0001). **Figure 6** shows the distribution of municipalities according to each quartile, based on GDP and coverage. Note the predominant number of municipalities in the GDP *per capita* Q3 and Q4 groups associated with the upper quartile groups based on coverage.

We observed a high concentration of low-density municipalities in the lower coverage quartile, and most of the municipalities in the upper coverage quartile were represented by the inner quartiles (Q2 and Q3). This difference in the population density distribution was significant (*P* < 0.0001).

## DISCUSSION

The Brazilian National Health Service provides full public health coverage for all Brazilian citizens and recommends biennial screening mammograms for all women aged 50-69 years, according to the 2015 guidelines [10]. According to the World Health Organization (WHO), at least 70% of the target population must have access to screening mammograms to significantly reduce breast cancer mortality rates [25]. Our data demonstrate that the Brazilian screening program did not meet the minimum desired coverage. Although there was an evident increase in coverage from 2010 to 2014, the investment in breast cancer screening did not follow Brazilian population growth, and a substantial percentage of the investment was spent to screen the non-target population. Areas with low GDP *per capita* and low population density are the most affected, demonstrating the impact of the social and economic inequalities in breast cancer screening in Brazil.

Rodrigues et al. [9], based on 2016 data, reported on Brazil’s limited capacity to screen the target population. Although they stated that the low coverage was not due to the number of mammography machines available or the travel distance to undergo a screening mammogram, we can observe in their manuscript that some states within the most populated regions in Brazil, such as São Paulo, Rio de Janeiro, and Paraná, had an insufficient number of mammography machines. Interestingly, some states had more mammography machines than required, which did not result in better coverage. For example, Paraíba and Amazonia had very low mammography screening coverage (10.8% and 23%, respectively), despite having 3 and 2.4 times more machines than needed, respectively. Their observations support a multifaceted mechanism involved in the failure of the breast cancer screening program, where the low productivity of the mammography machines seems to be more important than their number or location. Our analyses brought up new insights to resolve these problems.

Overall, we observed that 41.4% of screening mammograms were performed in a non-target Brazilian population. This percentage varied across the 27 states in Brazil, and all the states with mammograms performed in the non-target population below the median were in the North, Northeast, and Central-West regions. Most mammograms not performed in the target women aged 50-70 years were performed in women aged 40-49 years (32%). The 2015 guidelines for breast cancer screening in Brazil recommend against screening for this age group [10]. This may explain the significant decrease in the number of mammograms performed in the population aged 40-50 years after 2015. If all mammograms performed in women aged 40-49 years had been performed in women aged 50-70 years, the estimated coverage would be greater than 50% in nine Brazilian states (Pernambuco = 52.2%, Bahia = 52.8%, Alagoas = 55%, Rio Grande do Sul = 57.9%, Espirito Santo = 62.3%, Minas Gerais = 65%, Santa Catarina = 66.5%, Paraná = 71.6%, and São Paulo = 83.6%), with six of these states being located in the south and southeast regions. The population covered by these nine states represented 63.1% of the target population in Brazil, and 67% of new breast cancer diagnoses were performed in women living in these nine states in the previous years [26].

We estimated EBC detection rates in the Brazilian population. As previously reported [27,28], the EBC detection rate was low (29.7%). This may be explained in part by the low screening coverage. However, the EBC detection rate is still significantly lower than the ratio reported by some developed countries before the implementation of a breast cancer screening program [29]. In fact, a recent report demonstrating access to the health system in Brazil showed that 54.3% of privately insured women were diagnosed with EBC (stages 0 and I) compared to 29.8% of publicly insured patients [11]. Other factors that may contribute to more advanced stages of breast cancer at presentation in publicly insured women are the delay between mammography and imaging interpretation [28], long intervals between mammography or first symptoms to biopsy and initiation of treatment [30], and difficulties in accessing optimal treatment [31].

When stratified by regions and municipalities, we observed significant heterogeneity in the number of mammograms performed and the consequent coverage, with the south and southeast areas of the country being the regions with higher coverage and EBC detection rates. Additionally, we found a significant association between GDP *per capita* and mammogram coverage. This is an interesting observation and may reflect the higher socioeconomic and educational status of these regions, as well as the inequalities and distance to access health care services across Brazilian states and municipalities [9,27,32].

Additionally, we observed that half of the regions with the lowest population density had the lowest mammogram coverage. This may reflect the lack of providing and maintaining mammographic services in these areas or the difficulty of travelling considerable distances to undergo periodic examinations [33]. Interestingly, the regions with the highest population density did not have the highest coverage rates. While most of the mammographic machines and investments in public health are concentrated in densely populated areas, people living in the suburban periphery of these metropolitan areas are associated with lower socioeconomic status, crowded housing conditions, and unemployment. These factors are predictors of lower mammographic screening coverage rates [34].

Our study had some limitations. Because the data were obtained from online databases and secondary information, the analysis was restricted to the available information, some of which may have been underreported, such as data from the cancer database registry. A large proportion of the cases registered in the INCA databases do not contain information about the disease stage and had to be excluded to determine the EBC ratio. Additionally, as with any ecological study, our data are based on population aggregates and may not be generalizable to an individual level. However, the current study is a comprehensive analysis that gathered massive amounts of data from the Brazilian population, and we believe that our results, which will become publicly available, are an important source of information that can be accessed by public authorities to influence decision-making policies for breast cancer screening.

## CONCLUSIONS

As mammographic screening has been described as an effective method for EBC detection and mortality reduction, the present study demonstrated that the coverage of mammographic screening for women under the SUS is far below the WHO recommendations. Additionally, a significant number of mammograms were performed outside the age group recommended by the Ministry of Health, with resources that could have been reallocated to screen the target population. This scenario is an integral part of problematic screening programs in developing countries and contributes to low rates of EBC diagnosis. As a continental country with heterogeneous socioeconomic indicators, we observed an important variation in the number of mammograms performed by age groups in Brazil when separated by states and municipalities. Even adjusting the coverage by the privately insured women, municipalities with higher GDP *per capita* were associated with higher mammogram coverage. This study provides evidence for decision makers in public health to monitor and act to improve inequalities in the different regions in Brazil.

## Additional material


Online Supplementary Document
