# Supplementary material for: 10-year opportunistic mammographic screening scenario in Brazil and its impact on breast cancer early detection: a nationwide population-based study
Source: J Glob Health. 2022 Oct 14;12:04061. doi: 10.7189/jogh.12.04061 (PMC9564571; doi:10.7189/jogh.12.04061)
Supplement: Online Supplementary Document [file jogh-12-04061-s001.zip › Supplemental_table_municipality_data.doc]

| uf ibge_uf_idname_uf | | name_munic ibge_munic_id6ibge_munic_idavg_populationavg_pop_50_69avg_pop_40_49avg_pop_50_69_ans_adjavg_pop_40_49_ans_adjtotal_number_mmg_50_69total_number_mmg_40 | | | | | | | | | |
| --- | --- | --- | --- | --- | --- | --- | --- | --- | --- | --- | --- |
| 1RO | 11Rondônia | Alta Floresta  D'Oeste | 110001 | 1100015 | 24670.2 | 3459 | 3236.2 | 2838.5 | 2794.5 | 877 |  |
| 2RO | 11Rondônia | Ariquemes | 110002 | 1100023 | 101047.2 | 14167.8 | 13255.4 | 11626.1 | 11446 | 11688 |  |
| 3RO | 11Rondônia | Cabixi | 110003 | 1100031 | 6120.3 | 858.1 | 802.9 | 704.2 | 693.3 | 891 |  |
| 4RO | 11Rondônia | Cacoal | 110004 | 1100049 | 84306.3 | 11820.6 | 11059.3 | 9700 | 9549.7 | 1940 |  |
| 5RO | 11Rondônia | Cerejeiras | 110005 | 1100056 | 17352 | 2432.9 | 2276.2 | 1996.5 | 1965.5 | 1725 |  |
| 6RO | 11Rondônia | Colorado do Oeste | 110006 | 1100064 | 18124.5 | 2541.2 | 2377.6 | 2085.3 | 2053 | 1592 |  |
| 7RO | 11Rondônia | Corumbiara | 110007 | 1100072 | 8514.9 | 1193.9 | 1117 | 979.7 | 964.5 | 354 |  |
| 8RO | 11Rondônia | Costa Marques | 110008 | 1100080 | 16143.4 | 2263.5 | 2117.7 | 1857.4 | 1828.6 | 87 |  |
| 9RO | 11Rondônia | Espigão D'Oeste | 110009 | 1100098 | 31317.4 | 4391 | 4108.2 | 3603.3 | 3547.4 | 754 |  |
| 10RO | 11Rondônia | Guajará-Mirim | 110010 | 1100106 | 45084.3 | 6321.3 | 5914.2 | 5187.2 | 5106.9 | 2050 |  |
| 11RO | 11Rondônia | Jaru | 110011 | 1100114 | 53804.2 | 7543.9 | 7058 | 6190.5 | 6094.6 | 2446 |  |
| 12RO | 11Rondônia | Ji-Paraná | 110012 | 1100122 | 126085.5 | 17678.4 | 16539.9 | 14506.9 | 14282.2 | 6132 |  |
| 13RO | 11Rondônia | Machadinho  D'Oeste | 110013 | 1100130 | 36012.5 | 5049.3 | 4724.1 | 4143.5 | 4079.3 | 773 |  |
| 14RO | 11Rondônia | Nova Brasilândia  D'Oeste | 110014 | 1100148 | 20852.7 | 2923.8 | 2735.5 | 2399.2 | 2362.1 | 235 |  |
| 15RO | 11Rondônia | Ouro Preto do  Oeste | 110015 | 1100155 | 38511.8 | 5399.7 | 5052 | 4431 | 4362.4 | 963 |  |
| 16RO | 11Rondônia | Pimenta Bueno | 110018 | 1100189 | 36255 | 5083.3 | 4755.9 | 4171.4 | 4106.7 | 587 |  |
| 17RO | 11Rondônia | Porto Velho | 110020 | 1100205 | 486844.3 | 68260.4 | 63864.2 | 56014.5 | 55146.8 | 58794 | 26 |
| 18RO | 11Rondônia | Presidente Médici | 110025 | 1100254 | 21724.9 | 3046 | 2849.9 | 2499.6 | 2460.9 | 682 |  |
| 19RO | 11Rondônia | Rio Crespo | 110026 | 1100262 | 3626.6 | 508.5 | 475.7 | 417.3 | 410.8 | 448 |  |
| 20RO | 11Rondônia | Rolim de Moura | 110028 | 1100288 | 54359.3 | 7621.7 | 7130.9 | 6254.4 | 6157.5 | 1772 |  |
| 21RO | 11Rondônia | Santa Luzia  D'Oeste | 110029 | 1100296 | 8200 | 1149.7 | 1075.7 | 943.5 | 928.8 | 324 |  |
| 22RO | 11Rondônia | Vilhena | 110030 | 1100304 | 88975.7 | 12475.3 | 11671.8 | 10237.2 | 10078.6 | 5312 |  |
| 23RO | 11Rondônia | São Miguel do  Guaporé | 110032 | 1100320 | 23121.3 | 3241.8 | 3033.1 | 2660.3 | 2619 | 333 |  |
| 24RO | 11Rondônia | Nova Mamoré | 110033 | 1100338 | 26764.5 | 3752.7 | 3511 | 3079.4 | 3031.7 | 1237 |  |
| 25RO | 11Rondônia | Alvorada D'Oeste | 110034 | 1100346 | 16435.3 | 2304.4 | 2156 | 1891 | 1861.7 | 229 |  |
| 26RO | 11Rondônia | Alto Alegre dos  Parecis | 110037 | 1100379 | 13463 | 1887.6 | 1766.1 | 1549 | 1525 | 305 |  |
| 27RO | 11Rondônia | Alto Paraíso | 110040 | 1100403 | 19574.2 | 2744.5 | 2567.7 | 2252.1 | 2217.2 | 785 |  |
| 28RO | 11Rondônia | Buritis | 110045 | 1100452 | 36636.3 | 5136.8 | 4805.9 | 4215.2 | 4149.9 | 2029 |  |
| 29RO | 11Rondônia | Novo Horizonte do  Oeste | 110050 | 1100502 | 9894.2 | 1387.3 | 1297.9 | 1138.4 | 1120.8 | 195 |  |
| 30RO | 11Rondônia | Cacaulândia | 110060 | 1100601 | 6153.8 | 862.8 | 807.3 | 708 | 697.1 | 455 |  |
| 31RO | 11Rondônia | Campo Novo de  Rondônia | 110070 | 1100700 | 13749.5 | 1927.8 | 1803.7 | 1582 | 1557.5 | 780 |  |
| 32RO | 11Rondônia | Candeias do  Jamari | 110080 | 1100809 | 23421.9 | 3284 | 3072.5 | 2694.8 | 2653.1 | 2454 |  |
| 33RO | 11Rondônia | Castanheiras | 110090 | 1100908 | 3484.3 | 488.5 | 457.1 | 400.9 | 394.7 | 81 |  |
| 34RO | 11Rondônia | Chupinguaia | 110092 | 1100924 | 9821.3 | 1377 | 1288.4 | 1130 | 1112.5 | 234 |  |
| 35RO | 11Rondônia | Cujubim | 110094 | 1100940 | 20387.7 | 2858.6 | 2674.5 | 2345.7 | 2309.4 | 631 |  |
| 36RO | 11Rondônia | Governador Jorge  Teixeira | 110100 | 1101005 | 9735.2 | 1365 | 1277.1 | 1120.1 | 1102.7 | 395 |  |
| 37RO | 11Rondônia | Itapuã do Oeste | 110110 | 1101104 | 9677.8 | 1356.9 | 1269.5 | 1113.5 | 1096.2 | 872 |  |
| 38RO | 11Rondônia | Ministro  Andreazza | 110120 | 1101203 | 10437.2 | 1463.4 | 1369.2 | 1200.9 | 1182.3 | 281 |  |
| 39RO | 11Rondônia | Mirante da Serra | 110130 | 1101302 | 11918 | 1671 | 1563.4 | 1371.2 | 1350 | 190 |  |
| 40RO | 11Rondônia | Monte Negro | 110140 | 1101401 | 15349.6 | 2152.2 | 2013.6 | 1766.1 | 1738.7 | 1321 |  |
| 41RO | 11Rondônia | Nova União | 110143 | 1101435 | 7545.3 | 1057.9 | 989.8 | 868.1 | 854.7 | 103 |  |
| 42RO | 11Rondônia | Parecis | 110145 | 1101450 | 5519.1 | 773.8 | 724 | 635 | 625.2 | 98 |  |
| 43RO | 11Rondônia | Pimenteiras do  Oeste | 110146 | 1101468 | 2338 | 327.8 | 306.7 | 269 | 264.8 | 172 |  |
| 44RO | 11Rondônia | Primavera de  Rondônia | 110147 | 1101476 | 3370.2 | 472.5 | 442.1 | 387.8 | 381.8 | 158 |  |
| 45RO | 11Rondônia | São Felipe  D'Oeste | 110148 | 1101484 | 5879.4 | 824.4 | 771.3 | 676.5 | 666 | 220 |  |
| 46RO | 11Rondônia | São Francisco do  Guaporé | 110149 | 1101492 | 18407.3 | 2580.9 | 2414.7 | 2117.9 | 2085.1 | 173 |  |
| 47RO | 11Rondônia | Seringueiras | 110150 | 1101500 | 12148.7 | 1703.4 | 1593.7 | 1397.8 | 1376.1 | 136 |  |
| 48RO | 11Rondônia | Teixeirópolis | 110155 | 1101559 | 4821.1 | 676 | 632.4 | 554.7 | 546.1 | 95 |  |
| 49RO | 11Rondônia | Theobroma | 110160 | 1101609 | 10950.6 | 1535.4 | 1436.5 | 1259.9 | 1240.4 | 376 |  |
| 50RO | 11Rondônia | Urupá | 110170 | 1101708 | 12810 | 1796.1 | 1680.4 | 1473.9 | 1451 | 167 |  |
| 51RO | 11Rondônia | Vale do Anari | 110175 | 1101757 | 10495 | 1471.5 | 1376.7 | 1207.5 | 1188.8 | 168 |  |
| 52RO | 11Rondônia | Vale do Paraíso | 110180 | 1101807 | 7924.5 | 1111.1 | 1039.5 | 911.8 | 897.6 | 346 |  |
| 53AC | 12Acre | Acrelândia | 120001 | 1200013 | 13792.4 | 1490.7 | 1450.8 | 1281.7 | 1312.1 | 1409 |  |
| 54AC | 12Acre | Assis Brasil | 120005 | 1200054 | 6696.6 | 723.8 | 704.4 | 622.3 | 637.1 | 429 |  |
| 55AC | 12Acre | Brasiléia | 120010 | 1200104 | 23682.4 | 2559.6 | 2491.2 | 2200.7 | 2253 | 1114 |  |
| 56AC | 12Acre | Bujari | 120013 | 1200138 | 9294.1 | 1004.5 | 977.6 | 863.7 | 884.2 | 613 |  |
| 57AC | 12Acre | Capixaba | 120017 | 1200179 | 10290.2 | 1112.2 | 1082.4 | 956.2 | 978.9 | 813 |  |
| 58AC | 12Acre | Cruzeiro do Sul | 120020 | 1200203 | 82109.4 | 8874.4 | 8637.1 | 7630.2 | 7811.4 | 2753 |  |
| 59AC | 12Acre | Epitaciolândia | 120025 | 1200252 | 16633.1 | 1797.7 | 1749.6 | 1545.7 | 1582.4 | 714 |  |
| 60AC | 12Acre | Feijó | 120030 | 1200302 | 32884 | 3554.1 | 3459.1 | 3055.8 | 3128.4 | 342 |  |
| 61AC | 12Acre | Jordão | 120032 | 1200328 | 7422 | 802.2 | 780.7 | 689.7 | 706.1 | 46 |  |
| 62AC | 12Acre | Mâncio Lima | 120033 | 1200336 | 17009.7 | 1838.4 | 1789.3 | 1580.7 | 1618.2 | 327 |  |
| 63AC | 12Acre | Manoel Urbano | 120034 | 1200344 | 8629.6 | 932.7 | 907.7 | 801.9 | 821 | 208 |  |
| 64AC | 12Acre | Marechal  Thaumaturgo | 120035 | 1200351 | 16575.9 | 1791.5 | 1743.6 | 1540.4 | 1576.9 | 191 |  |
| 65AC | 12Acre | Plácido de Castro | 120038 | 1200385 | 18230.2 | 1970.3 | 1917.6 | 1694.1 | 1734.3 | 1286 |  |
| 66AC | 12Acre | Porto Walter | 120039 | 1200393 | 10580.4 | 1143.5 | 1113 | 983.2 | 1006.6 | 146 |  |
| 67AC | 12Acre | Rio Branco | 120040 | 1200401 | 368733.6 | 39852.7 | 38787.1 | 34265.4 | 35079 | 34605 | 14 |
| 68AC | 12Acre | Rodrigues Alves | 120042 | 1200427 | 16674.1 | 1802.1 | 1753.9 | 1549.5 | 1586.3 | 328 |  |
| 69AC | 12Acre | Santa Rosa do  Purus | 120043 | 1200435 | 5656 | 611.3 | 595 | 525.6 | 538.1 | 500 |  |
| 70AC | 12Acre | Senador  Guiomard | 120045 | 1200450 | 21288.1 | 2300.8 | 2239.3 | 1978.2 | 2025.2 | 1496 |  |
| 71AC | 12Acre | Sena Madureira | 120050 | 1200500 | 41581.5 | 4494.1 | 4374 | 3864.1 | 3955.8 | 1069 |  |
| 72AC | 12Acre | Tarauacá | 120060 | 1200609 | 38712.4 | 4184 | 4072.2 | 3597.4 | 3682.9 | 489 |  |
| 73AC | 12Acre | Xapuri | 120070 | 1200708 | 17548.4 | 1896.6 | 1845.9 | 1630.7 | 1669.4 | 699 |  |
| 74AC | 12Acre | Porto Acre | 120080 | 1200807 | 16606.2 | 1794.8 | 1746.8 | 1543.2 | 1579.8 | 1678 |  |
| 75AM | 13Amazonas | Alvarães | 130002 | 1300029 | 15231.2 | 1682 | 1646.3 | 1387.6 | 1312.3 | 126 |  |
| 76AM | 13Amazonas | Amaturá | 130006 | 1300060 | 10597.8 | 1170.3 | 1145.5 | 965.5 | 913.1 | 114 |  |
| 77AM | 13Amazonas | Anamã | 130008 | 1300086 | 11992.5 | 1324.3 | 1296.3 | 1092.6 | 1033.3 | 103 |  |
| 78AM | 13Amazonas | Anori | 130010 | 1300102 | 18805.2 | 2076.7 | 2032.7 | 1713.2 | 1620.2 | 331 |  |
| 79AM | 13Amazonas | Apuí | 130014 | 1300144 | 20172.4 | 2227.6 | 2180.4 | 1837.8 | 1738 | 552 |  |
| 80AM | 13Amazonas | Atalaia do Norte | 130020 | 1300201 | 17659.9 | 1950.2 | 1908.9 | 1608.9 | 1521.6 | 125 |  |
| 81AM | 13Amazonas | Autazes | 130030 | 1300300 | 36166.9 | 3993.9 | 3909.3 | 3295 | 3116.1 | 189 |  |
| 82AM | 13Amazonas | Barcelos | 130040 | 1300409 | 26951.5 | 2976.3 | 2913.2 | 2455.4 | 2322.1 | 44 |  |
| 83AM | 13Amazonas | Barreirinha | 130050 | 1300508 | 30003.2 | 3313.3 | 3243 | 2733.4 | 2585 | 24 |  |
| 84AM | 13Amazonas | Benjamin Constant | 130060 | 1300607 | 38488.5 | 4250.3 | 4160.2 | 3506.5 | 3316.1 | 54 |  |
| 85AM | 13Amazonas | Beruri | 130063 | 1300631 | 17722.3 | 1957.1 | 1915.6 | 1614.6 | 1526.9 | 69 |  |
| 86AM | 13Amazonas | Boa Vista do  Ramos | 130068 | 1300680 | 17224.9 | 1902.1 | 1861.8 | 1569.3 | 1484.1 | 33 |  |
| 87AM | 13Amazonas | Boca do Acre | 130070 | 1300706 | 32844.7 | 3627 | 3550.2 | 2992.3 | 2829.9 | 327 |  |
| 88AM | 13Amazonas | Borba | 130080 | 1300805 | 38445.6 | 4245.5 | 4155.6 | 3502.6 | 3312.4 | 106 |  |
| 89AM | 13Amazonas | Caapiranga | 130083 | 1300839 | 12145.8 | 1341.3 | 1312.8 | 1106.5 | 1046.5 | 67 |  |
| 90AM | 13Amazonas | Canutama | 130090 | 1300904 | 14632.5 | 1615.9 | 1581.6 | 1333.1 | 1260.7 | 50 |  |
| 91AM | 13Amazonas | Carauari | 130100 | 1301001 | 27360.7 | 3021.4 | 2957.4 | 2492.7 | 2357.4 | 66 |  |
| 92AM | 13Amazonas | Careiro | 130110 | 1301100 | 35676.1 | 3939.7 | 3856.2 | 3250.3 | 3073.8 | 295 |  |
| 93AM | 13Amazonas | Careiro da Várzea | 130115 | 1301159 | 27297 | 3014.4 | 2950.5 | 2486.9 | 2351.9 | 313 |  |
| 94AM | 13Amazonas | Coari | 130120 | 1301209 | 81458.8 | 8995.5 | 8804.9 | 7421.3 | 7018.4 | 360 |  |
| 95AM | 13Amazonas | Codajás | 130130 | 1301308 | 26148.8 | 2887.6 | 2826.4 | 2382.3 | 2252.9 | 68 |  |

4

1

1

2

1

1

| 96AM | 13Amazonas | Eirunepé | 130140 | 1301407 | 33324.3 | 3680 | 3602 | 3036 | 2871.2 | 88 |  |
| --- | --- | --- | --- | --- | --- | --- | --- | --- | --- | --- | --- |
| 97AM | 13Amazonas | Envira | 130150 | 1301506 | 18349.1 | 2026.3 | 1983.4 | 1671.7 | 1580.9 | 36 |  |
| 98AM | 13Amazonas | Fonte Boa | 130160 | 1301605 | 20862 | 2303.8 | 2255 | 1900.6 | 1797.4 | 65 |  |
| 99AM | 13Amazonas | Guajará | 130165 | 1301654 | 15475.4 | 1708.9 | 1672.7 | 1409.9 | 1333.3 | 39 |  |
| 100AM | 13Amazonas | Humaitá | 130170 | 1301704 | 50077.2 | 5530 | 5412.8 | 4562.3 | 4314.6 | 927 |  |
| 101AM | 13Amazonas | Ipixuna | 130180 | 1301803 | 26142.9 | 2887 | 2825.8 | 2381.7 | 2252.4 | 66 |  |
| 102AM | 13Amazonas | Iranduba | 130185 | 1301852 | 44981.5 | 4967.3 | 4862.1 | 4098 | 3875.5 | 825 |  |
| 103AM | 13Amazonas | Itacoatiara | 130190 | 1301902 | 95063.6 | 10497.9 | 10275.4 | 8660.7 | 8190.5 | 949 |  |
| 104AM | 13Amazonas | Itamarati | 130195 | 1301951 | 8066.7 | 890.8 | 871.9 | 734.9 | 695 | 11 |  |
| 105AM | 13Amazonas | Itapiranga | 130200 | 1302009 | 8780.7 | 969.7 | 949.1 | 800 | 756.5 | 78 |  |
| 106AM | 13Amazonas | Japurá | 130210 | 1302108 | 5385.4 | 594.7 | 582.1 | 490.6 | 464 | 15 |  |
| 107AM | 13Amazonas | Juruá | 130220 | 1302207 | 12834.1 | 1417.3 | 1387.2 | 1169.2 | 1105.8 | 38 |  |
| 108AM | 13Amazonas | Jutaí | 130230 | 1302306 | 16646.2 | 1838.2 | 1799.3 | 1516.5 | 1434.2 | 37 |  |
| 109AM | 13Amazonas | Lábrea | 130240 | 1302405 | 42264.3 | 4667.2 | 4568.3 | 3850.5 | 3641.4 | 109 |  |
| 110AM | 13Amazonas | Manacapuru | 130250 | 1302504 | 92257.3 | 10188 | 9972.1 | 8405.1 | 7948.8 | 2492 |  |
| 111AM | 13Amazonas | Manaquiri | 130255 | 1302553 | 27592.5 | 3047 | 2982.5 | 2513.8 | 2377.3 | 605 |  |
| 112AM | 13Amazonas | Manaus | 130260 | 1302603 | 2010932.6 | 222067.3 | 217361.7 | 183205.5 | 173259 | 377611 | 152 |
| 113AM | 13Amazonas | Manicoré | 130270 | 1302702 | 51893.6 | 5730.6 | 5609.2 | 4727.8 | 4471.1 | 307 |  |
| 114AM | 13Amazonas | Maraã | 130280 | 1302801 | 18120.4 | 2001 | 1958.6 | 1650.9 | 1561.2 | 29 |  |
| 115AM | 13Amazonas | Maués | 130290 | 1302900 | 58594.9 | 6470.6 | 6333.5 | 5338.3 | 5048.5 | 435 |  |
| 116AM | 13Amazonas | Nhamundá | 130300 | 1303007 | 19933.5 | 2201.3 | 2154.6 | 1816 | 1717.4 | 67 |  |
| 117AM | 13Amazonas | Nova Olinda do  Norte | 130310 | 1303106 | 34348.9 | 3793.1 | 3712.8 | 3129.3 | 2959.5 | 96 |  |
| 118AM | 13Amazonas | Novo Airão | 130320 | 1303205 | 17206 | 1900.1 | 1859.8 | 1567.5 | 1482.4 | 115 |  |
| 119AM | 13Amazonas | Novo Aripuanã | 130330 | 1303304 | 23775.8 | 2625.6 | 2569.9 | 2166.1 | 2048.5 | 24 |  |
| 120AM | 13Amazonas | Parintins | 130340 | 1303403 | 109400.6 | 12081.1 | 11825.1 | 9966.9 | 9425.8 | 175 |  |
| 121AM | 13Amazonas | Pauini | 130350 | 1303502 | 19037.5 | 2102.3 | 2057.8 | 1734.4 | 1640.2 | 44 |  |
| 122AM | 13Amazonas | Presidente  Figueiredo | 130353 | 1303536 | 31935.4 | 3526.6 | 3451.9 | 2909.5 | 2751.5 | 981 |  |
| 123AM | 13Amazonas | Rio Preto da Eva | 130356 | 1303569 | 29751 | 3285.4 | 3215.8 | 2710.5 | 2563.3 | 868 |  |
| 124AM | 13Amazonas | Santa Isabel do  Rio Negro | 130360 | 1303601 | 21770.7 | 2404.1 | 2353.2 | 1983.4 | 1875.7 | 13 |  |
| 125AM | 13Amazonas | Santo Antônio do  Içá | 130370 | 1303700 | 23610.1 | 2607.3 | 2552 | 2151 | 2034.2 | 31 |  |
| 126AM | 13Amazonas | São Gabriel da  Cachoeira | 130380 | 1303809 | 42127.4 | 4652.1 | 4553.6 | 3838 | 3629.6 | 57 |  |
| 127AM | 13Amazonas | São Paulo de  Olivença | 130390 | 1303908 | 35657.6 | 3937.7 | 3854.2 | 3248.6 | 3072.2 | 71 |  |
| 128AM | 13Amazonas | São Sebastião do  Uatumã | 130395 | 1303957 | 12450.1 | 1374.9 | 1345.7 | 1134.3 | 1072.7 | 34 |  |
| 129AM | 13Amazonas | Silves | 130400 | 1304005 | 8916.3 | 984.6 | 963.8 | 812.3 | 768.2 | 33 |  |
| 130AM | 13Amazonas | Tabatinga | 130406 | 1304062 | 59542.5 | 6575.3 | 6435.9 | 5424.6 | 5130.1 | 101 |  |
| 131AM | 13Amazonas | Tapauá | 130410 | 1304104 | 18116.5 | 2000.6 | 1958.2 | 1650.5 | 1560.9 | 126 |  |
| 132AM | 13Amazonas | Tefé | 130420 | 1304203 | 61592 | 6801.6 | 6657.5 | 5611.3 | 5306.7 | 2688 |  |
| 133AM | 13Amazonas | Tonantins | 130423 | 1304237 | 18133.5 | 2002.5 | 1960.1 | 1652 | 1562.4 | 37 |  |
| 134AM | 13Amazonas | Uarini | 130426 | 1304260 | 12856.3 | 1419.7 | 1389.6 | 1171.3 | 1107.7 | 26 |  |
| 135AM | 13Amazonas | Urucará | 130430 | 1304302 | 16945.8 | 1871.3 | 1831.7 | 1543.8 | 1460 | 91 |  |
| 136AM | 13Amazonas | Urucurituba | 130440 | 1304401 | 20603.3 | 2275.2 | 2227 | 1877.1 | 1775.2 | 87 |  |
| 137RR | 14Roraima | Amajari | 140002 | 1400027 | 10909.4 | 1176.4 | 1189 | 1022.1 | 1075.9 | 196 |  |
| 138RR | 14Roraima | Alto Alegre | 140005 | 1400050 | 16105.1 | 1736.6 | 1755.3 | 1508.9 | 1588.4 | 540 |  |
| 139RR | 14Roraima | Boa Vista | 140010 | 1400100 | 324964.9 | 35041 | 35417.9 | 30447.1 | 32049.7 | 34508 | 15 |
| 140RR | 14Roraima | Bonfim | 140015 | 1400159 | 11654.8 | 1256.7 | 1270.3 | 1092 | 1149.5 | 296 |  |
| 141RR | 14Roraima | Cantá | 140017 | 1400175 | 15983.2 | 1723.5 | 1742 | 1497.5 | 1576.3 | 564 |  |
| 142RR | 14Roraima | Caracaraí | 140020 | 1400209 | 20090.3 | 2166.3 | 2189.6 | 1882.3 | 1981.4 | 523 |  |
| 143RR | 14Roraima | Caroebe | 140023 | 1400233 | 9082.5 | 979.4 | 989.9 | 851 | 895.8 | 238 |  |
| 144RR | 14Roraima | Iracema | 140028 | 1400282 | 10210.7 | 1101 | 1112.9 | 956.7 | 1007 | 345 |  |
| 145RR | 14Roraima | Mucajaí | 140030 | 1400308 | 16244.2 | 1751.6 | 1770.5 | 1522 | 1602.1 | 841 |  |
| 146RR | 14Roraima | Normandia | 140040 | 1400407 | 10051.5 | 1083.9 | 1095.5 | 941.8 | 991.3 | 120 |  |
| 147RR | 14Roraima | Pacaraima | 140045 | 1400456 | 12458.1 | 1343.4 | 1357.8 | 1167.2 | 1228.7 | 289 |  |
| 148RR | 14Roraima | Rorainópolis | 140047 | 1400472 | 27049.8 | 2916.8 | 2948.2 | 2534.4 | 2667.8 | 584 |  |
| 149RR | 14Roraima | São João da  Baliza | 140050 | 1400506 | 7451.3 | 803.5 | 812.1 | 698.1 | 734.9 | 273 |  |
| 150RR | 14Roraima | São Luiz | 140060 | 1400605 | 7345 | 792 | 800.5 | 688.2 | 724.4 | 216 |  |
| 151RR | 14Roraima | Uiramutã | 140070 | 1400704 | 9401.9 | 1013.8 | 1024.7 | 880.9 | 927.3 | 30 |  |
| 152PA | 15Pará | Abaetetuba | 150010 | 1500107 | 149417.5 | 18269.3 | 16858.8 | 15644 | 14576.1 | 1525 |  |
| 153PA | 15Pará | Abel Figueiredo | 150013 | 1500131 | 7096.3 | 867.7 | 800.7 | 743 | 692.3 | 68 |  |
| 154PA | 15Pará | Acará | 150020 | 1500206 | 54245.7 | 6632.6 | 6120.5 | 5679.5 | 5291.8 | 818 |  |
| 155PA | 15Pará | Afuá | 150030 | 1500305 | 37139.1 | 4541 | 4190.4 | 3888.5 | 3623 | 22 |  |
| 156PA | 15Pará | Água Azul do  Norte | 150034 | 1500347 | 26200.7 | 3203.6 | 2956.2 | 2743.2 | 2556 | 465 |  |
| 157PA | 15Pará | Alenquer | 150040 | 1500404 | 54552.4 | 6670.1 | 6155.1 | 5711.6 | 5321.7 | 735 |  |
| 158PA | 15Pará | Almeirim | 150050 | 1500503 | 33589.3 | 4107 | 3789.9 | 3516.8 | 3276.7 | 199 |  |
| 159PA | 15Pará | Altamira | 150060 | 1500602 | 107157.2 | 13102.1 | 12090.5 | 11219.3 | 10453.5 | 5852 |  |
| 160PA | 15Pará | Anajás | 150070 | 1500701 | 27149.5 | 3319.6 | 3063.3 | 2842.5 | 2648.5 | 239 |  |
| 161PA | 15Pará | Ananindeua | 150080 | 1500800 | 501601.1 | 61330.8 | 56595.7 | 52517.5 | 48932.6 | 100143 | 29 |
| 162PA | 15Pará | Anapu | 150085 | 1500859 | 24613.2 | 3009.5 | 2777.1 | 2577 | 2401.1 | 582 |  |
| 163PA | 15Pará | Augusto Corrêa | 150090 | 1500909 | 43311.7 | 5295.7 | 4886.9 | 4534.7 | 4225.2 | 820 |  |
| 164PA | 15Pará | Aurora do Pará | 150095 | 1500958 | 29079.4 | 3555.5 | 3281 | 3044.6 | 2836.8 | 351 |  |
| 165PA | 15Pará | Aveiro | 150100 | 1501006 | 16014.6 | 1958.1 | 1806.9 | 1676.7 | 1562.3 | 59 |  |
| 166PA | 15Pará | Bagre | 150110 | 1501105 | 27591 | 3373.6 | 3113.1 | 2888.8 | 2691.6 | 360 |  |
| 167PA | 15Pará | Baião | 150120 | 1501204 | 42666.7 | 5216.9 | 4814.1 | 4467.2 | 4162.3 | 210 |  |
| 168PA | 15Pará | Bannach | 150125 | 1501253 | 3315.3 | 405.4 | 374.1 | 347.1 | 323.4 | 324 |  |
| 169PA | 15Pará | Barcarena | 150130 | 1501303 | 113328.8 | 13856.7 | 12786.9 | 11865.5 | 11055.5 | 7220 |  |
| 170PA | 15Pará | Belém | 150140 | 1501402 | 1438100.6 | 175836.6 | 162260.9 | 150568.8 | 140290.8 | 145932 | 48 |
| 171PA | 15Pará | Belterra | 150145 | 1501451 | 16986.5 | 2076.9 | 1916.6 | 1778.5 | 1657.1 | 466 |  |
| 172PA | 15Pará | Benevides | 150150 | 1501501 | 57601.5 | 7042.9 | 6499.2 | 6030.9 | 5619.2 | 3080 |  |
| 173PA | 15Pará | Bom Jesus do  Tocantins | 150157 | 1501576 | 16132.4 | 1972.5 | 1820.2 | 1689.1 | 1573.8 | 282 |  |
| 174PA | 15Pará | Bonito | 150160 | 1501600 | 15044.2 | 1839.5 | 1697.4 | 1575.1 | 1467.6 | 99 |  |
| 175PA | 15Pará | Bragança | 150170 | 1501709 | 120562.8 | 14741.2 | 13603.1 | 12622.9 | 11761.2 | 9592 |  |
| 176PA | 15Pará | Brasil Novo | 150172 | 1501725 | 15444.7 | 1888.4 | 1742.6 | 1617.1 | 1506.7 | 1003 |  |
| 177PA | 15Pará | Brejo Grande do  Araguaia | 150175 | 1501758 | 7285.3 | 890.8 | 822 | 762.8 | 710.7 | 77 |  |
| 178PA | 15Pará | Breu Branco | 150178 | 1501782 | 59866.7 | 7319.9 | 6754.8 | 6268 | 5840.2 | 492 |  |
| 179PA | 15Pará | Breves | 150180 | 1501808 | 97706.8 | 11946.6 | 11024.3 | 10229.9 | 9531.6 | 4013 |  |
| 180PA | 15Pará | Bujaru | 150190 | 1501907 | 27449.7 | 3356.3 | 3097.1 | 2874 | 2677.8 | 985 |  |
| 181PA | 15Pará | Cachoeira do Piriá | 150195 | 1501956 | 30539.6 | 3734.1 | 3445.8 | 3197.5 | 2979.2 | 55 |  |
| 182PA | 15Pará | Cachoeira do Arari | 150200 | 1502004 | 22180.9 | 2712.1 | 2502.7 | 2322.3 | 2163.8 | 126 |  |
| 183PA | 15Pará | Cametá | 150210 | 1502103 | 129631.4 | 15850 | 14626.3 | 13572.4 | 12645.9 | 532 |  |
| 184PA | 15Pará | Canaã dos  Carajás | 150215 | 1502152 | 32482 | 3971.6 | 3664.9 | 3400.9 | 3168.7 | 429 |  |
| 185PA | 15Pará | Capanema | 150220 | 1502202 | 66173.7 | 8091.1 | 7466.4 | 6928.4 | 6455.4 | 1388 |  |
| 186PA | 15Pará | Capitão Poço | 150230 | 1502301 | 52809.8 | 6457.1 | 5958.5 | 5529.2 | 5151.7 | 868 |  |
| 187PA | 15Pará | Castanhal | 150240 | 1502400 | 187575.8 | 22934.9 | 21164.2 | 19639.1 | 18298.5 | 17810 |  |
| 188PA | 15Pará | Chaves | 150250 | 1502509 | 22383.1 | 2736.8 | 2525.5 | 2343.5 | 2183.5 | 9 |  |
| 189PA | 15Pará | Colares | 150260 | 1502608 | 11684.2 | 1428.6 | 1318.3 | 1223.3 | 1139.8 | 343 |  |
| 190PA | 15Pará | Conceição do  Araguaia | 150270 | 1502707 | 46472.3 | 5682.2 | 5243.5 | 4865.6 | 4533.5 | 2089 |  |
| 191PA | 15Pará | Concórdia do Pará | 150275 | 1502756 | 30913.2 | 3779.8 | 3487.9 | 3236.6 | 3015.7 | 539 |  |
| 192PA | 15Pará | Cumaru do Norte | 150276 | 1502764 | 12112.6 | 1481 | 1366.7 | 1268.2 | 1181.6 | 268 |  |
| 193PA | 15Pará | Curionópolis | 150277 | 1502772 | 17910.2 | 2189.9 | 2020.8 | 1875.2 | 1747.2 | 124 |  |
| 194PA | 15Pará | Curralinho | 150280 | 1502806 | 31705.7 | 3876.7 | 3577.4 | 3319.6 | 3093 | 267 |  |
| 195PA | 15Pará | Curuá | 150285 | 1502855 | 13381.4 | 1636.1 | 1509.8 | 1401 | 1305.4 | 179 |  |

1

1

2

3

1

4

1

7

| 196PA | 15Pará | Curuçá | 150290 | 1502905 | 37323.6 | 4563.6 | 4211.2 | 3907.8 | 3641 | 622 |
| --- | --- | --- | --- | --- | --- | --- | --- | --- | --- | --- |
| 197PA | 15Pará | Dom Eliseu | 150293 | 1502939 | 55715.3 | 6812.3 | 6286.4 | 5833.4 | 5435.2 | 291 |
| 198PA | 15Pará | Eldorado dos  Carajás | 150295 | 1502954 | 32663.6 | 3993.8 | 3685.4 | 3419.9 | 3186.4 | 98 |
| 199PA | 15Pará | Faro | 150300 | 1503002 | 7531.5 | 920.9 | 849.8 | 788.5 | 734.7 | 21 |
| 200PA | 15Pará | Floresta do  Araguaia | 150304 | 1503044 | 19074.3 | 2332.2 | 2152.2 | 1997.1 | 1860.8 | 589 |
| 201PA | 15Pará | Garrafão do Norte | 150307 | 1503077 | 25400.1 | 3105.7 | 2865.9 | 2659.4 | 2477.9 | 262 |
| 202PA | 15Pará | Goianésia do Pará | 150309 | 1503093 | 36685.1 | 4485.5 | 4139.2 | 3840.9 | 3578.7 | 223 |
| 203PA | 15Pará | Gurupá | 150310 | 1503101 | 31295.1 | 3826.5 | 3531 | 3276.6 | 3052.9 | 241 |
| 204PA | 15Pará | Igarapé-Açu | 150320 | 1503200 | 37247.9 | 4554.3 | 4202.7 | 3899.8 | 3633.6 | 1116 |
| 205PA | 15Pará | Igarapé-Miri | 150330 | 1503309 | 60218.5 | 7362.9 | 6794.5 | 6304.9 | 5874.5 | 1223 |
| 206PA | 15Pará | Inhangapi | 150340 | 1503408 | 10915.2 | 1334.6 | 1231.6 | 1142.8 | 1064.8 | 343 |
| 207PA | 15Pará | Ipixuna do Pará | 150345 | 1503457 | 57515.4 | 7032.4 | 6489.5 | 6021.9 | 5610.8 | 1003 |
| 208PA | 15Pará | Irituia | 150350 | 1503507 | 31760.8 | 3883.4 | 3583.6 | 3325.3 | 3098.4 | 726 |
| 209PA | 15Pará | Itaituba | 150360 | 1503606 | 98767.1 | 12076.3 | 11143.9 | 10340.9 | 9635 | 329 |
| 210PA | 15Pará | Itupiranga | 150370 | 1503705 | 51933.8 | 6349.9 | 5859.7 | 5437.5 | 5066.3 | 186 |
| 211PA | 15Pará | Jacareacanga | 150375 | 1503754 | 26095.8 | 3190.7 | 2944.4 | 2732.2 | 2545.7 | 47 |
| 212PA | 15Pará | Jacundá | 150380 | 1503804 | 55404.8 | 6774.3 | 6251.3 | 5800.9 | 5404.9 | 728 |
| 213PA | 15Pará | Juruti | 150390 | 1503903 | 52946 | 6473.7 | 5973.9 | 5543.4 | 5165 | 304 |
| 214PA | 15Pará | Limoeiro do Ajuru | 150400 | 1504000 | 27059.4 | 3308.6 | 3053.1 | 2833.1 | 2639.7 | 130 |
| 215PA | 15Pará | Mãe do Rio | 150405 | 1504059 | 28905.5 | 3534.3 | 3261.4 | 3026.4 | 2819.8 | 783 |
| 216PA | 15Pará | Magalhães Barata | 150410 | 1504109 | 8290.5 | 1013.7 | 935.4 | 868 | 808.8 | 160 |
| 217PA | 15Pará | Marabá | 150420 | 1504208 | 257995.3 | 31545.1 | 29109.6 | 27012.1 | 25168.2 | 11451 |
| 218PA | 15Pará | Maracanã | 150430 | 1504307 | 28749.1 | 3515.2 | 3243.8 | 3010 | 2804.6 | 635 |
| 219PA | 15Pará | Marapanim | 150440 | 1504406 | 27362.4 | 3345.6 | 3087.3 | 2864.8 | 2669.3 | 586 |
| 220PA | 15Pará | Marituba | 150442 | 1504422 | 120741.1 | 14763 | 13623.2 | 12641.6 | 11778.6 | 9699 |
| 221PA | 15Pará | Medicilândia | 150445 | 1504455 | 29550.9 | 3613.2 | 3334.2 | 3094 | 2882.8 | 1087 |
| 222PA | 15Pará | Melgaço | 150450 | 1504505 | 26228.5 | 3207 | 2959.4 | 2746.1 | 2558.7 | 227 |
| 223PA | 15Pará | Mocajuba | 150460 | 1504604 | 29038.3 | 3550.5 | 3276.4 | 3040.3 | 2832.8 | 223 |
| 224PA | 15Pará | Moju | 150470 | 1504703 | 76372.9 | 9338.1 | 8617.2 | 7996.2 | 7450.4 | 731 |
| 225PA | 15Pará | Monte Alegre | 150480 | 1504802 | 56438 | 6900.7 | 6367.9 | 5909 | 5505.7 | 1597 |
| 226PA | 15Pará | Muaná | 150490 | 1504901 | 37450.5 | 4579.1 | 4225.5 | 3921.1 | 3653.4 | 182 |
| 227PA | 15Pará | Nova Esperança  do Piriá | 150495 | 1504950 | 20672.5 | 2527.6 | 2332.5 | 2164.4 | 2016.7 | 132 |
| 228PA | 15Pará | Nova Ipixuna | 150497 | 1504976 | 15688.8 | 1918.3 | 1770.2 | 1642.6 | 1530.5 | 519 |
| 229PA | 15Pará | Nova Timboteua | 150500 | 1505007 | 14525.2 | 1776 | 1638.9 | 1520.8 | 1417 | 273 |
| 230PA | 15Pará | Novo Progresso | 150503 | 1505031 | 25261.3 | 3088.7 | 2850.2 | 2644.9 | 2464.3 | 162 |
| 231PA | 15Pará | Novo  Repartimento | 150506 | 1505064 | 69518.3 | 8500 | 7843.7 | 7278.6 | 6781.7 | 358 |
| 232PA | 15Pará | Óbidos | 150510 | 1505106 | 50501.8 | 6174.9 | 5698.1 | 5287.5 | 4926.6 | 728 |
| 233PA | 15Pará | Oeiras do Pará | 150520 | 1505205 | 30601.6 | 3741.7 | 3452.8 | 3204 | 2985.3 | 97 |
| 234PA | 15Pará | Oriximiná | 150530 | 1505304 | 68186.5 | 8337.2 | 7693.5 | 7139.1 | 6651.8 | 591 |
| 235PA | 15Pará | Ourém | 150540 | 1505403 | 17048 | 2084.5 | 1923.5 | 1784.9 | 1663.1 | 846 |
| 236PA | 15Pará | Ourilândia do  Norte | 150543 | 1505437 | 30279.9 | 3702.3 | 3416.5 | 3170.3 | 2953.9 | 695 |
| 237PA | 15Pará | Pacajá | 150548 | 1505486 | 44089.9 | 5390.9 | 4974.7 | 4616.2 | 4301.1 | 843 |
| 238PA | 15Pará | Palestina do Pará | 150549 | 1505494 | 7471.8 | 913.6 | 843 | 782.3 | 728.9 | 33 |
| 239PA | 15Pará | Paragominas | 150550 | 1505502 | 105800.8 | 12936.3 | 11937.5 | 11077.3 | 10321.2 | 10592 |
| 240PA | 15Pará | Parauapebas | 150553 | 1505536 | 184010.3 | 22498.9 | 20761.9 | 19265.8 | 17950.7 | 3985 |
| 241PA | 15Pará | Pau D'Arco | 150555 | 1505551 | 5658.3 | 691.8 | 638.4 | 592.4 | 552 | 566 |
| 242PA | 15Pará | Peixe-Boi | 150560 | 1505601 | 7910.5 | 967.2 | 892.5 | 828.2 | 771.7 | 114 |
| 243PA | 15Pará | Piçarra | 150563 | 1505635 | 12743.8 | 1558.2 | 1437.9 | 1334.3 | 1243.2 | 96 |
| 244PA | 15Pará | Placas | 150565 | 1505650 | 27799.8 | 3399.1 | 3136.7 | 2910.6 | 2711.9 | 186 |
| 245PA | 15Pará | Ponta de Pedras | 150570 | 1505700 | 28705.7 | 3509.8 | 3238.9 | 3005.5 | 2800.3 | 334 |
| 246PA | 15Pará | Portel | 150580 | 1505809 | 57412.8 | 7019.9 | 6477.9 | 6011.1 | 5600.8 | 1272 |
| 247PA | 15Pará | Porto de Moz | 150590 | 1505908 | 37805.1 | 4622.4 | 4265.5 | 3958.2 | 3688 | 386 |
| 248PA | 15Pará | Prainha | 150600 | 1506005 | 29377.4 | 3592 | 3314.7 | 3075.8 | 2865.8 | 282 |
| 249PA | 15Pará | Primavera | 150610 | 1506104 | 10496.6 | 1283.4 | 1184.3 | 1099 | 1024 | 425 |
| 250PA | 15Pará | Quatipuru | 150611 | 1506112 | 12990.1 | 1588.3 | 1465.7 | 1360.1 | 1267.2 | 120 |
| 251PA | 15Pará | Redenção | 150613 | 1506138 | 80209 | 9807.2 | 9050 | 8397.9 | 7824.6 | 5235 |
| 252PA | 15Pará | Rio Maria | 150616 | 1506161 | 17820.9 | 2179 | 2010.7 | 1865.8 | 1738.5 | 1298 |
| 253PA | 15Pará | Rondon do Pará | 150618 | 1506187 | 49656.6 | 6071.5 | 5602.8 | 5199 | 4844.1 | 216 |
| 254PA | 15Pará | Rurópolis | 150619 | 1506195 | 45760 | 5595.1 | 5163.1 | 4791.1 | 4464 | 408 |
| 255PA | 15Pará | Salinópolis | 150620 | 1506203 | 38961.3 | 4763.8 | 4396 | 4079.2 | 3800.8 | 776 |
| 256PA | 15Pará | Salvaterra | 150630 | 1506302 | 22066.4 | 2698.1 | 2489.8 | 2310.3 | 2152.6 | 227 |
| 257PA | 15Pará | Santa Bárbara do  Pará | 150635 | 1506351 | 19266.6 | 2355.7 | 2173.9 | 2017.2 | 1879.5 | 1023 |
| 258PA | 15Pará | Santa Cruz do  Arari | 150640 | 1506401 | 9223.8 | 1127.8 | 1040.7 | 965.7 | 899.8 | 26 |
| 259PA | 15Pará | Santa Isabel do  Pará | 150650 | 1506500 | 65488 | 8007.2 | 7389 | 6856.6 | 6388.5 | 4330 |
| 260PA | 15Pará | Santa Luzia do  Pará | 150655 | 1506559 | 19489.8 | 2383 | 2199 | 2040.6 | 1901.3 | 211 |
| 261PA | 15Pará | Santa Maria das  Barreiras | 150658 | 1506583 | 19507.4 | 2385.2 | 2201 | 2042.4 | 1903 | 582 |
| 262PA | 15Pará | Santa Maria do  Pará | 150660 | 1506609 | 23877.1 | 2919.5 | 2694.1 | 2499.9 | 2329.3 | 708 |
| 263PA | 15Pará | Santana do  Araguaia | 150670 | 1506708 | 65296.7 | 7983.8 | 7367.4 | 6836.6 | 6369.9 | 694 |
| 264PA | 15Pará | Santarém | 150680 | 1506807 | 296054.6 | 36198.6 | 33403.8 | 30996.9 | 28881 | 14194 |
| 265PA | 15Pará | Santarém Novo | 150690 | 1506906 | 6413.3 | 784.2 | 723.6 | 671.5 | 625.6 | 42 |
| 266PA | 15Pará | Santo Antônio do  Tauá | 150700 | 1507003 | 29215.4 | 3572.2 | 3296.4 | 3058.8 | 2850 | 1199 |
| 267PA | 15Pará | São Caetano de  Odivelas | 150710 | 1507102 | 17407.3 | 2128.4 | 1964.1 | 1822.5 | 1698.1 | 703 |
| 268PA | 15Pará | São Domingos do  Araguaia | 150715 | 1507151 | 24323.3 | 2974 | 2744.4 | 2546.6 | 2372.8 | 171 |
| 269PA | 15Pará | São Domingos do  Capim | 150720 | 1507201 | 30812.8 | 3767.5 | 3476.6 | 3226.1 | 3005.9 | 361 |
| 270PA | 15Pará | São Félix do Xingu | 150730 | 1507300 | 112032.8 | 13698.3 | 12640.7 | 11729.8 | 10929.1 | 432 |
| 271PA | 15Pará | São Francisco do  Pará | 150740 | 1507409 | 15397.6 | 1882.7 | 1737.3 | 1612.1 | 1502.1 | 700 |
| 272PA | 15Pará | São Geraldo do  Araguaia | 150745 | 1507458 | 24920.4 | 3047 | 2811.8 | 2609.2 | 2431.1 | 126 |
| 273PA | 15Pará | São João da  Ponta | 150746 | 1507466 | 5723.3 | 699.8 | 645.8 | 599.2 | 558.3 | 183 |
| 274PA | 15Pará | São João de  Pirabas | 150747 | 1507474 | 21846.5 | 2671.2 | 2464.9 | 2287.3 | 2131.2 | 241 |
| 275PA | 15Pará | São João do  Araguaia | 150750 | 1507508 | 13520.4 | 1653.1 | 1525.5 | 1415.6 | 1319 | 170 |
| 276PA | 15Pará | São Miguel do  Guamá | 150760 | 1507607 | 55392 | 6772.8 | 6249.9 | 5799.5 | 5403.6 | 1136 |
| 277PA | 15Pará | São Sebastião da  Boa Vista | 150770 | 1507706 | 24858.3 | 3039.4 | 2804.8 | 2602.7 | 2425 | 116 |
| 278PA | 15Pará | Sapucaia | 150775 | 1507755 | 5512.3 | 674 | 622 | 577.1 | 537.7 | 429 |
| 279PA | 15Pará | Senador José  Porfírio | 150780 | 1507805 | 12120 | 1481.9 | 1367.5 | 1269 | 1182.3 | 297 |
| 280PA | 15Pará | Soure | 150790 | 1507904 | 24164.5 | 2954.6 | 2726.5 | 2530 | 2357.3 | 210 |
| 281PA | 15Pará | Tailândia | 150795 | 1507953 | 94244.1 | 11523.2 | 10633.6 | 9867.3 | 9193.8 | 400 |
| 282PA | 15Pará | Terra Alta | 150796 | 1507961 | 11013 | 1346.6 | 1242.6 | 1153.1 | 1074.3 | 343 |
| 283PA | 15Pará | Terra Santa | 150797 | 1507979 | 17847.7 | 2182.2 | 2013.8 | 1868.7 | 1741.1 | 51 |
| 284PA | 15Pará | Tomé-Açu | 150800 | 1508001 | 60012.8 | 7337.8 | 6771.2 | 6283.3 | 5854.4 | 725 |
| 285PA | 15Pará | Tracuateua | 150803 | 1508035 | 29232 | 3574.2 | 3298.2 | 3060.6 | 2851.7 | 698 |
| 286PA | 15Pará | Trairão | 150805 | 1508050 | 17944.9 | 2194.1 | 2024.7 | 1878.8 | 1750.6 | 47 |
| 287PA | 15Pará | Tucumã | 150808 | 1508084 | 36807.1 | 4500.4 | 4152.9 | 3853.7 | 3590.6 | 1080 |
| 288PA | 15Pará | Tucuruí | 150810 | 1508100 | 105814.5 | 12937.9 | 11939.1 | 11078.8 | 10322.5 | 3733 |

4

3

3

1

2

1

5

1

| 289PA | 15Pará | Ulianópolis | 150812 | 1508126 | 52142.5 | 6375.5 | 5883.2 | 5459.3 | 5086.6 | 724 |
| --- | --- | --- | --- | --- | --- | --- | --- | --- | --- | --- |
| 290PA | 15Pará | Uruará | 150815 | 1508159 | 44771.8 | 5474.2 | 5051.6 | 4687.6 | 4367.6 | 493 |
| 291PA | 15Pará | Vigia | 150820 | 1508209 | 50807.2 | 6212.2 | 5732.6 | 5319.5 | 4956.4 | 1157 |
| 292PA | 15Pará | Viseu | 150830 | 1508308 | 58908.9 | 7202.8 | 6646.7 | 6167.8 | 5746.7 | 395 |
| 293PA | 15Pará | Vitória do Xingu | 150835 | 1508357 | 14294.2 | 1747.8 | 1612.8 | 1496.6 | 1394.4 | 364 |
| 294PA | 15Pará | Xinguara | 150840 | 1508407 | 42620.1 | 5211.2 | 4808.8 | 4462.3 | 4157.7 | 1347 |
| 295AP | 16Amapá | Serra do Navio | 160005 | 1600055 | 4877.6 | 479.6 | 527 | 378.5 | 442.5 | 14 |
| 296AP | 16Amapá | Amapá | 160010 | 1600105 | 8566.7 | 842.4 | 925.6 | 664.7 | 777.3 | 155 |
| 297AP | 16Amapá | Pedra Branca do  Amapari | 160015 | 1600154 | 13620.2 | 1339.3 | 1471.7 | 1056.8 | 1235.8 | 29 |
| 298AP | 16Amapá | Calçoene | 160020 | 1600204 | 10036.5 | 986.9 | 1084.4 | 778.8 | 910.6 | 48 |
| 299AP | 16Amapá | Cutias | 160021 | 1600212 | 5328.9 | 524 | 575.8 | 413.5 | 483.5 | 74 |
| 300AP | 16Amapá | Ferreira Gomes | 160023 | 1600238 | 6778.5 | 666.5 | 732.4 | 526 | 615 | 529 |
| 301AP | 16Amapá | Itaubal | 160025 | 1600253 | 4873.8 | 479.2 | 526.6 | 378.2 | 442.2 | 286 |
| 302AP | 16Amapá | Laranjal do Jari | 160027 | 1600279 | 45079.9 | 4432.7 | 4870.9 | 3497.8 | 4090.1 | 143 |
| 303AP | 16Amapá | Macapá | 160030 | 1600303 | 449812.7 | 44230.1 | 48602.3 | 34902 | 40811.3 | 6342 |
| 304AP | 16Amapá | Mazagão | 160040 | 1600402 | 19291.8 | 1897 | 2084.5 | 1496.9 | 1750.3 | 53 |
| 305AP | 16Amapá | Oiapoque | 160050 | 1600501 | 23844.4 | 2344.6 | 2576.4 | 1850.1 | 2163.4 | 24 |
| 306AP | 16Amapá | Porto Grande | 160053 | 1600535 | 19351.8 | 1902.9 | 2091 | 1501.5 | 1755.8 | 77 |
| 307AP | 16Amapá | Pracuúba | 160055 | 1600550 | 4448.2 | 437.4 | 480.6 | 345.1 | 403.6 | 11 |
| 308AP | 16Amapá | Santana | 160060 | 1600600 | 111050.8 | 10919.6 | 11999 | 8616.7 | 10075.6 | 872 |
| 309AP | 16Amapá | Tartarugalzinho | 160070 | 1600709 | 14913.4 | 1466.4 | 1611.4 | 1157.2 | 1353.1 | 23 |
| 310AP | 16Amapá | Vitória do Jari | 160080 | 1600808 | 14150.6 | 1391.4 | 1529 | 1098 | 1283.9 | 16 |
| 311TO | 17Tocantins | Abreulândia | 170025 | 1700251 | 2505.7 | 339.5 | 301.2 | 312.5 | 274.3 | 161 |
| 312TO | 17Tocantins | Aguiarnópolis | 170030 | 1700301 | 5997.4 | 812.7 | 720.9 | 747.9 | 656.4 | 436 |
| 313TO | 17Tocantins | Aliança do  Tocantins | 170035 | 1700350 | 5575 | 755.5 | 670.1 | 695.2 | 610.2 | 638 |
| 314TO | 17Tocantins | Almas | 170040 | 1700400 | 7379.7 | 1000 | 887 | 920.2 | 807.7 | 465 |
| 315TO | 17Tocantins | Alvorada | 170070 | 1700707 | 8461.2 | 1146.6 | 1017 | 1055.1 | 926.1 | 873 |
| 316TO | 17Tocantins | Ananás | 170100 | 1701002 | 9785.1 | 1326 | 1176.2 | 1220.2 | 1071 | 691 |
| 317TO | 17Tocantins | Angico | 170105 | 1701051 | 3333.1 | 451.7 | 400.6 | 415.6 | 364.8 | 325 |
| 318TO | 17Tocantins | Aparecida do Rio  Negro | 170110 | 1701101 | 4541.5 | 615.4 | 545.9 | 566.3 | 497.1 | 300 |
| 319TO | 17Tocantins | Aragominas | 170130 | 1701309 | 5867.6 | 795.1 | 705.3 | 731.7 | 642.2 | 946 |
| 320TO | 17Tocantins | Araguacema | 170190 | 1701903 | 6756.6 | 915.6 | 812.1 | 842.5 | 739.5 | 367 |
| 321TO | 17Tocantins | Araguaçu | 170200 | 1702000 | 8720.8 | 1181.8 | 1048.2 | 1087.5 | 954.5 | 807 |
| 322TO | 17Tocantins | Araguaína | 170210 | 1702109 | 166846.8 | 22609.4 | 20055 | 20805.2 | 18262.1 | 12507 |
| 323TO | 17Tocantins | Araguanã | 170215 | 1702158 | 5424.1 | 735 | 652 | 676.4 | 593.7 | 362 |
| 324TO | 17Tocantins | Araguatins | 170220 | 1702208 | 33821.1 | 4583.1 | 4065.3 | 4217.4 | 3701.9 | 506 |
| 325TO | 17Tocantins | Arapoema | 170230 | 1702307 | 6747.2 | 914.3 | 811 | 841.4 | 738.5 | 627 |
| 326TO | 17Tocantins | Arraias | 170240 | 1702406 | 10692.1 | 1448.9 | 1285.2 | 1333.3 | 1170.3 | 225 |
| 327TO | 17Tocantins | Augustinópolis | 170255 | 1702554 | 17322.3 | 2347.3 | 2082.1 | 2160 | 1896 | 897 |
| 328TO | 17Tocantins | Aurora do  Tocantins | 170270 | 1702703 | 3631 | 492 | 436.4 | 452.8 | 397.4 | 247 |
| 329TO | 17Tocantins | Axixá do  Tocantins | 170290 | 1702901 | 9593.2 | 1300 | 1153.1 | 1196.2 | 1050 | 377 |
| 330TO | 17Tocantins | Babaçulândia | 170300 | 1703008 | 10630 | 1440.5 | 1277.7 | 1325.5 | 1163.5 | 793 |
| 331TO | 17Tocantins | Bandeirantes do  Tocantins | 170305 | 1703057 | 3364.3 | 455.9 | 404.4 | 419.5 | 368.2 | 123 |
| 332TO | 17Tocantins | Barra do Ouro | 170307 | 1703073 | 4392.8 | 595.3 | 528 | 547.8 | 480.8 | 313 |
| 333TO | 17Tocantins | Barrolândia | 170310 | 1703107 | 5535.6 | 750.1 | 665.4 | 690.3 | 605.9 | 477 |
| 334TO | 17Tocantins | Bernardo Sayão | 170320 | 1703206 | 4493.7 | 608.9 | 540.1 | 560.3 | 491.9 | 387 |
| 335TO | 17Tocantins | Bom Jesus do  Tocantins | 170330 | 1703305 | 4367.6 | 591.9 | 525 | 544.6 | 478.1 | 143 |
| 336TO | 17Tocantins | Brasilândia do  Tocantins | 170360 | 1703602 | 2150.1 | 291.4 | 258.4 | 268.1 | 235.3 | 153 |
| 337TO | 17Tocantins | Brejinho de  Nazaré | 170370 | 1703701 | 5384.9 | 729.7 | 647.3 | 671.5 | 589.4 | 213 |
| 338TO | 17Tocantins | Buriti do Tocantins | 170380 | 1703800 | 10645.1 | 1442.5 | 1279.5 | 1327.4 | 1165.2 | 414 |
| 339TO | 17Tocantins | Cachoeirinha | 170382 | 1703826 | 2229.4 | 302.1 | 268 | 278 | 244 | 195 |
| 340TO | 17Tocantins | Campos Lindos | 170384 | 1703842 | 9203.4 | 1247.2 | 1106.2 | 1147.6 | 1007.4 | 154 |
| 341TO | 17Tocantins | Cariri do Tocantins | 170386 | 1703867 | 4102.7 | 556 | 493.1 | 511.6 | 449.1 | 433 |
| 342TO | 17Tocantins | Carmolândia | 170388 | 1703883 | 2468.7 | 334.5 | 296.7 | 307.8 | 270.2 | 256 |
| 343TO | 17Tocantins | Carrasco Bonito | 170389 | 1703891 | 3923.3 | 531.6 | 471.6 | 489.2 | 429.4 | 199 |
| 344TO | 17Tocantins | Caseara | 170390 | 1703909 | 5026.7 | 681.2 | 604.2 | 626.8 | 550.2 | 154 |
| 345TO | 17Tocantins | Centenário | 170410 | 1704105 | 2758.1 | 373.8 | 331.5 | 343.9 | 301.9 | 25 |
| 346TO | 17Tocantins | Chapada de Areia | 170460 | 1704600 | 1381 | 187.1 | 166 | 172.2 | 151.2 | 148 |
| 347TO | 17Tocantins | Chapada da  Natividade | 170510 | 1705102 | 3331.4 | 451.4 | 400.4 | 415.4 | 364.6 | 253 |
| 348TO | 17Tocantins | Colinas do  Tocantins | 170550 | 1705508 | 33403.9 | 4526.6 | 4015.1 | 4165.3 | 3656.2 | 3347 |
| 349TO | 17Tocantins | Combinado | 170555 | 1705557 | 4798.9 | 650.3 | 576.8 | 598.4 | 525.3 | 333 |
| 350TO | 17Tocantins | Conceição do  Tocantins | 170560 | 1705607 | 4177.7 | 566.1 | 502.2 | 520.9 | 457.3 | 367 |
| 351TO | 17Tocantins | Cristalândia | 170610 | 1706100 | 7320.5 | 992 | 879.9 | 912.8 | 801.3 | 396 |
| 352TO | 17Tocantins | Crixás do  Tocantins | 170625 | 1706258 | 1656.3 | 224.4 | 199.1 | 206.5 | 181.3 | 185 |
| 353TO | 17Tocantins | Darcinópolis | 170650 | 1706506 | 5731.9 | 776.7 | 689 | 714.7 | 627.4 | 583 |
| 354TO | 17Tocantins | Dianópolis | 170700 | 1707009 | 20796.3 | 2818.1 | 2499.7 | 2593.2 | 2276.2 | 1283 |
| 355TO | 17Tocantins | Divinópolis do  Tocantins | 170710 | 1707108 | 6685.7 | 906 | 803.6 | 833.7 | 731.8 | 908 |
| 356TO | 17Tocantins | Dois Irmãos do  Tocantins | 170720 | 1707207 | 7238 | 980.8 | 870 | 902.6 | 792.2 | 532 |
| 357TO | 17Tocantins | Dueré | 170730 | 1707306 | 4676.8 | 633.8 | 562.2 | 583.2 | 511.9 | 399 |
| 358TO | 17Tocantins | Esperantina | 170740 | 1707405 | 10320.6 | 1398.5 | 1240.5 | 1286.9 | 1129.6 | 436 |
| 359TO | 17Tocantins | Fátima | 170755 | 1707553 | 3851 | 521.8 | 462.9 | 480.2 | 421.5 | 381 |
| 360TO | 17Tocantins | Figueirópolis | 170765 | 1707652 | 5344.8 | 724.3 | 642.4 | 666.5 | 585 | 692 |
| 361TO | 17Tocantins | Filadélfia | 170770 | 1707702 | 8751 | 1185.8 | 1051.9 | 1091.2 | 957.8 | 366 |
| 362TO | 17Tocantins | Formoso do  Araguaia | 170820 | 1708205 | 18583.1 | 2518.2 | 2233.7 | 2317.2 | 2034 | 1266 |
| 363TO | 17Tocantins | Fortaleza do  Tabocão | 170825 | 1708254 | 2524.2 | 342.1 | 303.4 | 314.8 | 276.3 | 175 |
| 364TO | 17Tocantins | Goianorte | 170830 | 1708304 | 5080.6 | 688.5 | 610.7 | 633.5 | 556.1 | 387 |
| 365TO | 17Tocantins | Goiatins | 170900 | 1709005 | 12644.2 | 1713.4 | 1519.8 | 1576.7 | 1384 | 493 |
| 366TO | 17Tocantins | Guaraí | 170930 | 1709302 | 24763.6 | 3355.7 | 2976.6 | 3087.9 | 2710.5 | 1679 |
| 367TO | 17Tocantins | Gurupi | 170950 | 1709500 | 82373.1 | 11162.4 | 9901.2 | 10271.6 | 9016.1 | 8659 |
| 368TO | 17Tocantins | Ipueiras | 170980 | 1709807 | 1841.8 | 249.6 | 221.4 | 229.7 | 201.6 | 50 |
| 369TO | 17Tocantins | Itacajá | 171050 | 1710508 | 7327.7 | 993 | 880.8 | 913.7 | 802 | 176 |
| 370TO | 17Tocantins | Itaguatins | 171070 | 1710706 | 5994.4 | 812.3 | 720.5 | 747.5 | 656.1 | 333 |
| 371TO | 17Tocantins | Itapiratins | 171090 | 1710904 | 3684.7 | 499.3 | 442.9 | 459.5 | 403.3 | 118 |
| 372TO | 17Tocantins | Itaporã do  Tocantins | 171110 | 1711100 | 2456.1 | 332.8 | 295.2 | 306.3 | 268.8 | 153 |
| 373TO | 17Tocantins | Jaú do Tocantins | 171150 | 1711506 | 3707.9 | 502.5 | 445.7 | 462.4 | 405.8 | 300 |
| 374TO | 17Tocantins | Juarina | 171180 | 1711803 | 2229.1 | 302.1 | 267.9 | 278 | 244 | 243 |
| 375TO | 17Tocantins | Lagoa da  Confusão | 171190 | 1711902 | 11881.9 | 1610.1 | 1428.2 | 1481.6 | 1300.5 | 346 |
| 376TO | 17Tocantins | Lagoa do  Tocantins | 171195 | 1711951 | 3953 | 535.7 | 475.2 | 492.9 | 432.7 | 189 |
| 377TO | 17Tocantins | Lajeado | 171200 | 1712009 | 2977.6 | 403.5 | 357.9 | 371.3 | 325.9 | 174 |
| 378TO | 17Tocantins | Lavandeira | 171215 | 1712157 | 1778.5 | 241 | 213.8 | 221.8 | 194.7 | 168 |
| 379TO | 17Tocantins | Lizarda | 171240 | 1712405 | 3763 | 509.9 | 452.3 | 469.2 | 411.9 | 53 |
| 380TO | 17Tocantins | Luzinópolis | 171245 | 1712454 | 2888.9 | 391.5 | 347.2 | 360.2 | 316.2 | 97 |
| 381TO | 17Tocantins | Marianópolis do  Tocantins | 171250 | 1712504 | 4802.8 | 650.8 | 577.3 | 598.9 | 525.7 | 335 |
| 382TO | 17Tocantins | Mateiros | 171270 | 1712702 | 2473.9 | 335.2 | 297.4 | 308.5 | 270.8 | 14 |

2

4

1

2

| 383TO | 17Tocantins | Maurilândia do  Tocantins | 171280 | 1712801 | 3316.8 | 449.5 | 398.7 | 413.6 | 363 | 147 |  |
| --- | --- | --- | --- | --- | --- | --- | --- | --- | --- | --- | --- |
| 384TO | 17Tocantins | Miracema do  Tocantins | 171320 | 1713205 | 19621.6 | 2658.9 | 2358.5 | 2446.7 | 2147.7 | 1699 |  |
| 385TO | 17Tocantins | Miranorte | 171330 | 1713304 | 13134.8 | 1779.9 | 1578.8 | 1637.9 | 1437.7 | 718 |  |
| 386TO | 17Tocantins | Monte do Carmo | 171360 | 1713601 | 7392.6 | 1001.8 | 888.6 | 921.8 | 809.2 | 245 |  |
| 387TO | 17Tocantins | Monte Santo do  Tocantins | 171370 | 1713700 | 2199.9 | 298.1 | 264.4 | 274.3 | 240.8 | 297 |  |
| 388TO | 17Tocantins | Palmeiras do  Tocantins | 171380 | 1713809 | 6250.2 | 847 | 751.3 | 779.4 | 684.1 | 467 |  |
| 389TO | 17Tocantins | Muricilândia | 171395 | 1713957 | 3379 | 457.9 | 406.2 | 421.3 | 369.8 | 254 |  |
| 390TO | 17Tocantins | Natividade | 171420 | 1714203 | 9196.5 | 1246.2 | 1105.4 | 1146.8 | 1006.6 | 161 |  |
| 391TO | 17Tocantins | Nazaré | 171430 | 1714302 | 4173.6 | 565.6 | 501.7 | 520.4 | 456.8 | 605 |  |
| 392TO | 17Tocantins | Nova Olinda | 171488 | 1714880 | 11344.6 | 1537.3 | 1363.6 | 1414.6 | 1241.7 | 684 |  |
| 393TO | 17Tocantins | Nova Rosalândia | 171500 | 1715002 | 4047.7 | 548.5 | 486.5 | 504.7 | 443 | 515 |  |
| 394TO | 17Tocantins | Novo Acordo | 171510 | 1715101 | 4085.8 | 553.7 | 491.1 | 509.5 | 447.2 | 240 |  |
| 395TO | 17Tocantins | Novo Alegre | 171515 | 1715150 | 2328 | 315.5 | 279.8 | 290.3 | 254.8 | 274 |  |
| 396TO | 17Tocantins | Novo Jardim | 171525 | 1715259 | 2610.7 | 353.8 | 313.8 | 325.5 | 285.8 | 200 |  |
| 397TO | 17Tocantins | Oliveira de Fátima | 171550 | 1715507 | 1083.5 | 146.8 | 130.2 | 135.1 | 118.6 | 264 |  |
| 398TO | 17Tocantins | Palmeirante | 171570 | 1715705 | 5535.5 | 750.1 | 665.4 | 690.3 | 605.9 | 213 |  |
| 399TO | 17Tocantins | Palmeirópolis | 171575 | 1715754 | 7560 | 1024.5 | 908.7 | 942.7 | 827.5 | 360 |  |
| 400TO | 17Tocantins | Paraíso do  Tocantins | 171610 | 1716109 | 48228.9 | 6535.5 | 5797.1 | 6014 | 5278.9 | 4513 |  |
| 401TO | 17Tocantins | Paranã | 171620 | 1716208 | 10477.4 | 1419.8 | 1259.4 | 1306.5 | 1146.8 | 403 |  |
| 402TO | 17Tocantins | Pau D'Arco | 171630 | 1716307 | 4756.8 | 644.6 | 571.8 | 593.2 | 520.7 | 396 |  |
| 403TO | 17Tocantins | Pedro Afonso | 171650 | 1716505 | 12662.1 | 1715.8 | 1522 | 1578.9 | 1385.9 | 461 |  |
| 404TO | 17Tocantins | Peixe | 171660 | 1716604 | 11157.5 | 1512 | 1341.1 | 1391.3 | 1221.2 | 790 |  |
| 405TO | 17Tocantins | Pequizeiro | 171665 | 1716653 | 5308.9 | 719.4 | 638.1 | 662 | 581.1 | 484 |  |
| 406TO | 17Tocantins | Colméia | 171670 | 1716703 | 8475.9 | 1148.6 | 1018.8 | 1056.9 | 927.7 | 649 |  |
| 407TO | 17Tocantins | Pindorama do  Tocantins | 171700 | 1717008 | 4512.7 | 611.5 | 542.4 | 562.7 | 493.9 | 55 |  |
| 408TO | 17Tocantins | Piraquê | 171720 | 1717206 | 2998.4 | 406.3 | 360.4 | 373.9 | 328.2 | 460 |  |
| 409TO | 17Tocantins | Pium | 171750 | 1717503 | 7233.6 | 980.2 | 869.5 | 902 | 791.7 | 446 |  |
| 410TO | 17Tocantins | Ponte Alta do Bom  Jesus | 171780 | 1717800 | 4607 | 624.3 | 553.8 | 574.5 | 504.3 | 404 |  |
| 411TO | 17Tocantins | Ponte Alta do  Tocantins | 171790 | 1717909 | 7672.4 | 1039.7 | 922.2 | 956.7 | 839.8 | 142 |  |
| 412TO | 17Tocantins | Porto Alegre do  Tocantins | 171800 | 1718006 | 2991.9 | 405.4 | 359.6 | 373.1 | 327.5 | 304 |  |
| 413TO | 17Tocantins | Porto Nacional | 171820 | 1718204 | 51496.2 | 6978.3 | 6189.8 | 6421.4 | 5636.5 | 3829 |  |
| 414TO | 17Tocantins | Praia Norte | 171830 | 1718303 | 8110.9 | 1099.1 | 974.9 | 1011.4 | 887.8 | 724 |  |
| 415TO | 17Tocantins | Presidente  Kennedy | 171840 | 1718402 | 3712.3 | 503.1 | 446.2 | 462.9 | 406.3 | 384 |  |
| 416TO | 17Tocantins | Pugmil | 171845 | 1718451 | 2549.3 | 345.5 | 306.4 | 317.9 | 279 | 284 |  |
| 417TO | 17Tocantins | Recursolândia | 171850 | 1718501 | 4063.9 | 550.7 | 488.5 | 506.8 | 444.8 | 28 |  |
| 418TO | 17Tocantins | Riachinho | 171855 | 1718550 | 4453.6 | 603.5 | 535.3 | 555.3 | 487.5 | 385 |  |
| 419TO | 17Tocantins | Rio da Conceição | 171865 | 1718659 | 1937.5 | 262.6 | 232.9 | 241.6 | 212.1 | 178 |  |
| 420TO | 17Tocantins | Rio dos Bois | 171870 | 1718709 | 2724 | 369.1 | 327.4 | 339.7 | 298.2 | 142 |  |
| 421TO | 17Tocantins | Rio Sono | 171875 | 1718758 | 6417.6 | 869.6 | 771.4 | 800.3 | 702.4 | 110 |  |
| 422TO | 17Tocantins | Sampaio | 171880 | 1718808 | 4323.5 | 585.9 | 519.7 | 539.1 | 473.2 | 262 |  |
| 423TO | 17Tocantins | Sandolândia | 171884 | 1718840 | 3378.3 | 457.8 | 406.1 | 421.3 | 369.8 | 246 |  |
| 424TO | 17Tocantins | Santa Fé do  Araguaia | 171886 | 1718865 | 7113.4 | 963.9 | 855 | 887 | 778.6 | 779 |  |
| 425TO | 17Tocantins | Santa Maria do  Tocantins | 171888 | 1718881 | 3190.3 | 432.3 | 383.5 | 397.8 | 349.2 | 97 |  |
| 426TO | 17Tocantins | Santa Rita do  Tocantins | 171889 | 1718899 | 2265.4 | 307 | 272.3 | 282.5 | 248 | 211 |  |
| 427TO | 17Tocantins | Santa Rosa do  Tocantins | 171890 | 1718907 | 4736.8 | 641.9 | 569.4 | 590.7 | 518.5 | 461 |  |
| 428TO | 17Tocantins | Santa Tereza do  Tocantins | 171900 | 1719004 | 2716.7 | 368.1 | 326.5 | 338.8 | 297.4 | 293 |  |
| 429TO | 17Tocantins | Santa Terezinha do Tocantins | 172000 | 1720002 | 2521.6 | 341.7 | 303.1 | 314.4 | 276 | 379 |  |
| 430TO | 17Tocantins | São Bento do  Tocantins | 172010 | 1720101 | 5007.4 | 678.6 | 601.9 | 624.4 | 548.1 | 331 |  |
| 431TO | 17Tocantins | São Félix do  Tocantins | 172015 | 1720150 | 1523.2 | 206.4 | 183.1 | 189.9 | 166.7 | 78 |  |
| 432TO | 17Tocantins | São Miguel do  Tocantins | 172020 | 1720200 | 11396.8 | 1544.4 | 1369.9 | 1421.1 | 1247.4 | 512 |  |
| 433TO | 17Tocantins | São Salvador do  Tocantins | 172025 | 1720259 | 3020.8 | 409.3 | 363.1 | 376.7 | 330.6 | 239 |  |
| 434TO | 17Tocantins | São Sebastião do  Tocantins | 172030 | 1720309 | 4581.3 | 620.8 | 550.7 | 571.3 | 501.4 | 342 |  |
| 435TO | 17Tocantins | Silvanópolis | 172065 | 1720655 | 5278.6 | 715.3 | 634.5 | 658.2 | 577.8 | 553 |  |
| 436TO | 17Tocantins | Sítio Novo do  Tocantins | 172080 | 1720804 | 9169.3 | 1242.5 | 1102.1 | 1143.4 | 1003.6 | 755 |  |
| 437TO | 17Tocantins | Sucupira | 172085 | 1720853 | 1869.5 | 253.3 | 224.7 | 233.1 | 204.6 | 226 |  |
| 438TO | 17Tocantins | Taguatinga | 172090 | 1720903 | 15997.4 | 2167.8 | 1922.9 | 1994.8 | 1751 | 810 |  |
| 439TO | 17Tocantins | Taipas do  Tocantins | 172093 | 1720937 | 2063.5 | 279.6 | 248 | 257.3 | 225.9 | 71 |  |
| 440TO | 17Tocantins | Talismã | 172097 | 1720978 | 2699.3 | 365.8 | 324.5 | 336.6 | 295.4 | 350 |  |
| 441TO | 17Tocantins | Palmas | 172100 | 1721000 | 265938.1 | 36037.3 | 31965.8 | 33161.5 | 29108 | 33051 | 12 |
| 442TO | 17Tocantins | Tocantínia | 172110 | 1721109 | 7199.5 | 975.6 | 865.4 | 897.8 | 788 | 246 |  |
| 443TO | 17Tocantins | Tocantinópolis | 172120 | 1721208 | 22929.6 | 3107.2 | 2756.1 | 2859.2 | 2509.7 | 1858 |  |
| 444TO | 17Tocantins | Tupirama | 172125 | 1721257 | 1747 | 236.7 | 210 | 217.8 | 191.2 | 99 |  |
| 445TO | 17Tocantins | Tupiratins | 172130 | 1721307 | 2403.9 | 325.8 | 288.9 | 299.8 | 263.1 | 89 |  |
| 446TO | 17Tocantins | Wanderlândia | 172208 | 1722081 | 11424.2 | 1548.1 | 1373.2 | 1424.6 | 1250.4 | 1024 |  |
| 447TO | 17Tocantins | Xambioá | 172210 | 1722107 | 11605.8 | 1572.7 | 1395 | 1447.2 | 1270.3 | 1117 |  |
| 448MA | 21Maranhão | Açailândia | 210005 | 2100055 | 108804.7 | 14435.1 | 11869.5 | 13260.1 | 10845.2 | 910 |  |
| 449MA | 21Maranhão | Afonso Cunha | 210010 | 2100105 | 6271.9 | 832.1 | 684.2 | 764.4 | 625.2 | 220 |  |
| 450MA | 21Maranhão | Água Doce do  Maranhão | 210015 | 2100154 | 12147.8 | 1611.6 | 1325.2 | 1480.5 | 1210.8 | 59 |  |
| 451MA | 21Maranhão | Alcântara | 210020 | 2100204 | 21782.5 | 2889.9 | 2376.3 | 2654.6 | 2171.2 | 369 |  |
| 452MA | 21Maranhão | Aldeias Altas | 210030 | 2100303 | 25486.8 | 3381.3 | 2780.4 | 3106.1 | 2540.4 | 656 |  |
| 453MA | 21Maranhão | Altamira do  Maranhão | 210040 | 2100402 | 10302.1 | 1366.8 | 1123.9 | 1255.5 | 1026.9 | 30 |  |
| 454MA | 21Maranhão | Alto Alegre do  Maranhão | 210043 | 2100436 | 26042.8 | 3455.1 | 2841 | 3173.9 | 2595.8 | 441 |  |
| 455MA | 21Maranhão | Alto Alegre do  Pindaré | 210047 | 2100477 | 31361.1 | 4160.7 | 3421.2 | 3822 | 3125.9 | 191 |  |
| 456MA | 21Maranhão | Alto Parnaíba | 210050 | 2100501 | 10956.2 | 1453.6 | 1195.2 | 1335.2 | 1092.1 | 210 |  |
| 457MA | 21Maranhão | Amapá do  Maranhão | 210055 | 2100550 | 6733 | 893.3 | 734.5 | 820.6 | 671.1 | 78 |  |
| 458MA | 21Maranhão | Amarante do  Maranhão | 210060 | 2100600 | 39966.5 | 5302.4 | 4359.9 | 4870.7 | 3983.7 | 291 |  |
| 459MA | 21Maranhão | Anajatuba | 210070 | 2100709 | 26460.5 | 3510.5 | 2886.6 | 3224.8 | 2637.5 | 314 |  |
| 460MA | 21Maranhão | Anapurus | 210080 | 2100808 | 15030.2 | 1994.1 | 1639.6 | 1831.7 | 1498.1 | 272 |  |
| 461MA | 21Maranhão | Apicum-Açu | 210083 | 2100832 | 16973.5 | 2251.9 | 1851.6 | 2068.6 | 1691.8 | 276 |  |
| 462MA | 21Maranhão | Araguanã | 210087 | 2100873 | 14565.8 | 1932.4 | 1589 | 1775.1 | 1451.9 | 108 |  |
| 463MA | 21Maranhão | Araioses | 210090 | 2100907 | 44791.5 | 5942.5 | 4886.3 | 5458.8 | 4464.6 | 192 |  |
| 464MA | 21Maranhão | Arame | 210095 | 2100956 | 32065.7 | 4254.2 | 3498 | 3907.9 | 3196.2 | 270 |  |
| 465MA | 21Maranhão | Arari | 210100 | 2101004 | 29152.7 | 3867.7 | 3180.3 | 3552.9 | 2905.8 | 577 |  |
| 466MA | 21Maranhão | Axixá | 210110 | 2101103 | 11794.2 | 1564.7 | 1286.6 | 1437.4 | 1175.6 | 127 |  |
| 467MA | 21Maranhão | Bacabal | 210120 | 2101202 | 102455.6 | 13592.8 | 11176.9 | 12486.3 | 10212.3 | 3045 |  |
| 468MA |  | Bacabeira | 210125 | 2101251 | 16236.5 | 2154.1 | 1771.2 | 1978.8 | 1618.4 | 448 |  |
| 469MA |  | Bacuri | 210130 | 2101301 | 17441.9 | 2314 | 1902.7 | 2125.7 | 1738.5 | 283 |  |
| 470MA |  | Bacurituba | 210135 | 2101350 | 5482.8 | 727.4 | 598.1 | 668.2 | 546.5 | 79 |  |

1

1

1

| 471MA | 21Maranhão | Balsas | 210140 | 2101400 | 90485.8 | 12004.8 | 9871.1 | 11027.6 | 9019.2 | 11432 |  |
| --- | --- | --- | --- | --- | --- | --- | --- | --- | --- | --- | --- |
| 472MA | 21Maranhão | Barão de Grajaú | 210150 | 2101509 | 18304.6 | 2428.5 | 1996.8 | 2230.8 | 1824.5 | 236 |  |
| 473MA | 21Maranhão | Barra do Corda | 210160 | 2101608 | 85717.1 | 11372.1 | 9350.9 | 10446.4 | 8543.9 | 396 |  |
| 474MA | 21Maranhão | Barreirinhas | 210170 | 2101707 | 59638.1 | 7912.2 | 6505.9 | 7268.1 | 5944.5 | 202 |  |
| 475MA | 21Maranhão | Belágua | 210173 | 2101731 | 7135.1 | 946.6 | 778.4 | 869.6 | 711.2 | 104 |  |
| 476MA | 21Maranhão | Bela Vista do  Maranhão | 210177 | 2101772 | 11353.2 | 1506.2 | 1238.5 | 1383.6 | 1131.6 | 184 |  |
| 477MA | 21Maranhão | Benedito Leite | 210180 | 2101806 | 5530.4 | 733.7 | 603.3 | 674 | 551.2 | 149 |  |
| 478MA | 21Maranhão | Bequimão | 210190 | 2101905 | 20830.9 | 2763.6 | 2272.4 | 2538.7 | 2076.3 | 285 |  |
| 479MA | 21Maranhão | Bernardo do  Mearim | 210193 | 2101939 | 6006.2 | 796.8 | 655.2 | 732 | 598.7 | 66 |  |
| 480MA | 21Maranhão | Boa Vista do  Gurupi | 210197 | 2101970 | 8785.7 | 1165.6 | 958.4 | 1070.7 | 875.7 | 134 |  |
| 481MA | 21Maranhão | Bom Jardim | 210200 | 2102002 | 40447.1 | 5366.1 | 4412.4 | 4929.3 | 4031.6 | 208 |  |
| 482MA | 21Maranhão | Bom Jesus das  Selvas | 210203 | 2102036 | 31984.4 | 4243.4 | 3489.2 | 3898 | 3188.1 | 253 |  |
| 483MA | 21Maranhão | Bom Lugar | 210207 | 2102077 | 15701.3 | 2083.1 | 1712.9 | 1913.5 | 1565 | 47 |  |
| 484MA | 21Maranhão | Brejo | 210210 | 2102101 | 35119.7 | 4659.3 | 3831.2 | 4280.1 | 3500.6 | 259 |  |
| 485MA | 21Maranhão | Brejo de Areia | 210215 | 2102150 | 7154.6 | 949.2 | 780.5 | 871.9 | 713.1 | 132 |  |
| 486MA | 21Maranhão | Buriti | 210220 | 2102200 | 27899.1 | 3701.4 | 3043.5 | 3400.1 | 2780.9 | 264 |  |
| 487MA | 21Maranhão | Buriti Bravo | 210230 | 2102309 | 23359.3 | 3099.1 | 2548.3 | 2846.8 | 2328.4 | 895 |  |
| 488MA | 21Maranhão | Buriticupu | 210232 | 2102325 | 69481.8 | 9218.2 | 7579.8 | 8467.8 | 6925.6 | 386 |  |
| 489MA | 21Maranhão | Buritirana | 210235 | 2102358 | 15088 | 2001.7 | 1645.9 | 1838.8 | 1503.9 | 538 |  |
| 490MA | 21Maranhão | Cachoeira Grande | 210237 | 2102374 | 8848.4 | 1173.9 | 965.3 | 1078.4 | 882 | 119 |  |
| 491MA | 21Maranhão | Cajapió | 210240 | 2102408 | 10893.3 | 1445.2 | 1188.4 | 1327.6 | 1085.8 | 675 |  |
| 492MA | 21Maranhão | Cajari | 210250 | 2102507 | 18878.3 | 2504.6 | 2059.4 | 2300.7 | 1881.7 | 268 |  |
| 493MA | 21Maranhão | Campestre do  Maranhão | 210255 | 2102556 | 13930 | 1848.1 | 1519.6 | 1697.7 | 1388.5 | 408 |  |
| 494MA | 21Maranhão | Cândido Mendes | 210260 | 2102606 | 19512.4 | 2588.7 | 2128.6 | 2378 | 1944.9 | 109 |  |
| 495MA | 21Maranhão | Cantanhede | 210270 | 2102705 | 21312.5 | 2827.5 | 2325 | 2597.4 | 2124.3 | 274 |  |
| 496MA | 21Maranhão | Capinzal do Norte | 210275 | 2102754 | 10759.9 | 1427.5 | 1173.8 | 1311.3 | 1072.5 | 318 |  |
| 497MA | 21Maranhão | Carolina | 210280 | 2102804 | 23987.3 | 3182.4 | 2616.8 | 2923.3 | 2390.9 | 320 |  |
| 498MA | 21Maranhão | Carutapera | 210290 | 2102903 | 23028.5 | 3055.2 | 2512.2 | 2806.5 | 2295.4 | 89 |  |
| 499MA | 21Maranhão | Caxias | 210300 | 2103000 | 160402.6 | 21280.6 | 17498.3 | 19548.4 | 15988.2 | 38855 | 13 |
| 500MA | 21Maranhão | Cedral | 210310 | 2103109 | 10460.6 | 1387.8 | 1141.1 | 1274.8 | 1042.7 | 182 |  |
| 501MA | 21Maranhão | Central do  Maranhão | 210312 | 2103125 | 8349.4 | 1107.7 | 910.8 | 1017.5 | 832.2 | 238 |  |
| 502MA | 21Maranhão | Centro do  Guilherme | 210315 | 2103158 | 12918.4 | 1713.9 | 1409.3 | 1574.4 | 1287.6 | 38 |  |
| 503MA | 21Maranhão | Centro Novo do  Maranhão | 210317 | 2103174 | 20350.6 | 2699.9 | 2220 | 2480.1 | 2028.5 | 175 |  |
| 504MA | 21Maranhão | Chapadinha | 210320 | 2103208 | 76979.6 | 10212.9 | 8397.7 | 9381.6 | 7673 | 1555 |  |
| 505MA | 21Maranhão | Cidelândia | 210325 | 2103257 | 14247.6 | 1890.2 | 1554.3 | 1736.4 | 1420.1 | 367 |  |
| 506MA | 21Maranhão | Codó | 210330 | 2103307 | 120236.6 | 15951.8 | 13116.6 | 14653.3 | 11984.6 | 15387 |  |
| 507MA | 21Maranhão | Coelho Neto | 210340 | 2103406 | 48148.5 | 6387.9 | 5252.5 | 5867.9 | 4799.2 | 4586 |  |
| 508MA | 21Maranhão | Colinas | 210350 | 2103505 | 40165.7 | 5328.8 | 4381.7 | 4895 | 4003.5 | 2644 |  |
| 509MA | 21Maranhão | Conceição do  Lago-Açu | 210355 | 2103554 | 15528.7 | 2060.2 | 1694 | 1892.5 | 1547.8 | 138 |  |
| 510MA | 21Maranhão | Coroatá | 210360 | 2103604 | 63589.1 | 8436.4 | 6936.9 | 7749.6 | 6338.3 | 1557 |  |
| 511MA | 21Maranhão | Cururupu | 210370 | 2103703 | 31776.7 | 4215.8 | 3466.5 | 3872.6 | 3167.4 | 1490 |  |
| 512MA | 21Maranhão | Davinópolis | 210375 | 2103752 | 12687.9 | 1683.3 | 1384.1 | 1546.3 | 1264.7 | 357 |  |
| 513MA | 21Maranhão | Dom Pedro | 210380 | 2103802 | 22928.4 | 3041.9 | 2501.3 | 2794.3 | 2285.4 | 3328 |  |
| 514MA | 21Maranhão | Duque Bacelar | 210390 | 2103901 | 11027.1 | 1463 | 1202.9 | 1343.9 | 1099.1 | 258 |  |
| 515MA | 21Maranhão | Esperantinópolis | 210400 | 2104008 | 17423.9 | 2311.6 | 1900.8 | 2123.5 | 1736.7 | 140 |  |
| 516MA | 21Maranhão | Estreito | 210405 | 2104057 | 39661.9 | 5261.9 | 4326.7 | 4833.6 | 3953.3 | 588 |  |
| 517MA | 21Maranhão | Feira Nova do  Maranhão | 210407 | 2104073 | 8309.3 | 1102.4 | 906.5 | 1012.7 | 828.2 | 355 |  |
| 518MA | 21Maranhão | Fernando Falcão | 210408 | 2104081 | 9917.6 | 1315.8 | 1081.9 | 1208.7 | 988.5 | 11 |  |
| 519MA | 21Maranhão | Formosa da Serra  Negra | 210409 | 2104099 | 18436.1 | 2445.9 | 2011.2 | 2246.8 | 1837.6 | 58 |  |
| 520MA | 21Maranhão | Fortaleza dos  Nogueiras | 210410 | 2104107 | 12274.1 | 1628.4 | 1339 | 1495.9 | 1223.4 | 410 |  |
| 521MA | 21Maranhão | Fortuna | 210420 | 2104206 | 15268.7 | 2025.7 | 1665.7 | 1860.8 | 1521.9 | 1481 |  |
| 522MA | 21Maranhão | Godofredo Viana | 210430 | 2104305 | 11303.4 | 1499.6 | 1233.1 | 1377.6 | 1126.7 | 84 |  |
| 523MA | 21Maranhão | Gonçalves Dias | 210440 | 2104404 | 17629.5 | 2338.9 | 1923.2 | 2148.5 | 1757.2 | 318 |  |
| 524MA | 21Maranhão | Governador  Archer | 210450 | 2104503 | 10543.6 | 1398.8 | 1150.2 | 1285 | 1050.9 | 467 |  |
| 525MA | 21Maranhão | Governador  Edison Lobão | 210455 | 2104552 | 17381.8 | 2306 | 1896.2 | 2118.3 | 1732.5 | 427 |  |
| 526MA | 21Maranhão | Governador  Eugênio Barros | 210460 | 2104602 | 16414.2 | 2177.7 | 1790.6 | 2000.4 | 1636.1 | 333 |  |
| 527MA | 21Maranhão | Governador Luiz  Rocha | 210462 | 2104628 | 7589.2 | 1006.9 | 827.9 | 924.9 | 756.5 | 468 |  |
| 528MA | 21Maranhão | Governador  Newton Bello | 210465 | 2104651 | 10441.7 | 1385.3 | 1139.1 | 1272.5 | 1040.8 | 117 |  |
| 529MA | 21Maranhão | Governador Nunes  Freire | 210467 | 2104677 | 25288.8 | 3355.1 | 2758.8 | 3082 | 2520.7 | 185 |  |
| 530MA | 21Maranhão | Graça Aranha | 210470 | 2104701 | 6167.2 | 818.2 | 672.8 | 751.6 | 614.7 | 298 |  |
| 531MA | 21Maranhão | Grajaú | 210480 | 2104800 | 66461.6 | 8817.5 | 7250.3 | 8099.7 | 6624.6 | 576 |  |
| 532MA | 21Maranhão | Guimarães | 210490 | 2104909 | 11936 | 1583.5 | 1302.1 | 1454.6 | 1189.7 | 362 |  |
| 533MA | 21Maranhão | Humberto de  Campos | 210500 | 2105005 | 27668.4 | 3670.8 | 3018.3 | 3372 | 2757.9 | 234 |  |
| 534MA | 21Maranhão | Icatu | 210510 | 2105104 | 26252.7 | 3482.9 | 2863.9 | 3199.4 | 2616.8 | 179 |  |
| 535MA | 21Maranhão | Igarapé do Meio | 210515 | 2105153 | 13541.3 | 1796.5 | 1477.2 | 1650.3 | 1349.7 | 153 |  |
| 536MA | 21Maranhão | Igarapé Grande | 210520 | 2105203 | 11358.5 | 1506.9 | 1239.1 | 1384.3 | 1132.2 | 239 |  |
| 537MA | 21Maranhão | Imperatriz | 210530 | 2105302 | 252842.4 | 33544.6 | 27582.6 | 30814.1 | 25202.2 | 33357 | 12 |
| 538MA | 21Maranhão | Itaipava do Grajaú | 210535 | 2105351 | 14974.2 | 1986.6 | 1633.5 | 1824.9 | 1492.6 | 165 |  |
| 539MA | 21Maranhão | Itapecuru Mirim | 210540 | 2105401 | 65684.3 | 8714.3 | 7165.5 | 8005 | 6547.1 | 3320 |  |
| 540MA | 21Maranhão | Itinga do  Maranhão | 210542 | 2105427 | 25408.9 | 3371 | 2771.9 | 3096.6 | 2532.6 | 249 |  |
| 541MA | 21Maranhão | Jatobá | 210545 | 2105450 | 9553.8 | 1267.5 | 1042.2 | 1164.3 | 952.3 | 435 |  |
| 542MA | 21Maranhão | Jenipapo dos  Vieiras | 210547 | 2105476 | 16029.3 | 2126.6 | 1748.6 | 1953.5 | 1597.7 | 98 |  |
| 543MA | 21Maranhão | João Lisboa | 210550 | 2105500 | 23110.6 | 3066.1 | 2521.1 | 2816.5 | 2303.6 | 456 |  |
| 544MA | 21Maranhão | Joselândia | 210560 | 2105609 | 15804.7 | 2096.8 | 1724.1 | 1926.1 | 1575.3 | 156 |  |
| 545MA | 21Maranhão | Junco do  Maranhão | 210565 | 2105658 | 3584.4 | 475.5 | 391 | 436.8 | 357.3 | 235 |  |
| 546MA | 21Maranhão | Lago da Pedra | 210570 | 2105708 | 48506 | 6435.3 | 5291.5 | 5911.5 | 4834.9 | 299 |  |
| 547MA | 21Maranhão | Lago do Junco | 210580 | 2105807 | 10639 | 1411.5 | 1160.6 | 1296.6 | 1060.4 | 75 |  |
| 548MA | 21Maranhão | Lago Verde | 210590 | 2105906 | 15845.4 | 2102.2 | 1728.6 | 1931.1 | 1579.4 | 125 |  |
| 549MA | 21Maranhão | Lagoa do Mato | 210592 | 2105922 | 11043.9 | 1465.2 | 1204.8 | 1345.9 | 1100.8 | 529 |  |
| 550MA | 21Maranhão | Lago dos  Rodrigues | 210594 | 2105948 | 8465.7 | 1123.1 | 923.5 | 1031.7 | 843.8 | 54 |  |
| 551MA | 21Maranhão | Lagoa Grande do  Maranhão | 210596 | 2105963 | 11548.4 | 1532.1 | 1259.8 | 1407.4 | 1151.1 | 133 |  |
| 552MA | 21Maranhão | Lajeado Novo | 210598 | 2105989 | 7286.4 | 966.7 | 794.9 | 888 | 726.3 | 222 |  |
| 553MA | 21Maranhão | Lima Campos | 210600 | 2106003 | 11638.2 | 1544 | 1269.6 | 1418.4 | 1160 | 111 |  |
| 554MA | 21Maranhão | Loreto | 210610 | 2106102 | 11806.9 | 1566.4 | 1288 | 1438.9 | 1176.9 | 199 |  |
| 555MA | 21Maranhão | Luís Domingues | 210620 | 2106201 | 6750.2 | 895.5 | 736.4 | 822.7 | 672.8 | 55 |  |
| 556MA | 21Maranhão | Magalhães de  Almeida | 210630 | 2106300 | 18948.7 | 2513.9 | 2067.1 | 2309.3 | 1888.7 | 90 |  |
| 557MA | 21Maranhão | Maracaçumé | 210632 | 2106326 | 20511 | 2721.2 | 2237.5 | 2499.7 | 2044.4 | 188 |  |
| 558MA | 21Maranhão | Marajá do Sena | 210635 | 2106359 | 7773.4 | 1031.3 | 848 | 947.3 | 774.8 | 67 |  |
| 559MA |  | Maranhãozinho | 210637 | 2106375 | 15392 | 2042.1 | 1679.1 | 1875.8 | 1534.2 | 104 |  |
| 560MA |  | Mata Roma | 210640 | 2106409 | 16152.9 | 2143 | 1762.1 | 1968.6 | 1610 | 375 |  |
| 561MA |  | Matinha | 210650 | 2106508 | 22694.9 | 3010.9 | 2475.8 | 2765.8 | 2262.1 | 339 |  |

4

5

1

1

1

| 562MA | 21Maranhão | Matões | 210660 | 2106607 | 32680.3 | 4335.7 | 3565.1 | 3982.8 | 3257.4 | 1814 |  |
| --- | --- | --- | --- | --- | --- | --- | --- | --- | --- | --- | --- |
| 563MA | 21Maranhão | Matões do Norte | 210663 | 2106631 | 15673.6 | 2079.4 | 1709.8 | 1910.2 | 1562.3 | 225 |  |
| 564MA | 21Maranhão | Milagres do  Maranhão | 210667 | 2106672 | 8279.3 | 1098.4 | 903.2 | 1009 | 825.2 | 191 |  |
| 565MA | 21Maranhão | Mirador | 210670 | 2106706 | 20647.5 | 2739.3 | 2252.4 | 2516.3 | 2058.1 | 1021 |  |
| 566MA | 21Maranhão | Miranda do Norte | 210675 | 2106755 | 26891.4 | 3567.7 | 2933.6 | 3277.3 | 2680.4 | 35920 | 13 |
| 567MA | 21Maranhão | Mirinzal | 210680 | 2106805 | 14594.6 | 1936.3 | 1592.1 | 1778.7 | 1454.7 | 341 |  |
| 568MA | 21Maranhão | Monção | 210690 | 2106904 | 32581.6 | 4322.6 | 3554.3 | 3970.7 | 3247.6 | 202 |  |
| 569MA | 21Maranhão | Montes Altos | 210700 | 2107001 | 9155 | 1214.6 | 998.7 | 1115.7 | 912.5 | 266 |  |
| 570MA | 21Maranhão | Morros | 210710 | 2107100 | 18742.8 | 2486.6 | 2044.7 | 2284.2 | 1868.2 | 118 |  |
| 571MA | 21Maranhão | Nina Rodrigues | 210720 | 2107209 | 13703.2 | 1818 | 1494.9 | 1670 | 1365.9 | 199 |  |
| 572MA | 21Maranhão | Nova Colinas | 210725 | 2107258 | 5179.7 | 687.2 | 565.1 | 631.3 | 516.3 | 156 |  |
| 573MA | 21Maranhão | Nova Iorque | 210730 | 2107308 | 4610.9 | 611.7 | 503 | 561.9 | 459.6 | 206 |  |
| 574MA | 21Maranhão | Nova Olinda do  Maranhão | 210735 | 2107357 | 20179.2 | 2677.2 | 2201.3 | 2459.3 | 2011.4 | 36 |  |
| 575MA | 21Maranhão | Olho d'Água das  Cunhãs | 210740 | 2107407 | 19045.4 | 2526.8 | 2077.7 | 2321.1 | 1898.4 | 298 |  |
| 576MA | 21Maranhão | Olinda Nova do  Maranhão | 210745 | 2107456 | 14093.4 | 1869.8 | 1537.4 | 1717.6 | 1404.8 | 347 |  |
| 577MA | 21Maranhão | Paço do Lumiar | 210750 | 2107506 | 115530.7 | 15327.5 | 12603.2 | 14079.8 | 11515.6 | 6963 |  |
| 578MA | 21Maranhão | Palmeirândia | 210760 | 2107605 | 19245 | 2553.2 | 2099.4 | 2345.4 | 1918.3 | 263 |  |
| 579MA | 21Maranhão | Paraibano | 210770 | 2107704 | 20791.8 | 2758.4 | 2268.2 | 2533.9 | 2072.4 | 869 |  |
| 580MA | 21Maranhão | Parnarama | 210780 | 2107803 | 34344.5 | 4556.5 | 3746.6 | 4185.6 | 3423.3 | 4365 |  |
| 581MA | 21Maranhão | Passagem Franca | 210790 | 2107902 | 18391.9 | 2440.1 | 2006.4 | 2241.4 | 1833.2 | 590 |  |
| 582MA | 21Maranhão | Pastos Bons | 210800 | 2108009 | 18857 | 2501.8 | 2057.1 | 2298.1 | 1879.6 | 659 |  |
| 583MA | 21Maranhão | Paulino Neves | 210805 | 2108058 | 15416.1 | 2045.3 | 1681.7 | 1878.8 | 1536.6 | 32 |  |
| 584MA | 21Maranhão | Paulo Ramos | 210810 | 2108108 | 20568.5 | 2728.8 | 2243.8 | 2506.7 | 2050.2 | 171 |  |
| 585MA | 21Maranhão | Pedreiras | 210820 | 2108207 | 38997.7 | 5173.8 | 4254.3 | 4752.7 | 3887.1 | 652 |  |
| 586MA | 21Maranhão | Pedro do Rosário | 210825 | 2108256 | 24163.9 | 3205.8 | 2636 | 2944.9 | 2408.5 | 239 |  |
| 587MA | 21Maranhão | Penalva | 210830 | 2108306 | 36830.3 | 4886.3 | 4017.8 | 4488.5 | 3671.1 | 358 |  |
| 588MA | 21Maranhão | Peri Mirim | 210840 | 2108405 | 14025.5 | 1860.8 | 1530 | 1709.3 | 1398 | 233 |  |
| 589MA | 21Maranhão | Peritoró | 210845 | 2108454 | 22363.6 | 2967 | 2439.6 | 2725.5 | 2229.1 | 126 |  |
| 590MA | 21Maranhão | Pindaré-Mirim | 210850 | 2108504 | 32083.8 | 4256.6 | 3500 | 3910.1 | 3198 | 380 |  |
| 591MA | 21Maranhão | Pinheiro | 210860 | 2108603 | 80999.8 | 10746.2 | 8836.3 | 9871.5 | 8073.7 | 3912 |  |
| 592MA | 21Maranhão | Pio XII | 210870 | 2108702 | 21453.9 | 2846.3 | 2340.4 | 2614.6 | 2138.4 | 197 |  |
| 593MA | 21Maranhão | Pirapemas | 210880 | 2108801 | 18067.6 | 2397 | 1971 | 2201.9 | 1800.9 | 278 |  |
| 594MA | 21Maranhão | Poção de Pedras | 210890 | 2108900 | 18317.7 | 2430.2 | 1998.3 | 2232.4 | 1825.8 | 155 |  |
| 595MA | 21Maranhão | Porto Franco | 210900 | 2109007 | 22934 | 3042.7 | 2501.9 | 2795 | 2286 | 1978 |  |
| 596MA | 21Maranhão | Porto Rico do  Maranhão | 210905 | 2109056 | 5937.6 | 787.7 | 647.7 | 723.6 | 591.8 | 151 |  |
| 597MA | 21Maranhão | Presidente Dutra | 210910 | 2109106 | 46411.7 | 6157.4 | 5063.1 | 5656.2 | 4626.1 | 18853 |  |
| 598MA | 21Maranhão | Presidente  Juscelino | 210920 | 2109205 | 12246.7 | 1624.8 | 1336 | 1492.5 | 1220.7 | 288 |  |
| 599MA | 21Maranhão | Presidente Médici | 210923 | 2109239 | 6751.3 | 895.7 | 736.5 | 822.8 | 672.9 | 102 |  |
| 600MA | 21Maranhão | Presidente Sarney | 210927 | 2109270 | 18199.3 | 2414.5 | 1985.4 | 2218 | 1814 | 232 |  |
| 601MA | 21Maranhão | Presidente Vargas | 210930 | 2109304 | 11131.8 | 1476.9 | 1214.4 | 1356.6 | 1109.6 | 143 |  |
| 602MA | 21Maranhão | Primeira Cruz | 210940 | 2109403 | 14751.8 | 1957.1 | 1609.3 | 1797.8 | 1470.4 | 105 |  |
| 603MA | 21Maranhão | Raposa | 210945 | 2109452 | 29081.6 | 3858.3 | 3172.5 | 3544.2 | 2898.7 | 1250 |  |
| 604MA | 21Maranhão | Riachão | 210950 | 2109502 | 20014.5 | 2655.3 | 2183.4 | 2439.2 | 1995 | 1077 |  |
| 605MA | 21Maranhão | Ribamar Fiquene | 210955 | 2109551 | 7571.9 | 1004.6 | 826 | 922.8 | 754.7 | 205 |  |
| 606MA | 21Maranhão | Rosário | 210960 | 2109601 | 41365.3 | 5487.9 | 4512.5 | 5041.2 | 4123.1 | 648 |  |
| 607MA | 21Maranhão | Sambaíba | 210970 | 2109700 | 5561.4 | 737.8 | 606.7 | 677.8 | 554.3 | 258 |  |
| 608MA | 21Maranhão | Santa Filomena do  Maranhão | 210975 | 2109759 | 7481.2 | 992.5 | 816.1 | 911.7 | 745.7 | 27 |  |
| 609MA | 21Maranhão | Santa Helena | 210980 | 2109809 | 40907.4 | 5427.2 | 4462.6 | 4985.4 | 4077.5 | 365 |  |
| 610MA | 21Maranhão | Santa Inês | 210990 | 2109908 | 83146.5 | 11031 | 9070.5 | 10133.1 | 8287.7 | 3787 |  |
| 611MA | 21Maranhão | Santa Luzia | 211000 | 2110005 | 73377.1 | 9734.9 | 8004.7 | 8942.5 | 7313.9 | 357 |  |
| 612MA | 21Maranhão | Santa Luzia do  Paruá | 211003 | 2110039 | 24109.1 | 3198.6 | 2630.1 | 2938.2 | 2403.1 | 202 |  |
| 613MA | 21Maranhão | Santa Quitéria do  Maranhão | 211010 | 2110104 | 26838 | 3560.6 | 2927.8 | 3270.8 | 2675.1 | 98 |  |
| 614MA | 21Maranhão | Santa Rita | 211020 | 2110203 | 35428.5 | 4700.3 | 3864.9 | 4317.7 | 3531.4 | 485 |  |
| 615MA | 21Maranhão | Santana do  Maranhão | 211023 | 2110237 | 12727.2 | 1688.5 | 1388.4 | 1551.1 | 1268.6 | 93 |  |
| 616MA | 21Maranhão | Santo Amaro do  Maranhão | 211027 | 2110278 | 15070.8 | 1999.4 | 1644.1 | 1836.7 | 1502.2 | 37 |  |
| 617MA | 21Maranhão | Santo Antônio dos  Lopes | 211030 | 2110302 | 14320.6 | 1899.9 | 1562.2 | 1745.3 | 1427.4 | 804 |  |
| 618MA | 21Maranhão | São Benedito do  Rio Preto | 211040 | 2110401 | 18223.6 | 2417.7 | 1988 | 2220.9 | 1816.4 | 126 |  |
| 619MA | 21Maranhão | São Bento | 211050 | 2110500 | 43721.8 | 5800.6 | 4769.6 | 5328.4 | 4358 | 655 |  |
| 620MA | 21Maranhão | São Bernardo | 211060 | 2110609 | 27614.7 | 3663.6 | 3012.5 | 3365.4 | 2752.5 | 198 |  |
| 621MA | 21Maranhão | São Domingos do  Azeitão | 211065 | 2110658 | 7197 | 954.8 | 785.1 | 877.1 | 717.4 | 45 |  |
| 622MA | 21Maranhão | São Domingos do  Maranhão | 211070 | 2110708 | 33823.1 | 4487.3 | 3689.8 | 4122 | 3371.3 | 1784 |  |
| 623MA | 21Maranhão | São Félix de  Balsas | 211080 | 2110807 | 4580.2 | 607.7 | 499.7 | 558.2 | 456.5 | 373 |  |
| 624MA | 21Maranhão | São Francisco do  Brejão | 211085 | 2110856 | 11211.7 | 1487.5 | 1223.1 | 1366.4 | 1117.5 | 123 |  |
| 625MA | 21Maranhão | São Francisco do  Maranhão | 211090 | 2110906 | 12048.9 | 1598.5 | 1314.4 | 1468.4 | 1201 | 448 |  |
| 626MA | 21Maranhão | São João Batista | 211100 | 2111003 | 20244.4 | 2685.8 | 2208.5 | 2467.2 | 2017.9 | 199 |  |
| 627MA | 21Maranhão | São João do Carú | 211102 | 2111029 | 14946.4 | 1982.9 | 1630.5 | 1821.5 | 1489.8 | 17 |  |
| 628MA | 21Maranhão | São João do  Paraíso | 211105 | 2111052 | 10962.2 | 1454.4 | 1195.9 | 1336 | 1092.7 | 302 |  |
| 629MA | 21Maranhão | São João do Soter | 211107 | 2111078 | 17967.4 | 2383.7 | 1960.1 | 2189.7 | 1790.9 | 2594 |  |
| 630MA | 21Maranhão | São João dos  Patos | 211110 | 2111102 | 25366 | 3365.3 | 2767.2 | 3091.4 | 2528.4 | 598 |  |
| 631MA | 21Maranhão | São José de  Ribamar | 211120 | 2111201 | 171970.3 | 22815.3 | 18760.2 | 20958.1 | 17141.2 | 11747 |  |
| 632MA | 21Maranhão | São José dos  Basílios | 211125 | 2111250 | 7526.2 | 998.5 | 821 | 917.2 | 750.2 | 239 |  |
| 633MA | 21Maranhão | São Luís | 211130 | 2111300 | 1064524.2 | 141230.4 | 116128.9 | 129734.3 | 106107 | 133648 | 53 |
| 634MA | 21Maranhão | São Luís Gonzaga do Maranhão | 211140 | 2111409 | 19129.9 | 2538 | 2086.9 | 2331.4 | 1906.8 | 260 |  |
| 635MA | 21Maranhão | São Mateus do  Maranhão | 211150 | 2111508 | 40391.6 | 5358.8 | 4406.3 | 4922.6 | 4026.1 | 1150 |  |
| 636MA | 21Maranhão | São Pedro da  Água Branca | 211153 | 2111532 | 12368.6 | 1640.9 | 1349.3 | 1507.4 | 1232.8 | 153 |  |
| 637MA | 21Maranhão | São Pedro dos  Crentes | 211157 | 2111573 | 4549.5 | 603.6 | 496.3 | 554.5 | 453.5 | 58 |  |
| 638MA | 21Maranhão | São Raimundo das Mangabeiras | 211160 | 2111607 | 18262.1 | 2422.8 | 1992.2 | 2225.6 | 1820.3 | 651 |  |
| 639MA | 21Maranhão | São Raimundo do  Doca Bezerra | 211163 | 2111631 | 5454.9 | 723.7 | 595.1 | 664.8 | 543.7 | 133 |  |
| 640MA | 21Maranhão | São Roberto | 211167 | 2111672 | 6420.9 | 851.9 | 700.5 | 782.5 | 640 | 119 |  |
| 641MA | 21Maranhão | São Vicente Ferrer | 211170 | 2111706 | 21296.6 | 2825.4 | 2323.2 | 2595.4 | 2122.7 | 379 |  |
| 642MA | 21Maranhão | Satubinha | 211172 | 2111722 | 13188.7 | 1749.7 | 1438.8 | 1607.3 | 1314.6 | 118 |  |
| 643MA | 21Maranhão | Senador  Alexandre Costa | 211174 | 2111748 | 10763.9 | 1428 | 1174.2 | 1311.8 | 1072.9 | 746 |  |
| 644MA | 21Maranhão | Senador La  Rocque | 211176 | 2111763 | 14609.7 | 1938.3 | 1593.8 | 1780.5 | 1456.2 | 368 |  |
| 645MA |  | Serrano do  Maranhão | 211178 | 2111789 | 10732.7 | 1423.9 | 1170.8 | 1308 | 1069.8 | 202 |  |
| 646MA |  | Sítio Novo | 211180 | 2111805 | 17580.1 | 2332.4 | 1917.8 | 2142.5 | 1752.3 | 401 |  |
| 647MA |  | Sucupira do Norte | 211190 | 2111904 | 10479.5 | 1390.3 | 1143.2 | 1277.1 | 1044.5 | 413 |  |

2

1

1

7

1

5

| 648MA | 21Maranhão | Sucupira do  Riachão | 211195 | 2111953 | 5370.3 | 712.5 | 585.8 | 654.5 | 535.3 | 9 |  |
| --- | --- | --- | --- | --- | --- | --- | --- | --- | --- | --- | --- |
| 649MA | 21Maranhão | Tasso Fragoso | 211200 | 2112001 | 8217.7 | 1090.2 | 896.5 | 1001.5 | 819.1 | 372 |  |
| 650MA | 21Maranhão | Timbiras | 211210 | 2112100 | 28507.4 | 3782.1 | 3109.9 | 3474.2 | 2841.5 | 163 |  |
| 651MA | 21Maranhão | Timon | 211220 | 2112209 | 163329.5 | 21668.9 | 17817.6 | 19905.1 | 16280 | 40688 | 14 |
| 652MA | 21Maranhão | Trizidela do Vale | 211223 | 2112233 | 20529.8 | 2723.7 | 2239.6 | 2502 | 2046.3 | 306 |  |
| 653MA | 21Maranhão | Tufilândia | 211227 | 2112274 | 5711 | 757.7 | 623 | 696 | 569.2 | 17 |  |
| 654MA | 21Maranhão | Tuntum | 211230 | 2112308 | 40624.7 | 5389.7 | 4431.7 | 4951 | 4049.3 | 241 |  |
| 655MA | 21Maranhão | Turiaçu | 211240 | 2112407 | 34758.9 | 4611.5 | 3791.8 | 4236.1 | 3464.6 | 144 |  |
| 656MA | 21Maranhão | Turilândia | 211245 | 2112456 | 24522.2 | 3253.4 | 2675.1 | 2988.5 | 2444.3 | 53 |  |
| 657MA | 21Maranhão | Tutóia | 211250 | 2112506 | 56433.1 | 7487 | 6156.3 | 6877.5 | 5625 | 86 |  |
| 658MA | 21Maranhão | Urbano Santos | 211260 | 2112605 | 29759 | 3948.1 | 3246.4 | 3626.7 | 2966.2 | 225 |  |
| 659MA | 21Maranhão | Vargem Grande | 211270 | 2112704 | 53785.8 | 7135.8 | 5867.5 | 6554.9 | 5361.1 | 418 |  |
| 660MA | 21Maranhão | Viana | 211280 | 2112803 | 51045.9 | 6772.3 | 5568.6 | 6221 | 5088 | 689 |  |
| 661MA | 21Maranhão | Vila Nova dos  Martírios | 211285 | 2112852 | 12606 | 1672.4 | 1375.2 | 1536.3 | 1256.5 | 152 |  |
| 662MA | 21Maranhão | Vitória do Mearim | 211290 | 2112902 | 31982.8 | 4243.2 | 3489 | 3897.8 | 3187.9 | 2439 |  |
| 663MA | 21Maranhão | Vitorino Freire | 211300 | 2113009 | 31268.7 | 4148.4 | 3411.1 | 3810.7 | 3116.7 | 269 |  |
| 664MA | 21Maranhão | Zé Doca | 211400 | 2114007 | 50845.5 | 6745.7 | 5546.7 | 6196.6 | 5068.1 | 565 |  |
| 665PI | 22Piauí | Acauã | 220005 | 2200053 | 6918.6 | 1122.2 | 849.2 | 1018.7 | 761.4 | 776 |  |
| 666PI | 22Piauí | Agricolândia | 220010 | 2200103 | 5091.9 | 825.9 | 625 | 749.8 | 560.4 | 465 |  |
| 667PI | 22Piauí | Água Branca | 220020 | 2200202 | 16956.4 | 2750.3 | 2081.2 | 2496.7 | 1866 | 1876 |  |
| 668PI | 22Piauí | Alagoinha do Piauí | 220025 | 2200251 | 7491.2 | 1215.1 | 919.5 | 1103 | 824.4 | 652 |  |
| 669PI | 22Piauí | Alegrete do Piauí | 220027 | 2200277 | 5024.7 | 815 | 616.7 | 739.9 | 553 | 437 |  |
| 670PI | 22Piauí | Alto Longá | 220030 | 2200301 | 13977.8 | 2267.2 | 1715.6 | 2058.2 | 1538.2 | 902 |  |
| 671PI | 22Piauí | Altos | 220040 | 2200400 | 39656.9 | 6432.3 | 4867.5 | 5839.3 | 4364.2 | 3785 |  |
| 672PI | 22Piauí | Alvorada do  Gurguéia | 220045 | 2200459 | 5252.4 | 851.9 | 644.7 | 773.4 | 578 | 123 |  |
| 673PI | 22Piauí | Amarante | 220050 | 2200509 | 17320.6 | 2809.4 | 2125.9 | 2550.4 | 1906.1 | 714 |  |
| 674PI | 22Piauí | Angical do Piauí | 220060 | 2200608 | 6703.4 | 1087.3 | 822.8 | 987 | 737.7 | 358 |  |
| 675PI | 22Piauí | Anísio de Abreu | 220070 | 2200707 | 9536.8 | 1546.9 | 1170.5 | 1404.2 | 1049.5 | 560 |  |
| 676PI | 22Piauí | Antônio Almeida | 220080 | 2200806 | 3099.5 | 502.7 | 380.4 | 456.4 | 341.1 | 98 |  |
| 677PI | 22Piauí | Aroazes | 220090 | 2200905 | 5778.3 | 937.2 | 709.2 | 850.8 | 635.9 | 1312 |  |
| 678PI | 22Piauí | Aroeiras do Itaim | 220095 | 2200954 | 2477.5 | 401.9 | 304.1 | 364.8 | 272.6 | 105 |  |
| 679PI | 22Piauí | Arraial | 220100 | 2201002 | 4683 | 759.6 | 574.8 | 689.5 | 515.4 | 611 |  |
| 680PI | 22Piauí | Assunção do Piauí | 220105 | 2201051 | 7673.2 | 1244.6 | 941.8 | 1129.8 | 844.4 | 477 |  |
| 681PI | 22Piauí | Avelino Lopes | 220110 | 2201101 | 11316.8 | 1835.6 | 1389 | 1666.3 | 1245.4 | 154 |  |
| 682PI | 22Piauí | Baixa Grande do  Ribeiro | 220115 | 2201150 | 11128.7 | 1805.1 | 1365.9 | 1638.6 | 1224.7 | 64 |  |
| 683PI | 22Piauí | Barra D'Alcântara | 220117 | 2201176 | 3890.2 | 631 | 477.5 | 572.8 | 428.1 | 1975 |  |
| 684PI | 22Piauí | Barras | 220120 | 2201200 | 45973.5 | 7456.9 | 5642.8 | 6769.4 | 5059.3 | 4852 |  |
| 685PI | 22Piauí | Barreiras do Piauí | 220130 | 2201309 | 3285.8 | 533 | 403.3 | 483.8 | 361.6 | 19 |  |
| 686PI | 22Piauí | Barro Duro | 220140 | 2201408 | 6820.8 | 1106.3 | 837.2 | 1004.3 | 750.6 | 711 |  |
| 687PI | 22Piauí | Batalha | 220150 | 2201507 | 26298.3 | 4265.6 | 3227.9 | 3872.3 | 2894.1 | 2633 |  |
| 688PI | 22Piauí | Bela Vista do Piauí | 220155 | 2201556 | 3904.6 | 633.3 | 479.3 | 574.9 | 429.7 | 135 |  |
| 689PI | 22Piauí | Belém do Piauí | 220157 | 2201572 | 3442.4 | 558.4 | 422.5 | 506.9 | 378.8 | 153 |  |
| 690PI | 22Piauí | Beneditinos | 220160 | 2201606 | 10084.1 | 1635.6 | 1237.7 | 1484.8 | 1109.7 | 562 |  |
| 691PI | 22Piauí | Bertolínia | 220170 | 2201705 | 5400.8 | 876 | 662.9 | 795.2 | 594.4 | 847 |  |
| 692PI | 22Piauí | Betânia do Piauí | 220173 | 2201739 | 6097.4 | 989 | 748.4 | 897.8 | 671 | 603 |  |
| 693PI | 22Piauí | Boa Hora | 220177 | 2201770 | 6564.1 | 1064.7 | 805.7 | 966.5 | 722.4 | 1216 |  |
| 694PI | 22Piauí | Bocaina | 220180 | 2201804 | 4430 | 718.5 | 543.7 | 652.3 | 487.5 | 1458 |  |
| 695PI | 22Piauí | Bom Jesus | 220190 | 2201903 | 24104.2 | 3909.7 | 2958.5 | 3549.2 | 2652.6 | 841 |  |
| 696PI | 22Piauí | Bom Princípio do  Piauí | 220191 | 2201919 | 5477.5 | 888.5 | 672.3 | 806.5 | 602.8 | 266 |  |
| 697PI | 22Piauí | Bonfim do Piauí | 220192 | 2201929 | 5535.3 | 897.8 | 679.4 | 815 | 609.2 | 809 |  |
| 698PI | 22Piauí | Boqueirão do  Piauí | 220194 | 2201945 | 6317.5 | 1024.7 | 775.4 | 930.2 | 695.2 | 3142 |  |
| 699PI | 22Piauí | Brasileira | 220196 | 2201960 | 8145.7 | 1321.2 | 999.8 | 1199.4 | 896.4 | 1033 |  |
| 700PI | 22Piauí | Brejo do Piauí | 220198 | 2201988 | 3813.9 | 618.6 | 468.1 | 561.6 | 419.7 | 219 |  |
| 701PI | 22Piauí | Buriti dos Lopes | 220200 | 2202000 | 19402.3 | 3147.1 | 2381.4 | 2856.9 | 2135.2 | 1114 |  |
| 702PI | 22Piauí | Buriti dos Montes | 220202 | 2202026 | 8105.5 | 1314.7 | 994.9 | 1193.5 | 892 | 606 |  |
| 703PI | 22Piauí | Cabeceiras do  Piauí | 220205 | 2202059 | 10283.1 | 1667.9 | 1262.1 | 1514.1 | 1131.6 | 2622 |  |
| 704PI | 22Piauí | Cajazeiras do  Piauí | 220207 | 2202075 | 3458.4 | 561 | 424.5 | 509.2 | 380.6 | 206 |  |
| 705PI | 22Piauí | Cajueiro da Praia | 220208 | 2202083 | 7421.1 | 1203.7 | 910.9 | 1092.7 | 816.7 | 442 |  |
| 706PI | 22Piauí | Caldeirão Grande do Piauí | 220209 | 2202091 | 5705.8 | 925.5 | 700.3 | 840.2 | 627.9 | 186 |  |
| 707PI | 22Piauí | Campinas do Piauí | 220210 | 2202109 | 5503.3 | 892.6 | 675.5 | 810.3 | 605.6 | 394 |  |
| 708PI | 22Piauí | Campo Alegre do  Fidalgo | 220211 | 2202117 | 4886.4 | 792.6 | 599.8 | 719.5 | 537.7 | 447 |  |
| 709PI | 22Piauí | Campo Grande do  Piauí | 220213 | 2202133 | 5779.4 | 937.4 | 709.4 | 851 | 636 | 598 |  |
| 710PI | 22Piauí | Campo Largo do  Piauí | 220217 | 2202174 | 7062.3 | 1145.5 | 866.8 | 1039.9 | 777.2 | 502 |  |
| 711PI | 22Piauí | Campo Maior | 220220 | 2202208 | 45942.6 | 7451.9 | 5639 | 6764.8 | 5055.9 | 22690 |  |
| 712PI | 22Piauí | Canavieira | 220225 | 2202251 | 3915.1 | 635 | 480.5 | 576.5 | 430.9 | 522 |  |
| 713PI | 22Piauí | Canto do Buriti | 220230 | 2202307 | 20634.2 | 3346.9 | 2532.6 | 3038.3 | 2270.8 | 2315 |  |
| 714PI | 22Piauí | Capitão de  Campos | 220240 | 2202406 | 11192.7 | 1815.5 | 1373.8 | 1648.1 | 1231.7 | 4977 |  |
| 715PI | 22Piauí | Capitão Gervásio  Oliveira | 220245 | 2202455 | 3995.2 | 648 | 490.4 | 588.3 | 439.7 | 213 |  |
| 716PI | 22Piauí | Caracol | 220250 | 2202505 | 10594.7 | 1718.5 | 1300.4 | 1560 | 1165.9 | 184 |  |
| 717PI | 22Piauí | Caraúbas do Piauí | 220253 | 2202539 | 5707.9 | 925.8 | 700.6 | 840.5 | 628.1 | 426 |  |
| 718PI | 22Piauí | Caridade do Piauí | 220255 | 2202554 | 4964.1 | 805.2 | 609.3 | 730.9 | 546.3 | 1058 |  |
| 719PI | 22Piauí | Castelo do Piauí | 220260 | 2202604 | 18835.4 | 3055.1 | 2311.9 | 2773.4 | 2072.8 | 2035 |  |
| 720PI | 22Piauí | Caxingó | 220265 | 2202653 | 5251.5 | 851.8 | 644.6 | 773.3 | 577.9 | 302 |  |
| 721PI | 22Piauí | Cocal | 220270 | 2202703 | 27182.9 | 4409.1 | 3336.4 | 4002.6 | 2991.4 | 625 |  |
| 722PI | 22Piauí | Cocal de Telha | 220271 | 2202711 | 4686 | 760.1 | 575.2 | 690 | 515.7 | 1899 |  |
| 723PI | 22Piauí | Cocal dos Alves | 220272 | 2202729 | 5891.9 | 955.7 | 723.2 | 867.6 | 648.4 | 296 |  |
| 724PI | 22Piauí | Coivaras | 220273 | 2202737 | 3919.4 | 635.7 | 481.1 | 577.1 | 431.3 | 850 |  |
| 725PI | 22Piauí | Colônia do  Gurguéia | 220275 | 2202752 | 6282.4 | 1019 | 771.1 | 925.1 | 691.4 | 420 |  |
| 726PI | 22Piauí | Colônia do Piauí | 220277 | 2202778 | 7528 | 1221 | 924 | 1108.5 | 828.4 | 815 |  |
| 727PI | 22Piauí | Conceição do  Canindé | 220280 | 2202802 | 4591.9 | 744.8 | 563.6 | 676.1 | 505.3 | 358 |  |
| 728PI | 22Piauí | Coronel José Dias | 220285 | 2202851 | 4602 | 746.4 | 564.8 | 677.6 | 506.4 | 407 |  |
| 729PI | 22Piauí | Corrente | 220290 | 2202901 | 26031.3 | 4222.3 | 3195.1 | 3833 | 2864.7 | 332 |  |
| 730PI | 22Piauí | Cristalândia do  Piauí | 220300 | 2203008 | 8075.3 | 1309.8 | 991.2 | 1189 | 888.7 | 57 |  |
| 731PI | 22Piauí | Cristino Castro | 220310 | 2203107 | 10198.7 | 1654.2 | 1251.8 | 1501.7 | 1122.4 | 97 |  |
| 732PI | 22Piauí | Curimatá | 220320 | 2203206 | 11087.8 | 1798.4 | 1360.9 | 1632.6 | 1220.2 | 23 |  |
| 733PI | 22Piauí | Currais | 220323 | 2203230 | 4832.8 | 783.9 | 593.2 | 711.6 | 531.8 | 97 |  |
| 734PI | 22Piauí | Curralinhos | 220325 | 2203255 | 4321.3 | 700.9 | 530.4 | 636.3 | 475.6 | 299 |  |
| 735PI | 22Piauí | Curral Novo do  Piauí | 220327 | 2203271 | 5102.3 | 827.6 | 626.3 | 751.3 | 561.5 | 433 |  |
| 736PI | 22Piauí | Demerval Lobão | 220330 | 2203305 | 13536.9 | 2195.7 | 1661.5 | 1993.2 | 1489.7 | 1050 |  |
| 737PI | 22Piauí | Dirceu Arcoverde | 220335 | 2203354 | 6846.4 | 1110.5 | 840.3 | 1008.1 | 753.4 | 256 |  |
| 738PI | 22Piauí | Dom Expedito  Lopes | 220340 | 2203404 | 6740.2 | 1093.3 | 827.3 | 992.5 | 741.8 | 956 |  |
| 739PI | 22Piauí | Domingos Mourão | 220342 | 2203420 | 4294.6 | 696.6 | 527.1 | 632.4 | 472.6 | 184 |  |
| 740PI | 22Piauí | Dom Inocêncio | 220345 | 2203453 | 9384.1 | 1522.1 | 1151.8 | 1381.8 | 1032.7 | 767 |  |
| 741PI | 22Piauí | Elesbão Veloso | 220350 | 2203503 | 14476.9 | 2348.2 | 1776.9 | 2131.7 | 1593.2 | 1024 |  |
| 742PI | 22Piauí | Eliseu Martins | 220360 | 2203602 | 4794.7 | 777.7 | 588.5 | 706 | 527.7 | 299 |  |
| 743PI | 22Piauí | Esperantina | 220370 | 2203701 | 38778.7 | 6289.9 | 4759.7 | 5710 | 4267.5 | 2540 |  |
| 744PI | 22Piauí | Fartura do Piauí | 220375 | 2203750 | 5189.9 | 841.8 | 637 | 764.2 | 571.1 | 221 |  |

1

6

1

5

1

1

3

9

1

| 745PI | 22Piauí | Flores do Piauí | 220380 | 2203800 | 4399.7 | 713.6 | 540 | 647.8 | 484.2 | 218 |
| --- | --- | --- | --- | --- | --- | --- | --- | --- | --- | --- |
| 746PI | 22Piauí | Floresta do Piauí | 220385 | 2203859 | 2514.9 | 407.9 | 308.7 | 370.3 | 276.8 | 87 |
| 747PI | 22Piauí | Floriano | 220390 | 2203909 | 58750.3 | 9529.3 | 7211 | 8650.7 | 6465.4 | 17657 |
| 748PI | 22Piauí | Francinópolis | 220400 | 2204006 | 5273.3 | 855.3 | 647.2 | 776.5 | 580.3 | 724 |
| 749PI | 22Piauí | Francisco Ayres | 220410 | 2204105 | 4366.3 | 708.2 | 535.9 | 642.9 | 480.5 | 1394 |
| 750PI | 22Piauí | Francisco Macedo | 220415 | 2204154 | 3051.1 | 494.9 | 374.5 | 449.3 | 335.8 | 55 |
| 751PI | 22Piauí | Francisco Santos | 220420 | 2204204 | 8998.9 | 1459.6 | 1104.5 | 1325 | 990.3 | 1337 |
| 752PI | 22Piauí | Fronteiras | 220430 | 2204303 | 11380.6 | 1845.9 | 1396.9 | 1675.7 | 1252.4 | 272 |
| 753PI | 22Piauí | Geminiano | 220435 | 2204352 | 5335.3 | 865.4 | 654.9 | 785.6 | 587.1 | 640 |
| 754PI | 22Piauí | Gilbués | 220440 | 2204402 | 10518.9 | 1706.2 | 1291.1 | 1548.9 | 1157.6 | 17 |
| 755PI | 22Piauí | Guadalupe | 220450 | 2204501 | 10349.5 | 1678.7 | 1270.3 | 1523.9 | 1138.9 | 968 |
| 756PI | 22Piauí | Guaribas | 220455 | 2204550 | 4475.5 | 725.9 | 549.3 | 659 | 492.5 | 226 |
| 757PI | 22Piauí | Hugo Napoleão | 220460 | 2204600 | 3814.5 | 618.7 | 468.2 | 561.7 | 419.8 | 146 |
| 758PI | 22Piauí | Ilha Grande | 220465 | 2204659 | 9182.9 | 1489.5 | 1127.1 | 1352.1 | 1010.6 | 568 |
| 759PI | 22Piauí | Inhuma | 220470 | 2204709 | 15044.9 | 2440.3 | 1846.6 | 2215.3 | 1655.7 | 3455 |
| 760PI | 22Piauí | Ipiranga do Piauí | 220480 | 2204808 | 9575.6 | 1553.2 | 1175.3 | 1410 | 1053.8 | 1662 |
| 761PI | 22Piauí | Isaías Coelho | 220490 | 2204907 | 8390.9 | 1361 | 1029.9 | 1235.5 | 923.4 | 715 |
| 762PI | 22Piauí | Itainópolis | 220500 | 2205003 | 11323.4 | 1836.7 | 1389.8 | 1667.3 | 1246.1 | 2118 |
| 763PI | 22Piauí | Itaueira | 220510 | 2205102 | 10827.2 | 1756.2 | 1328.9 | 1594.3 | 1191.5 | 1286 |
| 764PI | 22Piauí | Jacobina do Piauí | 220515 | 2205151 | 5699.6 | 924.5 | 699.6 | 839.2 | 627.2 | 1545 |
| 765PI | 22Piauí | Jaicós | 220520 | 2205201 | 18599.3 | 3016.8 | 2282.9 | 2738.7 | 2046.8 | 1605 |
| 766PI | 22Piauí | Jardim do Mulato | 220525 | 2205250 | 4405.3 | 714.5 | 540.7 | 648.7 | 484.8 | 213 |
| 767PI | 22Piauí | Jatobá do Piauí | 220527 | 2205276 | 4759.5 | 772 | 584.2 | 700.8 | 523.8 | 2162 |
| 768PI | 22Piauí | Jerumenha | 220530 | 2205300 | 4401.5 | 713.9 | 540.2 | 648.1 | 484.4 | 1429 |
| 769PI | 22Piauí | João Costa | 220535 | 2205359 | 2971.2 | 481.9 | 364.7 | 437.5 | 327 | 468 |
| 770PI | 22Piauí | Joaquim Pires | 220540 | 2205409 | 14070.8 | 2282.3 | 1727 | 2071.9 | 1548.5 | 1321 |
| 771PI | 22Piauí | Joca Marques | 220545 | 2205458 | 5285.8 | 857.4 | 648.8 | 778.3 | 581.7 | 562 |
| 772PI | 22Piauí | José de Freitas | 220550 | 2205508 | 38197.6 | 6195.7 | 4688.4 | 5624.4 | 4203.6 | 1876 |
| 773PI | 22Piauí | Juazeiro do Piauí | 220551 | 2205516 | 5109.6 | 828.8 | 627.2 | 752.4 | 562.3 | 1226 |
| 774PI | 22Piauí | Júlio Borges | 220552 | 2205524 | 5500.2 | 892.1 | 675.1 | 809.9 | 605.3 | 34 |
| 775PI | 22Piauí | Jurema | 220553 | 2205532 | 4644.4 | 753.3 | 570.1 | 683.9 | 511.1 | 251 |
| 776PI | 22Piauí | Lagoinha do Piauí | 220554 | 2205540 | 2759.3 | 447.6 | 338.7 | 406.3 | 303.7 | 234 |
| 777PI | 22Piauí | Lagoa Alegre | 220555 | 2205557 | 8296 | 1345.6 | 1018.3 | 1221.5 | 913 | 269 |
| 778PI | 22Piauí | Lagoa do Barro do  Piauí | 220556 | 2205565 | 4577.4 | 742.5 | 561.8 | 674 | 503.7 | 446 |
| 779PI | 22Piauí | Lagoa de São  Francisco | 220557 | 2205573 | 6594.8 | 1069.7 | 809.4 | 971.1 | 725.7 | 628 |
| 780PI | 22Piauí | Lagoa do Piauí | 220558 | 2205581 | 3966 | 643.3 | 486.8 | 584 | 436.5 | 304 |
| 781PI | 22Piauí | Lagoa do Sítio | 220559 | 2205599 | 5026.3 | 815.3 | 616.9 | 740.1 | 553.1 | 1159 |
| 782PI | 22Piauí | Landri Sales | 220560 | 2205607 | 5256.5 | 852.6 | 645.2 | 774 | 578.5 | 867 |
| 783PI | 22Piauí | Luís Correia | 220570 | 2205706 | 29435.3 | 4774.4 | 3612.9 | 4334.2 | 3239.3 | 1494 |
| 784PI | 22Piauí | Luzilândia | 220580 | 2205805 | 25050.2 | 4063.1 | 3074.7 | 3688.5 | 2756.7 | 1904 |
| 785PI | 22Piauí | Madeiro | 220585 | 2205854 | 8080.5 | 1310.7 | 991.8 | 1189.8 | 889.2 | 986 |
| 786PI | 22Piauí | Manoel Emídio | 220590 | 2205904 | 5266.7 | 854.3 | 646.4 | 775.5 | 579.6 | 548 |
| 787PI | 22Piauí | Marcolândia | 220595 | 2205953 | 8193.6 | 1329 | 1005.7 | 1206.5 | 901.7 | 770 |
| 788PI | 22Piauí | Marcos Parente | 220600 | 2206001 | 4486.6 | 727.7 | 550.7 | 660.6 | 493.7 | 1104 |
| 789PI | 22Piauí | Massapê do Piauí | 220605 | 2206050 | 6320.8 | 1025.2 | 775.8 | 930.7 | 695.6 | 491 |
| 790PI | 22Piauí | Matias Olímpio | 220610 | 2206100 | 10701.2 | 1735.7 | 1313.5 | 1575.7 | 1177.7 | 939 |
| 791PI | 22Piauí | Miguel Alves | 220620 | 2206209 | 33019 | 5355.7 | 4052.8 | 4861.9 | 3633.7 | 821 |
| 792PI | 22Piauí | Miguel Leão | 220630 | 2206308 | 1240.6 | 201.2 | 152.3 | 182.7 | 136.5 | 151 |
| 793PI | 22Piauí | Milton Brandão | 220635 | 2206357 | 6663.7 | 1080.9 | 817.9 | 981.2 | 733.3 | 857 |
| 794PI | 22Piauí | Monsenhor Gil | 220640 | 2206407 | 10418.3 | 1689.8 | 1278.7 | 1534 | 1146.5 | 722 |
| 795PI | 22Piauí | Monsenhor  Hipólito | 220650 | 2206506 | 7571.2 | 1228 | 929.3 | 1114.8 | 833.2 | 491 |
| 796PI | 22Piauí | Monte Alegre do  Piauí | 220660 | 2206605 | 10450.3 | 1695 | 1282.7 | 1538.8 | 1150 | 51 |
| 797PI | 22Piauí | Morro Cabeça no  Tempo | 220665 | 2206654 | 4161.4 | 675 | 510.8 | 612.7 | 458 | 3 |
| 798PI | 22Piauí | Morro do Chapéu do Piauí | 220667 | 2206670 | 6646.3 | 1078 | 815.8 | 978.6 | 731.4 | 1004 |
| 799PI | 22Piauí | Murici dos  Portelas | 220669 | 2206696 | 8851.4 | 1435.7 | 1086.4 | 1303.3 | 974.1 | 336 |
| 800PI | 22Piauí | Nazaré do Piauí | 220670 | 2206704 | 7286.4 | 1181.9 | 894.3 | 1072.9 | 801.9 | 1878 |
| 801PI | 22Piauí | Nazária | 220672 | 2206720 | 8335.8 | 1352.1 | 1023.1 | 1227.4 | 917.3 | 349 |
| 802PI | 22Piauí | Nossa Senhora de  Nazaré | 220675 | 2206753 | 4726.3 | 766.6 | 580.1 | 695.9 | 520.1 | 1674 |
| 803PI | 22Piauí | Nossa Senhora dos Remédios | 220680 | 2206803 | 8463.2 | 1372.7 | 1038.8 | 1246.2 | 931.4 | 793 |
| 804PI | 22Piauí | Novo Oriente do  Piauí | 220690 | 2206902 | 6499.6 | 1054.2 | 797.8 | 957 | 715.3 | 1582 |
| 805PI | 22Piauí | Novo Santo  Antônio | 220695 | 2206951 | 3183.6 | 516.4 | 390.8 | 468.8 | 350.4 | 390 |
| 806PI | 22Piauí | Oeiras | 220700 | 2207009 | 36296.6 | 5887.3 | 4455 | 5344.5 | 3994.4 | 5047 |
| 807PI | 22Piauí | Olho D'Água do  Piauí | 220710 | 2207108 | 2517.8 | 408.4 | 309 | 370.7 | 277.1 | 261 |
| 808PI | 22Piauí | Padre Marcos | 220720 | 2207207 | 6748.2 | 1094.6 | 828.3 | 993.6 | 742.6 | 918 |
| 809PI | 22Piauí | Paes Landim | 220730 | 2207306 | 4077.7 | 661.4 | 500.5 | 600.4 | 448.7 | 174 |
| 810PI | 22Piauí | Pajeú do Piauí | 220735 | 2207355 | 3386.1 | 549.2 | 415.6 | 498.6 | 372.6 | 975 |
| 811PI | 22Piauí | Palmeira do Piauí | 220740 | 2207405 | 4993.1 | 809.9 | 612.9 | 735.2 | 549.5 | 67 |
| 812PI | 22Piauí | Palmeirais | 220750 | 2207504 | 14162 | 2297.1 | 1738.2 | 2085.3 | 1558.5 | 748 |
| 813PI | 22Piauí | Paquetá | 220755 | 2207553 | 3935.1 | 638.3 | 483 | 579.4 | 433.1 | 564 |
| 814PI | 22Piauí | Parnaguá | 220760 | 2207603 | 10538 | 1709.3 | 1293.4 | 1551.7 | 1159.7 | 104 |
| 815PI | 22Piauí | Parnaíba | 220770 | 2207702 | 149463.4 | 24243 | 18345.1 | 22007.8 | 16448.3 | 13877 |
| 816PI | 22Piauí | Passagem Franca do Piauí | 220775 | 2207751 | 4455.8 | 722.7 | 546.9 | 656.1 | 490.4 | 203 |
| 817PI | 22Piauí | Patos do Piauí | 220777 | 2207777 | 6247.4 | 1013.3 | 766.8 | 919.9 | 687.5 | 921 |
| 818PI | 22Piauí | Pau D'Arco do  Piauí | 220779 | 2207793 | 3916.2 | 635.2 | 480.7 | 576.6 | 431 | 128 |
| 819PI | 22Piauí | Paulistana | 220780 | 2207801 | 20139.6 | 3266.6 | 2471.9 | 2965.5 | 2216.3 | 2489 |
| 820PI | 22Piauí | Pavussu | 220785 | 2207850 | 3648.9 | 591.9 | 447.9 | 537.3 | 401.6 | 704 |
| 821PI | 22Piauí | Pedro II | 220790 | 2207900 | 38048.3 | 6171.4 | 4670 | 5602.4 | 4187.2 | 3706 |
| 822PI | 22Piauí | Pedro Laurentino | 220793 | 2207934 | 2474.2 | 401.3 | 303.7 | 364.3 | 272.3 | 86 |
| 823PI | 22Piauí | Nova Santa Rita | 220795 | 2207959 | 4279.2 | 694.1 | 525.2 | 630.1 | 470.9 | 125 |
| 824PI | 22Piauí | Picos | 220800 | 2208007 | 76265.7 | 12370.3 | 9360.9 | 11229.8 | 8392.9 | 35456 |
| 825PI | 22Piauí | Pimenteiras | 220810 | 2208106 | 11909.6 | 1931.7 | 1461.8 | 1753.6 | 1310.6 | 1364 |
| 826PI | 22Piauí | Pio IX | 220820 | 2208205 | 18037.6 | 2925.7 | 2213.9 | 2655.9 | 1985 | 1530 |
| 827PI | 22Piauí | Piracuruca | 220830 | 2208304 | 28174.2 | 4569.9 | 3458.1 | 4148.5 | 3100.5 | 2863 |
| 828PI | 22Piauí | Piripiri | 220840 | 2208403 | 62654.1 | 10162.5 | 7690.2 | 9225.5 | 6895 | 9267 |
| 829PI | 22Piauí | Porto | 220850 | 2208502 | 12248 | 1986.6 | 1503.3 | 1803.5 | 1347.9 | 727 |
| 830PI | 22Piauí | Porto Alegre do  Piauí | 220855 | 2208551 | 2638.7 | 428 | 323.9 | 388.5 | 290.4 | 226 |
| 831PI | 22Piauí | Prata do Piauí | 220860 | 2208601 | 3108.9 | 504.3 | 381.6 | 457.8 | 342.1 | 115 |
| 832PI | 22Piauí | Queimada Nova | 220865 | 2208650 | 8778.4 | 1423.9 | 1077.5 | 1292.6 | 966.1 | 781 |
| 833PI | 22Piauí | Redenção do  Gurguéia | 220870 | 2208700 | 8586.6 | 1392.7 | 1053.9 | 1264.3 | 944.9 | 101 |
| 834PI | 22Piauí | Regeneração | 220880 | 2208809 | 17712.8 | 2873 | 2174.1 | 2608.1 | 1949.3 | 687 |
| 835PI | 22Piauí | Riacho Frio | 220885 | 2208858 | 4259.3 | 690.9 | 522.8 | 627.2 | 468.7 | 39 |
| 836PI | 22Piauí | Ribeira do Piauí | 220887 | 2208874 | 4371.6 | 709.1 | 536.6 | 643.7 | 481.1 | 232 |
| 837PI | 22Piauí | Ribeiro Gonçalves | 220890 | 2208908 | 7116.7 | 1154.3 | 873.5 | 1047.9 | 783.2 | 201 |
| 838PI | 22Piauí | Rio Grande do  Piauí | 220900 | 2209005 | 6334 | 1027.4 | 777.4 | 932.7 | 697 | 561 |
| 839PI | 22Piauí | Santa Cruz do  Piauí | 220910 | 2209104 | 6124 | 993.3 | 751.7 | 901.7 | 673.9 | 818 |
| 840PI | 22Piauí | Santa Cruz dos  Milagres | 220915 | 2209153 | 3913.1 | 634.7 | 480.3 | 576.2 | 430.6 | 421 |

| 841PI | 22Piauí | Santa Filomena | 220920 | 2209203 | 6157.4 | 998.7 | 755.8 | 906.6 | 677.6 | 21 |  |
| --- | --- | --- | --- | --- | --- | --- | --- | --- | --- | --- | --- |
| 842PI | 22Piauí | Santa Luz | 220930 | 2209302 | 5698.3 | 924.3 | 699.4 | 839 | 627.1 | 76 |  |
| 843PI | 22Piauí | Santana do Piauí | 220935 | 2209351 | 4584.3 | 743.6 | 562.7 | 675 | 504.5 | 339 |  |
| 844PI | 22Piauí | Santa Rosa do  Piauí | 220937 | 2209377 | 5184.8 | 841 | 636.4 | 763.4 | 570.6 | 319 |  |
| 845PI | 22Piauí | Santo Antônio de  Lisboa | 220940 | 2209401 | 6223.4 | 1009.4 | 763.9 | 916.4 | 684.9 | 534 |  |
| 846PI | 22Piauí | Santo Antônio dos  Milagres | 220945 | 2209450 | 2110.6 | 342.3 | 259.1 | 310.8 | 232.3 | 42 |  |
| 847PI | 22Piauí | Santo Inácio do  Piauí | 220950 | 2209500 | 3716.9 | 602.9 | 456.2 | 547.3 | 409 | 107 |  |
| 848PI | 22Piauí | São Braz do Piauí | 220955 | 2209559 | 4371.1 | 709 | 536.5 | 643.6 | 481 | 553 |  |
| 849PI | 22Piauí | São Félix do Piauí | 220960 | 2209609 | 2954 | 479.1 | 362.6 | 435 | 325.1 | 589 |  |
| 850PI | 22Piauí | São Francisco de  Assis do Piauí | 220965 | 2209658 | 5710.6 | 926.3 | 700.9 | 840.9 | 628.4 | 266 |  |
| 851PI | 22Piauí | São Francisco do  Piauí | 220970 | 2209708 | 6337.6 | 1028 | 777.9 | 933.2 | 697.4 | 910 |  |
| 852PI | 22Piauí | São Gonçalo do  Gurguéia | 220975 | 2209757 | 2944.4 | 477.6 | 361.4 | 433.5 | 324 | 40 |  |
| 853PI | 22Piauí | São Gonçalo do  Piauí | 220980 | 2209807 | 4889.5 | 793.1 | 600.1 | 720 | 538.1 | 194 |  |
| 854PI | 22Piauí | São João da  Canabrava | 220985 | 2209856 | 4520.3 | 733.2 | 554.8 | 665.6 | 497.5 | 1062 |  |
| 855PI | 22Piauí | São João da  Fronteira | 220987 | 2209872 | 5843.8 | 947.9 | 717.3 | 860.5 | 643.1 | 580 |  |
| 856PI | 22Piauí | São João da Serra | 220990 | 2209906 | 6106.7 | 990.5 | 749.5 | 899.2 | 672 | 314 |  |
| 857PI | 22Piauí | São João da  Varjota | 220995 | 2209955 | 4741.8 | 769.1 | 582 | 698.2 | 521.8 | 447 |  |
| 858PI | 22Piauí | São João do  Arraial | 220997 | 2209971 | 7704.1 | 1249.6 | 945.6 | 1134.4 | 847.8 | 865 |  |
| 859PI | 22Piauí | São João do Piauí | 221000 | 2210003 | 20092.7 | 3259 | 2466.2 | 2958.6 | 2211.2 | 402 |  |
| 860PI | 22Piauí | São José do  Divino | 221005 | 2210052 | 5241.2 | 850.1 | 643.3 | 771.7 | 576.8 | 642 |  |
| 861PI | 22Piauí | São José do Peixe | 221010 | 2210102 | 3705.4 | 601 | 454.8 | 545.6 | 407.8 | 407 |  |
| 862PI | 22Piauí | São José do Piauí | 221020 | 2210201 | 6621.6 | 1074 | 812.7 | 975 | 728.7 | 403 |  |
| 863PI | 22Piauí | São Julião | 221030 | 2210300 | 6009.7 | 974.8 | 737.6 | 884.9 | 661.4 | 290 |  |
| 864PI | 22Piauí | São Lourenço do  Piauí | 221035 | 2210359 | 4492 | 728.6 | 551.3 | 661.4 | 494.3 | 371 |  |
| 865PI | 22Piauí | São Luis do Piauí | 221037 | 2210375 | 2596.5 | 421.2 | 318.7 | 382.3 | 285.7 | 745 |  |
| 866PI | 22Piauí | São Miguel da  Baixa Grande | 221038 | 2210383 | 2355.7 | 382.1 | 289.1 | 346.9 | 259.2 | 116 |  |
| 867PI | 22Piauí | São Miguel do  Fidalgo | 221039 | 2210391 | 2996.9 | 486.1 | 367.8 | 441.3 | 329.8 | 61 |  |
| 868PI | 22Piauí | São Miguel do  Tapuio | 221040 | 2210409 | 17899.5 | 2903.3 | 2197 | 2635.6 | 1969.8 | 2455 |  |
| 869PI | 22Piauí | São Pedro do  Piauí | 221050 | 2210508 | 13966.7 | 2265.4 | 1714.3 | 2056.5 | 1537 | 655 |  |
| 870PI | 22Piauí | São Raimundo  Nonato | 221060 | 2210607 | 33635.4 | 5455.7 | 4128.4 | 4952.6 | 3701.5 | 7665 |  |
| 871PI | 22Piauí | Sebastião Barros | 221062 | 2210623 | 3479.6 | 564.4 | 427.1 | 512.4 | 382.9 | 15 |  |
| 872PI | 22Piauí | Sebastião Leal | 221063 | 2210631 | 4203.1 | 681.7 | 515.9 | 618.9 | 462.5 | 1318 |  |
| 873PI | 22Piauí | Sigefredo Pacheco | 221065 | 2210656 | 9822.8 | 1593.3 | 1205.7 | 1446.4 | 1081 | 2595 |  |
| 874PI | 22Piauí | Simões | 221070 | 2210706 | 14387.7 | 2333.7 | 1765.9 | 2118.5 | 1583.3 | 1594 |  |
| 875PI | 22Piauí | Simplício Mendes | 221080 | 2210805 | 12396.3 | 2010.7 | 1521.5 | 1825.3 | 1364.2 | 248 |  |
| 876PI | 22Piauí | Socorro do Piauí | 221090 | 2210904 | 4523.4 | 733.7 | 555.2 | 666 | 497.8 | 393 |  |
| 877PI | 22Piauí | Sussuapara | 221093 | 2210938 | 6507.9 | 1055.6 | 798.8 | 958.3 | 716.2 | 533 |  |
| 878PI | 22Piauí | Tamboril do Piauí | 221095 | 2210953 | 2841.1 | 460.8 | 348.7 | 418.3 | 312.7 | 243 |  |
| 879PI | 22Piauí | Tanque do Piauí | 221097 | 2210979 | 2695.7 | 437.2 | 330.9 | 396.9 | 296.7 | 406 |  |
| 880PI | 22Piauí | Teresina | 221100 | 2211001 | 841205.9 | 136443.6 | 103249.6 | 123863.5 | 92573.6 | 173708 | 68 |
| 881PI | 22Piauí | União | 221110 | 2211100 | 43546.3 | 7063.2 | 5344.9 | 6412 | 4792.2 | 1979 |  |
| 882PI | 22Piauí | Uruçuí | 221120 | 2211209 | 20916.2 | 3392.6 | 2567.3 | 3079.8 | 2301.8 | 200 |  |
| 883PI | 22Piauí | Valença do Piauí | 221130 | 2211308 | 20573.4 | 3337 | 2525.2 | 3029.3 | 2264.1 | 5560 |  |
| 884PI | 22Piauí | Várzea Branca | 221135 | 2211357 | 4903.7 | 795.4 | 601.9 | 722 | 539.6 | 356 |  |
| 885PI | 22Piauí | Várzea Grande | 221140 | 2211407 | 4343.8 | 704.6 | 533.2 | 639.6 | 478 | 612 |  |
| 886PI | 22Piauí | Vera Mendes | 221150 | 2211506 | 3025.3 | 490.7 | 371.3 | 445.5 | 332.9 | 674 |  |
| 887PI | 22Piauí | Vila Nova do Piauí | 221160 | 2211605 | 2989.9 | 485 | 367 | 440.2 | 329 | 993 |  |
| 888PI | 22Piauí | Wall Ferraz | 221170 | 2211704 | 4368.9 | 708.6 | 536.2 | 643.3 | 480.8 | 546 |  |
| 889CE | 23Ceará | Abaiara | 230010 | 2300101 | 11212.9 | 1803.4 | 1421.5 | 1521.3 | 1196.9 | 1324 |  |
| 890CE | 23Ceará | Acarape | 230015 | 2300150 | 15950 | 2565.2 | 2022 | 2164 | 1702.5 | 1162 |  |
| 891CE | 23Ceará | Acaraú | 230020 | 2300200 | 60575.2 | 9742.3 | 7679.1 | 8218.6 | 6465.8 | 3867 |  |
| 892CE | 23Ceará | Acopiara | 230030 | 2300309 | 52822.7 | 8495.5 | 6696.3 | 7166.8 | 5638.3 | 3387 |  |
| 893CE | 23Ceará | Aiuaba | 230040 | 2300408 | 16867.6 | 2712.8 | 2138.3 | 2288.5 | 1800.5 | 966 |  |
| 894CE | 23Ceará | Alcântaras | 230050 | 2300507 | 11242.4 | 1808.1 | 1425.2 | 1525.3 | 1200 | 1936 |  |
| 895CE | 23Ceará | Altaneira | 230060 | 2300606 | 7264.5 | 1168.3 | 920.9 | 985.6 | 775.4 | 630 |  |
| 896CE | 23Ceará | Alto Santo | 230070 | 2300705 | 16790.8 | 2700.5 | 2128.6 | 2278.1 | 1792.3 | 2989 |  |
| 897CE | 23Ceará | Amontada | 230075 | 2300754 | 41625.7 | 6694.7 | 5276.9 | 5647.6 | 4443.1 | 3118 |  |
| 898CE | 23Ceará | Antonina do Norte | 230080 | 2300804 | 7187.1 | 1155.9 | 911.1 | 975.1 | 767.2 | 424 |  |
| 899CE | 23Ceará | Apuiarés | 230090 | 2300903 | 14431.3 | 2321 | 1829.5 | 1958 | 1540.4 | 1665 |  |
| 900CE | 23Ceará | Aquiraz | 230100 | 2301000 | 76892.4 | 12366.6 | 9747.6 | 10432.5 | 8207.5 | 5749 |  |
| 901CE | 23Ceará | Aracati | 230110 | 2301109 | 72146.5 | 11603.3 | 9146 | 9788.6 | 7700.9 | 7626 |  |
| 902CE | 23Ceará | Aracoiaba | 230120 | 2301208 | 26005.6 | 4182.5 | 3296.7 | 3528.3 | 2775.8 | 7862 |  |
| 903CE | 23Ceará | Ararendá | 230125 | 2301257 | 10729 | 1725.5 | 1360.1 | 1455.7 | 1145.2 | 190 |  |
| 904CE | 23Ceará | Araripe | 230130 | 2301307 | 21188.3 | 3407.7 | 2686 | 2874.7 | 2261.6 | 1207 |  |
| 905CE | 23Ceará | Aratuba | 230140 | 2301406 | 11439.4 | 1839.8 | 1450.2 | 1552.1 | 1221 | 3480 |  |
| 906CE | 23Ceará | Arneiroz | 230150 | 2301505 | 7750.8 | 1246.6 | 982.6 | 1051.6 | 827.3 | 420 |  |
| 907CE | 23Ceará | Assaré | 230160 | 2301604 | 23006.9 | 3700.2 | 2916.6 | 3121.5 | 2455.8 | 1505 |  |
| 908CE | 23Ceará | Aurora | 230170 | 2301703 | 24592.6 | 3955.2 | 3117.6 | 3336.6 | 2625 | 2362 |  |
| 909CE | 23Ceará | Baixio | 230180 | 2301802 | 6169.4 | 992.2 | 782.1 | 837 | 658.5 | 550 |  |
| 910CE | 23Ceará | Banabuiú | 230185 | 2301851 | 17807.2 | 2863.9 | 2257.4 | 2416 | 1900.7 | 2780 |  |
| 911CE | 23Ceará | Barbalha | 230190 | 2301901 | 58191.9 | 9359 | 7377 | 7895.3 | 6211.4 | 8426 |  |
| 912CE | 23Ceará | Barreira | 230195 | 2301950 | 20623.2 | 3316.8 | 2614.4 | 2798.1 | 2201.3 | 1341 |  |
| 913CE | 23Ceará | Barro | 230200 | 2302008 | 22153.6 | 3563 | 2808.4 | 3005.7 | 2364.7 | 1949 |  |
| 914CE | 23Ceará | Barroquinha | 230205 | 2302057 | 14769.4 | 2375.4 | 1872.3 | 2003.9 | 1576.5 | 1097 |  |
| 915CE | 23Ceará | Baturité | 230210 | 2302107 | 34680.6 | 5577.7 | 4396.5 | 4705.3 | 3701.8 | 12560 |  |
| 916CE | 23Ceará | Beberibe | 230220 | 2302206 | 51798.1 | 8330.7 | 6566.4 | 7027.8 | 5528.9 | 4431 |  |
| 917CE | 23Ceará | Bela Cruz | 230230 | 2302305 | 31887.6 | 5128.5 | 4042.4 | 4326.4 | 3403.7 | 2074 |  |
| 918CE | 23Ceará | Boa Viagem | 230240 | 2302404 | 53606.8 | 8621.6 | 6795.7 | 7273.2 | 5722 | 2670 |  |
| 919CE | 23Ceará | Brejo Santo | 230250 | 2302503 | 47589.4 | 7653.8 | 6032.9 | 6456.7 | 5079.7 | 5694 |  |
| 920CE | 23Ceará | Camocim | 230260 | 2302602 | 62092.8 | 9986.4 | 7871.5 | 8424.5 | 6627.8 | 6600 |  |
| 921CE | 23Ceará | Campos Sales | 230270 | 2302701 | 27017.3 | 4345.2 | 3425 | 3665.6 | 2883.8 | 756 |  |
| 922CE | 23Ceará | Canindé | 230280 | 2302800 | 76451.1 | 12295.6 | 9691.7 | 10372.6 | 8160.4 | 2499 |  |
| 923CE | 23Ceará | Capistrano | 230290 | 2302909 | 17478.3 | 2811 | 2215.7 | 2371.4 | 1865.6 | 4986 |  |
| 924CE | 23Ceará | Caridade | 230300 | 2303006 | 21498.5 | 3457.6 | 2725.4 | 2916.8 | 2294.8 | 975 |  |
| 925CE | 23Ceará | Cariré | 230310 | 2303105 | 18558.1 | 2984.7 | 2352.6 | 2517.9 | 1980.9 | 1201 |  |
| 926CE | 23Ceará | Caririaçu | 230320 | 2303204 | 26764.3 | 4304.5 | 3392.9 | 3631.3 | 2856.8 | 2046 |  |
| 927CE | 23Ceará | Cariús | 230330 | 2303303 | 18742.7 | 3014.4 | 2376 | 2542.9 | 2000.6 | 1442 |  |
| 928CE | 23Ceará | Carnaubal | 230340 | 2303402 | 17323.5 | 2786.1 | 2196.1 | 2350.4 | 1849.1 | 1807 |  |
| 929CE | 23Ceará | Cascavel | 230350 | 2303501 | 69384.5 | 11159.1 | 8795.9 | 9413.8 | 7406.1 | 6236 |  |
| 930CE | 23Ceará | Catarina | 230360 | 2303600 | 19858 | 3193.8 | 2517.4 | 2694.3 | 2119.6 | 260 |  |
| 931CE | 23Ceará | Catunda | 230365 | 2303659 | 10225.2 | 1644.5 | 1296.2 | 1387.3 | 1091.4 | 720 |  |
| 932CE | 23Ceará | Caucaia | 230370 | 2303709 | 348654.9 | 56074.2 | 44199 | 47304.2 | 37215.5 | 28725 | 10 |
| 933CE | 23Ceará | Cedro | 230380 | 2303808 | 24958.8 | 4014.1 | 3164 | 3386.3 | 2664.1 | 1753 |  |
| 934CE | 23Ceará | Chaval | 230390 | 2303907 | 12861.1 | 2068.5 | 1630.4 | 1744.9 | 1372.8 | 1299 |  |
| 935CE | 23Ceará | Choró | 230393 | 2303931 | 13220.9 | 2126.3 | 1676 | 1793.8 | 1411.2 | 1717 |  |
| 936CE | 23Ceará | Chorozinho | 230395 | 2303956 | 19236.1 | 3093.7 | 2438.6 | 2609.9 | 2053.3 | 1495 |  |
| 937CE | 23Ceará | Coreaú | 230400 | 2304004 | 22712.7 | 3652.9 | 2879.3 | 3081.6 | 2424.4 | 2376 |  |

3

2

1

1

1

1

2

3

1

1

3

5

1

2

2

2

2

| 938CE | 23Ceará | Crateús | 230410 | 2304103 | 74026.7 | 11905.7 | 9384.4 | 10043.7 | 7901.6 | 2650 |  |
| --- | --- | --- | --- | --- | --- | --- | --- | --- | --- | --- | --- |
| 939CE | 23Ceará | Crato | 230420 | 2304202 | 127479.6 | 20502.5 | 16160.6 | 17295.9 | 13607.2 | 15275 |  |
| 940CE | 23Ceará | Croatá | 230423 | 2304236 | 17619.3 | 2833.7 | 2233.6 | 2390.5 | 1880.7 | 1150 |  |
| 941CE | 23Ceará | Cruz | 230425 | 2304251 | 23536.1 | 3785.3 | 2983.7 | 3193.3 | 2512.3 | 2122 |  |
| 942CE | 23Ceará | Deputado Irapuan  Pinheiro | 230426 | 2304269 | 9386.8 | 1509.7 | 1190 | 1273.6 | 1002 | 764 |  |
| 943CE | 23Ceará | Ererê | 230427 | 2304277 | 7056.7 | 1134.9 | 894.6 | 957.4 | 753.2 | 718 |  |
| 944CE | 23Ceará | Eusébio | 230428 | 2304285 | 50302.3 | 8090.1 | 6376.8 | 6824.8 | 5369.3 | 6004 |  |
| 945CE | 23Ceará | Farias Brito | 230430 | 2304301 | 18945.1 | 3046.9 | 2401.7 | 2570.4 | 2022.2 | 2311 |  |
| 946CE | 23Ceará | Forquilha | 230435 | 2304350 | 23239 | 3737.5 | 2946 | 3153 | 2480.5 | 1912 |  |
| 947CE | 23Ceará | Fortaleza | 230440 | 2304400 | 2569364.5 | 413230.9 | 325718.3 | 348601.6 | 274254.8 | 348233 | 112 |
| 948CE | 23Ceará | Fortim | 230445 | 2304459 | 15763.7 | 2535.3 | 1998.4 | 2138.8 | 1682.6 | 1744 |  |
| 949CE | 23Ceará | Frecheirinha | 230450 | 2304509 | 13475.9 | 2167.3 | 1708.3 | 1828.4 | 1438.4 | 898 |  |
| 950CE | 23Ceará | General Sampaio | 230460 | 2304608 | 6733.7 | 1083 | 853.6 | 913.6 | 718.8 | 655 |  |
| 951CE | 23Ceará | Graça | 230465 | 2304657 | 15149.2 | 2436.4 | 1920.5 | 2055.4 | 1617 | 1890 |  |
| 952CE | 23Ceará | Granja | 230470 | 2304707 | 53641.9 | 8627.2 | 6800.2 | 7277.9 | 5725.8 | 3461 |  |
| 953CE | 23Ceará | Granjeiro | 230480 | 2304806 | 4556 | 732.7 | 577.6 | 618.1 | 486.3 | 506 |  |
| 954CE | 23Ceará | Groaíras | 230490 | 2304905 | 10737.2 | 1726.9 | 1361.2 | 1456.8 | 1146.1 | 1719 |  |
| 955CE | 23Ceará | Guaiúba | 230495 | 2304954 | 25492.2 | 4099.9 | 3231.6 | 3458.7 | 2721 | 1234 |  |
| 956CE | 23Ceará | Guaraciaba do  Norte | 230500 | 2305001 | 39002.8 | 6272.8 | 4944.4 | 5291.8 | 4163.2 | 2597 |  |
| 957CE | 23Ceará | Guaramiranga | 230510 | 2305100 | 3958.6 | 636.7 | 501.8 | 537.1 | 422.5 | 1723 |  |
| 958CE | 23Ceará | Hidrolândia | 230520 | 2305209 | 19889.9 | 3198.9 | 2521.4 | 2698.6 | 2123.1 | 2472 |  |
| 959CE | 23Ceará | Horizonte | 230523 | 2305233 | 62043.7 | 9978.5 | 7865.3 | 8417.9 | 6622.6 | 6040 |  |
| 960CE | 23Ceará | Ibaretama | 230526 | 2305266 | 13145.5 | 2114.2 | 1666.5 | 1783.5 | 1403.2 | 1261 |  |
| 961CE | 23Ceará | Ibiapina | 230530 | 2305308 | 24501.9 | 3940.6 | 3106.1 | 3324.3 | 2615.3 | 1982 |  |
| 962CE | 23Ceará | Ibicuitinga | 230533 | 2305332 | 12000.6 | 1930.1 | 1521.3 | 1628.2 | 1280.9 | 2223 |  |
| 963CE | 23Ceará | Icapuí | 230535 | 2305357 | 19251 | 3096.1 | 2440.4 | 2611.9 | 2054.9 | 1727 |  |
| 964CE | 23Ceará | Icó | 230540 | 2305407 | 66898.6 | 10759.3 | 8480.7 | 9076.5 | 7140.8 | 2926 |  |
| 965CE | 23Ceará | Iguatu | 230550 | 2305506 | 100451.5 | 16155.6 | 12734.2 | 13628.9 | 10722.2 | 7781 |  |
| 966CE | 23Ceará | Independência | 230560 | 2305605 | 25892 | 4164.2 | 3282.3 | 3512.9 | 2763.7 | 390 |  |
| 967CE | 23Ceará | Ipaporanga | 230565 | 2305654 | 11472.8 | 1845.2 | 1454.4 | 1556.6 | 1224.6 | 207 |  |
| 968CE | 23Ceará | Ipaumirim | 230570 | 2305704 | 12255.4 | 1971 | 1553.6 | 1662.8 | 1308.1 | 220 |  |
| 969CE | 23Ceará | Ipu | 230580 | 2305803 | 41208.5 | 6627.6 | 5224 | 5591 | 4398.6 | 5137 |  |
| 970CE | 23Ceará | Ipueiras | 230590 | 2305902 | 37992.3 | 6110.3 | 4816.3 | 5154.7 | 4055.3 | 1212 |  |
| 971CE | 23Ceará | Iracema | 230600 | 2306009 | 14016.7 | 2254.3 | 1776.9 | 1901.7 | 1496.1 | 1979 |  |
| 972CE | 23Ceará | Irauçuba | 230610 | 2306108 | 23344.4 | 3754.5 | 2959.4 | 3167.3 | 2491.8 | 1394 |  |
| 973CE | 23Ceará | Itaiçaba | 230620 | 2306207 | 7600.2 | 1222.3 | 963.5 | 1031.2 | 811.2 | 2134 |  |
| 974CE | 23Ceará | Itaitinga | 230625 | 2306256 | 37907.2 | 6096.6 | 4805.5 | 5143.1 | 4046.2 | 1791 |  |
| 975CE | 23Ceará | Itapagé | 230630 | 2306306 | 50678 | 8150.5 | 6424.5 | 6875.8 | 5409.4 | 3248 |  |
| 976CE | 23Ceará | Itapipoca | 230640 | 2306405 | 123507.9 | 19863.8 | 15657.1 | 16757.1 | 13183.3 | 14111 |  |
| 977CE | 23Ceará | Itapiúna | 230650 | 2306504 | 19555.9 | 3145.2 | 2479.1 | 2653.3 | 2087.4 | 3290 |  |
| 978CE | 23Ceará | Itarema | 230655 | 2306553 | 39920.6 | 6420.4 | 5060.7 | 5416.3 | 4261.1 | 2176 |  |
| 979CE | 23Ceará | Itatira | 230660 | 2306603 | 20126.1 | 3236.9 | 2551.4 | 2730.6 | 2148.3 | 877 |  |
| 980CE | 23Ceará | Jaguaretama | 230670 | 2306702 | 17980.7 | 2891.8 | 2279.4 | 2439.6 | 1919.3 | 3003 |  |
| 981CE | 23Ceará | Jaguaribara | 230680 | 2306801 | 10982.6 | 1766.3 | 1392.3 | 1490.1 | 1172.3 | 1502 |  |
| 982CE | 23Ceará | Jaguaribe | 230690 | 2306900 | 34531.5 | 5553.7 | 4377.6 | 4685.1 | 3685.9 | 9049 |  |
| 983CE | 23Ceará | Jaguaruana | 230700 | 2307007 | 33226.4 | 5343.8 | 4212.1 | 4508 | 3546.6 | 6229 |  |
| 984CE | 23Ceará | Jardim | 230710 | 2307106 | 26994.3 | 4341.5 | 3422.1 | 3662.5 | 2881.4 | 2635 |  |
| 985CE | 23Ceará | Jati | 230720 | 2307205 | 7804 | 1255.1 | 989.3 | 1058.8 | 833 | 867 |  |
| 986CE | 23Ceará | Jijoca de  Jericoacoara | 230725 | 2307254 | 18609.6 | 2993 | 2359.1 | 2524.9 | 1986.4 | 1077 |  |
| 987CE | 23Ceará | Juazeiro do Norte | 230730 | 2307304 | 263420.7 | 42366 | 33393.8 | 35739.9 | 28117.6 | 23768 |  |
| 988CE | 23Ceará | Jucás | 230740 | 2307403 | 24367.9 | 3919.1 | 3089.1 | 3306.1 | 2601 | 1643 |  |
| 989CE | 23Ceará | Lavras da  Mangabeira | 230750 | 2307502 | 31325.7 | 5038.1 | 3971.2 | 4250.2 | 3343.7 | 1071 |  |
| 990CE | 23Ceará | Limoeiro do Norte | 230760 | 2307601 | 57890.7 | 9310.6 | 7338.8 | 7854.4 | 6179.3 | 10579 |  |
| 991CE | 23Ceará | Madalena | 230763 | 2307635 | 19167.8 | 3082.8 | 2429.9 | 2600.6 | 2046 | 714 |  |
| 992CE | 23Ceará | Maracanaú | 230765 | 2307650 | 219490.9 | 35300.7 | 27824.9 | 29779.7 | 23428.5 | 51457 | 19 |
| 993CE | 23Ceará | Maranguape | 230770 | 2307700 | 121994.6 | 19620.4 | 15465.3 | 16551.8 | 13021.7 | 5801 |  |
| 994CE | 23Ceará | Marco | 230780 | 2307809 | 26193.7 | 4212.7 | 3320.6 | 3553.9 | 2795.9 | 1512 |  |
| 995CE | 23Ceará | Martinópole | 230790 | 2307908 | 10784.2 | 1734.4 | 1367.1 | 1463.2 | 1151.1 | 659 |  |
| 996CE | 23Ceará | Massapê | 230800 | 2308005 | 37174.4 | 5978.8 | 4712.6 | 5043.7 | 3968 | 2281 |  |
| 997CE | 23Ceará | Mauriti | 230810 | 2308104 | 45895.2 | 7381.3 | 5818.1 | 6226.9 | 4898.9 | 3931 |  |
| 998CE | 23Ceará | Meruoca | 230820 | 2308203 | 14504.5 | 2332.8 | 1838.7 | 1967.9 | 1548.2 | 2030 |  |
| 999CE | 23Ceará | Milagres | 230830 | 2308302 | 28254 | 4544.1 | 3581.8 | 3833.4 | 3015.8 | 2734 |  |
| 1000CE | 23Ceará | Milhã | 230835 | 2308351 | 13147.1 | 2114.4 | 1666.7 | 1783.7 | 1403.3 | 2346 |  |
| 1001CE | 23Ceará | Miraíma | 230837 | 2308377 | 13332.4 | 2144.2 | 1690.1 | 1808.9 | 1423.1 | 810 |  |
| 1002CE | 23Ceará | Missão Velha | 230840 | 2308401 | 35149.2 | 5653 | 4455.9 | 4768.9 | 3751.8 | 2092 |  |
| 1003CE | 23Ceará | Mombaça | 230850 | 2308500 | 43431.2 | 6985 | 5505.8 | 5892.6 | 4635.9 | 2699 |  |
| 1004CE | 23Ceará | Monsenhor  Tabosa | 230860 | 2308609 | 16965.6 | 2728.6 | 2150.7 | 2301.8 | 1810.9 | 623 |  |
| 1005CE | 23Ceará | Morada Nova | 230870 | 2308708 | 61917.4 | 9958.2 | 7849.3 | 8400.7 | 6609.1 | 12157 |  |
| 1006CE | 23Ceará | Moraújo | 230880 | 2308807 | 8444.2 | 1358.1 | 1070.5 | 1145.7 | 901.3 | 790 |  |
| 1007CE | 23Ceará | Morrinhos | 230890 | 2308906 | 21711.2 | 3491.8 | 2752.3 | 2945.7 | 2317.5 | 1205 |  |
| 1008CE | 23Ceará | Mucambo | 230900 | 2309003 | 14317.6 | 2302.7 | 1815 | 1942.6 | 1528.3 | 1683 |  |
| 1009CE | 23Ceará | Mulungu | 230910 | 2309102 | 12135.9 | 1951.8 | 1538.5 | 1646.6 | 1295.4 | 3458 |  |
| 1010CE | 23Ceará | Nova Olinda | 230920 | 2309201 | 15023 | 2416.1 | 1904.5 | 2038.3 | 1603.6 | 904 |  |
| 1011CE | 23Ceará | Nova Russas | 230930 | 2309300 | 31718.8 | 5101.3 | 4021 | 4303.5 | 3385.7 | 795 |  |
| 1012CE | 23Ceará | Novo Oriente | 230940 | 2309409 | 28091.2 | 4517.9 | 3561.1 | 3811.3 | 2998.5 | 689 |  |
| 1013CE | 23Ceará | Ocara | 230945 | 2309458 | 24941.9 | 4011.4 | 3161.9 | 3384 | 2662.3 | 3812 |  |
| 1014CE | 23Ceará | Orós | 230950 | 2309508 | 21389.9 | 3440.1 | 2711.6 | 2902.1 | 2283.2 | 1390 |  |
| 1015CE | 23Ceará | Pacajus | 230960 | 2309607 | 67673.3 | 10883.9 | 8578.9 | 9181.7 | 7223.5 | 6432 |  |
| 1016CE | 23Ceará | Pacatuba | 230970 | 2309706 | 78980.8 | 12702.5 | 10012.4 | 10715.8 | 8430.4 | 3672 |  |
| 1017CE | 23Ceará | Pacoti | 230980 | 2309805 | 11879.1 | 1910.5 | 1505.9 | 1611.7 | 1268 | 2807 |  |
| 1018CE | 23Ceará | Pacujá | 230990 | 2309904 | 6165.1 | 991.5 | 781.5 | 836.5 | 658.1 | 1092 |  |
| 1019CE | 23Ceará | Palhano | 231000 | 2310001 | 9152.9 | 1472.1 | 1160.3 | 1241.8 | 977 | 2171 |  |
| 1020CE | 23Ceará | Palmácia | 231010 | 2310100 | 12748.9 | 2050.4 | 1616.2 | 1729.7 | 1360.8 | 797 |  |
| 1021CE | 23Ceará | Paracuru | 231020 | 2310209 | 33209.6 | 5341.1 | 4210 | 4505.8 | 3544.8 | 3630 |  |
| 1022CE | 23Ceará | Paraipaba | 231025 | 2310258 | 31648.5 | 5090 | 4012.1 | 4293.9 | 3378.2 | 2630 |  |
| 1023CE | 23Ceará | Parambu | 231030 | 2310308 | 31310.2 | 5035.6 | 3969.2 | 4248 | 3342.1 | 3879 |  |
| 1024CE | 23Ceará | Paramoti | 231040 | 2310407 | 11563.3 | 1859.7 | 1465.9 | 1568.9 | 1234.3 | 620 |  |
| 1025CE | 23Ceará | Pedra Branca | 231050 | 2310506 | 42607.1 | 6852.5 | 5401.3 | 5780.8 | 4547.9 | 5187 |  |
| 1026CE | 23Ceará | Penaforte | 231060 | 2310605 | 8718.5 | 1402.2 | 1105.2 | 1182.9 | 930.6 | 1071 |  |
| 1027CE | 23Ceará | Pentecoste | 231070 | 2310704 | 36574.6 | 5882.3 | 4636.6 | 4962.3 | 3904 | 3195 |  |
| 1028CE | 23Ceará | Pereiro | 231080 | 2310803 | 16055 | 2582.1 | 2035.3 | 2178.3 | 1713.7 | 2258 |  |
| 1029CE | 23Ceará | Pindoretama | 231085 | 2310852 | 19920.3 | 3203.8 | 2525.3 | 2702.7 | 2126.3 | 1668 |  |
| 1030CE | 23Ceará | Piquet Carneiro | 231090 | 2310902 | 16299.7 | 2621.5 | 2066.3 | 2211.5 | 1739.8 | 1015 |  |
| 1031CE | 23Ceará | Pires Ferreira | 231095 | 2310951 | 10603.7 | 1705.4 | 1344.2 | 1438.7 | 1131.8 | 1009 |  |
| 1032CE | 23Ceará | Poranga | 231100 | 2311009 | 12185.1 | 1959.7 | 1544.7 | 1653.2 | 1300.6 | 295 |  |
| 1033CE | 23Ceará | Porteiras | 231110 | 2311108 | 15015.2 | 2414.9 | 1903.5 | 2037.2 | 1602.7 | 1433 |  |
| 1034CE | 23Ceará | Potengi | 231120 | 2311207 | 10705.5 | 1721.8 | 1357.1 | 1452.5 | 1142.7 | 1065 |  |
| 1035CE | 23Ceará | Potiretama | 231123 | 2311231 | 6286.7 | 1011.1 | 797 | 853 | 671 | 886 |  |
| 1036CE | 23Ceará | Quiterianópolis | 231126 | 2311264 | 20563.6 | 3307.2 | 2606.8 | 2790 | 2195 | 517 |  |
| 1037CE | 23Ceará | Quixadá | 231130 | 2311306 | 84577.1 | 13602.5 | 10721.8 | 11475.1 | 9027.8 | 14256 |  |
| 1038CE | 23Ceará | Quixelô | 231135 | 2311355 | 15078.4 | 2425.1 | 1911.5 | 2045.8 | 1609.5 | 1295 |  |
| 1039CE | 23Ceará | Quixeramobim | 231140 | 2311405 | 76444.1 | 12294.5 | 9690.8 | 10371.6 | 8159.7 | 9476 |  |
| 1040CE | 23Ceará | Quixeré | 231150 | 2311504 | 21181.4 | 3406.6 | 2685.2 | 2873.8 | 2260.9 | 2992 |  |
| 1041CE | 23Ceará | Redenção | 231160 | 2311603 | 27264.1 | 4384.9 | 3456.3 | 3699.1 | 2910.2 | 1990 |  |
| 1042CE | 23Ceará | Reriutaba | 231170 | 2311702 | 19048.4 | 3063.6 | 2414.8 | 2584.4 | 2033.2 | 2055 |  |
| 1043CE | 23Ceará | Russas | 231180 | 2311801 | 74236.1 | 11939.4 | 9410.9 | 10072.1 | 7924 | 19334 |  |
| 1044CE | 23Ceará | Saboeiro | 231190 | 2311900 | 15755 | 2533.9 | 1997.3 | 2137.6 | 1681.7 | 1095 |  |

6

2

1

2

1

1

2

1

1

5

1

1

2

2

1

9

3

1

1

1

5

1

1

2

1

1

1

1

1

2

1

5

4

8

1045CE	23Ceará	Salitre	231195	2311959	16048.2	2581	2034.4	2177.4	1713	766

Santana do

1046CE	23Ceará	Acaraú	231200	2312007	31329.5	5038.7	3971.6	4250.7	3344.1	2661

1047CE	23Ceará	Santana do Cariri	231210	2312106	17424.4	2802.4	2208.9	2364.1	1859.9	1183

1048CE	23Ceará	Santa Quitéria	231220	2312205	43257.1	6957	5483.7	5869	4617.3	2726

1049CE	23Ceará	São Benedito	231230	2312304	45916.5	7384.8	5820.8	6229.8	4901.1	3593

São Gonçalo do

1050CE	23Ceará	Amarante	231240	2312403	46687.8	7508.8	5918.6	6334.4	4983.5	6331

São João do

1051CE	23Ceará	Jaguaribe	231250	2312502	7748.2	1246.1	982.2	1051.2	827	1613

1052CE	23Ceará	São Luís do Curu	231260	2312601	12691.5	2041.2	1608.9	1721.9	1354.7	1075

1053CE	23Ceará	Senador Pompeu	231270	2312700	26418.1	4248.8	3349	3584.3	2819.9	3205

1054CE	23Ceará	Senador Sá	231280	2312809	7283.9	1171.5	923.4	988.3	777.5	397

1055CE	23Ceará	Sobral	231290	2312908	199605	32102.5	25303.9	27081.6	21305.9	39291	15

1056CE	23Ceará	Solonópole	231300	2313005	18022.9	2898.6	2284.8	2445.3	1923.8	2832

1057CE	23Ceará	Tabuleiro do Norte	231310	2313104	30077.4	4837.3	3812.9	4080.8	3210.5	3434

1058CE	23Ceará	Tamboril	231320	2313203	25624.5	4121.2	3248.4	3476.6	2735.2	1216

1059CE	23Ceará	Tarrafas	231325	2313252	8867.8	1426.2	1124.2	1203.1	946.6	381

1060CE	23Ceará	Tauá	231330	2313302	57387.3	9229.6	7275	7786.1	6125.5	5978

1061CE	23Ceará	Tejuçuoca	231335	2313351	18224.1	2931	2310.3	2472.6	1945.2	1146

1062CE	23Ceará	Tianguá	231340	2313401	72743.5	11699.3	9221.7	9869.6	7764.7	7257

1063CE	23Ceará	Trairi	231350	2313500	53929.4	8673.5	6836.6	7316.9	5756.4	3120

1064CE	23Ceará	Tururu	231355	2313559	15410.7	2478.5	1953.6	2090.9	1644.9	929

1065CE	23Ceará	Ubajara	231360	2313609	33465.4	5382.2	4242.4	4540.5	3572.1	1990

1066CE	23Ceará	Umari	231370	2313708	7644.8	1229.5	969.1	1037.2	816	373

1067CE	23Ceará	Umirim	231375	2313757	19396.3	3119.5	2458.9	2631.6	2070.4	1246

1068CE	23Ceará	Uruburetama	231380	2313807	20964.5	3371.7	2657.7	2844.4	2237.8	1734

1069CE	23Ceará	Uruoca	231390	2313906	13415.2	2157.6	1700.6	1820.1	1431.9	838

1070CE	23Ceará	Varjota	231395	2313955	18046.4	2902.4	2287.7	2448.5	1926.3	2191

1071CE	23Ceará	Várzea Alegre	231400	2314003	39777.7	6397.4	5042.6	5396.9	4245.9	6094

1072CE	23Ceará	Viçosa do Ceará	231410	2314102	58276.9	9372.7	7387.8	7906.8	6220.5	3542

Rio Grande

1073RN	24do Norte	Acari	240010	2400109	11207.7	1854.3	1478.1	1481.4	1215.6	2067

Rio Grande

1074RN	24do Norte	Açu	240020	2400208	56295.6	9314.1	7424.3	7441	6105.7	7697

Rio Grande

1075RN	24do Norte	Afonso Bezerra	240030	2400307	11061.1	1830.1	1458.7	1462	1199.7	1315

Rio Grande

1076RN	24do Norte	Água Nova	240040	2400406	3153.5	521.7	415.9	416.8	342	432

Rio Grande

1077RN	24do Norte	Alexandria	240050	2400505	13690	2265	1805.4	1809.5	1484.8	3854

Rio Grande

1078RN	24do Norte	Almino Afonso	240060	2400604	4853.2	803	640	641.5	526.4	783

Rio Grande

1079RN	24do Norte	Alto do Rodrigues	240070	2400703	13595.2	2249.3	1792.9	1797	1474.5	2084

Rio Grande

1080RN	24do Norte	Angicos	240080	2400802	11760.3	1945.7	1550.9	1554.5	1275.5	1379

Rio Grande

1081RN	24do Norte	Antônio Martins	240090	2400901	7106.1	1175.7	937.2	939.3	770.7	1110

Rio Grande

1082RN	24do Norte	Apodi	240100	2401008	35702	5906.9	4708.4	4719	3872.2	5491

Rio Grande

1083RN	24do Norte	Areia Branca	240110	2401107	26743.2	4424.7	3526.9	3534.9	2900.5	2583

Rio Grande

1084RN	24do Norte	Arês	240120	2401206	13782.9	2280.4	1817.7	1821.8	1494.9	1381

Rio Grande

1085RN	24do Norte	Augusto Severo	240130	2401305	9579.5	1584.9	1263.3	1266.2	1039	722

Rio Grande

1086RN	24do Norte	Baía Formosa	240140	2401404	9028.2	1493.7	1190.6	1193.3	979.2	777

1

2

1

1

2

2

1

2

1

3

1

2

7

1

1

2

1

6

Rio Grande

1087RN	24do Norte	Baraúna	240145	2401453	26624.9	4405.1	3511.3	3519.2	2887.7	1883

Rio Grande

1088RN	24do Norte	Barcelona	240150	2401503	4017	664.6	529.8	531	435.7	927

Rio Grande

1089RN	24do Norte	Bento Fernandes	240160	2401602	5368.3	888.2	708	709.6	582.2	586

Rio Grande

1090RN	24do Norte	Bodó	240165	2401651	2346.3	388.2	309.4	310.1	254.5	138

Rio Grande

1091RN	24do Norte	Bom Jesus	240170	2401701	9943	1645.1	1311.3	1314.2	1078.4	1228

Rio Grande

1092RN	24do Norte	Brejinho	240180	2401800	12285.8	2032.7	1620.3	1623.9	1332.5	1797

Rio Grande

1093RN	24do Norte	Caiçara do Norte	240185	2401859	6381.6	1055.8	841.6	843.5	692.1	527

Rio Grande Caiçara do Rio do

1094RN	24do Norte	Vento	240190	2401909	3539.5	585.6	466.8	467.8	383.9	610

Rio Grande

1095RN	24do Norte	Caicó	240200	2402006	66116.6	10939	8719.5	8739.2	7170.9	20494

Rio Grande

1096RN	24do Norte	Campo Redondo	240210	2402105	10871.9	1798.8	1433.8	1437	1179.1	847

Rio Grande

1097RN	24do Norte	Canguaretama	240220	2402204	32998.6	5459.6	4351.9	4361.7	3579	3440

Rio Grande

1098RN	24do Norte	Caraúbas	240230	2402303	20391	3373.7	2689.2	2695.2	2211.6	3290

Rio Grande Carnaúba dos

1099RN	24do Norte	Dantas	240240	2402402	7900	1307.1	1041.9	1044.2	856.8	1419

Rio Grande

1100RN	24do Norte	Carnaubais	240250	2402501	10424.1	1724.7	1374.7	1377.8	1130.6	455

Rio Grande

1101RN	24do Norte	Ceará-Mirim	240260	2402600	71664.9	11857	9451.2	9472.5	7772.6	7622

Rio Grande

1102RN	24do Norte	Cerro Corá	240270	2402709	11171.3	1848.3	1473.3	1476.6	1211.6	1558

Rio Grande

1103RN	24do Norte	Coronel Ezequiel	240280	2402808	5516.2	912.7	727.5	729.1	598.3	633

Rio Grande Coronel João

1104RN	24do Norte	Pessoa	240290	2402907	4896.8	810.2	645.8	647.2	531.1	617

Rio Grande

1105RN	24do Norte	Cruzeta	240300	2403004	8069.6	1335.1	1064.2	1066.6	875.2	1183

Rio Grande

1106RN	24do Norte	Currais Novos	240310	2403103	44224.4	7316.9	5832.3	5845.5	4796.5	3106

Rio Grande

1107RN	24do Norte	Doutor Severiano	240320	2403202	7000.9	1158.3	923.3	925.4	759.3	871

Rio Grande

1108RN	24do Norte	Parnamirim	240325	2403251	235345.5	38937.9	31037.4	31107.5	25525.1	18864

Rio Grande

1109RN	24do Norte	Encanto	240330	2403301	5499.6	909.9	725.3	726.9	596.5	549

Rio Grande

1110RN	24do Norte	Equador	240340	2403400	6001.6	993	791.5	793.3	650.9	809

Rio Grande

1111RN	24do Norte	Espírito Santo	240350	2403509	10603.8	1754.4	1398.4	1401.6	1150.1	885

Rio Grande

1112RN	24do Norte	Extremoz	240360	2403608	26922.4	4454.3	3550.5	3558.5	2920	2193

Rio Grande

1113RN	24do Norte	Felipe Guerra	240370	2403707	5927	980.6	781.7	783.4	642.8	844

Rio Grande

1114RN	24do Norte	Fernando Pedroza	240375	2403756	2988.5	494.4	394.1	395	324.1	736

Rio Grande

1115RN	24do Norte	Florânia	240380	2403806	9138	1511.9	1205.1	1207.8	991.1	1562

Rio Grande

1116RN	24do Norte	Francisco Dantas	240390	2403905	2879.5	476.4	379.7	380.6	312.3	544

Rio Grande

1117RN	24do Norte	Frutuoso Gomes	240400	2404002	4192.9	693.7	553	554.2	454.8	525

Rio Grande

1118RN	24do Norte	Galinhos	240410	2404101	2508.8	415.1	330.9	331.6	272.1	300

Rio Grande

1119RN	24do Norte	Goianinha	240420	2404200	24725.9	4090.9	3260.9	3268.2	2681.7	3903

Rio Grande Governador Dix-

1120RN	24do Norte	Sept Rosado	240430	2404309	12852.7	2126.5	1695	1698.8	1394	818

Rio Grande

1121RN	24do Norte	Grossos	240440	2404408	10009.8	1656.1	1320.1	1323.1	1085.6	405

Rio Grande

1122RN	24do Norte	Guamaré	240450	2404507	14231.1	2354.5	1876.8	1881	1543.5	1956

Rio Grande

1123RN	24do Norte	Ielmo Marinho	240460	2404606	13132.6	2172.8	1731.9	1735.8	1424.3	1572

Rio Grande

1124RN	24do Norte	Ipanguaçu	240470	2404705	14856.9	2458.1	1959.3	1963.8	1611.4	1873

Rio Grande

1125RN	24do Norte	Ipueira	240480	2404804	2184.4	361.4	288.1	288.7	236.9	446

Rio Grande

1126RN	24do Norte	Itajá	240485	2404853	7327.7	1212.4	966.4	968.6	794.7	628

Rio Grande

1127RN	24do Norte	Itaú	240490	2404903	5787.9	957.6	763.3	765	627.7	622

Rio Grande

1128RN	24do Norte	Jaçanã	240500	2405009	8639.2	1429.4	1139.3	1141.9	937	1008

Rio Grande

1129RN	24do Norte	Jandaíra	240510	2405108	6892.1	1140.3	908.9	911	747.5	258

Rio Grande

1130RN	24do Norte	Janduís	240520	2405207	5363.1	887.3	707.3	708.9	581.7	778

Rio Grande

1131RN	24do Norte	Januário Cicco	240530	2405306	9698.5	1604.6	1279	1281.9	1051.9	570

Rio Grande

1132RN	24do Norte	Japi	240540	2405405	5339.1	883.4	704.1	705.7	579.1	357

Rio Grande

1133RN	24do Norte	Jardim de Angicos	240550	2405504	2638.3	436.5	347.9	348.7	286.1	306

Rio Grande Jardim de

1134RN	24do Norte	Piranhas	240560	2405603	14344.6	2373.3	1891.8	1896	1555.8	2017

Rio Grande

1135RN	24do Norte	Jardim do Seridó	240570	2405702	12391.1	2050.1	1634.1	1637.8	1343.9	2618

Rio Grande

1136RN	24do Norte	João Câmara	240580	2405801	33995.4	5624.5	4483.3	4493.4	3687.1	6260

Rio Grande

1137RN	24do Norte	João Dias	240590	2405900	2656.8	439.6	350.4	351.2	288.2	459

Rio Grande

1138RN	24do Norte	José da Penha	240600	2406007	5975	988.6	788	789.8	648	1131

Rio Grande

1139RN	24do Norte	Jucurutu	240610	2406106	18197.6	3010.8	2399.9	2405.3	1973.7	3643

Rio Grande

1140RN	24do Norte	Jundiá	240615	2406155	3785	626.2	499.2	500.3	410.5	627

Rio Grande

1141RN	24do Norte	Lagoa d'Anta	240620	2406205	6577	1088.2	867.4	869.3	713.3	683

Rio Grande

1142RN	24do Norte	Lagoa de Pedras	240630	2406304	7353.3	1216.6	969.8	971.9	797.5	1177

Rio Grande

1143RN	24do Norte	Lagoa de Velhos	240640	2406403	2729.6	451.6	360	360.8	296	314

Rio Grande

1144RN	24do Norte	Lagoa Nova	240650	2406502	14982.9	2478.9	1975.9	1980.4	1625	1741

Rio Grande

1145RN	24do Norte	Lagoa Salgada	240660	2406601	8000.4	1323.7	1055.1	1057.5	867.7	967

Rio Grande

1146RN	24do Norte	Lajes	240670	2406700	10959.5	1813.2	1445.3	1448.6	1188.6	2333

Rio Grande

1147RN	24do Norte	Lajes Pintadas	240680	2406809	4738.5	784	624.9	626.3	513.9	633

Rio Grande

1148RN	24do Norte	Lucrécia	240690	2406908	3861.6	638.9	509.3	510.4	418.8	1068

1

2

1

4

1

1

1

3

Rio Grande

1149RN	24do Norte	Luís Gomes	240700	2407005	9977.5	1650.8	1315.8	1318.8	1082.1	1013

Rio Grande

1150RN	24do Norte	Macaíba	240710	2407104	76187	12605.1	10047.5	10070.2	8263.1	11105

Rio Grande

1151RN	24do Norte	Macau	240720	2407203	30755.7	5088.5	4056.1	4065.2	3335.7	1908

Rio Grande

1152RN	24do Norte	Major Sales	240725	2407252	3826.4	633.1	504.6	505.8	415	445

Rio Grande

1153RN	24do Norte	Marcelino Vieira	240730	2407302	8397	1389.3	1107.4	1109.9	910.7	920

Rio Grande

1154RN	24do Norte	Martins	240740	2407401	8570.9	1418.1	1130.3	1132.9	929.6	474

Rio Grande

1155RN	24do Norte	Maxaranguape	240750	2407500	11557.5	1912.2	1524.2	1527.6	1253.5	1778

Rio Grande

1156RN	24do Norte	Messias Targino	240760	2407609	4448.4	736	586.7	588	482.5	463

Rio Grande

1157RN	24do Norte	Montanhas	240770	2407708	11452.8	1894.9	1510.4	1513.8	1242.1	1440

Rio Grande

1158RN	24do Norte	Monte Alegre	240780	2407807	21791.5	3605.4	2873.9	2880.4	2363.5	3312

Rio Grande Monte das

1159RN	24do Norte	Gameleiras	240790	2407906	2204.8	364.8	290.8	291.4	239.1	290

Rio Grande

1160RN	24do Norte	Mossoró	240800	2408003	282169.1	46684.9	37212.5	37296.5	30603.5	50747	20

Rio Grande

1161RN	24do Norte	Natal	240810	2408102	854263.9	141338	112660.3	112914.9	92651.8	157697	53

Rio Grande

1162RN	24do Norte	Nísia Floresta	240820	2408201	26027.5	4306.2	3432.5	3440.3	2822.9	4108

Rio Grande

1163RN	24do Norte	Nova Cruz	240830	2408300	36837.9	6094.8	4858.2	4869.2	3995.4	2789

Rio Grande Olho-d'Água do

1164RN	24do Norte	Borges	240840	2408409	4322.6	715.2	570.1	571.4	468.8	1054

Rio Grande

1165RN	24do Norte	Ouro Branco	240850	2408508	4808.4	795.5	634.1	635.6	521.5	684

Rio Grande

1166RN	24do Norte	Paraná	240860	2408607	4152.1	687	547.6	548.8	450.3	507

Rio Grande

1167RN	24do Norte	Paraú	240870	2408706	3853.5	637.6	508.2	509.3	417.9	479

Rio Grande

1168RN	24do Norte	Parazinho	240880	2408805	5100.4	843.9	672.6	674.2	553.2	705

Rio Grande

1169RN	24do Norte	Parelhas	240890	2408904	21158.7	3500.7	2790.4	2796.7	2294.8	1916

Rio Grande

1170RN	24do Norte	Rio do Fogo	240895	2408953	10578.7	1750.2	1395.1	1398.3	1147.3	1275

Rio Grande

1171RN	24do Norte	Passa e Fica	240910	2409100	12353.1	2043.8	1629.1	1632.8	1339.8	1343

Rio Grande

1172RN	24do Norte	Passagem	240920	2409209	3026.7	500.8	399.2	400.1	328.3	367

Rio Grande

1173RN	24do Norte	Patu	240930	2409308	12505.1	2069	1649.2	1652.9	1356.3	1375

Rio Grande

1174RN	24do Norte	Santa Maria	240933	2409332	5223.8	864.3	688.9	690.5	566.6	690

Rio Grande

1175RN	24do Norte	Pau dos Ferros	240940	2409407	29423.1	4868.1	3880.3	3889.1	3191.2	7402

Rio Grande

1176RN	24do Norte	Pedra Grande	240950	2409506	3411.2	564.4	449.9	450.9	370	479

Rio Grande

1177RN	24do Norte	Pedra Preta	240960	2409605	2549.2	421.8	336.2	336.9	276.5	292

Rio Grande

1178RN	24do Norte	Pedro Avelino	240970	2409704	7012.2	1160.2	924.8	926.9	760.5	658

Rio Grande

1179RN	24do Norte	Pedro Velho	240980	2409803	14626.5	2420	1928.9	1933.3	1586.4	2141

Rio Grande

1180RN	24do Norte	Pendências	240990	2409902	14460.6	2392.5	1907.1	1911.4	1568.4	1160

Rio Grande

1181RN	24do Norte	Pilões	241000	2410009	3690.9	610.7	486.8	487.9	400.3	563

Rio Grande

1182RN	24do Norte	Poço Branco	241010	2410108	14861.5	2458.8	1959.9	1964.4	1611.9	1932

Rio Grande

1183RN	24do Norte	Portalegre	241020	2410207	7683.4	1271.2	1013.3	1015.6	833.3	862

Rio Grande

1184RN	24do Norte	Porto do Mangue	241025	2410256	5995.7	992	790.7	792.5	650.3	576

Rio Grande Presidente

1185RN	24do Norte	Juscelino	241030	2410306	9641.9	1595.3	1271.6	1274.4	1045.7	912

Rio Grande

1186RN	24do Norte	Pureza	241040	2410405	9138.1	1511.9	1205.1	1207.9	991.1	936

Rio Grande

1187RN	24do Norte	Rafael Fernandes	241050	2410504	4953.8	819.6	653.3	654.8	537.3	683

Rio Grande

1188RN	24do Norte	Rafael Godeiro	241060	2410603	3167.3	524	417.7	418.6	343.5	335

Rio Grande

1189RN	24do Norte	Riacho da Cruz	241070	2410702	3414.9	565	450.4	451.4	370.4	196

Rio Grande Riacho de

1190RN	24do Norte	Santana	241080	2410801	4226.4	699.3	557.4	558.6	458.4	608

Rio Grande

1191RN	24do Norte	Riachuelo	241090	2410900	7696.2	1273.3	1015	1017.3	834.7	890

Rio Grande Rodolfo

1192RN	24do Norte	Fernandes	241100	2411007	4491.7	743.2	592.4	593.7	487.2	655

Rio Grande

1193RN	24do Norte	Tibau	241105	2411056	3944.2	652.6	520.2	521.3	427.8	164

Rio Grande

1194RN	24do Norte	Ruy Barbosa	241110	2411106	3636.5	601.7	479.6	480.7	394.4	465

Rio Grande

1195RN	24do Norte	Santa Cruz	241120	2411205	38201.7	6320.5	5038	5049.4	4143.3	3738

Rio Grande

1196RN	24do Norte	Santana do Matos	241140	2411403	13422.1	2220.7	1770.1	1774.1	1455.7	1202

Rio Grande

1197RN	24do Norte	Santana do Seridó	241142	2411429	2633.4	435.7	347.3	348.1	285.6	831

Rio Grande

1198RN	24do Norte	Santo Antônio	241150	2411502	23455.6	3880.7	3093.3	3100.3	2544	2484

Rio Grande São Bento do

1199RN	24do Norte	Norte	241160	2411601	2888.7	477.9	381	381.8	313.3	232

Rio Grande São Bento do

1200RN	24do Norte	Trairí	241170	2411700	4229.8	699.8	557.8	559.1	458.8	381

Rio Grande

1201RN	24do Norte	São Fernando	241180	2411809	3533.4	584.6	466	467	383.2	778

Rio Grande São Francisco do

1202RN	24do Norte	Oeste	241190	2411908	4099.8	678.3	540.7	541.9	444.7	533

Rio Grande São Gonçalo do

1203RN	24do Norte	Amarante	241200	2412005	96204.3	15917	12687.4	12716.1	10434.1	7834

Rio Grande São João do

1204RN	24do Norte	Sabugi	241210	2412104	6127.7	1013.8	808.1	809.9	664.6	928

Rio Grande São José de

1205RN	24do Norte	Mipibu	241220	2412203	42387.7	7013	5590.1	5602.7	4597.3	6896

Rio Grande São José do

1206RN	24do Norte	Campestre	241230	2412302	12750.6	2109.6	1681.5	1685.3	1382.9	785

Rio Grande São José do

1207RN	24do Norte	Seridó	241240	2412401	4486.4	742.3	591.7	593	486.6	1063

Rio Grande

1208RN	24do Norte	São Miguel	241250	2412500	22971.2	3800.6	3029.4	3036.3	2491.4	3099

Rio Grande São Miguel do

1209RN	24do Norte	Gostoso	241255	2412559	9319.3	1541.9	1229	1231.8	1010.8	1021

Rio Grande São Paulo do

1210RN	24do Norte	Potengi	241260	2412609	16917.7	2799	2231.1	2236.1	1834.9	3820

1

2

2

1

1

Rio Grande

1211RN	24do Norte	São Pedro	241270	2412708	6165	1020	813	814.9	668.6	727

Rio Grande

1212RN	24do Norte	São Rafael	241280	2412807	8246.2	1364.3	1087.5	1090	894.4	1438

Rio Grande

1213RN	24do Norte	São Tomé	241290	2412906	11062.5	1830.3	1458.9	1462.2	1199.8	1573

Rio Grande

1214RN	24do Norte	São Vicente	241300	2413003	6299.1	1042.2	830.7	832.6	683.2	1233

Rio Grande Senador Elói de

1215RN	24do Norte	Souza	241310	2413102	5960.7	986.2	786.1	787.9	646.5	987

Rio Grande Senador Georgino

1216RN	24do Norte	Avelino	241320	2413201	4235.1	700.7	558.5	559.8	459.3	936

Rio Grande Serra de São

1217RN	24do Norte	Bento	241330	2413300	5815.2	962.1	766.9	768.6	630.7	660

Rio Grande

1218RN	24do Norte	Serra do Mel	241335	2413359	11257.1	1862.5	1484.6	1487.9	1220.9	1159

Rio Grande Serra Negra do

1219RN	24do Norte	Norte	241340	2413409	8015.1	1326.1	1057	1059.4	869.3	2142

Rio Grande

1220RN	24do Norte	Serrinha	241350	2413508	6469.8	1070.4	853.2	855.2	701.7	1159

Rio Grande Serrinha dos

1221RN	24do Norte	Pintos	241355	2413557	4724.4	781.7	623.1	624.5	512.4	564

Rio Grande

1222RN	24do Norte	Severiano Melo	241360	2413607	4215.2	697.4	555.9	557.2	457.2	1123

Rio Grande

1223RN	24do Norte	Sítio Novo	241370	2413706	5335.3	882.7	703.6	705.2	578.7	463

Rio Grande

1224RN	24do Norte	Taboleiro Grande	241380	2413805	2471.6	408.9	326	326.7	268.1	398

Rio Grande

1225RN	24do Norte	Taipu	241390	2413904	12194.5	2017.6	1608.2	1611.8	1322.6	1653

Rio Grande

1226RN	24do Norte	Tangará	241400	2414001	15192.6	2513.6	2003.6	2008.1	1647.8	1422

Rio Grande

1227RN	24do Norte	Tenente Ananias	241410	2414100	10459.7	1730.6	1379.4	1382.5	1134.4	1088

Rio Grande Tenente

1228RN	24do Norte	Laurentino Cruz	241415	2414159	5755.5	952.2	759	760.8	624.2	780

Rio Grande

1229RN	24do Norte	Tibau do Sul	241420	2414209	12962.5	2144.6	1709.5	1713.4	1405.9	1658

Rio Grande Timbaúba dos

1230RN	24do Norte	Batistas	241430	2414308	2382	394.1	314.1	314.8	258.3	617

Rio Grande

1231RN	24do Norte	Touros	241440	2414407	32851.6	5435.3	4332.5	4342.3	3563	2358

Rio Grande

1232RN	24do Norte	Triunfo Potiguar	241445	2414456	3336.9	552.1	440.1	441.1	361.9	251

Rio Grande

1233RN	24do Norte	Umarizal	241450	2414506	10720.6	1773.7	1413.8	1417	1162.7	1242

Rio Grande

1234RN	24do Norte	Upanema	241460	2414605	13873.9	2295.4	1829.7	1833.8	1504.7	1477

Rio Grande

1235RN	24do Norte	Várzea	241470	2414704	5430.3	898.4	716.1	717.8	589	609

Rio Grande

1236RN	24do Norte	Venha-Ver	241475	2414753	4047.9	669.7	533.8	535	439	617

Rio Grande

1237RN	24do Norte	Vera Cruz	241480	2414803	11752.1	1944.4	1549.9	1553.4	1274.6	1937

Rio Grande

1238RN	24do Norte	Viçosa	241490	2414902	1687.4	279.2	222.5	223	183	561

Rio Grande

1239RN	24do Norte	Vila Flor	241500	2415008	3058.6	506	403.4	404.3	331.7	322

1240PB	Água Branca	250010	2500106	9938.2	1682.6	1285.1	1443.9	1138.2	1671

1241PB	Aguiar	250020	2500205	5564.5	942.1	719.5	808.4	637.3	609

1242PB	Alagoa Grande	250030	2500304	28563.6	4836.1	3693.6	4149.9	3271.4	2646

| 1243PB |  | Alagoa Nova | 250040 | 2500403 | 20321.2 | 3440.6 | 2627.7 | 2952.4 | 2327.4 | 2871 |  |
| --- | --- | --- | --- | --- | --- | --- | --- | --- | --- | --- | --- |
| 1244PB |  | Alagoinha | 250050 | 2500502 | 14123.1 | 2391.2 | 1826.3 | 2051.9 | 1617.5 | 1736 |  |
| 1245PB |  | Alcantil | 250053 | 2500536 | 5399.5 | 914.2 | 698.2 | 784.5 | 618.4 | 1004 |  |
| 1246PB | 25Paraíba | Algodão de  Jandaíra | 250057 | 2500577 | 2456 | 415.8 | 317.6 | 356.8 | 281.3 | 363 |  |
| 1247PB | 25Paraíba | Alhandra | 250060 | 2500601 | 18963.2 | 3210.7 | 2452.1 | 2755.1 | 2171.9 | 2068 |  |
| 1248PB | 25Paraíba | São João do Rio do Peixe | 250070 | 2500700 | 17966.3 | 3041.9 | 2323.2 | 2610.2 | 2057.7 | 1221 |  |
| 1249PB | 25Paraíba | Amparo | 250073 | 2500734 | 2183.4 | 369.7 | 282.3 | 317.2 | 250.1 | 1230 |  |
| 1250PB | 25Paraíba | Aparecida | 250077 | 2500775 | 8124.3 | 1375.5 | 1050.6 | 1180.3 | 930.5 | 1924 |  |
| 1251PB | 25Paraíba | Araçagi | 250080 | 2500809 | 17116.4 | 2898 | 2213.3 | 2486.8 | 1960.3 | 1709 |  |
| 1252PB | 25Paraíba | Arara | 250090 | 2500908 | 13187.5 | 2232.8 | 1705.3 | 1915.9 | 1510.4 | 1062 |  |
| 1253PB | 25Paraíba | Araruna | 250100 | 2501005 | 19775 | 3348.1 | 2557.1 | 2873 | 2264.8 | 723 |  |
| 1254PB | 25Paraíba | Areia | 250110 | 2501104 | 23225.5 | 3932.3 | 3003.3 | 3374.3 | 2660 | 2094 |  |
| 1255PB | 25Paraíba | Areia de Baraúnas | 250115 | 2501153 | 1995.4 | 337.8 | 258 | 289.9 | 228.5 | 384 |  |
| 1256PB | 25Paraíba | Areial | 250120 | 2501203 | 6793.4 | 1150.2 | 878.5 | 987 | 778 | 824 |  |
| 1257PB | 25Paraíba | Aroeiras | 250130 | 2501302 | 19151.5 | 3242.5 | 2476.5 | 2782.4 | 2193.4 | 2096 |  |
| 1258PB | 25Paraíba | Assunção | 250135 | 2501351 | 3769.5 | 638.2 | 487.4 | 547.7 | 431.7 | 677 |  |
| 1259PB | 25Paraíba | Baía da Traição | 250140 | 2501401 | 8641.4 | 1463.1 | 1117.4 | 1255.5 | 989.7 | 802 |  |
| 1260PB | 25Paraíba | Bananeiras | 250150 | 2501500 | 21480.7 | 3636.9 | 2777.7 | 3120.8 | 2460.2 | 1915 |  |
| 1261PB | 25Paraíba | Baraúna | 250153 | 2501534 | 4639.5 | 785.5 | 599.9 | 674 | 531.4 | 1158 |  |
| 1262PB | 25Paraíba | Barra de Santana | 250157 | 2501575 | 8254.3 | 1397.5 | 1067.4 | 1199.2 | 945.4 | 1009 |  |
| 1263PB | 25Paraíba | Barra de Santa  Rosa | 250160 | 2501609 | 14920.7 | 2526.2 | 1929.4 | 2167.8 | 1708.9 | 1906 |  |
| 1264PB | 25Paraíba | Barra de São  Miguel | 250170 | 2501708 | 5842.6 | 989.2 | 755.5 | 848.8 | 669.2 | 948 |  |
| 1265PB | 25Paraíba | Bayeux | 250180 | 2501807 | 98202.4 | 16626.6 | 12698.6 | 14267.3 | 11247.1 | 8555 |  |
| 1266PB | 25Paraíba | Belém | 250190 | 2501906 | 17466.5 | 2957.3 | 2258.6 | 2537.6 | 2000.4 | 1302 |  |
| 1267PB | 25Paraíba | Belém do Brejo do  Cruz | 250200 | 2502003 | 7273.4 | 1231.5 | 940.5 | 1056.7 | 833 | 769 |  |
| 1268PB | 25Paraíba | Bernardino Batista | 250205 | 2502052 | 3300.7 | 558.8 | 426.8 | 479.5 | 378 | 621 |  |
| 1269PB | 25Paraíba | Boa Ventura | 250210 | 2502102 | 5523.2 | 935.1 | 714.2 | 802.4 | 632.6 | 417 |  |
| 1270PB | 25Paraíba | Boa Vista | 250215 | 2502151 | 6738.8 | 1140.9 | 871.4 | 979 | 771.8 | 1325 |  |
| 1271PB | 25Paraíba | Bom Jesus | 250220 | 2502201 | 2501.4 | 423.5 | 323.5 | 363.4 | 286.5 | 311 |  |
| 1272PB | 25Paraíba | Bom Sucesso | 250230 | 2502300 | 5005.5 | 847.5 | 647.3 | 727.2 | 573.3 | 1239 |  |
| 1273PB | 25Paraíba | Bonito de Santa  Fé | 250240 | 2502409 | 11487.9 | 1945 | 1485.5 | 1669 | 1315.7 | 490 |  |
| 1274PB | 25Paraíba | Boqueirão | 250250 | 2502508 | 17468.5 | 2957.6 | 2258.9 | 2537.9 | 2000.7 | 2859 |  |
| 1275PB | 25Paraíba | Igaracy | 250260 | 2502607 | 6169.3 | 1044.5 | 797.8 | 896.3 | 706.6 | 490 |  |
| 1276PB | 25Paraíba | Borborema | 250270 | 2502706 | 5290.2 | 895.7 | 684.1 | 768.6 | 605.9 | 657 |  |
| 1277PB | 25Paraíba | Brejo do Cruz | 250280 | 2502805 | 13724.9 | 2323.8 | 1774.8 | 1994 | 1571.9 | 1825 |  |
| 1278PB | 25Paraíba | Brejo dos Santos | 250290 | 2502904 | 6359.1 | 1076.7 | 822.3 | 923.9 | 728.3 | 1061 |  |
| 1279PB | 25Paraíba | Caaporã | 250300 | 2503001 | 21279.3 | 3602.8 | 2751.6 | 3091.6 | 2437.1 | 1865 |  |
| 1280PB | 25Paraíba | Cabaceiras | 250310 | 2503100 | 5362.4 | 907.9 | 693.4 | 779.1 | 614.2 | 1111 |  |
| 1281PB | 25Paraíba | Cabedelo | 250320 | 2503209 | 63961 | 10829.2 | 8270.8 | 9292.6 | 7325.4 | 7369 |  |
| 1282PB | 25Paraíba | Cachoeira dos  Índios | 250330 | 2503308 | 9982.8 | 1690.2 | 1290.9 | 1450.4 | 1143.3 | 726 |  |
| 1283PB | 25Paraíba | Cacimba de Areia | 250340 | 2503407 | 3669.1 | 621.2 | 474.5 | 533.1 | 420.2 | 536 |  |
| 1284PB | 25Paraíba | Cacimba de  Dentro | 250350 | 2503506 | 17046.6 | 2886.2 | 2204.3 | 2476.6 | 1952.3 | 1149 |  |
| 1285PB | 25Paraíba | Cacimbas | 250355 | 2503555 | 7040.8 | 1192.1 | 910.4 | 1022.9 | 806.4 | 565 |  |
| 1286PB | 25Paraíba | Caiçara | 250360 | 2503605 | 7259.6 | 1229.1 | 938.7 | 1054.7 | 831.4 | 947 |  |
| 1287PB | 25Paraíba | Cajazeiras | 250370 | 2503704 | 60721.4 | 10280.7 | 7851.9 | 8821.9 | 6954.4 | 2809 |  |
| 1288PB | 25Paraíba | Cajazeirinhas | 250375 | 2503753 | 3133.7 | 530.6 | 405.2 | 455.3 | 358.9 | 532 |  |
| 1289PB | 25Paraíba | Caldas Brandão | 250380 | 2503803 | 5873.5 | 994.4 | 759.5 | 853.3 | 672.7 | 739 |  |
| 1290PB | 25Paraíba | Camalaú | 250390 | 2503902 | 5916.9 | 1001.8 | 765.1 | 859.6 | 677.7 | 1466 |  |
| 1291PB | 25Paraíba | Campina Grande | 250400 | 2504009 | 400612.6 | 67827.7 | 51803.2 | 58203 | 45882.1 | 90129 | 33 |
| 1292PB | 25Paraíba | Capim | 250403 | 2504033 | 6168.1 | 1044.3 | 797.6 | 896.1 | 706.4 | 515 |  |
| 1293PB | 25Paraíba | Caraúbas | 250407 | 2504074 | 4064.5 | 688.2 | 525.6 | 590.5 | 465.5 | 254 |  |
| 1294PB | 25Paraíba | Carrapateira | 250410 | 2504108 | 2551.7 | 432 | 330 | 370.7 | 292.2 | 238 |  |
| 1295PB | 25Paraíba | Casserengue | 250415 | 2504157 | 7314.5 | 1238.4 | 945.8 | 1062.7 | 837.7 | 1021 |  |
| 1296PB | 25Paraíba | Catingueira | 250420 | 2504207 | 4891 | 828.1 | 632.5 | 710.6 | 560.2 | 711 |  |
| 1297PB | 25Paraíba | Catolé do Rocha | 250430 | 2504306 | 29850.6 | 5054 | 3860 | 4336.8 | 3418.8 | 5862 |  |
| 1298PB | 25Paraíba | Caturité | 250435 | 2504355 | 4726 | 800.2 | 611.1 | 686.6 | 541.3 | 1165 |  |
| 1299PB | 25Paraíba | Conceição | 250440 | 2504405 | 18736.6 | 3172.3 | 2422.8 | 2722.1 | 2145.9 | 2333 |  |
| 1300PB | 25Paraíba | Condado | 250450 | 2504504 | 6673.8 | 1129.9 | 863 | 969.6 | 764.3 | 1045 |  |
| 1301PB | 25Paraíba | Conde | 250460 | 2504603 | 23412.3 | 3963.9 | 3027.4 | 3401.5 | 2681.4 | 2899 |  |
| 1302PB | 25Paraíba | Congo | 250470 | 2504702 | 4753.9 | 804.9 | 614.7 | 690.7 | 544.5 | 1191 |  |
| 1303PB | 25Paraíba | Coremas | 250480 | 2504801 | 15336.3 | 2596.6 | 1983.1 | 2228.1 | 1756.5 | 1213 |  |
| 1304PB | 25Paraíba | Coxixola | 250485 | 2504850 | 1864.4 | 315.7 | 241.1 | 270.9 | 213.5 | 789 |  |
| 1305PB | 25Paraíba | Cruz do Espírito  Santo | 250490 | 2504900 | 16944 | 2868.8 | 2191 | 2461.7 | 1940.6 | 1300 |  |
| 1306PB | 25Paraíba | Cubati | 250500 | 2505006 | 7222.5 | 1222.8 | 933.9 | 1049.3 | 827.2 | 1375 |  |
| 1307PB | 25Paraíba | Cuité | 250510 | 2505105 | 20224.3 | 3424.2 | 2615.2 | 2938.3 | 2316.3 | 3410 |  |
| 1308PB | 25Paraíba | Cuitegi | 250520 | 2505204 | 6841.8 | 1158.4 | 884.7 | 994 | 783.6 | 860 |  |
| 1309PB | 25Paraíba | Cuité de  Mamanguape | 250523 | 2505238 | 6302.5 | 1067.1 | 815 | 915.7 | 721.8 | 531 |  |
| 1310PB | 25Paraíba | Curral de Cima | 250527 | 2505279 | 5229.6 | 885.4 | 676.2 | 759.8 | 598.9 | 624 |  |
| 1311PB | 25Paraíba | Curral Velho | 250530 | 2505303 | 2515.5 | 425.9 | 325.3 | 365.5 | 288.1 | 467 |  |
| 1312PB | 25Paraíba | Damião | 250535 | 2505352 | 5167.7 | 874.9 | 668.2 | 750.8 | 591.9 | 872 |  |
| 1313PB | 25Paraíba | Desterro | 250540 | 2505402 | 8187.5 | 1386.2 | 1058.7 | 1189.5 | 937.7 | 906 |  |
| 1314PB | 25Paraíba | Vista Serrana | 250550 | 2505501 | 3691.1 | 624.9 | 477.3 | 536.3 | 422.7 | 524 |  |
| 1315PB | 25Paraíba | Diamante | 250560 | 2505600 | 6586.7 | 1115.2 | 851.7 | 956.9 | 754.4 | 561 |  |
| 1316PB | 25Paraíba | Dona Inês | 250570 | 2505709 | 10460.1 | 1771 | 1352.6 | 1519.7 | 1198 | 934 |  |
| 1317PB | 25Paraíba | Duas Estradas | 250580 | 2505808 | 3617.3 | 612.4 | 467.8 | 525.5 | 414.3 | 661 |  |
| 1318PB | 25Paraíba | Emas | 250590 | 2505907 | 3445.9 | 583.4 | 445.6 | 500.6 | 394.7 | 253 |  |
| 1319PB | 25Paraíba | Esperança | 250600 | 2506004 | 32383.9 | 5482.9 | 4187.6 | 4704.9 | 3708.9 | 4240 |  |
| 1320PB | 25Paraíba | Fagundes | 250610 | 2506103 | 11361.1 | 1923.5 | 1469.1 | 1650.6 | 1301.2 | 1366 |  |
| 1321PB | 25Paraíba | Frei Martinho | 250620 | 2506202 | 2971 | 503 | 384.2 | 431.6 | 340.3 | 916 |  |
| 1322PB | 25Paraíba | Gado Bravo | 250625 | 2506251 | 8411.2 | 1424.1 | 1087.7 | 1222 | 963.3 | 1170 |  |
| 1323PB | 25Paraíba | Guarabira | 250630 | 2506301 | 57502 | 9735.7 | 7435.6 | 8354.2 | 6585.7 | 13977 |  |
| 1324PB | 25Paraíba | Gurinhém | 250640 | 2506400 | 14046.6 | 2378.2 | 1816.4 | 2040.8 | 1608.8 | 1415 |  |
| 1325PB | 25Paraíba | Gurjão | 250650 | 2506509 | 3326.6 | 563.2 | 430.2 | 483.3 | 381 | 1083 |  |
| 1326PB | 25Paraíba | Ibiara | 250660 | 2506608 | 5977.3 | 1012 | 772.9 | 868.4 | 684.6 | 498 |  |
| 1327PB | 25Paraíba | Imaculada | 250670 | 2506707 | 11651 | 1972.6 | 1506.6 | 1692.7 | 1334.4 | 1146 |  |
| 1328PB | 25Paraíba | Ingá | 250680 | 2506806 | 18037.3 | 3053.9 | 2332.4 | 2620.5 | 2065.8 | 3102 |  |
| 1329PB | 25Paraíba | Itabaiana | 250690 | 2506905 | 24512.7 | 4150.2 | 3169.7 | 3561.3 | 2807.4 | 1835 |  |
| 1330PB | 25Paraíba | Itaporanga | 250700 | 2507002 | 24185.3 | 4094.8 | 3127.4 | 3513.8 | 2769.9 | 1019 |  |
| 1331PB | 25Paraíba | Itapororoca | 250710 | 2507101 | 18031.5 | 3052.9 | 2331.7 | 2619.7 | 2065.1 | 3055 |  |
| 1332PB | 25Paraíba | Itatuba | 250720 | 2507200 | 10623.4 | 1798.6 | 1373.7 | 1543.4 | 1216.7 | 1186 |  |
| 1333PB | 25Paraíba | Jacaraú | 250730 | 2507309 | 14225.3 | 2408.5 | 1839.5 | 2066.7 | 1629.2 | 1777 |  |
| 1334PB | 25Paraíba | Jericó | 250740 | 2507408 | 7669.4 | 1298.5 | 991.7 | 1114.2 | 878.4 | 1509 |  |
| 1335PB | 25Paraíba | João Pessoa | 250750 | 2507507 | 776358.4 | 131445.2 | 100390.9 | 112793.2 | 88916.2 | 146210 | 52 |
| 1336PB | 25Paraíba | Juarez Távora | 250760 | 2507606 | 7758.6 | 1313.6 | 1003.3 | 1127.2 | 888.6 | 833 |  |
| 1337PB | 25Paraíba | Juazeirinho | 250770 | 2507705 | 17645.2 | 2987.5 | 2281.7 | 2563.6 | 2020.9 | 1085 |  |
| 1338PB | 25Paraíba | Junco do Seridó | 250780 | 2507804 | 6959.6 | 1178.3 | 899.9 | 1011.1 | 797.1 | 751 |  |
| 1339PB | 25Paraíba | Juripiranga | 250790 | 2507903 | 10565.5 | 1788.8 | 1366.2 | 1535 | 1210.1 | 1918 |  |
| 1340PB | 25Paraíba | Juru | 250800 | 2508000 | 9864.6 | 1670.2 | 1275.6 | 1433.2 | 1129.8 | 1043 |  |
| 1341PB | 25Paraíba | Lagoa | 250810 | 2508109 | 4678.8 | 792.2 | 605 | 679.8 | 535.9 | 785 |  |
| 1342PB | 25Paraíba | Lagoa de Dentro | 250820 | 2508208 | 7562.7 | 1280.4 | 977.9 | 1098.7 | 866.2 | 1364 |  |
| 1343PB | 25Paraíba | Lagoa Seca | 250830 | 2508307 | 26891.2 | 4552.9 | 3477.3 | 3906.9 | 3079.8 | 2319 |  |
| 1344PB |  | Lastro | 250840 | 2508406 | 2777.8 | 470.3 | 359.2 | 403.6 | 318.1 | 220 |  |
| 1345PB |  | Livramento | 250850 | 2508505 | 7292.6 | 1234.7 | 943 | 1059.5 | 835.2 | 1294 |  |
| 1346PB |  | Logradouro | 250855 | 2508554 | 4183.7 | 708.3 | 541 | 607.8 | 479.2 | 480 |  |

3

2

1

2

1

1

1

5

| 1347PB |  | Lucena | 250860 | 2508604 | 12565 | 2127.4 | 1624.8 | 1825.5 | 1439.1 | 1623 |
| --- | --- | --- | --- | --- | --- | --- | --- | --- | --- | --- |
| 1348PB |  | Mãe d'Água | 250870 | 2508703 | 4018.7 | 680.4 | 519.7 | 583.9 | 460.3 | 685 |
| 1349PB |  | Malta | 250880 | 2508802 | 5670.6 | 960.1 | 733.3 | 823.9 | 649.5 | 1137 |
| 1350PB | 25Paraíba | Mamanguape | 250890 | 2508901 | 43875.7 | 7428.6 | 5673.6 | 6374.5 | 5025.1 | 4391 |
| 1351PB | 25Paraíba | Manaíra | 250900 | 2509008 | 10971.4 | 1857.6 | 1418.7 | 1594 | 1256.6 | 699 |
| 1352PB | 25Paraíba | Marcação | 250905 | 2509057 | 8194.6 | 1387.4 | 1059.6 | 1190.6 | 938.5 | 764 |
| 1353PB | 25Paraíba | Mari | 250910 | 2509107 | 21605.6 | 3658 | 2793.8 | 3139 | 2474.5 | 2514 |
| 1354PB | 25Paraíba | Marizópolis | 250915 | 2509156 | 6442.9 | 1090.8 | 833.1 | 936.1 | 737.9 | 623 |
| 1355PB | 25Paraíba | Massaranduba | 250920 | 2509206 | 13488.8 | 2283.8 | 1744.2 | 1959.7 | 1544.9 | 1780 |
| 1356PB | 25Paraíba | Mataraca | 250930 | 2509305 | 8040.5 | 1361.3 | 1039.7 | 1168.2 | 920.9 | 663 |
| 1357PB | 25Paraíba | Matinhas | 250933 | 2509339 | 4451.2 | 753.6 | 575.6 | 646.7 | 509.8 | 718 |
| 1358PB | 25Paraíba | Mato Grosso | 250937 | 2509370 | 2830.8 | 479.3 | 366.1 | 411.3 | 324.2 | 543 |
| 1359PB | 25Paraíba | Maturéia | 250939 | 2509396 | 6328.9 | 1071.5 | 818.4 | 919.5 | 724.8 | 965 |
| 1360PB | 25Paraíba | Mogeiro | 250940 | 2509404 | 13131.4 | 2223.3 | 1698 | 1907.8 | 1503.9 | 1608 |
| 1361PB | 25Paraíba | Montadas | 250950 | 2509503 | 5409.2 | 915.8 | 699.5 | 785.9 | 619.5 | 1040 |
| 1362PB | 25Paraíba | Monte Horebe | 250960 | 2509602 | 4700.8 | 795.9 | 607.9 | 683 | 538.4 | 302 |
| 1363PB | 25Paraíba | Monteiro | 250970 | 2509701 | 32332.2 | 5474.2 | 4180.9 | 4697.4 | 3703 | 10574 |
| 1364PB | 25Paraíba | Mulungu | 250980 | 2509800 | 9748.7 | 1650.6 | 1260.6 | 1416.3 | 1116.5 | 1830 |
| 1365PB | 25Paraíba | Natuba | 250990 | 2509909 | 10457.1 | 1770.5 | 1352.2 | 1519.3 | 1197.7 | 743 |
| 1366PB | 25Paraíba | Nazarezinho | 251000 | 2510006 | 7300.4 | 1236 | 944 | 1060.6 | 836.1 | 418 |
| 1367PB | 25Paraíba | Nova Floresta | 251010 | 2510105 | 10610.8 | 1796.5 | 1372.1 | 1541.6 | 1215.3 | 1348 |
| 1368PB | 25Paraíba | Nova Olinda | 251020 | 2510204 | 6004.9 | 1016.7 | 776.5 | 872.4 | 687.7 | 640 |
| 1369PB | 25Paraíba | Nova Palmeira | 251030 | 2510303 | 4691.5 | 794.3 | 606.7 | 681.6 | 537.3 | 633 |
| 1370PB | 25Paraíba | Olho d'Água | 251040 | 2510402 | 6694.9 | 1133.5 | 865.7 | 972.7 | 766.8 | 491 |
| 1371PB | 25Paraíba | Olivedos | 251050 | 2510501 | 3821.8 | 647.1 | 494.2 | 555.2 | 437.7 | 825 |
| 1372PB | 25Paraíba | Ouro Velho | 251060 | 2510600 | 2999.4 | 507.8 | 387.9 | 435.8 | 343.5 | 725 |
| 1373PB | 25Paraíba | Parari | 251065 | 2510659 | 1684.9 | 285.3 | 217.9 | 244.8 | 193 | 592 |
| 1374PB | 25Paraíba | Passagem | 251070 | 2510709 | 2348.8 | 397.7 | 303.7 | 341.2 | 269 | 253 |
| 1375PB | 25Paraíba | Patos | 251080 | 2510808 | 105005.9 | 17778.5 | 13578.3 | 15255.8 | 12026.3 | 16948 |
| 1376PB | 25Paraíba | Paulista | 251090 | 2510907 | 12116.1 | 2051.4 | 1566.7 | 1760.3 | 1387.7 | 1575 |
| 1377PB | 25Paraíba | Pedra Branca | 251100 | 2511004 | 3774.8 | 639.1 | 488.1 | 548.4 | 432.3 | 258 |
| 1378PB | 25Paraíba | Pedra Lavrada | 251110 | 2511103 | 7812.4 | 1322.7 | 1010.2 | 1135 | 894.8 | 1117 |
| 1379PB | 25Paraíba | Pedras de Fogo | 251120 | 2511202 | 28015.7 | 4743.3 | 3622.7 | 4070.3 | 3208.6 | 2414 |
| 1380PB | 25Paraíba | Piancó | 251130 | 2511301 | 15855.8 | 2684.5 | 2050.3 | 2303.6 | 1816 | 1017 |
| 1381PB | 25Paraíba | Picuí | 251140 | 2511400 | 18549.2 | 3140.6 | 2398.6 | 2694.9 | 2124.4 | 5103 |
| 1382PB | 25Paraíba | Pilar | 251150 | 2511509 | 11646.6 | 1971.9 | 1506 | 1692.1 | 1333.9 | 2043 |
| 1383PB | 25Paraíba | Pilões | 251160 | 2511608 | 6771.7 | 1146.5 | 875.6 | 983.8 | 775.6 | 960 |
| 1384PB | 25Paraíba | Pilõezinhos | 251170 | 2511707 | 5107.8 | 864.8 | 660.5 | 742.1 | 585 | 593 |
| 1385PB | 25Paraíba | Pirpirituba | 251180 | 2511806 | 10493.2 | 1776.6 | 1356.9 | 1524.5 | 1201.8 | 1158 |
| 1386PB | 25Paraíba | Pitimbu | 251190 | 2511905 | 18312.2 | 3100.4 | 2368 | 2660.5 | 2097.3 | 1353 |
| 1387PB | 25Paraíba | Pocinhos | 251200 | 2512002 | 17990.5 | 3046 | 2326.4 | 2613.7 | 2060.4 | 2809 |
| 1388PB | 25Paraíba | Poço Dantas | 251203 | 2512036 | 3779.4 | 639.9 | 488.7 | 549.1 | 432.9 | 223 |
| 1389PB | 25Paraíba | Poço de José de  Moura | 251207 | 2512077 | 4183.5 | 708.3 | 541 | 607.8 | 479.1 | 410 |
| 1390PB | 25Paraíba | Pombal | 251210 | 2512101 | 32547.1 | 5510.5 | 4208.7 | 4728.6 | 3727.6 | 5289 |
| 1391PB | 25Paraíba | Prata | 251220 | 2512200 | 4054.8 | 686.5 | 524.3 | 589.1 | 464.4 | 1149 |
| 1392PB | 25Paraíba | Princesa Isabel | 251230 | 2512309 | 22603 | 3826.9 | 2922.8 | 3283.9 | 2588.7 | 2968 |
| 1393PB | 25Paraíba | Puxinanã | 251240 | 2512408 | 13406.3 | 2269.8 | 1733.6 | 1947.7 | 1535.4 | 1612 |
| 1394PB | 25Paraíba | Queimadas | 251250 | 2512507 | 42906.4 | 7264.5 | 5548.2 | 6233.7 | 4914.1 | 4926 |
| 1395PB | 25Paraíba | Quixabá | 251260 | 2512606 | 1857.2 | 314.4 | 240.2 | 269.8 | 212.7 | 332 |
| 1396PB | 25Paraíba | Remígio | 251270 | 2512705 | 18810.7 | 3184.8 | 2432.4 | 2732.9 | 2154.4 | 2402 |
| 1397PB | 25Paraíba | Pedro Régis | 251272 | 2512721 | 5969.9 | 1010.8 | 772 | 867.3 | 683.7 | 338 |
| 1398PB | 25Paraíba | Riachão | 251274 | 2512747 | 3470.4 | 587.6 | 448.8 | 504.2 | 397.5 | 369 |
| 1399PB | 25Paraíba | Riachão do  Bacamarte | 251275 | 2512754 | 4426 | 749.4 | 572.3 | 643 | 506.9 | 656 |
| 1400PB | 25Paraíba | Riachão do Poço | 251276 | 2512762 | 4378.7 | 741.4 | 566.2 | 636.2 | 501.5 | 497 |
| 1401PB | 25Paraíba | Riacho de Santo  Antônio | 251278 | 2512788 | 1876.3 | 317.7 | 242.6 | 272.6 | 214.9 | 329 |
| 1402PB | 25Paraíba | Riacho dos  Cavalos | 251280 | 2512804 | 8492.2 | 1437.8 | 1098.1 | 1233.8 | 972.6 | 1360 |
| 1403PB | 25Paraíba | Rio Tinto | 251290 | 2512903 | 23780.3 | 4026.2 | 3075 | 3454.9 | 2723.6 | 3354 |
| 1404PB | 25Paraíba | Salgadinho | 251300 | 2513000 | 3782.8 | 640.5 | 489.2 | 549.6 | 433.2 | 450 |
| 1405PB | 25Paraíba | Salgado de São  Félix | 251310 | 2513109 | 12092.4 | 2047.4 | 1563.7 | 1756.8 | 1384.9 | 1631 |
| 1406PB | 25Paraíba | Santa Cecília | 251315 | 2513158 | 6588.1 | 1115.4 | 851.9 | 957.2 | 754.5 | 1085 |
| 1407PB | 25Paraíba | Santa Cruz | 251320 | 2513208 | 6547.6 | 1108.6 | 846.7 | 951.3 | 749.9 | 582 |
| 1408PB | 25Paraíba | Santa Helena | 251330 | 2513307 | 5799.1 | 981.8 | 749.9 | 842.5 | 664.2 | 747 |
| 1409PB | 25Paraíba | Santa Inês | 251335 | 2513356 | 3577.7 | 605.7 | 462.6 | 519.8 | 409.8 | 342 |
| 1410PB | 25Paraíba | Santa Luzia | 251340 | 2513406 | 15141.4 | 2563.6 | 1957.9 | 2199.8 | 1734.1 | 1518 |
| 1411PB | 25Paraíba | Santana de  Mangueira | 251350 | 2513505 | 5237.5 | 886.8 | 677.3 | 760.9 | 599.9 | 486 |
| 1412PB | 25Paraíba | Santana dos  Garrotes | 251360 | 2513604 | 7133.9 | 1207.8 | 922.5 | 1036.4 | 817 | 629 |
| 1413PB | 25Paraíba | Santa Rita | 251370 | 2513703 | 130277.4 | 22057.3 | 16846.2 | 18927.3 | 14920.7 | 17521 |
| 1414PB | 25Paraíba | Santa Teresinha | 251380 | 2513802 | 4582.8 | 775.9 | 592.6 | 665.8 | 524.9 | 664 |
| 1415PB | 25Paraíba | Santo André | 251385 | 2513851 | 2556.9 | 432.9 | 330.6 | 371.5 | 292.8 | 790 |
| 1416PB | 25Paraíba | São Bento | 251390 | 2513901 | 32876.6 | 5566.3 | 4251.3 | 4776.5 | 3765.4 | 3025 |
| 1417PB | 25Paraíba | São Bentinho | 251392 | 2513927 | 4380.7 | 741.7 | 566.5 | 636.4 | 501.7 | 265 |
| 1418PB | 25Paraíba | São Domingos do  Cariri | 251394 | 2513943 | 2532 | 428.7 | 327.4 | 367.9 | 290 | 294 |
| 1419PB | 25Paraíba | São Francisco | 251398 | 2513984 | 3371 | 570.7 | 435.9 | 489.8 | 386.1 | 828 |
| 1420PB | 25Paraíba | São João do Cariri | 251400 | 2514008 | 4311.1 | 729.9 | 557.5 | 626.3 | 493.7 | 911 |
| 1421PB | 25Paraíba | São João do Tigre | 251410 | 2514107 | 4418.2 | 748 | 571.3 | 641.9 | 506 | 1017 |
| 1422PB | 25Paraíba | São José da  Lagoa Tapada | 251420 | 2514206 | 7637.9 | 1293.2 | 987.7 | 1109.7 | 874.8 | 619 |
| 1423PB | 25Paraíba | São José de  Caiana | 251430 | 2514305 | 6185.8 | 1047.3 | 799.9 | 898.7 | 708.5 | 430 |
| 1424PB | 25Paraíba | São José de  Espinharas | 251440 | 2514404 | 4697.5 | 795.3 | 607.4 | 682.5 | 538 | 569 |
| 1425PB | 25Paraíba | São José dos  Ramos | 251445 | 2514453 | 5787.3 | 979.8 | 748.4 | 840.8 | 662.8 | 835 |
| 1426PB | 25Paraíba | São José de  Piranhas | 251450 | 2514503 | 19763 | 3346.1 | 2555.6 | 2871.3 | 2263.5 | 1522 |
| 1427PB | 25Paraíba | São José de  Princesa | 251455 | 2514552 | 4018.3 | 680.3 | 519.6 | 583.8 | 460.2 | 355 |
| 1428PB | 25Paraíba | São José do  Bonfim | 251460 | 2514602 | 3433.8 | 581.4 | 444 | 498.9 | 393.3 | 309 |
| 1429PB | 25Paraíba | São José do Brejo do Cruz | 251465 | 2514651 | 1757.8 | 297.6 | 227.3 | 255.4 | 201.3 | 209 |
| 1430PB | 25Paraíba | São José do  Sabugi | 251470 | 2514701 | 4095 | 693.3 | 529.5 | 594.9 | 469 | 452 |
| 1431PB | 25Paraíba | São José dos  Cordeiros | 251480 | 2514800 | 3766.6 | 637.7 | 487.1 | 547.2 | 431.4 | 829 |
| 1432PB | 25Paraíba | São Mamede | 251490 | 2514909 | 7743.5 | 1311.1 | 1001.3 | 1125 | 886.9 | 1286 |
| 1433PB | 25Paraíba | São Miguel de  Taipu | 251500 | 2515005 | 7010.6 | 1187 | 906.5 | 1018.5 | 802.9 | 576 |
| 1434PB | 25Paraíba | São Sebastião de  Lagoa de Roça | 251510 | 2515104 | 11513.6 | 1949.4 | 1488.8 | 1672.8 | 1318.7 | 1502 |
| 1435PB | 25Paraíba | São Sebastião do  Umbuzeiro | 251520 | 2515203 | 3393.4 | 574.5 | 438.8 | 493 | 388.6 | 857 |
| 1436PB | 25Paraíba | Sapé | 251530 | 2515302 | 51717.5 | 8756.3 | 6687.6 | 7513.8 | 5923.2 | 6789 |
| 1437PB | 25Paraíba | Serra Branca | 251550 | 2515500 | 13424.6 | 2272.9 | 1735.9 | 1950.4 | 1537.5 | 2207 |
| 1438PB |  | Serra da Raiz | 251560 | 2515609 | 3162.2 | 535.4 | 408.9 | 459.4 | 362.2 | 855 |
| 1439PB |  | Serra Grande | 251570 | 2515708 | 3034 | 513.7 | 392.3 | 440.8 | 347.5 | 318 |
| 1440PB |  | Serra Redonda | 251580 | 2515807 | 7044.3 | 1192.7 | 910.9 | 1023.4 | 806.8 | 1124 |

1

3

6

1

1

2

1

6

1

2

| 1441PB |  | Serraria | 251590 | 2515906 | 6162.1 | 1043.3 | 796.8 | 895.3 | 705.7 | 885 |  |
| --- | --- | --- | --- | --- | --- | --- | --- | --- | --- | --- | --- |
| 1442PB |  | Sertãozinho | 251593 | 2515930 | 4782.8 | 809.8 | 618.5 | 694.9 | 547.8 | 786 |  |
| 1443PB |  | Sobrado | 251597 | 2515971 | 7631.7 | 1292.1 | 986.9 | 1108.8 | 874.1 | 665 |  |
| 1444PB | 25Paraíba | Solânea | 251600 | 2516003 | 26553.7 | 4495.8 | 3433.7 | 3857.9 | 3041.2 | 3513 |  |
| 1445PB | 25Paraíba | Soledade | 251610 | 2516102 | 14496.7 | 2454.4 | 1874.6 | 2106.2 | 1660.3 | 1473 |  |
| 1446PB | 25Paraíba | Sossêgo | 251615 | 2516151 | 3407.6 | 576.9 | 440.6 | 495.1 | 390.3 | 1104 |  |
| 1447PB | 25Paraíba | Sousa | 251620 | 2516201 | 68103.6 | 11530.6 | 8806.5 | 9894.4 | 7799.9 | 11983 |  |
| 1448PB | 25Paraíba | Sumé | 251630 | 2516300 | 16614.3 | 2813 | 2148.4 | 2413.8 | 1902.8 | 4500 |  |
| 1449PB | 25Paraíba | Taperoá | 251650 | 2516508 | 15166.4 | 2567.8 | 1961.2 | 2203.4 | 1737 | 1974 |  |
| 1450PB | 25Paraíba | Tavares | 251660 | 2516607 | 14459.7 | 2448.2 | 1869.8 | 2100.8 | 1656.1 | 1038 |  |
| 1451PB | 25Paraíba | Teixeira | 251670 | 2516706 | 14784 | 2503.1 | 1911.7 | 2147.9 | 1693.2 | 1844 |  |
| 1452PB | 25Paraíba | Tenório | 251675 | 2516755 | 2965.8 | 502.1 | 383.5 | 430.9 | 339.7 | 739 |  |
| 1453PB | 25Paraíba | Triunfo | 251680 | 2516805 | 9385.3 | 1589 | 1213.6 | 1363.5 | 1074.9 | 1059 |  |
| 1454PB | 25Paraíba | Uiraúna | 251690 | 2516904 | 15062.6 | 2550.2 | 1947.7 | 2188.4 | 1725.1 | 1479 |  |
| 1455PB | 25Paraíba | Umbuzeiro | 251700 | 2517001 | 9755 | 1651.6 | 1261.4 | 1417.3 | 1117.2 | 1316 |  |
| 1456PB | 25Paraíba | Várzea | 251710 | 2517100 | 2693.3 | 456 | 348.3 | 391.3 | 308.5 | 438 |  |
| 1457PB | 25Paraíba | Vieirópolis | 251720 | 2517209 | 5236.2 | 886.5 | 677.1 | 760.7 | 599.7 | 840 |  |
| 1458PB | 25Paraíba | Zabelê | 251740 | 2517407 | 2177.9 | 368.7 | 281.6 | 316.4 | 249.4 | 736 |  |
| 1459PE | 26PernambucoAbreu e Lima | | 260005 | 2600054 | 97707 | 16372.8 | 12830.9 | 13355.3 | 10479 | 29452 |  |
| 1460PE | Afogados da  26PernambucoIngazeira | | 260010 | 2600104 | 36364.6 | 6093.6 | 4775.4 | 4970.6 | 3900.1 | 16278 |  |
| 1461PE | 26PernambucoAfrânio | | 260020 | 2600203 | 18755.8 | 3142.9 | 2463 | 2563.7 | 2011.5 | 1465 |  |
| 1462PE | 26PernambucoAgrestina | | 260030 | 2600302 | 23947.5 | 4012.9 | 3144.8 | 3273.3 | 2568.3 | 3013 |  |
| 1463PE | 26PernambucoÁgua Preta | | 260040 | 2600401 | 35198.1 | 5898.1 | 4622.2 | 4811.1 | 3775 | 3009 |  |
| 1464PE | 26PernambucoÁguas Belas | | 260050 | 2600500 | 42094.5 | 7053.8 | 5527.8 | 5753.8 | 4514.6 | 4003 |  |
| 1465PE | 26PernambucoAlagoinha | | 260060 | 2600609 | 14223.7 | 2383.5 | 1867.9 | 1944.2 | 1525.5 | 2529 |  |
| 1466PE | 26PernambucoAliança | | 260070 | 2600708 | 38033.1 | 6373.2 | 4994.5 | 5198.6 | 4079 | 5676 |  |
| 1467PE | 26PernambucoAltinho | | 260080 | 2600807 | 22740.1 | 3810.6 | 2986.2 | 3108.3 | 2438.9 | 6132 |  |
| 1468PE | 26PernambucoAmaraji | | 260090 | 2600906 | 22478.7 | 3766.8 | 2951.9 | 3072.5 | 2410.8 | 1854 |  |
| 1469PE | 26PernambucoAngelim | | 260100 | 2601003 | 10747.7 | 1801 | 1411.4 | 1469.1 | 1152.7 | 1560 |  |
| 1470PE | 26PernambucoAraçoiaba | | 260105 | 2601052 | 19504.1 | 3268.3 | 2561.3 | 2666 | 2091.8 | 2168 |  |
| 1471PE | 26PernambucoAraripina | | 260110 | 2601102 | 81446.7 | 13648 | 10695.6 | 11132.7 | 8735.1 | 4902 |  |
| 1472PE | 26PernambucoArcoverde | | 260120 | 2601201 | 72042 | 12072.1 | 9460.6 | 9847.2 | 7726.4 | 18097 |  |
| 1473PE | Barra de 26PernambucoGuabiraba | | 260130 | 2601300 | 13698.8 | 2295.5 | 1798.9 | 1872.4 | 1469.2 | 1351 |  |
| 1474PE | 26PernambucoBarreiros | | 260140 | 2601409 | 41885.1 | 7018.7 | 5500.4 | 5725.1 | 4492.1 | 3839 |  |
| 1475PE | 26PernambucoBelém de Maria | | 260150 | 2601508 | 11775.2 | 1973.2 | 1546.3 | 1609.5 | 1262.9 | 1683 |  |
| 1476PE | 26PernambucoBelo Jardim | | 260170 | 2601706 | 74803.6 | 12534.8 | 9823.2 | 10224.7 | 8022.6 | 8017 |  |
| 1477PE | 26PernambucoBetânia | | 260180 | 2601805 | 12424.4 | 2082 | 1631.6 | 1698.3 | 1332.5 | 1172 |  |
| 1478PE | 26PernambucoBezerros | | 260190 | 2601904 | 59973 | 10049.7 | 7875.7 | 8197.5 | 6432 | 16891 |  |
| 1479PE | 26PernambucoBodocó | | 260200 | 2602001 | 36885.1 | 6180.8 | 4843.8 | 5041.7 | 3955.9 | 2649 |  |
| 1480PE | 26PernambucoBom Conselho | | 260210 | 2602100 | 47288.1 | 7924.1 | 6209.9 | 6463.7 | 5071.6 | 7945 |  |
| 1481PE | 26PernambucoBom Jardim | | 260220 | 2602209 | 38659.1 | 6478.1 | 5076.7 | 5284.2 | 4146.2 | 7234 |  |
| 1482PE | 26PernambucoBonito | | 260230 | 2602308 | 37955.9 | 6360.3 | 4984.4 | 5188.1 | 4070.7 | 5848 |  |
| 1483PE | 26PernambucoBrejão | | 260240 | 2602407 | 8945.3 | 1499 | 1174.7 | 1222.7 | 959.4 | 1333 |  |
| 1484PE | 26PernambucoBrejinho | | 260250 | 2602506 | 7422.2 | 1243.7 | 974.7 | 1014.5 | 796 | 2887 |  |
| 1485PE | Brejo da Madre de  26PernambucoDeus | | 260260 | 2602605 | 48350.9 | 8102.2 | 6349.4 | 6608.9 | 5185.6 | 3831 |  |
| 1486PE | 26PernambucoBuenos Aires | | 260270 | 2602704 | 12906.2 | 2162.7 | 1694.8 | 1764.1 | 1384.2 | 2615 |  |
| 1487PE | 26PernambucoBuíque | | 260280 | 2602803 | 55683.8 | 9330.9 | 7312.4 | 7611.2 | 5972 | 7165 |  |
| 1488PE | Cabo de Santo  26PernambucoAgostinho | | 260290 | 2602902 | 197593.5 | 33110.7 | 25948 | 27008.4 | 21191.7 | 59454 | 16 |
| 1489PE | 26PernambucoCabrobó | | 260300 | 2603009 | 32789.8 | 5494.6 | 4306 | 4481.9 | 3516.7 | 1729 |  |
| 1490PE | 26PernambucoCachoeirinha | | 260310 | 2603108 | 19723 | 3305 | 2590 | 2695.9 | 2115.3 | 1688 |  |
| 1491PE | 26PernambucoCaetés | | 260320 | 2603207 | 27829.2 | 4663.3 | 3654.5 | 3803.9 | 2984.7 | 2819 |  |
| 1492PE | 26PernambucoCalçado | | 260330 | 2603306 | 11109.2 | 1861.6 | 1458.9 | 1518.5 | 1191.5 | 1564 |  |
| 1493PE | 26PernambucoCalumbi | | 260340 | 2603405 | 5716.4 | 957.9 | 750.7 | 781.4 | 613.1 | 974 |  |
| 1494PE | 26PernambucoCamaragibe | | 260345 | 2603454 | 152162.3 | 25497.8 | 19982 | 20798.6 | 16319.3 | 41314 | 14 |
| 1495PE | Camocim de São  26PernambucoFélix | | 260350 | 2603504 | 18059.1 | 3026.2 | 2371.5 | 2468.4 | 1936.8 | 1870 |  |
| 1496PE | 26PernambucoCamutanga | | 260360 | 2603603 | 8386.9 | 1405.4 | 1101.4 | 1146.4 | 899.5 | 1474 |  |
| 1497PE | 26PernambucoCanhotinho | | 260370 | 2603702 | 24730.4 | 4144.1 | 3247.6 | 3380.3 | 2652.3 | 3139 |  |
| 1498PE | 26PernambucoCapoeiras | | 260380 | 2603801 | 19887 | 3332.5 | 2611.6 | 2718.3 | 2132.9 | 2930 |  |
| 1499PE | 26PernambucoCarnaíba | | 260390 | 2603900 | 19154.2 | 3209.7 | 2515.3 | 2618.1 | 2054.3 | 6094 |  |
| 1500PE | Carnaubeira da  26PernambucoPenha | | 260392 | 2603926 | 12442.6 | 2085 | 1634 | 1700.7 | 1334.5 | 1335 |  |
| 1501PE | 26PernambucoCarpina | | 260400 | 2604007 | 79872.9 | 13384.3 | 10488.9 | 10917.6 | 8566.3 | 14176 |  |
| 1502PE | 26PernambucoCaruaru | | 260410 | 2604106 | 341122.2 | 57161.8 | 44796.2 | 46626.9 | 36585 | 35519 | 10 |
| 1503PE | 26PernambucoCasinhas | | 260415 | 2604155 | 14113.6 | 2365 | 1853.4 | 1929.1 | 1513.7 | 2788 |  |
| 1504PE | 26PernambucoCatende | | 260420 | 2604205 | 40704.4 | 6820.8 | 5345.3 | 5563.8 | 4365.5 | 2579 |  |
| 1505PE | 26PernambucoCedro | | 260430 | 2604304 | 11370.4 | 1905.3 | 1493.2 | 1554.2 | 1219.5 | 1633 |  |
| 1506PE | 26PernambucoChã de Alegria | | 260440 | 2604403 | 13046.6 | 2186.2 | 1713.3 | 1783.3 | 1399.2 | 2681 |  |
| 1507PE | 26PernambucoChã Grande | | 260450 | 2604502 | 21043.2 | 3526.2 | 2763.4 | 2876.3 | 2256.9 | 2854 |  |
| 1508PE | 26PernambucoCondado | | 260460 | 2604601 | 25516.7 | 4275.8 | 3350.9 | 3487.8 | 2736.6 | 2972 |  |
| 1509PE | 26PernambucoCorrentes | | 260470 | 2604700 | 17846.4 | 2990.5 | 2343.6 | 2439.4 | 1914 | 1592 |  |
| 1510PE | 26PernambucoCortês | | 260480 | 2604809 | 12549.2 | 2102.9 | 1648 | 1715.3 | 1345.9 | 1470 |  |
| 1511PE | 26PernambucoCumaru | | 260490 | 2604908 | 14032.1 | 2351.4 | 1842.7 | 1918 | 1504.9 | 3636 |  |
| 1512PE | 26PernambucoCupira | | 260500 | 2605004 | 23731 | 3976.6 | 3116.4 | 3243.7 | 2545.1 | 2707 |  |
| 1513PE | 26PernambucoCustódia | | 260510 | 2605103 | 35727.1 | 5986.8 | 4691.7 | 4883.4 | 3831.7 | 5138 |  |
| 1514PE | 26PernambucoDormentes | | 260515 | 2605152 | 18053.8 | 3025.3 | 2370.8 | 2467.7 | 1936.3 | 2259 |  |
| 1515PE | 26PernambucoEscada | | 260520 | 2605202 | 66608 | 11161.5 | 8747 | 9104.4 | 7143.6 | 9505 |  |
| 1516PE | 26PernambucoExu | | 260530 | 2605301 | 31809.3 | 5330.3 | 4177.2 | 4347.9 | 3411.5 | 2173 |  |
| 1517PE | 26PernambucoFeira Nova | | 260540 | 2605400 | 21477.5 | 3599 | 2820.4 | 2935.7 | 2303.4 | 5459 |  |
| 1518PE | Fernando de  26PernambucoNoronha | | 260545 | 2605459 | 2874.5 | 481.7 | 377.5 | 392.9 | 308.3 | 105 |  |
| 1519PE | 26PernambucoFerreiros | | 260550 | 2605509 | 12071.5 | 2022.8 | 1585.2 | 1650 | 1294.7 | 2169 |  |
| 1520PE | 26PernambucoFlores | | 260560 | 2605608 | 22469.2 | 3765.2 | 2950.7 | 3071.2 | 2409.8 | 4026 |  |
| 1521PE | 26PernambucoFloresta | | 260570 | 2605707 | 31330.8 | 5250.1 | 4114.4 | 4282.5 | 3360.2 | 3504 |  |
| 1522PE | 26PernambucoFrei Miguelinho | | 260580 | 2605806 | 14966.9 | 2508 | 1965.5 | 2045.8 | 1605.2 | 2303 |  |
| 1523PE | 26PernambucoGameleira | | 260590 | 2605905 | 29707 | 4978 | 3901.1 | 4060.6 | 3186 | 2380 |  |
| 1524PE | 26PernambucoGaranhuns | | 260600 | 2606002 | 135424.7 | 22693.1 | 17784 | 18510.8 | 14524.2 | 26852 |  |
| 1525PE | 26PernambucoGlória do Goitá | | 260610 | 2606101 | 29957.9 | 5020 | 3934.1 | 4094.9 | 3213 | 5536 |  |
| 1526PE | 26PernambucoGoiana | | 260620 | 2606200 | 77978.5 | 13066.9 | 10240.1 | 10658.6 | 8363.1 | 12983 |  |
| 1527PE | 26PernambucoGranito | | 260630 | 2606309 | 7218.6 | 1209.6 | 947.9 | 986.7 | 774.2 | 550 |  |
| 1528PE | 26PernambucoGravatá | | 260640 | 2606408 | 80832.2 | 13545.1 | 10614.9 | 11048.7 | 8669.2 | 14016 |  |
| 1529PE | 26PernambucoIati | | 260650 | 2606507 | 18862.3 | 3160.8 | 2477 | 2578.2 | 2023 | 1424 |  |
| 1530PE | 26PernambucoIbimirim | | 260660 | 2606606 | 28273.5 | 4737.8 | 3712.9 | 3864.6 | 3032.3 | 5378 |  |
| 1531PE | 26PernambucoIbirajuba | | 260670 | 2606705 | 7676.5 | 1286.4 | 1008.1 | 1049.3 | 823.3 | 1257 |  |
| 1532PE | 26PernambucoIgarassu | | 260680 | 2606804 | 110527.5 | 18521.1 | 14514.5 | 15107.7 | 11854 | 21600 |  |
| 1533PE | 26PernambucoIguaraci | | 260690 | 2606903 | 12052.3 | 2019.6 | 1582.7 | 1647.4 | 1292.6 | 3736 |  |
| 1534PE | 26PernambucoInajá | | 260700 | 2607000 | 21423.6 | 3590 | 2813.3 | 2928.3 | 2297.7 | 1691 |  |
| 1535PE | 26PernambucoIngazeira | | 260710 | 2607109 | 4535.5 | 760 | 595.6 | 619.9 | 486.4 | 2520 |  |
| 1536PE | 26PernambucoIpojuca | | 260720 | 2607208 | 89411.3 | 14982.7 | 11741.5 | 12221.3 | 9589.3 | 16285 |  |
| 1537PE | 26PernambucoIpubi | | 260730 | 2607307 | 29651.7 | 4968.7 | 3893.9 | 4053 | 3180.1 | 1973 |  |
| 1538PE | 26PernambucoItacuruba | | 260740 | 2607406 | 4681.6 | 784.5 | 614.8 | 639.9 | 502.1 | 1065 |  |
| 1539PE | 26PernambucoItaíba | | 260750 | 2607505 | 26358.5 | 4416.9 | 3461.4 | 3602.9 | 2826.9 | 2463 |  |
| 1540PE | 26PernambucoIlha de Itamaracá | | 260760 | 2607604 | 24347.8 | 4080 | 3197.4 | 3328 | 2611.3 | 4074 |  |
| 1541PE | 26PernambucoItambé | | 260765 | 2607653 | 36054.4 | 6041.6 | 4734.7 | 4928.2 | 3866.8 | 4230 |  |
| 1542PE | 26PernambucoItapetim | | 260770 | 2607703 | 13765.1 | 2306.6 | 1807.6 | 1881.5 | 1476.3 | 5368 |  |
| 1543PE | 26PernambucoItapissuma | | 260775 | 2607752 | 25413 | 4258.5 | 3337.2 | 3473.6 | 2725.5 | 6237 |  |
| 1544PE | 26PernambucoItaquitinga | | 260780 | 2607802 | 16527.2 | 2769.5 | 2170.4 | 2259.1 | 1772.5 | 2501 |  |
| 1545PE | Jaboatão dos  26PernambucoGuararapes | | 260790 | 2607901 | 677887.2 | 113593.6 | 89020.1 | 92658.3 | 72702.8 | 194844 | 67 |

1

4

1

9

4

1

1

2

1

5

1

4

2

1

1

2

1

1

2

1

1

1

1

1

8

1

3

4

1

5

6

1

1

2

| 1546PE | 26PernambucoJaqueira | 260795 | 2607950 | 11611.6 | 1945.8 | 1524.8 | 1587.2 | 1245.3 | 396 |  |
| --- | --- | --- | --- | --- | --- | --- | --- | --- | --- | --- |
| 1547PE | 26PernambucoJataúba | 260800 | 2608008 | 16644.2 | 2789.1 | 2185.7 | 2275 | 1785.1 | 1556 |  |
| 1548PE | 26PernambucoJatobá | 260805 | 2608057 | 14453.8 | 2422 | 1898.1 | 1975.6 | 1550.2 | 2557 |  |
| 1549PE | 26PernambucoJoão Alfredo | 260810 | 2608107 | 32510.9 | 5447.9 | 4269.3 | 4443.8 | 3486.8 | 5131 |  |
| 1550PE | 26PernambucoJoaquim Nabuco | 260820 | 2608206 | 15945.2 | 2671.9 | 2093.9 | 2179.5 | 1710.1 | 1414 |  |
| 1551PE | 26PernambucoJucati | 260825 | 2608255 | 11080.4 | 1856.7 | 1455.1 | 1514.5 | 1188.4 | 2048 |  |
| 1552PE | 26PernambucoJupi | 260830 | 2608305 | 14360 | 2406.3 | 1885.8 | 1962.8 | 1540.1 | 2804 |  |
| 1553PE | 26PernambucoJurema | 260840 | 2608404 | 15035.2 | 2519.4 | 1974.4 | 2055.1 | 1612.5 | 1419 |  |
| 1554PE | 26PernambucoLagoa do Carro | 260845 | 2608453 | 17181.8 | 2879.2 | 2256.3 | 2348.5 | 1842.7 | 3829 |  |
| 1555PE | 26PernambucoLagoa do Ouro | 260860 | 2608602 | 12718.1 | 2131.2 | 1670.1 | 1738.4 | 1364 | 3025 |  |
| 1556PE | 26PernambucoLagoa dos Gatos | 260870 | 2608701 | 16034.6 | 2686.9 | 2105.7 | 2191.7 | 1719.7 | 838 |  |
| 1557PE | 26PernambucoLagoa Grande | 260875 | 2608750 | 24379.5 | 4085.3 | 3201.5 | 3332.4 | 2614.7 | 1539 |  |
| 1558PE | 26PernambucoLajedo | 260880 | 2608800 | 38730.2 | 6490 | 5086 | 5293.9 | 4153.8 | 6266 |  |
| 1559PE | 26PernambucoLimoeiro | 260890 | 2608909 | 56007.9 | 9385.2 | 7355 | 7655.5 | 6006.8 | 16266 |  |
| 1560PE | 26PernambucoMacaparana | 260900 | 2609006 | 24779.2 | 4152.3 | 3254 | 3387 | 2657.5 | 4679 |  |
| 1561PE | 26PernambucoMachados | 260910 | 2609105 | 15002.1 | 2513.9 | 1970.1 | 2050.6 | 1609 | 2202 |  |
| 1562PE | 26PernambucoManari | 260915 | 2609154 | 19964.8 | 3345.5 | 2621.8 | 2728.9 | 2141.2 | 1415 |  |
| 1563PE | 26PernambucoMaraial | 260920 | 2609204 | 11768.3 | 1972 | 1545.4 | 1608.6 | 1262.1 | 378 |  |
| 1564PE | 26PernambucoMirandiba | 260930 | 2609303 | 14936.9 | 2503 | 1961.5 | 2041.7 | 1602 | 1229 |  |
| 1565PE | 26PernambucoMoreno | 260940 | 2609402 | 60182.5 | 10084.8 | 7903.2 | 8226.2 | 6454.5 | 13274 |  |
| 1566PE | 26PernambucoNazaré da Mata | 260950 | 2609501 | 31787.9 | 5326.7 | 4174.4 | 4345 | 3409.2 | 4509 |  |
| 1567PE | 26PernambucoOlinda | 260960 | 2609600 | 386726.1 | 64803.7 | 50784.9 | 52860.4 | 41476 | 121087 | 42 |
| 1568PE | 26PernambucoOrobó | 260970 | 2609709 | 23483.7 | 3935.2 | 3083.9 | 3209.9 | 2518.6 | 4269 |  |
| 1569PE | 26PernambucoOrocó | 260980 | 2609808 | 14208.9 | 2381 | 1865.9 | 1942.2 | 1523.9 | 644 |  |
| 1570PE | 26PernambucoOuricuri | 260990 | 2609907 | 67268.4 | 11272.2 | 8833.7 | 9194.7 | 7214.5 | 2951 |  |
| 1571PE | 26PernambucoPalmares | 261000 | 2610004 | 61712.8 | 10341.2 | 8104.1 | 8435.3 | 6618.6 | 9063 |  |
| 1572PE | 26PernambucoPalmeirina | 261010 | 2610103 | 7979.2 | 1337.1 | 1047.8 | 1090.7 | 855.8 | 1072 |  |
| 1573PE | 26PernambucoPanelas | 261020 | 2610202 | 26256.9 | 4399.9 | 3448.1 | 3589 | 2816 | 3367 |  |
| 1574PE | 26PernambucoParanatama | 261030 | 2610301 | 11321 | 1897.1 | 1486.7 | 1547.4 | 1214.2 | 2154 |  |
| 1575PE | 26PernambucoParnamirim | 261040 | 2610400 | 21084.8 | 3533.2 | 2768.9 | 2882 | 2261.3 | 2171 |  |
| 1576PE | 26PernambucoPassira | 261050 | 2610509 | 28859.3 | 4836 | 3789.8 | 3944.7 | 3095.1 | 7423 |  |
| 1577PE | 26PernambucoPaudalho | 261060 | 2610608 | 54312.9 | 9101.2 | 7132.4 | 7423.9 | 5825 | 10623 |  |
| 1578PE | 26PernambucoPaulista | 261070 | 2610707 | 318415.2 | 53356.8 | 41814.3 | 43523.2 | 34149.7 | 80354 | 27 |
| 1579PE | 26PernambucoPedra | 261080 | 2610806 | 21870.3 | 3664.8 | 2872 | 2989.4 | 2345.6 | 3094 |  |
| 1580PE | 26PernambucoPesqueira | 261090 | 2610905 | 65491.3 | 10974.4 | 8600.3 | 8951.8 | 7023.9 | 12101 |  |
| 1581PE | 26PernambucoPetrolândia | 261100 | 2611002 | 34803.2 | 5832 | 4570.4 | 4757.1 | 3732.6 | 8838 |  |
| 1582PE | 26PernambucoPetrolina | 261110 | 2611101 | 325083.8 | 54474.3 | 42690 | 44434.7 | 34864.9 | 49432 | 19 |
| 1583PE | 26PernambucoPoção | 261120 | 2611200 | 11244.2 | 1884.2 | 1476.6 | 1536.9 | 1205.9 | 879 |  |
| 1584PE | 26PernambucoPombos | 261130 | 2611309 | 26255.3 | 4399.6 | 3447.8 | 3588.8 | 2815.9 | 5667 |  |
| 1585PE | 26PernambucoPrimavera | 261140 | 2611408 | 14305.3 | 2397.1 | 1878.6 | 1955.3 | 1534.2 | 1584 |  |
| 1586PE | 26PernambucoQuipapá | 261150 | 2611507 | 25261.1 | 4233 | 3317.3 | 3452.9 | 2709.2 | 819 |  |
| 1587PE | 26PernambucoQuixaba | 261153 | 2611533 | 6794.4 | 1138.5 | 892.2 | 928.7 | 728.7 | 4495 |  |
| 1588PE | 26PernambucoRecife | 261160 | 2611606 | 1600728.4 | 268234.1 | 210207.7 | 218798.5 | 171676.6 | 425588 | 120 |
| 1589PE | 26PernambucoRiacho das Almas | 261170 | 2611705 | 19968 | 3346 | 2622.2 | 2729.4 | 2141.5 | 5939 |  |
| 1590PE | 26PernambucoRibeirão | 261180 | 2611804 | 46193.9 | 7740.7 | 6066.2 | 6314.1 | 4954.3 | 4507 |  |
| 1591PE | 26PernambucoRio Formoso | 261190 | 2611903 | 22963.4 | 3848 | 3015.6 | 3138.8 | 2462.8 | 2976 |  |
| 1592PE | 26PernambucoSairé | 261200 | 2612000 | 10542.4 | 1766.6 | 1384.4 | 1441 | 1130.7 | 1384 |  |
| 1593PE | 26PernambucoSalgadinho | 261210 | 2612109 | 10220.5 | 1712.6 | 1342.2 | 1397 | 1096.1 | 1391 |  |
| 1594PE | 26PernambucoSalgueiro | 261220 | 2612208 | 59128.3 | 9908.1 | 7764.7 | 8082.1 | 6341.5 | 7562 |  |
| 1595PE | 26PernambucoSaloá | 261230 | 2612307 | 15636.8 | 2620.3 | 2053.4 | 2137.3 | 1677 | 2356 |  |
| 1596PE | 26PernambucoSanharó | 261240 | 2612406 | 24492.4 | 4104.2 | 3216.3 | 3347.8 | 2626.8 | 2975 |  |
| 1597PE | 26PernambucoSanta Cruz | 261245 | 2612455 | 14620 | 2449.9 | 1919.9 | 1998.4 | 1568 | 1532 |  |
| 1598PE | Santa Cruz da  26PernambucoBaixa Verde | 261247 | 2612471 | 12249 | 2052.6 | 1608.5 | 1674.3 | 1313.7 | 2476 |  |
| 1599PE | Santa Cruz do  26PernambucoCapibaribe | 261250 | 2612505 | 99016.4 | 16592.2 | 13002.8 | 13534.2 | 10619.4 | 5987 |  |
| 1600PE | 26PernambucoSanta Filomena | 261255 | 2612554 | 14011.1 | 2347.8 | 1839.9 | 1915.1 | 1502.7 | 1095 |  |
| 1601PE | Santa Maria da  26PernambucoBoa Vista | 261260 | 2612604 | 40900.1 | 6853.6 | 5371 | 5590.5 | 4386.5 | 2306 |  |
| 1602PE | Santa Maria do  26PernambucoCambucá | 261270 | 2612703 | 13665.4 | 2289.9 | 1794.5 | 1867.9 | 1465.6 | 2577 |  |
| 1603PE | 26PernambucoSanta Terezinha | 261280 | 2612802 | 11474.6 | 1922.8 | 1506.8 | 1568.4 | 1230.6 | 3375 |  |
| 1604PE | São Benedito do  26PernambucoSul | 261290 | 2612901 | 15050.6 | 2522 | 1976.4 | 2057.2 | 1614.2 | 347 |  |
| 1605PE | 26PernambucoSão Bento do Una | 261300 | 2613008 | 56817.5 | 9520.9 | 7461.3 | 7766.2 | 6093.6 | 10289 |  |
| 1606PE | 26PernambucoSão Caitano | 261310 | 2613107 | 36440 | 6106.3 | 4785.3 | 4980.9 | 3908.2 | 8593 |  |
| 1607PE | 26PernambucoSão João | 261320 | 2613206 | 22176.7 | 3716.1 | 2912.2 | 3031.3 | 2378.4 | 4600 |  |
| 1608PE | São Joaquim do  26PernambucoMonte | 261330 | 2613305 | 21013 | 3521.1 | 2759.4 | 2872.2 | 2253.6 | 3136 |  |
| 1609PE | São José da 26PernambucoCoroa Grande | 261340 | 2613404 | 19942.1 | 3341.7 | 2618.8 | 2725.8 | 2138.8 | 1802 |  |
| 1610PE | São José do  26PernambucoBelmonte | 261350 | 2613503 | 33429.9 | 5601.8 | 4390 | 4569.4 | 3585.3 | 3578 |  |
| 1611PE | 26PernambucoSão José do Egito | 261360 | 2613602 | 33066.4 | 5540.9 | 4342.3 | 4519.7 | 3546.3 | 8788 |  |
| 1612PE | São Lourenço da  26PernambucoMata | 261370 | 2613701 | 108828.2 | 18236.3 | 14291.3 | 14875.4 | 11671.7 | 14425 |  |
| 1613PE | 26PernambucoSão Vicente Ferrer | 261380 | 2613800 | 17599.6 | 2949.2 | 2311.2 | 2405.6 | 1887.5 | 3144 |  |
| 1614PE | 26PernambucoSerra Talhada | 261390 | 2613909 | 83336.9 | 13964.8 | 10943.8 | 11391.1 | 8937.8 | 22378 |  |
| 1615PE | 26PernambucoSerrita | 261400 | 2614006 | 18859.8 | 3160.3 | 2476.7 | 2577.9 | 2022.7 | 1940 |  |
| 1616PE | 26PernambucoSertânia | 261410 | 2614105 | 35032.1 | 5870.3 | 4600.4 | 4788.4 | 3757.2 | 6372 |  |
| 1617PE | 26PernambucoSirinhaém | 261420 | 2614204 | 43460.3 | 7282.6 | 5707.2 | 5940.5 | 4661.1 | 4269 |  |
| 1618PE | 26PernambucoMoreilândia | 261430 | 2614303 | 11203.6 | 1877.4 | 1471.3 | 1531.4 | 1201.6 | 738 |  |
| 1619PE | 26PernambucoSolidão | 261440 | 2614402 | 5902.4 | 989.1 | 775.1 | 806.8 | 633 | 2294 |  |
| 1620PE | 26PernambucoSurubim | 261450 | 2614501 | 62274.2 | 10435.3 | 8177.8 | 8512.1 | 6678.8 | 17698 |  |
| 1621PE | 26PernambucoTabira | 261460 | 2614600 | 27648.4 | 4633 | 3630.8 | 3779.2 | 2965.3 | 9530 |  |
| 1622PE | 26PernambucoTacaimbó | 261470 | 2614709 | 12835.3 | 2150.8 | 1685.5 | 1754.4 | 1376.6 | 928 |  |
| 1623PE | 26PernambucoTacaratu | 261480 | 2614808 | 24158.5 | 4048.2 | 3172.5 | 3302.1 | 2591 | 3215 |  |
| 1624PE | 26PernambucoTamandaré | 261485 | 2614857 | 22237.5 | 3726.3 | 2920.2 | 3039.6 | 2385 | 3405 |  |
| 1625PE | Taquaritinga do  26PernambucoNorte | 261500 | 2615003 | 27096.4 | 4540.5 | 3558.3 | 3703.7 | 2906.1 | 3194 |  |
| 1626PE | 26PernambucoTerezinha | 261510 | 2615102 | 6990.1 | 1171.3 | 917.9 | 955.5 | 749.7 | 1041 |  |
| 1627PE | 26PernambucoTerra Nova | 261520 | 2615201 | 9920.2 | 1662.3 | 1302.7 | 1356 | 1063.9 | 1505 |  |
| 1628PE | 26PernambucoTimbaúba | 261530 | 2615300 | 53270.3 | 8926.5 | 6995.5 | 7281.3 | 5713.2 | 11602 |  |
| 1629PE | 26PernambucoToritama | 261540 | 2615409 | 40970.2 | 6865.4 | 5380.2 | 5600.1 | 4394 | 1833 |  |
| 1630PE | 26PernambucoTracunhaém | 261550 | 2615508 | 13478.1 | 2258.5 | 1769.9 | 1842.3 | 1445.5 | 1830 |  |
| 1631PE | 26PernambucoTrindade | 261560 | 2615607 | 28649.9 | 4800.9 | 3762.3 | 3916.1 | 3072.7 | 2029 |  |
| 1632PE | 26PernambucoTriunfo | 261570 | 2615706 | 15175.8 | 2543 | 1992.9 | 2074.3 | 1627.6 | 3462 |  |
| 1633PE | 26PernambucoTupanatinga | 261580 | 2615805 | 26068.2 | 4368.2 | 3423.3 | 3563.2 | 2795.8 | 2547 |  |
| 1634PE | 26PernambucoTuparetama | 261590 | 2615904 | 8095.1 | 1356.5 | 1063 | 1106.5 | 868.2 | 2723 |  |
| 1635PE | 26PernambucoVenturosa | 261600 | 2616001 | 17522.2 | 2936.2 | 2301 | 2395.1 | 1879.2 | 3338 |  |
| 1636PE | 26PernambucoVerdejante | 261610 | 2616100 | 9379.1 | 1571.7 | 1231.7 | 1282 | 1005.9 | 1707 |  |
| 1637PE | 26PernambucoVertente do Lério | 261618 | 2616183 | 7749.3 | 1298.6 | 1017.6 | 1059.2 | 831.1 | 1311 |  |
| 1638PE | 26PernambucoVertentes | 261620 | 2616209 | 19648 | 3292.4 | 2580.2 | 2685.6 | 2107.2 | 3390 |  |
| 1639PE | 26PernambucoVicência | 261630 | 2616308 | 31855 | 5337.9 | 4183.2 | 4354.2 | 3416.4 | 4411 |  |
| 1640PE | Vitória de Santo  26PernambucoAntão | 261640 | 2616407 | 134634.3 | 22560.7 | 17680.2 | 18402.7 | 14439.4 | 39470 | 12 |
| 1641PE | 26PernambucoXexéu | 261650 | 2616506 | 14472.3 | 2425.1 | 1900.5 | 1978.2 | 1552.1 | 940 |  |
| 1642AL | 27Alagoas Água Branca | 270010 | 2700102 | 20139.9 | 2999 | 2489.7 | 2517.7 | 2113.5 | 2992 |  |
| 1643AL | 27Alagoas Anadia | 270020 | 2700201 | 17674.2 | 2631.9 | 2184.9 | 2209.5 | 1854.7 | 3714 |  |
| 1644AL | 27Alagoas Arapiraca | 270030 | 2700300 | 226529.5 | 33732.5 | 28003.6 | 28318.4 | 23772.2 | 67724 | 22 |
| 1645AL | 27Alagoas Atalaia | 270040 | 2700409 | 46441.9 | 6915.7 | 5741.1 | 5805.7 | 4873.7 | 4998 |  |
| 1646AL | Barra de Santo  27Alagoas Antônio | 270050 | 2700508 | 15372.7 | 2289.1 | 1900.4 | 1921.7 | 1613.2 | 2373 |  |

1

1

1

3

3

2

1

1

3

3

3

1

1

1

1

2

1

2

3

1

1

1

5

8

2

1

4

2

1

2

1

1

1

1

1

1

| 1647AL | 27Alagoas | Barra de São  Miguel | 270060 | 2700607 | 8088.7 | 1204.5 | 999.9 | 1011.2 | 848.8 | 1786 |  |
| --- | --- | --- | --- | --- | --- | --- | --- | --- | --- | --- | --- |
| 1648AL | 27Alagoas | Batalha | 270070 | 2700706 | 18087.2 | 2693.4 | 2235.9 | 2261.1 | 1898.1 | 2656 |  |
| 1649AL | 27Alagoas | Belém | 270080 | 2700805 | 4533.3 | 675.1 | 560.4 | 566.7 | 475.7 | 1056 |  |
| 1650AL | 27Alagoas | Belo Monte | 270090 | 2700904 | 6784.3 | 1010.3 | 838.7 | 848.1 | 712 | 807 |  |
| 1651AL | 27Alagoas | Boca da Mata | 270100 | 2701001 | 26887.6 | 4003.8 | 3323.8 | 3361.2 | 2821.6 | 5871 |  |
| 1652AL | 27Alagoas | Branquinha | 270110 | 2701100 | 10633.8 | 1583.5 | 1314.6 | 1329.3 | 1115.9 | 2303 |  |
| 1653AL | 27Alagoas | Cacimbinhas | 270120 | 2701209 | 10641.2 | 1584.6 | 1315.5 | 1330.3 | 1116.7 | 1715 |  |
| 1654AL | 27Alagoas | Cajueiro | 270130 | 2701308 | 21107.7 | 3143.1 | 2609.3 | 2638.7 | 2215.1 | 2206 |  |
| 1655AL | 27Alagoas | Campestre | 270135 | 2701357 | 6861.5 | 1021.7 | 848.2 | 857.8 | 720.1 | 946 |  |
| 1656AL | 27Alagoas | Campo Alegre | 270140 | 2701407 | 55033 | 8195 | 6803.2 | 6879.7 | 5775.2 | 6433 |  |
| 1657AL | 27Alagoas | Campo Grande | 270150 | 2701506 | 9476.9 | 1411.2 | 1171.5 | 1184.7 | 994.5 | 995 |  |
| 1658AL | 27Alagoas | Canapi | 270160 | 2701605 | 17697.2 | 2635.3 | 2187.7 | 2212.3 | 1857.2 | 1895 |  |
| 1659AL | 27Alagoas | Capela | 270170 | 2701704 | 17208.1 | 2562.5 | 2127.3 | 2151.2 | 1805.8 | 2363 |  |
| 1660AL | 27Alagoas | Carneiros | 270180 | 2701803 | 8815.4 | 1312.7 | 1089.8 | 1102 | 925.1 | 909 |  |
| 1661AL | 27Alagoas | Chã Preta | 270190 | 2701902 | 7317 | 1089.6 | 904.5 | 914.7 | 767.9 | 849 |  |
| 1662AL | 27Alagoas | Coité do Nóia | 270200 | 2702009 | 10895 | 1622.4 | 1346.8 | 1362 | 1143.3 | 2901 |  |
| 1663AL | 27Alagoas | Colônia  Leopoldina | 270210 | 2702108 | 21203.8 | 3157.5 | 2621.2 | 2650.7 | 2225.1 | 1451 |  |
| 1664AL | 27Alagoas | Coqueiro Seco | 270220 | 2702207 | 5768.3 | 859 | 713.1 | 721.1 | 605.3 | 1416 |  |
| 1665AL | 27Alagoas | Coruripe | 270230 | 2702306 | 55457.4 | 8258.2 | 6855.6 | 6932.7 | 5819.8 | 6902 |  |
| 1666AL | 27Alagoas | Craíbas | 270235 | 2702355 | 23794.5 | 3543.2 | 2941.5 | 2974.5 | 2497 | 3438 |  |
| 1667AL | 27Alagoas | Delmiro Gouveia | 270240 | 2702405 | 50849.1 | 7571.9 | 6286 | 6356.6 | 5336.2 | 13253 |  |
| 1668AL | 27Alagoas | Dois Riachos | 270250 | 2702504 | 11083.3 | 1650.4 | 1370.1 | 1385.5 | 1163.1 | 1289 |  |
| 1669AL | 27Alagoas | Estrela de Alagoas | 270255 | 2702553 | 17978.6 | 2677.2 | 2222.5 | 2247.5 | 1886.7 | 3291 |  |
| 1670AL | 27Alagoas | Feira Grande | 270260 | 2702603 | 22047.3 | 3283.1 | 2725.5 | 2756.1 | 2313.7 | 3372 |  |
| 1671AL | 27Alagoas | Feliz Deserto | 270270 | 2702702 | 4641.5 | 691.2 | 573.8 | 580.2 | 487.1 | 1110 |  |
| 1672AL | 27Alagoas | Flexeiras | 270280 | 2702801 | 12719.6 | 1894.1 | 1572.4 | 1590.1 | 1334.8 | 2501 |  |
| 1673AL | 27Alagoas | Girau do Ponciano | 270290 | 2702900 | 39554.8 | 5890.1 | 4889.8 | 4944.7 | 4150.9 | 3338 |  |
| 1674AL | 27Alagoas | Ibateguara | 270300 | 2703007 | 15572.6 | 2318.9 | 1925.1 | 1946.7 | 1634.2 | 1723 |  |
| 1675AL | 27Alagoas | Igaci | 270310 | 2703106 | 25696 | 3826.4 | 3176.5 | 3212.3 | 2696.6 | 4168 |  |
| 1676AL | 27Alagoas | Igreja Nova | 270320 | 2703205 | 24204 | 3604.2 | 2992.1 | 3025.7 | 2540 | 2417 |  |
| 1677AL | 27Alagoas | Inhapi | 270330 | 2703304 | 18336.9 | 2730.5 | 2266.8 | 2292.3 | 1924.3 | 2210 |  |
| 1678AL | 27Alagoas | Jacaré dos  Homens | 270340 | 2703403 | 5381.9 | 801.4 | 665.3 | 672.8 | 564.8 | 1067 |  |
| 1679AL | 27Alagoas | Jacuípe | 270350 | 2703502 | 7079.2 | 1054.2 | 875.1 | 885 | 742.9 | 512 |  |
| 1680AL | 27Alagoas | Japaratinga | 270360 | 2703601 | 8187.6 | 1219.2 | 1012.2 | 1023.5 | 859.2 | 870 |  |
| 1681AL | 27Alagoas | Jaramataia | 270370 | 2703700 | 5646.9 | 840.9 | 698.1 | 705.9 | 592.6 | 1077 |  |
| 1682AL | 27Alagoas | Jequiá da Praia | 270375 | 2703759 | 11843.8 | 1763.7 | 1464.1 | 1480.6 | 1242.9 | 2235 |  |
| 1683AL | 27Alagoas | Joaquim Gomes | 270380 | 2703809 | 23630.9 | 3518.9 | 2921.3 | 2954.1 | 2479.9 | 3386 |  |
| 1684AL | 27Alagoas | Jundiá | 270390 | 2703908 | 4212.2 | 627.2 | 520.7 | 526.6 | 442 | 661 |  |
| 1685AL | 27Alagoas | Junqueiro | 270400 | 2704005 | 24670.6 | 3673.7 | 3049.8 | 3084.1 | 2589 | 3566 |  |
| 1686AL | 27Alagoas | Lagoa da Canoa | 270410 | 2704104 | 18191.8 | 2708.9 | 2248.9 | 2274.2 | 1909.1 | 3462 |  |
| 1687AL | 27Alagoas | Limoeiro de  Anadia | 270420 | 2704203 | 28145.6 | 4191.2 | 3479.4 | 3518.5 | 2953.6 | 2895 |  |
| 1688AL | 27Alagoas | Maceió | 270430 | 2704302 | 992724.3 | 147826.6 | 122720.6 | 124100.4 | 104177.5 | 259875 | 100 |
| 1689AL | 27Alagoas | Major Isidoro | 270440 | 2704401 | 19688.3 | 2931.8 | 2433.9 | 2461.2 | 2066.1 | 2790 |  |
| 1690AL | 27Alagoas | Maragogi | 270450 | 2704500 | 31362.2 | 4670.1 | 3877 | 3920.6 | 3291.2 | 2417 |  |
| 1691AL | 27Alagoas | Maravilha | 270460 | 2704609 | 9768.3 | 1454.6 | 1207.6 | 1221.1 | 1025.1 | 1517 |  |
| 1692AL | 27Alagoas | Marechal Deodoro | 270470 | 2704708 | 49897.1 | 7430.2 | 6168.3 | 6237.6 | 5236.3 | 11522 |  |
| 1693AL | 27Alagoas | Maribondo | 270480 | 2704807 | 13540.3 | 2016.3 | 1673.9 | 1692.7 | 1420.9 | 2961 |  |
| 1694AL | 27Alagoas | Mar Vermelho | 270490 | 2704906 | 3608 | 537.3 | 446 | 451 | 378.6 | 973 |  |
| 1695AL | 27Alagoas | Mata Grande | 270500 | 2705002 | 25203.7 | 3753.1 | 3115.7 | 3150.7 | 2644.9 | 2974 |  |
| 1696AL | 27Alagoas | Matriz de  Camaragibe | 270510 | 2705101 | 24563.4 | 3657.7 | 3036.5 | 3070.7 | 2577.7 | 3932 |  |
| 1697AL | 27Alagoas | Messias | 270520 | 2705200 | 17128.4 | 2550.6 | 2117.4 | 2141.2 | 1797.5 | 3544 |  |
| 1698AL | 27Alagoas | Minador do  Negrão | 270530 | 2705309 | 5358.3 | 797.9 | 662.4 | 669.8 | 562.3 | 652 |  |
| 1699AL | 27Alagoas | Monteirópolis | 270540 | 2705408 | 7134.4 | 1062.4 | 882 | 891.9 | 748.7 | 693 |  |
| 1700AL | 27Alagoas | Murici | 270550 | 2705507 | 27871.5 | 4150.3 | 3445.5 | 3484.2 | 2924.9 | 1427 |  |
| 1701AL | 27Alagoas | Novo Lino | 270560 | 2705606 | 12511.2 | 1863 | 1546.6 | 1564 | 1312.9 | 2052 |  |
| 1702AL | 27Alagoas | Olho d'Água das  Flores | 270570 | 2705705 | 21257.1 | 3165.4 | 2627.8 | 2657.3 | 2230.7 | 2637 |  |
| 1703AL | 27Alagoas | Olho d'Água do  Casado | 270580 | 2705804 | 9093.7 | 1354.1 | 1124.2 | 1136.8 | 954.3 | 1246 |  |
| 1704AL | 27Alagoas | Olho d'Água  Grande | 270590 | 2705903 | 5101.7 | 759.7 | 630.7 | 637.8 | 535.4 | 545 |  |
| 1705AL | 27Alagoas | Olivença | 270600 | 2706000 | 11493.9 | 1711.6 | 1420.9 | 1436.9 | 1206.2 | 1244 |  |
| 1706AL | 27Alagoas | Ouro Branco | 270610 | 2706109 | 11344.7 | 1689.3 | 1402.4 | 1418.2 | 1190.5 | 1635 |  |
| 1707AL | 27Alagoas | Palestina | 270620 | 2706208 | 5047.6 | 751.6 | 624 | 631 | 529.7 | 347 |  |
| 1708AL | 27Alagoas | Palmeira dos  Índios | 270630 | 2706307 | 72736.8 | 10831.2 | 8991.7 | 9092.8 | 7633.1 | 18342 |  |
| 1709AL | 27Alagoas | Pão de Açúcar | 270640 | 2706406 | 24443.9 | 3639.9 | 3021.8 | 3055.7 | 2565.2 | 2284 |  |
| 1710AL | 27Alagoas | Pariconha | 270642 | 2706422 | 10520 | 1566.5 | 1300.5 | 1315.1 | 1104 | 3283 |  |
| 1711AL | 27Alagoas | Paripueira | 270644 | 2706448 | 12536 | 1866.7 | 1549.7 | 1567.1 | 1315.5 | 1805 |  |
| 1712AL | 27Alagoas | Passo de  Camaragibe | 270650 | 2706505 | 15191.4 | 2262.2 | 1878 | 1899.1 | 1594.2 | 2514 |  |
| 1713AL | 27Alagoas | Paulo Jacinto | 270660 | 2706604 | 7579.8 | 1128.7 | 937 | 947.6 | 795.4 | 2206 |  |
| 1714AL | 27Alagoas | Penedo | 270670 | 2706703 | 62940.5 | 9372.5 | 7780.7 | 7868.2 | 6605 | 10528 |  |
| 1715AL | 27Alagoas | Piaçabuçu | 270680 | 2706802 | 17738.5 | 2641.4 | 2192.8 | 2217.5 | 1861.5 | 3301 |  |
| 1716AL | 27Alagoas | Pilar | 270690 | 2706901 | 34694.4 | 5166.3 | 4288.9 | 4337.1 | 3640.9 | 9286 |  |
| 1717AL | 27Alagoas | Pindoba | 270700 | 2707008 | 2918.5 | 434.6 | 360.8 | 364.8 | 306.3 | 1122 |  |
| 1718AL | 27Alagoas | Piranhas | 270710 | 2707107 | 24445 | 3640.1 | 3021.9 | 3055.9 | 2565.3 | 2861 |  |
| 1719AL | 27Alagoas | Poço das  Trincheiras | 270720 | 2707206 | 14283.2 | 2126.9 | 1765.7 | 1785.5 | 1498.9 | 1158 |  |
| 1720AL | 27Alagoas | Porto Calvo | 270730 | 2707305 | 26817.4 | 3993.4 | 3315.2 | 3352.4 | 2814.2 | 4807 |  |
| 1721AL | 27Alagoas | Porto de Pedras | 270740 | 2707404 | 8132 | 1210.9 | 1005.3 | 1016.6 | 853.4 | 1073 |  |
| 1722AL | 27Alagoas | Porto Real do  Colégio | 270750 | 2707503 | 19911.9 | 2965.1 | 2461.5 | 2489.2 | 2089.6 | 1120 |  |
| 1723AL | 27Alagoas | Quebrangulo | 270760 | 2707602 | 11482.4 | 1709.8 | 1419.5 | 1435.4 | 1205 | 1632 |  |
| 1724AL | 27Alagoas | Rio Largo | 270770 | 2707701 | 73072.9 | 10881.3 | 9033.3 | 9134.8 | 7668.3 | 15042 |  |
| 1725AL | 27Alagoas | Roteiro | 270780 | 2707800 | 6725.8 | 1001.5 | 831.4 | 840.8 | 705.8 | 1240 |  |
| 1726AL | 27Alagoas | Santa Luzia do  Norte | 270790 | 2707909 | 7197.3 | 1071.7 | 889.7 | 899.7 | 755.3 | 1578 |  |
| 1727AL | 27Alagoas | Santana do  Ipanema | 270800 | 2708006 | 46975.2 | 6995.1 | 5807.1 | 5872.4 | 4929.6 | 8950 |  |
| 1728AL | 27Alagoas | Santana do  Mundaú | 270810 | 2708105 | 10918.7 | 1625.9 | 1349.8 | 1364.9 | 1145.8 | 1493 |  |
| 1729AL | 27Alagoas | São Brás | 270820 | 2708204 | 6927.1 | 1031.5 | 856.3 | 866 | 726.9 | 569 |  |
| 1730AL | 27Alagoas | São José da Laje | 270830 | 2708303 | 23638.3 | 3520 | 2922.2 | 2955 | 2480.6 | 3172 |  |
| 1731AL | 27Alagoas | São José da  Tapera | 270840 | 2708402 | 31662.4 | 4714.8 | 3914.1 | 3958.1 | 3322.7 | 3545 |  |
| 1732AL | 27Alagoas | São Luís do  Quitunde | 270850 | 2708501 | 33991.7 | 5061.7 | 4202.1 | 4249.3 | 3567.1 | 3453 |  |
| 1733AL | 27Alagoas | São Miguel dos  Campos | 270860 | 2708600 | 59086.1 | 8798.5 | 7304.2 | 7386.4 | 6200.6 | 12381 |  |
| 1734AL | 27Alagoas | São Miguel dos  Milagres | 270870 | 2708709 | 7697.8 | 1146.3 | 951.6 | 962.3 | 807.8 | 1448 |  |
| 1735AL | 27Alagoas | São Sebastião | 270880 | 2708808 | 33585 | 5001.1 | 4151.8 | 4198.5 | 3524.4 | 3237 |  |
| 1736AL | 27Alagoas | Satuba | 270890 | 2708907 | 14211.4 | 2116.2 | 1756.8 | 1776.6 | 1491.4 | 3810 |  |
| 1737AL | 27Alagoas | Senador Rui  Palmeira | 270895 | 2708956 | 13659.6 | 2034.1 | 1688.6 | 1707.6 | 1433.5 | 1289 |  |
| 1738AL | 27Alagoas | Tanque d'Arca | 270900 | 2709004 | 6233.1 | 928.2 | 770.5 | 779.2 | 654.1 | 1210 |  |
| 1739AL | 27Alagoas | Taquarana | 270910 | 2709103 | 19682.8 | 2931 | 2433.2 | 2460.5 | 2065.5 | 3590 |  |
| 1740AL | 27Alagoas | Teotônio Vilela | 270915 | 2709152 | 43331.9 | 6452.6 | 5356.7 | 5416.9 | 4547.3 | 10641 |  |
| 1741AL | 27Alagoas | Traipu | 270920 | 2709202 | 27223.9 | 4053.9 | 3365.4 | 3403.3 | 2856.9 | 3473 |  |

2

2

1

4

1

1

1

1

1

1

1

1

4

1

1

1

1

6

1

1

2

1

2

1

1

5

3

1

1

1

4

1

1

1

3

1

| 1742AL | 27Alagoas | União dos  Palmares | 270930 | 2709301 | 64900.6 | 9664.3 | 8023 | 8113.2 | 6810.7 | 12686 |  |
| --- | --- | --- | --- | --- | --- | --- | --- | --- | --- | --- | --- |
| 1743AL | 27Alagoas | Viçosa | 270940 | 2709400 | 25870.7 | 3852.4 | 3198.1 | 3234.1 | 2714.9 | 4785 |  |
| 1744SE | 28Sergipe | Amparo de São  Francisco | 280010 | 2800100 | 2345.9 | 356.6 | 305.5 | 293.6 | 255.7 | 312 |  |
| 1745SE | 28Sergipe | Aquidabã | 280020 | 2800209 | 21011 | 3193.7 | 2736.5 | 2629.4 | 2290.2 | 2112 |  |
| 1746SE | 28Sergipe | Aracaju | 280030 | 2800308 | 620708.1 | 94347.6 | 80841 | 77676.4 | 67655.9 | 112731 | 44 |
| 1747SE | 28Sergipe | Arauá | 280040 | 2800407 | 10425.1 | 1584.6 | 1357.8 | 1304.6 | 1136.3 | 2665 |  |
| 1748SE | 28Sergipe | Areia Branca | 280050 | 2800506 | 17877.5 | 2717.4 | 2328.4 | 2237.2 | 1948.6 | 3064 |  |
| 1749SE | 28Sergipe | Barra dos  Coqueiros | 280060 | 2800605 | 28016.1 | 4258.4 | 3648.8 | 3506 | 3053.7 | 4174 |  |
| 1750SE | 28Sergipe | Boquim | 280067 | 2800670 | 26417.4 | 4015.4 | 3440.6 | 3305.9 | 2879.4 | 5311 |  |
| 1751SE | 28Sergipe | Brejo Grande | 280070 | 2800704 | 8103.1 | 1231.7 | 1055.3 | 1014 | 883.2 | 453 |  |
| 1752SE | 28Sergipe | Campo do Brito | 280100 | 2801009 | 17598.8 | 2675 | 2292.1 | 2202.3 | 1918.2 | 2302 |  |
| 1753SE | 28Sergipe | Canhoba | 280110 | 2801108 | 4017.1 | 610.6 | 523.2 | 502.7 | 437.9 | 394 |  |
| 1754SE | 28Sergipe | Canindé de São  Francisco | 280120 | 2801207 | 27630 | 4199.8 | 3598.5 | 3457.7 | 3011.6 | 2777 |  |
| 1755SE | 28Sergipe | Capela | 280130 | 2801306 | 32819.6 | 4988.6 | 4274.4 | 4107.1 | 3577.3 | 1720 |  |
| 1756SE | 28Sergipe | Carira | 280140 | 2801405 | 21173.6 | 3218.4 | 2757.6 | 2649.7 | 2307.9 | 3186 |  |
| 1757SE | 28Sergipe | Carmópolis | 280150 | 2801504 | 15248.2 | 2317.7 | 1985.9 | 1908.2 | 1662 | 1677 |  |
| 1758SE | 28Sergipe | Cedro de São  João | 280160 | 2801603 | 5818.4 | 884.4 | 757.8 | 728.1 | 634.2 | 715 |  |
| 1759SE | 28Sergipe | Cristinápolis | 280170 | 2801702 | 17566.9 | 2670.2 | 2287.9 | 2198.3 | 1914.8 | 2320 |  |
| 1760SE | 28Sergipe | Cumbe | 280190 | 2801900 | 3936.3 | 598.3 | 512.7 | 492.6 | 429 | 762 |  |
| 1761SE | 28Sergipe | Divina Pastora | 280200 | 2802007 | 4785.9 | 727.5 | 623.3 | 598.9 | 521.7 | 567 |  |
| 1762SE | 28Sergipe | Estância | 280210 | 2802106 | 67442 | 10251.2 | 8783.6 | 8439.8 | 7351 | 17434 |  |
| 1763SE | 28Sergipe | Feira Nova | 280220 | 2802205 | 5504.6 | 836.7 | 716.9 | 688.9 | 600 | 915 |  |
| 1764SE | 28Sergipe | Frei Paulo | 280230 | 2802304 | 14797.6 | 2249.2 | 1927.2 | 1851.8 | 1612.9 | 1698 |  |
| 1765SE | 28Sergipe | Gararu | 280240 | 2802403 | 11605.5 | 1764 | 1511.5 | 1452.3 | 1265 | 894 |  |
| 1766SE | 28Sergipe | General Maynard | 280250 | 2802502 | 3171.2 | 482 | 413 | 396.8 | 345.7 | 300 |  |
| 1767SE | 28Sergipe | Gracho Cardoso | 280260 | 2802601 | 5783.1 | 879 | 753.2 | 723.7 | 630.3 | 1780 |  |
| 1768SE | 28Sergipe | Ilha das Flores | 280270 | 2802700 | 8508.2 | 1293.2 | 1108.1 | 1064.7 | 927.4 | 1203 |  |
| 1769SE | 28Sergipe | Indiaroba | 280280 | 2802809 | 17072.2 | 2595 | 2223.5 | 2136.4 | 1860.8 | 1818 |  |
| 1770SE | 28Sergipe | Itabaiana | 280290 | 2802908 | 92110.3 | 14000.8 | 11996.4 | 11526.8 | 10039.8 | 20344 |  |
| 1771SE | 28Sergipe | Itabaianinha | 280300 | 2803005 | 40811.7 | 6203.4 | 5315.3 | 5107.2 | 4448.4 | 5675 |  |
| 1772SE | 28Sergipe | Itabi | 280310 | 2803104 | 4978.1 | 756.7 | 648.3 | 623 | 542.6 | 1553 |  |
| 1773SE | 28Sergipe | Itaporanga d'Ajuda | 280320 | 2803203 | 32727 | 4974.5 | 4262.4 | 4095.5 | 3567.2 | 4274 |  |
| 1774SE | 28Sergipe | Japaratuba | 280330 | 2803302 | 17985.6 | 2733.8 | 2342.4 | 2250.7 | 1960.4 | 2166 |  |
| 1775SE | 28Sergipe | Japoatã | 280340 | 2803401 | 13154.2 | 1999.4 | 1713.2 | 1646.1 | 1433.8 | 1176 |  |
| 1776SE | 28Sergipe | Lagarto | 280350 | 2803500 | 100637.2 | 15296.9 | 13107 | 12593.9 | 10969.2 | 15049 |  |
| 1777SE | 28Sergipe | Laranjeiras | 280360 | 2803609 | 28652.9 | 4355.2 | 3731.8 | 3585.7 | 3123.1 | 2938 |  |
| 1778SE | 28Sergipe | Macambira | 280370 | 2803708 | 6725 | 1022.2 | 875.9 | 841.6 | 733 | 1112 |  |
| 1779SE | 28Sergipe | Malhada dos Bois | 280380 | 2803807 | 3603.3 | 547.7 | 469.3 | 450.9 | 392.8 | 609 |  |
| 1780SE | 28Sergipe | Malhador | 280390 | 2803906 | 12443.8 | 1891.5 | 1620.7 | 1557.2 | 1356.3 | 2239 |  |
| 1781SE | 28Sergipe | Maruim | 280400 | 2804003 | 16934.5 | 2574 | 2205.5 | 2119.2 | 1845.8 | 1447 |  |
| 1782SE | 28Sergipe | Moita Bonita | 280410 | 2804102 | 11268.7 | 1712.8 | 1467.6 | 1410.2 | 1228.3 | 2753 |  |
| 1783SE | 28Sergipe | Monte Alegre de  Sergipe | 280420 | 2804201 | 14574.3 | 2215.3 | 1898.2 | 1823.9 | 1588.6 | 1556 |  |
| 1784SE | 28Sergipe | Muribeca | 280430 | 2804300 | 7552.8 | 1148 | 983.7 | 945.2 | 823.2 | 906 |  |
| 1785SE | 28Sergipe | Neópolis | 280440 | 2804409 | 18774.4 | 2853.7 | 2445.2 | 2349.5 | 2046.4 | 2052 |  |
| 1786SE | 28Sergipe | Nossa Senhora  Aparecida | 280445 | 2804458 | 8730.2 | 1327 | 1137 | 1092.5 | 951.6 | 2271 |  |
| 1787SE | 28Sergipe | Nossa Senhora da  Glória | 280450 | 2804508 | 35078.2 | 5331.9 | 4568.6 | 4389.7 | 3823.4 | 7143 |  |
| 1788SE | 28Sergipe | Nossa Senhora das Dores | 280460 | 2804607 | 25855.2 | 3930 | 3367.4 | 3235.6 | 2818.2 | 1546 |  |
| 1789SE | 28Sergipe | Nossa Senhora de  Lourdes | 280470 | 2804706 | 6418.4 | 975.6 | 835.9 | 803.2 | 699.6 | 1371 |  |
| 1790SE | 28Sergipe | Nossa Senhora do  Socorro | 280480 | 2804805 | 174065.2 | 26457.9 | 22670.3 | 21782.8 | 18972.7 | 17778 |  |
| 1791SE | 28Sergipe | Pacatuba | 280490 | 2804904 | 13935.6 | 2118.2 | 1815 | 1743.9 | 1519 | 702 |  |
| 1792SE | 28Sergipe | Pedra Mole | 280500 | 2805000 | 3148.8 | 478.6 | 410.1 | 394 | 343.2 | 828 |  |
| 1793SE | 28Sergipe | Pedrinhas | 280510 | 2805109 | 9308.3 | 1414.9 | 1212.3 | 1164.9 | 1014.6 | 2137 |  |
| 1794SE | 28Sergipe | Pinhão | 280520 | 2805208 | 6337.8 | 963.3 | 825.4 | 793.1 | 690.8 | 936 |  |
| 1795SE | 28Sergipe | Pirambu | 280530 | 2805307 | 8914.6 | 1355 | 1161 | 1115.6 | 971.7 | 879 |  |
| 1796SE | 28Sergipe | Poço Redondo | 280540 | 2805406 | 33167.4 | 5041.4 | 4319.7 | 4150.6 | 3615.2 | 2028 |  |
| 1797SE | 28Sergipe | Poço Verde | 280550 | 2805505 | 23078.4 | 3507.9 | 3005.7 | 2888.1 | 2515.5 | 2560 |  |
| 1798SE | 28Sergipe | Porto da Folha | 280560 | 2805604 | 28131.4 | 4276 | 3663.8 | 3520.4 | 3066.3 | 3180 |  |
| 1799SE | 28Sergipe | Propriá | 280570 | 2805703 | 29304.4 | 4454.3 | 3816.6 | 3667.2 | 3194.1 | 2804 |  |
| 1800SE | 28Sergipe | Riachão do  Dantas | 280580 | 2805802 | 19768.4 | 3004.8 | 2574.6 | 2473.8 | 2154.7 | 1757 |  |
| 1801SE | 28Sergipe | Riachuelo | 280590 | 2805901 | 9880.7 | 1501.9 | 1286.9 | 1236.5 | 1077 | 774 |  |
| 1802SE | 28Sergipe | Ribeirópolis | 280600 | 2806008 | 18088.9 | 2749.5 | 2355.9 | 2263.7 | 1971.7 | 2399 |  |
| 1803SE | 28Sergipe | Rosário do Catete | 280610 | 2806107 | 10149.8 | 1542.8 | 1321.9 | 1270.2 | 1106.3 | 794 |  |
| 1804SE | 28Sergipe | Salgado | 280620 | 2806206 | 19858.5 | 3018.5 | 2586.4 | 2485.1 | 2164.5 | 2210 |  |
| 1805SE | 28Sergipe | Santa Luzia do  Itanhy | 280630 | 2806305 | 13726.3 | 2086.4 | 1787.7 | 1717.7 | 1496.1 | 1885 |  |
| 1806SE | 28Sergipe | Santana do São  Francisco | 280640 | 2806404 | 7484 | 1137.6 | 974.7 | 936.6 | 815.7 | 525 |  |
| 1807SE | 28Sergipe | Santa Rosa de  Lima | 280650 | 2806503 | 3866.2 | 587.7 | 503.5 | 483.8 | 421.4 | 444 |  |
| 1808SE | 28Sergipe | Santo Amaro das  Brotas | 280660 | 2806602 | 11867.1 | 1803.8 | 1545.6 | 1485.1 | 1293.5 | 684 |  |
| 1809SE | 28Sergipe | São Cristóvão | 280670 | 2806701 | 85369.2 | 12976.1 | 11118.5 | 10683.2 | 9305.1 | 7947 |  |
| 1810SE | 28Sergipe | São Domingos | 280680 | 2806800 | 10809.1 | 1643 | 1407.8 | 1352.7 | 1178.2 | 1849 |  |
| 1811SE | 28Sergipe | São Francisco | 280690 | 2806909 | 3731.5 | 567.2 | 486 | 467 | 406.7 | 487 |  |
| 1812SE | 28Sergipe | São Miguel do  Aleixo | 280700 | 2807006 | 3850.2 | 585.2 | 501.5 | 481.8 | 419.7 | 849 |  |
| 1813SE | 28Sergipe | Simão Dias | 280710 | 2807105 | 40011.8 | 6081.8 | 5211.1 | 5007.1 | 4361.2 | 3021 |  |
| 1814SE | 28Sergipe | Siriri | 280720 | 2807204 | 8534.4 | 1297.2 | 1111.5 | 1068 | 930.2 | 1037 |  |
| 1815SE | 28Sergipe | Telha | 280730 | 2807303 | 3122.3 | 474.6 | 406.6 | 390.7 | 340.3 | 375 |  |
| 1816SE | 28Sergipe | Tobias Barreto | 280740 | 2807402 | 50609.3 | 7692.6 | 6591.4 | 6333.3 | 5516.3 | 7504 |  |
| 1817SE | 28Sergipe | Tomar do Geru | 280750 | 2807501 | 13116.6 | 1993.7 | 1708.3 | 1641.4 | 1429.7 | 1572 |  |
| 1818SE | 28Sergipe | Umbaúba | 280760 | 2807600 | 24138.5 | 3669.1 | 3143.8 | 3020.7 | 2631 | 4094 |  |
| 1819BA | 29Bahia | Abaíra | 290010 | 2900108 | 8918 | 1444.6 | 1146.5 | 1256.1 | 983.6 | 2887 |  |
| 1820BA | 29Bahia | Abaré | 290020 | 2900207 | 19000.9 | 3078 | 2442.8 | 2676.3 | 2095.6 | 2817 |  |
| 1821BA | 29Bahia | Acajutiba | 290030 | 2900306 | 15285.4 | 2476.1 | 1965.1 | 2153 | 1685.9 | 1962 |  |
| 1822BA | 29Bahia | Adustina | 290035 | 2900355 | 16697.8 | 2704.9 | 2146.7 | 2351.9 | 1841.6 | 1986 |  |
| 1823BA | 29Bahia | Água Fria | 290040 | 2900405 | 16710.7 | 2707 | 2148.3 | 2353.7 | 1843.1 | 2635 |  |
| 1824BA | 29Bahia | Érico Cardoso | 290050 | 2900504 | 11071.3 | 1793.4 | 1423.3 | 1559.4 | 1221.1 | 2299 |  |
| 1825BA | 29Bahia | Aiquara | 290060 | 2900603 | 4642.4 | 752 | 596.8 | 653.9 | 512 | 806 |  |
| 1826BA | 29Bahia | Alagoinhas | 290070 | 2900702 | 150267.2 | 24341.8 | 19318.4 | 21165.2 | 16573.2 | 22584 |  |
| 1827BA | 29Bahia | Alcobaça | 290080 | 2900801 | 22521.3 | 3648.2 | 2895.3 | 3172.1 | 2483.9 | 2020 |  |
| 1828BA | 29Bahia | Almadina | 290090 | 2900900 | 6051.1 | 980.2 | 777.9 | 852.3 | 667.4 | 2156 |  |
| 1829BA | 29Bahia | Amargosa | 290100 | 2901007 | 36681.5 | 5942 | 4715.8 | 5166.6 | 4045.7 | 4993 |  |
| 1830BA | 29Bahia | Amélia Rodrigues | 290110 | 2901106 | 25793.9 | 4178.4 | 3316.1 | 3633.1 | 2844.9 | 5008 |  |
| 1831BA | 29Bahia | América Dourada | 290115 | 2901155 | 16436.5 | 2662.5 | 2113.1 | 2315.1 | 1812.8 | 2230 |  |
| 1832BA | 29Bahia | Anagé | 290120 | 2901205 | 20707.9 | 3354.5 | 2662.2 | 2916.7 | 2283.9 | 3760 |  |
| 1833BA | 29Bahia | Andaraí | 290130 | 2901304 | 13779.5 | 2232.1 | 1771.5 | 1940.8 | 1519.8 | 2103 |  |
| 1834BA | 29Bahia | Andorinha | 290135 | 2901353 | 14784.8 | 2395 | 1900.7 | 2082.4 | 1630.6 | 3548 |  |
| 1835BA | 29Bahia | Angical | 290140 | 2901403 | 14371.5 | 2328 | 1847.6 | 2024.2 | 1585.1 | 2239 |  |
| 1836BA | 29Bahia | Anguera | 290150 | 2901502 | 10987.2 | 1779.8 | 1412.5 | 1547.6 | 1211.8 | 1984 |  |
| 1837BA | 29Bahia | Antas | 290160 | 2901601 | 18613.2 | 3015.2 | 2392.9 | 2621.7 | 2052.9 | 1889 |  |
| 1838BA | 29Bahia | Antônio Cardoso | 290170 | 2901700 | 11907.7 | 1928.9 | 1530.9 | 1677.2 | 1313.3 | 2154 |  |
| 1839BA | 29Bahia | Antônio Gonçalves | 290180 | 2901809 | 11718.4 | 1898.3 | 1506.5 | 1650.5 | 1292.4 | 3087 |  |

4

1

1

1

1

2

1

1

1

1

6

8

2

1

6

1

1

1

2

7

1

1

1

1

1

3

1

2

1

6

1

1

1

1

| 1840BA |  | Aporá | 290190 | 2901908 | 18355.9 | 2973.5 | 2359.8 | 2585.4 | 2024.5 | 1985 |  |
| --- | --- | --- | --- | --- | --- | --- | --- | --- | --- | --- | --- |
| 1841BA | 29Bahia | Apuarema | 290195 | 2901957 | 7578.5 | 1227.6 | 974.3 | 1067.4 | 835.8 | 1230 |  |
| 1842BA | 29Bahia | Aracatu | 290200 | 2902005 | 13775.4 | 2231.5 | 1771 | 1940.3 | 1519.3 | 2697 |  |
| 1843BA | 29Bahia | Araças | 290205 | 2902054 | 12143.1 | 1967.1 | 1561.1 | 1710.4 | 1339.3 | 1785 |  |
| 1844BA | 29Bahia | Araci | 290210 | 2902104 | 54406.8 | 8813.4 | 6994.5 | 7663.2 | 6000.6 | 7935 |  |
| 1845BA | 29Bahia | Aramari | 290220 | 2902203 | 11015.5 | 1784.4 | 1416.2 | 1551.5 | 1214.9 | 1103 |  |
| 1846BA | 29Bahia | Arataca | 290225 | 2902252 | 11184.1 | 1811.7 | 1437.8 | 1575.3 | 1233.5 | 2237 |  |
| 1847BA | 29Bahia | Aratuípe | 290230 | 2902302 | 8918.3 | 1444.7 | 1146.5 | 1256.1 | 983.6 | 1167 |  |
| 1848BA | 29Bahia | Aurelino Leal | 290240 | 2902401 | 12879.3 | 2086.3 | 1655.8 | 1814.1 | 1420.5 | 2739 |  |
| 1849BA | 29Bahia | Baianópolis | 290250 | 2902500 | 13968.7 | 2262.8 | 1795.8 | 1967.5 | 1540.6 | 2874 |  |
| 1850BA | 29Bahia | Baixa Grande | 290260 | 2902609 | 20744.5 | 3360.4 | 2666.9 | 2921.9 | 2287.9 | 2434 |  |
| 1851BA | 29Bahia | Banzaê | 290265 | 2902658 | 12823.5 | 2077.3 | 1648.6 | 1806.2 | 1414.3 | 2446 |  |
| 1852BA | 29Bahia | Barra | 290270 | 2902708 | 52681.7 | 8533.9 | 6772.8 | 7420.2 | 5810.4 | 10927 |  |
| 1853BA | 29Bahia | Barra da Estiva | 290280 | 2902807 | 21602.1 | 3499.3 | 2777.2 | 3042.7 | 2382.5 | 11484 |  |
| 1854BA | 29Bahia | Barra do Choça | 290290 | 2902906 | 34315.6 | 5558.8 | 4411.6 | 4833.4 | 3784.7 | 4429 |  |
| 1855BA | 29Bahia | Barra do Mendes | 290300 | 2903003 | 14249.6 | 2308.3 | 1831.9 | 2007.1 | 1571.6 | 2215 |  |
| 1856BA | 29Bahia | Barra do Rocha | 290310 | 2903102 | 6185.8 | 1002 | 795.2 | 871.3 | 682.2 | 1122 |  |
| 1857BA | 29Bahia | Barreiras | 290320 | 2903201 | 149724.1 | 24253.8 | 19248.5 | 21088.7 | 16513.3 | 33091 | 12 |
| 1858BA | 29Bahia | Barro Alto | 290323 | 2903235 | 14679.6 | 2377.9 | 1887.2 | 2067.6 | 1619 | 1863 |  |
| 1859BA | 29Bahia | Barrocas | 290327 | 2903276 | 15418.2 | 2497.6 | 1982.2 | 2171.7 | 1700.5 | 4648 |  |
| 1860BA | 29Bahia | Barro Preto | 290330 | 2903300 | 6269.8 | 1015.6 | 806 | 883.1 | 691.5 | 1596 |  |
| 1861BA | 29Bahia | Belmonte | 290340 | 2903409 | 23109.6 | 3743.5 | 2971 | 3255 | 2548.8 | 4889 |  |
| 1862BA | 29Bahia | Belo Campo | 290350 | 2903508 | 17602.9 | 2851.5 | 2263 | 2479.4 | 1941.5 | 3435 |  |
| 1863BA | 29Bahia | Biritinga | 290360 | 2903607 | 15709.3 | 2544.7 | 2019.6 | 2212.7 | 1732.6 | 2661 |  |
| 1864BA | 29Bahia | Boa Nova | 290370 | 2903706 | 14362.7 | 2326.6 | 1846.5 | 2023 | 1584.1 | 2266 |  |
| 1865BA | 29Bahia | Boa Vista do  Tupim | 290380 | 2903805 | 18627.1 | 3017.4 | 2394.7 | 2623.6 | 2054.4 | 2141 |  |
| 1866BA | 29Bahia | Bom Jesus da  Lapa | 290390 | 2903904 | 67753.5 | 10975.4 | 8710.4 | 9543.1 | 7472.6 | 16483 |  |
| 1867BA | 29Bahia | Bom Jesus da  Serra | 290395 | 2903953 | 10289.5 | 1666.8 | 1322.8 | 1449.3 | 1134.8 | 1792 |  |
| 1868BA | 29Bahia | Boninal | 290400 | 2904001 | 14329.1 | 2321.2 | 1842.1 | 2018.3 | 1580.4 | 3167 |  |
| 1869BA | 29Bahia | Bonito | 290405 | 2904050 | 16221.5 | 2627.7 | 2085.4 | 2284.8 | 1789.1 | 1990 |  |
| 1870BA | 29Bahia | Boquira | 290410 | 2904100 | 22126 | 3584.2 | 2844.5 | 3116.5 | 2440.3 | 4053 |  |
| 1871BA | 29Bahia | Botuporã | 290420 | 2904209 | 10879.2 | 1762.3 | 1398.6 | 1532.3 | 1199.9 | 2547 |  |
| 1872BA | 29Bahia | Brejões | 290430 | 2904308 | 14690.4 | 2379.7 | 1888.6 | 2069.1 | 1620.2 | 2298 |  |
| 1873BA | 29Bahia | Brejolândia | 290440 | 2904407 | 10800.5 | 1749.6 | 1388.5 | 1521.3 | 1191.2 | 4158 |  |
| 1874BA | 29Bahia | Brotas de  Macaúbas | 290450 | 2904506 | 10791.6 | 1748.1 | 1387.4 | 1520 | 1190.2 | 2433 |  |
| 1875BA | 29Bahia | Brumado | 290460 | 2904605 | 67481 | 10931.2 | 8675.4 | 9504.7 | 7442.6 | 22254 |  |
| 1876BA | 29Bahia | Buerarema | 290470 | 2904704 | 18885.5 | 3059.3 | 2427.9 | 2660 | 2082.9 | 4954 |  |
| 1877BA | 29Bahia | Buritirama | 290475 | 2904753 | 20896.1 | 3385 | 2686.4 | 2943.2 | 2304.7 | 3940 |  |
| 1878BA | 29Bahia | Caatiba | 290480 | 2904803 | 9617.8 | 1558 | 1236.5 | 1354.7 | 1060.8 | 814 |  |
| 1879BA | 29Bahia | Cabaceiras do  Paraguaçu | 290485 | 2904852 | 18492.2 | 2995.6 | 2377.4 | 2604.6 | 2039.5 | 3571 |  |
| 1880BA | 29Bahia | Cachoeira | 290490 | 2904902 | 33710.2 | 5460.7 | 4333.8 | 4748.1 | 3718 | 6338 |  |
| 1881BA | 29Bahia | Caculé | 290500 | 2905008 | 23109.4 | 3743.5 | 2970.9 | 3255 | 2548.8 | 4258 |  |
| 1882BA | 29Bahia | Caém | 290510 | 2905107 | 10024.4 | 1623.9 | 1288.7 | 1411.9 | 1105.6 | 1589 |  |
| 1883BA | 29Bahia | Caetanos | 290515 | 2905156 | 15327.6 | 2482.9 | 1970.5 | 2158.9 | 1690.5 | 2241 |  |
| 1884BA | 29Bahia | Caetité | 290520 | 2905206 | 50737.1 | 8218.9 | 6522.8 | 7146.3 | 5595.9 | 7454 |  |
| 1885BA | 29Bahia | Cafarnaum | 290530 | 2905305 | 18240.7 | 2954.8 | 2345 | 2569.2 | 2011.8 | 3658 |  |
| 1886BA | 29Bahia | Cairu | 290540 | 2905404 | 17167.8 | 2781 | 2207.1 | 2418.1 | 1893.5 | 2155 |  |
| 1887BA | 29Bahia | Caldeirão Grande | 290550 | 2905503 | 13232.9 | 2143.6 | 1701.2 | 1863.9 | 1459.5 | 2309 |  |
| 1888BA | 29Bahia | Camacan | 290560 | 2905602 | 32443.4 | 5255.5 | 4170.9 | 4569.7 | 3578.2 | 4463 |  |
| 1889BA | 29Bahia | Camaçari | 290570 | 2905701 | 277314.3 | 44922.1 | 35651.5 | 39059.8 | 30585.4 | 46895 | 17 |
| 1890BA | 29Bahia | Camamu | 290580 | 2905800 | 35953.4 | 5824.1 | 4622.2 | 5064 | 3965.4 | 4491 |  |
| 1891BA | 29Bahia | Campo Alegre de  Lourdes | 290590 | 2905909 | 29168 | 4724.9 | 3749.8 | 4108.3 | 3217 | 9277 |  |
| 1892BA | 29Bahia | Campo Formoso | 290600 | 2906006 | 70524.9 | 11424.3 | 9066.7 | 9933.5 | 7778.3 | 25544 |  |
| 1893BA | 29Bahia | Canápolis | 290610 | 2906105 | 9834.3 | 1593.1 | 1264.3 | 1385.2 | 1084.6 | 9135 |  |
| 1894BA | 29Bahia | Canarana | 290620 | 2906204 | 25709.6 | 4164.7 | 3305.2 | 3621.2 | 2835.6 | 4594 |  |
| 1895BA | 29Bahia | Canavieiras | 290630 | 2906303 | 32513.8 | 5266.9 | 4180 | 4579.6 | 3586 | 5610 |  |
| 1896BA | 29Bahia | Candeal | 290640 | 2906402 | 8797.7 | 1425.1 | 1131 | 1239.2 | 970.3 | 2455 |  |
| 1897BA | 29Bahia | Candeias | 290650 | 2906501 | 87019 | 14096.2 | 11187.2 | 12256.7 | 9597.5 | 10419 |  |
| 1898BA | 29Bahia | Candiba | 290660 | 2906600 | 14171.3 | 2295.6 | 1821.9 | 1996 | 1563 | 3277 |  |
| 1899BA | 29Bahia | Cândido Sales | 290670 | 2906709 | 26619.5 | 4312.1 | 3422.2 | 3749.4 | 2935.9 | 4529 |  |
| 1900BA | 29Bahia | Cansanção | 290680 | 2906808 | 34625.7 | 5609 | 4451.5 | 4877 | 3818.9 | 7034 |  |
| 1901BA | 29Bahia | Canudos | 290682 | 2906824 | 16667.2 | 2699.9 | 2142.7 | 2347.6 | 1838.3 | 3246 |  |
| 1902BA | 29Bahia | Capela do Alto  Alegre | 290685 | 2906857 | 11858.5 | 1921 | 1524.5 | 1670.3 | 1307.9 | 5090 |  |
| 1903BA | 29Bahia | Capim Grosso | 290687 | 2906873 | 29146.2 | 4721.4 | 3747 | 4105.3 | 3214.6 | 4552 |  |
| 1904BA | 29Bahia | Caraíbas | 290689 | 2906899 | 9832.7 | 1592.8 | 1264.1 | 1384.9 | 1084.5 | 1495 |  |
| 1905BA | 29Bahia | Caravelas | 290690 | 2906907 | 22119.8 | 3583.2 | 2843.7 | 3115.6 | 2439.6 | 1882 |  |
| 1906BA | 29Bahia | Cardeal da Silva | 290700 | 2907004 | 9382.1 | 1519.8 | 1206.2 | 1321.5 | 1034.8 | 1306 |  |
| 1907BA | 29Bahia | Carinhanha | 290710 | 2907103 | 29307.8 | 4747.6 | 3767.8 | 4128 | 3232.4 | 5099 |  |
| 1908BA | 29Bahia | Casa Nova | 290720 | 2907202 | 70090.5 | 11354 | 9010.8 | 9872.3 | 7730.4 | 15012 |  |
| 1909BA | 29Bahia | Castro Alves | 290730 | 2907301 | 26489.4 | 4291 | 3405.5 | 3731 | 2921.6 | 4972 |  |
| 1910BA | 29Bahia | Catolândia | 290740 | 2907400 | 3369 | 545.7 | 433.1 | 474.5 | 371.6 | 741 |  |
| 1911BA | 29Bahia | Catu | 290750 | 2907509 | 54196.6 | 8779.3 | 6967.5 | 7633.6 | 5977.4 | 8536 |  |
| 1912BA | 29Bahia | Caturama | 290755 | 2907558 | 9397 | 1522.2 | 1208.1 | 1323.6 | 1036.4 | 1947 |  |
| 1913BA | 29Bahia | Central | 290760 | 2907608 | 17582.9 | 2848.3 | 2260.5 | 2476.6 | 1939.2 | 2157 |  |
| 1914BA | 29Bahia | Chorrochó | 290770 | 2907707 | 11222.2 | 1817.9 | 1442.7 | 1580.7 | 1237.7 | 1914 |  |
| 1915BA | 29Bahia | Cícero Dantas | 290780 | 2907806 | 33572.6 | 5438.4 | 4316.1 | 4728.7 | 3702.8 | 3564 |  |
| 1916BA | 29Bahia | Cipó | 290790 | 2907905 | 16866.8 | 2732.3 | 2168.4 | 2375.7 | 1860.3 | 2142 |  |
| 1917BA | 29Bahia | Coaraci | 290800 | 2908002 | 19477.2 | 3155.1 | 2504 | 2743.4 | 2148.2 | 7123 |  |
| 1918BA | 29Bahia | Cocos | 290810 | 2908101 | 18907 | 3062.7 | 2430.7 | 2663.1 | 2085.3 | 5071 |  |
| 1919BA | 29Bahia | Conceição da  Feira | 290820 | 2908200 | 21999.6 | 3563.7 | 2828.3 | 3098.7 | 2426.4 | 5182 |  |
| 1920BA | 29Bahia | Conceição do  Almeida | 290830 | 2908309 | 18022.7 | 2919.5 | 2317 | 2538.5 | 1987.8 | 2972 |  |
| 1921BA | 29Bahia | Conceição do  Coité | 290840 | 2908408 | 65952.2 | 10683.6 | 8478.8 | 9289.4 | 7274 | 21725 |  |
| 1922BA | 29Bahia | Conceição do  Jacuípe | 290850 | 2908507 | 32400.8 | 5248.6 | 4165.4 | 4563.7 | 3573.5 | 9744 |  |
| 1923BA | 29Bahia | Conde | 290860 | 2908606 | 25370.7 | 4109.8 | 3261.7 | 3573.5 | 2798.2 | 3150 |  |
| 1924BA | 29Bahia | Condeúba | 290870 | 2908705 | 17708.7 | 2868.6 | 2276.6 | 2494.3 | 1953.1 | 7736 |  |
| 1925BA | 29Bahia | Contendas do  Sincorá | 290880 | 2908804 | 4369.7 | 707.8 | 561.8 | 615.5 | 481.9 | 667 |  |
| 1926BA | 29Bahia | Coração de Maria | 290890 | 2908903 | 22969.2 | 3720.8 | 2952.9 | 3235.2 | 2533.3 | 6041 |  |
| 1927BA | 29Bahia | Cordeiros | 290900 | 2909000 | 8597.1 | 1392.6 | 1105.2 | 1210.9 | 948.2 | 2751 |  |
| 1928BA | 29Bahia | Coribe | 290910 | 2909109 | 14609.5 | 2366.6 | 1878.2 | 2057.8 | 1611.3 | 6515 |  |
| 1929BA | 29Bahia | Coronel João Sá | 290920 | 2909208 | 16780.7 | 2718.3 | 2157.3 | 2363.6 | 1850.8 | 2397 |  |
| 1930BA | 29Bahia | Correntina | 290930 | 2909307 | 32407.1 | 5249.6 | 4166.3 | 4564.5 | 3574.2 | 6481 |  |
| 1931BA | 29Bahia | Cotegipe | 290940 | 2909406 | 14046.4 | 2275.4 | 1805.8 | 1978.4 | 1549.2 | 2286 |  |
| 1932BA | 29Bahia | Cravolândia | 290950 | 2909505 | 5342.8 | 865.5 | 686.9 | 752.5 | 589.3 | 784 |  |
| 1933BA | 29Bahia | Crisópolis | 290960 | 2909604 | 21062.7 | 3411.9 | 2707.8 | 2966.7 | 2323 | 2021 |  |
| 1934BA | 29Bahia | Cristópolis | 290970 | 2909703 | 13925.8 | 2255.8 | 1790.3 | 1961.5 | 1535.9 | 2306 |  |
| 1935BA | 29Bahia | Cruz das Almas | 290980 | 2909802 | 62397.2 | 10107.7 | 8021.8 | 8788.7 | 6881.9 | 9809 |  |
| 1936BA | 29Bahia | Curaçá | 290990 | 2909901 | 34204.2 | 5540.7 | 4397.3 | 4817.7 | 3772.4 | 9133 |  |
| 1937BA | 29Bahia | Dário Meira | 291000 | 2910008 | 11979 | 1940.5 | 1540 | 1687.2 | 1321.2 | 1661 |  |
| 1938BA | 29Bahia | Dias d'Ávila | 291005 | 2910057 | 75474.5 | 12226.1 | 9703 | 10630.6 | 8324.2 | 7165 |  |
| 1939BA | 29Bahia | Dom Basílio | 291010 | 2910107 | 12104.4 | 1960.8 | 1556.1 | 1704.9 | 1335 | 2984 |  |
| 1940BA | 29Bahia | Dom Macedo  Costa | 291020 | 2910206 | 4059.3 | 657.6 | 521.9 | 571.8 | 447.7 | 1055 |  |

2

3

1

1

4

1

7

1

1

1

1

3

8

3

3

1

1

1

6

1

1

1

1

1

7

2

2

1

1

2

2

4

1

| 1941BA |  | Elísio Medrado | 291030 | 2910305 | 8229.2 | 1333 | 1057.9 | 1159.1 | 907.6 | 1870 |  |
| --- | --- | --- | --- | --- | --- | --- | --- | --- | --- | --- | --- |
| 1942BA | 29Bahia | Encruzilhada | 291040 | 2910404 | 20663.2 | 3347.2 | 2656.5 | 2910.4 | 2279 | 2899 |  |
| 1943BA | 29Bahia | Entre Rios | 291050 | 2910503 | 41838.3 | 6777.4 | 5378.7 | 5892.9 | 4614.4 | 5758 |  |
| 1944BA | 29Bahia | Esplanada | 291060 | 2910602 | 35767.8 | 5794 | 4598.3 | 5037.9 | 3944.9 | 2962 |  |
| 1945BA | 29Bahia | Euclides da Cunha | 291070 | 2910701 | 59600.7 | 9654.7 | 7662.3 | 8394.8 | 6573.5 | 8509 |  |
| 1946BA | 29Bahia | Eunápolis | 291072 | 2910727 | 109554.5 | 17746.7 | 14084.3 | 15430.8 | 12082.9 | 24743 |  |
| 1947BA | 29Bahia | Fátima | 291075 | 2910750 | 18071.1 | 2927.3 | 2323.2 | 2545.3 | 1993.1 | 3129 |  |
| 1948BA | 29Bahia | Feira da Mata | 291077 | 2910776 | 5944.7 | 963 | 764.3 | 837.3 | 655.7 | 2750 |  |
| 1949BA | 29Bahia | Feira de Santana | 291080 | 2910800 | 599777.5 | 97158 | 77107.4 | 84478.8 | 66150.4 | 165629 | 52 |
| 1950BA | 29Bahia | Filadélfia | 291085 | 2910859 | 17004.3 | 2754.5 | 2186.1 | 2395.1 | 1875.4 | 6036 |  |
| 1951BA | 29Bahia | Firmino Alves | 291090 | 2910909 | 5634.4 | 912.7 | 724.4 | 793.6 | 621.4 | 1675 |  |
| 1952BA | 29Bahia | Floresta Azul | 291100 | 2911006 | 10981.6 | 1778.9 | 1411.8 | 1546.8 | 1211.2 | 2576 |  |
| 1953BA | 29Bahia | Formosa do Rio  Preto | 291110 | 2911105 | 24626.1 | 3989.2 | 3165.9 | 3468.6 | 2716.1 | 2437 |  |
| 1954BA | 29Bahia | Gandu | 291120 | 2911204 | 32132.1 | 5205.1 | 4130.9 | 4525.8 | 3543.9 | 4487 |  |
| 1955BA | 29Bahia | Gavião | 291125 | 2911253 | 4620.9 | 748.5 | 594.1 | 650.9 | 509.6 | 1685 |  |
| 1956BA | 29Bahia | Gentio do Ouro | 291130 | 2911303 | 11179.1 | 1810.9 | 1437.2 | 1574.6 | 1233 | 1267 |  |
| 1957BA | 29Bahia | Glória | 291140 | 2911402 | 15547.8 | 2518.6 | 1998.8 | 2189.9 | 1714.8 | 2663 |  |
| 1958BA | 29Bahia | Gongogi | 291150 | 2911501 | 7941.1 | 1286.4 | 1020.9 | 1118.5 | 875.8 | 1375 |  |
| 1959BA | 29Bahia | Governador  Mangabeira | 291160 | 2911600 | 20753.7 | 3361.9 | 2668.1 | 2923.2 | 2289 | 5979 |  |
| 1960BA | 29Bahia | Guajeru | 291165 | 2911659 | 8749.2 | 1417.3 | 1124.8 | 1232.3 | 965 | 3382 |  |
| 1961BA | 29Bahia | Guanambi | 291170 | 2911709 | 83546.4 | 13533.7 | 10740.7 | 11767.5 | 9214.5 | 23437 |  |
| 1962BA | 29Bahia | Guaratinga | 291180 | 2911808 | 21964.7 | 3558.1 | 2823.8 | 3093.7 | 2422.5 | 4683 |  |
| 1963BA | 29Bahia | Heliópolis | 291185 | 2911857 | 13435.9 | 2176.5 | 1727.3 | 1892.5 | 1481.9 | 1465 |  |
| 1964BA | 29Bahia | Iaçu | 291190 | 2911907 | 25642.7 | 4153.9 | 3296.6 | 3611.8 | 2828.2 | 4775 |  |
| 1965BA | 29Bahia | Ibiassucê | 291200 | 2912004 | 10071.8 | 1631.5 | 1294.8 | 1418.6 | 1110.8 | 3743 |  |
| 1966BA | 29Bahia | Ibicaraí | 291210 | 2912103 | 23567.1 | 3817.6 | 3029.8 | 3319.4 | 2599.3 | 5933 |  |
| 1967BA | 29Bahia | Ibicoara | 291220 | 2912202 | 18884.4 | 3059.1 | 2427.8 | 2659.9 | 2082.8 | 2373 |  |
| 1968BA | 29Bahia | Ibicuí | 291230 | 2912301 | 16308.7 | 2641.8 | 2096.6 | 2297.1 | 1798.7 | 2244 |  |
| 1969BA | 29Bahia | Ibipeba | 291240 | 2912400 | 18059.1 | 2925.4 | 2321.7 | 2543.6 | 1991.8 | 2653 |  |
| 1970BA | 29Bahia | Ibipitanga | 291250 | 2912509 | 14890.1 | 2412 | 1914.3 | 2097.3 | 1642.3 | 2739 |  |
| 1971BA | 29Bahia | Ibiquera | 291260 | 2912608 | 4658.3 | 754.6 | 598.9 | 656.1 | 513.8 | 361 |  |
| 1972BA | 29Bahia | Ibirapitanga | 291270 | 2912707 | 23552.8 | 3815.3 | 3027.9 | 3317.4 | 2597.7 | 3777 |  |
| 1973BA | 29Bahia | Ibirapuã | 291280 | 2912806 | 8493.9 | 1375.9 | 1092 | 1196.4 | 936.8 | 1078 |  |
| 1974BA | 29Bahia | Ibirataia | 291290 | 2912905 | 17605.1 | 2851.9 | 2263.3 | 2479.7 | 1941.7 | 4262 |  |
| 1975BA | 29Bahia | Ibitiara | 291300 | 2913002 | 16297.2 | 2640 | 2095.2 | 2295.5 | 1797.4 | 3946 |  |
| 1976BA | 29Bahia | Ibititá | 291310 | 2913101 | 17979 | 2912.4 | 2311.4 | 2532.3 | 1982.9 | 2917 |  |
| 1977BA | 29Bahia | Ibotirama | 291320 | 2913200 | 26830.5 | 4346.3 | 3449.3 | 3779.1 | 2959.2 | 4617 |  |
| 1978BA | 29Bahia | Ichu | 291330 | 2913309 | 6055.1 | 980.9 | 778.4 | 852.9 | 667.8 | 1735 |  |
| 1979BA | 29Bahia | Igaporã | 291340 | 2913408 | 15805 | 2560.3 | 2031.9 | 2226.1 | 1743.2 | 4023 |  |
| 1980BA | 29Bahia | Igrapiúna | 291345 | 2913457 | 13716.2 | 2221.9 | 1763.4 | 1931.9 | 1512.8 | 3065 |  |
| 1981BA | 29Bahia | Iguaí | 291350 | 2913507 | 27022 | 4377.3 | 3473.9 | 3806.1 | 2980.3 | 4606 |  |
| 1982BA | 29Bahia | Ilhéus | 291360 | 2913606 | 178625.3 | 28935.5 | 22964.1 | 25159.4 | 19700.9 | 39658 | 12 |
| 1983BA | 29Bahia | Inhambupe | 291370 | 2913705 | 39252.9 | 6358.6 | 5046.4 | 5528.8 | 4329.3 | 6740 |  |
| 1984BA | 29Bahia | Ipecaetá | 291380 | 2913804 | 15264.6 | 2472.7 | 1962.4 | 2150 | 1683.6 | 2183 |  |
| 1985BA | 29Bahia | Ipiaú | 291390 | 2913903 | 46246.6 | 7491.5 | 5945.5 | 6513.8 | 5100.6 | 6962 |  |
| 1986BA | 29Bahia | Ipirá | 291400 | 2914000 | 60871.9 | 9860.6 | 7825.7 | 8573.8 | 6713.7 | 9156 |  |
| 1987BA | 29Bahia | Ipupiara | 291410 | 2914109 | 9813 | 1589.6 | 1261.6 | 1382.2 | 1082.3 | 1716 |  |
| 1988BA | 29Bahia | Irajuba | 291420 | 2914208 | 7297.9 | 1182.2 | 938.2 | 1027.9 | 804.9 | 961 |  |
| 1989BA | 29Bahia | Iramaia | 291430 | 2914307 | 10492.6 | 1699.7 | 1348.9 | 1477.9 | 1157.2 | 1755 |  |
| 1990BA | 29Bahia | Iraquara | 291440 | 2914406 | 24434.9 | 3958.2 | 3141.4 | 3441.7 | 2695 | 3431 |  |
| 1991BA | 29Bahia | Irará | 291450 | 2914505 | 28975 | 4693.7 | 3725 | 4081.1 | 3195.7 | 5464 |  |
| 1992BA | 29Bahia | Irecê | 291460 | 2914604 | 71247.5 | 11541.4 | 9159.6 | 10035.2 | 7858 | 12512 |  |
| 1993BA | 29Bahia | Itabela | 291465 | 2914653 | 30197.9 | 4891.8 | 3882.2 | 4253.4 | 3330.6 | 5786 |  |
| 1994BA | 29Bahia | Itaberaba | 291470 | 2914703 | 64589.8 | 10462.9 | 8303.7 | 9097.5 | 7123.7 | 5885 |  |
| 1995BA | 29Bahia | Itabuna | 291480 | 2914802 | 213996.2 | 34665.2 | 27511.4 | 30141.4 | 23602 | 47279 | 13 |
| 1996BA | 29Bahia | Itacaré | 291490 | 2914901 | 26851.8 | 4349.7 | 3452.1 | 3782.1 | 2961.5 | 2414 |  |
| 1997BA | 29Bahia | Itaeté | 291500 | 2915007 | 15895.3 | 2574.9 | 2043.5 | 2238.9 | 1753.1 | 2657 |  |
| 1998BA | 29Bahia | Itagi | 291510 | 2915106 | 13031 | 2110.9 | 1675.3 | 1835.4 | 1437.2 | 1886 |  |
| 1999BA | 29Bahia | Itagibá | 291520 | 2915205 | 15333.7 | 2483.9 | 1971.3 | 2159.8 | 1691.2 | 2239 |  |
| 2000BA | 29Bahia | Itagimirim | 291530 | 2915304 | 7173.2 | 1162 | 922.2 | 1010.3 | 791.1 | 1784 |  |
| 2001BA | 29Bahia | Itaguaçu da Bahia | 291535 | 2915353 | 14170 | 2295.4 | 1821.7 | 1995.8 | 1562.8 | 1204 |  |
| 2002BA | 29Bahia | Itaju do Colônia | 291540 | 2915403 | 7188 | 1164.4 | 924.1 | 1012.4 | 792.8 | 1194 |  |
| 2003BA | 29Bahia | Itajuípe | 291550 | 2915502 | 21280.9 | 3447.3 | 2735.9 | 2997.4 | 2347.1 | 9006 |  |
| 2004BA | 29Bahia | Itamaraju | 291560 | 2915601 | 65439.4 | 10600.5 | 8412.9 | 9217.2 | 7217.4 | 7021 |  |
| 2005BA | 29Bahia | Itamari | 291570 | 2915700 | 8199 | 1328.2 | 1054.1 | 1154.8 | 904.3 | 1212 |  |
| 2006BA | 29Bahia | Itambé | 291580 | 2915809 | 23359.1 | 3783.9 | 3003 | 3290.1 | 2576.3 | 2231 |  |
| 2007BA | 29Bahia | Itanagra | 291590 | 2915908 | 7452.1 | 1207.2 | 958 | 1049.6 | 821.9 | 804 |  |
| 2008BA | 29Bahia | Itanhém | 291600 | 2916005 | 20232.1 | 3277.4 | 2601 | 2849.7 | 2231.4 | 2703 |  |
| 2009BA | 29Bahia | Itaparica | 291610 | 2916104 | 21995.3 | 3563 | 2827.7 | 3098 | 2425.9 | 3431 |  |
| 2010BA | 29Bahia | Itapé | 291620 | 2916203 | 10112.1 | 1638.1 | 1300 | 1424.3 | 1115.3 | 2795 |  |
| 2011BA | 29Bahia | Itapebi | 291630 | 2916302 | 10632.3 | 1722.3 | 1366.9 | 1497.6 | 1172.7 | 2262 |  |
| 2012BA | 29Bahia | Itapetinga | 291640 | 2916401 | 73955 | 11980 | 9507.7 | 10416.6 | 8156.6 | 5934 |  |
| 2013BA | 29Bahia | Itapicuru | 291650 | 2916500 | 34814.7 | 5639.6 | 4475.8 | 4903.7 | 3839.8 | 3208 |  |
| 2014BA | 29Bahia | Itapitanga | 291660 | 2916609 | 10521.9 | 1704.4 | 1352.7 | 1482 | 1160.5 | 2199 |  |
| 2015BA | 29Bahia | Itaquara | 291670 | 2916708 | 8213 | 1330.4 | 1055.9 | 1156.8 | 905.8 | 1468 |  |
| 2016BA | 29Bahia | Itarantim | 291680 | 2916807 | 19566.2 | 3169.5 | 2515.4 | 2755.9 | 2158 | 2228 |  |
| 2017BA | 29Bahia | Itatim | 291685 | 2916856 | 14506.3 | 2349.9 | 1864.9 | 2043.2 | 1599.9 | 2569 |  |
| 2018BA | 29Bahia | Itiruçu | 291690 | 2916906 | 12956.6 | 2098.8 | 1665.7 | 1824.9 | 1429 | 2022 |  |
| 2019BA | 29Bahia | Itiúba | 291700 | 2917003 | 37092.4 | 6008.6 | 4768.6 | 5224.5 | 4091 | 6288 |  |
| 2020BA | 29Bahia | Itororó | 291710 | 2917102 | 20641.9 | 3343.8 | 2653.7 | 2907.4 | 2276.6 | 2842 |  |
| 2021BA | 29Bahia | Ituaçu | 291720 | 2917201 | 18949.5 | 3069.6 | 2436.1 | 2669 | 2090 | 2929 |  |
| 2022BA | 29Bahia | Ituberá | 291730 | 2917300 | 28272.5 | 4579.9 | 3634.7 | 3982.2 | 3118.2 | 5265 |  |
| 2023BA | 29Bahia | Iuiú | 291733 | 2917334 | 11140.1 | 1804.6 | 1432.2 | 1569.1 | 1228.7 | 2376 |  |
| 2024BA | 29Bahia | Jaborandi | 291735 | 2917359 | 8958.4 | 1451.2 | 1151.7 | 1261.8 | 988 | 5132 |  |
| 2025BA | 29Bahia | Jacaraci | 291740 | 2917409 | 14852.4 | 2405.9 | 1909.4 | 2092 | 1638.1 | 4442 |  |
| 2026BA | 29Bahia | Jacobina | 291750 | 2917508 | 81994.1 | 13282.2 | 10541.2 | 11548.9 | 9043.3 | 10129 |  |
| 2027BA | 29Bahia | Jaguaquara | 291760 | 2917607 | 53982.2 | 8744.6 | 6940 | 7603.4 | 5953.8 | 5859 |  |
| 2028BA | 29Bahia | Jaguarari | 291770 | 2917706 | 32632.4 | 5286.1 | 4195.2 | 4596.3 | 3599.1 | 8071 |  |
| 2029BA | 29Bahia | Jaguaripe | 291780 | 2917805 | 18054.9 | 2924.7 | 2321.1 | 2543 | 1991.3 | 2113 |  |
| 2030BA | 29Bahia | Jandaíra | 291790 | 2917904 | 10776.5 | 1745.7 | 1385.4 | 1517.9 | 1188.6 | 1223 |  |
| 2031BA | 29Bahia | Jequié | 291800 | 2918001 | 157632.8 | 25534.9 | 20265.3 | 22202.6 | 17385.6 | 15597 |  |
| 2032BA | 29Bahia | Jeremoabo | 291810 | 2918100 | 39999.3 | 6479.5 | 5142.3 | 5633.9 | 4411.6 | 5753 |  |
| 2033BA | 29Bahia | Jiquiriçá | 291820 | 2918209 | 14655.2 | 2374 | 1884.1 | 2064.2 | 1616.3 | 1997 |  |
| 2034BA | 29Bahia | Jitaúna | 291830 | 2918308 | 13007.2 | 2107 | 1672.2 | 1832.1 | 1434.6 | 1978 |  |
| 2035BA | 29Bahia | João Dourado | 291835 | 2918357 | 24432.3 | 3957.8 | 3141 | 3441.3 | 2694.7 | 2918 |  |
| 2036BA | 29Bahia | Juazeiro | 291840 | 2918407 | 212280.1 | 34387.3 | 27290.7 | 29899.7 | 23412.7 | 57088 | 27 |
| 2037BA | 29Bahia | Jucuruçu | 291845 | 2918456 | 9956.7 | 1612.9 | 1280 | 1402.4 | 1098.1 | 1238 |  |
| 2038BA | 29Bahia | Jussara | 291850 | 2918506 | 15521.9 | 2514.4 | 1995.5 | 2186.3 | 1711.9 | 2112 |  |
| 2039BA | 29Bahia | Jussari | 291855 | 2918555 | 6283.1 | 1017.8 | 807.8 | 885 | 693 | 1429 |  |
| 2040BA | 29Bahia | Jussiape | 291860 | 2918605 | 7273.5 | 1178.2 | 935.1 | 1024.5 | 802.2 | 1235 |  |
| 2041BA | 29Bahia | Lafaiete Coutinho | 291870 | 2918704 | 3913 | 633.9 | 503.1 | 551.1 | 431.6 | 828 |  |
| 2042BA | 29Bahia | Lagoa Real | 291875 | 2918753 | 15237.1 | 2468.3 | 1958.9 | 2146.2 | 1680.5 | 2443 |  |
| 2043BA | 29Bahia | Laje | 291880 | 2918803 | 23501.3 | 3807 | 3021.3 | 3310.2 | 2592 | 2858 |  |
| 2044BA | 29Bahia | Lajedão | 291890 | 2918902 | 3926.6 | 636.1 | 504.8 | 553.1 | 433.1 | 516 |  |
| 2045BA | 29Bahia | Lajedinho | 291900 | 2919009 | 3941.1 | 638.4 | 506.7 | 555.1 | 434.7 | 637 |  |
| 2046BA | 29Bahia | Lajedo do Tabocal | 291905 | 2919058 | 8632.6 | 1398.4 | 1109.8 | 1215.9 | 952.1 | 1375 |  |
| 2047BA | 29Bahia | Lamarão | 291910 | 2919108 | 9175.6 | 1486.4 | 1179.6 | 1292.4 | 1012 | 1496 |  |
| 2048BA | 29Bahia | Lapão | 291915 | 2919157 | 27024.2 | 4377.7 | 3474.2 | 3806.4 | 2980.5 | 2545 |  |
| 2049BA | 29Bahia | Lauro de Freitas | 291920 | 2919207 | 185144.3 | 29991.5 | 23802.2 | 26077.6 | 20419.9 | 42049 | 14 |
| 2050BA | 29Bahia | Lençóis | 291930 | 2919306 | 11142.6 | 1805 | 1432.5 | 1569.4 | 1228.9 | 1131 |  |

2

6

1

1

6

1

1

1

1

2

1

2

1

3

1

2

1

1

1

1

1

1

1

3

4

| 2051BA |  | Licínio de Almeida | 291940 | 2919405 | 12649.7 | 2049.1 | 1626.2 | 1781.7 | 1395.2 | 2894 |  |
| --- | --- | --- | --- | --- | --- | --- | --- | --- | --- | --- | --- |
| 2052BA | 29Bahia | Livramento de  Nossa Senhora | 291950 | 2919504 | 45049.3 | 7297.5 | 5791.5 | 6345.2 | 4968.6 | 7197 |  |
| 2053BA | 29Bahia | Luís Eduardo  Magalhães | 291955 | 2919553 | 75596.8 | 12245.9 | 9718.7 | 10647.8 | 8337.7 | 4432 |  |
| 2054BA | 29Bahia | Macajuba | 291960 | 2919603 | 11562.2 | 1873 | 1486.4 | 1628.5 | 1275.2 | 1185 |  |
| 2055BA | 29Bahia | Macarani | 291970 | 2919702 | 18285.5 | 2962.1 | 2350.8 | 2575.5 | 2016.7 | 2193 |  |
| 2056BA | 29Bahia | Macaúbas | 291980 | 2919801 | 49293.9 | 7985.1 | 6337.2 | 6943.1 | 5436.7 | 9583 |  |
| 2057BA | 29Bahia | Macururé | 291990 | 2919900 | 8150.9 | 1320.4 | 1047.9 | 1148.1 | 899 | 1062 |  |
| 2058BA | 29Bahia | Madre de Deus | 291992 | 2919926 | 19680.4 | 3188 | 2530.1 | 2772 | 2170.6 | 4696 |  |
| 2059BA | 29Bahia | Maetinga | 291995 | 2919959 | 5233.7 | 847.8 | 672.8 | 737.2 | 577.2 | 3051 |  |
| 2060BA | 29Bahia | Maiquinique | 292000 | 2920007 | 9741.2 | 1578 | 1252.3 | 1372.1 | 1074.4 | 1000 |  |
| 2061BA | 29Bahia | Mairi | 292010 | 2920106 | 19502.7 | 3159.2 | 2507.3 | 2747 | 2151 | 2685 |  |
| 2062BA | 29Bahia | Malhada | 292020 | 2920205 | 16904.6 | 2738.4 | 2173.3 | 2381 | 1864.4 | 2379 |  |
| 2063BA | 29Bahia | Malhada de  Pedras | 292030 | 2920304 | 8659.5 | 1402.8 | 1113.3 | 1219.7 | 955.1 | 2852 |  |
| 2064BA | 29Bahia | Manoel Vitorino | 292040 | 2920403 | 14151.3 | 2292.4 | 1819.3 | 1993.2 | 1560.8 | 2170 |  |
| 2065BA | 29Bahia | Mansidão | 292045 | 2920452 | 13408.1 | 2172 | 1723.7 | 1888.5 | 1478.8 | 1534 |  |
| 2066BA | 29Bahia | Maracás | 292050 | 2920502 | 23537.7 | 3812.9 | 3026 | 3315.3 | 2596 | 4274 |  |
| 2067BA | 29Bahia | Maragogipe | 292060 | 2920601 | 44826.8 | 7261.5 | 5762.9 | 6313.9 | 4944 | 7758 |  |
| 2068BA | 29Bahia | Maraú | 292070 | 2920700 | 20440.6 | 3311.2 | 2627.8 | 2879.1 | 2254.4 | 1996 |  |
| 2069BA | 29Bahia | Marcionílio Souza | 292080 | 2920809 | 10707.1 | 1734.4 | 1376.5 | 1508.1 | 1180.9 | 1224 |  |
| 2070BA | 29Bahia | Mascote | 292090 | 2920908 | 14592.6 | 2363.9 | 1876 | 2055.4 | 1609.4 | 2880 |  |
| 2071BA | 29Bahia | Mata de São João | 292100 | 2921005 | 44410.8 | 7194.1 | 5709.5 | 6255.3 | 4898.1 | 7630 |  |
| 2072BA | 29Bahia | Matina | 292105 | 2921054 | 11958.6 | 1937.2 | 1537.4 | 1684.4 | 1318.9 | 2505 |  |
| 2073BA | 29Bahia | Medeiros Neto | 292110 | 2921104 | 22752.8 | 3685.7 | 2925.1 | 3204.7 | 2509.4 | 2800 |  |
| 2074BA | 29Bahia | Miguel Calmon | 292120 | 2921203 | 26902.6 | 4358 | 3458.6 | 3789.2 | 2967.1 | 7875 |  |
| 2075BA | 29Bahia | Milagres | 292130 | 2921302 | 11244.9 | 1821.6 | 1445.6 | 1583.8 | 1240.2 | 1863 |  |
| 2076BA | 29Bahia | Mirangaba | 292140 | 2921401 | 17681.4 | 2864.2 | 2273.1 | 2490.4 | 1950.1 | 1859 |  |
| 2077BA | 29Bahia | Mirante | 292145 | 2921450 | 9772.4 | 1583 | 1256.3 | 1376.4 | 1077.8 | 1807 |  |
| 2078BA | 29Bahia | Monte Santo | 292150 | 2921500 | 52391.4 | 8486.9 | 6735.4 | 7379.3 | 5778.3 | 8913 |  |
| 2079BA | 29Bahia | Morpará | 292160 | 2921609 | 8666.9 | 1404 | 1114.2 | 1220.7 | 955.9 | 1850 |  |
| 2080BA | 29Bahia | Morro do Chapéu | 292170 | 2921708 | 36074.8 | 5843.8 | 4637.8 | 5081.1 | 3978.7 | 5603 |  |
| 2081BA | 29Bahia | Mortugaba | 292180 | 2921807 | 12315.6 | 1995 | 1583.3 | 1734.7 | 1358.3 | 4694 |  |
| 2082BA | 29Bahia | Mucugê | 292190 | 2921906 | 10060.2 | 1629.7 | 1293.3 | 1417 | 1109.6 | 1642 |  |
| 2083BA | 29Bahia | Mucuri | 292200 | 2922003 | 39802.9 | 6447.7 | 5117.1 | 5606.3 | 4389.9 | 2922 |  |
| 2084BA | 29Bahia | Mulungu do Morro | 292205 | 2922052 | 11892.3 | 1926.4 | 1528.9 | 1675 | 1311.6 | 2651 |  |
| 2085BA | 29Bahia | Mundo Novo | 292210 | 2922102 | 26239.5 | 4250.5 | 3373.4 | 3695.8 | 2894 | 2117 |  |
| 2086BA | 29Bahia | Muniz Ferreira | 292220 | 2922201 | 7576.4 | 1227.3 | 974 | 1067.1 | 835.6 | 1566 |  |
| 2087BA | 29Bahia | Muquém de São  Francisco | 292225 | 2922250 | 11141.3 | 1804.8 | 1432.3 | 1569.3 | 1228.8 | 1577 |  |
| 2088BA | 29Bahia | Muritiba | 292230 | 2922300 | 29883.5 | 4840.8 | 3841.8 | 4209.1 | 3295.9 | 6294 |  |
| 2089BA | 29Bahia | Mutuípe | 292240 | 2922409 | 22338.2 | 3618.6 | 2871.8 | 3146.3 | 2463.7 | 5147 |  |
| 2090BA | 29Bahia | Nazaré | 292250 | 2922508 | 28589 | 4631.1 | 3675.4 | 4026.8 | 3153.1 | 4428 |  |
| 2091BA | 29Bahia | Nilo Peçanha | 292260 | 2922607 | 13631 | 2208.1 | 1752.4 | 1919.9 | 1503.4 | 2738 |  |
| 2092BA | 29Bahia | Nordestina | 292265 | 2922656 | 13042.5 | 2112.8 | 1676.7 | 1837 | 1438.5 | 2992 |  |
| 2093BA | 29Bahia | Nova Canaã | 292270 | 2922706 | 16782.4 | 2718.6 | 2157.5 | 2363.8 | 1851 | 2394 |  |
| 2094BA | 29Bahia | Nova Fátima | 292273 | 2922730 | 7899.5 | 1279.6 | 1015.6 | 1112.6 | 871.2 | 3431 |  |
| 2095BA | 29Bahia | Nova Ibiá | 292275 | 2922755 | 6805.6 | 1102.4 | 874.9 | 958.6 | 750.6 | 1124 |  |
| 2096BA | 29Bahia | Nova Itarana | 292280 | 2922805 | 8025.8 | 1300.1 | 1031.8 | 1130.4 | 885.2 | 1304 |  |
| 2097BA | 29Bahia | Nova Redenção | 292285 | 2922854 | 8879.8 | 1438.4 | 1141.6 | 1250.7 | 979.4 | 1046 |  |
| 2098BA | 29Bahia | Nova Soure | 292290 | 2922904 | 25282.5 | 4095.5 | 3250.3 | 3561 | 2788.4 | 3334 |  |
| 2099BA | 29Bahia | Nova Viçosa | 292300 | 2923001 | 41940.5 | 6793.9 | 5391.9 | 5907.3 | 4625.7 | 3144 |  |
| 2100BA | 29Bahia | Novo Horizonte | 292303 | 2923035 | 11814.4 | 1913.8 | 1518.9 | 1664.1 | 1303 | 2526 |  |
| 2101BA | 29Bahia | Novo Triunfo | 292305 | 2923050 | 15595.5 | 2526.3 | 2005 | 2196.6 | 1720.1 | 1790 |  |
| 2102BA | 29Bahia | Olindina | 292310 | 2923100 | 26773.9 | 4337.1 | 3442.1 | 3771.1 | 2952.9 | 3180 |  |
| 2103BA | 29Bahia | Oliveira dos  Brejinhos | 292320 | 2923209 | 22299 | 3612.2 | 2866.8 | 3140.8 | 2459.4 | 3713 |  |
| 2104BA | 29Bahia | Ouriçangas | 292330 | 2923308 | 8631.8 | 1398.3 | 1109.7 | 1215.8 | 952 | 1911 |  |
| 2105BA | 29Bahia | Ourolândia | 292335 | 2923357 | 17339.3 | 2808.8 | 2229.1 | 2442.2 | 1912.4 | 1836 |  |
| 2106BA | 29Bahia | Palmas de Monte  Alto | 292340 | 2923407 | 21801.3 | 3531.6 | 2802.8 | 3070.7 | 2404.5 | 4297 |  |
| 2107BA | 29Bahia | Palmeiras | 292350 | 2923506 | 8917.2 | 1444.5 | 1146.4 | 1256 | 983.5 | 1175 |  |
| 2108BA | 29Bahia | Paramirim | 292360 | 2923605 | 21681.3 | 3512.2 | 2787.3 | 3053.8 | 2391.3 | 3194 |  |
| 2109BA | 29Bahia | Paratinga | 292370 | 2923704 | 31601.1 | 5119.1 | 4062.6 | 4451 | 3485.3 | 2608 |  |
| 2110BA | 29Bahia | Paripiranga | 292380 | 2923803 | 29090.6 | 4712.4 | 3739.9 | 4097.4 | 3208.4 | 5068 |  |
| 2111BA | 29Bahia | Pau Brasil | 292390 | 2923902 | 10638.5 | 1723.3 | 1367.7 | 1498.4 | 1173.3 | 2354 |  |
| 2112BA | 29Bahia | Paulo Afonso | 292400 | 2924009 | 115824.4 | 18762.4 | 14890.4 | 16313.9 | 12774.5 | 43806 | 14 |
| 2113BA | 29Bahia | Pé de Serra | 292405 | 2924058 | 14024.5 | 2271.8 | 1803 | 1975.4 | 1546.8 | 2766 |  |
| 2114BA | 29Bahia | Pedrão | 292410 | 2924108 | 7299.1 | 1182.4 | 938.4 | 1028.1 | 805 | 1067 |  |
| 2115BA | 29Bahia | Pedro Alexandre | 292420 | 2924207 | 17587.5 | 2849 | 2261 | 2477.2 | 1939.8 | 1252 |  |
| 2116BA | 29Bahia | Piatã | 292430 | 2924306 | 17915.9 | 2902.2 | 2303.3 | 2523.5 | 1976 | 3872 |  |
| 2117BA | 29Bahia | Pilão Arcado | 292440 | 2924405 | 34584.1 | 5602.3 | 4446.1 | 4871.2 | 3814.3 | 7422 |  |
| 2118BA | 29Bahia | Pindaí | 292450 | 2924504 | 16348.8 | 2648.3 | 2101.8 | 2302.7 | 1803.1 | 3697 |  |
| 2119BA | 29Bahia | Pindobaçu | 292460 | 2924603 | 20617.2 | 3339.8 | 2650.5 | 2903.9 | 2273.9 | 3505 |  |
| 2120BA | 29Bahia | Pintadas | 292465 | 2924652 | 10616.3 | 1719.7 | 1364.8 | 1495.3 | 1170.9 | 2156 |  |
| 2121BA | 29Bahia | Piraí do Norte | 292467 | 2924678 | 10082.7 | 1633.3 | 1296.2 | 1420.2 | 1112 | 936 |  |
| 2122BA | 29Bahia | Piripá | 292470 | 2924702 | 12041.8 | 1950.7 | 1548.1 | 1696.1 | 1328.1 | 3669 |  |
| 2123BA | 29Bahia | Piritiba | 292480 | 2924801 | 24174.3 | 3916 | 3107.8 | 3405 | 2666.2 | 3295 |  |
| 2124BA | 29Bahia | Planaltino | 292490 | 2924900 | 9275.7 | 1502.6 | 1192.5 | 1306.5 | 1023 | 1516 |  |
| 2125BA | 29Bahia | Planalto | 292500 | 2925006 | 25911 | 4197.3 | 3331.1 | 3649.6 | 2857.8 | 3761 |  |
| 2126BA | 29Bahia | Poções | 292510 | 2925105 | 47267.9 | 7656.9 | 6076.8 | 6657.7 | 5213.3 | 6051 |  |
| 2127BA | 29Bahia | Pojuca | 292520 | 2925204 | 36819.7 | 5964.4 | 4733.5 | 5186.1 | 4060.9 | 4592 |  |
| 2128BA | 29Bahia | Ponto Novo | 292525 | 2925253 | 15727.6 | 2547.7 | 2021.9 | 2215.2 | 1734.6 | 4018 |  |
| 2129BA | 29Bahia | Porto Seguro | 292530 | 2925303 | 140969.3 | 22835.6 | 18123 | 19855.6 | 15547.7 | 22865 |  |
| 2130BA | 29Bahia | Potiraguá | 292540 | 2925402 | 8832.5 | 1430.8 | 1135.5 | 1244.1 | 974.2 | 945 |  |
| 2131BA | 29Bahia | Prado | 292550 | 2925501 | 28537.6 | 4622.8 | 3668.8 | 4019.5 | 3147.5 | 2604 |  |
| 2132BA | 29Bahia | Presidente Dutra | 292560 | 2925600 | 14704.5 | 2382 | 1890.4 | 2071.1 | 1621.8 | 2316 |  |
| 2133BA | 29Bahia | Presidente Jânio  Quadros | 292570 | 2925709 | 13154.9 | 2131 | 1691.2 | 1852.9 | 1450.9 | 3309 |  |
| 2134BA | 29Bahia | Presidente  Tancredo Neves | 292575 | 2925758 | 26450.6 | 4284.7 | 3400.5 | 3725.6 | 2917.3 | 3229 |  |
| 2135BA | 29Bahia | Queimadas | 292580 | 2925808 | 25592.7 | 4145.8 | 3290.2 | 3604.7 | 2822.7 | 6487 |  |
| 2136BA | 29Bahia | Quijingue | 292590 | 2925907 | 28091.8 | 4550.6 | 3611.5 | 3956.7 | 3098.3 | 3215 |  |
| 2137BA | 29Bahia | Quixabeira | 292593 | 2925931 | 9550.5 | 1547.1 | 1227.8 | 1345.2 | 1053.3 | 2046 |  |
| 2138BA | 29Bahia | Rafael Jambeiro | 292595 | 2925956 | 23391.5 | 3789.2 | 3007.2 | 3294.7 | 2579.9 | 3228 |  |
| 2139BA | 29Bahia | Remanso | 292600 | 2926004 | 41100.2 | 6657.8 | 5283.8 | 5789 | 4533 | 12417 |  |
| 2140BA | 29Bahia | Retirolândia | 292610 | 2926103 | 13417.4 | 2173.5 | 1724.9 | 1889.8 | 1479.8 | 7117 |  |
| 2141BA | 29Bahia | Riachão das  Neves | 292620 | 2926202 | 22681.2 | 3674.1 | 2915.9 | 3194.7 | 2501.5 | 2832 |  |
| 2142BA | 29Bahia | Riachão do  Jacuípe | 292630 | 2926301 | 34196.5 | 5539.5 | 4396.3 | 4816.6 | 3771.6 | 9794 |  |
| 2143BA | 29Bahia | Riacho de  Santana | 292640 | 2926400 | 34330.3 | 5561.2 | 4413.5 | 4835.4 | 3786.3 | 4270 |  |
| 2144BA | 29Bahia | Ribeira do Amparo | 292650 | 2926509 | 14878.4 | 2410.2 | 1912.8 | 2095.6 | 1641 | 2116 |  |
| 2145BA | 29Bahia | Ribeira do Pombal | 292660 | 2926608 | 51284.5 | 8307.6 | 6593.1 | 7223.4 | 5656.3 | 5887 |  |
| 2146BA | 29Bahia | Ribeirão do Largo | 292665 | 2926657 | 8126.5 | 1316.4 | 1044.7 | 1144.6 | 896.3 | 900 |  |
| 2147BA | 29Bahia | Rio de Contas | 292670 | 2926707 | 13294 | 2153.5 | 1709.1 | 1872.5 | 1466.2 | 1949 |  |
| 2148BA | 29Bahia | Rio do Antônio | 292680 | 2926806 | 15351.5 | 2486.8 | 1973.6 | 2162.3 | 1693.1 | 2546 |  |
| 2149BA | 29Bahia | Rio do Pires | 292690 | 2926905 | 11951.5 | 1936 | 1536.5 | 1683.4 | 1318.2 | 2136 |  |
| 2150BA | 29Bahia | Rio Real | 292700 | 2927002 | 39796.8 | 6446.7 | 5116.3 | 5605.4 | 4389.3 | 4088 |  |
| 2151BA | 29Bahia | Rodelas | 292710 | 2927101 | 8726 | 1413.5 | 1121.8 | 1229.1 | 962.4 | 1411 |  |
| 2152BA | 29Bahia | Ruy Barbosa | 292720 | 2927200 | 31081.5 | 5034.9 | 3995.8 | 4377.8 | 3428 | 4797 |  |

1

2

2

1

2

1

1

1

1

1

1

2

1

1

1

1

5

1

4

2

2

1

| 2153BA | 29Bahia | Salinas da  Margarida | 292730 | 2927309 | 14904.9 | 2414.4 | 1916.2 | 2099.4 | 1643.9 | 4625 |  |
| --- | --- | --- | --- | --- | --- | --- | --- | --- | --- | --- | --- |
| 2154BA | 29Bahia | Salvador | 292740 | 2927408 | 2840967.9 | 460208.4 | 365234.8 | 400151.2 | 313335 | 750112 | 262 |
| 2155BA | 29Bahia | Santa Bárbara | 292750 | 2927507 | 20364.3 | 3298.8 | 2618 | 2868.3 | 2246 | 3597 |  |
| 2156BA | 29Bahia | Santa Brígida | 292760 | 2927606 | 14872 | 2409.1 | 1911.9 | 2094.7 | 1640.3 | 2545 |  |
| 2157BA | 29Bahia | Santa Cruz  Cabrália | 292770 | 2927705 | 27580.8 | 4467.8 | 3545.8 | 3884.8 | 3041.9 | 3851 |  |
| 2158BA | 29Bahia | Santa Cruz da  Vitória | 292780 | 2927804 | 6620.9 | 1072.5 | 851.2 | 932.6 | 730.2 | 1976 |  |
| 2159BA | 29Bahia | Santa Inês | 292790 | 2927903 | 10787.6 | 1747.5 | 1386.9 | 1519.4 | 1189.8 | 1774 |  |
| 2160BA | 29Bahia | Santaluz | 292800 | 2928000 | 36337.5 | 5886.3 | 4671.5 | 5118.1 | 4007.7 | 13499 |  |
| 2161BA | 29Bahia | Santa Luzia | 292805 | 2928059 | 13271.9 | 2149.9 | 1706.2 | 1869.4 | 1463.8 | 2138 |  |
| 2162BA | 29Bahia | Santa Maria da  Vitória | 292810 | 2928109 | 40945.4 | 6632.7 | 5263.9 | 5767.2 | 4515.9 | 10493 |  |
| 2163BA | 29Bahia | Santana | 292820 | 2928208 | 26400.1 | 4276.6 | 3394 | 3718.5 | 2911.7 | 11573 |  |
| 2164BA | 29Bahia | Santanópolis | 292830 | 2928307 | 9099.4 | 1474 | 1169.8 | 1281.7 | 1003.6 | 2146 |  |
| 2165BA | 29Bahia | Santa Rita de  Cássia | 292840 | 2928406 | 27980.5 | 4532.6 | 3597.2 | 3941.1 | 3086 | 3450 |  |
| 2166BA | 29Bahia | Santa Teresinha | 292850 | 2928505 | 10273.5 | 1664.2 | 1320.8 | 1447 | 1133.1 | 1883 |  |
| 2167BA | 29Bahia | Santo Amaro | 292860 | 2928604 | 60171.4 | 9747.2 | 7735.6 | 8475.2 | 6636.4 | 6181 |  |
| 2168BA | 29Bahia | Santo Antônio de  Jesus | 292870 | 2928703 | 98554.3 | 15964.8 | 12670.1 | 13881.4 | 10869.7 | 19321 |  |
| 2169BA | 29Bahia | Santo Estêvão | 292880 | 2928802 | 51589.1 | 8356.9 | 6632.3 | 7266.3 | 5689.8 | 5088 |  |
| 2170BA | 29Bahia | São Desidério | 292890 | 2928901 | 31514.7 | 5105.1 | 4051.5 | 4438.9 | 3475.8 | 3065 |  |
| 2171BA | 29Bahia | São Domingos | 292895 | 2928950 | 9416.9 | 1525.4 | 1210.6 | 1326.4 | 1038.6 | 4726 |  |
| 2172BA | 29Bahia | São Félix | 292900 | 2929008 | 14756.9 | 2390.5 | 1897.1 | 2078.5 | 1627.6 | 5151 |  |
| 2173BA | 29Bahia | São Félix do  Coribe | 292905 | 2929057 | 14834 | 2403 | 1907.1 | 2089.4 | 1636.1 | 5297 |  |
| 2174BA | 29Bahia | São Felipe | 292910 | 2929107 | 21163.6 | 3428.3 | 2720.8 | 2980.9 | 2334.2 | 3736 |  |
| 2175BA | 29Bahia | São Francisco do  Conde | 292920 | 2929206 | 37511.6 | 6076.5 | 4822.5 | 5283.5 | 4137.2 | 3435 |  |
| 2176BA | 29Bahia | São Gabriel | 292925 | 2929255 | 19063.9 | 3088.2 | 2450.9 | 2685.2 | 2102.6 | 2514 |  |
| 2177BA | 29Bahia | São Gonçalo dos  Campos | 292930 | 2929305 | 36292.7 | 5879.1 | 4665.8 | 5111.8 | 4002.8 | 5414 |  |
| 2178BA | 29Bahia | São José da  Vitória | 292935 | 2929354 | 5895.6 | 955 | 757.9 | 830.4 | 650.2 | 1530 |  |
| 2179BA | 29Bahia | São José do  Jacuípe | 292937 | 2929370 | 10610.9 | 1718.9 | 1364.1 | 1494.5 | 1170.3 | 2345 |  |
| 2180BA | 29Bahia | São Miguel das  Matas | 292940 | 2929404 | 11387.2 | 1844.6 | 1463.9 | 1603.9 | 1255.9 | 1490 |  |
| 2181BA | 29Bahia | São Sebastião do  Passé | 292950 | 2929503 | 44277.4 | 7172.5 | 5692.3 | 6236.5 | 4883.4 | 8650 |  |
| 2182BA | 29Bahia | Sapeaçu | 292960 | 2929602 | 17367.6 | 2813.4 | 2232.8 | 2446.2 | 1915.5 | 4819 |  |
| 2183BA | 29Bahia | Sátiro Dias | 292970 | 2929701 | 19552.4 | 3167.3 | 2513.7 | 2754 | 2156.5 | 1924 |  |
| 2184BA | 29Bahia | Saubara | 292975 | 2929750 | 11902.2 | 1928 | 1530.1 | 1676.4 | 1312.7 | 2076 |  |
| 2185BA | 29Bahia | Saúde | 292980 | 2929800 | 12625 | 2045.1 | 1623.1 | 1778.2 | 1392.4 | 2025 |  |
| 2186BA | 29Bahia | Seabra | 292990 | 2929909 | 43990.5 | 7126 | 5655.4 | 6196.1 | 4851.8 | 7963 |  |
| 2187BA | 29Bahia | Sebastião  Laranjeiras | 293000 | 2930006 | 11179.8 | 1811 | 1437.3 | 1574.7 | 1233 | 2311 |  |
| 2188BA | 29Bahia | Senhor do Bonfim | 293010 | 2930105 | 78678 | 12745 | 10114.8 | 11081.8 | 8677.5 | 14898 |  |
| 2189BA | 29Bahia | Serra do Ramalho | 293015 | 2930154 | 32280.5 | 5229.1 | 4150 | 4546.7 | 3560.3 | 4887 |  |
| 2190BA | 29Bahia | Sento Sé | 293020 | 2930204 | 40154.4 | 6504.6 | 5162.2 | 5655.8 | 4428.7 | 11143 |  |
| 2191BA | 29Bahia | Serra Dourada | 293030 | 2930303 | 18090.5 | 2930.5 | 2325.7 | 2548.1 | 1995.2 | 4530 |  |
| 2192BA | 29Bahia | Serra Preta | 293040 | 2930402 | 15426.6 | 2499 | 1983.2 | 2172.8 | 1701.4 | 3260 |  |
| 2193BA | 29Bahia | Serrinha | 293050 | 2930501 | 80642.7 | 13063.3 | 10367.4 | 11358.5 | 8894.2 | 10078 |  |
| 2194BA | 29Bahia | Serrolândia | 293060 | 2930600 | 13148.2 | 2129.9 | 1690.3 | 1851.9 | 1450.1 | 2188 |  |
| 2195BA | 29Bahia | Simões Filho | 293070 | 2930709 | 129202.5 | 20929.5 | 16610.3 | 18198.2 | 14250 | 15367 |  |
| 2196BA | 29Bahia | Sítio do Mato | 293075 | 2930758 | 12887.6 | 2087.7 | 1656.8 | 1815.2 | 1421.4 | 1390 |  |
| 2197BA | 29Bahia | Sítio do Quinto | 293076 | 2930766 | 11599.7 | 1879 | 1491.3 | 1633.8 | 1279.3 | 2680 |  |
| 2198BA | 29Bahia | Sobradinho | 293077 | 2930774 | 23005.3 | 3726.6 | 2957.6 | 3240.3 | 2537.3 | 5345 |  |
| 2199BA | 29Bahia | Souto Soares | 293080 | 2930808 | 16840.3 | 2728 | 2165 | 2372 | 1857.3 | 3721 |  |
| 2200BA | 29Bahia | Tabocas do Brejo  Velho | 293090 | 2930907 | 12445.4 | 2016 | 1600 | 1752.9 | 1372.6 | 2153 |  |
| 2201BA | 29Bahia | Tanhaçu | 293100 | 2931004 | 20724.6 | 3357.2 | 2664.4 | 2919.1 | 2285.7 | 4194 |  |
| 2202BA | 29Bahia | Tanque Novo | 293105 | 2931053 | 17180.6 | 2783.1 | 2208.7 | 2419.9 | 1894.9 | 2848 |  |
| 2203BA | 29Bahia | Tanquinho | 293110 | 2931103 | 8193.2 | 1327.2 | 1053.3 | 1154 | 903.6 | 1605 |  |
| 2204BA | 29Bahia | Taperoá | 293120 | 2931202 | 20404.4 | 3305.3 | 2623.2 | 2874 | 2250.4 | 3367 |  |
| 2205BA | 29Bahia | Tapiramutá | 293130 | 2931301 | 17133.6 | 2775.5 | 2202.7 | 2413.3 | 1889.7 | 2436 |  |
| 2206BA | 29Bahia | Teixeira de Freitas | 293135 | 2931350 | 152933.4 | 24773.7 | 19661.1 | 21540.7 | 16867.3 | 30358 | 10 |
| 2207BA | 29Bahia | Teodoro Sampaio | 293140 | 2931400 | 7850.3 | 1271.7 | 1009.2 | 1105.7 | 865.8 | 1848 |  |
| 2208BA | 29Bahia | Teofilândia | 293150 | 2931509 | 22511.7 | 3646.7 | 2894.1 | 3170.8 | 2482.9 | 6365 |  |
| 2209BA | 29Bahia | Teolândia | 293160 | 2931608 | 14959.2 | 2423.2 | 1923.2 | 2107 | 1649.9 | 2336 |  |
| 2210BA | 29Bahia | Terra Nova | 293170 | 2931707 | 13206.4 | 2139.3 | 1697.8 | 1860.1 | 1456.6 | 2738 |  |
| 2211BA | 29Bahia | Tremedal | 293180 | 2931806 | 17524.5 | 2838.8 | 2252.9 | 2468.3 | 1932.8 | 3916 |  |
| 2212BA | 29Bahia | Tucano | 293190 | 2931905 | 53173.7 | 8613.6 | 6836 | 7489.5 | 5864.6 | 5025 |  |
| 2213BA | 29Bahia | Uauá | 293200 | 2932002 | 24835.2 | 4023.1 | 3192.8 | 3498 | 2739.1 | 9801 |  |
| 2214BA | 29Bahia | Ubaíra | 293210 | 2932101 | 20504.3 | 3321.5 | 2636 | 2888 | 2261.5 | 3112 |  |
| 2215BA | 29Bahia | Ubaitaba | 293220 | 2932200 | 20380.8 | 3301.5 | 2620.2 | 2870.6 | 2247.8 | 4528 |  |
| 2216BA | 29Bahia | Ubatã | 293230 | 2932309 | 26567.7 | 4303.7 | 3415.5 | 3742.1 | 2930.2 | 2314 |  |
| 2217BA | 29Bahia | Uibaí | 293240 | 2932408 | 14097.8 | 2283.7 | 1812.4 | 1985.7 | 1554.9 | 2262 |  |
| 2218BA | 29Bahia | Umburanas | 293245 | 2932457 | 18531.3 | 3001.9 | 2382.4 | 2610.1 | 2043.8 | 1418 |  |
| 2219BA | 29Bahia | Una | 293250 | 2932507 | 21979.5 | 3560.5 | 2825.7 | 3095.8 | 2424.2 | 4555 |  |
| 2220BA | 29Bahia | Urandi | 293260 | 2932606 | 16924.2 | 2741.6 | 2175.8 | 2383.8 | 1866.6 | 4518 |  |
| 2221BA | 29Bahia | Uruçuca | 293270 | 2932705 | 20963.2 | 3395.8 | 2695 | 2952.7 | 2312.1 | 5422 |  |
| 2222BA | 29Bahia | Utinga | 293280 | 2932804 | 19115.5 | 3096.5 | 2457.5 | 2692.4 | 2108.3 | 1730 |  |
| 2223BA | 29Bahia | Valença | 293290 | 2932903 | 94782.2 | 15353.8 | 12185.2 | 13350.1 | 10453.7 | 21483 |  |
| 2224BA | 29Bahia | Valente | 293300 | 2933000 | 27124.4 | 4393.9 | 3487.1 | 3820.5 | 2991.6 | 17553 |  |
| 2225BA | 29Bahia | Várzea da Roça | 293305 | 2933059 | 14299.1 | 2316.3 | 1838.3 | 2014 | 1577.1 | 2053 |  |
| 2226BA | 29Bahia | Várzea do Poço | 293310 | 2933109 | 9137.3 | 1480.2 | 1174.7 | 1287 | 1007.8 | 1660 |  |
| 2227BA | 29Bahia | Várzea Nova | 293315 | 2933158 | 13197.1 | 2137.8 | 1696.6 | 1858.8 | 1455.5 | 2265 |  |
| 2228BA | 29Bahia | Varzedo | 293317 | 2933174 | 9187.7 | 1488.3 | 1181.2 | 1294.1 | 1013.3 | 1468 |  |
| 2229BA | 29Bahia | Vera Cruz | 293320 | 2933208 | 41349 | 6698.1 | 5315.8 | 5824 | 4560.4 | 5620 |  |
| 2230BA | 29Bahia | Vereda | 293325 | 2933257 | 6617.4 | 1072 | 850.7 | 932.1 | 729.8 | 854 |  |
| 2231BA | 29Bahia | Vitória da  Conquista | 293330 | 2933307 | 332856.4 | 53919.4 | 42792 | 46882.9 | 36711.3 | 52433 | 14 |
| 2232BA | 29Bahia | Wagner | 293340 | 2933406 | 9409.5 | 1524.2 | 1209.7 | 1325.3 | 1037.8 | 1029 |  |
| 2233BA | 29Bahia | Wanderley | 293345 | 2933455 | 12684.7 | 2054.8 | 1630.7 | 1786.6 | 1399 | 4037 |  |
| 2234BA | 29Bahia | Wenceslau  Guimarães | 293350 | 2933505 | 22148.2 | 3587.8 | 2847.4 | 3119.6 | 2442.8 | 2534 |  |
| 2235BA | 29Bahia | Xique-Xique | 293360 | 2933604 | 47098.3 | 7629.5 | 6055 | 6633.8 | 5194.5 | 4458 |  |
| 2236MG |  | Abadia dos  Dourados | 310010 | 3100104 | 6920.2 | 1344.5 | 950 | 969 | 687.6 | 1688 |  |
| 2237MG |  | Abaeté | 310020 | 3100203 | 23227 | 4512.8 | 3188.6 | 3252.4 | 2307.9 | 6354 |  |
| 2238MG |  | Abre Campo | 310030 | 3100302 | 13543.6 | 2631.4 | 1859.3 | 1896.4 | 1345.7 | 2096 |  |
| 2239MG |  | Acaiaca | 310040 | 3100401 | 4003.2 | 777.8 | 549.6 | 560.5 | 397.8 | 1737 |  |
| 2240MG |  | Açucena | 310050 | 3100500 | 10031.3 | 1949 | 1377.1 | 1404.6 | 996.7 | 2752 |  |
| 2241MG |  | Água Boa | 310060 | 3100609 | 14579.8 | 2832.7 | 2001.5 | 2041.5 | 1448.7 | 2555 |  |
| 2242MG |  | Água Comprida | 310070 | 3100708 | 2038.4 | 396 | 279.8 | 285.4 | 202.5 | 458 |  |
| 2243MG |  | Aguanil | 310080 | 3100807 | 4305 | 836.4 | 591 | 602.8 | 427.8 | 1786 |  |

1

1

4

3

3

1

5

1

1

1

1

1

2

1

2

4

1

4

1

2

4

2

1

4

1

1

6

6

1

1

2244MG	Águas Formosas	310090	3100906	19047.5	3700.7	2614.8	2667.1	1892.6	3838

2245MG	Águas Vermelhas	310100	3101003	13259.5	2576.2	1820.3	1856.7	1317.5	1852

2246MG	Aimorés	310110	3101102	25367.2	4928.6	3482.4	3552	2520.6	6002

2247MG	Aiuruoca	310120	3101201	6165.5	1197.9	846.4	863.3	612.6	2007

2248MG	Alagoa	310130	3101300	2726.4	529.7	374.3	381.8	270.9	1107

2249MG	Albertina	310140	3101409	2992.8	581.5	410.9	419.1	297.4	827

2250MG	Além Paraíba	310150	3101508	35248	6848.3	4838.8	4935.6	3502.4	8567

2251MG	Alfenas	310160	3101607	77578.7	15072.8	10650	10862.9	7708.5	30012	10

Alfredo

2252MG	Vasconcelos	310163	3101631	6536.5	1270	897.3	915.3	649.5	2693

2253MG	Almenara	310170	3101706	40705.4	7908.7	5588	5699.8	4044.6	7221

Minas

2254MG	31Gerais	Alpercata	310180	3101805	7378.4	1433.5	1012.9	1033.2	733.1	1732

Minas

2255MG	31Gerais	Alpinópolis	310190	3101904	19351.6	3759.8	2656.6	2709.7	1922.8	9283

Minas

2256MG	31Gerais	Alterosa	310200	3102001	14235.6	2765.8	1954.3	1993.3	1414.5	4298

Minas

2257MG	31Gerais	Alto Caparaó	310205	3102050	5618.1	1091.5	771.3	786.7	558.2	1839

Minas

2258MG	31Gerais	Alto Rio Doce	310210	3102100	11774.4	2287.6	1616.4	1648.7	1169.9	3190

Minas

2259MG	31Gerais	Alvarenga	310220	3102209	4251.2	826	583.6	595.3	422.4	1383

Minas

2260MG	31Gerais	Alvinópolis	310230	3102308	15425	2996.9	2117.5	2159.9	1532.7	4853

Minas

2261MG	31Gerais	Alvorada de Minas	310240	3102407	3618	702.9	496.7	506.6	359.5	1192

Minas

2262MG	31Gerais	Amparo do Serra	310250	3102506	4930.9	958	676.9	690.4	490	2006

Minas

2263MG	31Gerais	Andradas	310260	3102605	39499.6	7674.4	5422.5	5530.9	3924.8	11939

Minas	Cachoeira de

2264MG	31Gerais	Pajeú	310270	3102704	9280.9	1803.2	1274.1	1299.6	922.2	1651

Minas

2265MG	31Gerais	Andrelândia	310280	3102803	12348.9	2399.3	1695.3	1729.2	1227	3892

Minas

2266MG	31Gerais	Angelândia	310285	3102852	8342.2	1620.8	1145.2	1168.1	828.9	1862

Minas

2267MG	31Gerais	Antônio Carlos	310290	3102902	11406.8	2216.2	1565.9	1597.2	1133.4	3402

Minas

2268MG	31Gerais	Antônio Dias	310300	3103009	9569.8	1859.3	1313.7	1340	950.9	2410

Minas	Antônio Prado de

2269MG	31Gerais	Minas	310310	3103108	1658.1	322.2	227.6	232.2	164.8	1024

Minas

2270MG	31Gerais	Araçaí	310320	3103207	2320	450.8	318.5	324.9	230.5	961

Minas

2271MG	31Gerais	Aracitaba	310330	3103306	2085.7	405.2	286.3	292	207.2	1688

Minas

2272MG	31Gerais	Araçuaí	310340	3103405	36785.8	7147.1	5050	5150.9	3655.2	6113

Minas

2273MG	31Gerais	Araguari	310350	3103504	114632.9	22272	15736.8	16051.4	11390.3	28776

Minas

2274MG	31Gerais	Arantina	310360	3103603	2845.2	552.8	390.6	398.4	282.7	1220

Minas

1

2

3

1

1

1

3

1

1

1

6

5

4

1

1

1

3

6

2

1

2275MG	31Gerais	Araponga	310370	3103702	8387.4	1629.6	1151.4	1174.4	833.4	3557

Minas

2276MG	31Gerais	Araporã	310375	3103751	6556.4	1273.8	900.1	918.1	651.5	1026

Minas

2277MG	31Gerais	Arapuá	310380	3103801	2837.2	551.2	389.5	397.3	281.9	1055

Minas

2278MG	31Gerais	Araújos	310390	3103900	8625.5	1675.8	1184.1	1207.8	857.1	1501

Minas

2279MG	31Gerais	Araxá	310400	3104007	100660	19557.2	13818.6	14094.9	10001.9	16498

Minas

2280MG	31Gerais	Arceburgo	310410	3104106	10212.9	1984.3	1402	1430.1	1014.8	2800

Minas

2281MG	31Gerais	Arcos	310420	3104205	38674	7514	5309.2	5415.3	3842.8	13449

Minas

2282MG	31Gerais	Areado	310430	3104304	14523.4	2821.8	1993.8	2033.6	1443.1	4338

Minas

2283MG	31Gerais	Argirita	310440	3104403	2857.3	555.1	392.3	400.1	283.9	1000

Minas

2284MG	31Gerais	Aricanduva	310445	3104452	5043	979.8	692.3	706.1	501.1	1439

Minas

2285MG	31Gerais	Arinos	310450	3104502	17988.1	3494.9	2469.4	2518.8	1787.4	2187

Minas

2286MG	31Gerais	Astolfo Dutra	310460	3104601	13735.7	2668.7	1885.6	1923.3	1364.8	3594

Minas

2287MG	31Gerais	Ataléia	310470	3104700	13900.5	2700.7	1908.3	1946.4	1381.2	3056

Minas

2288MG	31Gerais	Augusto de Lima	310480	3104809	4980.1	967.6	683.7	697.3	494.8	2372

Minas

2289MG	31Gerais	Baependi	310490	3104908	18925.7	3677.1	2598.1	2650.1	1880.5	6222

Minas

2290MG	31Gerais	Baldim	310500	3105004	7971.9	1548.9	1094.4	1116.3	792.1	2624

Minas

2291MG	31Gerais	Bambuí	310510	3105103	23525.3	4570.7	3229.6	3294.1	2337.6	7110

Minas

2292MG	31Gerais	Bandeira	310520	3105202	4960.9	963.9	681	694.6	492.9	1380

Minas

2293MG	31Gerais	Bandeira do Sul	310530	3105301	5593.7	1086.8	767.9	783.3	555.8	1246

Minas

2294MG	31Gerais	Barão de Cocais	310540	3105400	30754.1	5975.2	4221.9	4306.3	3055.8	11299

Minas	Barão de Monte

2295MG	31Gerais	Alto	310550	3105509	5642.2	1096.2	774.6	790	560.6	1897

Minas

2296MG	31Gerais	Barbacena	310560	3105608	132972	25835.1	18254.4	18619.4	13212.5	26149

Minas

2297MG	31Gerais	Barra Longa	310570	3105707	5750.6	1117.3	789.4	805.2	571.4	2006

2298MG	Barroso	310590	3105905	20404.9	3964.5	2801.2	2857.2	2027.5	8363

Bela Vista de

2299MG	Minas	310600	3106002	10245.1	1990.5	1406.4	1434.6	1018	3274

2300MG	Belmiro Braga	310610	3106101	3457.1	671.7	474.6	484.1	343.5	1531

2301MG	Belo Horizonte	310620	3106200	2468029.7	479513.5	338811.1	345585.4	245231.5	559433	171

2302MG	Belo Oriente	310630	3106309	25219.9	4900	3462.2	3531.4	2505.9	5177

2303MG	Belo Vale	310640	3106408	7713.6	1498.7	1058.9	1080.1	766.4	2414

2304MG	Berilo	310650	3106507	12283.5	2386.6	1686.3	1720	1220.5	2315

2305MG	Bertópolis	310660	3106606	4603.2	894.4	631.9	644.6	457.4	1516

2306MG	Berizal	310665	3106655	4594.1	892.6	630.7	643.3	456.5	1529

2307MG	Betim	310670	3106705	410773.1	79809.1	56390.9	57518.4	40815.8	72122	21

2308MG	Bias Fortes	310680	3106804	3648.7	708.9	500.9	510.9	362.5	1167

2309MG	Bicas	310690	3106903	14212.9	2761.4	1951.1	1990.2	1412.2	3854

2310MG	Biquinhas	310700	3107000	2609.9	507.1	358.3	365.5	259.3	816

2311MG	Boa Esperança	310710	3107109	39743.7	7721.8	5456	5565.1	3949.1	16444

2312MG	31Gerais	Bocaina de Minas	310720	3107208	5107.9	992.4	701.2	715.2	507.5	597

Minas

2313MG	31Gerais	Bocaiúva	310730	3107307	48868.6	9494.7	6708.7	6842.8	4855.7	8735

Minas

2314MG	31Gerais	Bom Despacho	310740	3107406	48501.7	9423.4	6658.3	6791.4	4819.3	12000

Minas	Bom Jardim de

2315MG	31Gerais	Minas	310750	3107505	6570	1276.5	901.9	920	652.8	1427

Minas	Bom Jesus da

2316MG	31Gerais	Penha	310760	3107604	4089.3	794.5	561.4	572.6	406.3	4331

Minas	Bom Jesus do

2317MG	31Gerais	Amparo	310770	3107703	5834.2	1133.5	800.9	816.9	579.7	1177

Minas	Bom Jesus do

Gerais	Galho

2318MG	31	310780	3107802	15357.4	2983.8	2108.3	2150.4	1526	2641

Minas

2319MG	31Gerais	Bom Repouso	310790	3107901	10629.9	2065.3	1459.3	1488.4	1056.2	2788

Minas

2320MG	31Gerais	Bom Sucesso	310800	3108008	17625.7	3424.5	2419.7	2468	1751.3	3923

Minas

2321MG	31Gerais	Bonfim	310810	3108107	6926.7	1345.8	950.9	969.9	688.3	3144

Minas	Bonfinópolis de

2322MG	31Gerais	Minas	310820	3108206	5766.4	1120.4	791.6	807.4	573	1102

Minas

2323MG	31Gerais	Bonito de Minas	310825	3108255	10509.7	2041.9	1442.8	1471.6	1044.3	1110

Minas

2324MG	31Gerais	Borda da Mata	310830	3108305	18394.1	3573.8	2525.1	2575.6	1827.7	4535

Minas

2325MG	31Gerais	Botelhos	310840	3108404	15130.5	2939.7	2077.1	2118.6	1503.4	3100

Minas

2326MG	31Gerais	Botumirim	310850	3108503	6496	1262.1	891.8	909.6	645.5	1398

Minas	Brasilândia de

2327MG	31Gerais	Minas	310855	3108552	15474.9	3006.6	2124.4	2166.9	1537.6	2254

Minas

2328MG	31Gerais	Brasília de Minas	310860	3108602	32128.7	6242.3	4410.6	4498.8	3192.4	4598

Minas

2329MG	31Gerais	Brás Pires	310870	3108701	4554.1	884.8	625.2	637.7	452.5	1949

Minas

2330MG	31Gerais	Braúnas	310880	3108800	4987.1	968.9	684.6	698.3	495.5	1348

Minas

2331MG	31Gerais	Brumadinho	310900	3109006	37237.4	7234.9	5112	5214.2	3700	11544

Minas

2332MG	31Gerais	Bueno Brandão	310910	3109105	11079	2152.5	1520.9	1551.3	1100.8	2265

Minas

2333MG	31Gerais	Buenópolis	310920	3109204	10454.5	2031.2	1435.2	1463.9	1038.8	3231

Minas

2334MG	31Gerais	Bugre	310925	3109253	4071.2	791	558.9	570.1	404.5	1546

Minas

2335MG	31Gerais	Buritis	310930	3109303	23996.1	4662.2	3294.2	3360	2384.3	2901

Minas

2336MG	31Gerais	Buritizeiro	310940	3109402	27783.6	5398.1	3814.1	3890.4	2760.7	4412

1

4

2

3

1

1

1

1

3

1

1

1

2

1

4

1

1

1

Minas

2337MG	31Gerais	Cabeceira Grande	310945	3109451	6763.3	1314	928.5	947	672	841

Minas

2338MG	31Gerais	Cabo Verde	310950	3109501	14112.5	2741.9	1937.4	1976.1	1402.3	3840

Minas	Cachoeira da

2339MG	31Gerais	Prata	310960	3109600	3676	714.2	504.6	514.7	365.3	1610

Minas	Cachoeira de

2340MG	31Gerais	Minas	310970	3109709	11409.9	2216.8	1566.4	1597.7	1133.7	3156

Minas	Cachoeira

2341MG	31Gerais	Dourada	310980	3109808	2623.1	509.6	360.1	367.3	260.6	883

Minas

2342MG	31Gerais	Caetanópolis	310990	3109907	10996.8	2136.6	1509.6	1539.8	1092.7	2592

Minas

2343MG	31Gerais	Caeté	311000	3110004	43097.3	8373.4	5916.4	6034.7	4282.3	5848

Minas

2344MG	31Gerais	Caiana	311010	3110103	5274.7	1024.8	724.1	738.6	524.1	2240

Minas

2345MG	31Gerais	Cajuri	311020	3110202	4070.4	790.8	558.8	570	404.4	2989

Minas

2346MG	31Gerais	Caldas	311030	3110301	14195.8	2758.1	1948.8	1987.8	1410.5	2913

Minas

2347MG	31Gerais	Camacho	311040	3110400	3076.1	597.7	422.3	430.7	305.7	1284

Minas

2348MG	31Gerais	Camanducaia	311050	3110509	21663.6	4209	2974	3033.4	2152.6	2895

Minas

2349MG	31Gerais	Cambuí	311060	3110608	28237.8	5486.3	3876.5	3954	2805.8	5333

Minas

2350MG	31Gerais	Cambuquira	311070	3110707	12857.9	2498.2	1765.1	1800.4	1277.6	2986

Minas

2351MG	31Gerais	Campanário	311080	3110806	3681.8	715.3	505.4	515.5	365.8	960

Minas

2352MG	31Gerais	Campanha	311090	3110905	16196.3	3146.8	2223.4	2267.9	1609.3	3720

Minas

2353MG	31Gerais	Campestre	311100	3111002	21115.6	4102.5	2898.7	2956.7	2098.1	7108

Minas

2354MG	31Gerais	Campina Verde	311110	3111101	19760.9	3839.3	2712.8	2767	1963.5	5094

Minas

2355MG	31Gerais	Campo Azul	311115	3111150	3791.6	736.7	520.5	530.9	376.7	1225

Minas

2356MG	31Gerais	Campo Belo	311120	3111200	53339.6	10363.4	7322.5	7468.9	5300	16493

Minas

Gerais

2357MG	31	Campo do Meio	311130	3111309	11702.7	2273.7	1606.5	1638.7	1162.8	3653

Minas

2358MG	31Gerais	Campo Florido	311140	3111408	7549.1	1466.7	1036.3	1057.1	750.1	818

2359MG	Campos Altos	311150	3111507	14965.5	2907.6	2054.5	2095.5	1487	2988

2360MG	Campos Gerais	311160	3111606	28489	5535.1	3911	3989.2	2830.8	4379

2361MG	Canaã	311170	3111705	4655.8	904.6	639.1	651.9	462.6	2094

2362MG	Canápolis	311180	3111804	11844.8	2301.3	1626.1	1658.6	1176.9	3272

2363MG	Cana Verde	311190	3111903	5665	1100.7	777.7	793.2	562.9	1674

2364MG	Candeias	311200	3112000	14912.2	2897.3	2047.1	2088.1	1481.7	5319

2365MG	Cantagalo	311205	3112059	4400.3	854.9	604.1	616.2	437.2	1191

2366MG	Caparaó	311210	3112109	5380.3	1045.3	738.6	753.4	534.6	1626

2367MG	Capela Nova	311220	3112208	4767.3	926.2	654.5	667.5	473.7	2266

2368MG	Capelinha	311230	3112307	36748.5	7139.9	5044.8	5145.7	3651.5	7624

2369MG	Capetinga	311240	3112406	7099.3	1379.3	974.6	994.1	705.4	3174

2370MG	Capim Branco	311250	3112505	9396.7	1825.7	1290	1315.8	933.7	2171

2371MG	Capinópolis	311260	3112604	15889.8	3087.2	2181.4	2225	1578.9	6351

2372MG	Capitão Andrade	311265	3112653	5238	1017.7	719.1	733.4	520.5	1318

2373MG	Capitão Enéas	311270	3112703	14860.3	2887.2	2040	2080.8	1476.6	2491

2374MG	Capitólio	311280	3112802	8493.5	1650.2	1166	1189.3	843.9	5016

2375MG	Caputira	311290	3112901	9267.5	1800.6	1272.2	1297.7	920.8	1341

2376MG	Caraí	311300	3113008	23244	4516.1	3190.9	3254.7	2309.6	3644

Minas

2377MG	31Gerais	Caranaíba	311310	3113107	3280.2	637.3	450.3	459.3	325.9	900

Minas

2378MG	31Gerais	Carandaí	311320	3113206	24648	4788.9	3383.7	3451.3	2449.1	10892

Minas

2379MG	31Gerais	Carangola	311330	3113305	33026.7	6416.8	4533.9	4624.6	3281.6	13265

Minas

2380MG	31Gerais	Caratinga	311340	3113404	89471.4	17383.4	12282.6	12528.2	8890.2	21676

Minas

2381MG	31Gerais	Carbonita	311350	3113503	9381.8	1822.8	1287.9	1313.7	932.2	2866

Minas

2382MG	31Gerais	Careaçu	311360	3113602	6589.4	1280.3	904.6	922.7	654.7	1648

Minas

2383MG	31Gerais	Carlos Chagas	311370	3113701	19749.5	3837.1	2711.2	2765.4	1962.4	5251

Minas

2384MG	31Gerais	Carmésia	311380	3113800	2562.8	497.9	351.8	358.9	254.6	745

Minas	Carmo da

2385MG	31Gerais	Cachoeira	311390	3113909	12139.3	2358.5	1666.5	1699.8	1206.2	2582

Minas

2386MG	31Gerais	Carmo da Mata	311400	3114006	11317.9	2199	1553.7	1584.8	1124.6	1913

Minas

2387MG	31Gerais	Carmo de Minas	311410	3114105	14435.6	2804.7	1981.7	2021.3	1434.4	3546

Minas

2388MG	31Gerais	Carmo do Cajuru	311420	3114204	21404.7	4158.7	2938.4	2997.2	2126.8	4945

Minas	Carmo do

2389MG	31Gerais	Paranaíba	311430	3114303	30382.1	5902.9	4170.9	4254.2	3018.9	10108

Minas	Carmo do Rio

2390MG	31Gerais	Claro	311440	3114402	21051.6	4090.1	2890	2947.7	2091.8	8803

Minas	Carmópolis de

2391MG	31Gerais	Minas	311450	3114501	18330.5	3561.4	2516.4	2566.7	1821.4	6374

Minas

2392MG	31Gerais	Carneirinho	311455	3114550	9847	1913.2	1351.8	1378.8	978.4	1966

Minas

2393MG	31Gerais	Carrancas	311460	3114600	4042.9	785.5	555	566.1	401.7	1484

Minas

2394MG	31Gerais	Carvalhópolis	311470	3114709	3493.3	678.7	479.6	489.1	347.1	1256

Minas

2395MG	31Gerais	Carvalhos	311480	3114808	4576.9	889.2	628.3	640.9	454.8	953

Minas

Gerais

2396MG	31	Casa Grande	311490	3114907	2278.5	442.7	312.8	319	226.4	1167

Minas

2397MG	31Gerais	Cascalho Rico	311500	3115003	2993.7	581.6	411	419.2	297.5	681

2

1

2

1

3

4

5

1

1

1

2

2

2

2

6

1

1

2

1

1

1

1

Minas

2398MG	31Gerais	Cássia	311510	3115102	17781.1	3454.7	2441	2489.8	1766.8	8480

Minas	Conceição da

2399MG	31Gerais	Barra de Minas	311520	3115201	4003	777.7	549.5	560.5	397.8	1114

Minas

2400MG	31Gerais	Cataguases	311530	3115300	73115	14205.5	10037.2	10237.9	7264.9	22178

Minas

2401MG	31Gerais	Catas Altas	311535	3115359	5152.2	1001	707.3	721.4	511.9	1897

Minas	Catas Altas da

2402MG	31Gerais	Noruega	311540	3115409	3588.3	697.2	492.6	502.5	356.5	846

Minas

2403MG	31Gerais	Catuji	311545	3115458	6608.2	1283.9	907.2	925.3	656.6	1933

Minas

2404MG	31Gerais	Catuti	311547	3115474	5112.2	993.2	701.8	715.8	508	1608

Minas

2405MG	31Gerais	Caxambu	311550	3115508	21953.6	4265.4	3013.8	3074	2181.4	6235

Minas

2406MG	31Gerais	Cedro do Abaeté	311560	3115607	1203.7	233.9	165.2	168.5	119.6	416

Minas

2407MG	31Gerais	Central de Minas	311570	3115706	6977.2	1355.6	957.8	977	693.3	1965

Minas

2408MG	31Gerais	Centralina	311580	3115805	10459.5	2032.2	1435.9	1464.6	1039.3	2337

Minas

2409MG	31Gerais	Chácara	311590	3115904	2994.9	581.9	411.1	419.4	297.6	1879

Minas

2410MG	31Gerais	Chalé	311600	3116001	5743	1115.8	788.4	804.2	570.6	1295

Minas

2411MG	31Gerais	Chapada do Norte	311610	3116100	15456.7	3003.1	2121.9	2164.3	1535.8	3524

Minas

2412MG	31Gerais	Chapada Gaúcha	311615	3116159	12271.3	2384.2	1684.6	1718.3	1219.3	1674

Minas

2413MG	31Gerais	Chiador	311620	3116209	2774.3	539	380.9	388.5	275.7	1133

Minas

2414MG	31Gerais	Cipotânea	311630	3116308	6740.5	1309.6	925.3	943.8	669.8	1842

Minas

2415MG	31Gerais	Claraval	311640	3116407	4734.7	919.9	650	663	470.5	1978

Minas

2416MG	31Gerais	Claro dos Poções	311650	3116506	7768.6	1509.4	1066.5	1087.8	771.9	3139

Minas

2417MG	31Gerais	Cláudio	311660	3116605	27411.3	5325.7	3763	3838.3	2723.7	6638

Minas

2418MG	31Gerais	Coimbra	311670	3116704	7374.5	1432.8	1012.4	1032.6	732.8	3839

Minas

2419MG	31Gerais	Coluna	311680	3116803	9066.8	1761.6	1244.7	1269.6	900.9	1719

Minas	Comendador

2420MG	31Gerais	Gomes	311690	3116902	3073.9	597.2	422	430.4	305.4	770

Minas

2421MG	31Gerais	Comercinho	311700	3117009	7768.4	1509.3	1066.4	1087.8	771.9	1714

Minas	Conceição da

2422MG	31Gerais	Aparecida	311710	3117108	10161.5	1974.3	1395	1422.9	1009.7	3804

Minas	Conceição das

2423MG	31Gerais	Pedras	311720	3117207	2812.5	546.4	386.1	393.8	279.5	1123

Minas	Conceição das

2424MG	31Gerais	Alagoas	311730	3117306	25577.9	4969.5	3511.3	3581.5	2541.5	4075

Minas	Conceição de

2425MG	31Gerais	Ipanema	311740	3117405	4566.4	887.2	626.9	639.4	453.7	833

Minas	Conceição do

2426MG	31Gerais	Mato Dentro	311750	3117504	18003.3	3497.9	2471.5	2520.9	1788.9	2508

Minas	Conceição do

2427MG	31Gerais	Pará	311760	3117603	5383.8	1046	739.1	753.9	535	1970

Minas	Conceição do Rio

2428MG	31Gerais	Verde	311770	3117702	13430.1	2609.3	1843.7	1880.5	1334.5	3172 Conceição dos

2429MG	Ouros	311780	3117801	11095.5	2155.7	1523.2	1553.6	1102.5	2393

2430MG	Cônego Marinho	311783	3117836	7446.1	1446.7	1022.2	1042.6	739.9	1538

2431MG	Confins	311787	3117876	6378.3	1239.2	875.6	893.1	633.8	1363

2432MG	Congonhal	311790	3117900	11285.5	2192.7	1549.3	1580.2	1121.4	3157

2433MG	Congonhas	311800	3118007	52017.6	10106.5	7141	7283.8	5168.6	11160

Congonhas do

2434MG	Norte	311810	3118106	5052.3	981.6	693.6	707.4	502	1812

Gerais

2435MG	31	Conquista	311820	3118205	6799	1321	933.4	952	675.6	1239

Minas	Conselheiro

2436MG	31Gerais	Lafaiete	311830	3118304	123563.5	24007.2	16962.8	17302	12277.7	51670	15

Minas

2437MG	31Gerais	Conselheiro Pena	311840	3118403	22834.8	4436.6	3134.8	3197.4	2268.9	3472

Minas

2438MG	31Gerais	Consolação	311850	3118502	1776	345.1	243.8	248.7	176.5	488

Minas

2439MG	31Gerais	Contagem	311860	3118601	639147.9	124180	87742.2	89496.6	63507.8	129263	38

Minas

2440MG	31Gerais	Coqueiral	311870	3118700	9345.1	1815.7	1282.9	1308.5	928.6	3458

Minas

2441MG	31Gerais	Coração de Jesus	311880	3118809	26622.4	5172.5	3654.7	3727.8	2645.3	4887

Minas

2442MG	31Gerais	Cordisburgo	311890	3118908	8879.2	1725.1	1218.9	1243.3	882.3	2371

Minas

2443MG	31Gerais	Cordislândia	311900	3119005	3525.6	685	484	493.7	350.3	602

Minas

2444MG	31Gerais	Corinto	311910	3119104	24129	4688	3312.4	3378.7	2397.5	7092

Minas

2445MG	31Gerais	Coroaci	311920	3119203	10269.2	1995.2	1409.8	1437.9	1020.4	1759

Minas

2446MG	31Gerais	Coromandel	311930	3119302	28089.2	5457.5	3856.1	3933.2	2791	7051

Minas

2447MG	31Gerais	Coronel Fabriciano	311940	3119401	107845.5	20953.3	14805	15101	10715.9	23258

Minas

2448MG	31Gerais	Coronel Murta	311950	3119500	9279.5	1802.9	1273.9	1299.4	922	1919

Minas

2449MG	31Gerais	Coronel Pacheco	311960	3119609	3068	596.1	421.2	429.6	304.8	1717

Minas	Coronel Xavier

2450MG	31Gerais	Chaves	311970	3119708	3404.2	661.4	467.3	476.7	338.3	1327

Minas

2451MG	31Gerais	Córrego Danta	311980	3119807	3352.3	651.3	460.2	469.4	333.1	1412

Minas	Córrego do Bom

2452MG	31Gerais	Jesus	311990	3119906	3764.3	731.4	516.8	527.1	374	1153

Minas

2453MG	31Gerais	Córrego Fundo	311995	3119955	6116	1188.3	839.6	856.4	607.7	2389

Minas

2454MG	31Gerais	Córrego Novo	312000	3120003	3001.7	583.2	412.1	420.3	298.3	1150

Couto de

Minas	Magalhães de

2455MG	31Gerais	Minas	312010	3120102	4351.9	845.5	597.4	609.4	432.4	1425

Minas

2456MG	31Gerais	Crisólita	312015	3120151	6427.2	1248.7	882.3	900	638.6	1540

Minas

2457MG	31Gerais	Cristais	312020	3120201	12127.5	2356.3	1664.9	1698.2	1205	2535

Minas

2458MG	31Gerais	Cristália	312030	3120300	5929.9	1152.1	814.1	830.3	589.2	1227

Minas

2

1

1

1

6

1

7

1

4

2

2459MG	31Gerais	Cristiano Otoni	312040	3120409	5135.9	997.9	705.1	719.2	510.3	2237

Minas

2460MG	31Gerais	Cristina	312050	3120508	10352.1	2011.3	1421.1	1449.6	1028.6	2184

Minas

2461MG	31Gerais	Crucilândia	312060	3120607	4944.7	960.7	678.8	692.4	491.3	1969

Minas	Cruzeiro da

2462MG	31Gerais	Fortaleza	312070	3120706	4030.8	783.1	553.3	564.4	400.5	917

Minas

2463MG	31Gerais	Cruzília	312080	3120805	15156.3	2944.7	2080.7	2122.3	1506	6207

Minas

2464MG	31Gerais	Cuparaque	312083	3120839	4878.5	947.8	669.7	683.1	484.7	1100

Minas

2465MG	31Gerais	Curral de Dentro	312087	3120870	7377.6	1433.4	1012.8	1033	733.1	1698

Minas

2466MG	31Gerais	Curvelo	312090	3120904	77809.7	15117.6	10681.7	10895.3	7731.4	26599

Minas

2467MG	31Gerais	Datas	312100	3121001	5368.4	1043	737	751.7	533.4	1793

Minas

2468MG	31Gerais	Delfim Moreira	312110	3121100	8096.4	1573	1111.5	1133.7	804.5	2042

Minas

2469MG	31Gerais	Delfinópolis	312120	3121209	7047	1369.2	967.4	986.8	700.2	4526

Minas

2470MG	31Gerais	Delta	312125	3121258	9322.3	1811.2	1279.8	1305.4	926.3	1422

Minas

2471MG	31Gerais	Descoberto	312130	3121308	4941	960	678.3	691.9	491	1853

Minas	Desterro de Entre

2472MG	31Gerais	Rios	312140	3121407	7201.3	1399.1	988.6	1008.4	715.5	2864

Minas

2473MG	31Gerais	Desterro do Melo	312150	3121506	3000.6	583	411.9	420.2	298.1	1051

Minas

2474MG	31Gerais	Diamantina	312160	3121605	47307.6	9191.4	6494.4	6624.2	4700.6	13805

Minas	Diogo de

2475MG	31Gerais	Vasconcelos	312170	3121704	3875	752.9	532	542.6	385	1712

Minas

2476MG	31Gerais	Dionísio	312180	3121803	8380.6	1628.3	1150.5	1173.5	832.7	2336

Minas

2477MG	31Gerais	Divinésia	312190	3121902	3391.3	658.9	465.6	474.9	337	1985

Minas

2478MG	31Gerais	Divino	312200	3122009	19742.2	3835.7	2710.2	2764.4	1961.6	6791

Minas	Divino das

2479MG	31Gerais	Laranjeiras	312210	3122108	5017.8	974.9	688.8	702.6	498.6	1084

Minas	Divinolândia de

2480MG	31Gerais	Minas	312220	3122207	7365.4	1431	1011.1	1031.3	731.9	1743

Minas

2481MG	31Gerais	Divinópolis	312230	3122306	227359.1	44173.6	31211.9	31835.9	22591.1	53468	16

Minas

2482MG	31Gerais	Divisa Alegre	312235	3122355	6375.3	1238.7	875.2	892.7	633.5	717

Minas

2483MG	31Gerais	Divisa Nova	312240	3122405	5949.7	1156	816.8	833.1	591.2	1896

Minas

2484MG	31Gerais	Divisópolis	312245	3122454	10033.2	1949.4	1377.4	1404.9	996.9	1496

Minas

2485MG	31Gerais	Dom Bosco	312247	3122470	3798.5	738	521.5	531.9	377.4	582

Minas

2486MG	31Gerais	Dom Cavati	312250	3122504	5210.9	1012.4	715.4	729.7	517.8	1251

Minas

2487MG	31Gerais	Dom Joaquim	312260	3122603	4532.8	880.7	622.3	634.7	450.4	1509

Minas

2488MG	31Gerais	Dom Silvério	312270	3122702	5280	1025.9	724.8	739.3	524.6	2202

Minas

2489MG	31Gerais	Dom Viçoso	312280	3122801	3034.7	589.6	416.6	424.9	301.5	870

2490MG	Dona Eusébia	312290	3122900	6340.9	1232	870.5	887.9	630.1	2004

2491MG	Dores de Campos	312300	3123007	9811	1906.2	1346.9	1373.8	974.9	2417

Dores de

2492MG	Guanhães	312310	3123106	5263.2	1022.6	722.5	737	523	1598

2493MG	Dores do Indaiá	312320	3123205	13813.9	2683.9	1896.4	1934.3	1372.6	4580

2494MG	Dores do Turvo	312330	3123304	4423.5	859.4	607.3	619.4	439.5	1902

2495MG	Doresópolis	312340	3123403	1498.3	291.1	205.7	209.8	148.9	742

2496MG	Douradoquara	312350	3123502	1894.8	368.1	260.1	265.3	188.3	579

2497MG	Durandé	312352	3123528	7710.1	1498	1058.4	1079.6	766.1	1908

2498MG	Elói Mendes	312360	3123601	26858.6	5218.4	3687.1	3760.9	2668.8	8439

Engenheiro

2499MG	Caldas	312370	3123700	10804.3	2099.2	1483.2	1512.9	1073.6	2643

Minas	Engenheiro

2500MG	31Gerais	Navarro	312380	3123809	7266.7	1411.8	997.6	1017.5	722	2133

Minas

2501MG	31Gerais	Entre Folhas	312385	3123858	5324.3	1034.5	730.9	745.5	529	1112

Minas	Entre Rios de

2502MG	31Gerais	Minas	312390	3123908	14909.6	2896.8	2046.8	2087.7	1481.5	5485

Minas

2503MG	31Gerais	Ervália	312400	3124005	18609.7	3615.7	2554.7	2605.8	1849.1	6493

Minas

2504MG	31Gerais	Esmeraldas	312410	3124104	66038	12830.5	9065.7	9247	6561.8	7993

Minas

2505MG	31Gerais	Espera Feliz	312420	3124203	24112.3	4684.8	3310.1	3376.3	2395.9	10149

Minas

2506MG	31Gerais	Espinosa	312430	3124302	31735.7	6165.9	4356.7	4443.8	3153.4	6009

Minas	Espírito Santo do

2507MG	31Gerais	Dourado	312440	3124401	4606	894.9	632.3	645	457.7	1356

Minas

2508MG	31Gerais	Estiva	312450	3124500	11216.5	2179.3	1539.8	1570.6	1114.5	1916

Minas

2509MG	31Gerais	Estrela Dalva	312460	3124609	2442.5	474.6	335.3	342	242.7	566

Minas

2510MG	31Gerais	Estrela do Indaiá	312470	3124708	3551.8	690.1	487.6	497.3	352.9	1079

Minas

2511MG	31Gerais	Estrela do Sul	312480	3124807	7785.4	1512.6	1068.8	1090.1	773.6	1124

Minas

2512MG	31Gerais	Eugenópolis	312490	3124906	11012.1	2139.5	1511.7	1542	1094.2	3705

Minas	Ewbank da

2513MG	31Gerais	Câmara	312500	3125002	3874.3	752.7	531.9	542.5	385	1950

Minas

2514MG	31Gerais	Extrema	312510	3125101	32488.3	6312.2	4460	4549.2	3228.1	5880

Minas

2515MG	31Gerais	Fama	312520	3125200	2391.9	464.7	328.4	334.9	237.7	917

Minas

2516MG	31Gerais	Faria Lemos	312530	3125309	3356.1	652.1	460.7	469.9	333.5	1193

Minas

2517MG	31Gerais	Felício dos Santos	312540	3125408	5026.4	976.6	690	703.8	499.4	2002

Minas	São Gonçalo do

2518MG	31Gerais	Rio Preto	312550	3125507	3145.9	611.2	431.9	440.5	312.6	1538

Minas

2519MG	31Gerais	Felisburgo	312560	3125606	7232.2	1405.1	992.8	1012.7	718.6	1532

Minas

1

1

1

2

2

3

1

1

1

4

1

1

2

1

2520MG	31Gerais	Felixlândia	312570	3125705	14860.5	2887.2	2040	2080.8	1476.6	5273

Minas	Fernandes

2521MG	31Gerais	Tourinho	312580	3125804	3253.3	632.1	446.6	455.5	323.3	936

Minas

2522MG	31Gerais	Ferros	312590	3125903	10501.6	2040.4	1441.7	1470.5	1043.5	1644

Minas

2523MG	31Gerais	Fervedouro	312595	3125952	10783.1	2095	1480.3	1509.9	1071.4	3470

Minas

2524MG	31Gerais	Florestal	312600	3126000	7085.7	1376.7	972.7	992.2	704.1	1606

Minas

2525MG	31Gerais	Formiga	312610	3126109	67126.2	13041.9	9215.1	9399.3	6669.9	18446

Minas

2526MG	31Gerais	Formoso	312620	3126208	8920.8	1733.2	1224.6	1249.1	886.4	909

Minas	Fortaleza de

2527MG	31Gerais	Minas	312630	3126307	4294.9	834.5	589.6	601.4	426.8	3808

Minas

2528MG	31Gerais	Fortuna de Minas	312640	3126406	2851.1	553.9	391.4	399.2	283.3	1288

Minas

2529MG	31Gerais	Francisco Badaró	312650	3126505	10415.3	2023.6	1429.8	1458.4	1034.9	2950

Minas

2530MG	31Gerais	Francisco Dumont	312660	3126604	5086.8	988.3	698.3	712.3	505.4	1336

Minas

2531MG	31Gerais	Francisco Sá	312670	3126703	25855.7	5023.5	3549.5	3620.4	2569.1	5226

Minas

2532MG	31Gerais	Franciscópolis	312675	3126752	5682.9	1104.1	780.1	795.7	564.7	1269

Minas

2533MG	31Gerais	Frei Gaspar	312680	3126802	5952.9	1156.6	817.2	833.6	591.5	1565

Minas

2534MG	31Gerais	Frei Inocêncio	312690	3126901	9351.7	1816.9	1283.8	1309.5	929.2	2105

Minas

2535MG	31Gerais	Frei Lagonegro	312695	3126950	3439.8	668.3	472.2	481.7	341.8	908

Minas

2536MG	31Gerais	Fronteira	312700	3127008	16097.9	3127.7	2209.9	2254.1	1599.5	2156

Minas	Fronteira dos

2537MG	31Gerais	Vales	312705	3127057	4696.6	912.5	644.7	657.6	466.7	1431

Minas

2538MG	31Gerais	Fruta de Leite	312707	3127073	5749.8	1117.1	789.3	805.1	571.3	996

Minas

2539MG	31Gerais	Frutal	312710	3127107	56928.4	11060.6	7815.1	7971.4	5656.6	10823

Minas

2540MG	31Gerais	Funilândia	312720	3127206	4132	802.8	567.2	578.6	410.6	1293

Minas

2541MG	31Gerais	Galiléia	312730	3127305	6975.9	1355.3	957.7	976.8	693.1	1247

2542MG	Gameleiras	312733	3127339	5189.4	1008.2	712.4	726.6	515.6	1598

2543MG	Glaucilândia	312735	3127354	3086.3	599.6	423.7	432.2	306.7	976

2544MG	Goiabeira	312737	3127370	3230.8	627.7	443.5	452.4	321	1117

2545MG	Goianá	312738	3127388	3846.8	747.4	528.1	538.6	382.2	1976

2546MG	Gonçalves	312740	3127404	4333	841.9	594.8	606.7	430.5	1766

2547MG	Gonzaga	312750	3127503	6104.7	1186.1	838.1	854.8	606.6	1299

2548MG	Gouveia	312760	3127602	11893.6	2310.8	1632.8	1665.4	1181.8	4179

Governador

2549MG	Valadares	312770	3127701	274490.1	53330.7	37682	38435.4	27274.2	56007	16

2550MG	Grão Mogol	312780	3127800	15587.9	3028.6	2139.9	2182.7	1548.9	3146

2551MG	Grupiara	312790	3127909	1397.6	271.5	191.9	195.7	138.9	750

2552MG	Guanhães	312800	3128006	33069.3	6425	4539.8	4630.5	3285.9	8660

2553MG	Guapé	312810	3128105	14218.2	2762.5	1951.9	1990.9	1412.8	4995

2554MG	Guaraciaba	312820	3128204	10397.7	2020.2	1427.4	1455.9	1033.1	4617

2555MG	Guaraciama	312825	3128253	4894.2	950.9	671.9	685.3	486.3	2652

2556MG	Guaranésia	312830	3128303	19090.3	3709.1	2620.7	2673.1	1896.9	6022

2557MG	Guarani	312840	3128402	8894.9	1728.2	1221.1	1245.5	883.8	4614

2558MG	Guarará	312850	3128501	3916.8	761	537.7	548.4	389.2	1227

2559MG	Guarda-Mor	312860	3128600	6653.7	1292.7	913.4	931.7	661.1	1785

2560MG	Guaxupé	312870	3128709	51200.8	9947.8	7028.8	7169.4	5087.5	14367

Minas

2561MG	31Gerais	Guidoval	312880	3128808	7237.4	1406.2	993.6	1013.4	719.1	3189

Minas

2562MG	31Gerais	Guimarânia	312890	3128907	7714.6	1498.9	1059.1	1080.2	766.5	2435

Minas

2563MG	31Gerais	Guiricema	312900	3129004	8670.3	1684.6	1190.3	1214.1	861.5	3255

Minas

2564MG	31Gerais	Gurinhatã	312910	3129103	5983.1	1162.5	821.4	837.8	594.5	1723

Minas

2565MG	31Gerais	Heliodora	312920	3129202	6400.4	1243.5	878.6	896.2	636	2133

Minas

2566MG	31Gerais	Iapu	312930	3129301	10729	2084.5	1472.9	1502.3	1066.1	3375

Minas

2567MG	31Gerais	Ibertioga	312940	3129400	5092	989.3	699	713	506	1576

Minas

2568MG	31Gerais	Ibiá	312950	3129509	24426.2	4745.8	3353.2	3420.3	2427.1	4924

Minas

2569MG	31Gerais	Ibiaí	312960	3129608	8194.5	1592.1	1124.9	1147.4	814.2	1435

Minas

2570MG	31Gerais	Ibiracatu	312965	3129657	6078.8	1181.1	834.5	851.2	604	1327

Minas

2571MG	31Gerais	Ibiraci	312970	3129707	13097.4	2544.7	1798	1834	1301.4	5649

Minas

2572MG	31Gerais	Ibirité	312980	3129806	171089.2	33240.9	23487.1	23956.7	17000	28578	10

Minas

2573MG	31Gerais	Ibitiúra de Minas	312990	3129905	3473.5	674.9	476.8	486.4	345.1	1609

Minas

2574MG	31Gerais	Ibituruna	313000	3130002	2959.1	574.9	406.2	414.3	294	906

Minas

2575MG	31Gerais	Icaraí de Minas	313005	3130051	11457.3	2226	1572.9	1604.3	1138.4	1568

Minas

2576MG	31Gerais	Igarapé	313010	3130101	39082.4	7593.3	5365.2	5472.5	3883.4	7437

Minas

2577MG	31Gerais	Igaratinga	313020	3130200	10119.1	1966	1389.2	1416.9	1005.5	2172

Minas

2578MG	31Gerais	Iguatama	313030	3130309	8091.2	1572	1110.8	1133	804	3198

Minas

2579MG	31Gerais	Ijaci	313040	3130408	6252.4	1214.8	858.3	875.5	621.3	1408

Minas

2580MG	31Gerais	Ilicínea	313050	3130507	12042.6	2339.8	1653.2	1686.3	1196.6	4733

Minas

2581MG	31Gerais	Imbé de Minas	313055	3130556	6726.2	1306.8	923.4	941.8	668.3	1546

2

1

1

1

1

3

1

1

1

2

2

1

2

1

1

7

5

1

2

2

1

2

2

1

Minas

2582MG	31Gerais	Inconfidentes	313060	3130606	7188.8	1396.7	986.9	1006.6	714.3	2112

Minas

2583MG	31Gerais	Indaiabira	313065	3130655	7431.3	1443.8	1020.2	1040.6	738.4	2221

Minas

2584MG	31Gerais	Indianópolis	313070	3130705	6592.4	1280.8	905	923.1	655	1660

Minas

2585MG	31Gerais	Ingaí	313080	3130804	2725.8	529.6	374.2	381.7	270.8	1111

Minas

2586MG	31Gerais	Inhapim	313090	3130903	24527	4765.4	3367.1	3434.4	2437.1	7945

Minas

2587MG	31Gerais	Inhaúma	313100	3131000	6068.8	1179.1	833.1	849.8	603	1860

Minas

2588MG	31Gerais	Inimutaba	313110	3131109	7248.8	1408.4	995.1	1015	720.3	3179

Minas

2589MG	31Gerais	Ipaba	313115	3131158	17796.4	3457.7	2443.1	2491.9	1768.3	3926

Minas

2590MG	31Gerais	Ipanema	313120	3131208	19180.4	3726.6	2633.1	2685.7	1905.8	4654

Minas

2591MG	31Gerais	Ipatinga	313130	3131307	253553.7	49262.9	34807.9	35503.8	25193.9	83179	23

Minas

2592MG	31Gerais	Ipiaçu	313140	3131406	4211.9	818.3	578.2	589.8	418.5	1207

Minas

2593MG	31Gerais	Ipuiúna	313150	3131505	9898.8	1923.2	1358.9	1386.1	983.6	1662

Minas

2594MG	31Gerais	Iraí de Minas	313160	3131604	6788.2	1318.9	931.9	950.5	674.5	1009

Minas

2595MG	31Gerais	Itabira	313170	3131703	115916.8	22521.5	15913.1	16231.2	11517.9	22485

Minas

2596MG	31Gerais	Itabirinha	313180	3131802	11205.6	2177.1	1538.3	1569.1	1113.4	2560

Minas

2597MG	31Gerais	Itabirito	313190	3131901	48992.8	9518.8	6725.7	6860.2	4868.1	15156

Minas

2598MG	31Gerais	Itacambira	313200	3132008	5234.4	1017	718.6	732.9	520.1	1496

Minas

2599MG	31Gerais	Itacarambi	313210	3132107	18142.2	3524.8	2490.6	2540.4	1802.7	2541

Minas

2600MG	31Gerais	Itaguara	313220	3132206	12983.6	2522.6	1782.4	1818	1290.1	4903

Minas

2601MG	31Gerais	Itaipé	313230	3132305	12391.4	2407.5	1701.1	1735.1	1231.2	2006

Minas

2602MG	31Gerais	Itajubá	313240	3132404	94669.2	18393.3	12996.2	13256	9406.6	16242

Minas

2603MG	31Gerais	Itamarandiba	313250	3132503	33763.3	6559.9	4635	4727.7	3354.8	9479

Minas

2604MG	31Gerais	Itamarati de Minas	313260	3132602	4257.6	827.2	584.5	596.2	423	1911

2605MG	Itambacuri	313270	3132701	23280.2	4523.1	3195.9	3259.8	2313.2	3452

Itambé do Mato

2606MG	Dentro	313280	3132800	2218.4	431	304.5	310.6	220.4	939

2607MG	Itamogi	313290	3132909	10406.1	2021.8	1428.5	1457.1	1034	6092

2608MG	Itamonte	313300	3133006	14908.7	2896.6	2046.7	2087.6	1481.4	6338

2609MG	Itanhandu	313310	3133105	14888.3	2892.6	2043.9	2084.7	1479.4	5920

2610MG	Itanhomi	313320	3133204	12176.6	2365.8	1671.6	1705	1209.9	3389

2611MG	Itaobim	313330	3133303	21292	4136.8	2923	2981.4	2115.6	4800

2612MG	Itapagipe	313340	3133402	14561.6	2829.2	1999	2039	1446.9	3594

2613MG	Itapecerica	313350	3133501	21822.6	4239.9	2995.8	3055.7	2168.4	5972

2614MG	Itapeva	313360	3133600	9291.1	1805.2	1275.5	1301	923.2	1891

2615MG	Itatiaiuçu	313370	3133709	10616.8	2062.7	1457.5	1486.6	1054.9	3716

2616MG	Itaú de Minas	313375	3133758	15670.6	3044.6	2151.3	2194.3	1557.1	8245

2617MG	Itaúna	313380	3133808	90123	17510	12372.1	12619.5	8954.9	15309

2618MG	Itaverava	313390	3133907	5695.2	1106.5	781.8	797.5	565.9	2259

2619MG	Itinga	313400	3134004	14856.9	2886.5	2039.6	2080.3	1476.2	2826

2620MG	Itueta	313410	3134103	6005.5	1166.8	824.4	840.9	596.7	2443

2621MG	Ituiutaba	313420	3134202	101860.6	19790.5	13983.4	14263	10121.2	30695	10

2622MG	31Gerais	Itumirim	313430	3134301	6161.9	1197.2	845.9	862.8	612.3	1781

Minas

2623MG	31Gerais	Iturama	313440	3134400	37113.8	7210.8	5095	5196.9	3687.7	6227

Minas

2624MG	31Gerais	Itutinga	313450	3134509	3903.8	758.5	535.9	546.6	387.9	1409

Minas

2625MG	31Gerais	Jaboticatubas	313460	3134608	18742	3641.4	2572.9	2624.3	1862.3	3249

Minas

2626MG	31Gerais	Jacinto	313470	3134707	12375.1	2404.4	1698.9	1732.8	1229.6	2462

Minas

2627MG	31Gerais	Jacuí	313480	3134806	7681.4	1492.4	1054.5	1075.6	763.2	4400

Minas

2628MG	31Gerais	Jacutinga	313490	3134905	24542.1	4768.3	3369.1	3436.5	2438.6	2155

Minas

2629MG	31Gerais	Jaguaraçu	313500	3135001	3093.5	601	424.7	433.2	307.4	1110

Minas

2630MG	31Gerais	Jaíba	313505	3135050	36471.2	7086	5006.8	5106.9	3623.9	3996

Minas

2631MG	31Gerais	Jampruca	313507	3135076	5286.4	1027.1	725.7	740.2	525.3	940

Minas

2632MG	31Gerais	Janaúba	313510	3135100	69882.6	13577.5	9593.5	9785.3	6943.8	11260

Minas

2633MG	31Gerais	Januária	313520	3135209	67337.4	13083	9244.1	9428.9	6690.9	5384

Minas

2634MG	31Gerais	Japaraíba	313530	3135308	4178.6	811.9	573.6	585.1	415.2	1061

Minas

2635MG	31Gerais	Japonvar	313535	3135357	8484.4	1648.4	1164.7	1188	843	2064

Minas

2636MG	31Gerais	Jeceaba	313540	3135407	5238.8	1017.8	719.2	733.6	520.5	2394

Minas

2637MG	31Gerais	Jenipapo de Minas	313545	3135456	7471.7	1451.7	1025.7	1046.2	742.4	1945

Minas

2638MG	31Gerais	Jequeri	313550	3135506	12794.6	2485.9	1756.4	1791.6	1271.3	3960

Minas

2639MG	31Gerais	Jequitaí	313560	3135605	7886.1	1532.2	1082.6	1104.2	783.6	2134

Minas

2640MG	31Gerais	Jequitibá	313570	3135704	5245.6	1019.2	720.1	734.5	521.2	1604

Minas

2641MG	31Gerais	Jequitinhonha	313580	3135803	25016.9	4860.5	3434.3	3503	2485.8	6901

Minas

2642MG	31Gerais	Jesuânia	313590	3135902	4836.8	939.7	664	677.3	480.6	1200

Minas

1

1

3

3

2

1

2

1

1

2

5

2

1

3

1

1

1

1

1

5

2643MG	31Gerais	Joaíma	313600	3136009	15355.5	2983.4	2108	2150.2	1525.8	2584

Minas

2644MG	31Gerais	Joanésia	313610	3136108	5098.5	990.6	699.9	713.9	506.6	2093

Minas

2645MG	31Gerais	João Monlevade	313620	3136207	77449	15047.6	10632.2	10844.8	7695.6	18294

Minas

2646MG	31Gerais	João Pinheiro	313630	3136306	47350.1	9199.7	6500.2	6630.2	4704.9	7740

Minas

2647MG	31Gerais	Joaquim Felício	313640	3136405	4539.7	882	623.2	635.7	451.1	1333

Minas

2648MG	31Gerais	Jordânia	313650	3136504	10679.1	2074.8	1466	1495.3	1061.1	2387

Minas	José Gonçalves

2649MG	31Gerais	de Minas	313652	3136520	4586.1	891	629.6	642.2	455.7	1199

Minas

2650MG	31Gerais	José Raydan	313655	3136553	4717.9	916.6	647.7	660.6	468.8	1175

Minas

2651MG	31Gerais	Josenópolis	313657	3136579	4762.4	925.3	653.8	666.9	473.2	1180

Minas

2652MG	31Gerais	Nova União	313660	3136603	5703.1	1108.1	782.9	798.6	566.7	1373

Minas

2653MG	31Gerais	Juatuba	313665	3136652	24672.8	4793.7	3387.1	3454.8	2451.6	5155

Minas

2654MG	31Gerais	Juiz de Fora	313670	3136702	547080.6	106292.3	75103.2	76604.9	54359.7	135295	33

Minas

2655MG	31Gerais	Juramento	313680	3136801	4265.5	828.7	585.6	597.3	423.8	1228

Minas

2656MG	31Gerais	Juruaia	313690	3136900	9967	1936.5	1368.3	1395.6	990.4	2204

Minas

2657MG	31Gerais	Juvenília	313695	3136959	5787.1	1124.4	794.5	810.3	575	1313

Minas

2658MG	31Gerais	Ladainha	313700	3137007	17724.1	3443.6	2433.2	2481.8	1761.1	3027

Minas

2659MG	31Gerais	Lagamar	313710	3137106	7700.9	1496.2	1057.2	1078.3	765.2	2301

Minas

2660MG	31Gerais	Lagoa da Prata	313720	3137205	49422.3	9602.3	6784.7	6920.3	4910.8	12359

Minas

2661MG	31Gerais	Lagoa dos Patos	313730	3137304	4221.2	820.1	579.5	591.1	419.4	1049

Minas

2662MG	31Gerais	Lagoa Dourada	313740	3137403	12758.1	2478.8	1751.4	1786.5	1267.7	4041

Minas

2663MG	31Gerais	Lagoa Formosa	313750	3137502	17789.1	3456.2	2442.1	2490.9	1767.6	6801

2664MG	Lagoa Grande	313753	3137536	9156.5	1779	1257	1282.1	909.8	2000

2665MG	Lagoa Santa	313760	3137601	58738.2	11412.2	8063.6	8224.8	5836.4	4032

2666MG	Lajinha	313770	3137700	20000.2	3885.8	2745.6	2800.5	1987.3	5610

2667MG	Lambari	313780	3137809	20382.4	3960.1	2798.1	2854	2025.3	6485

2668MG	Lamim	313790	3137908	3467.5	673.7	476	485.5	344.5	2068

2669MG	Laranjal	313800	3138005	6705.6	1302.8	920.5	939	666.3	2379

2670MG	Lassance	313810	3138104	6578.7	1278.2	903.1	921.2	653.7	1788

2671MG	Lavras	313820	3138203	98713.6	19179.1	13551.4	13822.4	9808.5	19682

2672MG	Leandro Ferreira	313830	3138302	3256.2	632.6	447	455.9	323.5	683

2673MG	Leme do Prado	313835	3138351	4917.2	955.4	675	688.5	488.6	1500

2674MG	Leopoldina	313840	3138401	52444.2	10189.4	7199.5	7343.5	5211	9624

2675MG	Liberdade	313850	3138500	5285.1	1026.8	725.5	740	525.1	955

2676MG	Lima Duarte	313860	3138609	16605.3	3226.2	2279.6	2325.2	1650	4419

2677MG	Limeira do Oeste	313862	3138625	7275.3	1413.5	998.8	1018.7	722.9	837

2678MG	Lontra	313865	3138658	8853.5	1720.1	1215.4	1239.7	879.7	1906

2679MG	Luisburgo	313867	3138674	6326.8	1229.2	868.5	885.9	628.7	1813

2680MG	Luislândia	313868	3138682	6618.7	1285.9	908.6	926.8	657.7	877

2681MG	Luminárias	313870	3138708	5500.8	1068.8	755.1	770.2	546.6	1879

2682MG	Luz	313880	3138807	18042.9	3505.6	2476.9	2526.5	1792.8	5684

2683MG	Machacalis	313890	3138906	7125.7	1384.5	978.2	997.8	708	2067

2684MG	31Gerais	Machado	313900	3139003	40767.9	7920.8	5596.6	5708.5	4050.8	8275

Minas	Madre de Deus de

2685MG	31Gerais	Minas	313910	3139102	5055.1	982.2	694	707.8	502.3	1247

Minas

2686MG	31Gerais	Malacacheta	313920	3139201	18952.9	3682.4	2601.9	2653.9	1883.2	4419

Minas

2687MG	31Gerais	Mamonas	313925	3139250	6503	1263.5	892.7	910.6	646.2	2658

Minas

2688MG	31Gerais	Manga	313930	3139300	19409.4	3771.1	2664.5	2717.8	1928.6	2688

Minas

2689MG	31Gerais	Manhuaçu	313940	3139409	85504.6	16612.7	11738.1	11972.8	8496	23701

Minas

2690MG	31Gerais	Manhumirim	313950	3139508	22262.7	4325.4	3056.2	3117.3	2212.1	4799

Minas

2691MG	31Gerais	Mantena	313960	3139607	27696.8	5381.2	3802.2	3878.2	2752	7073

Minas

2692MG	31Gerais	Maravilhas	313970	3139706	7628.7	1482.2	1047.3	1068.2	758	2592

Minas

2693MG	31Gerais	Mar de Espanha	313980	3139805	12389.5	2407.2	1700.8	1734.8	1231.1	2888

Minas

2694MG	31Gerais	Maria da Fé	313990	3139904	14338.3	2785.8	1968.4	2007.7	1424.7	3322

Minas

2695MG	31Gerais	Mariana	314000	3140001	57910.7	11251.5	7950	8108.9	5754.2	8105

Minas

2696MG	31Gerais	Marilac	314010	3140100	4223.6	820.6	579.8	591.4	419.7	907

Minas

2697MG	31Gerais	Mário Campos	314015	3140159	14387.7	2795.4	1975.1	2014.6	1429.6	3063

Minas

2698MG	31Gerais	Maripá de Minas	314020	3140209	2908.6	565.1	399.3	407.3	289	1323

Minas

2699MG	31Gerais	Marliéria	314030	3140308	4074.8	791.7	559.4	570.6	404.9	1338

Minas

2700MG	31Gerais	Marmelópolis	314040	3140407	2906.2	564.6	399	406.9	288.8	698

Minas

2701MG	31Gerais	Martinho Campos	314050	3140506	13128.6	2550.8	1802.3	1838.3	1304.5	4518

Minas

2702MG	31Gerais	Martins Soares	314053	3140530	7838.9	1523	1076.1	1097.6	778.9	2124

Minas

2703MG	31Gerais	Mata Verde	314055	3140555	8302.4	1613.1	1139.8	1162.5	825	1680

Minas

2704MG	31Gerais	Materlândia	314060	3140605	4589.7	891.7	630.1	642.7	456	1949

Minas

2

1

1

2

1

7

1

1

3

1

1

2

1

1

1

1

1

1

1

1

1

1

3

2705MG	31Gerais	Mateus Leme	314070	3140704	29700.5	5770.5	4077.3	4158.8	2951.1	7758

Minas

2706MG	31Gerais	Matias Barbosa	314080	3140803	14081.4	2735.9	1933.1	1971.7	1399.2	5900

Minas

2707MG	31Gerais	Matias Cardoso	314085	3140852	10651	2069.4	1462.2	1491.4	1058.3	1767

Minas

2708MG	31Gerais	Matipó	314090	3140902	18447.8	3584.2	2532.5	2583.1	1833	3173

Minas

2709MG	31Gerais	Mato Verde	314100	3141009	12738.9	2475	1748.8	1783.8	1265.8	3114

Minas

2710MG	31Gerais	Matozinhos	314110	3141108	36168.3	7027.1	4965.2	5064.5	3593.8	3803

Minas

2711MG	31Gerais	Matutina	314120	3141207	3802.8	738.8	522	532.5	377.9	1405

Minas

2712MG	31Gerais	Medeiros	314130	3141306	3652.7	709.7	501.4	511.5	362.9	1142

Minas

2713MG	31Gerais	Medina	314140	3141405	21194	4117.8	2909.5	2967.7	2105.9	3861

Minas

2714MG	31Gerais	Mendes Pimentel	314150	3141504	6463.9	1255.9	887.4	905.1	642.3	1865

Minas

2715MG	31Gerais	Mercês	314160	3141603	10669.4	2073	1464.7	1494	1060.1	4180

Minas

2716MG	31Gerais	Mesquita	314170	3141702	5929.5	1152	814	830.3	589.2	2402

Minas

2717MG	31Gerais	Minas Novas	314180	3141801	31498.6	6119.9	4324.1	4410.6	3129.8	6339

Minas

2718MG	31Gerais	Minduri	314190	3141900	3912.9	760.2	537.2	547.9	388.8	1113

Minas

2719MG	31Gerais	Mirabela	314200	3142007	13458.8	2614.9	1847.6	1884.6	1337.3	4031

Minas

2720MG	31Gerais	Miradouro	314210	3142106	10612.5	2061.9	1456.9	1486	1054.5	2724

Minas

2721MG	31Gerais	Miraí	314220	3142205	14539.4	2824.9	1996	2035.9	1444.7	5416

Minas

2722MG	31Gerais	Miravânia	314225	3142254	4763	925.4	653.9	666.9	473.3	1082

Minas

2723MG	31Gerais	Moeda	314230	3142304	4854.6	943.2	666.4	679.8	482.4	1205

Minas

2724MG	31Gerais	Moema	314240	3142403	7342.7	1426.6	1008	1028.2	729.6	2312

Minas

2725MG	31Gerais	Monjolos	314250	3142502	2325.2	451.8	319.2	325.6	231	1673

2726MG	Monsenhor Paulo	314260	3142601	8507.4	1652.9	1167.9	1191.2	845.3	1598

2727MG	Montalvânia	314270	3142700	15604.6	3031.8	2142.2	2185	1550.5	3147

Monte Alegre de

2728MG	Minas	314280	3142809	20559.6	3994.5	2822.4	2878.9	2042.9	3860

2729MG	Monte Azul	314290	3142908	21741.2	4224.1	2984.6	3044.3	2160.3	5382

2730MG	Monte Belo	314300	3143005	13273.2	2578.9	1822.1	1858.6	1318.9	2605

2731MG	Monte Carmelo	314310	3143104	47288	9187.6	6491.7	6621.5	4698.7	7085

2732MG	Monte Formoso	314315	3143153	4830.1	938.4	663.1	676.3	479.9	982

Monte Santo de

2733MG	Minas	314320	3143203	21633.7	4203.2	2969.9	3029.3	2149.6	13241

2734MG	Montes Claros	314330	3143302	388318.5	75446.4	53308.4	54374.2	38584.6	92684	25

2735MG	Monte Sião	314340	3143401	22672.2	4405	3112.4	3174.7	2252.8	3134

2736MG	Montezuma	314345	3143450	7921.3	1539	1087.4	1109.2	787.1	1973

Morada Nova de

2737MG	Minas	314350	3143500	8639.7	1678.6	1186.1	1209.8	858.5	1460

2738MG	Morro da Garça	314360	3143609	2601.7	505.5	357.2	364.3	258.5	1636

2739MG	Morro do Pilar	314370	3143708	3340.9	649.1	458.6	467.8	332	768

2740MG	Munhoz	314380	3143807	6229.9	1210.4	855.2	872.3	619	1206

2741MG	Muriaé	314390	3143906	105729.8	20542.2	14514.6	14804.8	10505.7	39944

2742MG	Mutum	314400	3144003	27141.8	5273.4	3726	3800.5	2696.9	9341

2743MG	Muzambinho	314410	3144102	20750	4031.5	2848.6	2905.5	2061.8	3786

2744MG	Nacip Raydan	314420	3144201	3223.5	626.3	442.5	451.4	320.3	468

2745MG	Nanuque	314430	3144300	41306.5	8025.4	5670.6	5783.9	4104.3	7112

2746MG	31Gerais	Naque	314435	3144359	6723.4	1306.3	923	941.4	668.1	1688

Minas

2747MG	31Gerais	Natalândia	314437	3144375	3335.8	648.1	457.9	467.1	331.5	296

Minas

2748MG	31Gerais	Natércia	314440	3144409	4749.6	922.8	652	665.1	471.9	1577

Minas

2749MG	31Gerais	Nazareno	314450	3144508	8356.6	1623.6	1147.2	1170.1	830.3	1474

Minas

2750MG	31Gerais	Nepomuceno	314460	3144607	26534.9	5155.5	3642.7	3715.5	2636.6	6940

Minas

2751MG	31Gerais	Ninheira	314465	3144656	10160.3	1974	1394.8	1422.7	1009.6	2629

Minas

2752MG	31Gerais	Nova Belém	314467	3144672	3527.3	685.3	484.2	493.9	350.5	687

Minas

2753MG	31Gerais	Nova Era	314470	3144706	17770.8	3452.7	2439.6	2488.4	1765.8	3801

Minas

2754MG	31Gerais	Nova Lima	314480	3144805	88445.4	17184.1	12141.8	12384.5	8788.2	20885

Minas

2755MG	31Gerais	Nova Módica	314490	3144904	3749.1	728.4	514.7	525	372.5	1174

Minas

2756MG	31Gerais	Nova Ponte	314500	3145000	14238	2766.3	1954.6	1993.7	1414.7	2061

Minas

2757MG	31Gerais	Nova Porteirinha	314505	3145059	7538	1464.6	1034.8	1055.5	749	1271

Minas

2758MG	31Gerais	Nova Resende	314510	3145109	16191.2	3145.8	2222.7	2267.2	1608.8	7238

Minas

2759MG	31Gerais	Nova Serrana	314520	3145208	88050	17107.2	12087.5	12329.2	8748.9	12842

Minas

2760MG	31Gerais	Novo Cruzeiro	314530	3145307	31390.1	6098.8	4309.2	4395.4	3119	6469

Minas	Novo Oriente de

2761MG	31Gerais	Minas	314535	3145356	10661.3	2071.4	1463.6	1492.8	1059.3	2879

Minas

2762MG	31Gerais	Novorizonte	314537	3145372	5180.9	1006.6	711.2	725.5	514.8	1448

Minas

2763MG	31Gerais	Olaria	314540	3145406	1894.7	368.1	260.1	265.3	188.3	778

Minas

2764MG	31Gerais	Olhos-d'Água	314545	3145455	5716.2	1110.6	784.7	800.4	568	1411

Minas

2765MG	31Gerais	Olímpio Noronha	314550	3145505	2682.2	521.1	368.2	375.6	266.5	1466

Minas

2766MG	31Gerais	Oliveira	314560	3145604	40988.3	7963.6	5626.9	5739.4	4072.7	9835

Minas

9

2

1

1

5

2

4

1

3

2

5

1

3

7

1

1

2

6

2767MG	31Gerais	Oliveira Fortes	314570	3145703	2154.2	418.5	295.7	301.6	214	1454

Minas

2768MG	31Gerais	Onça de Pitangui	314580	3145802	3136.5	609.4	430.6	439.2	311.7	848

Minas

2769MG	31Gerais	Oratórios	314585	3145851	4623.9	898.4	634.8	647.5	459.4	1691

Minas

2770MG	31Gerais	Orizânia	314587	3145877	7715.1	1499	1059.1	1080.3	766.6	2409

Minas

2771MG	31Gerais	Ouro Branco	314590	3145901	37669.3	7318.8	5171.2	5274.6	3742.9	8724

Minas

2772MG	31Gerais	Ouro Fino	314600	3146008	32922.2	6396.5	4519.6	4609.9	3271.3	2317

Minas

2773MG	31Gerais	Ouro Preto	314610	3146107	73013	14185.7	10023.2	10223.6	7254.8	17533

Minas	Ouro Verde de

2774MG	31Gerais	Minas	314620	3146206	6053.1	1176.1	831	847.6	601.5	1422

Minas

2775MG	31Gerais	Padre Carvalho	314625	3146255	6159.1	1196.7	845.5	862.4	612	1049

Minas

2776MG	31Gerais	Padre Paraíso	314630	3146305	19689.3	3825.4	2702.9	2757	1956.4	3998

Minas

2777MG	31Gerais	Paineiras	314640	3146404	4622	898	634.5	647.2	459.3	1621

Minas

2778MG	31Gerais	Pains	314650	3146503	8239.4	1600.8	1131.1	1153.7	818.7	3352

Minas

2779MG	31Gerais	Pai Pedro	314655	3146552	6080.5	1181.4	834.7	851.4	604.2	1582

Minas

2780MG	31Gerais	Paiva	314660	3146602	1564.1	303.9	214.7	219	155.4	751

Minas

2781MG	31Gerais	Palma	314670	3146701	6659.8	1293.9	914.3	932.5	661.7	2638

Minas

2782MG	31Gerais	Palmópolis	314675	3146750	6358.3	1235.4	872.9	890.3	631.8	1889

Minas

2783MG	31Gerais	Papagaios	314690	3146909	15047	2923.5	2065.7	2107	1495.1	3801

Minas

2784MG	31Gerais	Paracatu	314700	3147006	89686.7	17425.2	12312.2	12558.4	8911.6	11722

Minas

2785MG	31Gerais	Pará de Minas	314710	3147105	89785.8	17444.5	12325.8	12572.2	8921.4	22984

Minas

2786MG	31Gerais	Paraguaçu	314720	3147204	21085.6	4096.7	2894.6	2952.5	2095.1	4990

Minas

2787MG	31Gerais	Paraisópolis	314730	3147303	20410.5	3965.6	2802	2858	2028.1	3794

2788MG	Paraopeba	314740	3147402	23761.3	4616.6	3262	3327.2	2361	4364

2789MG	Passabém	314750	3147501	1733.7	336.8	238	242.8	172.3	506

2790MG	Passa Quatro	314760	3147600	16130	3133.9	2214.3	2258.6	1602.7	6073

2791MG	Passa Tempo	314770	3147709	8247.2	1602.3	1132.2	1154.8	819.5	2746

2792MG	Passa-Vinte	314780	3147808	2086.7	405.4	286.5	292.2	207.3	512

2793MG	Passos	314790	3147907	111505.5	21664.4	15307.5	15613.5	11079.6	49161	15

2794MG	Patis	314795	3147956	5830.9	1132.9	800.5	816.5	579.4	1388

2795MG	Patos de Minas	314800	3148004	146637	28490.1	20130.3	20532.8	14570.3	46232	14

2796MG	Patrocínio	314810	3148103	87340.9	16969.5	11990.2	12229.9	8678.5	23142

Patrocínio do

2797MG	Muriaé	314820	3148202	5537.2	1075.8	760.1	775.3	550.2	1735

2798MG	Paula Cândido	314830	3148301	9525.8	1850.8	1307.7	1333.8	946.5	5048

2799MG	Paulistas	314840	3148400	4939.1	959.6	678	691.6	490.8	1062

2800MG	Pavão	314850	3148509	8632.3	1677.2	1185	1208.7	857.7	1812

2801MG	Peçanha	314860	3148608	17605.9	3420.7	2416.9	2465.3	1749.4	2534

2802MG	Pedra Azul	314870	3148707	24362.1	4733.3	3344.4	3411.3	2420.7	5209

2803MG	Pedra Bonita	314875	3148756	6952.6	1350.8	954.5	973.5	690.8	1771

2804MG	Pedra do Anta	314880	3148806	3290.2	639.3	451.7	460.7	326.9	2224

2805MG	Pedra do Indaiá	314890	3148905	3968.3	771	544.8	555.7	394.3	1303

2806MG	Pedra Dourada	314900	3149002	2363.5	459.2	324.5	330.9	234.8	1077

2807MG	Pedralva	314910	3149101	11484.3	2231.3	1576.6	1608.1	1141.1	2297

Pedras de Maria

2808MG	31Gerais	da Cruz	314915	3149150	11077.5	2152.2	1520.7	1551.1	1100.7	1676

Minas

2809MG	31Gerais	Pedrinópolis	314920	3149200	3600.8	699.6	494.3	504.2	357.8	1519

Minas

2810MG	31Gerais	Pedro Leopoldo	314930	3149309	62031.2	12052	8515.6	8685.9	6163.6	13852

Minas

2811MG	31Gerais	Pedro Teixeira	314940	3149408	1817.4	353.1	249.5	254.5	180.6	1094

Minas

2812MG	31Gerais	Pequeri	314950	3149507	3277	636.7	449.9	458.9	325.6	1294

Minas

2813MG	31Gerais	Pequi	314960	3149606	4280	831.6	587.6	599.3	425.3	1808

Minas

2814MG	31Gerais	Perdigão	314970	3149705	10224.8	1986.6	1403.7	1431.7	1016	1903

Minas

2815MG	31Gerais	Perdizes	314980	3149804	15401.1	2992.3	2114.3	2156.5	1530.3	2996

Minas

2816MG	31Gerais	Perdões	314990	3149903	20942	4068.8	2874.9	2932.4	2080.9	6817

Minas

2817MG	31Gerais	Periquito	314995	3149952	7019.3	1363.8	963.6	982.9	697.5	1239

Minas

2818MG	31Gerais	Pescador	315000	3150000	4236.7	823.1	581.6	593.2	421	1398

Minas

2819MG	31Gerais	Piau	315010	3150109	2833.7	550.6	389	396.8	281.6	1574

Minas	Piedade de

2820MG	31Gerais	Caratinga	315015	3150158	7873.1	1529.7	1080.8	1102.4	782.3	1889

Minas	Piedade de Ponte

2821MG	31Gerais	Nova	315020	3150208	4148.7	806.1	569.5	580.9	412.2	1232

Minas	Piedade do Rio

2822MG	31Gerais	Grande	315030	3150307	4669.2	907.2	641	653.8	463.9	1288

Minas	Piedade dos

2823MG	31Gerais	Gerais	315040	3150406	4856.7	943.6	666.7	680.1	482.6	1429

Minas

2824MG	31Gerais	Pimenta	315050	3150505	8535.8	1658.4	1171.8	1195.2	848.1	3567

Minas

2825MG	31Gerais	Pingo-d'Água	315053	3150539	4716.5	916.4	647.5	660.4	468.6	1910

Minas

2826MG	31Gerais	Pintópolis	315057	3150570	7438.5	1445.2	1021.2	1041.6	739.1	1574

Minas

2827MG	31Gerais	Piracema	315060	3150604	6487.7	1260.5	890.6	908.4	644.6	3202

Minas

2828MG	31Gerais	Pirajuba	315070	3150703	5428.6	1054.7	745.2	760.1	539.4	873

Minas

1

1

4

1

1

1

1

2

1

5

1

3

4

1

1

1

6

1

1

1

1

2829MG	31Gerais	Piranga	315080	3150802	17630.4	3425.4	2420.3	2468.7	1751.8	6238

Minas

2830MG	31Gerais	Piranguçu	315090	3150901	5400.4	1049.2	741.4	756.2	536.6	990

Minas

2831MG	31Gerais	Piranguinho	315100	3151008	8385	1629.1	1151.1	1174.1	833.2	2032

Minas

2832MG	31Gerais	Pirapetinga	315110	3151107	10673.3	2073.7	1465.2	1494.5	1060.5	3274

Minas

2833MG	31Gerais	Pirapora	315120	3151206	55452.4	10773.8	7612.5	7764.7	5509.9	9580

Minas

2834MG	31Gerais	Piraúba	315130	3151305	10963.3	2130.1	1505	1535.1	1089.3	2969

Minas

2835MG	31Gerais	Pitangui	315140	3151404	26868.1	5220.2	3688.5	3762.2	2669.7	5671

Minas

2836MG	31Gerais	Piumhi	315150	3151503	33582.2	6524.7	4610.2	4702.3	3336.8	15702

Minas

2837MG	31Gerais	Planura	315160	3151602	11323.9	2200.1	1554.5	1585.6	1125.2	1724

Minas

2838MG	31Gerais	Poço Fundo	315170	3151701	16544.4	3214.4	2271.2	2316.6	1643.9	4358

Minas

2839MG	31Gerais	Poços de Caldas	315180	3151800	161272	31333.5	22139.4	22582.1	16024.5	44084	10

Minas

2840MG	31Gerais	Pocrane	315190	3151909	8841.6	1717.8	1213.8	1238	878.5	2002

Minas

2841MG	31Gerais	Pompéu	315200	3152006	30723	5969.2	4217.7	4302	3052.7	9287

Minas

2842MG	31Gerais	Ponte Nova	315210	3152105	59197.5	11501.5	8126.6	8289.1	5882.1	17875

Minas

2843MG	31Gerais	Ponto Chique	315213	3152131	4152.3	806.8	570	581.4	412.6	987

Minas	Ponto dos

2844MG	31Gerais	Volantes	315217	3152170	11846.6	2301.7	1626.3	1658.8	1177.1	2172

Minas

2845MG	31Gerais	Porteirinha	315220	3152204	38227.5	7427.2	5247.9	5352.8	3798.4	8696

Minas

2846MG	31Gerais	Porto Firme	315230	3152303	10947.1	2126.9	1502.8	1532.9	1087.7	6084

Minas

2847MG	31Gerais	Poté	315240	3152402	16274.3	3161.9	2234.1	2278.8	1617.1	3779

Minas

2848MG	31Gerais	Pouso Alegre	315250	3152501	141568.8	27505.4	19434.6	19823.1	14066.7	32903

Minas

2849MG	Pouso Alto	315260	3152600	6164.2	1197.6	846.2	863.1	612.5	3656

2850MG	Prados	315270	3152709	8792.4	1708.3	1207	1231.2	873.6	2384

2851MG	Prata	315280	3152808	27076.2	5260.6	3717	3791.3	2690.4	3761

2852MG	Pratápolis	315290	3152907	8823.1	1714.2	1211.2	1235.5	876.7	5787

2853MG	Pratinha	315300	3153004	3462.5	672.7	475.3	484.8	344	818

Presidente

2854MG	Bernardes	315310	3153103	5528.4	1074.1	758.9	774.1	549.3	2557

Presidente

2855MG	Juscelino	315320	3153202	3832.9	744.7	526.2	536.7	380.8	2475

Presidente

2856MG	Kubitschek	315330	3153301	3016.5	586.1	414.1	422.4	299.7	1323

Presidente

2857MG	Olegário	315340	3153400	19219	3734.1	2638.4	2691.1	1909.7	4322

2858MG	Alto Jequitibá	315350	3153509	8421.4	1636.2	1156.1	1179.2	836.8	2831

Prudente de

2859MG	Morais	315360	3153608	10230.5	1987.7	1404.4	1432.5	1016.5	2542

2860MG	Quartel Geral	315370	3153707	3465.2	673.3	475.7	485.2	344.3	1300

2861MG	Queluzito	315380	3153806	1920.4	373.1	263.6	268.9	190.8	1062

2862MG	Raposos	315390	3153905	16002.9	3109.2	2196.9	2240.8	1590.1	3815

2863MG	Raul Soares	315400	3154002	24089.8	4680.4	3307	3373.2	2393.6	9064

2864MG	Recreio	315410	3154101	10528.6	2045.6	1445.4	1474.3	1046.2	3485

2865MG	Reduto	315415	3154150	6921.8	1344.8	950.2	969.2	687.8	1482

2866MG	Resende Costa	315420	3154200	11320.9	2199.5	1554.1	1585.2	1124.9	5521

2867MG	Resplendor	315430	3154309	17445.8	3389.5	2395	2442.8	1733.5	6423

2868MG	Ressaquinha	315440	3154408	4802.7	933.1	659.3	672.5	477.2	1785

2869MG	Riachinho	315445	3154457	8167.4	1586.8	1121.2	1143.6	811.5	1300

Riacho dos

2870MG	31Gerais	Machados	315450	3154507	9533.7	1852.3	1308.8	1335	947.3	1789

Minas	Ribeirão das

2871MG	31Gerais	Neves	315460	3154606	317748.2	61735.3	43620.5	44492.6	31572.5	62731	23

Minas

2872MG	31Gerais	Ribeirão Vermelho	315470	3154705	3970.1	771.4	545	555.9	394.5	1467

Minas

2873MG	31Gerais	Rio Acima	315480	3154804	9770.5	1898.3	1341.3	1368.1	970.8	3111

Minas

2874MG	31Gerais	Rio Casca	315490	3154903	14083.6	2736.3	1933.4	1972.1	1399.4	4245

Minas

2875MG	31Gerais	Rio Doce	315500	3155009	2563.5	498.1	351.9	359	254.7	811

Minas

2876MG	31Gerais	Rio do Prado	315510	3155108	5251.3	1020.3	720.9	735.3	521.8	1532

Minas

2877MG	31Gerais	Rio Espera	315520	3155207	5869.6	1140.4	805.8	821.9	583.2	1304

Minas

2878MG	31Gerais	Rio Manso	315530	3155306	5599.6	1087.9	768.7	784.1	556.4	2015

Minas

2879MG	31Gerais	Rio Novo	315540	3155405	8930.9	1735.2	1226	1250.5	887.4	3211

Minas

2880MG	31Gerais	Rio Paranaíba	315550	3155504	12232.3	2376.6	1679.3	1712.8	1215.4	2428

Minas	Rio Pardo de

2881MG	31Gerais	Minas	315560	3155603	30303.7	5887.7	4160.1	4243.3	3011.1	5612

Minas

2882MG	31Gerais	Rio Piracicaba	315570	3155702	14414.2	2800.5	1978.8	2018.3	1432.2	3478

Minas

2883MG	31Gerais	Rio Pomba	315580	3155801	17694.7	3437.9	2429.1	2477.7	1758.2	5770

Minas

2884MG	31Gerais	Rio Preto	315590	3155900	5443.5	1057.6	747.3	762.2	540.9	2388

Minas

2885MG	31Gerais	Rio Vermelho	315600	3156007	13445.3	2612.3	1845.8	1882.7	1336	3409

Minas

2886MG	31Gerais	Ritápolis	315610	3156106	4837.8	939.9	664.1	677.4	480.7	1720

Minas

2887MG	31Gerais	Rochedo de Minas	315620	3156205	2229.9	433.2	306.1	312.2	221.6	931

Minas

2888MG	31Gerais	Rodeiro	315630	3156304	7528.2	1462.7	1033.5	1054.1	748	2787

Minas

2889MG	31Gerais	Romaria	315640	3156403	3612.2	701.8	495.9	505.8	358.9	1080

Minas

2890MG	31Gerais	Rosário da Limeira	315645	3156452	4460.5	866.6	612.3	624.6	443.2	1765

Minas

2

1

1

1

1

3

1

1

2

1

4

1

1

2891MG	31Gerais	Rubelita	315650	3156502	7046.1	1369	967.3	986.6	700.1	2216

Minas

2892MG	31Gerais	Rubim	315660	3156601	10192.5	1980.3	1399.2	1427.2	1012.8	2189

Minas

2893MG	31Gerais	Sabará	315670	3156700	132473.7	25738.3	18186	18549.6	13163	17017

Minas

2894MG	31Gerais	Sabinópolis	315680	3156809	15791.9	3068.2	2167.9	2211.3	1569.1	6628

Minas

2895MG	31Gerais	Sacramento	315690	3156908	25255	4906.8	3467	3536.3	2509.4	6471

Minas

2896MG	31Gerais	Salinas	315700	3157005	40748.4	7917	5593.9	5705.8	4048.9	7919

Minas

2897MG	31Gerais	Salto da Divisa	315710	3157104	7014.3	1362.8	962.9	982.2	697	2056

Minas

2898MG	31Gerais	Santa Bárbara	315720	3157203	29738	5777.8	4082.4	4164.1	2954.9	6487

Minas	Santa Bárbara do

2899MG	31Gerais	Leste	315725	3157252	7994	1553.2	1097.4	1119.4	794.3	1819

Minas	Santa Bárbara do

2900MG	31Gerais	Monte Verde	315727	3157278	2990.4	581	410.5	418.7	297.1	1072

Minas	Santa Bárbara do

2901MG	31Gerais	Tugúrio	315730	3157302	4562.3	886.4	626.3	638.8	453.3	954

Minas	Santa Cruz de

2902MG	31Gerais	Minas	315733	3157336	8305.6	1613.7	1140.2	1163	825.3	2380

Minas	Santa Cruz de

2903MG	31Gerais	Salinas	315737	3157377	4333.9	842	595	606.9	430.6	1577

Minas	Santa Cruz do

2904MG	31Gerais	Escalvado	315740	3157401	4945.9	960.9	679	692.5	491.4	1905

Minas	Santa Efigênia de

2905MG	31Gerais	Minas	315750	3157500	4568.9	887.7	627.2	639.8	454	1087

Minas

2906MG	31Gerais	Santa Fé de Minas	315760	3157609	3961.2	769.6	543.8	554.7	393.6	1069

Minas	Santa Helena de

2907MG	31Gerais	Minas	315765	3157658	6274.9	1219.2	861.4	878.6	623.5	2189

Minas

2908MG	31Gerais	Santa Juliana	315770	3157708	12714.1	2470.2	1745.4	1780.3	1263.3	1828

Minas

2909MG	31Gerais	Santa Luzia	315780	3157807	213115.2	41406.2	29256.5	29841.4	21175.8	13710

Minas

2910MG	31Gerais	Santa Margarida	315790	3157906	15753.4	3060.7	2162.6	2205.9	1565.3	3536

Minas	Santa Maria de

2911MG	Itabira	315800	3158003	10820.4	2102.3	1485.4	1515.1	1075.2	2280

Santa Maria do

2912MG	Salto	315810	3158102	5326.4	1034.9	731.2	745.8	529.2	1127

Santa Maria do

2913MG	Suaçuí	315820	3158201	14677.1	2851.6	2014.9	2055.2	1458.4	2722

Santana da

2914MG	Vargem	315830	3158300	7260.6	1410.7	996.7	1016.7	721.4	2918

Santana de

2915MG	Cataguases	315840	3158409	3782.7	734.9	519.3	529.7	375.9	1518

Santana de

2916MG	Pirapama	315850	3158508	7939.5	1542.6	1089.9	1111.7	788.9	2451

Santana do

2917MG	Deserto	315860	3158607	3962.2	769.8	543.9	554.8	393.7	1605

Santana do

2918MG	Garambéu	315870	3158706	2365.3	459.6	324.7	331.2	235	752

2919MG	Santana do Jacaré	315880	3158805	4763.3	925.5	653.9	667	473.3	1254

Santana do

2920MG	Manhuaçu	315890	3158904	8731.5	1696.4	1198.7	1222.6	867.6	1663

Santana do

2921MG	Paraíso	315895	3158953	31032.9	6029.4	4260.2	4345.4	3083.5	5366

Santana do

2922MG	Riacho	315900	3159001	4198.8	815.8	576.4	587.9	417.2	911

Santana dos

2923MG	Montes	315910	3159100	3849.1	747.8	528.4	539	382.5	1164

Santa Rita de

2924MG	Caldas	315920	3159209	9103.9	1768.8	1249.8	1274.8	904.6	2967

Santa Rita de

2925MG	Jacutinga	315930	3159308	5004.7	972.4	687	700.8	497.3	1560

Santa Rita de

2926MG	Minas	315935	3159357	6937.6	1347.9	952.4	971.4	689.3	1798

Santa Rita de

2927MG	Ibitipoca	315940	3159407	3554.4	690.6	487.9	497.7	353.2	757

Santa Rita do

2928MG	Itueto	315950	3159506	5671.9	1102	778.6	794.2	563.6	1829

Santa Rita do

2929MG	Sapucaí	315960	3159605	40776.2	7922.4	5597.8	5709.7	4051.7	5082

Santa Rosa da

2930MG	Serra	315970	3159704	3322.5	645.5	456.1	465.2	330.1	1024

2931MG	Santa Vitória	315980	3159803	19107.9	3712.5	2623.1	2675.6	1898.6	5782

Santo Antônio do

2932MG	31Gerais	Amparo	315990	3159902	18108.6	3518.3	2485.9	2535.7	1799.3	6281

Minas	Santo Antônio do

2933MG	31Gerais	Aventureiro	316000	3160009	3612	701.8	495.9	505.8	358.9	2051

Minas	Santo Antônio do

2934MG	31Gerais	Grama	316010	3160108	4055.6	788	556.8	567.9	403	1967

Minas	Santo Antônio do

2935MG	31Gerais	Itambé	316020	3160207	4048.8	786.6	555.8	566.9	402.3	1450

Minas	Santo Antônio do

2936MG	31Gerais	Jacinto	316030	3160306	11860.2	2304.3	1628.2	1660.7	1178.5	3133

Minas	Santo Antônio do

2937MG	31Gerais	Monte	316040	3160405	27350.5	5313.9	3754.7	3829.7	2717.6	8467

Minas	Santo Antônio do

2938MG	31Gerais	Retiro	316045	3160454	7191.1	1397.2	987.2	1006.9	714.5	1774

Minas	Santo Antônio do

2939MG	31Gerais	Rio Abaixo	316050	3160504	1793.8	348.5	246.3	251.2	178.2	532

Minas

2940MG	31Gerais	Santo Hipólito	316060	3160603	3208.6	623.4	440.5	449.3	318.8	1461

Minas

2941MG	31Gerais	Santos Dumont	316070	3160702	46957.4	9123.4	6446.3	6575.2	4665.8	14451

Minas

2942MG	31Gerais	São Bento Abade	316080	3160801	4962.9	964.2	681.3	694.9	493.1	1553

Minas	São Brás do

2943MG	31Gerais	Suaçuí	316090	3160900	3660.6	711.2	502.5	512.6	363.7	1512

Minas	São Domingos

2944MG	31Gerais	das Dores	316095	3160959	5585	1085.1	766.7	782	554.9	1609

Minas	São Domingos do

2945MG	31Gerais	Prata	316100	3161007	17575.1	3414.7	2412.7	2460.9	1746.3	5322

Minas	São Félix de

2946MG	31Gerais	Minas	316105	3161056	3418.8	664.2	469.3	478.7	339.7	757

Minas

2947MG	31Gerais	São Francisco	316110	3161106	55656.8	10813.6	7640.6	7793.3	5530.2	8027

Minas	São Francisco de

2948MG	31Gerais	Paula	316120	3161205	6584.8	1279.4	904	922	654.3	2009

Minas	São Francisco de

2949MG	31Gerais	Sales	316130	3161304	6062.2	1177.8	832.2	848.9	602.4	1548

Minas	São Francisco do

2950MG	31Gerais	Glória	316140	3161403	5088.1	988.6	698.5	712.5	505.6	2333

Minas

2951MG	31Gerais	São Geraldo	316150	3161502	11364.9	2208.1	1560.2	1591.4	1129.3	4546

Minas	São Geraldo da

2952MG	31Gerais	Piedade	316160	3161601	4245.7	824.9	582.8	594.5	421.9	1110

1

2

1

2

3

1

1

1

1

1

1

2

7

1

2

1

2

1

6

Minas	São Geraldo do

2953MG	31Gerais	Baixio	316165	3161650	3773.5	733.2	518	528.4	374.9	776

Minas	São Gonçalo do

2954MG	31Gerais	Abaeté	316170	3161700	6818	1324.7	936	954.7	677.5	1612

Minas	São Gonçalo do

2955MG	31Gerais	Pará	316180	3161809	11460.2	2226.6	1573.3	1604.7	1138.7	2685

Minas	São Gonçalo do

2956MG	31Gerais	Rio Abaixo	316190	3161908	10428.5	2026.2	1431.6	1460.2	1036.2	3229

Minas	São Gonçalo do

2957MG	31Gerais	Sapucaí	316200	3162005	24920.4	4841.8	3421.1	3489.5	2476.2	2879

Minas

2958MG	31Gerais	São Gotardo	316210	3162104	33907.5	6587.9	4654.8	4747.9	3369.2	5856

Minas	São João Batista

2959MG	31Gerais	do Glória	316220	3162203	7235.4	1405.8	993.3	1013.1	718.9	6133

Minas	São João da

2960MG	31Gerais	Lagoa	316225	3162252	4834.2	939.2	663.6	676.9	480.3	1667

Minas

2961MG	31Gerais	São João da Mata	316230	3162302	2773.8	538.9	380.8	388.4	275.6	1167

Minas	São João da

2962MG	31Gerais	Ponte	316240	3162401	25585.8	4971.1	3512.4	3582.6	2542.3	4412

Minas	São João das

2963MG	31Gerais	Missões	316245	3162450	12463.1	2421.5	1710.9	1745.1	1238.4	1547

Minas

2964MG	31Gerais	São João del Rei	316250	3162500	88125.5	17121.9	12097.9	12339.8	8756.4	24614

Minas	São João do

2965MG	31Gerais	Manhuaçu	316255	3162559	10981.9	2133.7	1507.6	1537.7	1091.2	2439

Minas	São João do

2966MG	31Gerais	Manteninha	316257	3162575	5563.6	1081	763.8	779	552.8	1371

Minas	São João do

2967MG	31Gerais	Oriente	316260	3162609	7798.2	1515.1	1070.5	1091.9	774.9	2463

Minas	São João do

2968MG	31Gerais	Pacuí	316265	3162658	4276.6	830.9	587.1	598.8	424.9	830

Minas	São João do

2969MG	31Gerais	Paraíso	316270	3162708	23200.2	4507.6	3184.9	3248.6	2305.2	6488

Minas	São João

2970MG	31Gerais	Evangelista	316280	3162807	15850.2	3079.5	2175.9	2219.4	1574.9	3240

Minas	São João

2971MG	31Gerais	Nepomuceno	316290	3162906	25974.8	5046.6	3565.8	3637.1	2580.9	11688

Minas	São Joaquim de

2972MG	31Gerais	Bicas	316292	3162922	28654.5	5567.3	3933.7	4012.3	2847.2	5676

Minas

2973MG	São José da Barra	316294	3162948	7163.2	1391.7	983.4	1003	711.8	4975

2974MG	São José da Lapa	316295	3162955	21884.3	4251.9	3004.3	3064.3	2174.5	4204

São José da

2975MG	Safira	316300	3163003	4215.5	819	578.7	590.3	418.9	993

São José da

2976MG	Varginha	316310	3163102	4623.9	898.4	634.8	647.5	459.4	1258

São José do

2977MG	Alegre	316320	3163201	4139.3	804.2	568.2	579.6	411.3	915

São José do

2978MG	Divino	316330	3163300	3894.1	756.6	534.6	545.3	386.9	1449

São José do

2979MG	Goiabal	316340	3163409	5606.8	1089.3	769.7	785.1	557.1	2313

São José do

2980MG	Jacuri	316350	3163508	6589.3	1280.2	904.6	922.7	654.7	1773

São José do

2981MG	Mantimento	316360	3163607	2716.3	527.7	372.9	380.3	269.9	974

2982MG	São Lourenço	316370	3163706	44120.8	8572.2	6056.9	6178	4384	19750

São Miguel do

2983MG	Anta	316380	3163805	6927.1	1345.9	951	970	688.3	3107 São Pedro da

2984MG	União	316390	3163904	4926.1	957.1	676.3	689.8	489.5	3435

São Pedro dos

2985MG	Ferros	316400	3164001	8193.8	1592	1124.8	1147.3	814.2	2656

São Pedro do

2986MG	Suaçuí	316410	3164100	5489.9	1066.6	753.7	768.7	545.5	1191

2987MG	São Romão	316420	3164209	11359.3	2207	1559.4	1590.6	1128.7	1571

São Roque de

2988MG	Minas	316430	3164308	6938.7	1348.1	952.5	971.6	689.5	2996

São Sebastião da

2989MG	Bela Vista	316440	3164407	5267.8	1023.5	723.2	737.6	523.4	2046

São Sebastião da

2990MG	Vargem Alegre	316443	3164431	2930.3	569.3	402.3	410.3	291.2	1297

São Sebastião do

2991MG	Anta	316447	3164472	6188.5	1202.4	849.6	866.5	614.9	1360

São Sebastião do

2992MG	Maranhão	316450	3164506	10501	2040.2	1441.6	1470.4	1043.4	2780

São Sebastião do

2993MG	Oeste	316460	3164605	6326.8	1229.2	868.5	885.9	628.7	2480

São Sebastião do

2994MG	31Gerais	Paraíso	316470	3164704	68560.8	13320.7	9412	9600.2	6812.4	28443	10

Minas	São Sebastião do

2995MG	31Gerais	Rio Preto	316480	3164803	1583.4	307.6	217.4	221.7	157.3	516

Minas	São Sebastião do

2996MG	31Gerais	Rio Verde	316490	3164902	2197.2	426.9	301.6	307.7	218.3	1327

Minas

2997MG	31Gerais	São Tiago	316500	3165008	10869.5	2111.8	1492.2	1522	1080	2856

Minas	São Tomás de

2998MG	31Gerais	Aquino	316510	3165107	7149	1389	981.4	1001	710.3	3357

Minas	São Thomé das

2999MG	31Gerais	Letras	316520	3165206	6939.2	1348.2	952.6	971.7	689.5	1923

Minas	São Vicente de

3000MG	31Gerais	Minas	316530	3165305	7440.2	1445.6	1021.4	1041.8	739.3	2016

Minas

3001MG	31Gerais	Sapucaí-Mirim	316540	3165404	6638	1289.7	911.3	929.5	659.6	1244

Minas

3002MG	31Gerais	Sardoá	316550	3165503	5990.6	1163.9	822.4	838.8	595.2	1308

Minas

3003MG	31Gerais	Sarzedo	316553	3165537	29350.7	5702.5	4029.3	4109.8	2916.4	7037

Minas

3004MG	31Gerais	Setubinha	316555	3165552	11656.8	2264.8	1600.2	1632.2	1158.3	1700

Minas

3005MG	31Gerais	Sem-Peixe	316556	3165560	2783.6	540.8	382.1	389.8	276.6	1498

Minas

3006MG	31Gerais	Senador Amaral	316557	3165578	5348	1039.1	734.2	748.9	531.4	1309

Minas

3007MG	31Gerais	Senador Cortes	316560	3165602	2021	392.7	277.4	283	200.8	1276

Minas

3008MG	31Gerais	Senador Firmino	316570	3165701	7589.8	1474.6	1041.9	1062.8	754.1	2616

Minas	Senador José

3009MG	31Gerais	Bento	316580	3165800	1722	334.6	236.4	241.1	171.1	969

Senador

Minas	Modestino

3010MG	31Gerais	Gonçalves	316590	3165909	4437.4	862.1	609.2	621.3	440.9	2156

Minas	Senhora de

3011MG	31Gerais	Oliveira	316600	3166006	5802.3	1127.3	796.5	812.5	576.5	2976

Minas

3012MG	31Gerais	Senhora do Porto	316610	3166105	3553.2	690.4	487.8	497.5	353.1	1462

Minas	Senhora dos

3013MG	31Gerais	Remédios	316620	3166204	10445.7	2029.5	1434	1462.7	1037.9	3350

Minas

1

2

1

1

1

2

1

4

1

3014MG	31Gerais	Sericita	316630	3166303	7308.7	1420	1003.3	1023.4	726.2	1283

Minas

3015MG	31Gerais	Seritinga	316640	3166402	1840.2	357.5	252.6	257.7	182.8	1346

Minas	Serra Azul de

3016MG	31Gerais	Minas	316650	3166501	4306.6	836.7	591.2	603	427.9	1367

Minas

3017MG	31Gerais	Serra da Saudade	316660	3166600	809.2	157.2	111.1	113.3	80.4	342

Minas

3018MG	31Gerais	Serra dos Aimorés	316670	3166709	8650.2	1680.6	1187.5	1211.2	859.5	1555

Minas

3019MG	31Gerais	Serra do Salitre	316680	3166808	11159.1	2168.1	1531.9	1562.6	1108.8	2297

Minas

3020MG	31Gerais	Serrania	316690	3166907	7695.1	1495.1	1056.4	1077.5	764.6	2536

Minas	Serranópolis de

3021MG	31Gerais	Minas	316695	3166956	4645.1	902.5	637.7	650.4	461.6	1363

Minas

3022MG	31Gerais	Serranos	316700	3167004	2005	389.6	275.2	280.7	199.2	805

Minas

3023MG	31Gerais	Serro	316710	3167103	21155.9	4110.4	2904.3	2962.4	2102.1	6145

Minas

3024MG	31Gerais	Sete Lagoas	316720	3167202	228606.4	44415.9	31383.1	32010.6	22715.1	53645	16

Minas

3025MG	31Gerais	Silveirânia	316730	3167301	2251.7	437.5	309.1	315.3	223.7	2346

Minas

3026MG	31Gerais	Silvianópolis	316740	3167400	6199.6	1204.5	851.1	868.1	616	1924

Minas

3027MG	31Gerais	Simão Pereira	316750	3167509	2605	506.1	357.6	364.8	258.8	1310

Minas

3028MG	31Gerais	Simonésia	316760	3167608	19145.8	3719.8	2628.3	2680.9	1902.4	3672

Minas

3029MG	31Gerais	Sobrália	316770	3167707	5774.9	1122	792.8	808.6	573.8	1050

Minas	Soledade de

3030MG	31Gerais	Minas	316780	3167806	5967.5	1159.4	819.2	835.6	593	2114

Minas

3031MG	31Gerais	Tabuleiro	316790	3167905	3977.9	772.9	546.1	557	395.3	2151

Minas

3032MG	31Gerais	Taiobeiras	316800	3168002	32800.8	6372.9	4502.9	4592.9	3259.2	9395

Minas

3033MG	31Gerais	Taparuba	316805	3168051	3163.4	614.6	434.3	443	314.3	1082

Minas

3034MG	Tapira	316810	3168101	4469.6	868.4	613.6	625.9	444.1	921

3035MG	Tapiraí	316820	3168200	1897.6	368.7	260.5	265.7	188.6	738

Taquaraçu de

3036MG	Minas	316830	3168309	3972.7	771.9	545.4	556.3	394.7	873

3037MG	Tarumirim	316840	3168408	14487.1	2814.7	1988.8	2028.6	1439.5	2845

3038MG	Teixeiras	316850	3168507	11638.3	2261.2	1597.7	1629.7	1156.4	5552

3039MG	Teófilo Otoni	316860	3168606	139139	27033.3	19101	19482.9	13825.3	35066	11

3040MG	Timóteo	316870	3168705	86242.2	16756	11839.3	12076	8569.3	17367

3041MG	Tiradentes	316880	3168804	7520.5	1461.2	1032.4	1053.1	747.3	1502

3042MG	Tiros	316890	3168903	6795.1	1320.2	932.8	951.5	675.2	1801

3043MG	Tocantins	316900	3169000	16408.4	3188	2252.5	2297.6	1630.4	4247

3044MG	Tocos do Moji	316905	3169059	4069.2	790.6	558.6	569.8	404.3	1681

3045MG	Toledo	316910	3169109	6064.7	1178.3	832.6	849.2	602.6	817

3046MG	Tombos	316920	3169208	8955.2	1739.9	1229.4	1254	889.8	4855

3047MG	Três Corações	316930	3169307	76786.1	14918.8	10541.2	10752	7629.7	14584

3048MG	Três Marias	316935	3169356	30543.3	5934.3	4193	4276.8	3034.9	9002

3049MG	Três Pontas	316940	3169406	55870.8	10855.1	7669.9	7823.3	5551.5	12929

3050MG	Tumiritinga	316950	3169505	6575.2	1277.5	902.6	920.7	653.3	923

3051MG	Tupaciguara	316960	3169604	25018.1	4860.8	3434.5	3503.2	2485.9	4473

3052MG	Turmalina	316970	3169703	19165	3723.6	2631	2683.6	1904.3	7465

3053MG	Turvolândia	316980	3169802	4893	950.7	671.7	685.1	486.2	1307

3054MG	Ubá	316990	3169901	109289.2	21233.8	15003.2	15303.2	10859.3	27564	10

3055MG	31Gerais	Ubaí	317000	3170008	12221.5	2374.5	1677.8	1711.3	1214.4	2089

Minas

3056MG	31Gerais	Ubaporanga	317005	3170057	12390.3	2407.3	1700.9	1734.9	1231.1	2750

Minas

3057MG	31Gerais	Uberaba	317010	3170107	317196.5	61628.1	43544.7	44415.4	31517.7	98014	29

Minas

3058MG	31Gerais	Uberlândia	317020	3170206	652000.5	126677.2	89506.6	91296.2	64784.9	217604	77

Minas

3059MG	31Gerais	Umburatiba	317030	3170305	2695	523.6	370	377.4	267.8	1249

Minas

3060MG	31Gerais	Unaí	317040	3170404	81690.3	15871.6	11214.4	11438.7	8117	7585

Minas

3061MG	31Gerais	União de Minas	317043	3170438	4420.6	858.9	606.9	619	439.2	745

Minas

3062MG	31Gerais	Uruana de Minas	317047	3170479	3290.3	639.3	451.7	460.7	326.9	658

Minas

3063MG	31Gerais	Urucânia	317050	3170503	10450.9	2030.5	1434.7	1463.4	1038.4	3643

Minas

3064MG	31Gerais	Urucuia	317052	3170529	15284.6	2969.6	2098.3	2140.2	1518.7	1797

Minas

3065MG	31Gerais	Vargem Alegre	317057	3170578	6550.5	1272.7	899.3	917.2	650.9	2221

Minas

3066MG	31Gerais	Vargem Bonita	317060	3170602	2185.2	424.6	300	306	217.1	1431

Minas	Vargem Grande

3067MG	31Gerais	do Rio Pardo	317065	3170651	4919.6	955.8	675.4	688.9	488.8	1335

Minas

3068MG	31Gerais	Varginha	317070	3170701	130399.5	25335.3	17901.2	18259.2	12956.9	30633

Minas

3069MG	31Gerais	Varjão de Minas	317075	3170750	6636.4	1289.4	911	929.3	659.4	1538

Minas

3070MG	31Gerais	Várzea da Palma	317080	3170800	37963.5	7375.9	5211.6	5315.8	3772.2	7112

Minas

3071MG	31Gerais	Varzelândia	317090	3170909	19449.6	3778.9	2670	2723.4	1932.6	3168

Minas

3072MG	31Gerais	Vazante	317100	3171006	20372	3958.1	2796.7	2852.6	2024.2	4162

Minas

3073MG	31Gerais	Verdelândia	317103	3171030	8918.3	1732.7	1224.3	1248.8	886.2	1400

Minas

3074MG	31Gerais	Veredinha	317107	3171071	5697.2	1106.9	782.1	797.7	566.1	1680

Minas

3075MG	31Gerais	Veríssimo	317110	3171105	3765.9	731.7	517	527.3	374.2	648

1

4

2

3

1

1

1

1

7

2

1

4

5

1

5

2

1

1

1

4

1

2

1

2

Minas

3076MG	31Gerais	Vermelho Novo	317115	3171154	4817.7	936	661.4	674.6	478.7	1115

Minas

3077MG	31Gerais	Vespasiano	317120	3171204	116526.2	22639.9	15996.7	16316.6	11578.4	14846

Minas

3078MG	31Gerais	Viçosa	317130	3171303	76192.4	14803.4	10459.7	10668.8	7570.7	19764

Minas

3079MG	31Gerais	Vieiras	317140	3171402	3720.6	722.9	510.8	521	369.7	1783

Minas

3080MG	31Gerais	Mathias Lobato	317150	3171501	3334.5	647.9	457.8	466.9	331.3	454

Minas

3081MG	31Gerais	Virgem da Lapa	317160	3171600	13851	2691.1	1901.5	1939.5	1376.3	2735

Minas

3082MG	31Gerais	Virgínia	317170	3171709	8754.5	1700.9	1201.8	1225.8	869.9	3983

Minas

3083MG	31Gerais	Virginópolis	317180	3171808	10675.9	2074.2	1465.6	1494.9	1060.8	2621

Minas

3084MG	31Gerais	Virgolândia	317190	3171907	5599.7	1088	768.7	784.1	556.4	2339

Minas	Visconde do Rio

3085MG	31Gerais	Branco	317200	3172004	40557.1	7879.8	5567.7	5679	4029.9	16437

Minas

3086MG	31Gerais	Volta Grande	317210	3172103	5217.7	1013.7	716.3	730.6	518.4	944

Minas

3087MG	31Gerais	Wenceslau Braz	317220	3172202	2584.5	502.1	354.8	361.9	256.8	847

Espirito

3088ES	32Santo	Afonso Cláudio	320010	3200102	31659.4	5779.1	4282.3	3896.9	2942.8	9712

Espirito

3089ES	32Santo	Águia Branca	320013	3200136	9815.9	1791.8	1327.7	1208.2	912.4	1148

Espirito	Água Doce do

3090ES	32Santo	Norte	320016	3200169	11737.5	2142.6	1587.6	1444.7	1091	2122

Espirito

3091ES	32Santo	Alegre	320020	3200201	31377	5727.6	4244.1	3862.1	2916.5	5361

Espirito

3092ES	32Santo	Alfredo Chaves	320030	3200300	14596.9	2664.5	1974.4	1796.7	1356.8	3986

Espirito

3093ES	32Santo	Alto Rio Novo	320035	3200359	7733	1411.6	1046	951.8	718.8	1677

Espirito

3094ES	32Santo	Anchieta	320040	3200409	26884.6	4907.5	3636.4	3309.1	2498.9	3489

Espirito

3095ES	32Santo	Apiacá	320050	3200508	7728	1410.7	1045.3	951.2	718.3	1108

Espirito

3096ES	32Santo	Aracruz	320060	3200607	92502	16885.3	12511.8	11385.8	8598.1	13725

Espirito

3097ES	32Santo	Atilio Vivacqua	320070	3200706	10980.3	2004.3	1485.2	1351.5	1020.6	3210

Espirito

3098ES	32Santo	Baixo Guandu	320080	3200805	30670.9	5598.7	4148.5	3775.2	2850.9	7540

Espirito	Barra de São

3099ES	32Santo	Francisco	320090	3200904	43456.1	7932.5	5877.9	5348.9	4039.3	4082

Espirito

3100ES	32Santo	Boa Esperança	320100	3201001	14931.6	2725.6	2019.6	1837.9	1387.9	3357

Espirito	Bom Jesus do

3101ES	32Santo	Norte	320110	3201100	9920.7	1810.9	1341.9	1221.1	922.1	1375

Espirito

3102ES	32Santo	Brejetuba	320115	3201159	12435.4	2270	1682	1530.6	1155.9	1699

Espirito	Cachoeiro de

3103ES	32Santo	Itapemirim	320120	3201209	203224.4	37096.6	27488.1	25014.2	18889.8	47068	10

Espirito

3104ES	32Santo	Cariacica	320130	3201308	372035.2	67911.3	50321.5	45792.6	34580.9	96519	30

Espirito

3105ES	32Santo	Castelo	320140	3201407	36866.2	6729.6	4986.5	4537.7	3426.7	8204

Espirito

3106ES	32Santo	Colatina	320150	3201506	119446.8	21803.8	16156.4	14702.3	11102.7	48331	18

Espirito	Conceição da

3107ES	32Santo	Barra	320160	3201605	30331.3	5536.7	4102.6	3733.4	2819.3	2204

Espirito	Conceição do

3108ES	32Santo	Castelo	320170	3201704	12439.8	2270.8	1682.6	1531.2	1156.3	3925

Espirito	Divino de São

3109ES	32Santo	Lourenço	320180	3201803	4537	828.2	613.7	558.4	421.7	712

Espirito

3110ES	32Santo	Domingos Martins	320190	3201902	33545.6	6123.4	4537.4	4129	3118.1	7272

Espirito	Dores do Rio

3111ES	32Santo	Preto	320200	3202009	6716	1225.9	908.4	826.7	624.3	1433

Espirito

3112ES	32Santo	Ecoporanga	320210	3202108	23675.6	4321.7	3202.4	2914.2	2200.7	2257

Espirito

3113ES	32Santo	Fundão	320220	3202207	19444	3549.3	2630	2393.3	1807.3	4423

Espirito	Governador

3114ES	32Santo	Lindenberg	320225	3202256	11960.9	2183.3	1617.8	1472.2	1111.8	4060

Espirito

3115ES	32Santo	Guaçuí	320230	3202306	29895.8	5457.2	4043.7	3679.8	2778.8	7838

Espirito

3116ES	32Santo	Guarapari	320240	3202405	116635.3	21290.6	15776.1	14356.3	10841.3	15746

Espirito

3117ES	32Santo	Ibatiba	320245	3202454	24581.2	4487.1	3324.9	3025.6	2284.8	1955

Espirito

3118ES	32Santo	Ibiraçu	320250	3202504	12039	2197.6	1628.4	1481.8	1119	3200

Espirito

3119ES	32Santo	Ibitirama	320255	3202553	9155.3	1671.2	1238.3	1126.9	851	1112

Espirito

3120ES	32Santo	Iconha	320260	3202603	13433.7	2452.2	1817	1653.5	1248.7	4576

Espirito

3121ES	32Santo	Irupi	320265	3202652	12754.6	2328.2	1725.2	1569.9	1185.5	891

Espirito

3122ES	32Santo	Itaguaçu	320270	3202702	14464.1	2640.3	1956.4	1780.3	1344.4	5581

Espirito

3123ES	32Santo	Itapemirim	320280	3202801	33304.4	6079.4	4504.8	4099.3	3095.7	3676

Espirito

3124ES	32Santo	Itarana	320290	3202900	11014	2010.5	1489.8	1355.7	1023.8	5340

Espirito

3125ES	32Santo	Iúna	320300	3203007	28835.7	5263.7	3900.3	3549.3	2680.3	1833

Espirito

3126ES	32Santo	Jaguaré	320305	3203056	27874.6	5088.2	3770.3	3431	2591	3786

Espirito

3127ES	32Santo	Jerônimo Monteiro	320310	3203106	11609.9	2119.3	1570.4	1429	1079.1	2838

Espirito

3128ES	32Santo	João Neiva	320313	3203130	16592.6	3028.8	2244.3	2042.3	1542.3	3629

Espirito

3129ES	32Santo	Laranja da Terra	320316	3203163	11155	2036.2	1508.8	1373	1036.9	1938

Espirito

3130ES	32Santo	Linhares	320320	3203205	159215.2	29063.1	21535.4	19597.3	14799.2	27623

Espirito

3131ES	32Santo	Mantenópolis	320330	3203304	14728.8	2688.6	1992.2	1812.9	1369.1	2034

Espirito

3132ES	32Santo	Marataízes	320332	3203320	36940.2	6743.1	4996.5	4546.8	3433.6	6156

Espirito

3133ES	32Santo	Marechal Floriano	320334	3203346	15702.7	2866.4	2123.9	1932.8	1459.6	2795

Espirito

3134ES	32Santo	Marilândia	320335	3203353	12087.4	2206.4	1634.9	1487.8	1123.5	6224

Espirito

3135ES	32Santo	Mimoso do Sul	320340	3203403	26672.7	4868.8	3607.7	3283.1	2479.2	5188

Espirito

3136ES	32Santo	Montanha	320350	3203502	18739.5	3420.7	2534.7	2306.6	1741.8	4046

1

1

1

1

3

1

1

1

1

1

1

9

1

2

1

1

5

1

1

1

1

5

2

2

6

1

2

3

1

8

1

Espirito

3137ES	32Santo	Mucurici	320360	3203601	5741.1	1048	776.5	706.7	533.6	1476

Espirito

3138ES	32Santo	Muniz Freire	320370	3203700	18453	3368.4	2496	2271.3	1715.2	2658

Espirito

3139ES	32Santo	Muqui	320380	3203809	15229.3	2780	2059.9	1874.5	1415.6	2618

Espirito

3140ES	32Santo	Nova Venécia	320390	3203908	49009.8	8946.2	6629.1	6032.5	4555.5	15566

Espirito

3141ES	32Santo	Pancas	320400	3204005	22822.1	4165.9	3086.9	2809.1	2121.3	5536

Espirito

3142ES	32Santo	Pedro Canário	320405	3204054	25458.3	4647.2	3443.5	3133.6	2366.4	1252

Espirito

3143ES	32Santo	Pinheiros	320410	3204104	25899.6	4727.7	3503.2	3187.9	2407.4	3969

Espirito

3144ES	32Santo	Piúma	320420	3204203	20171.7	3682.1	2728.4	2482.9	1875	3027

Espirito

3145ES	32Santo	Ponto Belo	320425	3204252	7548.4	1377.9	1021	929.1	701.6	1895

Espirito	Presidente

3146ES	32Santo	Kennedy	320430	3204302	11097.5	2025.7	1501	1366	1031.5	3877

Espirito

3147ES	32Santo	Rio Bananal	320435	3204351	18690.5	3411.8	2528.1	2300.6	1737.3	1012

Espirito

3148ES	32Santo	Rio Novo do Sul	320440	3204401	11745.1	2144	1588.6	1445.7	1091.7	4277

Espirito

3149ES	32Santo	Santa Leopoldina	320450	3204500	12561.9	2293	1699.1	1546.2	1167.6	2773

Espirito	Santa Maria de

3150ES	32Santo	Jetibá	320455	3204559	37822.3	6904.1	5115.8	4655.4	3515.6	18148

Espirito

3151ES	32Santo	Santa Teresa	320460	3204609	23138.4	4223.7	3129.7	2848	2150.7	8012

Espirito	São Domingos do

3152ES	32Santo	Norte	320465	3204658	8487.2	1549.3	1148	1044.7	788.9	1645

Espirito	São Gabriel da

3153ES	32Santo	Palha	320470	3204708	35366.4	6455.8	4783.7	4353.1	3287.3	6395

Espirito	São José do

3154ES	32Santo	Calçado	320480	3204807	10738.8	1960.3	1452.5	1321.8	998.2	1956

Espirito

3155ES	32Santo	São Mateus	320490	3204906	121332	22147.9	16411.4	14934.4	11277.9	20136

Espirito	São Roque do

3156ES	32Santo	Canaã	320495	3204955	12066	2202.5	1632	1485.2	1121.5	3374

Espirito

3157ES	32Santo	Serra	320500	3205002	469882.1	85772.3	63556.3	57836.2	43675.9	71535	23

Espirito

3158ES	32Santo	Sooretama	320501	3205010	27208.3	4966.6	3680.2	3349	2529	737

Espirito

3159ES	32Santo	Vargem Alta	320503	3205036	20620.8	3764.1	2789.2	2538.1	1916.7	4795

Espirito	Venda Nova do

3160ES	32Santo	Imigrante	320506	3205069	23106.4	4217.8	3125.4	2844.1	2147.8	7874

Espirito

3161ES	32Santo	Viana	320510	3205101	72518.6	13237.5	9808.9	8926.1	6740.7	12764

Espirito

3162ES	32Santo	Vila Pavão	320515	3205150	9130.6	1666.7	1235	1123.9	848.7	2136

Espirito

3163ES	32Santo	Vila Valério	320517	3205176	14292.8	2609	1933.2	1759.3	1328.5	3055

Espirito

3164ES	32Santo	Vila Velha	320520	3205200	460242.6	84012.7	62252.4	56649.8	42779.9	87258	26

Espirito

3165ES	32Santo	Vitória	320530	3205309	349079.5	63721	47216.5	42967.1	32447.2	102765	32

Rio de

3166RJ	33Janeiro	Angra dos Reis	330010	3300100	186499.8	39534.2	26197.6	23392.4	16577.9	31297

Rio de

3167RJ	33Janeiro	Aperibé	330015	3300159	10960.3	2323.4	1539.6	1374.7	974.3	3200

Rio de

3168RJ	33Janeiro	Araruama	330020	3300209	121997.4	25861	17137	15302	10844.3	28511

Rio de

3169RJ	33Janeiro	Areal	330022	3300225	11949.5	2533.1	1678.5	1498.8	1062.2	2076

Rio de	Armação dos

3170RJ	33Janeiro	Búzios	330023	3300233	31381.3	6652.2	4408.1	3936.1	2789.5	8627

Rio de

3171RJ	33Janeiro	Arraial do Cabo	330025	3300258	28943.6	6135.5	4065.7	3630.4	2572.8	5092

Rio de

3172RJ	33Janeiro	Barra do Piraí	330030	3300308	97041.3	20570.8	13631.4	12171.8	8625.9	16358

Rio de

3173RJ	33Janeiro	Barra Mansa	330040	3300407	180209.7	38200.9	25314.1	22603.4	16018.7	42127	13

Rio de

3174RJ	33Janeiro	Belford Roxo	330045	3300456	486347.6	103096	68317.2	61001.9	43231.2	63245	19

Rio de

3175RJ	33Janeiro	Bom Jardim	330050	3300506	26268.8	5568.5	3690	3294.9	2335	3840

Rio de	Bom Jesus do

3176RJ	33Janeiro	Itabapoana	330060	3300605	36049.9	7641.9	5063.9	4521.7	3204.5	10073

Rio de

3177RJ	33Janeiro	Cabo Frio	330070	3300704	205623.7	43588.1	28884	25791.1	18277.8	65730	22

Rio de	Cachoeiras de

3178RJ	33Janeiro	Macacu	330080	3300803	56316.2	11937.9	7910.7	7063.7	5005.9	2145

Rio de

3179RJ	33Janeiro	Cambuci	330090	3300902	15001.3	3180	2107.2	1881.6	1333.5	3592

Rio de

3180RJ	33Janeiro	Carapebus	330093	3300936	14841	3146	2084.7	1861.5	1319.2	731

Rio de	Comendador Levy

3181RJ	33Janeiro	Gasparian	330095	3300951	8303	1760.1	1166.3	1041.4	738	1592

Rio de	Campos dos

3182RJ	33Janeiro	Goytacazes	330100	3301009	483438.9	102479.4	67908.7	60637	42972.6	100649	35

Rio de

3183RJ	33Janeiro	Cantagalo	330110	3301108	19863.9	4210.7	2790.3	2491.5	1765.7	6197

Rio de

3184RJ	33Janeiro	Cardoso Moreira	330115	3301157	12624.2	2676.1	1773.3	1583.4	1122.2	2894

Rio de

3185RJ	33Janeiro	Carmo	330120	3301207	18124.5	3842	2545.9	2273.3	1611.1	4906

Rio de

3186RJ	33Janeiro	Casimiro de Abreu	330130	3301306	39790.3	8434.7	5589.3	4990.8	3536.9	2304

Rio de	Conceição de

3187RJ	33Janeiro	Macabu	330140	3301405	22132	4691.5	3108.9	2776	1967.3	701

Rio de

3188RJ	33Janeiro	Cordeiro	330150	3301504	21073.8	4467.2	2960.2	2643.3	1873.2	5582

Rio de

3189RJ	33Janeiro	Duas Barras	330160	3301603	11147.3	2363	1565.9	1398.2	990.9	1497

Rio de

3190RJ	33Janeiro	Duque de Caxias	330170	3301702	883021.7	187182.9	124038.1	110756.1	78491.3	158277	41

Rio de	Engenheiro Paulo

3191RJ	33Janeiro	de Frontin	330180	3301801	13569.4	2876.4	1906.1	1702	1206.2	3233

Rio de

3192RJ	33Janeiro	Guapimirim	330185	3301850	55953.5	11861	7859.8	7018.2	4973.7	2123

Rio de

3193RJ	33Janeiro	Iguaba Grande	330187	3301876	25588.6	5424.3	3594.4	3209.5	2274.6	2610

Rio de

3194RJ	33Janeiro	Itaboraí	330190	3301900	228488.2	48434.9	32095.7	28658.9	20310.2	40232	12

Rio de

3195RJ	33Janeiro	Itaguaí	330200	3302007	118765.8	25176	16683	14896.6	10557	6904

Rio de

3196RJ	33Janeiro	Italva	330205	3302056	14567.1	3087.9	2046.2	1827.1	1294.9	2458

Rio de

3197RJ	33Janeiro	Itaocara	330210	3302106	22905.8	4855.6	3217.6	2873	2036.1	7693

Rio de

8

2

1

2

3

1

2

1

1

2

2

3

1

5

2

5

1

1

5

1

3

1

9

3

3198RJ	33Janeiro	Itaperuna	330220	3302205	99049.8	20996.6	13913.5	12423.7	8804.5	33137	11

Rio de

3199RJ	33Janeiro	Itatiaia	330225	3302254	30177	6396.9	4239	3785.1	2682.4	2891

Rio de

3200RJ	33Janeiro	Japeri	330227	3302270	99718.2	21138.3	14007.4	12507.5	8863.9	9562

Rio de

3201RJ	33Janeiro	Laje do Muriaé	330230	3302304	7360.5	1560.3	1033.9	923.2	654.3	837

Rio de

3202RJ	33Janeiro	Macaé	330240	3302403	231771.9	49131	32557	29070.8	20602.1	4936

Rio de

3203RJ	33Janeiro	Macuco	330245	3302452	5405.6	1145.9	759.3	678	480.5	1723

Rio de

3204RJ	33Janeiro	Magé	330250	3302502	235019.1	49819.3	33013.1	29478.1	20890.7	15783

Rio de

3205RJ	33Janeiro	Mangaratiba	330260	3302601	40412.6	8566.7	5676.8	5068.9	3592.3	1951

Rio de

3206RJ	33Janeiro	Maricá	330270	3302700	144502.8	30631.7	20298.3	18124.8	12844.8	7997

Rio de

3207RJ	33Janeiro	Mendes	330280	3302809	18162.2	3850	2551.2	2278.1	1614.4	3088

Rio de

3208RJ	33Janeiro	Mesquita	330285	3302858	171231.1	36297.6	24052.8	21477.3	15220.6	18519

Rio de

3209RJ	33Janeiro	Miguel Pereira	330290	3302908	24933.8	5285.5	3502.5	3127.4	2216.4	2276

Rio de

3210RJ	33Janeiro	Miracema	330300	3303005	26818.1	5684.9	3767.1	3363.8	2383.8	5024

Rio de

3211RJ	33Janeiro	Natividade	330310	3303104	15094.6	3199.8	2120.3	1893.3	1341.8	3020

Rio de

3212RJ	33Janeiro	Nilópolis	330320	3303203	158941.9	33692.5	22326.6	19935.9	14128.3	6993

Rio de

3213RJ	33Janeiro	Niterói	330330	3303302	497773.6	105518	69922.3	62435	44246.8	55638	14

Rio de

3214RJ	33Janeiro	Nova Friburgo	330340	3303401	185278.6	39275.4	26026.1	23239.2	16469.3	43213	12

Rio de

3215RJ	33Janeiro	Nova Iguaçu	330350	3303500	805161.9	170678.2	113101.1	100990.3	71570.4	99475	29

Rio de

3216RJ	33Janeiro	Paracambi	330360	3303609	49482.3	10489.3	6950.8	6206.5	4398.5	16539

Rio de

3217RJ	33Janeiro	Paraíba do Sul	330370	3303708	42454.8	8999.6	5963.6	5325	3773.8	2575

Rio de

3218RJ	33Janeiro	Paty do Alferes	330385	3303856	26905.2	5703.4	3779.4	3374.7	2391.6	4731

Rio de

3219RJ	33Janeiro	Petrópolis	330390	3303906	299199.2	63424.2	42028.5	37528.1	26595.6	73884	23

Rio de

3220RJ	33Janeiro	Pinheiral	330395	3303955	23841.6	5053.9	3349	2990.4	2119.3	2567

Rio de

3221RJ	33Janeiro	Piraí	330400	3304003	27721.2	5876.3	3894	3477	2464.1	12332

Rio de

3222RJ	33Janeiro	Porciúncula	330410	3304102	18221.4	3862.6	2559.6	2285.5	1619.7	3392

Rio de

3223RJ	33Janeiro	Porto Real	330411	3304110	18114.5	3839.9	2544.5	2272.1	1610.2	3061

Rio de

3224RJ	33Janeiro	Quatis	330412	3304128	13500.8	2861.9	1896.5	1693.4	1200.1	1396

Rio de

3225RJ	33Janeiro	Queimados	330414	3304144	143511.3	30421.5	20159	18000.4	12756.6	36666	11

Rio de

3226RJ	33Janeiro	Quissamã	330415	3304151	22459.5	4761	3154.9	2817.1	1996.4	2285

Rio de

3227RJ	33Janeiro	Resende	330420	3304201	125037.1	26505.4	17564	15683.2	11114.5	31439

Rio de

3228RJ	33Janeiro	Rio Bonito	330430	3304300	57607.9	12211.7	8092.2	7225.7	5120.7	11685

| 3229RJ | Rio de 33Janeiro | Rio Claro | 330440 | 3304409 | 17866.9 | 3787.4 | 2509.8 | 2241 | 1588.2 | 545 |  |
| --- | --- | --- | --- | --- | --- | --- | --- | --- | --- | --- | --- |
| 3230RJ | Rio de 33Janeiro | Rio das Flores | 330450 | 3304508 | 8884.3 | 1883.3 | 1248 | 1114.3 | 789.7 | 3040 |  |
| 3231RJ | Rio de 33Janeiro | Rio das Ostras | 330452 | 3304524 | 128855.1 | 27314.7 | 18100.3 | 16162.1 | 11453.9 | 7240 |  |
| 3232RJ | Rio de 33Janeiro | Rio de Janeiro | 330455 | 3304557 | 6485385.4 | 1374772 | 911002.1 | 813452.6 | 576482.1 | 723485 | 160 |
| 3233RJ | Rio de 33Janeiro | Santa Maria  Madalena | 330460 | 3304607 | 10287.9 | 2180.8 | 1445.1 | 1290.4 | 914.5 | 2218 |  |
| 3234RJ | Rio de 33Janeiro | Santo Antônio de  Pádua | 330470 | 3304706 | 41291.7 | 8753 | 5800.2 | 5179.2 | 3670.4 | 5377 |  |
| 3235RJ | Rio de 33Janeiro | São Francisco de  Itabapoana | 330475 | 3304755 | 41497.8 | 8796.7 | 5829.2 | 5205 | 3688.7 | 4458 |  |
| 3236RJ | Rio de 33Janeiro | São Fidélis | 330480 | 3304805 | 37861.1 | 8025.8 | 5318.3 | 4748.9 | 3365.5 | 4510 |  |
| 3237RJ | Rio de 33Janeiro | São Gonçalo | 330490 | 3304904 | 1037582.1 | 219946.7 | 145749.2 | 130142.4 | 92230.1 | 96110 | 25 |
| 3238RJ | Rio de 33Janeiro | São João da Barra | 330500 | 3305000 | 34450 | 7302.7 | 4839.2 | 4321 | 3062.2 | 1471 |  |
| 3239RJ | Rio de 33Janeiro | São João de Meriti | 330510 | 3305109 | 462554.5 | 98052.3 | 64975 | 58017.5 | 41116.2 | 68516 | 20 |
| 3240RJ | Rio de 33Janeiro | São José de Ubá | 330513 | 3305133 | 7116.2 | 1508.5 | 999.6 | 892.6 | 632.6 | 1652 |  |
| 3241RJ | Rio de 33Janeiro | São José do Vale do Rio Preto | 330515 | 3305158 | 20921.7 | 4435 | 2938.9 | 2624.2 | 1859.7 | 4388 |  |
| 3242RJ | Rio de 33Janeiro | São Pedro da  Aldeia | 330520 | 3305208 | 96075.1 | 20366 | 13495.7 | 12050.6 | 8540.1 | 17823 |  |
| 3243RJ | Rio de 33Janeiro | São Sebastião do  Alto | 330530 | 3305307 | 9074.9 | 1923.7 | 1274.8 | 1138.3 | 806.7 | 2427 |  |
| 3244RJ | Rio de 33Janeiro | Sapucaia | 330540 | 3305406 | 17728.5 | 3758.1 | 2490.3 | 2223.7 | 1575.9 | 2999 |  |
| 3245RJ | Rio de 33Janeiro | Saquarema | 330550 | 3305505 | 81615.5 | 17300.9 | 11464.5 | 10236.9 | 7254.8 | 9242 |  |
| 3246RJ | Rio de 33Janeiro | Seropédica | 330555 | 3305554 | 82088.2 | 17401.1 | 11530.9 | 10296.2 | 7296.8 | 3040 |  |
| 3247RJ | Rio de 33Janeiro | Silva Jardim | 330560 | 3305604 | 21415.4 | 4539.6 | 3008.2 | 2686.1 | 1903.6 | 1872 |  |
| 3248RJ | Rio de 33Janeiro | Sumidouro | 330570 | 3305703 | 15170.6 | 3215.9 | 2131 | 1902.8 | 1348.5 | 1641 |  |
| 3249RJ | Rio de 33Janeiro | Tanguá | 330575 | 3305752 | 32352.3 | 6858 | 4544.5 | 4057.9 | 2875.8 | 1539 |  |
| 3250RJ | Rio de 33Janeiro | Teresópolis | 330580 | 3305802 | 172560.2 | 36579.3 | 24239.5 | 21644 | 15338.8 | 41466 | 14 |
| 3251RJ | Rio de 33Janeiro | Três Rios | 330600 | 3306008 | 79241.3 | 16797.6 | 11131 | 9939.1 | 7043.7 | 26189 |  |
| 3252RJ | Rio de 33Janeiro | Valença | 330610 | 3306107 | 73803.4 | 15644.8 | 10367.2 | 9257.1 | 6560.3 | 26773 |  |
| 3253RJ | Rio de 33Janeiro | Varre-Sai | 330615 | 3306156 | 10201 | 2162.4 | 1432.9 | 1279.5 | 906.8 | 1537 |  |
| 3254RJ | Rio de 33Janeiro | Vassouras | 330620 | 3306206 | 35471.2 | 7519.2 | 4982.6 | 4449.1 | 3153 | 4535 |  |
| 3255RJ | Rio de 33Janeiro | Volta Redonda | 330630 | 3306305 | 263761.5 | 55912.2 | 37050.6 | 33083.2 | 23445.6 | 123554 | 39 |
| 3256SP | 35São Paulo | Adamantina | 350010 | 3500105 | 34678.6 | 6814.3 | 4877.2 | 3812.6 | 2643 | 15422 |  |
| 3257SP | 35São Paulo | Adolfo | 350020 | 3500204 | 3589.3 | 705.3 | 504.8 | 394.6 | 273.6 | 2902 |  |
| 3258SP | 35São Paulo | Aguaí | 350030 | 3500303 | 34388.1 | 6757.3 | 4836.3 | 3780.7 | 2620.8 | 7370 |  |
| 3259SP | 35São Paulo | Águas da Prata | 350040 | 3500402 | 7929.3 | 1558.1 | 1115.2 | 871.8 | 604.3 | 2301 |  |
| 3260SP | 35São Paulo | Águas de Lindóia | 350050 | 3500501 | 18091.5 | 3555 | 2544.4 | 1989 | 1378.8 | 9075 |  |
| 3261SP | 35São Paulo | Águas de Santa  Bárbara | 350055 | 3500550 | 5872.1 | 1153.9 | 825.9 | 645.6 | 447.5 | 1378 |  |
| 3262SP | 35São Paulo | Águas de São  Pedro | 350060 | 3500600 | 3082.9 | 605.8 | 433.6 | 338.9 | 235 | 1474 |  |
| 3263SP | 35São Paulo | Agudos | 350070 | 3500709 | 36087.2 | 7091.1 | 5075.3 | 3967.5 | 2750.3 | 7208 |  |
| 3264SP | 35São Paulo | Alambari | 350075 | 3500758 | 5466.5 | 1074.2 | 768.8 | 601 | 416.6 | 2355 |  |
| 3265SP | 35São Paulo | Alfredo Marcondes | 350080 | 3500808 | 4053.2 | 796.5 | 570 | 445.6 | 308.9 | 2453 |  |
| 3266SP | 35São Paulo | Altair | 350090 | 3500907 | 4010.4 | 788 | 564 | 440.9 | 305.6 | 2234 |  |
| 3267SP | 35São Paulo | Altinópolis | 350100 | 3501004 | 16009.2 | 3145.8 | 2251.5 | 1760.1 | 1220.1 | 7191 |  |
| 3268SP | 35São Paulo | Alto Alegre | 350110 | 3501103 | 4134.6 | 812.4 | 581.5 | 454.6 | 315.1 | 3009 |  |
| 3269SP | 35São Paulo | Alumínio | 350115 | 3501152 | 17827.8 | 3503.2 | 2507.3 | 1960 | 1358.7 | 3805 |  |
| 3270SP | 35São Paulo | Álvares Florence | 350120 | 3501202 | 3825.7 | 751.8 | 538 | 420.6 | 291.6 | 3340 |  |
| 3271SP | 35São Paulo | Álvares Machado | 350130 | 3501301 | 24372.5 | 4789.2 | 3427.7 | 2679.6 | 1857.5 | 6824 |  |
| 3272SP | 35São Paulo | Álvaro de  Carvalho | 350140 | 3501400 | 4962.5 | 975.1 | 697.9 | 545.6 | 378.2 | 696 |  |
| 3273SP | 35São Paulo | Alvinlândia | 350150 | 3501509 | 3130.2 | 615.1 | 440.2 | 344.1 | 238.6 | 995 |  |
| 3274SP | 35São Paulo | Americana | 350160 | 3501608 | 226134.3 | 44435.4 | 31803.5 | 24861.6 | 17234.3 | 62142 | 21 |
| 3275SP | 35São Paulo | Américo  Brasiliense | 350170 | 3501707 | 37625.8 | 7393.5 | 5291.7 | 4136.6 | 2867.6 | 10731 |  |
| 3276SP | 35São Paulo | Américo de  Campos | 350180 | 3501806 | 5877.9 | 1155 | 826.7 | 646.2 | 448 | 3166 |  |
| 3277SP | 35São Paulo | Amparo | 350190 | 3501905 | 69396.3 | 13636.4 | 9759.9 | 7629.6 | 5288.9 | 19478 |  |
| 3278SP | 35São Paulo | Analândia | 350200 | 3502002 | 4661.9 | 916.1 | 655.6 | 512.5 | 355.3 | 1515 |  |
| 3279SP | 35São Paulo | Andradina | 350210 | 3502101 | 56655.4 | 11132.8 | 7968 | 6228.8 | 4317.9 | 25738 |  |
| 3280SP | 35São Paulo | Angatuba | 350220 | 3502200 | 23826.9 | 4682 | 3351 | 2619.6 | 1815.9 | 7436 |  |
| 3281SP | 35São Paulo | Anhembi | 350230 | 3502309 | 6208.8 | 1220 | 873.2 | 682.6 | 473.2 | 1573 |  |
| 3282SP | 35São Paulo | Anhumas | 350240 | 3502408 | 3947.8 | 775.7 | 555.2 | 434 | 300.9 | 1134 |  |
| 3283SP | 35São Paulo | Aparecida | 350250 | 3502507 | 35841 | 7042.8 | 5040.7 | 3940.4 | 2731.5 | 4665 |  |
| 3284SP | 35São Paulo | Aparecida d'Oeste | 350260 | 3502606 | 4365.8 | 857.9 | 614 | 480 | 332.7 | 2972 |  |
| 3285SP | 35São Paulo | Apiaí | 350270 | 3502705 | 25009.8 | 4914.4 | 3517.4 | 2749.6 | 1906.1 | 3831 |  |
| 3286SP | 35São Paulo | Araçariguama | 350275 | 3502754 | 19732.1 | 3877.4 | 2775.1 | 2169.4 | 1503.8 | 4422 |  |
| 3287SP | 35São Paulo | Araçatuba | 350280 | 3502804 | 190409.2 | 37415.4 | 26779.1 | 20933.9 | 14511.6 | 70063 | 23 |
| 3288SP | 35São Paulo | Araçoiaba da  Serra | 350290 | 3502903 | 30777.8 | 6047.8 | 4328.6 | 3383.8 | 2345.7 | 6163 |  |
| 3289SP | 35São Paulo | Aramina | 350300 | 3503000 | 5417.4 | 1064.5 | 761.9 | 595.6 | 412.9 | 2807 |  |
| 3290SP | 35São Paulo | Arandu | 350310 | 3503109 | 6284.3 | 1234.9 | 883.8 | 690.9 | 478.9 | 1109 |  |
| 3291SP | 35São Paulo | Arapeí | 350315 | 3503158 | 2502 | 491.6 | 351.9 | 275.1 | 190.7 | 1273 |  |
| 3292SP | 35São Paulo | Araraquara | 350320 | 3503208 | 223404.9 | 43899.1 | 31419.7 | 24561.5 | 17026.3 | 72331 | 24 |
| 3293SP | 35São Paulo | Araras | 350330 | 3503307 | 127136.6 | 24982.3 | 17880.5 | 13977.6 | 9689.4 | 28257 |  |
| 3294SP | 35São Paulo | Arco-Íris | 350335 | 3503356 | 1877.5 | 368.9 | 264.1 | 206.4 | 143.1 | 1272 |  |
| 3295SP | 35São Paulo | Arealva | 350340 | 3503406 | 8247.4 | 1620.6 | 1159.9 | 906.7 | 628.6 | 1816 |  |
| 3296SP | 35São Paulo | Areias | 350350 | 3503505 | 3816.7 | 750 | 536.8 | 419.6 | 290.9 | 975 |  |
| 3297SP | 35São Paulo | Areiópolis | 350360 | 3503604 | 10927.3 | 2147.2 | 1536.8 | 1201.4 | 832.8 | 2075 |  |
| 3298SP | 35São Paulo | Ariranha | 350370 | 3503703 | 9150.1 | 1798 | 1286.9 | 1006 | 697.4 | 2241 |  |
| 3299SP | 35São Paulo | Artur Nogueira | 350380 | 3503802 | 49403.1 | 9707.7 | 6948.1 | 5431.5 | 3765.1 | 7575 |  |
| 3300SP |  | Arujá | 350390 | 3503901 | 82612 | 16233.3 | 11618.6 | 9082.5 | 6296.1 | 37254 | 13 |
| 3301SP |  | Aspásia | 350395 | 3503950 | 1830.3 | 359.7 | 257.4 | 201.2 | 139.5 | 1558 |  |
| 3302SP |  | Assis | 350400 | 3504008 | 100318.6 | 19712.6 | 14108.8 | 11029.2 | 7645.6 | 25950 |  |
| 3303SP |  | Atibaia | 350410 | 3504107 | 135323.5 | 26591.1 | 19031.9 | 14877.7 | 10313.4 | 37982 | 12 |
| 3304SP |  | Auriflama | 350420 | 3504206 | 14787.4 | 2905.7 | 2079.7 | 1625.8 | 1127 | 7596 |  |
| 3305SP |  | Avaí | 350430 | 3504305 | 5210.3 | 1023.8 | 732.8 | 572.8 | 397.1 | 673 |  |
| 3306SP |  | Avanhandava | 350440 | 3504404 | 12514.9 | 2459.2 | 1760.1 | 1375.9 | 953.8 | 4287 |  |
| 3307SP |  | Avaré | 350450 | 3504503 | 87285.2 | 17151.5 | 12275.8 | 9596.3 | 6652.3 | 15181 |  |
| 3308SP |  | Bady Bassitt | 350460 | 3504602 | 16100.9 | 3163.8 | 2264.4 | 1770.2 | 1227.1 | 6774 |  |
| 3309SP |  | Balbinos | 350470 | 3504701 | 4699.2 | 923.4 | 660.9 | 516.6 | 358.1 | 443 |  |
| 3310SP |  | Bálsamo | 350480 | 3504800 | 8658.5 | 1701.4 | 1217.7 | 951.9 | 659.9 | 4439 |  |
| 3311SP |  | Bananal | 350490 | 3504909 | 10649.8 | 2092.7 | 1497.8 | 1170.9 | 811.7 | 2668 |  |
| 3312SP |  | Barão de Antonina | 350500 | 3505005 | 3309.4 | 650.3 | 465.4 | 363.8 | 252.2 | 868 |  |
| 3313SP |  | Barbosa | 350510 | 3505104 | 7032 | 1381.8 | 989 | 773.1 | 535.9 | 3241 |  |

1

2

1

1

1

1

5

2

1

6

8

1

4

1

2

3

2

1

2

1

1

1

1

4

1

5

8

2

1

1

1

1

2

9

2

8

2

1

2

2

1

1

| 3314SP |  | Bariri | 350520 | 3505203 | 33597.7 | 6601.9 | 4725.2 | 3693.8 | 2560.6 | 5013 |  |
| --- | --- | --- | --- | --- | --- | --- | --- | --- | --- | --- | --- |
| 3315SP |  | Barra Bonita | 350530 | 3505302 | 35954 | 7065 | 5056.6 | 3952.8 | 2740.2 | 8246 |  |
| 3316SP |  | Barra do Chapéu | 350535 | 3505351 | 5515.7 | 1083.8 | 775.7 | 606.4 | 420.4 | 595 |  |
| 3317SP |  | Barra do Turvo | 350540 | 3505401 | 7759.1 | 1524.7 | 1091.2 | 853 | 591.3 | 1345 |  |
| 3318SP |  | Barretos | 350550 | 3505500 | 117773.9 | 23142.6 | 16563.7 | 12948.3 | 8975.9 | 44419 | 19 |
| 3319SP |  | Barrinha | 350560 | 3505609 | 30782 | 6048.7 | 4329.2 | 3384.2 | 2346 | 1553 |  |
| 3320SP |  | Barueri | 350570 | 3505708 | 258618.5 | 50818.5 | 36372.1 | 28433 | 19710 | 114605 | 45 |
| 3321SP |  | Bastos | 350580 | 3505807 | 20854.5 | 4097.9 | 2933 | 2292.8 | 1589.4 | 8747 |  |
| 3322SP |  | Batatais | 350590 | 3505906 | 59807.3 | 11752.1 | 8411.3 | 6575.3 | 4558.1 | 12219 |  |
| 3323SP | 35São Paulo | Bauru | 350600 | 3506003 | 362392.3 | 71210.1 | 50966.9 | 39842 | 27618.9 | 45536 |  |
| 3324SP | 35São Paulo | Bebedouro | 350610 | 3506102 | 76821.6 | 15095.4 | 10804.2 | 8445.9 | 5854.8 | 27460 | 12 |
| 3325SP | 35São Paulo | Bento de Abreu | 350620 | 3506201 | 2841.3 | 558.3 | 399.6 | 312.4 | 216.5 | 1247 |  |
| 3326SP | 35São Paulo | Bernardino de  Campos | 350630 | 3506300 | 11040.8 | 2169.5 | 1552.8 | 1213.8 | 841.5 | 4172 |  |
| 3327SP | 35São Paulo | Bertioga | 350635 | 3506359 | 55454.1 | 10896.7 | 7799.1 | 6096.7 | 4226.3 | 11734 |  |
| 3328SP | 35São Paulo | Bilac | 350640 | 3506409 | 7574.7 | 1488.4 | 1065.3 | 832.8 | 577.3 | 3505 |  |
| 3329SP | 35São Paulo | Birigui | 350650 | 3506508 | 116708.8 | 22933.3 | 16413.9 | 12831.2 | 8894.7 | 18811 |  |
| 3330SP | 35São Paulo | Biritiba-Mirim | 350660 | 3506607 | 30722 | 6036.9 | 4320.7 | 3377.6 | 2341.4 | 5671 |  |
| 3331SP | 35São Paulo | Boa Esperança do  Sul | 350670 | 3506706 | 14364.5 | 2822.6 | 2020.2 | 1579.3 | 1094.8 | 3758 |  |
| 3332SP | 35São Paulo | Bocaina | 350680 | 3506805 | 11646.8 | 2288.6 | 1638 | 1280.5 | 887.6 | 2260 |  |
| 3333SP | 35São Paulo | Bofete | 350690 | 3506904 | 10700.6 | 2102.7 | 1504.9 | 1176.4 | 815.5 | 4097 |  |
| 3334SP | 35São Paulo | Boituva | 350700 | 3507001 | 54739.8 | 10756.4 | 7698.6 | 6018.2 | 4171.9 | 9741 |  |
| 3335SP | 35São Paulo | Bom Jesus dos  Perdões | 350710 | 3507100 | 22598.9 | 4440.7 | 3178.3 | 2484.6 | 1722.3 | 8476 |  |
| 3336SP | 35São Paulo | Bom Sucesso de  Itararé | 350715 | 3507159 | 3782.1 | 743.2 | 531.9 | 415.8 | 288.2 | 746 |  |
| 3337SP | 35São Paulo | Borá | 350720 | 3507209 | 827.3 | 162.6 | 116.4 | 91 | 63.1 | 474 |  |
| 3338SP | 35São Paulo | Boracéia | 350730 | 3507308 | 4567.3 | 897.5 | 642.3 | 502.1 | 348.1 | 679 |  |
| 3339SP | 35São Paulo | Borborema | 350740 | 3507407 | 15369.3 | 3020.1 | 2161.5 | 1689.7 | 1171.3 | 4823 |  |
| 3340SP | 35São Paulo | Borebi | 350745 | 3507456 | 2482.7 | 487.9 | 349.2 | 273 | 189.2 | 742 |  |
| 3341SP | 35São Paulo | Botucatu | 350750 | 3507506 | 137486.3 | 27016.1 | 19336.1 | 15115.5 | 10478.2 | 47020 | 12 |
| 3342SP | 35São Paulo | Bragança Paulista | 350760 | 3507605 | 158371.3 | 31120 | 22273.3 | 17411.6 | 12069.9 | 50854 | 17 |
| 3343SP | 35São Paulo | Braúna | 350770 | 3507704 | 5378.7 | 1056.9 | 756.5 | 591.3 | 409.9 | 2559 |  |
| 3344SP | 35São Paulo | Brejo Alegre | 350775 | 3507753 | 2732.5 | 536.9 | 384.3 | 300.4 | 208.3 | 1505 |  |
| 3345SP | 35São Paulo | Brodowski | 350780 | 3507803 | 23102 | 4539.5 | 3249.1 | 2539.9 | 1760.7 | 9227 |  |
| 3346SP | 35São Paulo | Brotas | 350790 | 3507902 | 23098.9 | 4538.9 | 3248.6 | 2539.5 | 1760.4 | 4527 |  |
| 3347SP | 35São Paulo | Buri | 350800 | 3508009 | 19341.1 | 3800.5 | 2720.1 | 2126.4 | 1474 | 3784 |  |
| 3348SP | 35São Paulo | Buritama | 350810 | 3508108 | 16365.7 | 3215.9 | 2301.7 | 1799.3 | 1247.3 | 9132 |  |
| 3349SP | 35São Paulo | Buritizal | 350820 | 3508207 | 4289.6 | 842.9 | 603.3 | 471.6 | 326.9 | 2234 |  |
| 3350SP | 35São Paulo | Cabrália Paulista | 350830 | 3508306 | 4353.3 | 855.4 | 612.2 | 478.6 | 331.8 | 898 |  |
| 3351SP | 35São Paulo | Cabreúva | 350840 | 3508405 | 45798 | 8999.3 | 6441 | 5035.1 | 3490.4 | 8452 |  |
| 3352SP | 35São Paulo | Caçapava | 350850 | 3508504 | 89969.7 | 17679 | 12653.3 | 9891.4 | 6856.8 | 26115 |  |
| 3353SP | 35São Paulo | Cachoeira Paulista | 350860 | 3508603 | 31876.5 | 6263.7 | 4483.1 | 3504.6 | 2429.4 | 6083 |  |
| 3354SP | 35São Paulo | Caconde | 350870 | 3508702 | 18847.7 | 3703.6 | 2650.7 | 2072.1 | 1436.4 | 7591 |  |
| 3355SP | 35São Paulo | Cafelândia | 350880 | 3508801 | 17294.9 | 3398.4 | 2432.4 | 1901.4 | 1318.1 | 2696 |  |
| 3356SP | 35São Paulo | Caiabu | 350890 | 3508900 | 4162.7 | 818 | 585.4 | 457.7 | 317.3 | 1468 |  |
| 3357SP | 35São Paulo | Caieiras | 350900 | 3509007 | 94342.3 | 18538.3 | 13268.3 | 10372.2 | 7190.1 | 20311 |  |
| 3358SP | 35São Paulo | Caiuá | 350910 | 3509106 | 5477.5 | 1076.3 | 770.4 | 602.2 | 417.5 | 2075 |  |
| 3359SP | 35São Paulo | Cajamar | 350920 | 3509205 | 70671.8 | 13887 | 9939.3 | 7769.8 | 5386.1 | 21264 |  |
| 3360SP | 35São Paulo | Cajati | 350925 | 3509254 | 28689.2 | 5637.4 | 4034.8 | 3154.1 | 2186.5 | 7027 |  |
| 3361SP | 35São Paulo | Cajobi | 350930 | 3509304 | 10216.5 | 2007.5 | 1436.8 | 1123.2 | 778.6 | 4863 |  |
| 3362SP | 35São Paulo | Cajuru | 350940 | 3509403 | 24892.8 | 4891.4 | 3500.9 | 2736.8 | 1897.2 | 8639 |  |
| 3363SP | 35São Paulo | Campina do  Monte Alegre | 350945 | 3509452 | 5829.9 | 1145.6 | 819.9 | 640.9 | 444.3 | 2252 |  |
| 3364SP | 35São Paulo | Campinas | 350950 | 3509502 | 1148489.7 | 225678.2 | 161523.6 | 126267 | 87529.6 | 258622 | 88 |
| 3365SP | 35São Paulo | Campo Limpo  Paulista | 350960 | 3509601 | 79709.7 | 15663 | 11210.4 | 8763.4 | 6074.9 | 13461 |  |
| 3366SP | 35São Paulo | Campos do  Jordão | 350970 | 3509700 | 50224.9 | 9869.2 | 7063.6 | 5521.8 | 3827.8 | 14223 |  |
| 3367SP | 35São Paulo | Campos Novos  Paulista | 350980 | 3509809 | 4778.9 | 939.1 | 672.1 | 525.4 | 364.2 | 1104 |  |
| 3368SP | 35São Paulo | Cananéia | 350990 | 3509908 | 12475.9 | 2451.5 | 1754.6 | 1371.6 | 950.8 | 3467 |  |
| 3369SP | 35São Paulo | Canas | 350995 | 3509957 | 4779.1 | 939.1 | 672.1 | 525.4 | 364.2 | 1134 |  |
| 3370SP | 35São Paulo | Cândido Mota | 351000 | 3510005 | 30793 | 6050.8 | 4330.7 | 3385.4 | 2346.8 | 10451 |  |
| 3371SP | 35São Paulo | Cândido  Rodrigues | 351010 | 3510104 | 2749.5 | 540.3 | 386.7 | 302.3 | 209.5 | 1045 |  |
| 3372SP | 35São Paulo | Canitar | 351015 | 3510153 | 4807.9 | 944.8 | 676.2 | 528.6 | 366.4 | 771 |  |
| 3373SP | 35São Paulo | Capão Bonito | 351020 | 3510203 | 47013.7 | 9238.2 | 6612 | 5168.8 | 3583 | 9964 |  |
| 3374SP | 35São Paulo | Capela do Alto | 351030 | 3510302 | 19184.9 | 3769.8 | 2698.2 | 2109.2 | 1462.1 | 3274 |  |
| 3375SP | 35São Paulo | Capivari | 351040 | 3510401 | 52394.6 | 10295.5 | 7368.8 | 5760.4 | 3993.1 | 10475 |  |
| 3376SP | 35São Paulo | Caraguatatuba | 351050 | 3510500 | 111504.5 | 21910.6 | 15682 | 12259 | 8498.1 | 44094 | 14 |
| 3377SP | 35São Paulo | Carapicuíba | 351060 | 3510609 | 387518.9 | 76147.5 | 54500.7 | 42604.5 | 29533.9 | 59390 | 18 |
| 3378SP | 35São Paulo | Cardoso | 351070 | 3510708 | 12149.4 | 2387.4 | 1708.7 | 1335.7 | 925.9 | 8185 |  |
| 3379SP | 35São Paulo | Casa Branca | 351080 | 3510807 | 29525.1 | 5801.7 | 4152.4 | 3246 | 2250.2 | 9070 |  |
| 3380SP | 35São Paulo | Cássia dos  Coqueiros | 351090 | 3510906 | 2603.2 | 511.5 | 366.1 | 286.2 | 198.4 | 1547 |  |
| 3381SP | 35São Paulo | Castilho | 351100 | 3511003 | 19578.4 | 3847.2 | 2753.5 | 2152.5 | 1492.1 | 9316 |  |
| 3382SP | 35São Paulo | Catanduva | 351110 | 3511102 | 118044.5 | 23195.7 | 16601.8 | 12978 | 8996.5 | 36415 | 10 |
| 3383SP | 35São Paulo | Catiguá | 351120 | 3511201 | 7507.8 | 1475.3 | 1055.9 | 825.4 | 572.2 | 1841 |  |
| 3384SP | 35São Paulo | Cedral | 351130 | 3511300 | 8639.2 | 1697.6 | 1215 | 949.8 | 658.4 | 3544 |  |
| 3385SP | 35São Paulo | Cerqueira César | 351140 | 3511409 | 18841.9 | 3702.4 | 2649.9 | 2071.5 | 1436 | 4310 |  |
| 3386SP | 35São Paulo | Cerquilho | 351150 | 3511508 | 44379.2 | 8720.5 | 6241.5 | 4879.1 | 3382.3 | 6546 |  |
| 3387SP | 35São Paulo | Cesário Lange | 351160 | 3511607 | 16907.7 | 3322.4 | 2377.9 | 1858.9 | 1288.6 | 4452 |  |
| 3388SP | 35São Paulo | Charqueada | 351170 | 3511706 | 16210.1 | 3185.3 | 2279.8 | 1782.2 | 1235.4 | 4120 |  |
| 3389SP | 35São Paulo | Clementina | 351190 | 3511904 | 7860.5 | 1544.6 | 1105.5 | 864.2 | 599.1 | 3274 |  |
| 3390SP | 35São Paulo | Colina | 351200 | 3512001 | 18035.6 | 3544 | 2536.5 | 1982.9 | 1374.5 | 9684 |  |
| 3391SP | 35São Paulo | Colômbia | 351210 | 3512100 | 6145.8 | 1207.6 | 864.3 | 675.7 | 468.4 | 4001 |  |
| 3392SP | 35São Paulo | Conchal | 351220 | 3512209 | 26777.3 | 5261.7 | 3766 | 2943.9 | 2040.8 | 8215 |  |
| 3393SP | 35São Paulo | Conchas | 351230 | 3512308 | 17186 | 3377 | 2417 | 1889.5 | 1309.8 | 3473 |  |
| 3394SP | 35São Paulo | Cordeirópolis | 351240 | 3512407 | 22892.1 | 4498.3 | 3219.5 | 2516.8 | 1744.7 | 6000 |  |
| 3395SP | 35São Paulo | Coroados | 351250 | 3512506 | 5670.3 | 1114.2 | 797.5 | 623.4 | 432.1 | 2444 |  |
| 3396SP | 35São Paulo | Coronel Macedo | 351260 | 3512605 | 4889.6 | 960.8 | 687.7 | 537.6 | 372.7 | 1417 |  |
| 3397SP | 35São Paulo | Corumbataí | 351270 | 3512704 | 3992.3 | 784.5 | 561.5 | 438.9 | 304.3 | 1287 |  |
| 3398SP | 35São Paulo | Cosmópolis | 351280 | 3512803 | 65692.4 | 12908.6 | 9239 | 7222.3 | 5006.6 | 6825 |  |
| 3399SP | 35São Paulo | Cosmorama | 351290 | 3512902 | 7317.3 | 1437.8 | 1029.1 | 804.5 | 557.7 | 5052 |  |
| 3400SP | 35São Paulo | Cotia | 351300 | 3513009 | 225647.6 | 44339.8 | 31735.1 | 24808.1 | 17197.2 | 55757 | 19 |
| 3401SP | 35São Paulo | Cravinhos | 351310 | 3513108 | 33663 | 6614.8 | 4734.4 | 3701 | 2565.6 | 4284 |  |
| 3402SP | 35São Paulo | Cristais Paulista | 351320 | 3513207 | 8145.8 | 1600.6 | 1145.6 | 895.6 | 620.8 | 2256 |  |
| 3403SP |  | Cruzália | 351330 | 3513306 | 2196.8 | 431.7 | 309 | 241.5 | 167.4 | 1600 |  |
| 3404SP |  | Cruzeiro | 351340 | 3513405 | 80142.7 | 15748 | 11271.3 | 8811 | 6107.9 | 9884 |  |
| 3405SP |  | Cubatão | 351350 | 3513504 | 125392.1 | 24639.5 | 17635.1 | 13785.8 | 9556.5 | 36652 | 12 |
| 3406SP |  | Cunha | 351360 | 3513603 | 21894.6 | 4302.3 | 3079.3 | 2407.1 | 1668.6 | 4355 |  |
| 3407SP |  | Descalvado | 351370 | 3513702 | 32576.9 | 6401.4 | 4581.6 | 3581.6 | 2482.8 | 9419 |  |
| 3408SP |  | Diadema | 351380 | 3513801 | 407227 | 80020.1 | 57272.4 | 44771.2 | 31035.9 | 99789 | 33 |
| 3409SP |  | Dirce Reis | 351385 | 3513850 | 1752.3 | 344.3 | 246.4 | 192.7 | 133.5 | 1434 |  |
| 3410SP |  | Divinolândia | 351390 | 3513900 | 11325.8 | 2225.5 | 1592.9 | 1245.2 | 863.2 | 5526 |  |
| 3411SP |  | Dobrada | 351400 | 3514007 | 8475.5 | 1665.4 | 1192 | 931.8 | 645.9 | 1916 |  |
| 3412SP |  | Dois Córregos | 351410 | 3514106 | 26178.5 | 5144.1 | 3681.7 | 2878.1 | 1995.1 | 3332 |  |
| 3413SP |  | Dolcinópolis | 351420 | 3514205 | 2122 | 417 | 298.4 | 233.3 | 161.7 | 1480 |  |
| 3414SP |  | Dourado | 351430 | 3514304 | 8805.2 | 1730.2 | 1238.4 | 968.1 | 671.1 | 2096 |  |
| 3415SP |  | Dracena | 351440 | 3514403 | 45293.7 | 8900.2 | 6370.1 | 4979.7 | 3452 | 23994 |  |
| 3416SP |  | Duartina | 351450 | 3514502 | 12444 | 2445.2 | 1750.1 | 1368.1 | 948.4 | 3822 |  |

2

2

3

8

1

4

1

7

1

1

3

3

1

1

3

1

1

3

2

7

1

2

7

7

1

2

3

4

4

3

3

1

3

2

3

3

1

1

1

1

1

1

4

2

3

1

2

2

1

1

3

1

3

1

7

| 3417SP |  | Dumont | 351460 | 3514601 | 9029.7 | 1774.3 | 1269.9 | 992.7 | 688.2 | 1092 |  |
| --- | --- | --- | --- | --- | --- | --- | --- | --- | --- | --- | --- |
| 3418SP |  | Echaporã | 351470 | 3514700 | 6266.7 | 1231.4 | 881.3 | 689 | 477.6 | 1866 |  |
| 3419SP |  | Eldorado | 351480 | 3514809 | 15166.6 | 2980.2 | 2133 | 1667.4 | 1155.9 | 2973 |  |
| 3420SP |  | Elias Fausto | 351490 | 3514908 | 16853.8 | 3311.8 | 2370.3 | 1852.9 | 1284.5 | 3994 |  |
| 3421SP |  | Elisiário | 351492 | 3514924 | 3398.6 | 667.8 | 478 | 373.6 | 259 | 1312 |  |
| 3422SP |  | Embaúba | 351495 | 3514957 | 2457.1 | 482.8 | 345.6 | 270.1 | 187.3 | 971 |  |
| 3423SP |  | Embu-Guaçu | 351510 | 3515103 | 66430 | 13053.5 | 9342.7 | 7303.4 | 5062.8 | 8843 |  |
| 3424SP |  | Emilianópolis | 351512 | 3515129 | 3137.4 | 616.5 | 441.2 | 344.9 | 239.1 | 1509 |  |
| 3425SP |  | Engenheiro  Coelho | 351515 | 3515152 | 18251.7 | 3586.5 | 2566.9 | 2006.6 | 1391 | 3454 |  |
| 3426SP |  | Espírito Santo do  Pinhal | 351518 | 3515186 | 43403.3 | 8528.7 | 6104.2 | 4771.8 | 3307.9 | 6500 |  |
| 3427SP | 35São Paulo | Espírito Santo do  Turvo | 351519 | 3515194 | 4557.1 | 895.5 | 640.9 | 501 | 347.3 | 2227 |  |
| 3428SP | 35São Paulo | Estrela d'Oeste | 351520 | 3515202 | 8376 | 1645.9 | 1178 | 920.9 | 638.4 | 4457 |  |
| 3429SP | 35São Paulo | Estrela do Norte | 351530 | 3515301 | 2731.3 | 536.7 | 384.1 | 300.3 | 208.2 | 795 |  |
| 3430SP | 35São Paulo | Euclides da Cunha  Paulista | 351535 | 3515350 | 9561.8 | 1878.9 | 1344.8 | 1051.2 | 728.7 | 5296 |  |
| 3431SP | 35São Paulo | Fartura | 351540 | 3515400 | 15786.4 | 3102 | 2220.2 | 1735.6 | 1203.1 | 5354 |  |
| 3432SP | 35São Paulo | Fernandópolis | 351550 | 3515509 | 67329 | 13230.1 | 9469.2 | 7402.3 | 5131.3 | 33209 | 13 |
| 3433SP | 35São Paulo | Fernando Prestes | 351560 | 3515608 | 5697.7 | 1119.6 | 801.3 | 626.4 | 434.2 | 1574 |  |
| 3434SP | 35São Paulo | Fernão | 351565 | 3515657 | 1648.3 | 323.9 | 231.8 | 181.2 | 125.6 | 1188 |  |
| 3435SP | 35São Paulo | Ferraz de  Vasconcelos | 351570 | 3515707 | 182033.9 | 35769.7 | 25601.2 | 20013.1 | 13873.3 | 25226 |  |
| 3436SP | 35São Paulo | Flora Rica | 351580 | 3515806 | 1629.4 | 320.2 | 229.2 | 179.1 | 124.2 | 819 |  |
| 3437SP | 35São Paulo | Floreal | 351590 | 3515905 | 2986.8 | 586.9 | 420.1 | 328.4 | 227.6 | 1866 |  |
| 3438SP | 35São Paulo | Flórida Paulista | 351600 | 3516002 | 13805.1 | 2712.7 | 1941.5 | 1517.8 | 1052.1 | 4444 |  |
| 3439SP | 35São Paulo | Florínia | 351610 | 3516101 | 2779.4 | 546.2 | 390.9 | 305.6 | 211.8 | 1085 |  |
| 3440SP | 35São Paulo | Franca | 351620 | 3516200 | 337679.4 | 66354 | 47491.2 | 37125.1 | 25735.5 | 96876 | 36 |
| 3441SP | 35São Paulo | Francisco Morato | 351630 | 3516309 | 165900 | 32599.3 | 23332.2 | 18239.3 | 12643.7 | 37182 | 13 |
| 3442SP | 35São Paulo | Franco da Rocha | 351640 | 3516408 | 143563 | 28210.1 | 20190.7 | 15783.6 | 10941.3 | 35242 | 12 |
| 3443SP | 35São Paulo | Gabriel Monteiro | 351650 | 3516507 | 2762.5 | 542.8 | 388.5 | 303.7 | 210.5 | 2166 |  |
| 3444SP | 35São Paulo | Gália | 351660 | 3516606 | 6848.3 | 1345.7 | 963.1 | 752.9 | 521.9 | 3222 |  |
| 3445SP | 35São Paulo | Garça | 351670 | 3516705 | 44075 | 8660.7 | 6198.7 | 4845.7 | 3359.1 | 10413 |  |
| 3446SP | 35São Paulo | Gastão Vidigal | 351680 | 3516804 | 4519.8 | 888.1 | 635.7 | 496.9 | 344.5 | 1169 |  |
| 3447SP | 35São Paulo | Gavião Peixoto | 351685 | 3516853 | 4631.3 | 910.1 | 651.3 | 509.2 | 353 | 1272 |  |
| 3448SP | 35São Paulo | General Salgado | 351690 | 3516903 | 10852.1 | 2132.4 | 1526.2 | 1193.1 | 827.1 | 5112 |  |
| 3449SP | 35São Paulo | Getulina | 351700 | 3517000 | 11159.6 | 2192.9 | 1569.5 | 1226.9 | 850.5 | 3014 |  |
| 3450SP | 35São Paulo | Glicério | 351710 | 3517109 | 4720.7 | 927.6 | 663.9 | 519 | 359.8 | 2639 |  |
| 3451SP | 35São Paulo | Guaiçara | 351720 | 3517208 | 11470 | 2253.9 | 1613.1 | 1261 | 874.2 | 2137 |  |
| 3452SP | 35São Paulo | Guaimbê | 351730 | 3517307 | 5631.3 | 1106.6 | 792 | 619.1 | 429.2 | 2176 |  |
| 3453SP | 35São Paulo | Guaíra | 351740 | 3517406 | 39320.5 | 7726.5 | 5530 | 4323 | 2996.7 | 17267 |  |
| 3454SP | 35São Paulo | Guapiaçu | 351750 | 3517505 | 19719.9 | 3875 | 2773.4 | 2168 | 1502.9 | 6302 |  |
| 3455SP | 35São Paulo | Guapiara | 351760 | 3517604 | 17745.9 | 3487.1 | 2495.8 | 1951 | 1352.5 | 3203 |  |
| 3456SP | 35São Paulo | Guará | 351770 | 3517703 | 20668.3 | 4061.3 | 2906.8 | 2272.3 | 1575.2 | 3343 |  |
| 3457SP | 35São Paulo | Guaraçaí | 351780 | 3517802 | 8450.8 | 1660.6 | 1188.5 | 929.1 | 644.1 | 6270 |  |
| 3458SP | 35São Paulo | Guaraci | 351790 | 3517901 | 10634.3 | 2089.6 | 1495.6 | 1169.2 | 810.5 | 4748 |  |
| 3459SP | 35São Paulo | Guarani d'Oeste | 351800 | 3518008 | 2000.3 | 393.1 | 281.3 | 219.9 | 152.4 | 1560 |  |
| 3460SP | 35São Paulo | Guarantã | 351810 | 3518107 | 6580.3 | 1293 | 925.5 | 723.4 | 501.5 | 1984 |  |
| 3461SP | 35São Paulo | Guararapes | 351820 | 3518206 | 31961.6 | 6280.5 | 4495.1 | 3513.9 | 2435.9 | 12579 |  |
| 3462SP | 35São Paulo | Guararema | 351830 | 3518305 | 27935.9 | 5489.4 | 3928.9 | 3071.3 | 2129.1 | 11832 |  |
| 3463SP | 35São Paulo | Guaratinguetá | 351840 | 3518404 | 117616 | 23111.5 | 16541.5 | 12930.9 | 8963.8 | 25844 |  |
| 3464SP | 35São Paulo | Guareí | 351850 | 3518503 | 16564.6 | 3254.9 | 2329.6 | 1821.1 | 1262.4 | 3838 |  |
| 3465SP | 35São Paulo | Guariba | 351860 | 3518602 | 37973.1 | 7461.7 | 5340.5 | 4174.8 | 2894 | 10110 |  |
| 3466SP | 35São Paulo | Guarujá | 351870 | 3518701 | 307261.6 | 60376.9 | 43213.3 | 33780.9 | 23417.3 | 82848 | 30 |
| 3467SP | 35São Paulo | Guarulhos | 351880 | 3518800 | 1306744.1 | 256775.2 | 183780.5 | 143665.7 | 99590.6 | 311330 | 111 |
| 3468SP | 35São Paulo | Guatapará | 351885 | 3518859 | 7351.5 | 1444.6 | 1033.9 | 808.2 | 560.3 | 1541 |  |
| 3469SP | 35São Paulo | Guzolândia | 351890 | 3518909 | 5037.1 | 989.8 | 708.4 | 553.8 | 383.9 | 3162 |  |
| 3470SP | 35São Paulo | Herculândia | 351900 | 3519006 | 9162.1 | 1800.4 | 1288.6 | 1007.3 | 698.3 | 4050 |  |
| 3471SP | 35São Paulo | Holambra | 351905 | 3519055 | 13117.6 | 2577.6 | 1844.9 | 1442.2 | 999.7 | 2709 |  |
| 3472SP | 35São Paulo | Hortolândia | 351907 | 3519071 | 212413.9 | 41739.3 | 29873.9 | 23353.2 | 16188.7 | 46254 | 12 |
| 3473SP | 35São Paulo | Iacanga | 351910 | 3519105 | 10901.7 | 2142.2 | 1533.2 | 1198.6 | 830.8 | 3067 |  |
| 3474SP | 35São Paulo | Iacri | 351920 | 3519204 | 6425.1 | 1262.5 | 903.6 | 706.4 | 489.7 | 3157 |  |
| 3475SP | 35São Paulo | Iaras | 351925 | 3519253 | 7790 | 1530.7 | 1095.6 | 856.4 | 593.7 | 1046 |  |
| 3476SP | 35São Paulo | Ibaté | 351930 | 3519303 | 33063.9 | 6497.1 | 4650.1 | 3635.1 | 2519.9 | 6370 |  |
| 3477SP | 35São Paulo | Ibirá | 351940 | 3519402 | 11696.7 | 2298.4 | 1645 | 1286 | 891.4 | 4635 |  |
| 3478SP | 35São Paulo | Ibirarema | 351950 | 3519501 | 7269.3 | 1428.4 | 1022.4 | 799.2 | 554 | 2058 |  |
| 3479SP | 35São Paulo | Ibitinga | 351960 | 3519600 | 56862.9 | 11173.6 | 7997.2 | 6251.6 | 4333.7 | 12868 |  |
| 3480SP | 35São Paulo | Ibiúna | 351970 | 3519709 | 75443.6 | 14824.7 | 10610.4 | 8294.4 | 5749.8 | 8125 |  |
| 3481SP | 35São Paulo | Icém | 351980 | 3519808 | 7894.7 | 1551.3 | 1110.3 | 868 | 601.7 | 2814 |  |
| 3482SP | 35São Paulo | Iepê | 351990 | 3519907 | 7942.9 | 1560.8 | 1117.1 | 873.3 | 605.4 | 4969 |  |
| 3483SP | 35São Paulo | Igaraçu do Tietê | 352000 | 3520004 | 24177.5 | 4750.9 | 3400.3 | 2658.1 | 1842.6 | 4095 |  |
| 3484SP | 35São Paulo | Igarapava | 352010 | 3520103 | 29361.3 | 5769.5 | 4129.4 | 3228 | 2237.7 | 6767 |  |
| 3485SP | 35São Paulo | Igaratá | 352020 | 3520202 | 9237.4 | 1815.1 | 1299.1 | 1015.6 | 704 | 4391 |  |
| 3486SP | 35São Paulo | Iguape | 352030 | 3520301 | 30036 | 5902.1 | 4224.3 | 3302.2 | 2289.1 | 5443 |  |
| 3487SP | 35São Paulo | Ilhabela | 352040 | 3520400 | 31648.3 | 6218.9 | 4451 | 3479.5 | 2412 | 6439 |  |
| 3488SP | 35São Paulo | Ilha Comprida | 352042 | 3520426 | 10116.8 | 1988 | 1422.8 | 1112.3 | 771 | 3648 |  |
| 3489SP | 35São Paulo | Ilha Solteira | 352044 | 3520442 | 26041.1 | 5117.1 | 3662.4 | 2863 | 1984.7 | 15591 |  |
| 3490SP | 35São Paulo | Indaiatuba | 352050 | 3520509 | 227046.7 | 44614.7 | 31931.8 | 24961.9 | 17303.9 | 58938 | 20 |
| 3491SP | 35São Paulo | Indiana | 352060 | 3520608 | 4893.3 | 961.5 | 688.2 | 538 | 372.9 | 2973 |  |
| 3492SP | 35São Paulo | Indiaporã | 352070 | 3520707 | 3932.5 | 772.7 | 553.1 | 432.3 | 299.7 | 3697 |  |
| 3493SP | 35São Paulo | Inúbia Paulista | 352080 | 3520806 | 3831.7 | 752.9 | 538.9 | 421.3 | 292 | 2054 |  |
| 3494SP | 35São Paulo | Ipaussu | 352090 | 3520905 | 14396.7 | 2829 | 2024.8 | 1582.8 | 1097.2 | 5429 |  |
| 3495SP | 35São Paulo | Iperó | 352100 | 3521002 | 32731.4 | 6431.7 | 4603.3 | 3598.5 | 2494.6 | 5402 |  |
| 3496SP | 35São Paulo | Ipeúna | 352110 | 3521101 | 6793.2 | 1334.9 | 955.4 | 746.9 | 517.7 | 1955 |  |
| 3497SP | 35São Paulo | Ipiguá | 352115 | 3521150 | 4941.3 | 971 | 694.9 | 543.3 | 376.6 | 2222 |  |
| 3498SP | 35São Paulo | Iporanga | 352120 | 3521200 | 4296.2 | 844.2 | 604.2 | 472.3 | 327.4 | 968 |  |
| 3499SP | 35São Paulo | Ipuã | 352130 | 3521309 | 15339.4 | 3014.2 | 2157.3 | 1686.4 | 1169.1 | 4429 |  |
| 3500SP | 35São Paulo | Iracemápolis | 352140 | 3521408 | 22192.8 | 4360.9 | 3121.2 | 2439.9 | 1691.4 | 4853 |  |
| 3501SP | 35São Paulo | Irapuã | 352150 | 3521507 | 7676.2 | 1508.4 | 1079.6 | 843.9 | 585 | 3046 |  |
| 3502SP | 35São Paulo | Irapuru | 352160 | 3521606 | 8092.8 | 1590.2 | 1138.2 | 889.7 | 616.8 | 2577 |  |
| 3503SP | 35São Paulo | Itaberá | 352170 | 3521705 | 17860.9 | 3509.7 | 2512 | 1963.7 | 1361.2 | 4277 |  |
| 3504SP | 35São Paulo | Itaí | 352180 | 3521804 | 25686.9 | 5047.5 | 3612.6 | 2824.1 | 1957.7 | 3975 |  |
| 3505SP |  | Itajobi | 352190 | 3521903 | 15011.1 | 2949.7 | 2111.2 | 1650.3 | 1144 | 7136 |  |
| 3506SP |  | Itaju | 352200 | 3522000 | 3551.9 | 697.9 | 499.5 | 390.5 | 270.7 | 770 |  |
| 3507SP |  | Itanhaém | 352210 | 3522109 | 94787.7 | 18625.8 | 13330.9 | 10421.1 | 7224 | 19648 |  |
| 3508SP |  | Itaóca | 352215 | 3522158 | 3302.2 | 648.9 | 464.4 | 363 | 251.7 | 985 |  |
| 3509SP |  | Itapecerica da  Serra | 352220 | 3522208 | 164838.6 | 32390.8 | 23182.9 | 18122.6 | 12562.8 | 37995 | 13 |
| 3510SP |  | Itapetininga | 352230 | 3522307 | 154844.2 | 30426.9 | 21777.3 | 17023.8 | 11801.1 | 40038 | 13 |
| 3511SP |  | Itapeva | 352240 | 3522406 | 91611.5 | 18001.7 | 12884.2 | 10071.9 | 6982 | 19837 |  |
| 3512SP |  | Itapevi | 352250 | 3522505 | 219974 | 43224.9 | 30937.1 | 24184.3 | 16764.8 | 56261 | 22 |
| 3513SP |  | Itapira | 352260 | 3522604 | 72064.2 | 14160.6 | 10135.1 | 7922.9 | 5492.2 | 17635 |  |
| 3514SP |  | Itapirapuã Paulista | 352265 | 3522653 | 4083.5 | 802.4 | 574.3 | 448.9 | 311.2 | 558 |  |
| 3515SP |  | Itápolis | 352270 | 3522703 | 41839.3 | 8221.4 | 5884.3 | 4599.9 | 3188.7 | 8226 |  |
| 3516SP |  | Itaporanga | 352280 | 3522802 | 14954 | 2938.5 | 2103.1 | 1644.1 | 1139.7 | 3514 |  |
| 3517SP |  | Itapuí | 352290 | 3522901 | 13137.7 | 2581.6 | 1847.7 | 1444.4 | 1001.3 | 1878 |  |
| 3518SP |  | Itapura | 352300 | 3523008 | 4653.8 | 914.5 | 654.5 | 511.6 | 354.7 | 2564 |  |
| 3519SP |  | Itaquaquecetuba | 352310 | 3523107 | 347730.1 | 68329 | 48904.8 | 38230.1 | 26501.5 | 40940 | 14 |
| 3520SP |  | Itararé | 352320 | 3523206 | 49548.8 | 9736.3 | 6968.5 | 5447.5 | 3776.3 | 10590 |  |
| 3521SP |  | Itariri | 352330 | 3523305 | 16532.1 | 3248.6 | 2325.1 | 1817.6 | 1260 | 3925 |  |
| 3522SP |  | Itatiba | 352340 | 3523404 | 111520.5 | 21913.8 | 15684.2 | 12260.8 | 8499.3 | 46494 | 14 |

1

2

1

1

1

1

1

9

1

2

1

1

7

2

1

2

2

3

4

9

1

3

1

1

1

2

1

3

2

1

1

1

2

1

1

2

5

1

1

1

1

1

1

1

1

2

5

6

5

2

3

1

| 3523SP |  | Itatinga | 352350 | 3523503 | 19457.8 | 3823.5 | 2736.5 | 2139.2 | 1482.9 | 3755 |  |
| --- | --- | --- | --- | --- | --- | --- | --- | --- | --- | --- | --- |
| 3524SP |  | Itirapina | 352360 | 3523602 | 16903.6 | 3321.6 | 2377.3 | 1858.4 | 1288.3 | 2999 |  |
| 3525SP |  | Itirapuã | 352370 | 3523701 | 6240.7 | 1226.3 | 877.7 | 686.1 | 475.6 | 1798 |  |
| 3526SP |  | Itobi | 352380 | 3523800 | 7748 | 1522.5 | 1089.7 | 851.8 | 590.5 | 2916 |  |
| 3527SP |  | Itu | 352390 | 3523909 | 164821.4 | 32387.4 | 23180.5 | 18120.8 | 12561.5 | 33358 | 12 |
| 3528SP |  | Itupeva | 352400 | 3524006 | 53020 | 10418.4 | 7456.7 | 5829.1 | 4040.8 | 14612 |  |
| 3529SP |  | Ituverava | 352410 | 3524105 | 40500.2 | 7958.3 | 5695.9 | 4452.7 | 3086.6 | 16290 |  |
| 3530SP |  | Jaborandi | 352420 | 3524204 | 6806.4 | 1337.5 | 957.3 | 748.3 | 518.7 | 3556 |  |
| 3531SP |  | Jaboticabal | 352430 | 3524303 | 74913.9 | 14720.6 | 10535.9 | 8236.2 | 5709.4 | 12807 |  |
| 3532SP | 35São Paulo | Jacareí | 352440 | 3524402 | 223619.9 | 43941.3 | 31449.9 | 24585.2 | 17042.7 | 81825 | 28 |
| 3533SP | 35São Paulo | Jaci | 352450 | 3524501 | 6374 | 1252.5 | 896.4 | 700.8 | 485.8 | 1878 |  |
| 3534SP | 35São Paulo | Jacupiranga | 352460 | 3524600 | 17662.5 | 3470.7 | 2484.1 | 1941.8 | 1346.1 | 4241 |  |
| 3535SP | 35São Paulo | Jaguariúna | 352470 | 3524709 | 50938.9 | 10009.5 | 7164 | 5600.3 | 3882.2 | 27611 | 10 |
| 3536SP | 35São Paulo | Jales | 352480 | 3524808 | 48394 | 9509.4 | 6806.1 | 5320.5 | 3688.2 | 26619 |  |
| 3537SP | 35São Paulo | Jambeiro | 352490 | 3524907 | 5988.9 | 1176.8 | 842.3 | 658.4 | 456.4 | 2023 |  |
| 3538SP | 35São Paulo | Jandira | 352500 | 3525003 | 117121.6 | 23014.4 | 16472 | 12876.6 | 8926.2 | 18186 |  |
| 3539SP | 35São Paulo | Jardinópolis | 352510 | 3525102 | 41164.5 | 8088.8 | 5789.4 | 4525.7 | 3137.3 | 7228 |  |
| 3540SP | 35São Paulo | Jarinu | 352520 | 3525201 | 26989 | 5303.3 | 3795.7 | 2967.2 | 2056.9 | 7569 |  |
| 3541SP | 35São Paulo | Jaú | 352530 | 3525300 | 141249.5 | 27755.5 | 19865.3 | 15529.2 | 10765 | 23748 |  |
| 3542SP | 35São Paulo | Jeriquara | 352540 | 3525409 | 3186 | 626 | 448.1 | 350.3 | 242.8 | 1202 |  |
| 3543SP | 35São Paulo | Joanópolis | 352550 | 3525508 | 12554.3 | 2466.9 | 1765.6 | 1380.2 | 956.8 | 3771 |  |
| 3544SP | 35São Paulo | João Ramalho | 352560 | 3525607 | 4361.5 | 857 | 613.4 | 479.5 | 332.4 | 2123 |  |
| 3545SP | 35São Paulo | José Bonifácio | 352570 | 3525706 | 35053.3 | 6888 | 4929.9 | 3853.8 | 2671.5 | 9103 |  |
| 3546SP | 35São Paulo | Júlio Mesquita | 352580 | 3525805 | 4631 | 910 | 651.3 | 509.1 | 352.9 | 906 |  |
| 3547SP | 35São Paulo | Jumirim | 352585 | 3525854 | 3091.6 | 607.5 | 434.8 | 339.9 | 235.6 | 472 |  |
| 3548SP | 35São Paulo | Jundiaí | 352590 | 3525904 | 396381.2 | 77888.9 | 55747.1 | 43578.8 | 30209.3 | 161369 | 54 |
| 3549SP | 35São Paulo | Junqueirópolis | 352600 | 3526001 | 19808.6 | 3892.4 | 2785.9 | 2177.8 | 1509.7 | 10441 |  |
| 3550SP | 35São Paulo | Juquiá | 352610 | 3526100 | 19197.6 | 3772.3 | 2700 | 2110.6 | 1463.1 | 4554 |  |
| 3551SP | 35São Paulo | Juquitiba | 352620 | 3526209 | 30259.7 | 5946 | 4255.7 | 3326.8 | 2306.2 | 6077 |  |
| 3552SP | 35São Paulo | Lagoinha | 352630 | 3526308 | 4906.8 | 964.2 | 690.1 | 539.5 | 374 | 2800 |  |
| 3553SP | 35São Paulo | Laranjal Paulista | 352640 | 3526407 | 27010.6 | 5307.6 | 3798.8 | 2969.6 | 2058.6 | 3815 |  |
| 3554SP | 35São Paulo | Lavínia | 352650 | 3526506 | 10372.9 | 2038.3 | 1458.8 | 1140.4 | 790.5 | 3149 |  |
| 3555SP | 35São Paulo | Lavrinhas | 352660 | 3526605 | 6962.6 | 1368.2 | 979.2 | 765.5 | 530.6 | 1045 |  |
| 3556SP | 35São Paulo | Leme | 352670 | 3526704 | 98040.9 | 19265 | 13788.5 | 10778.8 | 7472 | 26090 |  |
| 3557SP | 35São Paulo | Lençóis Paulista | 352680 | 3526803 | 65262.3 | 12824 | 9178.5 | 7175.1 | 4973.8 | 17275 |  |
| 3558SP | 35São Paulo | Limeira | 352690 | 3526902 | 292593.5 | 57494.6 | 41150.3 | 32168.2 | 22299.4 | 71694 | 24 |
| 3559SP | 35São Paulo | Lindóia | 352700 | 3527009 | 7369.4 | 1448.1 | 1036.4 | 810.2 | 561.6 | 2982 |  |
| 3560SP | 35São Paulo | Lins | 352710 | 3527108 | 75147.2 | 14766.4 | 10568.7 | 8261.8 | 5727.2 | 14007 |  |
| 3561SP | 35São Paulo | Lorena | 352720 | 3527207 | 86147.2 | 16927.9 | 12115.7 | 9471.2 | 6565.5 | 19735 |  |
| 3562SP | 35São Paulo | Lourdes | 352725 | 3527256 | 2222.3 | 436.7 | 312.5 | 244.3 | 169.4 | 1748 |  |
| 3563SP | 35São Paulo | Louveira | 352730 | 3527306 | 43020.4 | 8453.5 | 6050.4 | 4729.7 | 3278.7 | 8787 |  |
| 3564SP | 35São Paulo | Lucélia | 352740 | 3527405 | 20931.8 | 4113.1 | 2943.8 | 2301.3 | 1595.3 | 7891 |  |
| 3565SP | 35São Paulo | Lucianópolis | 352750 | 3527504 | 2336.5 | 459.1 | 328.6 | 256.9 | 178.1 | 938 |  |
| 3566SP | 35São Paulo | Luís Antônio | 352760 | 3527603 | 13119 | 2577.9 | 1845.1 | 1442.3 | 999.8 | 1279 |  |
| 3567SP | 35São Paulo | Luiziânia | 352770 | 3527702 | 5432.6 | 1067.5 | 764 | 597.3 | 414 | 2437 |  |
| 3568SP | 35São Paulo | Lupércio | 352780 | 3527801 | 4498.4 | 883.9 | 632.7 | 494.6 | 342.8 | 2201 |  |
| 3569SP | 35São Paulo | Lutécia | 352790 | 3527900 | 2705.9 | 531.7 | 380.6 | 297.5 | 206.2 | 1169 |  |
| 3570SP | 35São Paulo | Macatuba | 352800 | 3528007 | 16822.4 | 3305.6 | 2365.9 | 1849.5 | 1282.1 | 3267 |  |
| 3571SP | 35São Paulo | Macaubal | 352810 | 3528106 | 7942.8 | 1560.8 | 1117.1 | 873.2 | 605.3 | 3856 |  |
| 3572SP | 35São Paulo | Macedônia | 352820 | 3528205 | 3710.3 | 729.1 | 521.8 | 407.9 | 282.8 | 2674 |  |
| 3573SP | 35São Paulo | Magda | 352830 | 3528304 | 3188.1 | 626.5 | 448.4 | 350.5 | 243 | 1682 |  |
| 3574SP | 35São Paulo | Mairinque | 352840 | 3528403 | 45445.2 | 8930 | 6391.4 | 4996.3 | 3463.5 | 7770 |  |
| 3575SP | 35São Paulo | Mairiporã | 352850 | 3528502 | 90758.4 | 17834 | 12764.3 | 9978.1 | 6917 | 23293 |  |
| 3576SP | 35São Paulo | Manduri | 352860 | 3528601 | 9471.8 | 1861.2 | 1332.1 | 1041.3 | 721.9 | 2265 |  |
| 3577SP | 35São Paulo | Marabá Paulista | 352870 | 3528700 | 5346.6 | 1050.6 | 751.9 | 587.8 | 407.5 | 1538 |  |
| 3578SP | 35São Paulo | Maracaí | 352880 | 3528809 | 13760.2 | 2703.9 | 1935.2 | 1512.8 | 1048.7 | 3882 |  |
| 3579SP | 35São Paulo | Marapoama | 352885 | 3528858 | 2843.7 | 558.8 | 399.9 | 312.6 | 216.7 | 1589 |  |
| 3580SP | 35São Paulo | Mariápolis | 352890 | 3528908 | 4028.3 | 791.6 | 566.5 | 442.9 | 307 | 1986 |  |
| 3581SP | 35São Paulo | Marília | 352900 | 3529005 | 229048.2 | 45008 | 32213.3 | 25182 | 17456.4 | 61726 | 18 |
| 3582SP | 35São Paulo | Marinópolis | 352910 | 3529104 | 2130 | 418.5 | 299.6 | 234.2 | 162.3 | 1351 |  |
| 3583SP | 35São Paulo | Martinópolis | 352920 | 3529203 | 25484.2 | 5007.6 | 3584.1 | 2801.8 | 1942.2 | 10637 |  |
| 3584SP | 35São Paulo | Matão | 352930 | 3529302 | 80451.8 | 15808.8 | 11314.7 | 8845 | 6131.5 | 22261 |  |
| 3585SP | 35São Paulo | Mauá | 352940 | 3529401 | 447037.6 | 87842.9 | 62871.4 | 49148.1 | 34070 | 66795 | 20 |
| 3586SP | 35São Paulo | Mendonça | 352950 | 3529500 | 5082.3 | 998.7 | 714.8 | 558.8 | 387.3 | 3193 |  |
| 3587SP | 35São Paulo | Meridiano | 352960 | 3529609 | 3877.4 | 761.9 | 545.3 | 426.3 | 295.5 | 3151 |  |
| 3588SP | 35São Paulo | Mesópolis | 352965 | 3529658 | 1912 | 375.7 | 268.9 | 210.2 | 145.7 | 1419 |  |
| 3589SP | 35São Paulo | Miguelópolis | 352970 | 3529708 | 21462.5 | 4217.4 | 3018.5 | 2359.6 | 1635.7 | 4965 |  |
| 3590SP | 35São Paulo | Mineiros do Tietê | 352980 | 3529807 | 12550.8 | 2466.2 | 1765.1 | 1379.9 | 956.5 | 2555 |  |
| 3591SP | 35São Paulo | Miracatu | 352990 | 3529906 | 20374.7 | 4003.6 | 2865.5 | 2240 | 1552.8 | 5083 |  |
| 3592SP | 35São Paulo | Mira Estrela | 353000 | 3530003 | 2969.8 | 583.6 | 417.7 | 326.5 | 226.3 | 2328 |  |
| 3593SP | 35São Paulo | Mirandópolis | 353010 | 3530102 | 28698.3 | 5639.2 | 4036.1 | 3155.1 | 2187.2 | 15563 |  |
| 3594SP | 35São Paulo | Mirante do  Paranapanema | 353020 | 3530201 | 17769.3 | 3491.7 | 2499.1 | 1953.6 | 1354.2 | 4693 |  |
| 3595SP | 35São Paulo | Mirassol | 353030 | 3530300 | 57100.9 | 11220.3 | 8030.7 | 6277.8 | 4351.8 | 13163 |  |
| 3596SP | 35São Paulo | Mirassolândia | 353040 | 3530409 | 4604.1 | 904.7 | 647.5 | 506.2 | 350.9 | 2466 |  |
| 3597SP | 35São Paulo | Mococa | 353050 | 3530508 | 68068 | 13375.4 | 9573.1 | 7483.5 | 5187.7 | 23360 |  |
| 3598SP | 35São Paulo | Mogi das Cruzes | 353060 | 3530607 | 418565.4 | 82248.1 | 58867 | 46017.8 | 31900 | 128223 | 45 |
| 3599SP | 35São Paulo | Mogi Guaçu | 353070 | 3530706 | 145333.4 | 28558 | 20439.7 | 15978.2 | 11076.3 | 45040 | 14 |
| 3600SP | 35São Paulo | Moji Mirim | 353080 | 3530805 | 90392.9 | 17762.2 | 12712.9 | 9938 | 6889.1 | 15741 |  |
| 3601SP | 35São Paulo | Mombuca | 353090 | 3530904 | 3401 | 668.3 | 478.3 | 373.9 | 259.2 | 848 |  |
| 3602SP | 35São Paulo | Monções | 353100 | 3531001 | 2209.7 | 434.2 | 310.8 | 242.9 | 168.4 | 1824 |  |
| 3603SP | 35São Paulo | Mongaguá | 353110 | 3531100 | 51621.6 | 10143.6 | 7260.1 | 5675.4 | 3934.2 | 15281 |  |
| 3604SP | 35São Paulo | Monte Alegre do  Sul | 353120 | 3531209 | 7631.7 | 1499.6 | 1073.3 | 839 | 581.6 | 2644 |  |
| 3605SP | 35São Paulo | Monte Alto | 353130 | 3531308 | 48857.9 | 9600.6 | 6871.4 | 5371.5 | 3723.6 | 8706 |  |
| 3606SP | 35São Paulo | Monte Aprazível | 353140 | 3531407 | 23513 | 4620.3 | 3306.9 | 2585.1 | 1792 | 7062 |  |
| 3607SP | 35São Paulo | Monte Azul  Paulista | 353150 | 3531506 | 19123.1 | 3757.7 | 2689.5 | 2102.4 | 1457.4 | 9431 |  |
| 3608SP | 35São Paulo | Monte Castelo | 353160 | 3531605 | 4145.7 | 814.6 | 583.1 | 455.8 | 316 | 2155 |  |
| 3609SP | 35São Paulo | Monteiro Lobato | 353170 | 3531704 | 4406.7 | 865.9 | 619.8 | 484.5 | 335.8 | 1703 |  |
| 3610SP | 35São Paulo | Monte Mor | 353180 | 3531803 | 54496.2 | 10708.5 | 7664.3 | 5991.4 | 4153.3 | 7167 |  |
| 3611SP | 35São Paulo | Morro Agudo | 353190 | 3531902 | 31186.1 | 6128.1 | 4386 | 3428.7 | 2376.8 | 9537 |  |
| 3612SP | 35São Paulo | Morungaba | 353200 | 3532009 | 12746.2 | 2504.6 | 1792.6 | 1401.3 | 971.4 | 3469 |  |
| 3613SP | 35São Paulo | Motuca | 353205 | 3532058 | 4547.4 | 893.6 | 639.5 | 499.9 | 346.6 | 1025 |  |
| 3614SP | 35São Paulo | Murutinga do Sul | 353210 | 3532108 | 4363.2 | 857.4 | 613.6 | 479.7 | 332.5 | 2900 |  |
| 3615SP | 35São Paulo | Nantes | 353215 | 3532157 | 2935.6 | 576.8 | 412.9 | 322.7 | 223.7 | 1400 |  |
| 3616SP | 35São Paulo | Narandiba | 353220 | 3532207 | 4593.5 | 902.6 | 646 | 505 | 350.1 | 1458 |  |
| 3617SP | 35São Paulo | Natividade da  Serra | 353230 | 3532306 | 6724.8 | 1321.4 | 945.8 | 739.3 | 512.5 | 2126 |  |
| 3618SP |  | Nazaré Paulista | 353240 | 3532405 | 17551.9 | 3448.9 | 2468.5 | 1929.7 | 1337.7 | 3877 |  |
| 3619SP |  | Neves Paulista | 353250 | 3532504 | 8919.1 | 1752.6 | 1254.4 | 980.6 | 679.7 | 3531 |  |
| 3620SP |  | Nhandeara | 353260 | 3532603 | 11171.1 | 2195.1 | 1571.1 | 1228.2 | 851.4 | 6008 |  |
| 3621SP |  | Nipoã | 353270 | 3532702 | 4755.8 | 934.5 | 668.9 | 522.9 | 362.5 | 1647 |  |
| 3622SP |  | Nova Aliança | 353280 | 3532801 | 6453.9 | 1268.2 | 907.7 | 709.6 | 491.9 | 2859 |  |
| 3623SP |  | Nova Campina | 353282 | 3532827 | 9174.8 | 1802.8 | 1290.3 | 1008.7 | 699.2 | 987 |  |
| 3624SP |  | Nova Canaã  Paulista | 353284 | 3532843 | 2021.2 | 397.2 | 284.3 | 222.2 | 154 | 1737 |  |
| 3625SP |  | Nova Castilho | 353286 | 3532868 | 1201.3 | 236.1 | 169 | 132.1 | 91.6 | 602 |  |
| 3626SP |  | Nova Europa | 353290 | 3532900 | 10273 | 2018.6 | 1444.8 | 1129.4 | 782.9 | 2637 |  |
| 3627SP |  | Nova Granada | 353300 | 3533007 | 20440.4 | 4016.5 | 2874.7 | 2247.3 | 1557.8 | 6920 |  |
| 3628SP |  | Nova  Guataporanga | 353310 | 3533106 | 2261.3 | 444.3 | 318 | 248.6 | 172.3 | 1374 |  |

1

1

1

5

5

1

4

1

9

6

2

2

5

1

3

3

1

1

1

9

5

4

6

3

2

1

1

1

2

8

1

2

5

1

1

1

1

5

1

4

7

4

3

2

2

4

3

3

1

1

1

2

2

| 3629SP |  | Nova  Independência | 353320 | 3533205 | 3521.5 | 692 | 495.3 | 387.2 | 268.4 | 1934 |  |
| --- | --- | --- | --- | --- | --- | --- | --- | --- | --- | --- | --- |
| 3630SP |  | Novais | 353325 | 3533254 | 5218.2 | 1025.4 | 733.9 | 573.7 | 397.7 | 1263 |  |
| 3631SP |  | Nova Luzitânia | 353330 | 3533304 | 3783.3 | 743.4 | 532.1 | 415.9 | 288.3 | 1730 |  |
| 3632SP |  | Nova Odessa | 353340 | 3533403 | 55909.2 | 10986.2 | 7863.1 | 6146.8 | 4261 | 10678 |  |
| 3633SP |  | Novo Horizonte | 353350 | 3533502 | 39013.4 | 7666.1 | 5486.8 | 4289.2 | 2973.3 | 11805 |  |
| 3634SP |  | Nuporanga | 353360 | 3533601 | 7165.4 | 1408 | 1007.7 | 787.8 | 546.1 | 2098 |  |
| 3635SP |  | Ocauçu | 353370 | 3533700 | 4257.5 | 836.6 | 598.8 | 468.1 | 324.5 | 1578 |  |
| 3636SP |  | Óleo | 353380 | 3533809 | 2610.8 | 513 | 367.2 | 287 | 199 | 999 |  |
| 3637SP | 35São Paulo | Olímpia | 353390 | 3533908 | 52692.3 | 10354 | 7410.6 | 5793.1 | 4015.8 | 18154 |  |
| 3638SP | 35São Paulo | Onda Verde | 353400 | 3534005 | 4152 | 815.9 | 583.9 | 456.5 | 316.4 | 2015 |  |
| 3639SP | 35São Paulo | Oriente | 353410 | 3534104 | 6346 | 1247 | 892.5 | 697.7 | 483.6 | 2409 |  |
| 3640SP | 35São Paulo | Orindiúva | 353420 | 3534203 | 6382.3 | 1254.1 | 897.6 | 701.7 | 486.4 | 1837 |  |
| 3641SP | 35São Paulo | Orlândia | 353430 | 3534302 | 42127.3 | 8278 | 5924.8 | 4631.5 | 3210.6 | 14671 |  |
| 3642SP | 35São Paulo | Osasco | 353440 | 3534401 | 687274.6 | 135049.5 | 96658.3 | 75560.2 | 52379.1 | 136169 | 47 |
| 3643SP | 35São Paulo | Oscar Bressane | 353450 | 3534500 | 2588.7 | 508.7 | 364.1 | 284.6 | 197.3 | 1764 |  |
| 3644SP | 35São Paulo | Osvaldo Cruz | 353460 | 3534609 | 32103.2 | 6308.3 | 4515 | 3529.5 | 2446.7 | 6509 |  |
| 3645SP | 35São Paulo | Ourinhos | 353470 | 3534708 | 108876.1 | 21394.2 | 15312.3 | 11970 | 8297.8 | 34223 | 10 |
| 3646SP | 35São Paulo | Ouroeste | 353475 | 3534757 | 9403.6 | 1847.8 | 1322.5 | 1033.8 | 716.7 | 6009 |  |
| 3647SP | 35São Paulo | Ouro Verde | 353480 | 3534807 | 8226 | 1616.4 | 1156.9 | 904.4 | 626.9 | 3862 |  |
| 3648SP | 35São Paulo | Pacaembu | 353490 | 3534906 | 13796.2 | 2711 | 1940.3 | 1516.8 | 1051.4 | 4789 |  |
| 3649SP | 35São Paulo | Palestina | 353500 | 3535002 | 12047.6 | 2367.4 | 1694.4 | 1324.5 | 918.2 | 4531 |  |
| 3650SP | 35São Paulo | Palmares Paulista | 353510 | 3535101 | 12136.1 | 2384.7 | 1706.8 | 1334.3 | 924.9 | 2458 |  |
| 3651SP | 35São Paulo | Palmeira d'Oeste | 353520 | 3535200 | 9519.1 | 1870.5 | 1338.8 | 1046.5 | 725.5 | 6835 |  |
| 3652SP | 35São Paulo | Palmital | 353530 | 3535309 | 21852.2 | 4294 | 3073.3 | 2402.5 | 1665.4 | 6051 |  |
| 3653SP | 35São Paulo | Panorama | 353540 | 3535408 | 15270.8 | 3000.7 | 2147.7 | 1678.9 | 1163.8 | 5525 |  |
| 3654SP | 35São Paulo | Paraguaçu  Paulista | 353550 | 3535507 | 44253.9 | 8695.9 | 6223.9 | 4865.4 | 3372.7 | 13764 |  |
| 3655SP | 35São Paulo | Paraibuna | 353560 | 3535606 | 17927.3 | 3522.7 | 2521.3 | 1971 | 1366.3 | 5018 |  |
| 3656SP | 35São Paulo | Paraíso | 353570 | 3535705 | 6211.1 | 1220.5 | 873.5 | 682.9 | 473.4 | 2139 |  |
| 3657SP | 35São Paulo | Paranapanema | 353580 | 3535804 | 19089.8 | 3751.1 | 2684.8 | 2098.8 | 1454.9 | 1936 |  |
| 3658SP | 35São Paulo | Paranapuã | 353590 | 3535903 | 3971.4 | 780.4 | 558.5 | 436.6 | 302.7 | 2417 |  |
| 3659SP | 35São Paulo | Parapuã | 353600 | 3536000 | 10990.7 | 2159.7 | 1545.7 | 1208.3 | 837.6 | 4035 |  |
| 3660SP | 35São Paulo | Pardinho | 353610 | 3536109 | 6033.7 | 1185.6 | 848.6 | 663.4 | 459.8 | 1963 |  |
| 3661SP | 35São Paulo | Pariquera-Açu | 353620 | 3536208 | 19168.6 | 3766.6 | 2695.9 | 2107.4 | 1460.9 | 6521 |  |
| 3662SP | 35São Paulo | Parisi | 353625 | 3536257 | 2109.9 | 414.6 | 296.7 | 232 | 160.8 | 2035 |  |
| 3663SP | 35São Paulo | Patrocínio Paulista | 353630 | 3536307 | 13900.8 | 2731.5 | 1955 | 1528.3 | 1059.4 | 3280 |  |
| 3664SP | 35São Paulo | Paulicéia | 353640 | 3536406 | 6878.9 | 1351.7 | 967.4 | 756.3 | 524.3 | 2488 |  |
| 3665SP | 35São Paulo | Paulínia | 353650 | 3536505 | 95787.6 | 18822.3 | 13471.6 | 10531.1 | 7300.2 | 19719 |  |
| 3666SP | 35São Paulo | Paulistânia | 353657 | 3536570 | 1819.5 | 357.5 | 255.9 | 200 | 138.7 | 371 |  |
| 3667SP | 35São Paulo | Paulo de Faria | 353660 | 3536604 | 8828.7 | 1734.8 | 1241.7 | 970.6 | 672.9 | 2876 |  |
| 3668SP | 35São Paulo | Pederneiras | 353670 | 3536703 | 44304.5 | 8705.8 | 6231 | 4870.9 | 3376.6 | 3792 |  |
| 3669SP | 35São Paulo | Pedra Bela | 353680 | 3536802 | 5976.7 | 1174.4 | 840.6 | 657.1 | 455.5 | 2475 |  |
| 3670SP | 35São Paulo | Pedranópolis | 353690 | 3536901 | 2548.8 | 500.8 | 358.5 | 280.2 | 194.3 | 2151 |  |
| 3671SP | 35São Paulo | Pedregulho | 353700 | 3537008 | 16325.7 | 3208 | 2296 | 1794.9 | 1244.2 | 3799 |  |
| 3672SP | 35São Paulo | Pedreira | 353710 | 3537107 | 44923.1 | 8827.4 | 6318 | 4938.9 | 3423.7 | 8936 |  |
| 3673SP | 35São Paulo | Pedrinhas Paulista | 353715 | 3537156 | 3036.9 | 596.8 | 427.1 | 333.9 | 231.5 | 1825 |  |
| 3674SP | 35São Paulo | Pedro de Toledo | 353720 | 3537206 | 10824.2 | 2127 | 1522.3 | 1190 | 824.9 | 2414 |  |
| 3675SP | 35São Paulo | Penápolis | 353730 | 3537305 | 61318.2 | 12049 | 8623.8 | 6741.4 | 4673.2 | 32921 | 11 |
| 3676SP | 35São Paulo | Pereira Barreto | 353740 | 3537404 | 25503.4 | 5011.4 | 3586.8 | 2803.9 | 1943.7 | 13970 |  |
| 3677SP | 35São Paulo | Pereiras | 353750 | 3537503 | 8092.2 | 1590.1 | 1138.1 | 889.7 | 616.7 | 2062 |  |
| 3678SP | 35São Paulo | Peruíbe | 353760 | 3537602 | 64309.8 | 12636.9 | 9044.5 | 7070.3 | 4901.2 | 16774 |  |
| 3679SP | 35São Paulo | Piacatu | 353770 | 3537701 | 5660.1 | 1112.2 | 796 | 622.3 | 431.4 | 3402 |  |
| 3680SP | 35São Paulo | Piedade | 353780 | 3537800 | 54094.6 | 10629.6 | 7607.9 | 5947.3 | 4122.7 | 8818 |  |
| 3681SP | 35São Paulo | Pilar do Sul | 353790 | 3537909 | 27944.7 | 5491.1 | 3930.1 | 3072.3 | 2129.7 | 5814 |  |
| 3682SP | 35São Paulo | Pindamonhangaba | 353800 | 3538006 | 158343.1 | 31114.4 | 22269.4 | 17408.5 | 12067.8 | 49232 | 16 |
| 3683SP | 35São Paulo | Pindorama | 353810 | 3538105 | 16117.5 | 3167.1 | 2266.8 | 1772 | 1228.4 | 4308 |  |
| 3684SP | 35São Paulo | Pinhalzinho | 353820 | 3538204 | 14212.1 | 2792.7 | 1998.8 | 1562.5 | 1083.1 | 6092 |  |
| 3685SP | 35São Paulo | Piquerobi | 353830 | 3538303 | 3639.7 | 715.2 | 511.9 | 400.2 | 277.4 | 1278 |  |
| 3686SP | 35São Paulo | Piquete | 353850 | 3538501 | 14009.5 | 2752.9 | 1970.3 | 1540.2 | 1067.7 | 2866 |  |
| 3687SP | 35São Paulo | Piracaia | 353860 | 3538600 | 26361.8 | 5180.1 | 3707.5 | 2898.3 | 2009.1 | 8357 |  |
| 3688SP | 35São Paulo | Piracicaba | 353870 | 3538709 | 386375.9 | 75922.9 | 54339.9 | 42478.8 | 29446.8 | 94885 | 32 |
| 3689SP | 35São Paulo | Piraju | 353880 | 3538808 | 29341.7 | 5765.6 | 4126.6 | 3225.9 | 2236.2 | 13642 |  |
| 3690SP | 35São Paulo | Pirajuí | 353890 | 3538907 | 24216.1 | 4758.5 | 3405.8 | 2662.4 | 1845.6 | 2746 |  |
| 3691SP | 35São Paulo | Pirangi | 353900 | 3539004 | 11087.6 | 2178.7 | 1559.4 | 1219 | 845 | 4152 |  |
| 3692SP | 35São Paulo | Pirapora do Bom  Jesus | 353910 | 3539103 | 17365.5 | 3412.3 | 2442.3 | 1909.2 | 1323.5 | 3170 |  |
| 3693SP | 35São Paulo | Pirapozinho | 353920 | 3539202 | 26244 | 5156.9 | 3691 | 2885.3 | 2000.1 | 8077 |  |
| 3694SP | 35São Paulo | Pirassununga | 353930 | 3539301 | 73665 | 14475.2 | 10360.2 | 8098.9 | 5614.2 | 10840 |  |
| 3695SP | 35São Paulo | Piratininga | 353940 | 3539400 | 12914.5 | 2537.7 | 1816.3 | 1419.8 | 984.3 | 1623 |  |
| 3696SP | 35São Paulo | Pitangueiras | 353950 | 3539509 | 37694.8 | 7407 | 5301.4 | 4144.2 | 2872.8 | 7490 |  |
| 3697SP | 35São Paulo | Planalto | 353960 | 3539608 | 4867.1 | 956.4 | 684.5 | 535.1 | 370.9 | 1808 |  |
| 3698SP | 35São Paulo | Platina | 353970 | 3539707 | 3388.9 | 665.9 | 476.6 | 372.6 | 258.3 | 755 |  |
| 3699SP | 35São Paulo | Poá | 353980 | 3539806 | 112321.1 | 22071.1 | 15796.8 | 12348.8 | 8560.3 | 31752 | 10 |
| 3700SP | 35São Paulo | Poloni | 353990 | 3539905 | 5754.8 | 1130.8 | 809.4 | 632.7 | 438.6 | 2071 |  |
| 3701SP | 35São Paulo | Pompéia | 354000 | 3540002 | 21102.1 | 4146.6 | 2967.8 | 2320 | 1608.3 | 3888 |  |
| 3702SP | 35São Paulo | Pongaí | 354010 | 3540101 | 3478.5 | 683.5 | 489.2 | 382.4 | 265.1 | 732 |  |
| 3703SP | 35São Paulo | Pontal | 354020 | 3540200 | 45193.6 | 8880.5 | 6356 | 4968.7 | 3444.3 | 5738 |  |
| 3704SP | 35São Paulo | Pontalinda | 354025 | 3540259 | 4370.9 | 858.9 | 614.7 | 480.5 | 333.1 | 2780 |  |
| 3705SP | 35São Paulo | Pontes Gestal | 354030 | 3540309 | 2566.9 | 504.4 | 361 | 282.2 | 195.6 | 1645 |  |
| 3706SP | 35São Paulo | Populina | 354040 | 3540408 | 4231.9 | 831.6 | 595.2 | 465.3 | 322.5 | 2632 |  |
| 3707SP | 35São Paulo | Porangaba | 354050 | 3540507 | 9154.4 | 1798.8 | 1287.5 | 1006.5 | 697.7 | 2135 |  |
| 3708SP | 35São Paulo | Porto Feliz | 354060 | 3540606 | 51293.6 | 10079.2 | 7213.9 | 5639.3 | 3909.2 | 4958 |  |
| 3709SP | 35São Paulo | Porto Ferreira | 354070 | 3540705 | 54080.2 | 10626.8 | 7605.8 | 5945.7 | 4121.6 | 9643 |  |
| 3710SP | 35São Paulo | Potim | 354075 | 3540754 | 22050.6 | 4332.9 | 3101.2 | 2424.3 | 1680.5 | 2704 |  |
| 3711SP | 35São Paulo | Potirendaba | 354080 | 3540804 | 16484.4 | 3239.2 | 2318.4 | 1812.3 | 1256.3 | 4972 |  |
| 3712SP | 35São Paulo | Pracinha | 354085 | 3540853 | 3468.6 | 681.6 | 487.8 | 381.3 | 264.4 | 1467 |  |
| 3713SP | 35São Paulo | Pradópolis | 354090 | 3540903 | 19478.1 | 3827.4 | 2739.4 | 2141.5 | 1484.5 | 1546 |  |
| 3714SP | 35São Paulo | Praia Grande | 354100 | 3541000 | 294161.8 | 57802.8 | 41370.9 | 32340.7 | 22418.9 | 85971 | 22 |
| 3715SP | 35São Paulo | Pratânia | 354105 | 3541059 | 4951 | 972.9 | 696.3 | 544.3 | 377.3 | 1132 |  |
| 3716SP | 35São Paulo | Presidente Alves | 354110 | 3541109 | 4142.5 | 814 | 582.6 | 455.4 | 315.7 | 796 |  |
| 3717SP | 35São Paulo | Presidente  Bernardes | 354120 | 3541208 | 13460.9 | 2645.1 | 1893.1 | 1479.9 | 1025.9 | 4147 |  |
| 3718SP | 35São Paulo | Presidente  Epitácio | 354130 | 3541307 | 43027.4 | 8454.9 | 6051.4 | 4730.5 | 3279.2 | 11489 |  |
| 3719SP | 35São Paulo | Presidente  Prudente | 354140 | 3541406 | 219361.3 | 43104.5 | 30851 | 24117 | 16718.1 | 69214 | 24 |
| 3720SP | 35São Paulo | Presidente  Venceslau | 354150 | 3541505 | 38985.2 | 7660.6 | 5482.9 | 4286.1 | 2971.2 | 11222 |  |
| 3721SP | 35São Paulo | Promissão | 354160 | 3541604 | 38229.6 | 7512.1 | 5376.6 | 4203 | 2913.6 | 10382 |  |
| 3722SP | 35São Paulo | Quadra | 354165 | 3541653 | 3532.8 | 694.2 | 496.9 | 388.4 | 269.2 | 1649 |  |
| 3723SP | 35São Paulo | Quatá | 354170 | 3541703 | 13527 | 2658.1 | 1902.4 | 1487.2 | 1030.9 | 5105 |  |
| 3724SP | 35São Paulo | Queiroz | 354180 | 3541802 | 3115.1 | 612.1 | 438.1 | 342.5 | 237.4 | 841 |  |
| 3725SP | 35São Paulo | Queluz | 354190 | 3541901 | 12405.5 | 2437.7 | 1744.7 | 1363.9 | 945.5 | 1728 |  |
| 3726SP | 35São Paulo | Quintana | 354200 | 3542008 | 6354.4 | 1248.6 | 893.7 | 698.6 | 484.3 | 3164 |  |
| 3727SP | 35São Paulo | Rafard | 354210 | 3542107 | 8903.9 | 1749.6 | 1252.2 | 978.9 | 678.6 | 2260 |  |
| 3728SP | 35São Paulo | Rancharia | 354220 | 3542206 | 29469.9 | 5790.8 | 4144.6 | 3240 | 2246 | 14564 |  |
| 3729SP | 35São Paulo | Redenção da  Serra | 354230 | 3542305 | 3894.4 | 765.2 | 547.7 | 428.2 | 296.8 | 1784 |  |
| 3730SP | 35São Paulo | Regente Feijó | 354240 | 3542404 | 19485.6 | 3828.9 | 2740.5 | 2142.3 | 1485.1 | 10123 |  |
| 3731SP | 35São Paulo | Reginópolis | 354250 | 3542503 | 8475.3 | 1665.4 | 1192 | 931.8 | 645.9 | 1298 |  |
| 3732SP | 35São Paulo | Registro | 354260 | 3542602 | 55686.2 | 10942.3 | 7831.7 | 6122.2 | 4244 | 14878 |  |
| 3733SP | 35São Paulo | Restinga | 354270 | 3542701 | 7119.1 | 1398.9 | 1001.2 | 782.7 | 542.6 | 1428 |  |

3

3

7

4

1

2

1

1

1

2

1

1

4

1

1

1

1

6

1

3

5

5

1

3

2

1

1

1

3

3

1

1

2

3

2

2

1

1

3

1

1

1

4

3

2

1

4

2

3

| 3734SP | 35São Paulo | Ribeira | 354280 | 3542800 | 3377.4 | 663.7 | 475 | 371.3 | 257.4 | 1247 |  |
| --- | --- | --- | --- | --- | --- | --- | --- | --- | --- | --- | --- |
| 3735SP | 35São Paulo | Ribeirão Bonito | 354290 | 3542909 | 12750 | 2505.4 | 1793.2 | 1401.8 | 971.7 | 3551 |  |
| 3736SP | 35São Paulo | Ribeirão Branco | 354300 | 3543006 | 17551.6 | 3448.9 | 2468.5 | 1929.7 | 1337.7 | 3534 |  |
| 3737SP | 35São Paulo | Ribeirão Corrente | 354310 | 3543105 | 4519.9 | 888.2 | 635.7 | 496.9 | 344.5 | 1807 |  |
| 3738SP | 35São Paulo | Ribeirão do Sul | 354320 | 3543204 | 4527.5 | 889.7 | 636.7 | 497.8 | 345.1 | 2183 |  |
| 3739SP | 35São Paulo | Ribeirão dos  Índios | 354323 | 3543238 | 2223.2 | 436.9 | 312.7 | 244.4 | 169.4 | 1071 |  |
| 3740SP | 35São Paulo | Ribeirão Grande | 354325 | 3543253 | 7602.3 | 1493.9 | 1069.2 | 835.8 | 579.4 | 1759 |  |
| 3741SP | 35São Paulo | Ribeirão Pires | 354330 | 3543303 | 118904.3 | 23364.7 | 16722.7 | 13072.5 | 9062 | 10078 |  |
| 3742SP | 35São Paulo | Ribeirão Preto | 354340 | 3543402 | 656523.9 | 129006.9 | 92333.5 | 72179.4 | 50035.5 | 132695 | 45 |
| 3743SP | 35São Paulo | Riversul | 354350 | 3543501 | 5909.8 | 1161.3 | 831.2 | 649.7 | 450.4 | 998 |  |
| 3744SP | 35São Paulo | Rifaina | 354360 | 3543600 | 3556.4 | 698.8 | 500.2 | 391 | 271 | 1289 |  |
| 3745SP | 35São Paulo | Rincão | 354370 | 3543709 | 10682.4 | 2099.1 | 1502.4 | 1174.4 | 814.1 | 2719 |  |
| 3746SP | 35São Paulo | Rinópolis | 354380 | 3543808 | 10038.5 | 1972.6 | 1411.8 | 1103.7 | 765.1 | 5416 |  |
| 3747SP | 35São Paulo | Rio Claro | 354390 | 3543907 | 197370.8 | 38783.4 | 27758.2 | 21699.3 | 15042.2 | 43595 | 14 |
| 3748SP | 35São Paulo | Rio das Pedras | 354400 | 3544004 | 32465.6 | 6379.5 | 4566 | 3569.3 | 2474.3 | 4663 |  |
| 3749SP | 35São Paulo | Rio Grande da  Serra | 354410 | 3544103 | 47602.1 | 9353.8 | 6694.8 | 5233.5 | 3627.9 | 4558 |  |
| 3750SP | 35São Paulo | Riolândia | 354420 | 3544202 | 11585.4 | 2276.5 | 1629.4 | 1273.7 | 883 | 4567 |  |
| 3751SP | 35São Paulo | Rosana | 354425 | 3544251 | 18403.5 | 3616.3 | 2588.3 | 2023.3 | 1402.6 | 13211 |  |
| 3752SP | 35São Paulo | Roseira | 354430 | 3544301 | 10207.3 | 2005.7 | 1435.6 | 1122.2 | 777.9 | 4095 |  |
| 3753SP | 35São Paulo | Rubiácea | 354440 | 3544400 | 2940.9 | 577.9 | 413.6 | 323.3 | 224.1 | 1622 |  |
| 3754SP | 35São Paulo | Rubinéia | 354450 | 3544509 | 3021.5 | 593.7 | 424.9 | 332.2 | 230.3 | 2562 |  |
| 3755SP | 35São Paulo | Sabino | 354460 | 3544608 | 5437.2 | 1068.4 | 764.7 | 597.8 | 414.4 | 2273 |  |
| 3756SP | 35São Paulo | Sagres | 354470 | 3544707 | 2432.4 | 478 | 342.1 | 267.4 | 185.4 | 1043 |  |
| 3757SP | 35São Paulo | Sales | 354480 | 3544806 | 5914.2 | 1162.1 | 831.8 | 650.2 | 450.7 | 2855 |  |
| 3758SP | 35São Paulo | Sales Oliveira | 354490 | 3544905 | 11283.4 | 2217.2 | 1586.9 | 1240.5 | 859.9 | 3107 |  |
| 3759SP | 35São Paulo | Salesópolis | 354500 | 3545001 | 16478.3 | 3238 | 2317.5 | 1811.7 | 1255.9 | 3323 |  |
| 3760SP | 35São Paulo | Salmourão | 354510 | 3545100 | 5086.7 | 999.5 | 715.4 | 559.2 | 387.7 | 1658 |  |
| 3761SP | 35São Paulo | Saltinho | 354515 | 3545159 | 7700.4 | 1513.1 | 1083 | 846.6 | 586.9 | 2912 |  |
| 3762SP | 35São Paulo | Salto | 354520 | 3545209 | 112632 | 22132.2 | 15840.6 | 12383 | 8584 | 23400 |  |
| 3763SP | 35São Paulo | Salto de Pirapora | 354530 | 3545308 | 42976.2 | 8444.8 | 6044.2 | 4724.9 | 3275.3 | 11639 |  |
| 3764SP | 35São Paulo | Salto Grande | 354540 | 3545407 | 9117.8 | 1791.6 | 1282.3 | 1002.4 | 694.9 | 4122 |  |
| 3765SP | 35São Paulo | Sandovalina | 354550 | 3545506 | 4016.3 | 789.2 | 564.9 | 441.6 | 306.1 | 814 |  |
| 3766SP | 35São Paulo | Santa Adélia | 354560 | 3545605 | 14995.7 | 2946.7 | 2109 | 1648.7 | 1142.9 | 4586 |  |
| 3767SP | 35São Paulo | Santa Albertina | 354570 | 3545704 | 5905.4 | 1160.4 | 830.5 | 649.3 | 450.1 | 4340 |  |
| 3768SP | 35São Paulo | Santa Bárbara d'Oeste | 354580 | 3545803 | 187888.7 | 36920.1 | 26424.7 | 20656.8 | 14319.5 | 45473 | 16 |
| 3769SP | 35São Paulo | Santa Branca | 354600 | 3546009 | 14362.8 | 2822.3 | 2020 | 1579.1 | 1094.6 | 3935 |  |
| 3770SP | 35São Paulo | Santa Clara d'Oeste | 354610 | 3546108 | 2116.4 | 415.9 | 297.7 | 232.7 | 161.3 | 1862 |  |
| 3771SP | 35São Paulo | Santa Cruz da  Conceição | 354620 | 3546207 | 4273.3 | 839.7 | 601 | 469.8 | 325.7 | 1711 |  |
| 3772SP | 35São Paulo | Santa Cruz da  Esperança | 354625 | 3546256 | 2057.5 | 404.3 | 289.4 | 226.2 | 156.8 | 820 |  |
| 3773SP | 35São Paulo | Santa Cruz das  Palmeiras | 354630 | 3546306 | 32283.2 | 6343.6 | 4540.3 | 3549.3 | 2460.4 | 5051 |  |
| 3774SP | 35São Paulo | Santa Cruz do Rio  Pardo | 354640 | 3546405 | 46064.7 | 9051.7 | 6478.5 | 5064.4 | 3510.7 | 20189 |  |
| 3775SP | 35São Paulo | Santa Ernestina | 354650 | 3546504 | 5628.2 | 1105.9 | 791.6 | 618.8 | 428.9 | 1011 |  |
| 3776SP | 35São Paulo | Santa Fé do Sul | 354660 | 3546603 | 30944.9 | 6080.7 | 4352.1 | 3402.1 | 2358.4 | 20938 |  |
| 3777SP | 35São Paulo | Santa Gertrudes | 354670 | 3546702 | 24313.9 | 4777.7 | 3419.5 | 2673.1 | 1853 | 4786 |  |
| 3778SP | 35São Paulo | Santa Isabel | 354680 | 3546801 | 54162.9 | 10643 | 7617.5 | 5954.8 | 4127.9 | 19560 |  |
| 3779SP | 35São Paulo | Santa Lúcia | 354690 | 3546900 | 8586.2 | 1687.2 | 1207.6 | 944 | 654.4 | 1822 |  |
| 3780SP | 35São Paulo | Santa Maria da  Serra | 354700 | 3547007 | 5818.9 | 1143.4 | 818.4 | 639.7 | 443.5 | 1769 |  |
| 3781SP | 35São Paulo | Santa Mercedes | 354710 | 3547106 | 2905.9 | 571 | 408.7 | 319.5 | 221.5 | 1607 |  |
| 3782SP | 35São Paulo | Santana da Ponte  Pensa | 354720 | 3547205 | 1581.8 | 310.8 | 222.5 | 173.9 | 120.6 | 1675 |  |
| 3783SP | 35São Paulo | Santana de  Parnaíba | 354730 | 3547304 | 124268.9 | 24418.8 | 17477.2 | 13662.3 | 9470.9 | 54285 | 23 |
| 3784SP | 35São Paulo | Santa Rita d'Oeste | 354740 | 3547403 | 2542.4 | 499.6 | 357.6 | 279.5 | 193.8 | 2703 |  |
| 3785SP | 35São Paulo | Santa Rita do  Passa Quatro | 354750 | 3547502 | 27208.9 | 5346.5 | 3826.7 | 2991.4 | 2073.7 | 4395 |  |
| 3786SP | 35São Paulo | Santa Rosa de  Viterbo | 354760 | 3547601 | 25330.8 | 4977.5 | 3562.5 | 2784.9 | 1930.5 | 6060 |  |
| 3787SP | 35São Paulo | Santa Salete | 354765 | 3547650 | 1505.8 | 295.9 | 211.8 | 165.6 | 114.8 | 1263 |  |
| 3788SP | 35São Paulo | Santo Anastácio | 354770 | 3547700 | 20835.1 | 4094.1 | 2930.2 | 2290.6 | 1587.9 | 6230 |  |
| 3789SP | 35São Paulo | Santo André | 354780 | 3547809 | 702101.5 | 137962.9 | 98743.6 | 77190.3 | 53509.1 | 174239 | 51 |
| 3790SP | 35São Paulo | Santo Antônio da  Alegria | 354790 | 3547908 | 6653.3 | 1307.4 | 935.7 | 731.5 | 507.1 | 2272 |  |
| 3791SP | 35São Paulo | Santo Antônio de  Posse | 354800 | 3548005 | 22084.1 | 4339.5 | 3105.9 | 2428 | 1683.1 | 5727 |  |
| 3792SP | 35São Paulo | Santo Antônio do  Aracanguá | 354805 | 3548054 | 8065.2 | 1584.8 | 1134.3 | 886.7 | 614.7 | 5147 |  |
| 3793SP | 35São Paulo | Santo Antônio do  Jardim | 354810 | 3548104 | 5997 | 1178.4 | 843.4 | 659.3 | 457 | 1358 |  |
| 3794SP | 35São Paulo | Santo Antônio do  Pinhal | 354820 | 3548203 | 6693.3 | 1315.2 | 941.3 | 735.9 | 510.1 | 2975 |  |
| 3795SP | 35São Paulo | Santo Expedito | 354830 | 3548302 | 2972.3 | 584.1 | 418 | 326.8 | 226.5 | 1466 |  |
| 3796SP | 35São Paulo | Santópolis do  Aguapeí | 354840 | 3548401 | 4550.1 | 894.1 | 639.9 | 500.2 | 346.8 | 1981 |  |
| 3797SP | 35São Paulo | Santos | 354850 | 3548500 | 429457.6 | 84388.4 | 60398.9 | 47215.3 | 32730.2 | 131349 | 40 |
| 3798SP | 35São Paulo | São Bento do  Sapucaí | 354860 | 3548609 | 10748.9 | 2112.2 | 1511.7 | 1181.8 | 819.2 | 5428 |  |
| 3799SP | 35São Paulo | São Bernardo do  Campo | 354870 | 3548708 | 806676.6 | 158512 | 113451 | 88687.4 | 61479.1 | 235837 | 75 |
| 3800SP | 35São Paulo | São Caetano do  Sul | 354880 | 3548807 | 156128.9 | 30679.3 | 21958 | 17165.1 | 11899 | 63221 | 16 |
| 3801SP | 35São Paulo | São Carlos | 354890 | 3548906 | 238049.9 | 46776.8 | 33479.3 | 26171.6 | 18142.5 | 73696 | 25 |
| 3802SP | 35São Paulo | São Francisco | 354900 | 3549003 | 2829.7 | 556 | 398 | 311.1 | 215.7 | 2670 |  |
| 3803SP | 35São Paulo | São João da Boa  Vista | 354910 | 3549102 | 87925.9 | 17277.4 | 12365.9 | 9666.7 | 6701.1 | 26405 |  |
| 3804SP | 35São Paulo | São João das  Duas Pontes | 354920 | 3549201 | 2588.1 | 508.6 | 364 | 284.5 | 197.2 | 1423 |  |
| 3805SP | 35São Paulo | São João de  Iracema | 354925 | 3549250 | 1862.2 | 365.9 | 261.9 | 204.7 | 141.9 | 1301 |  |
| 3806SP | 35São Paulo | São João do Pau d'Alho | 354930 | 3549300 | 2121.2 | 416.8 | 298.3 | 233.2 | 161.7 | 1424 |  |
| 3807SP | 35São Paulo | São Joaquim da  Barra | 354940 | 3549409 | 49449.3 | 9716.8 | 6954.5 | 5436.5 | 3768.7 | 13372 |  |
| 3808SP | 35São Paulo | São José da Bela  Vista | 354950 | 3549508 | 8722.8 | 1714 | 1226.8 | 959 | 664.8 | 2054 |  |
| 3809SP | 35São Paulo | São José do  Barreiro | 354960 | 3549607 | 4144.2 | 814.3 | 582.8 | 455.6 | 315.8 | 1046 |  |
| 3810SP | 35São Paulo | São José do Rio  Pardo | 354970 | 3549706 | 53774.3 | 10566.6 | 7562.8 | 5912 | 4098.3 | 9369 |  |
| 3811SP | 35São Paulo | São José do Rio  Preto | 354980 | 3549805 | 436526.5 | 85777.5 | 61393.1 | 47992.5 | 33268.9 | 147261 | 51 |
| 3812SP | 35São Paulo | São José dos  Campos | 354990 | 3549904 | 678838.6 | 133391.8 | 95471.9 | 74632.7 | 51736.2 | 263309 | 90 |
| 3813SP | 35São Paulo | São Lourenço da  Serra | 354995 | 3549953 | 14968.2 | 2941.3 | 2105.1 | 1645.6 | 1140.8 | 5039 |  |
| 3814SP | 35São Paulo | São Luís do  Paraitinga | 355000 | 3550001 | 10620.9 | 2087 | 1493.7 | 1167.7 | 809.4 | 5025 |  |
| 3815SP | 35São Paulo | São Manuel | 355010 | 3550100 | 39898.9 | 7840.1 | 5611.4 | 4386.6 | 3040.8 | 6768 |  |
| 3816SP | 35São Paulo | São Miguel  Arcanjo | 355020 | 3550209 | 32412.5 | 6369.1 | 4558.5 | 3563.5 | 2470.2 | 10351 |  |
| 3817SP | 35São Paulo | São Paulo | 355030 | 3550308 | 11820591.2 | 2322746.2 | 1662447.9 | 1299576.5 | 900880.5 | 2994730 | 943 |

1

1

2

1

1

1

1

5

1

1

1

1

1

8

4

1

1

1

1

1

6

7

2

6

1

1

1

2

1

1

1

9

4

2

1

1

1

3

| 3818SP | 35São Paulo | São Pedro | 355040 | 3550407 | 33819.6 | 6645.6 | 4756.4 | 3718.2 | 2577.5 | 9743 |  |
| --- | --- | --- | --- | --- | --- | --- | --- | --- | --- | --- | --- |
| 3819SP | 35São Paulo | São Pedro do  Turvo | 355050 | 3550506 | 7480 | 1469.8 | 1052 | 822.4 | 570.1 | 2799 |  |
| 3820SP | 35São Paulo | São Roque | 355060 | 3550605 | 85265.3 | 16754.6 | 11991.7 | 9374.2 | 6498.3 | 19589 |  |
| 3821SP | 35São Paulo | São Sebastião | 355070 | 3550704 | 81697.4 | 16053.5 | 11489.9 | 8982 | 6226.4 | 23401 |  |
| 3822SP | 35São Paulo | São Sebastião da  Grama | 355080 | 3550803 | 12237.9 | 2404.7 | 1721.1 | 1345.5 | 932.7 | 5068 |  |
| 3823SP | 35São Paulo | São Simão | 355090 | 3550902 | 14928.2 | 2933.4 | 2099.5 | 1641.2 | 1137.7 | 3153 |  |
| 3824SP | 35São Paulo | São Vicente | 355100 | 3551009 | 351030.4 | 68977.5 | 49368.9 | 38592.9 | 26753 | 67283 | 21 |
| 3825SP | 35São Paulo | Sarapuí | 355110 | 3551108 | 9699 | 1905.9 | 1364.1 | 1066.3 | 739.2 | 2162 |  |
| 3826SP | 35São Paulo | Sarutaiá | 355120 | 3551207 | 3659.3 | 719.1 | 514.6 | 402.3 | 278.9 | 773 |  |
| 3827SP | 35São Paulo | Sebastianópolis do Sul | 355130 | 3551306 | 3285 | 645.5 | 462 | 361.2 | 250.4 | 2095 |  |
| 3828SP | 35São Paulo | Serra Azul | 355140 | 3551405 | 12967.4 | 2548.1 | 1823.7 | 1425.7 | 988.3 | 2506 |  |
| 3829SP | 35São Paulo | Serrana | 355150 | 3551504 | 42158.5 | 8284.1 | 5929.2 | 4635 | 3213 | 6340 |  |
| 3830SP | 35São Paulo | Serra Negra | 355160 | 3551603 | 27954.8 | 5493.1 | 3931.6 | 3073.4 | 2130.5 | 6728 |  |
| 3831SP | 35São Paulo | Sertãozinho | 355170 | 3551702 | 118461 | 23277.6 | 16660.4 | 13023.8 | 9028.2 | 29901 | 11 |
| 3832SP | 35São Paulo | Sete Barras | 355180 | 3551801 | 13029.4 | 2560.3 | 1832.5 | 1432.5 | 993 | 3097 |  |
| 3833SP | 35São Paulo | Severínia | 355190 | 3551900 | 16577.1 | 3257.4 | 2331.4 | 1822.5 | 1263.4 | 8603 |  |
| 3834SP | 35São Paulo | Silveiras | 355200 | 3552007 | 6082 | 1195.1 | 855.4 | 668.7 | 463.5 | 2379 |  |
| 3835SP | 35São Paulo | Socorro | 355210 | 3552106 | 39040.5 | 7671.5 | 5490.7 | 4292.2 | 2975.4 | 11869 |  |
| 3836SP | 35São Paulo | Sorocaba | 355220 | 3552205 | 635534.5 | 124882.5 | 89381.6 | 69871.8 | 48435.9 | 172616 | 60 |
| 3837SP | 35São Paulo | Sud Mennucci | 355230 | 3552304 | 7630.8 | 1499.5 | 1073.2 | 838.9 | 581.6 | 4698 |  |
| 3838SP | 35São Paulo | Sumaré | 355240 | 3552403 | 262265.1 | 51535.1 | 36885 | 28833.9 | 19988 | 46307 | 16 |
| 3839SP | 35São Paulo | Suzano | 355250 | 3552502 | 281347.8 | 55284.8 | 39568.8 | 30931.9 | 21442.3 | 60793 | 21 |
| 3840SP | 35São Paulo | Suzanápolis | 355255 | 3552551 | 3686.2 | 724.3 | 518.4 | 405.3 | 280.9 | 2519 |  |
| 3841SP | 35São Paulo | Tabapuã | 355260 | 3552601 | 11953.1 | 2348.8 | 1681.1 | 1314.1 | 911 | 4024 |  |
| 3842SP | 35São Paulo | Tabatinga | 355270 | 3552700 | 15667.5 | 3078.7 | 2203.5 | 1722.5 | 1194.1 | 4703 |  |
| 3843SP | 35São Paulo | Taboão da Serra | 355280 | 3552809 | 267992.9 | 52660.6 | 37690.5 | 29463.6 | 20424.5 | 56453 | 20 |
| 3844SP | 35São Paulo | Taciba | 355290 | 3552908 | 6032.5 | 1185.4 | 848.4 | 663.2 | 459.8 | 1574 |  |
| 3845SP | 35São Paulo | Taguaí | 355300 | 3553005 | 12357.4 | 2428.2 | 1737.9 | 1358.6 | 941.8 | 3325 |  |
| 3846SP | 35São Paulo | Taiaçu | 355310 | 3553104 | 6133 | 1205.1 | 862.5 | 674.3 | 467.4 | 3365 |  |
| 3847SP | 35São Paulo | Taiúva | 355320 | 3553203 | 5548.5 | 1090.3 | 780.3 | 610 | 422.9 | 3529 |  |
| 3848SP | 35São Paulo | Tambaú | 355330 | 3553302 | 22970.9 | 4513.8 | 3230.6 | 2525.5 | 1750.7 | 4713 |  |
| 3849SP | 35São Paulo | Tanabi | 355340 | 3553401 | 25161.8 | 4944.3 | 3538.8 | 2766.3 | 1917.7 | 9295 |  |
| 3850SP | 35São Paulo | Tapiraí | 355350 | 3553500 | 7980.6 | 1568.2 | 1122.4 | 877.4 | 608.2 | 2491 |  |
| 3851SP | 35São Paulo | Tapiratiba | 355360 | 3553609 | 12947.7 | 2544.2 | 1821 | 1423.5 | 986.8 | 6095 |  |
| 3852SP | 35São Paulo | Taquaral | 355365 | 3553658 | 2789.4 | 548.1 | 392.3 | 306.7 | 212.6 | 2725 |  |
| 3853SP | 35São Paulo | Taquaritinga | 355370 | 3553708 | 55947.5 | 10993.7 | 7868.5 | 6151 | 4263.9 | 12565 |  |
| 3854SP | 35São Paulo | Taquarituba | 355380 | 3553807 | 22915.3 | 4502.9 | 3222.8 | 2519.3 | 1746.4 | 5661 |  |
| 3855SP | 35São Paulo | Taquarivaí | 355385 | 3553856 | 5526.9 | 1086 | 777.3 | 607.6 | 421.2 | 1531 |  |
| 3856SP | 35São Paulo | Tarabai | 355390 | 3553906 | 7070.4 | 1389.3 | 994.4 | 777.3 | 538.9 | 2656 |  |
| 3857SP | 35São Paulo | Tarumã | 355395 | 3553955 | 13995.7 | 2750.2 | 1968.4 | 1538.7 | 1066.7 | 3872 |  |
| 3858SP | 35São Paulo | Tatuí | 355400 | 3554003 | 115071.6 | 22611.6 | 16183.7 | 12651.2 | 8769.9 | 29467 | 10 |
| 3859SP | 35São Paulo | Taubaté | 355410 | 3554102 | 298201.1 | 58596.5 | 41939 | 32784.8 | 22726.7 | 100566 | 33 |
| 3860SP | 35São Paulo | Tejupá | 355420 | 3554201 | 4717 | 926.9 | 663.4 | 518.6 | 359.5 | 1066 |  |
| 3861SP | 35São Paulo | Teodoro Sampaio | 355430 | 3554300 | 22399.9 | 4401.6 | 3150.3 | 2462.7 | 1707.2 | 7102 |  |
| 3862SP | 35São Paulo | Terra Roxa | 355440 | 3554409 | 8985.6 | 1765.7 | 1263.7 | 987.9 | 684.8 | 4259 |  |
| 3863SP | 35São Paulo | Tietê | 355450 | 3554508 | 39628.8 | 7787.1 | 5573.4 | 4356.9 | 3020.2 | 9445 |  |
| 3864SP | 35São Paulo | Timburi | 355460 | 3554607 | 2673.7 | 525.4 | 376 | 294 | 203.8 | 626 |  |
| 3865SP | 35São Paulo | Torre de Pedra | 355465 | 3554656 | 2347.4 | 461.3 | 330.1 | 258.1 | 178.9 | 709 |  |
| 3866SP | 35São Paulo | Torrinha | 355470 | 3554706 | 9730.1 | 1912 | 1368.4 | 1069.7 | 741.6 | 2970 |  |
| 3867SP | 35São Paulo | Trabiju | 355475 | 3554755 | 1642 | 322.7 | 230.9 | 180.5 | 125.1 | 739 |  |
| 3868SP | 35São Paulo | Tremembé | 355480 | 3554805 | 44268.3 | 8698.7 | 6225.9 | 4866.9 | 3373.8 | 9983 |  |
| 3869SP | 35São Paulo | Três Fronteiras | 355490 | 3554904 | 5652.3 | 1110.7 | 794.9 | 621.4 | 430.8 | 3752 |  |
| 3870SP | 35São Paulo | Tuiuti | 355495 | 3554953 | 6437 | 1264.9 | 905.3 | 707.7 | 490.6 | 2903 |  |
| 3871SP | 35São Paulo | Tupã | 355500 | 3555000 | 64971.2 | 12766.8 | 9137.5 | 7143 | 4951.6 | 28426 |  |
| 3872SP | 35São Paulo | Tupi Paulista | 355510 | 3555109 | 14968.4 | 2941.3 | 2105.2 | 1645.7 | 1140.8 | 11237 |  |
| 3873SP | 35São Paulo | Turiúba | 355520 | 3555208 | 1987 | 390.4 | 279.5 | 218.5 | 151.4 | 1178 |  |
| 3874SP | 35São Paulo | Turmalina | 355530 | 3555307 | 1875 | 368.4 | 263.7 | 206.1 | 142.9 | 1862 |  |
| 3875SP | 35São Paulo | Ubarana | 355535 | 3555356 | 5817.6 | 1143.2 | 818.2 | 639.6 | 443.4 | 2034 |  |
| 3876SP | 35São Paulo | Ubatuba | 355540 | 3555406 | 85151.3 | 16732.2 | 11975.7 | 9361.7 | 6489.6 | 13413 |  |
| 3877SP | 35São Paulo | Ubirajara | 355550 | 3555505 | 4630.8 | 910 | 651.3 | 509.1 | 352.9 | 1055 |  |
| 3878SP | 35São Paulo | Uchoa | 355560 | 3555604 | 9852.5 | 1936 | 1385.7 | 1083.2 | 750.9 | 4024 |  |
| 3879SP | 35São Paulo | União Paulista | 355570 | 3555703 | 1728.5 | 339.7 | 243.1 | 190 | 131.7 | 621 |  |
| 3880SP | 35São Paulo | Urânia | 355580 | 3555802 | 9040.6 | 1776.5 | 1271.5 | 993.9 | 689 | 6713 |  |
| 3881SP | 35São Paulo | Uru | 355590 | 3555901 | 1220.6 | 239.8 | 171.7 | 134.2 | 93 | 424 |  |
| 3882SP | 35São Paulo | Urupês | 355600 | 3556008 | 13339 | 2621.1 | 1876 | 1466.5 | 1016.6 | 4522 |  |
| 3883SP | 35São Paulo | Valentim Gentil | 355610 | 3556107 | 12215.9 | 2400.4 | 1718 | 1343 | 931 | 7647 |  |
| 3884SP | 35São Paulo | Valinhos | 355620 | 3556206 | 118318.5 | 23249.6 | 16640.3 | 13008.1 | 9017.4 | 20717 |  |
| 3885SP | 35São Paulo | Valparaíso | 355630 | 3556305 | 24617.7 | 4837.4 | 3462.2 | 2706.5 | 1876.2 | 8667 |  |
| 3886SP | 35São Paulo | Vargem | 355635 | 3556354 | 9698.5 | 1905.8 | 1364 | 1066.3 | 739.1 | 3074 |  |
| 3887SP | 35São Paulo | Vargem Grande do Sul | 355640 | 3556404 | 41289.6 | 8113.4 | 5807 | 4539.5 | 3146.8 | 6986 |  |
| 3888SP | 35São Paulo | Vargem Grande  Paulista | 355645 | 3556453 | 47914 | 9415.1 | 6738.6 | 5267.7 | 3651.7 | 9015 |  |
| 3889SP | 35São Paulo | Várzea Paulista | 355650 | 3556503 | 114979.5 | 22593.5 | 16170.7 | 12641 | 8762.9 | 31269 | 11 |
| 3890SP | 35São Paulo | Vera Cruz | 355660 | 3556602 | 10892.9 | 2140.5 | 1532 | 1197.6 | 830.2 | 3263 |  |
| 3891SP | 35São Paulo | Vinhedo | 355670 | 3556701 | 71319.9 | 14014.4 | 10030.4 | 7841 | 5435.5 | 12758 |  |
| 3892SP | 35São Paulo | Viradouro | 355680 | 3556800 | 18199.4 | 3576.2 | 2559.6 | 2000.9 | 1387 | 7681 |  |
| 3893SP | 35São Paulo | Vista Alegre do  Alto | 355690 | 3556909 | 7857 | 1543.9 | 1105 | 863.8 | 598.8 | 3358 |  |
| 3894SP | 35São Paulo | Vitória Brasil | 355695 | 3556958 | 1800.5 | 353.8 | 253.2 | 198 | 137.2 | 1536 |  |
| 3895SP | 35São Paulo | Votorantim | 355700 | 3557006 | 116201.4 | 22833.6 | 16342.6 | 12775.4 | 8856 | 25609 |  |
| 3896SP | 35São Paulo | Votuporanga | 355710 | 3557105 | 90072.2 | 17699.2 | 12667.8 | 9902.7 | 6864.7 | 48412 | 15 |
| 3897SP | 35São Paulo | Zacarias | 355715 | 3557154 | 2536.2 | 498.4 | 356.7 | 278.8 | 193.3 | 1137 |  |
| 3898SP | 35São Paulo | Chavantes | 355720 | 3557204 | 12357.9 | 2428.3 | 1738 | 1358.6 | 941.8 | 3847 |  |
| 3899SP | 35São Paulo | Estiva Gerbi | 355730 | 3557303 | 10725.7 | 2107.6 | 1508.5 | 1179.2 | 817.4 | 2551 |  |
| 3900PR | 41Paraná | Abatiá | 410010 | 4100103 | 7726.3 | 1498 | 1094.2 | 1082.9 | 794.7 | 2581 |  |
| 3901PR | 41Paraná | Adrianópolis | 410020 | 4100202 | 6255.7 | 1212.9 | 885.9 | 876.8 | 643.5 | 1498 |  |
| 3902PR | 41Paraná | Agudos do Sul | 410030 | 4100301 | 8859.6 | 1717.7 | 1254.7 | 1241.7 | 911.3 | 2367 |  |
| 3903PR | 41Paraná | Almirante  Tamandaré | 410040 | 4100400 | 111300.8 | 21579 | 15762.4 | 15599.5 | 11448.2 | 13132 |  |
| 3904PR | 41Paraná | Altamira do  Paraná | 410045 | 4100459 | 3318.6 | 643.4 | 470 | 465.1 | 341.3 | 1766 |  |
| 3905PR | 41Paraná | Altônia | 410050 | 4100509 | 21453.8 | 4159.5 | 3038.3 | 3006.9 | 2206.7 | 7173 |  |
| 3906PR | 41Paraná | Alto Paraná | 410060 | 4100608 | 14323.2 | 2777 | 2028.5 | 2007.5 | 1473.3 | 2193 |  |
| 3907PR | 41Paraná | Alto Piquiri | 410070 | 4100707 | 10156.4 | 1969.1 | 1438.3 | 1423.5 | 1044.7 | 3506 |  |
| 3908PR | 41Paraná | Alvorada do Sul | 410080 | 4100806 | 10907.2 | 2114.7 | 1544.7 | 1528.7 | 1121.9 | 3117 |  |
| 3909PR | 41Paraná | Amaporã | 410090 | 4100905 | 5870.2 | 1138.1 | 831.3 | 822.7 | 603.8 | 1434 |  |
| 3910PR | 41Paraná | Ampére | 410100 | 4101002 | 18338.6 | 3555.5 | 2597.1 | 2570.3 | 1886.3 | 6648 |  |
| 3911PR | 41Paraná | Anahy | 410105 | 4101051 | 2878.3 | 558 | 407.6 | 403.4 | 296.1 | 2961 |  |
| 3912PR | 41Paraná | Andirá | 410110 | 4101101 | 20614.6 | 3996.8 | 2919.4 | 2889.3 | 2120.4 | 5661 |  |
| 3913PR | 41Paraná | Ângulo | 410115 | 4101150 | 2925.6 | 567.2 | 414.3 | 410 | 300.9 | 857 |  |
| 3914PR | 41Paraná | Antonina | 410120 | 4101200 | 19168 | 3716.3 | 2714.6 | 2686.5 | 1971.6 | 3148 |  |
| 3915PR | 41Paraná | Antônio Olinto | 410130 | 4101309 | 7480.3 | 1450.3 | 1059.4 | 1048.4 | 769.4 | 1765 |  |
| 3916PR | 41Paraná | Apucarana | 410140 | 4101408 | 128647.6 | 24942.2 | 18219.1 | 18030.7 | 13232.5 | 37510 | 10 |
| 3917PR | 41Paraná | Arapongas | 410150 | 4101507 | 113782 | 22060.1 | 16113.8 | 15947.2 | 11703.5 | 46231 | 17 |
| 3918PR | 41Paraná | Arapoti | 410160 | 4101606 | 27176.7 | 5269 | 3848.8 | 3809 | 2795.4 | 3305 |  |
| 3919PR | 41Paraná | Arapuã | 410165 | 4101655 | 3387.5 | 656.8 | 479.7 | 474.8 | 348.4 | 2224 |  |
| 3920PR | 41Paraná | Araruna | 410170 | 4101705 | 13830.9 | 2681.5 | 1958.7 | 1938.5 | 1422.6 | 3004 |  |
| 3921PR | 41Paraná | Araucária | 410180 | 4101804 | 131519 | 25498.9 | 18625.7 | 18433.2 | 13527.9 | 35226 | 13 |
| 3922PR | 41Paraná | Ariranha do Ivaí | 410185 | 4101853 | 2332.1 | 452.1 | 330.3 | 326.9 | 239.9 | 1701 |  |

3

7

8

1

2

2

4

3

1

1

1

1

1

1

1

2

2

1

4

1

1

2

2

2

3

1

8

3

4

1

2

1

2

9

3

1

2

3

4

3

1

8

1

1

3

2

1

2

1

| 3923PR | 41Paraná | Assaí | 410190 | 4101903 | 16015.8 | 3105.1 | 2268.2 | 2244.7 | 1647.4 | 5476 |  |
| --- | --- | --- | --- | --- | --- | --- | --- | --- | --- | --- | --- |
| 3924PR | 41Paraná | Assis  Chateaubriand | 410200 | 4102000 | 33590.1 | 6512.4 | 4757 | 4707.8 | 3455 | 11907 |  |
| 3925PR | 41Paraná | Astorga | 410210 | 4102109 | 25632.4 | 4969.6 | 3630.1 | 3592.5 | 2636.5 | 7314 |  |
| 3926PR | 41Paraná | Atalaia | 410220 | 4102208 | 3953.2 | 766.4 | 559.9 | 554.1 | 406.6 | 935 |  |
| 3927PR | 41Paraná | Balsa Nova | 410230 | 4102307 | 12165.7 | 2358.7 | 1722.9 | 1705.1 | 1251.3 | 3058 |  |
| 3928PR | 41Paraná | Bandeirantes | 410240 | 4102406 | 32229.8 | 6248.7 | 4564.4 | 4517.2 | 3315.1 | 4155 |  |
| 3929PR | 41Paraná | Barbosa Ferraz | 410250 | 4102505 | 12336.5 | 2391.8 | 1747.1 | 1729 | 1268.9 | 3263 |  |
| 3930PR | 41Paraná | Barracão | 410260 | 4102604 | 10095.8 | 1957.4 | 1429.8 | 1415 | 1038.4 | 3900 |  |
| 3931PR | 41Paraná | Barra do Jacaré | 410270 | 4102703 | 2784.8 | 539.9 | 394.4 | 390.3 | 286.4 | 1880 |  |
| 3932PR | 41Paraná | Bela Vista da  Caroba | 410275 | 4102752 | 3803.2 | 737.4 | 538.6 | 533 | 391.2 | 1622 |  |
| 3933PR | 41Paraná | Bela Vista do  Paraíso | 410280 | 4102802 | 15409.7 | 2987.6 | 2182.3 | 2159.8 | 1585 | 4630 |  |
| 3934PR | 41Paraná | Bituruna | 410290 | 4102901 | 16292.2 | 3158.7 | 2307.3 | 2283.4 | 1675.8 | 3749 |  |
| 3935PR | 41Paraná | Boa Esperança | 410300 | 4103008 | 4422.4 | 857.4 | 626.3 | 619.8 | 454.9 | 2417 |  |
| 3936PR | 41Paraná | Boa Esperança do  Iguaçu | 410302 | 4103024 | 2683.9 | 520.4 | 380.1 | 376.2 | 276.1 | 840 |  |
| 3937PR | 41Paraná | Boa Ventura de  São Roque | 410304 | 4103040 | 6582.5 | 1276.2 | 932.2 | 922.6 | 677.1 | 1564 |  |
| 3938PR | 41Paraná | Boa Vista da  Aparecida | 410305 | 4103057 | 7869.5 | 1525.7 | 1114.5 | 1103 | 809.4 | 4013 |  |
| 3939PR | 41Paraná | Bocaiúva do Sul | 410310 | 4103107 | 11988 | 2324.2 | 1697.7 | 1680.2 | 1233.1 | 2022 |  |
| 3940PR | 41Paraná | Bom Jesus do Sul | 410315 | 4103156 | 3731.4 | 723.4 | 528.4 | 523 | 383.8 | 2237 |  |
| 3941PR | 41Paraná | Bom Sucesso | 410320 | 4103206 | 6851.6 | 1328.4 | 970.3 | 960.3 | 704.7 | 2818 |  |
| 3942PR | 41Paraná | Bom Sucesso do  Sul | 410322 | 4103222 | 3321.9 | 644 | 470.4 | 465.6 | 341.7 | 2445 |  |
| 3943PR | 41Paraná | Borrazópolis | 410330 | 4103305 | 7411.3 | 1436.9 | 1049.6 | 1038.7 | 762.3 | 3873 |  |
| 3944PR | 41Paraná | Braganey | 410335 | 4103354 | 5671.4 | 1099.6 | 803.2 | 794.9 | 583.4 | 2531 |  |
| 3945PR | 41Paraná | Brasilândia do Sul | 410337 | 4103370 | 3003.5 | 582.3 | 425.4 | 421 | 308.9 | 1784 |  |
| 3946PR | 41Paraná | Cafeara | 410340 | 4103404 | 2834.3 | 549.5 | 401.4 | 397.2 | 291.5 | 1531 |  |
| 3947PR | 41Paraná | Cafelândia | 410345 | 4103453 | 16370.6 | 3173.9 | 2318.4 | 2294.4 | 1683.9 | 7045 |  |
| 3948PR | 41Paraná | Cafezal do Sul | 410347 | 4103479 | 4235.6 | 821.2 | 599.8 | 593.6 | 435.7 | 2121 |  |
| 3949PR | 41Paraná | Califórnia | 410350 | 4103503 | 8392 | 1627 | 1188.5 | 1176.2 | 863.2 | 2966 |  |
| 3950PR | 41Paraná | Cambará | 410360 | 4103602 | 24836.9 | 4815.4 | 3517.4 | 3481 | 2554.7 | 6029 |  |
| 3951PR | 41Paraná | Cambé | 410370 | 4103701 | 102340.2 | 19841.7 | 14493.4 | 14343.6 | 10526.6 | 45115 | 14 |
| 3952PR | 41Paraná | Cambira | 410380 | 4103800 | 7604.6 | 1474.4 | 1077 | 1065.8 | 782.2 | 2205 |  |
| 3953PR | 41Paraná | Campina da  Lagoa | 410390 | 4103909 | 15062.9 | 2920.4 | 2133.2 | 2111.2 | 1549.3 | 5319 |  |
| 3954PR | 41Paraná | Campina do  Simão | 410395 | 4103958 | 4044.7 | 784.2 | 572.8 | 566.9 | 416 | 1233 |  |
| 3955PR | 41Paraná | Campina Grande do Sul | 410400 | 4104006 | 41249.5 | 7997.5 | 5841.8 | 5781.4 | 4242.9 | 8515 |  |
| 3956PR | 41Paraná | Campo Bonito | 410405 | 4104055 | 4209.7 | 816.2 | 596.2 | 590 | 433 | 1907 |  |
| 3957PR | 41Paraná | Campo do  Tenente | 410410 | 4104105 | 7587.8 | 1471.1 | 1074.6 | 1063.5 | 780.5 | 981 |  |
| 3958PR | 41Paraná | Campo Largo | 410420 | 4104204 | 122398.6 | 23730.6 | 17334.1 | 17154.9 | 12589.7 | 30384 |  |
| 3959PR | 41Paraná | Campo Magro | 410425 | 4104253 | 27128.5 | 5259.7 | 3841.9 | 3802.2 | 2790.4 | 6034 |  |
| 3960PR | 41Paraná | Campo Mourão | 410430 | 4104303 | 91676.2 | 17774.2 | 12983.2 | 12849 | 9429.7 | 22625 |  |
| 3961PR | 41Paraná | Cândido de Abreu | 410440 | 4104402 | 16144.2 | 3130 | 2286.3 | 2262.7 | 1660.6 | 4012 |  |
| 3962PR | 41Paraná | Candói | 410442 | 4104428 | 15611.2 | 3026.7 | 2210.9 | 2188 | 1605.7 | 2419 |  |
| 3963PR | 41Paraná | Cantagalo | 410445 | 4104451 | 13276.7 | 2574.1 | 1880.2 | 1860.8 | 1365.6 | 2311 |  |
| 3964PR | 41Paraná | Capanema | 410450 | 4104501 | 19023.7 | 3688.3 | 2694.1 | 2666.3 | 1956.8 | 9872 |  |
| 3965PR | 41Paraná | Capitão Leônidas  Marques | 410460 | 4104600 | 15516.3 | 3008.3 | 2197.4 | 2174.7 | 1596 | 5289 |  |
| 3966PR | 41Paraná | Carambeí | 410465 | 4104659 | 21278.4 | 4125.5 | 3013.4 | 2982.3 | 2188.7 | 2608 |  |
| 3967PR | 41Paraná | Carlópolis | 410470 | 4104709 | 14149.3 | 2743.3 | 2003.8 | 1983.1 | 1455.4 | 3346 |  |
| 3968PR | 41Paraná | Cascavel | 410480 | 4104808 | 308433.2 | 59799 | 43680.3 | 43228.7 | 31725 | 145272 | 52 |
| 3969PR | 41Paraná | Castro | 410490 | 4104907 | 69869.5 | 13546.3 | 9894.9 | 9792.6 | 7186.7 | 9291 |  |
| 3970PR | 41Paraná | Catanduvas | 410500 | 4105003 | 10325.3 | 2001.9 | 1462.3 | 1447.2 | 1062 | 3498 |  |
| 3971PR | 41Paraná | Centenário do Sul | 410510 | 4105102 | 11171.1 | 2165.9 | 1582.1 | 1565.7 | 1149 | 3756 |  |
| 3972PR | 41Paraná | Cerro Azul | 410520 | 4105201 | 17522.1 | 3397.2 | 2481.5 | 2455.8 | 1802.3 | 2144 |  |
| 3973PR | 41Paraná | Céu Azul | 410530 | 4105300 | 11494.1 | 2228.5 | 1627.8 | 1611 | 1182.3 | 4827 |  |
| 3974PR | 41Paraná | Chopinzinho | 410540 | 4105409 | 19740.3 | 3827.2 | 2795.6 | 2766.7 | 2030.5 | 5953 |  |
| 3975PR | 41Paraná | Cianorte | 410550 | 4105508 | 76420.3 | 14816.4 | 10822.6 | 10710.8 | 7860.5 | 29682 | 10 |
| 3976PR | 41Paraná | Cidade Gaúcha | 410560 | 4105607 | 11902.2 | 2307.6 | 1685.6 | 1668.2 | 1224.2 | 3450 |  |
| 3977PR | 41Paraná | Clevelândia | 410570 | 4105706 | 17157.2 | 3326.4 | 2429.8 | 2404.7 | 1764.8 | 3313 |  |
| 3978PR | 41Paraná | Colombo | 410580 | 4105805 | 229208.5 | 44438.9 | 32460.5 | 32124.9 | 23576.1 | 62433 | 24 |
| 3979PR | 41Paraná | Colorado | 410590 | 4105904 | 23361.7 | 4529.4 | 3308.5 | 3274.3 | 2403 | 5429 |  |
| 3980PR | 41Paraná | Congonhinhas | 410600 | 4106001 | 8620.7 | 1671.4 | 1220.9 | 1208.2 | 886.7 | 2370 |  |
| 3981PR | 41Paraná | Conselheiro  Mairinck | 410610 | 4106100 | 3780.4 | 732.9 | 535.4 | 529.8 | 388.8 | 1239 |  |
| 3982PR | 41Paraná | Contenda | 410620 | 4106209 | 17278.5 | 3350 | 2447 | 2421.7 | 1777.2 | 4514 |  |
| 3983PR | 41Paraná | Corbélia | 410630 | 4106308 | 16852.1 | 3267.3 | 2386.6 | 2361.9 | 1733.4 | 9378 |  |
| 3984PR | 41Paraná | Cornélio Procópio | 410640 | 4106407 | 47924.2 | 9291.5 | 6787 | 6716.9 | 4929.4 | 13014 |  |
| 3985PR | 41Paraná | Coronel Domingos  Soares | 410645 | 4106456 | 7455.2 | 1445.4 | 1055.8 | 1044.9 | 766.8 | 1909 |  |
| 3986PR | 41Paraná | Coronel Vivida | 410650 | 4106506 | 21575.9 | 4183.1 | 3055.6 | 3024 | 2219.3 | 7258 |  |
| 3987PR | 41Paraná | Corumbataí do Sul | 410655 | 4106555 | 3707.6 | 718.8 | 525.1 | 519.6 | 381.4 | 1550 |  |
| 3988PR | 41Paraná | Cruzeiro do  Iguaçu | 410657 | 4106571 | 4320.5 | 837.7 | 611.9 | 605.5 | 444.4 | 1596 |  |
| 3989PR | 41Paraná | Cruzeiro do Oeste | 410660 | 4106605 | 20902.2 | 4052.5 | 2960.2 | 2929.6 | 2150 | 8011 |  |
| 3990PR | 41Paraná | Cruzeiro do Sul | 410670 | 4106704 | 4578.9 | 887.8 | 648.5 | 641.8 | 471 | 845 |  |
| 3991PR | 41Paraná | Cruz Machado | 410680 | 4106803 | 18561.8 | 3598.8 | 2628.7 | 2601.5 | 1909.2 | 2643 |  |
| 3992PR | 41Paraná | Cruzmaltina | 410685 | 4106852 | 3108.5 | 602.7 | 440.2 | 435.7 | 319.7 | 1906 |  |
| 3993PR | 41Paraná | Curitiba | 410690 | 4106902 | 1853857.1 | 359425.8 | 262543.2 | 259828.9 | 190685.2 | 489596 | 129 |
| 3994PR | 41Paraná | Curiúva | 410700 | 4107009 | 14617.7 | 2834.1 | 2070.2 | 2048.8 | 1503.6 | 3396 |  |
| 3995PR | 41Paraná | Diamante do Norte | 410710 | 4107108 | 5396.6 | 1046.3 | 764.3 | 756.4 | 555.1 | 1740 |  |
| 3996PR | 41Paraná | Diamante do Sul | 410712 | 4107124 | 3523.3 | 683.1 | 499 | 493.8 | 362.4 | 1341 |  |
| 3997PR | 41Paraná | Diamante D'Oeste | 410715 | 4107157 | 5190.2 | 1006.3 | 735 | 727.4 | 533.9 | 1948 |  |
| 3998PR | 41Paraná | Dois Vizinhos | 410720 | 4107207 | 38601.5 | 7484.1 | 5466.7 | 5410.2 | 3970.5 | 12130 |  |
| 3999PR | 41Paraná | Douradina | 410725 | 4107256 | 8112.6 | 1572.9 | 1148.9 | 1137 | 834.5 | 3023 |  |
| 4000PR | 41Paraná | Doutor Camargo | 410730 | 4107306 | 5968.5 | 1157.2 | 845.3 | 836.5 | 613.9 | 1537 |  |
| 4001PR | 41Paraná | Enéas Marques | 410740 | 4107405 | 6117.4 | 1186 | 866.3 | 857.4 | 629.2 | 3288 |  |
| 4002PR | 41Paraná | Engenheiro  Beltrão | 410750 | 4107504 | 14123.1 | 2738.2 | 2000.1 | 1979.4 | 1452.7 | 4945 |  |
| 4003PR | 41Paraná | Esperança Nova | 410752 | 4107520 | 1875.6 | 363.6 | 265.6 | 262.9 | 192.9 | 1158 |  |
| 4004PR | 41Paraná | Entre Rios do  Oeste | 410753 | 4107538 | 4246.1 | 823.2 | 601.3 | 595.1 | 436.7 | 2293 |  |
| 4005PR | 41Paraná | Espigão Alto do  Iguaçu | 410754 | 4107546 | 4489 | 870.3 | 635.7 | 629.2 | 461.7 | 1713 |  |
| 4006PR | 41Paraná | Farol | 410755 | 4107553 | 3347.6 | 649 | 474.1 | 469.2 | 344.3 | 894 |  |
| 4007PR | 41Paraná | Faxinal | 410760 | 4107603 | 16932.8 | 3282.9 | 2398 | 2373.2 | 1741.7 | 3665 |  |
| 4008PR | 41Paraná | Fazenda Rio  Grande | 410765 | 4107652 | 90872.8 | 17618.4 | 12869.4 | 12736.4 | 9347 | 17225 |  |
| 4009PR | 41Paraná | Fênix | 410770 | 4107702 | 4845.1 | 939.4 | 686.2 | 679.1 | 498.4 | 1407 |  |
| 4010PR | 41Paraná | Fernandes  Pinheiro | 410773 | 4107736 | 5881 | 1140.2 | 832.9 | 824.3 | 604.9 | 2221 |  |
| 4011PR | 41Paraná | Figueira | 410775 | 4107751 | 8167.1 | 1583.4 | 1156.6 | 1144.7 | 840.1 | 2519 |  |
| 4012PR | 41Paraná | Floraí | 410780 | 4107801 | 5060.8 | 981.2 | 716.7 | 709.3 | 520.5 | 2128 |  |
| 4013PR | 41Paraná | Flor da Serra do  Sul | 410785 | 4107850 | 4741 | 919.2 | 671.4 | 664.5 | 487.7 | 3697 |  |
| 4014PR | 41Paraná | Floresta | 410790 | 4107900 | 6377.3 | 1236.4 | 903.2 | 893.8 | 656 | 1776 |  |
| 4015PR | 41Paraná | Florestópolis | 410800 | 4108007 | 11067 | 2145.7 | 1567.3 | 1551.1 | 1138.3 | 3765 |  |
| 4016PR | 41Paraná | Flórida | 410810 | 4108106 | 2639 | 511.6 | 373.7 | 369.9 | 271.4 | 874 |  |

1

3

1

1

1

1

1

1

1

1

2

1

1

3

9

2

5

1

3

1

1

2

1

1

1

2

1

1

1

3

4

2

2

1

4

1

1

1

5

1

1

| 4017PR | 41Paraná | Formosa do Oeste | 410820 | 4108205 | 7211.7 | 1398.2 | 1021.3 | 1010.8 | 741.8 | 5561 |  |
| --- | --- | --- | --- | --- | --- | --- | --- | --- | --- | --- | --- |
| 4018PR | 41Paraná | Foz do Iguaçu | 410830 | 4108304 | 260395.7 | 50485.5 | 36877.2 | 36496 | 26783.9 | 69450 | 26 |
| 4019PR | 41Paraná | Francisco Alves | 410832 | 4108320 | 6335.7 | 1228.4 | 897.3 | 888 | 651.7 | 2906 |  |
| 4020PR | 41Paraná | Francisco Beltrão | 410840 | 4108403 | 85293.3 | 16536.7 | 12079.2 | 11954.4 | 8773.1 | 36792 | 13 |
| 4021PR | 41Paraná | Foz do Jordão | 410845 | 4108452 | 5149.3 | 998.3 | 729.2 | 721.7 | 529.6 | 1169 |  |
| 4022PR | 41Paraná | General Carneiro | 410850 | 4108502 | 13859.3 | 2687 | 1962.8 | 1942.5 | 1425.5 | 1071 |  |
| 4023PR | 41Paraná | Godoy Moreira | 410855 | 4108551 | 3206.8 | 621.7 | 454.1 | 449.5 | 329.8 | 2929 |  |
| 4024PR | 41Paraná | Goioerê | 410860 | 4108601 | 29324.8 | 5685.5 | 4153 | 4110 | 3016.3 | 10656 |  |
| 4025PR | 41Paraná | Goioxim | 410865 | 4108650 | 7424.7 | 1439.5 | 1051.5 | 1040.6 | 763.7 | 1410 |  |
| 4026PR | 41Paraná | Grandes Rios | 410870 | 4108700 | 6264.8 | 1214.6 | 887.2 | 878 | 644.4 | 1837 |  |
| 4027PR | 41Paraná | Guaíra | 410880 | 4108809 | 32155.3 | 6234.3 | 4553.8 | 4506.8 | 3307.4 | 9216 |  |
| 4028PR | 41Paraná | Guairaçá | 410890 | 4108908 | 6444.6 | 1249.5 | 912.7 | 903.2 | 662.9 | 2244 |  |
| 4029PR | 41Paraná | Guamiranga | 410895 | 4108957 | 8369.1 | 1622.6 | 1185.2 | 1173 | 860.8 | 2233 |  |
| 4030PR | 41Paraná | Guapirama | 410900 | 4109005 | 3900.8 | 756.3 | 552.4 | 546.7 | 401.2 | 1795 |  |
| 4031PR | 41Paraná | Guaporema | 410910 | 4109104 | 2260 | 438.2 | 320.1 | 316.8 | 232.5 | 933 |  |
| 4032PR | 41Paraná | Guaraci | 410920 | 4109203 | 5380 | 1043.1 | 761.9 | 754 | 553.4 | 2143 |  |
| 4033PR | 41Paraná | Guaraniaçu | 410930 | 4109302 | 13836.4 | 2682.6 | 1959.5 | 1939.3 | 1423.2 | 6764 |  |
| 4034PR | 41Paraná | Guarapuava | 410940 | 4109401 | 175717.8 | 34068.2 | 24885.2 | 24627.9 | 18074.1 | 32574 | 10 |
| 4035PR | 41Paraná | Guaraqueçaba | 410950 | 4109500 | 7866.7 | 1525.2 | 1114.1 | 1102.6 | 809.2 | 953 |  |
| 4036PR | 41Paraná | Guaratuba | 410960 | 4109609 | 34691 | 6725.9 | 4912.9 | 4862.1 | 3568.3 | 6012 |  |
| 4037PR | 41Paraná | Honório Serpa | 410965 | 4109658 | 5702 | 1105.5 | 807.5 | 799.2 | 586.5 | 1854 |  |
| 4038PR | 41Paraná | Ibaiti | 410970 | 4109708 | 30265.1 | 5867.8 | 4286.1 | 4241.8 | 3113 | 7835 |  |
| 4039PR | 41Paraná | Ibema | 410975 | 4109757 | 6268.6 | 1215.4 | 887.8 | 878.6 | 644.8 | 2363 |  |
| 4040PR | 41Paraná | Ibiporã | 410980 | 4109807 | 51609 | 10006 | 7308.9 | 7233.3 | 5308.4 | 16953 |  |
| 4041PR | 41Paraná | Icaraíma | 410990 | 4109906 | 8538.7 | 1655.5 | 1209.3 | 1196.7 | 878.3 | 3854 |  |
| 4042PR | 41Paraná | Iguaraçu | 411000 | 4110003 | 4217.6 | 817.7 | 597.3 | 591.1 | 433.8 | 1255 |  |
| 4043PR | 41Paraná | Iguatu | 411005 | 4110052 | 2271.9 | 440.5 | 321.7 | 318.4 | 233.7 | 1504 |  |
| 4044PR | 41Paraná | Imbaú | 411007 | 4110078 | 12225.9 | 2370.4 | 1731.4 | 1713.5 | 1257.5 | 3847 |  |
| 4045PR | 41Paraná | Imbituva | 411010 | 4110102 | 30624.7 | 5937.5 | 4337.1 | 4292.2 | 3150 | 9242 |  |
| 4046PR | 41Paraná | Inácio Martins | 411020 | 4110201 | 11161.1 | 2163.9 | 1580.6 | 1564.3 | 1148 | 2384 |  |
| 4047PR | 41Paraná | Inajá | 411030 | 4110300 | 3078.8 | 596.9 | 436 | 431.5 | 316.7 | 788 |  |
| 4048PR | 41Paraná | Indianópolis | 411040 | 4110409 | 4422.7 | 857.5 | 626.3 | 619.9 | 454.9 | 2131 |  |
| 4049PR | 41Paraná | Ipiranga | 411050 | 4110508 | 14778.7 | 2865.3 | 2093 | 2071.3 | 1520.1 | 2099 |  |
| 4050PR | 41Paraná | Iporã | 411060 | 4110607 | 14705.3 | 2851.1 | 2082.6 | 2061 | 1512.6 | 4619 |  |
| 4051PR | 41Paraná | Iracema do Oeste | 411065 | 4110656 | 2482.4 | 481.3 | 351.6 | 347.9 | 255.3 | 1535 |  |
| 4052PR | 41Paraná | Irati | 411070 | 4110706 | 58908.3 | 11421.1 | 8342.6 | 8256.3 | 6059.2 | 14490 |  |
| 4053PR | 41Paraná | Iretama | 411080 | 4110805 | 10556.2 | 2046.6 | 1495 | 1479.5 | 1085.8 | 2427 |  |
| 4054PR | 41Paraná | Itaguajé | 411090 | 4110904 | 4580.7 | 888.1 | 648.7 | 642 | 471.2 | 967 |  |
| 4055PR | 41Paraná | Itaipulândia | 411095 | 4110953 | 10087.5 | 1955.8 | 1428.6 | 1413.8 | 1037.6 | 5005 |  |
| 4056PR | 41Paraná | Itambaracá | 411100 | 4111001 | 6766.3 | 1311.9 | 958.2 | 948.3 | 696 | 2029 |  |
| 4057PR | 41Paraná | Itambé | 411110 | 4111100 | 6111.7 | 1184.9 | 865.5 | 856.6 | 628.6 | 2156 |  |
| 4058PR | 41Paraná | Itapejara d'Oeste | 411120 | 4111209 | 11295.6 | 2190 | 1599.7 | 1583.1 | 1161.8 | 3504 |  |
| 4059PR | 41Paraná | Itaperuçu | 411125 | 4111258 | 26324.8 | 5103.9 | 3728.1 | 3689.6 | 2707.7 | 3284 |  |
| 4060PR | 41Paraná | Itaúna do Sul | 411130 | 4111308 | 3312.2 | 642.2 | 469.1 | 464.2 | 340.7 | 889 |  |
| 4061PR | 41Paraná | Ivaí | 411140 | 4111407 | 13445.5 | 2606.8 | 1904.2 | 1884.5 | 1383 | 1527 |  |
| 4062PR | 41Paraná | Ivaiporã | 411150 | 4111506 | 32291.3 | 6260.6 | 4573.1 | 4525.8 | 3321.4 | 14246 |  |
| 4063PR | 41Paraná | Ivaté | 411155 | 4111555 | 7904.8 | 1532.6 | 1119.5 | 1107.9 | 813.1 | 2062 |  |
| 4064PR | 41Paraná | Ivatuba | 411160 | 4111605 | 3157.6 | 612.2 | 447.2 | 442.6 | 324.8 | 1210 |  |
| 4065PR | 41Paraná | Jaboti | 411170 | 4111704 | 5127.5 | 994.1 | 726.2 | 718.6 | 527.4 | 1966 |  |
| 4066PR | 41Paraná | Jacarezinho | 411180 | 4111803 | 39727.3 | 7702.3 | 5626.2 | 5568 | 4086.3 | 10652 |  |
| 4067PR | 41Paraná | Jaguapitã | 411190 | 4111902 | 12994.1 | 2519.3 | 1840.2 | 1821.2 | 1336.6 | 4397 |  |
| 4068PR | 41Paraná | Jaguariaíva | 411200 | 4112009 | 34009.2 | 6593.7 | 4816.4 | 4766.6 | 3498.1 | 4078 |  |
| 4069PR | 41Paraná | Jandaia do Sul | 411210 | 4112108 | 20924.6 | 4056.9 | 2963.3 | 2932.7 | 2152.3 | 9510 |  |
| 4070PR | 41Paraná | Janiópolis | 411220 | 4112207 | 6047.1 | 1172.4 | 856.4 | 847.5 | 622 | 2451 |  |
| 4071PR | 41Paraná | Japira | 411230 | 4112306 | 5005.5 | 970.5 | 708.9 | 701.6 | 514.9 | 1930 |  |
| 4072PR | 41Paraná | Japurá | 411240 | 4112405 | 9042.9 | 1753.2 | 1280.7 | 1267.4 | 930.1 | 4692 |  |
| 4073PR | 41Paraná | Jardim Alegre | 411250 | 4112504 | 12042.3 | 2334.8 | 1705.4 | 1687.8 | 1238.7 | 5413 |  |
| 4074PR | 41Paraná | Jardim Olinda | 411260 | 4112603 | 1392.3 | 269.9 | 197.2 | 195.1 | 143.2 | 414 |  |
| 4075PR | 41Paraná | Jataizinho | 411270 | 4112702 | 12338.7 | 2392.2 | 1747.4 | 1729.3 | 1269.1 | 3944 |  |
| 4076PR | 41Paraná | Jesuítas | 411275 | 4112751 | 8854.7 | 1716.7 | 1254 | 1241 | 910.8 | 5480 |  |
| 4077PR | 41Paraná | Joaquim Távora | 411280 | 4112801 | 11387.3 | 2207.8 | 1612.7 | 1596 | 1171.3 | 3874 |  |
| 4078PR | 41Paraná | Jundiaí do Sul | 411290 | 4112900 | 3413.6 | 661.8 | 483.4 | 478.4 | 351.1 | 1281 |  |
| 4079PR | 41Paraná | Juranda | 411295 | 4112959 | 7603.9 | 1474.2 | 1076.9 | 1065.7 | 782.1 | 2886 |  |
| 4080PR | 41Paraná | Jussara | 411300 | 4113007 | 6870.9 | 1332.1 | 973.1 | 963 | 706.7 | 2577 |  |
| 4081PR | 41Paraná | Kaloré | 411310 | 4113106 | 4384.2 | 850 | 620.9 | 614.5 | 451 | 2680 |  |
| 4082PR | 41Paraná | Lapa | 411320 | 4113205 | 46922.9 | 9097.4 | 6645.2 | 6576.5 | 4826.4 | 8672 |  |
| 4083PR | 41Paraná | Laranjal | 411325 | 4113254 | 6216.3 | 1205.2 | 880.4 | 871.3 | 639.4 | 679 |  |
| 4084PR | 41Paraná | Laranjeiras do Sul | 411330 | 4113304 | 31729.4 | 6151.7 | 4493.5 | 4447.1 | 3263.6 | 10030 |  |
| 4085PR | 41Paraná | Leópolis | 411340 | 4113403 | 4113.1 | 797.4 | 582.5 | 576.5 | 423.1 | 2160 |  |
| 4086PR | 41Paraná | Lidianópolis | 411342 | 4113429 | 3731.1 | 723.4 | 528.4 | 522.9 | 383.8 | 2507 |  |
| 4087PR | 41Paraná | Lindoeste | 411345 | 4113452 | 5126.8 | 994 | 726.1 | 718.6 | 527.3 | 3235 |  |
| 4088PR | 41Paraná | Loanda | 411350 | 4113502 | 22298.9 | 4323.3 | 3158 | 3125.3 | 2293.6 | 4291 |  |
| 4089PR | 41Paraná | Lobato | 411360 | 4113601 | 4626.8 | 897 | 655.2 | 648.5 | 475.9 | 1174 |  |
| 4090PR | 41Paraná | Londrina | 411370 | 4113700 | 540801.2 | 104850.5 | 76588.3 | 75796.5 | 55626.1 | 202865 | 65 |
| 4091PR | 41Paraná | Luiziana | 411373 | 4113734 | 7379.7 | 1430.8 | 1045.1 | 1034.3 | 759.1 | 1663 |  |
| 4092PR | 41Paraná | Lunardelli | 411375 | 4113759 | 5064.1 | 981.8 | 717.2 | 709.8 | 520.9 | 2927 |  |
| 4093PR | 41Paraná | Lupionópolis | 411380 | 4113809 | 4794.4 | 929.5 | 679 | 672 | 493.1 | 2415 |  |
| 4094PR | 41Paraná | Mallet | 411390 | 4113908 | 13414.2 | 2600.7 | 1899.7 | 1880.1 | 1379.8 | 2632 |  |
| 4095PR | 41Paraná | Mamborê | 411400 | 4114005 | 13771.8 | 2670.1 | 1950.4 | 1930.2 | 1416.5 | 4856 |  |
| 4096PR | 41Paraná | Mandaguaçu | 411410 | 4114104 | 21369.7 | 4143.2 | 3026.4 | 2995.1 | 2198.1 | 4459 |  |
| 4097PR | 41Paraná | Mandaguari | 411420 | 4114203 | 33837.2 | 6560.4 | 4792 | 4742.5 | 3480.4 | 10616 |  |
| 4098PR | 41Paraná | Mandirituba | 411430 | 4114302 | 24548.8 | 4759.5 | 3476.6 | 3440.7 | 2525.1 | 4423 |  |
| 4099PR | 41Paraná | Manfrinópolis | 411435 | 4114351 | 2921.6 | 566.4 | 413.8 | 409.5 | 300.5 | 1403 |  |
| 4100PR | 41Paraná | Mangueirinha | 411440 | 4114401 | 17115.6 | 3318.4 | 2423.9 | 2398.9 | 1760.5 | 3739 |  |
| 4101PR | 41Paraná | Manoel Ribas | 411450 | 4114500 | 13482.4 | 2614 | 1909.4 | 1889.6 | 1386.8 | 4036 |  |
| 4102PR | 41Paraná | Marechal Cândido  Rondon | 411460 | 4114609 | 50108.5 | 9715 | 7096.4 | 7023 | 5154.1 | 10244 |  |
| 4103PR | 41Paraná | Maria Helena | 411470 | 4114708 | 5908 | 1145.4 | 836.7 | 828 | 607.7 | 1619 |  |
| 4104PR | 41Paraná | Marialva | 411480 | 4114807 | 33920.3 | 6576.5 | 4803.8 | 4754.1 | 3489 | 11258 |  |
| 4105PR | 41Paraná | Marilândia do Sul | 411490 | 4114906 | 8962.6 | 1737.7 | 1269.3 | 1256.2 | 921.9 | 2944 |  |
| 4106PR | 41Paraná | Marilena | 411500 | 4115002 | 7040.8 | 1365.1 | 997.1 | 986.8 | 724.2 | 1893 |  |
| 4107PR | 41Paraná | Mariluz | 411510 | 4115101 | 10405.9 | 2017.5 | 1473.7 | 1458.4 | 1070.3 | 4314 |  |
| 4108PR | 41Paraná | Maringá | 411520 | 4115200 | 391213.6 | 75848.5 | 55403.7 | 54830.9 | 40239.7 | 97988 | 21 |
| 4109PR | 41Paraná | Mariópolis | 411530 | 4115309 | 6497.9 | 1259.8 | 920.2 | 910.7 | 668.4 | 2081 |  |
| 4110PR | 41Paraná | Maripá | 411535 | 4115358 | 5719.8 | 1109 | 810 | 801.7 | 588.3 | 2985 |  |
| 4111PR | 41Paraná | Marmeleiro | 411540 | 4115408 | 14281.2 | 2768.8 | 2022.5 | 2001.6 | 1468.9 | 5780 |  |
| 4112PR | 41Paraná | Marquinho | 411545 | 4115457 | 4797 | 930 | 679.4 | 672.3 | 493.4 | 1669 |  |
| 4113PR | 41Paraná | Marumbi | 411550 | 4115507 | 4694.2 | 910.1 | 664.8 | 657.9 | 482.8 | 1524 |  |
| 4114PR | 41Paraná | Matelândia | 411560 | 4115606 | 17103 | 3315.9 | 2422.1 | 2397.1 | 1759.2 | 10876 |  |
| 4115PR | 41Paraná | Matinhos | 411570 | 4115705 | 32130.9 | 6229.5 | 4550.4 | 4503.3 | 3304.9 | 6058 |  |
| 4116PR | 41Paraná | Mato Rico | 411573 | 4115739 | 3625.9 | 703 | 513.5 | 508.2 | 373 | 1219 |  |
| 4117PR | 41Paraná | Mauá da Serra | 411575 | 4115754 | 9564.5 | 1854.4 | 1354.5 | 1340.5 | 983.8 | 2214 |  |
| 4118PR | 41Paraná | Medianeira | 411580 | 4115804 | 44275.2 | 8584.1 | 6270.3 | 6205.4 | 4554.1 | 16068 |  |
| 4119PR | 41Paraná | Mercedes | 411585 | 4115853 | 5325.2 | 1032.4 | 754.2 | 746.4 | 547.7 | 2200 |  |
| 4120PR | 41Paraná | Mirador | 411590 | 4115903 | 2305.8 | 447 | 326.5 | 323.2 | 237.2 | 1051 |  |
| 4121PR | 41Paraná | Miraselva | 411600 | 4116000 | 1861.2 | 360.8 | 263.6 | 260.9 | 191.4 | 1053 |  |
| 4122PR | 41Paraná | Missal | 411605 | 4116059 | 10706.6 | 2075.8 | 1516.3 | 1500.6 | 1101.3 | 5993 |  |
| 4123PR | 41Paraná | Moreira Sales | 411610 | 4116109 | 12550.8 | 2433.3 | 1777.4 | 1759.1 | 1291 | 4030 |  |
| 4124PR | 41Paraná | Morretes | 411620 | 4116208 | 16219.6 | 3144.7 | 2297 | 2273.3 | 1668.3 | 2145 |  |
| 4125PR | 41Paraná | Munhoz de Melo | 411630 | 4116307 | 3856.1 | 747.6 | 546.1 | 540.5 | 396.6 | 1888 |  |
| 4126PR | 41Paraná | Nossa Senhora das Graças | 411640 | 4116406 | 3993.9 | 774.3 | 565.6 | 559.8 | 410.8 | 1295 |  |

1

3

3

2

1

2

4

1

3

1

3

1

1

1

4

3

1

3

1

1

1

1

1

2

3

1

1

1

1

3

1

1

1

3

3

1

1

2

3

1

5

1

1

| 4127PR | 41Paraná | Nova Aliança do  Ivaí | 411650 | 4116505 | 1498.2 | 290.5 | 212.2 | 210 | 154.1 | 599 |  |
| --- | --- | --- | --- | --- | --- | --- | --- | --- | --- | --- | --- |
| 4128PR | 41Paraná | Nova América da  Colina | 411660 | 4116604 | 3507.4 | 680 | 496.7 | 491.6 | 360.8 | 1486 |  |
| 4129PR | 41Paraná | Nova Aurora | 411670 | 4116703 | 11401.6 | 2210.5 | 1614.7 | 1598 | 1172.8 | 8454 |  |
| 4130PR | 41Paraná | Nova Cantu | 411680 | 4116802 | 6583.3 | 1276.4 | 932.3 | 922.7 | 677.1 | 1738 |  |
| 4131PR | 41Paraná | Nova Esperança | 411690 | 4116901 | 27518.9 | 5335.4 | 3897.2 | 3856.9 | 2830.6 | 7066 |  |
| 4132PR | 41Paraná | Nova Esperança do Sudoeste | 411695 | 4116950 | 5139.6 | 996.5 | 727.9 | 720.3 | 528.7 | 1751 |  |
| 4133PR | 41Paraná | Nova Fátima | 411700 | 4117008 | 8252.5 | 1600 | 1168.7 | 1156.6 | 848.8 | 2529 |  |
| 4134PR | 41Paraná | Nova Laranjeiras | 411705 | 4117057 | 11713.4 | 2271 | 1658.9 | 1641.7 | 1204.8 | 4353 |  |
| 4135PR | 41Paraná | Nova Londrina | 411710 | 4117107 | 13296.3 | 2577.9 | 1883 | 1863.6 | 1367.6 | 2318 |  |
| 4136PR | 41Paraná | Nova Olímpia | 411720 | 4117206 | 5705.9 | 1106.3 | 808.1 | 799.7 | 586.9 | 2622 |  |
| 4137PR | 41Paraná | Nova Santa  Bárbara | 411721 | 4117214 | 4107.2 | 796.3 | 581.7 | 575.6 | 422.5 | 1472 |  |
| 4138PR | 41Paraná | Nova Santa Rosa | 411722 | 4117222 | 7983.8 | 1547.9 | 1130.7 | 1119 | 821.2 | 3571 |  |
| 4139PR | 41Paraná | Nova Prata do  Iguaçu | 411725 | 4117255 | 10583.4 | 2051.9 | 1498.8 | 1483.3 | 1088.6 | 3581 |  |
| 4140PR | 41Paraná | Nova Tebas | 411727 | 4117271 | 6720.4 | 1303 | 951.7 | 941.9 | 691.3 | 3194 |  |
| 4141PR | 41Paraná | Novo Itacolomi | 411729 | 4117297 | 2869.9 | 556.4 | 406.4 | 402.2 | 295.2 | 1016 |  |
| 4142PR | 41Paraná | Ortigueira | 411730 | 4117305 | 23129.2 | 4484.3 | 3275.6 | 3241.7 | 2379 | 6238 |  |
| 4143PR | 41Paraná | Ourizona | 411740 | 4117404 | 3443.5 | 667.6 | 487.7 | 482.6 | 354.2 | 1178 |  |
| 4144PR | 41Paraná | Ouro Verde do  Oeste | 411745 | 4117453 | 5897.6 | 1143.4 | 835.2 | 826.6 | 606.6 | 2722 |  |
| 4145PR | 41Paraná | Paiçandu | 411750 | 4117503 | 38745 | 7511.9 | 5487.1 | 5430.3 | 3985.3 | 5438 |  |
| 4146PR | 41Paraná | Palmas | 411760 | 4117602 | 46997.9 | 9112 | 6655.8 | 6587 | 4834.1 | 5361 |  |
| 4147PR | 41Paraná | Palmeira | 411770 | 4117701 | 33305.1 | 6457.2 | 4716.7 | 4667.9 | 3425.7 | 5881 |  |
| 4148PR | 41Paraná | Palmital | 411780 | 4117800 | 14306.7 | 2773.8 | 2026.1 | 2005.2 | 1471.6 | 2343 |  |
| 4149PR | 41Paraná | Palotina | 411790 | 4117909 | 30438.8 | 5901.5 | 4310.7 | 4266.2 | 3130.9 | 12346 |  |
| 4150PR | 41Paraná | Paraíso do Norte | 411800 | 4118006 | 12827.6 | 2487 | 1816.6 | 1797.9 | 1319.4 | 3413 |  |
| 4151PR | 41Paraná | Paranacity | 411810 | 4118105 | 10917.7 | 2116.7 | 1546.2 | 1530.2 | 1123 | 2443 |  |
| 4152PR | 41Paraná | Paranaguá | 411820 | 4118204 | 148616.3 | 28813.7 | 21047 | 20829.4 | 15286.5 | 19147 |  |
| 4153PR | 41Paraná | Paranapoema | 411830 | 4118303 | 3007.9 | 583.2 | 426 | 421.6 | 309.4 | 553 |  |
| 4154PR | 41Paraná | Paranavaí | 411840 | 4118402 | 85608.7 | 16597.8 | 12123.9 | 11998.6 | 8805.6 | 21781 |  |
| 4155PR | 41Paraná | Pato Bragado | 411845 | 4118451 | 5230.1 | 1014 | 740.7 | 733 | 538 | 2852 |  |
| 4156PR | 41Paraná | Pato Branco | 411850 | 4118501 | 77914.9 | 15106.1 | 11034.3 | 10920.2 | 8014.2 | 37148 | 14 |
| 4157PR | 41Paraná | Paula Freitas | 411860 | 4118600 | 5695.8 | 1104.3 | 806.6 | 798.3 | 585.9 | 1447 |  |
| 4158PR | 41Paraná | Paulo Frontin | 411870 | 4118709 | 7194.6 | 1394.9 | 1018.9 | 1008.4 | 740 | 1908 |  |
| 4159PR | 41Paraná | Peabiru | 411880 | 4118808 | 13960.1 | 2706.6 | 1977 | 1956.6 | 1435.9 | 2924 |  |
| 4160PR | 41Paraná | Perobal | 411885 | 4118857 | 5927.3 | 1149.2 | 839.4 | 830.7 | 609.7 | 2519 |  |
| 4161PR | 41Paraná | Pérola | 411890 | 4118907 | 10788.8 | 2091.7 | 1527.9 | 1512.1 | 1109.7 | 3730 |  |
| 4162PR | 41Paraná | Pérola d'Oeste | 411900 | 4119004 | 6663.7 | 1292 | 943.7 | 934 | 685.4 | 3743 |  |
| 4163PR | 41Paraná | Piên | 411910 | 4119103 | 12043 | 2334.9 | 1705.5 | 1687.9 | 1238.7 | 2845 |  |
| 4164PR | 41Paraná | Pinhais | 411915 | 4119152 | 125249.9 | 24283.5 | 17737.9 | 17554.5 | 12883 | 30738 |  |
| 4165PR | 41Paraná | Pinhalão | 411920 | 4119202 | 6342.2 | 1229.6 | 898.2 | 888.9 | 652.3 | 2043 |  |
| 4166PR | 41Paraná | Pinhal de São  Bento | 411925 | 4119251 | 2704.8 | 524.4 | 383.1 | 379.1 | 278.2 | 1244 |  |
| 4167PR | 41Paraná | Pinhão | 411930 | 4119301 | 31551.3 | 6117.2 | 4468.3 | 4422.1 | 3245.3 | 3033 |  |
| 4168PR | 41Paraná | Piraí do Sul | 411940 | 4119400 | 24617.7 | 4772.9 | 3486.4 | 3450.3 | 2532.1 | 2943 |  |
| 4169PR | 41Paraná | Piraquara | 411950 | 4119509 | 103030 | 19975.5 | 14591.1 | 14440.3 | 10597.5 | 16372 |  |
| 4170PR | 41Paraná | Pitanga | 411960 | 4119608 | 32024.2 | 6208.9 | 4535.3 | 4488.4 | 3294 | 7238 |  |
| 4171PR | 41Paraná | Pitangueiras | 411965 | 4119657 | 3030.4 | 587.5 | 429.2 | 424.7 | 311.7 | 1593 |  |
| 4172PR | 41Paraná | Planaltina do  Paraná | 411970 | 4119707 | 4221 | 818.4 | 597.8 | 591.6 | 434.2 | 1220 |  |
| 4173PR | 41Paraná | Planalto | 411980 | 4119806 | 13749.3 | 2665.7 | 1947.2 | 1927 | 1414.2 | 6284 |  |
| 4174PR | 41Paraná | Ponta Grossa | 411990 | 4119905 | 333220.2 | 64604.7 | 47190.6 | 46702.8 | 34274.6 | 33706 |  |
| 4175PR | 41Paraná | Pontal do Paraná | 411995 | 4119954 | 23988.3 | 4650.9 | 3397.2 | 3362.1 | 2467.4 | 4640 |  |
| 4176PR | 41Paraná | Porecatu | 412000 | 4120002 | 13805.9 | 2676.7 | 1955.2 | 1935 | 1420.1 | 4429 |  |
| 4177PR | 41Paraná | Porto Amazonas | 412010 | 4120101 | 4718.2 | 914.8 | 668.2 | 661.3 | 485.3 | 885 |  |
| 4178PR | 41Paraná | Porto Barreiro | 412015 | 4120150 | 3521.8 | 682.8 | 498.8 | 493.6 | 362.2 | 2494 |  |
| 4179PR | 41Paraná | Porto Rico | 412020 | 4120200 | 2574.5 | 499.1 | 364.6 | 360.8 | 264.8 | 760 |  |
| 4180PR | 41Paraná | Porto Vitória | 412030 | 4120309 | 4090.3 | 793 | 579.3 | 573.3 | 420.7 | 1141 |  |
| 4181PR | 41Paraná | Prado Ferreira | 412033 | 4120333 | 3618 | 701.5 | 512.4 | 507.1 | 372.1 | 2189 |  |
| 4182PR | 41Paraná | Pranchita | 412035 | 4120358 | 5491.3 | 1064.7 | 777.7 | 769.6 | 564.8 | 2992 |  |
| 4183PR | 41Paraná | Presidente  Castelo Branco | 412040 | 4120408 | 5074.2 | 983.8 | 718.6 | 711.2 | 521.9 | 1265 |  |
| 4184PR | 41Paraná | Primeiro de Maio | 412050 | 4120507 | 11097.2 | 2151.5 | 1571.6 | 1555.3 | 1141.4 | 3128 |  |
| 4185PR | 41Paraná | Prudentópolis | 412060 | 4120606 | 50896.5 | 9867.8 | 7208 | 7133.4 | 5235.1 | 5894 |  |
| 4186PR | 41Paraná | Quarto Centenário | 412065 | 4120655 | 4765.5 | 923.9 | 674.9 | 667.9 | 490.2 | 3637 |  |
| 4187PR | 41Paraná | Quatiguá | 412070 | 4120705 | 7311.7 | 1417.6 | 1035.5 | 1024.8 | 752.1 | 2931 |  |
| 4188PR | 41Paraná | Quatro Barras | 412080 | 4120804 | 21735.9 | 4214.2 | 3078.2 | 3046.4 | 2235.7 | 5760 |  |
| 4189PR | 41Paraná | Quatro Pontes | 412085 | 4120853 | 3944.7 | 764.8 | 558.6 | 552.9 | 405.7 | 1838 |  |
| 4190PR | 41Paraná | Quedas do Iguaçu | 412090 | 4120903 | 32532.1 | 6307.3 | 4607.2 | 4559.6 | 3346.2 | 7813 |  |
| 4191PR | 41Paraná | Querência do  Norte | 412100 | 4121000 | 12087.1 | 2343.4 | 1711.8 | 1694.1 | 1243.3 | 2548 |  |
| 4192PR | 41Paraná | Quinta do Sol | 412110 | 4121109 | 4925.4 | 954.9 | 697.5 | 690.3 | 506.6 | 1339 |  |
| 4193PR | 41Paraná | Quitandinha | 412120 | 4121208 | 18167.9 | 3522.4 | 2572.9 | 2546.3 | 1868.7 | 4029 |  |
| 4194PR | 41Paraná | Ramilândia | 412125 | 4121257 | 4326.1 | 838.7 | 612.7 | 606.3 | 445 | 1599 |  |
| 4195PR | 41Paraná | Rancho Alegre | 412130 | 4121307 | 3940.2 | 763.9 | 558 | 552.2 | 405.3 | 2242 |  |
| 4196PR | 41Paraná | Rancho Alegre  D'Oeste | 412135 | 4121356 | 2798.4 | 542.6 | 396.3 | 392.2 | 287.8 | 1690 |  |
| 4197PR | 41Paraná | Realeza | 412140 | 4121406 | 16801.4 | 3257.5 | 2379.4 | 2354.8 | 1728.2 | 7850 |  |
| 4198PR | 41Paraná | Rebouças | 412150 | 4121505 | 14673.5 | 2844.9 | 2078.1 | 2056.6 | 1509.3 | 4862 |  |
| 4199PR | 41Paraná | Renascença | 412160 | 4121604 | 6894.2 | 1336.6 | 976.4 | 966.3 | 709.1 | 3264 |  |
| 4200PR | 41Paraná | Reserva | 412170 | 4121703 | 26169.9 | 5073.8 | 3706.2 | 3667.9 | 2691.8 | 6967 |  |
| 4201PR | 41Paraná | Reserva do Iguaçu | 412175 | 4121752 | 7709.2 | 1494.7 | 1091.8 | 1080.5 | 793 | 680 |  |
| 4202PR | 41Paraná | Ribeirão Claro | 412180 | 4121802 | 10808.8 | 2095.6 | 1530.7 | 1514.9 | 1111.8 | 3780 |  |
| 4203PR | 41Paraná | Ribeirão do Pinhal | 412190 | 4121901 | 13476.3 | 2612.8 | 1908.5 | 1888.8 | 1386.2 | 3743 |  |
| 4204PR | 41Paraná | Rio Azul | 412200 | 4122008 | 14786.6 | 2866.8 | 2094.1 | 2072.4 | 1520.9 | 5750 |  |
| 4205PR | 41Paraná | Rio Bom | 412210 | 4122107 | 3318.4 | 643.4 | 470 | 465.1 | 341.3 | 1205 |  |
| 4206PR | 41Paraná | Rio Bonito do  Iguaçu | 412215 | 4122156 | 13468 | 2611.2 | 1907.3 | 1887.6 | 1385.3 | 4183 |  |
| 4207PR | 41Paraná | Rio Branco do Ivaí | 412217 | 4122172 | 4034.5 | 782.2 | 571.4 | 565.5 | 415 | 1019 |  |
| 4208PR | 41Paraná | Rio Branco do Sul | 412220 | 4122206 | 31806.2 | 6166.6 | 4504.4 | 4457.8 | 3271.5 | 6154 |  |
| 4209PR | 41Paraná | Rio Negro | 412230 | 4122305 | 32944.7 | 6387.3 | 4665.6 | 4617.4 | 3388.6 | 4326 |  |
| 4210PR | 41Paraná | Rolândia | 412240 | 4122404 | 62434.6 | 12104.8 | 8842 | 8750.6 | 6421.9 | 27055 | 10 |
| 4211PR | 41Paraná | Roncador | 412250 | 4122503 | 10938.3 | 2120.7 | 1549.1 | 1533.1 | 1125.1 | 3310 |  |
| 4212PR | 41Paraná | Rondon | 412260 | 4122602 | 9363.7 | 1815.4 | 1326.1 | 1312.4 | 963.1 | 2830 |  |
| 4213PR | 41Paraná | Rosário do Ivaí | 412265 | 4122651 | 5305.1 | 1028.6 | 751.3 | 743.5 | 545.7 | 3693 |  |
| 4214PR | 41Paraná | Sabáudia | 412270 | 4122701 | 6494.8 | 1259.2 | 919.8 | 910.3 | 668 | 2064 |  |
| 4215PR | 41Paraná | Salgado Filho | 412280 | 4122800 | 4095.9 | 794.1 | 580.1 | 574.1 | 421.3 | 2049 |  |
| 4216PR | 41Paraná | Salto do Itararé | 412290 | 4122909 | 5136.5 | 995.9 | 727.4 | 719.9 | 528.3 | 1707 |  |
| 4217PR | 41Paraná | Salto do Lontra | 412300 | 4123006 | 14344.6 | 2781.1 | 2031.5 | 2010.5 | 1475.5 | 5268 |  |
| 4218PR | 41Paraná | Santa Amélia | 412310 | 4123105 | 3640.6 | 705.8 | 515.6 | 510.3 | 374.5 | 1232 |  |
| 4219PR | 41Paraná | Santa Cecília do  Pavão | 412320 | 4123204 | 3554.3 | 689.1 | 503.4 | 498.2 | 365.6 | 1047 |  |
| 4220PR | 41Paraná | Santa Cruz de  Monte Castelo | 412330 | 4123303 | 8065 | 1563.6 | 1142.2 | 1130.4 | 829.6 | 2050 |  |
| 4221PR | 41Paraná | Santa Fé | 412340 | 4123402 | 11271.3 | 2185.3 | 1596.2 | 1579.7 | 1159.4 | 1771 |  |
| 4222PR | 41Paraná | Santa Helena | 412350 | 4123501 | 25064.6 | 4859.5 | 3549.6 | 3513 | 2578.1 | 9637 |  |
| 4223PR | 41Paraná | Santa Inês | 412360 | 4123600 | 1743.6 | 338 | 246.9 | 244.4 | 179.3 | 378 |  |
| 4224PR | 41Paraná | Santa Isabel do  Ivaí | 412370 | 4123709 | 8783.7 | 1703 | 1243.9 | 1231.1 | 903.5 | 2528 |  |

2

2

1

1

1

2

1

1

1

4

1

4

6

1

1

1

8

1

5

1

2

4

1

1

2

3

1

2

1

1

2

1

2

1

2

1

1

1

1

1

3

| 4225PR | 41Paraná | Santa Izabel do  Oeste | 412380 | 4123808 | 13971.2 | 2708.7 | 1978.6 | 1958.1 | 1437.1 | 10065 |  |
| --- | --- | --- | --- | --- | --- | --- | --- | --- | --- | --- | --- |
| 4226PR | 41Paraná | Santa Lúcia | 412382 | 4123824 | 3925.6 | 761.1 | 555.9 | 550.2 | 403.8 | 2410 |  |
| 4227PR | 41Paraná | Santa Maria do  Oeste | 412385 | 4123857 | 10851.3 | 2103.9 | 1536.8 | 1520.9 | 1116.1 | 2328 |  |
| 4228PR | 41Paraná | Santa Mariana | 412390 | 4123907 | 12278.9 | 2380.6 | 1738.9 | 1721 | 1263 | 3815 |  |
| 4229PR | 41Paraná | Santa Mônica | 412395 | 4123956 | 3796.6 | 736.1 | 537.7 | 532.1 | 390.5 | 888 |  |
| 4230PR | 41Paraná | Santana do Itararé | 412400 | 4124004 | 5202.1 | 1008.6 | 736.7 | 729.1 | 535.1 | 1756 |  |
| 4231PR | 41Paraná | Santa Tereza do  Oeste | 412402 | 4124020 | 10376.7 | 2011.8 | 1469.5 | 1454.4 | 1067.3 | 4456 |  |
| 4232PR | 41Paraná | Santa Terezinha de Itaipu | 412405 | 4124053 | 22260.1 | 4315.8 | 3152.5 | 3119.9 | 2289.6 | 9575 |  |
| 4233PR | 41Paraná | Santo Antônio da  Platina | 412410 | 4124103 | 44693.7 | 8665.2 | 6329.5 | 6264.1 | 4597.1 | 8038 |  |
| 4234PR | 41Paraná | Santo Antônio do  Caiuá | 412420 | 4124202 | 2723 | 527.9 | 385.6 | 381.6 | 280.1 | 675 |  |
| 4235PR | 41Paraná | Santo Antônio do  Paraíso | 412430 | 4124301 | 2305.5 | 447 | 326.5 | 323.1 | 237.1 | 915 |  |
| 4236PR | 41Paraná | Santo Antônio do  Sudoeste | 412440 | 4124400 | 19692.6 | 3818 | 2788.9 | 2760 | 2025.6 | 6154 |  |
| 4237PR | 41Paraná | Santo Inácio | 412450 | 4124509 | 5409.9 | 1048.9 | 766.2 | 758.2 | 556.5 | 1787 |  |
| 4238PR | 41Paraná | São Carlos do Ivaí | 412460 | 4124608 | 6665 | 1292.2 | 943.9 | 934.1 | 685.6 | 1589 |  |
| 4239PR | 41Paraná | São Jerônimo da  Serra | 412470 | 4124707 | 11406.5 | 2211.5 | 1615.4 | 1598.7 | 1173.3 | 2168 |  |
| 4240PR | 41Paraná | São João | 412480 | 4124806 | 10575.2 | 2050.3 | 1497.7 | 1482.2 | 1087.8 | 2705 |  |
| 4241PR | 41Paraná | São João do  Caiuá | 412490 | 4124905 | 5961 | 1155.7 | 844.2 | 835.5 | 613.1 | 1305 |  |
| 4242PR | 41Paraná | São João do Ivaí | 412500 | 4125001 | 11095.1 | 2151.1 | 1571.3 | 1555 | 1141.2 | 4754 |  |
| 4243PR | 41Paraná | São João do  Triunfo | 412510 | 4125100 | 14500.9 | 2811.4 | 2053.6 | 2032.4 | 1491.5 | 2297 |  |
| 4244PR | 41Paraná | São Jorge d'Oeste | 412520 | 4125209 | 9183.8 | 1780.6 | 1300.6 | 1287.2 | 944.6 | 3571 |  |
| 4245PR | 41Paraná | São Jorge do Ivaí | 412530 | 4125308 | 5601.3 | 1086 | 793.3 | 785.1 | 576.1 | 1433 |  |
| 4246PR | 41Paraná | São Jorge do  Patrocínio | 412535 | 4125357 | 5941.4 | 1151.9 | 841.4 | 832.7 | 611.1 | 2245 |  |
| 4247PR | 41Paraná | São José da Boa  Vista | 412540 | 4125407 | 6458.6 | 1252.2 | 914.7 | 905.2 | 664.3 | 2191 |  |
| 4248PR | 41Paraná | São José das  Palmeiras | 412545 | 4125456 | 3800.3 | 736.8 | 538.2 | 532.6 | 390.9 | 2205 |  |
| 4249PR | 41Paraná | São José dos  Pinhais | 412550 | 4125506 | 293599.8 | 56923.1 | 41579.6 | 41149.7 | 30199.3 | 52814 | 14 |
| 4250PR | 41Paraná | São Manoel do  Paraná | 412555 | 4125555 | 2151.6 | 417.2 | 304.7 | 301.6 | 221.3 | 992 |  |
| 4251PR | 41Paraná | São Mateus do  Sul | 412560 | 4125605 | 43982.7 | 8527.4 | 6228.8 | 6164.4 | 4524 | 6079 |  |
| 4252PR | 41Paraná | São Miguel do  Iguaçu | 412570 | 4125704 | 26835.8 | 5202.9 | 3800.5 | 3761.2 | 2760.3 | 9982 |  |
| 4253PR | 41Paraná | São Pedro do  Iguaçu | 412575 | 4125753 | 6311.3 | 1223.6 | 893.8 | 884.6 | 649.2 | 2253 |  |
| 4254PR | 41Paraná | São Pedro do Ivaí | 412580 | 4125803 | 10654.3 | 2065.7 | 1508.9 | 1493.3 | 1095.9 | 3223 |  |
| 4255PR | 41Paraná | São Pedro do  Paraná | 412590 | 4125902 | 2444 | 473.8 | 346.1 | 342.5 | 251.4 | 732 |  |
| 4256PR | 41Paraná | São Sebastião da  Amoreira | 412600 | 4126009 | 8835.3 | 1713 | 1251.3 | 1238.3 | 908.8 | 2125 |  |
| 4257PR | 41Paraná | São Tomé | 412610 | 4126108 | 5581 | 1082 | 790.4 | 782.2 | 574.1 | 2036 |  |
| 4258PR | 41Paraná | Sapopema | 412620 | 4126207 | 6820.6 | 1322.4 | 965.9 | 955.9 | 701.6 | 1166 |  |
| 4259PR | 41Paraná | Sarandi | 412625 | 4126256 | 89702.9 | 17391.6 | 12703.7 | 12572.4 | 9226.7 | 13092 |  |
| 4260PR | 41Paraná | Saudade do  Iguaçu | 412627 | 4126272 | 5299.5 | 1027.5 | 750.5 | 742.8 | 545.1 | 2420 |  |
| 4261PR | 41Paraná | Sengés | 412630 | 4126306 | 19048.3 | 3693.1 | 2697.6 | 2669.7 | 1959.3 | 1583 |  |
| 4262PR | 41Paraná | Serranópolis do  Iguaçu | 412635 | 4126355 | 4593.4 | 890.6 | 650.5 | 643.8 | 472.5 | 2422 |  |
| 4263PR | 41Paraná | Sertaneja | 412640 | 4126405 | 5655.6 | 1096.5 | 800.9 | 792.7 | 581.7 | 2160 |  |
| 4264PR | 41Paraná | Sertanópolis | 412650 | 4126504 | 16157.6 | 3132.6 | 2288.2 | 2264.6 | 1661.9 | 5916 |  |
| 4265PR | 41Paraná | Siqueira Campos | 412660 | 4126603 | 19816.2 | 3842 | 2806.4 | 2777.4 | 2038.3 | 6237 |  |
| 4266PR | 41Paraná | Sulina | 412665 | 4126652 | 3254.4 | 631 | 460.9 | 456.1 | 334.7 | 2045 |  |
| 4267PR | 41Paraná | Tamarana | 412667 | 4126678 | 13534 | 2624 | 1916.7 | 1896.9 | 1392.1 | 4471 |  |
| 4268PR | 41Paraná | Tamboara | 412670 | 4126702 | 4923.7 | 954.6 | 697.3 | 690.1 | 506.4 | 1958 |  |
| 4269PR | 41Paraná | Tapejara | 412680 | 4126801 | 15490.5 | 3003.3 | 2193.8 | 2171.1 | 1593.3 | 4052 |  |
| 4270PR | 41Paraná | Tapira | 412690 | 4126900 | 5778.7 | 1120.4 | 818.4 | 809.9 | 594.4 | 3511 |  |
| 4271PR | 41Paraná | Teixeira Soares | 412700 | 4127007 | 11331.1 | 2196.9 | 1604.7 | 1588.1 | 1165.5 | 2239 |  |
| 4272PR | 41Paraná | Telêmaco Borba | 412710 | 4127106 | 74765.1 | 14495.5 | 10588.2 | 10478.8 | 7690.2 | 29283 |  |
| 4273PR | 41Paraná | Terra Boa | 412720 | 4127205 | 16556 | 3209.9 | 2344.7 | 2320.4 | 1702.9 | 4827 |  |
| 4274PR | 41Paraná | Terra Rica | 412730 | 4127304 | 16104.6 | 3122.4 | 2280.7 | 2257.2 | 1656.5 | 4142 |  |
| 4275PR | 41Paraná | Terra Roxa | 412740 | 4127403 | 17288.2 | 3351.8 | 2448.4 | 2423 | 1778.2 | 6615 |  |
| 4276PR | 41Paraná | Tibagi | 412750 | 4127502 | 20107.4 | 3898.4 | 2847.6 | 2818.2 | 2068.2 | 5388 |  |
| 4277PR | 41Paraná | Tijucas do Sul | 412760 | 4127601 | 15747.4 | 3053.1 | 2230.1 | 2207.1 | 1619.8 | 4126 |  |
| 4278PR | 41Paraná | Toledo | 412770 | 4127700 | 130213.8 | 25245.9 | 18440.9 | 18250.2 | 13393.6 | 47507 | 17 |
| 4279PR | 41Paraná | Tomazina | 412780 | 4127809 | 8516.8 | 1651.2 | 1206.1 | 1193.7 | 876 | 2714 |  |
| 4280PR | 41Paraná | Três Barras do  Paraná | 412785 | 4127858 | 12068.9 | 2339.9 | 1709.2 | 1691.5 | 1241.4 | 4905 |  |
| 4281PR | 41Paraná | Tunas do Paraná | 412788 | 4127882 | 7442 | 1442.9 | 1053.9 | 1043 | 765.5 | 882 |  |
| 4282PR | 41Paraná | Tuneiras do Oeste | 412790 | 4127908 | 8747.8 | 1696 | 1238.9 | 1226.1 | 899.8 | 3565 |  |
| 4283PR | 41Paraná | Tupãssi | 412795 | 4127957 | 8154 | 1580.9 | 1154.8 | 1142.8 | 838.7 | 3097 |  |
| 4284PR | 41Paraná | Turvo | 412796 | 4127965 | 13664.7 | 2649.3 | 1935.2 | 1915.2 | 1405.5 | 2816 |  |
| 4285PR | 41Paraná | Ubiratã | 412800 | 4128005 | 21589.6 | 4185.8 | 3057.5 | 3025.9 | 2220.7 | 8793 |  |
| 4286PR | 41Paraná | Umuarama | 412810 | 4128104 | 106746 | 20695.9 | 15117.4 | 14961.1 | 10979.7 | 43044 | 13 |
| 4287PR | 41Paraná | União da Vitória | 412820 | 4128203 | 55507.6 | 10761.8 | 7861 | 7779.7 | 5709.4 | 11417 |  |
| 4288PR | 41Paraná | Uniflor | 412830 | 4128302 | 2558.4 | 496 | 362.3 | 358.6 | 263.2 | 634 |  |
| 4289PR | 41Paraná | Uraí | 412840 | 4128401 | 11546.9 | 2238.7 | 1635.3 | 1618.4 | 1187.7 | 3976 |  |
| 4290PR | 41Paraná | Wenceslau Braz | 412850 | 4128500 | 19592.9 | 3798.7 | 2774.7 | 2746.1 | 2015.3 | 4879 |  |
| 4291PR | 41Paraná | Ventania | 412853 | 4128534 | 10935.5 | 2120.2 | 1548.7 | 1532.7 | 1124.8 | 2489 |  |
| 4292PR | 41Paraná | Vera Cruz do  Oeste | 412855 | 4128559 | 8886.9 | 1723 | 1258.6 | 1245.6 | 914.1 | 3941 |  |
| 4293PR | 41Paraná | Verê | 412860 | 4128609 | 7704.1 | 1493.7 | 1091.1 | 1079.8 | 792.4 | 3631 |  |
| 4294PR | 41Paraná | Alto Paraíso | 412862 | 4128625 | 3041.4 | 589.7 | 430.7 | 426.3 | 312.8 | 1121 |  |
| 4295PR | 41Paraná | Doutor Ulysses | 412863 | 4128633 | 5735 | 1111.9 | 812.2 | 803.8 | 589.9 | 273 |  |
| 4296PR | 41Paraná | Virmond | 412865 | 4128658 | 4032.2 | 781.8 | 571 | 565.1 | 414.7 | 2414 |  |
| 4297PR | 41Paraná | Vitorino | 412870 | 4128708 | 6738.2 | 1306.4 | 954.3 | 944.4 | 693.1 | 2725 |  |
| 4298PR | 41Paraná | Xambrê | 412880 | 4128807 | 5941.6 | 1152 | 841.4 | 832.8 | 611.1 | 2582 |  |
| 4299SC |  | Abdon Batista | 420005 | 4200051 | 2622.2 | 511.1 | 371.8 | 385.3 | 280.1 | 931 |  |
| 4300SC |  | Abelardo Luz | 420010 | 4200101 | 17578.3 | 3426.4 | 2492.3 | 2582.8 | 1877.9 | 4135 |  |
| 4301SC |  | Agrolândia | 420020 | 4200200 | 10124.1 | 1973.4 | 1435.4 | 1487.5 | 1081.6 | 1756 |  |
| 4302SC |  | Agronômica | 420030 | 4200309 | 5220.1 | 1017.5 | 740.1 | 767 | 557.7 | 2462 |  |
| 4303SC |  | Água Doce | 420040 | 4200408 | 7085.3 | 1381.1 | 1004.6 | 1041 | 756.9 | 1812 |  |
| 4304SC |  | Águas de  Chapecó | 420050 | 4200507 | 6324.1 | 1232.7 | 896.6 | 929.2 | 675.6 | 1921 |  |
| 4305SC |  | Águas Frias | 420055 | 4200556 | 2403.4 | 468.5 | 340.8 | 353.1 | 256.8 | 983 |  |
| 4306SC |  | Águas Mornas | 420060 | 4200606 | 6026 | 1174.6 | 854.4 | 885.4 | 643.8 | 3091 |  |
| 4307SC |  | Alfredo Wagner | 420070 | 4200705 | 9762.2 | 1902.8 | 1384.1 | 1434.4 | 1042.9 | 3921 |  |
| 4308SC |  | Alto Bela Vista | 420075 | 4200754 | 1981.4 | 386.2 | 280.9 | 291.1 | 211.7 | 763 |  |

3

1

1

3

3

2

1

1

2

3

1

3

2

2

1

1

1

9

1

1

2

2

1

1

1

1

1

3

1

1

1

1

1

1

4309SC	Anchieta	420080	4200804	6017.2	1172.9	853.1	884.1	642.8	1987

4310SC	Angelina	420090	4200903	5056.1	985.5	716.9	742.9	540.1	3681

4311SC	Anita Garibaldi	420100	4201000	7935	1546.7	1125	1165.9	847.7	716

4312SC	Anitápolis	420110	4201109	3237.2	631	459	475.6	345.8	1253

4313SC	Antônio Carlos	420120	4201208	8011.8	1561.7	1135.9	1177.2	855.9	3751

4314SC	Apiúna	420125	4201257	10203.2	1988.8	1446.6	1499.2	1090	2268

4315SC	Arabutã	420127	4201273	4249.8	828.4	602.5	624.4	454	1651

4316SC	Araquari	420130	4201307	31459.5	6132.1	4460.3	4622.4	3360.9	3066

4317SC	Araranguá	420140	4201406	65005.7	12670.9	9216.5	9551.3	6944.6	21517

4318SC	Armazém	420150	4201505	8242.3	1606.6	1168.6	1211	880.5	3387

Santa

4319SC	42Catarina	Arroio Trinta	420160	4201604	3542.6	690.5	502.3	520.5	378.5	1442

Santa

4320SC	42Catarina	Arvoredo	420165	4201653	2261.2	440.8	320.6	332.2	241.6	1436

Santa

4321SC	42Catarina	Ascurra	420170	4201703	7707.1	1502.3	1092.7	1132.4	823.4	1669

Santa

4322SC	42Catarina	Atalanta	420180	4201802	3271.7	637.7	463.9	480.7	349.5	1878

Santa

4323SC	42Catarina	Aurora	420190	4201901	5639.2	1099.2	799.5	828.6	602.4	1959

Santa	Balneário Arroio

4324SC	42Catarina	do Silva	420195	4201950	11340.7	2210.5	1607.9	1666.3	1211.5	3285

Santa	Balneário

4325SC	42Catarina	Camboriú	420200	4202008	125381.5	24439.4	17776.6	18422.4	13394.7	24040

Santa	Balneário Barra do

4326SC	42Catarina	Sul	420205	4202057	9630.7	1877.2	1365.4	1415	1028.9	2505

Santa

4327SC	42Catarina	Balneário Gaivota	420207	4202073	9620	1875.1	1363.9	1413.5	1027.7	3135

Santa

4328SC	42Catarina	Bandeirante	420208	4202081	2808.2	547.4	398.1	412.6	300	1051

Santa

4329SC	42Catarina	Barra Bonita	420209	4202099	1792.2	349.3	254.1	263.3	191.5	863

Santa

4330SC	42Catarina	Barra Velha	420210	4202107	25819.1	5032.7	3660.6	3793.6	2758.3	4231

Santa	Bela Vista do

4331SC	42Catarina	Toldo	420213	4202131	6196.3	1207.8	878.5	910.4	662	795

Santa

4332SC	42Catarina	Belmonte	420215	4202156	2682.8	522.9	380.4	394.2	286.6	785

Santa

4333SC	42Catarina	Benedito Novo	420220	4202206	11030.8	2150.1	1563.9	1620.8	1178.4	2067

Santa

4334SC	42Catarina	Biguaçu	420230	4202305	63526.1	12382.5	9006.7	9333.9	6786.6	16293

Santa

4335SC	42Catarina	Blumenau	420240	4202404	334163.1	65135.1	47377.6	49098.8	35699.1	87109	35

Santa

4336SC	42Catarina	Bocaina do Sul	420243	4202438	3396.1	662	481.5	499	362.8	519

Santa

4337SC	42Catarina	Bombinhas	420245	4202453	17047.2	3322.8	2417	2504.8	1821.2	2594

Santa	Bom Jardim da

4338SC	42Catarina	Serra	420250	4202503	4586.4	894	650.3	673.9	490	418

Santa

4339SC	42Catarina	Bom Jesus	420253	4202537	2776.1	541.1	393.6	407.9	296.6	1731

1

1

1

5

1

8

1

5

1

6

6

2

1

2

2

1

2

5

Santa	Bom Jesus do

4340SC	42Catarina	Oeste	420257	4202578	2145.9	418.3	304.2	315.3	229.2	905

Santa

4341SC	42Catarina	Bom Retiro	420260	4202602	9487.2	1849.2	1345.1	1394	1013.5	2762

Santa

4342SC	42Catarina	Botuverá	420270	4202701	4870.8	949.4	690.6	715.7	520.4	1874

Santa

4343SC	42Catarina	Braço do Norte	420280	4202800	31331.5	6107.1	4442.2	4603.6	3347.2	15712

Santa	Braço do

4344SC	42Catarina	Trombudo	420285	4202859	3616.5	704.9	512.7	531.4	386.4	1358

Santa

4345SC	42Catarina	Brunópolis	420287	4202875	2653.2	517.2	376.2	389.8	283.4	1500

Santa

4346SC	42Catarina	Brusque	420290	4202909	120339.8	23456.6	17061.8	17681.6	12856	26910	10

Santa

4347SC	42Catarina	Caçador	420300	4203006	74946.9	14608.6	10626	11012	8006.7	15614

Santa

4348SC	42Catarina	Caibi	420310	4203105	6215.9	1211.6	881.3	913.3	664.1	2854

Santa

4349SC	42Catarina	Calmon	420315	4203154	3383.6	659.5	479.7	497.2	361.5	428

Santa

4350SC	42Catarina	Camboriú	420320	4203204	72775.6	14185.4	10318.1	10693	7774.7	6569

Santa

4351SC	42Catarina	Capão Alto	420325	4203253	2654.9	517.5	376.4	390.1	283.6	627

Santa

4352SC	42Catarina	Campo Alegre	420330	4203303	11918.3	2323.1	1689.8	1751.2	1273.2	1610

Santa	Campo Belo do

4353SC	42Catarina	Sul	420340	4203402	7290.5	1421.1	1033.6	1071.2	778.9	913

Santa

4354SC	42Catarina	Campo Erê	420350	4203501	9002	1754.7	1276.3	1322.7	961.7	2090

Santa

4355SC	42Catarina	Campos Novos	420360	4203600	34663.8	6756.7	4914.6	5093.2	3703.2	6239

Santa

4356SC	42Catarina	Canelinha	420370	4203709	11457.4	2233.3	1624.4	1683.4	1224	2084

Santa

4357SC	42Catarina	Canoinhas	420380	4203808	53820.9	10490.8	7630.7	7907.9	5749.8	7198

Santa

4358SC	42Catarina	Capinzal	420390	4203907	21889.2	4266.6	3103.5	3216.2	2338.5	6799

Santa

4359SC	42Catarina	Capivari de Baixo	420395	4203956	23346.5	4550.7	3310.1	3430.3	2494.1	6156

Santa

4360SC	Catanduvas	420400	4204004	10241.4	1996.3	1452	1504.8	1094.1	2037

4361SC	Caxambu do Sul	420410	4204103	4056	790.6	575.1	596	433.3	1946

4362SC	Celso Ramos	420415	4204152	2764.2	538.8	391.9	406.1	295.3	801

4363SC	Cerro Negro	420417	4204178	3374.3	657.7	478.4	495.8	360.5	410

Chapadão do

4364SC	Lageado	420419	4204194	2885	562.3	409	423.9	308.2	1088

4365SC	Chapecó	420420	4204202	202476.3	39466.7	28707.1	29750	21630.8	75600	29

4366SC	Cocal do Sul	420425	4204251	15980.4	3114.9	2265.7	2348	1707.2	8332

4367SC	Concórdia	420430	4204301	71906.4	14016	10194.9	10565.3	7681.8	21449

4368SC	Cordilheira Alta	420435	4204350	4120.8	803.2	584.2	605.5	440.2	2336

4369SC	Coronel Freitas	420440	4204400	10157.3	1979.9	1440.1	1492.4	1085.1	3999

4370SC	Coronel Martins	420445	4204459	2515.3	490.3	356.6	369.6	268.7	1067

4371SC	Corupá	420450	4204509	14928.2	2909.8	2116.5	2193.4	1594.8	2283

4372SC	Correia Pinto	420455	4204558	13881.9	2705.9	1968.2	2039.7	1483	1914

4373SC	Criciúma	420460	4204608	204462.1	39853.8	28988.6	30041.8	21842.9	75422	21

4374SC	Cunha Porã	420470	4204707	10897.3	2124.1	1545	1601.1	1164.2	2669

4375SC	Cunhataí	420475	4204756	1929.5	376.1	273.6	283.5	206.1	1451

4376SC	Curitibanos	420480	4204806	38911.5	7584.6	5516.9	5717.3	4157	10447

4377SC	Descanso	420490	4204905	8487.4	1654.4	1203.3	1247.1	906.7	2882

4378SC	Dionísio Cerqueira	420500	4205001	15220.3	2966.7	2157.9	2236.3	1626	2759

4379SC	Dona Emma	420510	4205100	3949.3	769.8	559.9	580.3	421.9	1366

Santa

4380SC	42Catarina	Doutor Pedrinho	420515	4205159	3868.9	754.1	548.5	568.5	413.3	1067

Santa

4381SC	42Catarina	Entre Rios	420517	4205175	3123.7	608.9	442.9	459	333.7	1066

Santa

4382SC	42Catarina	Ermo	420519	4205191	2066.8	402.9	293	303.7	220.8	820

Santa

4383SC	42Catarina	Erval Velho	420520	4205209	4421.4	861.8	626.9	649.6	472.3	954

Santa	Faxinal dos

4384SC	42Catarina	Guedes	420530	4205308	10716.7	2088.9	1519.4	1574.6	1144.9	3915

Santa

4385SC	42Catarina	Flor do Sertão	420535	4205357	1592.6	310.4	225.8	234	170.1	1180

Santa

4386SC	42Catarina	Florianópolis	420540	4205407	462378.1	90126.7	65556	67937.5	49396.4	92997	26

Santa

4387SC	42Catarina	Formosa do Sul	420543	4205431	2568.9	500.7	364.2	377.5	274.4	944

Santa

4388SC	42Catarina	Forquilhinha	420545	4205456	24738.9	4822.1	3507.5	3634.9	2642.9	8743

Santa

4389SC	42Catarina	Fraiburgo	420550	4205506	35647.1	6948.3	5054	5237.7	3808.2	9686

Santa

4390SC	42Catarina	Frei Rogério	420555	4205555	2265.6	441.6	321.2	332.9	242	804

Santa

4391SC	42Catarina	Galvão	420560	4205605	3225.4	628.7	457.3	473.9	344.6	1378

Santa

4392SC	42Catarina	Garopaba	420570	4205704	20647.9	4024.7	2927.5	3033.8	2205.8	4412

Santa

4393SC	42Catarina	Garuva	420580	4205803	16491.4	3214.5	2338.2	2423.1	1761.8	2615

Santa

4394SC	42Catarina	Gaspar	420590	4205902	63975.5	12470.1	9070.4	9400	6834.6	5747

Santa	Governador Celso

4395SC	42Catarina	Ramos	420600	4206009	13783.7	2686.7	1954.3	2025.2	1472.5	5738

Santa

4396SC	42Catarina	Grão Pará	420610	4206108	6423.6	1252.1	910.7	943.8	686.2	3232

Santa

4397SC	42Catarina	Gravatal	420620	4206207	11116.4	2166.8	1576.1	1633.3	1187.6	3738

Santa

4398SC	42Catarina	Guabiruba	420630	4206306	21167.5	4126	3001.1	3110.2	2261.4	1527

Santa

4399SC	42Catarina	Guaraciaba	420640	4206405	10349	2017.2	1467.3	1520.6	1105.6	3017

Santa

4400SC	42Catarina	Guaramirim	420650	4206504	40072	7810.8	5681.4	5887.8	4280.9	5626

Santa

3

1

2

3

1

1

1

1

1

1

1

1

6

5

2

1

5

1

2

4401SC	42Catarina	Guarujá do Sul	420660	4206603	5055.8	985.5	716.8	742.9	540.1	1960

Santa

4402SC	42Catarina	Guatambú	420665	4206652	4714.3	918.9	668.4	692.7	503.6	2064

Santa

4403SC	42Catarina	Herval d'Oeste	420670	4206702	22010.6	4290.3	3120.7	3234	2351.4	4466

Santa

4404SC	42Catarina	Ibiam	420675	4206751	1959.8	382	277.9	288	209.4	1091

Santa

4405SC	42Catarina	Ibicaré	420680	4206801	3306.6	644.5	468.8	485.8	353.2	843

Santa

4406SC	42Catarina	Ibirama	420690	4206900	18214.2	3550.3	2582.4	2676.2	1945.8	4421

Santa

4407SC	42Catarina	Içara	420700	4207007	55651.3	10847.6	7890.2	8176.9	5945.3	18983

Santa

4408SC	42Catarina	Ilhota	420710	4207106	13311.9	2594.8	1887.4	1955.9	1422.1	1984

Santa

4409SC	42Catarina	Imaruí	420720	4207205	10975.7	2139.4	1556.1	1612.7	1172.5	2649

Santa

4410SC	42Catarina	Imbituba	420730	4207304	42661.3	8315.5	6048.5	6268.3	4557.6	19151

Santa

4411SC	42Catarina	Imbuia	420740	4207403	5977.5	1165.1	847.5	878.3	638.6	1101

Santa

4412SC	42Catarina	Indaial	420750	4207502	62263.6	12136.4	8827.7	9148.4	6651.7	8619

Santa

4413SC	42Catarina	Iomerê	420757	4207577	2853.1	556.1	404.5	419.2	304.8	1029

Santa

4414SC	42Catarina	Ipira	420760	4207601	4629.4	902.4	656.4	680.2	494.6	1286

Santa

4415SC	42Catarina	Iporã do Oeste	420765	4207650	8740.2	1703.6	1239.2	1284.2	933.7	3664

Santa

4416SC	42Catarina	Ipuaçu	420768	4207684	7182	1399.9	1018.3	1055.3	767.3	2307

Santa

4417SC	42Catarina	Ipumirim	420770	4207700	7438.2	1449.9	1054.6	1092.9	794.6	2360

Santa

4418SC	42Catarina	Iraceminha	420775	4207759	4136.9	806.4	586.5	607.8	442	1643

Santa

4419SC	42Catarina	Irani	420780	4207809	10012.5	1951.6	1419.6	1471.1	1069.6	2199

Santa

4420SC	42Catarina	Irati	420785	4207858	2025.2	394.8	287.1	297.6	216.4	636

Santa

4421SC	Irineópolis	420790	4207908	10882.6	2121.2	1542.9	1599	1162.6	1742

4422SC	Itá	420800	4208005	6331.5	1234.1	897.7	930.3	676.4	3034

4423SC	Itaiópolis	420810	4208104	21071.3	4107.2	2987.5	3096	2251.1	2040

4424SC	Itajaí	420820	4208203	201993.2	39372.5	28638.6	29679	21579.2	60898	19

4425SC	Itapema	420830	4208302	55585.5	10834.7	7880.9	8167.2	5938.3	14806

4426SC	Itapiranga	420840	4208401	16213.9	3160.4	2298.8	2382.3	1732.2	4448

4427SC	Itapoá	420845	4208450	17683.9	3446.9	2507.2	2598.3	1889.2	2398

4428SC	Ituporanga	420850	4208500	23757	4630.7	3368.3	3490.6	2538	7187

4429SC	Jaborá	420860	4208609	4009.5	781.5	568.5	589.1	428.3	1460

4430SC	Jacinto Machado	420870	4208708	10566.8	2059.7	1498.2	1552.6	1128.9	3411

4431SC	Jaguaruna	420880	4208807	18714.9	3647.9	2653.4	2749.8	1999.3	3862

4432SC	Jaraguá do Sul	420890	4208906	160764.4	31336.2	22793.2	23621.2	17174.7	23742

4433SC	Jardinópolis	420895	4208955	1677.5	327	237.8	246.5	179.2	427

4434SC	Joaçaba	420900	4209003	28670.8	5588.5	4064.9	4212.6	3062.9	5707

4435SC	Joinville	420910	4209102	554659.6	108114.2	78639.6	81496.5	59255	198208	66

4436SC	José Boiteux	420915	4209151	4848.5	945.1	687.4	712.4	518	1130

4437SC	Jupiá	420917	4209177	2135	416.2	302.7	313.7	228.1	1369

4438SC	Lacerdópolis	420920	4209201	2232.6	435.2	316.5	328	238.5	890

4439SC	Lages	420930	4209300	157894.9	30776.9	22386.3	23199.6	16868.1	27107

4440SC	Laguna	420940	4209409	47014.9	9164.1	6665.8	6907.9	5022.7	16086

4441SC	Lajeado Grande	420945	4209458	1466.7	285.9	207.9	215.5	156.7	1252

Santa

4442SC	42Catarina	Laurentino	420950	4209508	6505.6	1268.1	922.4	955.9	695	2691

Santa

4443SC	42Catarina	Lauro Muller	420960	4209607	14867.2	2897.9	2107.9	2184.4	1588.3	5864

Santa

4444SC	42Catarina	Lebon Régis	420970	4209706	12028.1	2344.5	1705.3	1767.3	1285	1645

Santa

4445SC	42Catarina	Leoberto Leal	420980	4209805	3222.7	628.2	456.9	473.5	344.3	1005

Santa

4446SC	42Catarina	Lindóia do Sul	420985	4209854	4626.1	901.7	655.9	679.7	494.2	1643

Santa

4447SC	42Catarina	Lontras	420990	4209904	11218.5	2186.7	1590.6	1648.3	1198.5	2467

Santa

4448SC	42Catarina	Luiz Alves	421000	4210001	11687.5	2278.1	1657.1	1717.3	1248.6	2236

Santa

4449SC	42Catarina	Luzerna	421003	4210035	5668.5	1104.9	803.7	832.9	605.6	1933

Santa

4450SC	42Catarina	Macieira	421005	4210050	1809.5	352.7	256.6	265.9	193.3	927

Santa

4451SC	42Catarina	Mafra	421010	4210100	54827.3	10686.9	7773.4	8055.8	5857.3	4184

Santa

4452SC	42Catarina	Major Gercino	421020	4210209	3375.1	657.9	478.5	495.9	360.6	411

Santa

4453SC	42Catarina	Major Vieira	421030	4210308	7821.3	1524.5	1108.9	1149.2	835.6	603

Santa

4454SC	42Catarina	Maracajá	421040	4210407	6871.9	1339.5	974.3	1009.7	734.1	1829

Santa

4455SC	42Catarina	Maravilha	421050	4210506	23999.4	4678	3402.6	3526.2	2563.9	5743

Santa

4456SC	42Catarina	Marema	421055	4210555	2014.8	392.7	285.7	296	215.2	2130

Santa

4457SC	42Catarina	Massaranduba	421060	4210605	15823.4	3084.3	2243.4	2324.9	1690.4	2430

Santa

4458SC	42Catarina	Matos Costa	421070	4210704	2696.6	525.6	382.3	396.2	288.1	33

Santa

4459SC	42Catarina	Meleiro	421080	4210803	7035.5	1371.4	997.5	1033.7	751.6	2657

Santa

4460SC	42Catarina	Mirim Doce	421085	4210852	2425.3	472.7	343.9	356.4	259.1	529

Santa

4461SC	42Catarina	Modelo	421090	4210902	4141.8	807.3	587.2	608.6	442.5	2072

Santa

4462SC	42Catarina	Mondaí	421100	4211009	11030.8	2150.1	1563.9	1620.8	1178.4	3701

Santa

5

1

7

4

1

1

1

1

1

3

1

2

1

1

2

1

1

1

1

4463SC	42Catarina	Monte Carlo	421105	4211058	9620.4	1875.2	1364	1413.5	1027.8	1299

Santa

4464SC	42Catarina	Monte Castelo	421110	4211108	8396.6	1636.7	1190.5	1233.7	897	1066

Santa

4465SC	42Catarina	Morro da Fumaça	421120	4211207	17023.3	3318.2	2413.6	2501.2	1818.6	4763

Santa

4466SC	42Catarina	Morro Grande	421125	4211256	2906.2	566.5	412	427	310.5	1348

Santa

4467SC	42Catarina	Navegantes	421130	4211306	71104.1	13859.6	10081.1	10447.4	7596.1	11950

Santa

4468SC	42Catarina	Nova Erechim	421140	4211405	4659.9	908.3	660.7	684.7	497.8	1973

Santa

4469SC	42Catarina	Nova Itaberaba	421145	4211454	4316.1	841.3	611.9	634.2	461.1	2177

Santa

4470SC	42Catarina	Nova Trento	421150	4211504	13405.9	2613.1	1900.7	1969.7	1432.2	4263

Santa

4471SC	42Catarina	Nova Veneza	421160	4211603	14283.4	2784.1	2025.1	2098.7	1525.9	6048

Santa

4472SC	42Catarina	Novo Horizonte	421165	4211652	2612.4	509.2	370.4	383.8	279.1	1466

Santa

4473SC	42Catarina	Orleans	421170	4211702	22242.8	4335.6	3153.6	3268.2	2376.2	4607

Santa

4474SC	42Catarina	Otacílio Costa	421175	4211751	17596.4	3429.9	2494.8	2585.5	1879.8	1938

Santa

4475SC	42Catarina	Ouro	421180	4211801	7368.8	1436.3	1044.7	1082.7	787.2	3517

Santa

4476SC	42Catarina	Ouro Verde	421185	4211850	2255.4	439.6	319.8	331.4	240.9	1667

Santa

4477SC	42Catarina	Paial	421187	4211876	1645.3	320.7	233.3	241.7	175.8	777

Santa

4478SC	42Catarina	Painel	421189	4211892	2368.1	461.6	335.7	347.9	253	295

Santa

4479SC	42Catarina	Palhoça	421190	4211900	154895.8	30192.3	21961.1	22758.9	16547.7	53476	19

Santa

4480SC	42Catarina	Palma Sola	421200	4212007	7635.3	1488.3	1082.5	1121.9	815.7	2089

Santa

4481SC	42Catarina	Palmeira	421205	4212056	2508.9	489	355.7	368.6	268	362

Santa

4482SC	42Catarina	Palmitos	421210	4212106	16171.6	3152.2	2292.8	2376.1	1727.6	8350

Santa

4483SC	Papanduva	421220	4212205	18653.7	3636	2644.7	2740.8	1992.8	2946

4484SC	Paraíso	421223	4212239	3784.6	737.7	536.6	556.1	404.3	1874

4485SC	Passo de Torres	421225	4212254	7735.8	1507.9	1096.8	1136.6	826.4	1374

4486SC	Passos Maia	421227	4212270	4310.5	840.2	611.1	633.3	460.5	1753

4487SC	Paulo Lopes	421230	4212304	7117.7	1387.4	1009.1	1045.8	760.4	3305

4488SC	Pedras Grandes	421240	4212403	4059.3	791.2	575.5	596.4	433.7	1226

4489SC	Penha	421250	4212502	28885.2	5630.3	4095.3	4244.1	3085.8	4425

4490SC	Peritiba	421260	4212601	2904	566	411.7	426.7	310.2	1116

4491SC	Petrolândia	421270	4212700	6064.1	1182	859.8	891	647.8	2359

4492SC	Balneário Piçarras	421280	4212809	20135.6	3924.8	2854.8	2958.5	2151.1	4665

4493SC	Pinhalzinho	421290	4212908	18358.5	3578.4	2602.9	2697.4	1961.3	3908

4494SC	Pinheiro Preto	421300	4213005	3356.1	654.2	475.8	493.1	358.5	844

4495SC	Piratuba	421310	4213104	4352.8	848.4	617.1	639.6	465	851

4496SC	Planalto Alegre	421315	4213153	2774.2	540.7	393.3	407.6	296.4	1468

4497SC	Pomerode	421320	4213203	30676.3	5979.4	4349.3	4507.3	3277.2	9398

4498SC	Ponte Alta	421330	4213302	4814.4	938.4	682.6	707.4	514.3	723

Ponte Alta do

4499SC	Norte	421335	4213351	3373.5	657.6	478.3	495.7	360.4	530

4500SC	Ponte Serrada	421340	4213401	11360.4	2214.4	1610.7	1669.2	1213.6	2605

4501SC	Porto Belo	421350	4213500	18762	3657.1	2660.1	2756.7	2004.4	2578

4502SC	Porto União	421360	4213609	34590.1	6742.3	4904.2	5082.3	3695.3	5011

4503SC	Pouso Redondo	421370	4213708	16177.5	3153.3	2293.6	2377	1728.3	3937

Santa

4504SC	42Catarina	Praia Grande	421380	4213807	7329.5	1428.7	1039.2	1076.9	783	1904

Santa	Presidente

4505SC	42Catarina	Castello Branco	421390	4213906	1654.1	322.4	234.5	243	176.7	460

Santa

4506SC	42Catarina	Presidente Getúlio	421400	4214003	16228.8	3163.3	2300.9	2384.5	1733.7	4017

Santa

4507SC	42Catarina	Presidente Nereu	421410	4214102	2296.9	447.7	325.7	337.5	245.4	957

Santa

4508SC	42Catarina	Princesa	421415	4214151	2852.6	556	404.4	419.1	304.7	652

Santa

4509SC	42Catarina	Quilombo	421420	4214201	10121.2	1972.8	1435	1487.1	1081.3	2574

Santa

4510SC	42Catarina	Rancho Queimado	421430	4214300	2826.1	550.9	400.7	415.2	301.9	997

Santa

4511SC	42Catarina	Rio das Antas	421440	4214409	6207.4	1209.9	880.1	912.1	663.1	1607

Santa

4512SC	42Catarina	Rio do Campo	421450	4214508	6098.9	1188.8	864.7	896.1	651.6	1536

Santa

4513SC	42Catarina	Rio do Oeste	421460	4214607	7325.5	1427.9	1038.6	1076.3	782.6	3032

Santa

4514SC	42Catarina	Rio dos Cedros	421470	4214706	11015.8	2147.2	1561.8	1618.6	1176.8	2757

Santa

4515SC	42Catarina	Rio do Sul	421480	4214805	66309.7	12925.1	9401.4	9742.9	7083.9	31151	10

Santa

4516SC	42Catarina	Rio Fortuna	421490	4214904	4548.8	886.7	644.9	668.4	486	2535

Santa

4517SC	42Catarina	Rio Negrinho	421500	4215000	41243.4	8039.2	5847.5	6059.9	4406.1	2261

Santa

4518SC	42Catarina	Rio Rufino	421505	4215059	2470.6	481.6	350.3	363	263.9	372

Santa

4519SC	42Catarina	Riqueza	421507	4215075	4737.4	923.4	671.7	696.1	506.1	1757

Santa

4520SC	42Catarina	Rodeio	421510	4215109	11284	2199.5	1599.8	1658	1205.5	2770

Santa

4521SC	42Catarina	Romelândia	421520	4215208	5230.3	1019.5	741.6	768.5	558.8	1695

Santa

4522SC	42Catarina	Salete	421530	4215307	7539.4	1469.6	1068.9	1107.8	805.4	2905

Santa

4523SC	42Catarina	Saltinho	421535	4215356	3890.6	758.4	551.6	571.6	415.6	894

Santa

4524SC	42Catarina	Salto Veloso	421540	4215406	4526.9	882.4	641.8	665.1	483.6	1345

Santa

3

1

1

1

1

1

6

5

1

1

7

2

1

2

1

4

1

4525SC	42Catarina	Sangão	421545	4215455	11566.9	2254.6	1640	1699.5	1235.7	1901

Santa

4526SC	42Catarina	Santa Cecília	421550	4215505	16360.5	3189	2319.6	2403.9	1747.8	2009

Santa

4527SC	42Catarina	Santa Helena	421555	4215554	2311.7	450.6	327.8	339.7	247	1258

Santa	Santa Rosa de

4528SC	42Catarina	Lima	421560	4215604	2112.5	411.8	299.5	310.4	225.7	1238

Santa

4529SC	42Catarina	Santa Rosa do Sul	421565	4215653	8245.8	1607.3	1169.1	1211.6	880.9	3098

Santa

4530SC	42Catarina	Santa Terezinha	421567	4215679	8819.3	1719.1	1250.4	1295.8	942.2	1398

Santa	Santa Terezinha

4531SC	42Catarina	do Progresso	421568	4215687	2681.2	522.6	380.1	394	286.4	1049

Santa

4532SC	42Catarina	Santiago do Sul	421569	4215695	1371.5	267.3	194.5	201.5	146.5	990

Santa	Santo Amaro da

4533SC	42Catarina	Imperatriz	421570	4215703	21597.5	4209.8	3062.1	3173.3	2307.3	19345

Santa

4534SC	42Catarina	São Bernardino	421575	4215752	2544	495.9	360.7	373.8	271.8	808

Santa

4535SC	42Catarina	São Bento do Sul	421580	4215802	79925.9	15579.2	11331.9	11743.6	8538.6	17406

Santa

4536SC	42Catarina	São Bonifácio	421590	4215901	2939.7	573	416.8	431.9	314.1	1178

Santa

4537SC	42Catarina	São Carlos	421600	4216008	10827.2	2110.4	1535.1	1590.8	1156.7	4651

Santa	São Cristovão do

4538SC	42Catarina	Sul	421605	4216057	5299.9	1033.1	751.4	778.7	566.2	491

Santa

4539SC	42Catarina	São Domingos	421610	4216107	9484.9	1848.8	1344.8	1393.6	1013.3	3317

Santa	São Francisco do

4540SC	42Catarina	Sul	421620	4216206	47727.5	9303	6766.8	7012.6	5098.8	23101

Santa	São João do

4541SC	42Catarina	Oeste	421625	4216255	6220.5	1212.5	881.9	914	664.5	2487

Santa

4542SC	42Catarina	São João Batista	421630	4216305	31859.8	6210.1	4517.1	4681.2	3403.6	6153

Santa	São João do

4543SC	42Catarina	Itaperiú	421635	4216354	3597.8	701.3	510.1	528.6	384.4	548

Santa

4544SC	42Catarina	São João do Sul	421640	4216404	7176.2	1398.8	1017.4	1054.4	766.6	1797

Santa

4545SC	42Catarina	São Joaquim	421650	4216503	25982.8	5064.6	3683.8	3817.7	2775.8	1235

4546SC	São José	421660	4216602	228857.7	44608.9	32447.4	33626.2	24449.1	56688	19

São José do

4547SC	Cedro	421670	4216701	13822.3	2694.2	1959.7	2030.9	1476.7	3620

São José do

4548SC	Cerrito	421680	4216800	8839.8	1723.1	1253.3	1298.8	944.4	1097

São Lourenço do

4549SC	Oeste	421690	4216909	22997	4482.6	3260.5	3379	2456.8	6558

4550SC	São Ludgero	421700	4217006	12229.8	2383.8	1733.9	1796.9	1306.5	4749

4551SC	São Martinho	421710	4217105	3210.4	625.8	455.2	471.7	343	2035

São Miguel da

4552SC	Boa Vista	421715	4217154	1870.1	364.5	265.1	274.8	199.8	899

São Miguel do

4553SC	Oeste	421720	4217204	38530.2	7510.3	5462.8	5661.3	4116.2	12714

São Pedro de

4554SC	Alcântara	421725	4217253	5275.8	1028.4	748	775.2	563.6	1750

4555SC	Saudades	421730	4217303	9424.8	1837.1	1336.2	1384.8	1006.9	3580

4556SC	Schroeder	421740	4217402	18355.5	3577.9	2602.4	2697	1960.9	3062

4557SC	Seara	421750	4217501	17315.3	3375.1	2455	2544.1	1849.8	7475

4558SC	Serra Alta	421755	4217550	3293.9	642	467	484	351.9	1559

4559SC	Siderópolis	421760	4217600	13555.6	2642.3	1921.9	1991.7	1448.2	5087

4560SC	Sombrio	421770	4217709	28586.9	5572.2	4053.1	4200.3	3054	7897

4561SC	Sul Brasil	421775	4217758	2630.1	512.7	372.9	386.4	281	1487

4562SC	Taió	421780	4217808	17900.3	3489.1	2537.9	2630.1	1912.3	4475

4563SC	Tangará	421790	4217907	8714.9	1698.7	1235.6	1280.5	931	2152

4564SC	Tigrinhos	421795	4217956	1712.4	333.8	242.8	251.6	182.9	1294

4565SC	Tijucas	421800	4218004	34761	6775.6	4928.4	5107.5	3713.6	6446

Santa

4566SC	42Catarina	Timbé do Sul	421810	4218103	5353.3	1043.5	759	786.6	571.9	1532

Santa

4567SC	42Catarina	Timbó	421820	4218202	40611.7	7916	5757.9	5967.1	4338.6	6924

Santa

4568SC	42Catarina	Timbó Grande	421825	4218251	7549.8	1471.6	1070.4	1109.3	806.6	675

Santa

4569SC	42Catarina	Três Barras	421830	4218301	18779.4	3660.5	2662.5	2759.3	2006.2	1132

Santa

4570SC	42Catarina	Treviso	421835	4218350	3741.2	729.2	530.4	549.7	399.7	2721

Santa

4571SC	42Catarina	Treze de Maio	421840	4218400	7015.1	1367.4	994.6	1030.7	749.4	2153

Santa

4572SC	42Catarina	Treze Tílias	421850	4218509	7107.3	1385.4	1007.7	1044.3	759.3	1235

Santa

4573SC	42Catarina	Trombudo Central	421860	4218608	6975.9	1359.7	989	1025	745.2	1402

Santa

4574SC	42Catarina	Tubarão	421870	4218707	101848.8	19852.4	14440.1	14964.7	10880.6	33654

Santa

4575SC	42Catarina	Tunápolis	421875	4218756	4611.2	898.8	653.8	677.5	492.6	1761

Santa

4576SC	42Catarina	Turvo	421880	4218806	12423.9	2421.7	1761.5	1825.5	1327.3	3304

Santa

4577SC	42Catarina	União do Oeste	421885	4218855	2710.5	528.3	384.3	398.3	289.6	1605

Santa

4578SC	42Catarina	Urubici	421890	4218905	11013.9	2146.8	1561.6	1618.3	1176.6	842

Santa

4579SC	42Catarina	Urupema	421895	4218954	2485.9	484.6	352.5	365.3	265.6	474

Santa

4580SC	42Catarina	Urussanga	421900	4219002	20833.8	4060.9	2953.8	3061.1	2225.7	10743

Santa

4581SC	42Catarina	Vargeão	421910	4219101	3569.5	695.8	506.1	524.5	381.3	2008

Santa

4582SC	42Catarina	Vargem	421915	4219150	2648	516.1	375.4	389.1	282.9	547

Santa

4583SC	42Catarina	Vargem Bonita	421917	4219176	4669.3	910.1	662	686.1	498.8	1048

Santa

4584SC	42Catarina	Vidal Ramos	421920	4219200	6337.8	1235.4	898.6	931.2	677.1	3073

Santa

4585SC	42Catarina	Videira	421930	4219309	50305.3	9805.5	7132.3	7391.4	5374.2	11021

Santa

4586SC	42Catarina	Vitor Meireles	421935	4219358	5115.7	997.2	725.3	751.7	546.5	1623

Santa

1

1

3

1

2

2

8

1

3

1

4

4

2

1

1

1

1

1

1

2

1

1

4587SC	42Catarina	Witmarsum	421940	4219408	3798	740.3	538.5	558	405.7	1381

Santa

4588SC	42Catarina	Xanxerê	421950	4219507	47702.6	9298.2	6763.3	7009	5096.1	11964

Santa

4589SC	42Catarina	Xavantina	421960	4219606	4060	791.4	575.6	596.5	433.7	1920

Santa

4590SC	42Catarina	Xaxim	421970	4219705	27305.6	5322.4	3871.4	4012	2917.1	6625

Santa

4591SC	42Catarina	Zortéa	421985	4219853	3188	621.4	452	468.4	340.6	1044

Rio Grande

4592RS	43do Sul	Aceguá	430003	4300034	4654.9	1027.6	634.3	779.1	475.2	1039

Rio Grande

4593RS	43do Sul	Água Santa	430005	4300059	3785.1	835.6	515.8	633.6	386.4	2169

Rio Grande

4594RS	43do Sul	Agudo	430010	4300109	16859.3	3721.9	2297.2	2821.9	1721.1	4427

Rio Grande

4595RS	43do Sul	Ajuricaba	430020	4300208	7268.9	1604.7	990.5	1216.7	742.1	3270

Rio Grande

4596RS	43do Sul	Alecrim	430030	4300307	6717.9	1483	915.4	1124.4	685.8	4258

Rio Grande

4597RS	43do Sul	Alegrete	430040	4300406	77176.7	17037.5	10516.1	12917.9	7878.7	33918	12

Rio Grande

4598RS	43do Sul	Alegria	430045	4300455	4037.3	891.3	550.1	675.8	412.2	2458

Rio Grande Almirante

4599RS	43do Sul	Tamandaré do Sul	430047	4300471	2056.4	454	280.2	344.2	209.9	1252

Rio Grande

4600RS	43do Sul	Alpestre	430050	4300505	7451.6	1645	1015.4	1247.3	760.7	4045

Rio Grande

4601RS	43do Sul	Alto Alegre	430055	4300554	1792.3	395.7	244.2	300	183	1174

Rio Grande

4602RS	43do Sul	Alto Feliz	430057	4300570	2996.7	661.6	408.3	501.6	305.9	914

Rio Grande

4603RS	43do Sul	Alvorada	430060	4300604	204176.6	45074	27821.1	34175.1	20843.6	52710	15

Rio Grande

4604RS	43do Sul	Amaral Ferrador	430063	4300638	6708.3	1480.9	914.1	1122.8	684.8	709

Rio Grande

4605RS	43do Sul	Ametista do Sul	430064	4300646	7461.7	1647.2	1016.7	1248.9	761.7	3847

Rio Grande

4606RS	43do Sul	André da Rocha	430066	4300661	1279.2	282.4	174.3	214.1	130.6	340

Rio Grande

4607RS	Anta Gorda	430070	4300703	6125.7	1352.3	834.7	1025.3	625.3	2096

4608RS	Antônio Prado	430080	4300802	13100.9	2892.2	1785.1	2192.8	1337.4	3789

4609RS	Arambaré	430085	4300851	3702.2	817.3	504.5	619.7	377.9	1252

4610RS	Araricá	430087	4300877	5259.1	1161	716.6	880.3	536.9	2284

4611RS	Aratiba	430090	4300901	6530.1	1441.6	889.8	1093	666.6	2186

4612RS	Arroio do Meio	430100	4301008	19840.3	4379.9	2703.4	3320.9	2025.4	7364

4613RS	Arroio do Sal	430105	4301057	8757.4	1933.3	1193.3	1465.8	894	3521

4614RS	Arroio do Padre	430107	4301073	2849.9	629.1	388.3	477	290.9	892

4615RS	Arroio dos Ratos	430110	4301107	14013.1	3093.5	1909.4	2345.5	1430.5	4736

4616RS	Arroio do Tigre	430120	4301206	13137.7	2900.3	1790.1	2199	1341.2	5100

4617RS	Arroio Grande	430130	4301305	18664.9	4120.5	2543.3	3124.1	1905.4	2392

4618RS	Arvorezinha	430140	4301404	10448.8	2306.7	1423.8	1748.9	1066.7	3698

4619RS	Augusto Pestana	430150	4301503	7022.9	1550.4	956.9	1175.5	716.9	3202

4620RS	43	Áurea	430155	4301552	3673.5	811	500.6	614.9	375	1784

Rio Grande

4621RS	43do Sul	Bagé	430160	4301602	120159.3	26526.4	16372.9	20112.3	12266.6	16513

Rio Grande

4622RS	43do Sul	Balneário Pinhal	430163	4301636	12264.2	2707.4	1671.1	2052.8	1252	3223

Rio Grande

4623RS	43do Sul	Barão	430165	4301651	5991	1322.6	816.3	1002.8	611.6	1032

Rio Grande

4624RS	43do Sul	Barão de Cotegipe	430170	4301701	6659.4	1470.1	907.4	1114.7	679.8	2291

Rio Grande

4625RS	43do Sul	Barão do Triunfo	430175	4301750	7301	1611.8	994.8	1222	745.3	1509

Rio Grande

4626RS	43do Sul	Barracão	430180	4301800	5401.5	1192.4	736	904.1	551.4	2680

Rio Grande

4627RS	43do Sul	Barra do Guarita	430185	4301859	3195.3	705.4	435.4	534.8	326.2	1145

Rio Grande

4628RS	43do Sul	Barra do Quaraí	430187	4301875	4148.4	915.8	565.3	694.4	423.5	570

Rio Grande

4629RS	43do Sul	Barra do Ribeiro	430190	4301909	13109.8	2894.1	1786.3	2194.3	1338.3	2167

Rio Grande

4630RS	43do Sul	Barra do Rio Azul	430192	4301925	1909.6	421.6	260.2	319.6	194.9	821

Rio Grande

4631RS	43do Sul	Barra Funda	430195	4301958	2468.1	544.9	336.3	413.1	252	1198

Rio Grande

4632RS	43do Sul	Barros Cassal	430200	4302006	11317.6	2498.5	1542.1	1894.3	1155.4	1532

Rio Grande Benjamin Constant

4633RS	43do Sul	do Sul	430205	4302055	2217.6	489.6	302.2	371.2	226.4	709

Rio Grande

4634RS	43do Sul	Bento Gonçalves	430210	4302105	113118.5	24972	15413.5	18933.8	11547.8	33212	10

Rio Grande Boa Vista das

4635RS	43do Sul	Missões	430215	4302154	2137.6	471.9	291.3	357.8	218.2	653

Rio Grande Boa Vista do

4636RS	43do Sul	Buricá	430220	4302204	6721.8	1483.9	915.9	1125.1	686.2	3295

Rio Grande Boa Vista do

4637RS	43do Sul	Cadeado	430222	4302220	2487.5	549.1	338.9	416.4	253.9	476

Rio Grande

4638RS	43do Sul	Boa Vista do Incra	430223	4302238	2529.4	558.4	344.7	423.4	258.2	1098

Rio Grande

4639RS	43do Sul	Boa Vista do Sul	430225	4302253	2818.1	622.1	384	471.7	287.7	796

Rio Grande

4640RS	43do Sul	Bom Jesus	430230	4302303	11617.1	2564.6	1582.9	1944.5	1185.9	1399

Rio Grande

4641RS	43do Sul	Bom Princípio	430235	4302352	12841.2	2834.8	1749.7	2149.4	1310.9	1907

Rio Grande

4642RS	43do Sul	Bom Progresso	430237	4302378	2211	488.1	301.3	370.1	225.7	811

Rio Grande

4643RS	43do Sul	Bom Retiro do Sul	430240	4302402	11970	2642.5	1631	2003.5	1222	2766

Rio Grande

4644RS	43do Sul	Boqueirão do Leão	430245	4302451	7798.5	1721.6	1062.6	1305.3	796.1	1643

Rio Grande

4645RS	43do Sul	Bossoroca	430250	4302501	6746.4	1489.3	919.3	1129.2	688.7	3436

Rio Grande

4646RS	43do Sul	Bozano	430258	4302584	2201.6	486	300	368.5	224.8	1946

Rio Grande

4647RS	43do Sul	Braga	430260	4302600	3619.2	799	493.2	605.8	369.5	960

Rio Grande

4648RS	43do Sul	Brochier	430265	4302659	4897.2	1081.1	667.3	819.7	499.9	1052

Rio Grande

1

5

1

1

2

1

7

1

1

7

8

2

1

2

4

4

4649RS	43do Sul	Butiá	430270	4302709	20906	4615.2	2848.7	3499.3	2134.2	3771

Rio Grande

4650RS	43do Sul	Caçapava do Sul	430280	4302808	34145.3	7537.9	4652.6	5715.2	3485.8	8446

Rio Grande

4651RS	43do Sul	Cacequi	430290	4302907	13436.7	2966.3	1830.9	2249	1371.7	3436

Rio Grande

4652RS	43do Sul	Cachoeira do Sul	430300	4303004	84390	18629.9	11499	14125.2	8615	24294

Rio Grande

4653RS	43do Sul	Cachoeirinha	430310	4303103	124655.1	27518.9	16985.5	20864.8	12725.5	89159	24

Rio Grande

4654RS	43do Sul	Cacique Doble	430320	4303202	5014.1	1106.9	683.2	839.3	511.9	3698

Rio Grande

4655RS	43do Sul	Caibaté	430330	4303301	4979.5	1099.3	678.5	833.5	508.3	3412

Rio Grande

4656RS	43do Sul	Caiçara	430340	4303400	5015.3	1107.2	683.4	839.5	512	2877

Rio Grande

4657RS	43do Sul	Camaquã	430350	4303509	65024.8	14354.9	8860.3	10883.9	6638.1	21320

Rio Grande

4658RS	43do Sul	Camargo	430355	4303558	2684.3	592.6	365.8	449.3	274	791

Rio Grande

4659RS	43do Sul	Cambará do Sul	430360	4303608	6592.3	1455.3	898.3	1103.4	673	1036

Rio Grande Campestre da

4660RS	43do Sul	Serra	430367	4303673	3348.5	739.2	456.3	560.5	341.8	735

Rio Grande Campina das

4661RS	43do Sul	Missões	430370	4303707	5953.7	1314.3	811.3	996.5	607.8	3571

Rio Grande

4662RS	43do Sul	Campinas do Sul	430380	4303806	5564.3	1228.4	758.2	931.4	568	2529

Rio Grande

4663RS	43do Sul	Campo Bom	430390	4303905	63521.4	14023	8655.4	10632.2	6484.6	27236

Rio Grande

4664RS	43do Sul	Campo Novo	430400	4304002	5156.6	1138.4	702.6	863.1	526.4	1902

Rio Grande

4665RS	43do Sul	Campos Borges	430410	4304101	3476	767.4	473.6	581.8	354.9	1473

Rio Grande

4666RS	43do Sul	Candelária	430420	4304200	31067.9	6858.5	4233.3	5200.2	3171.6	5379

Rio Grande

4667RS	43do Sul	Cândido Godói	430430	4304309	6496.9	1434.3	885.3	1087.5	663.2	3856

Rio Grande

4668RS	43do Sul	Candiota	430435	4304358	9214	2034.1	1255.5	1542.2	940.6	849

Rio Grande

4669RS	Canela	430440	4304408	42040.9	9280.9	5728.5	7036.8	4291.8	8032

4670RS	Canguçu	430450	4304507	55106.5	12165.3	7508.8	9223.7	5625.6	12882

4671RS	Canoas	430460	4304606	337343.3	74471.9	45966.4	56464.6	34438	72562	19

4672RS	Canudos do Vale	430461	4304614	1797.5	396.8	244.9	300.9	183.5	569

Capão Bonito do

4673RS	Sul	430462	4304622	1739.9	384.1	237.1	291.2	177.6	620

4674RS	Capão da Canoa	430463	4304630	46935.4	10361.5	6395.4	7856.1	4791.4	12448

4675RS	Capão do Cipó	430465	4304655	3361.7	742.1	458.1	562.7	343.2	1143

4676RS	Capão do Leão	430466	4304663	25057.3	5531.6	3414.3	4194.1	2558	2286

4677RS	Capivari do Sul	430467	4304671	4245.9	937.3	578.5	710.7	433.4	2169

Capela de

4678RS	Santana	430468	4304689	11658.6	2573.8	1588.6	1951.4	1190.2	2638

4679RS	Capitão	430469	4304697	2720.9	600.7	370.7	455.4	277.8	1048

4680RS	Carazinho	430470	4304705	61253.8	13522.4	8346.4	10252.7	6253.2	19250

4681RS	Caraá	430471	4304713	7789.9	1719.7	1061.5	1303.9	795.2	1854

4682RS	Carlos Barbosa	430480	4304804	27362.9	6040.6	3728.5	4580	2793.4	3218

4683RS	Carlos Gomes	430485	4304853	1540.6	340.1	209.9	257.9	157.3	629

4684RS	Casca	430490	4304903	8922.7	1969.8	1215.8	1493.5	910.9	3711

4685RS	Caseiros	430495	4304952	3125.9	690.1	425.9	523.2	319.1	1269

4686RS	Catuípe	430500	4305009	9241.4	2040.1	1259.2	1546.8	943.4	3813

4687RS	Caxias do Sul	430510	4305108	471177.5	104017.1	64202.6	78865.8	48100.6	127483	44

4688RS	Centenário	430511	4305116	2978.5	657.5	405.9	498.5	304.1	1560

4689RS	Cerrito	430512	4305124	6372.4	1406.8	868.3	1066.6	650.5	1348

Rio Grande

4690RS	43do Sul	Cerro Branco	430513	4305132	4610.1	1017.7	628.2	771.6	470.6	1470

Rio Grande

4691RS	43do Sul	Cerro Grande	430515	4305157	2410	532	328.4	403.4	246	1082

Rio Grande Cerro Grande do

4692RS	43do Sul	Sul	430517	4305173	11183.5	2468.9	1523.9	1871.9	1141.7	1562

Rio Grande

4693RS	43do Sul	Cerro Largo	430520	4305207	13808.4	3048.3	1881.5	2311.3	1409.6	2148

Rio Grande

4694RS	43do Sul	Chapada	430530	4305306	9468.5	2090.3	1290.2	1584.8	966.6	5194

Rio Grande

4695RS	43do Sul	Charqueadas	430535	4305355	37958.5	8379.7	5172.2	6353.5	3875	9070

Rio Grande

4696RS	43do Sul	Charrua	430537	4305371	3445.7	760.7	469.5	576.7	351.8	1199

Rio Grande

4697RS	43do Sul	Chiapetta	430540	4305405	3989.3	880.7	543.6	667.7	407.3	2338

Rio Grande

4698RS	43do Sul	Chuí	430543	4305439	6308.8	1392.7	859.6	1056	644	138

Rio Grande

4699RS	43do Sul	Chuvisca	430544	4305447	5208.4	1149.8	709.7	871.8	531.7	1135

Rio Grande

4700RS	43do Sul	Cidreira	430545	4305454	14247.8	3145.3	1941.4	2384.8	1454.5	3337

Rio Grande

4701RS	43do Sul	Ciríaco	430550	4305504	4924.1	1087	671	824.2	502.7	2271

Rio Grande

4702RS	43do Sul	Colinas	430558	4305587	2462.1	543.5	335.5	412.1	251.3	904

Rio Grande

4703RS	43do Sul	Colorado	430560	4305603	3454.5	762.6	470.7	578.2	352.7	997

Rio Grande

4704RS	43do Sul	Condor	430570	4305702	6724.2	1484.4	916.2	1125.5	686.4	1757

Rio Grande

4705RS	43do Sul	Constantina	430580	4305801	9953.4	2197.3	1356.3	1666	1016.1	5368

Rio Grande

4706RS	43do Sul	Coqueiro Baixo	430583	4305835	1538.8	339.7	209.7	257.6	157.1	833

Rio Grande

4707RS	43do Sul	Coqueiros do Sul	430585	4305850	2433.5	537.2	331.6	407.3	248.4	1544

Rio Grande

4708RS	43do Sul	Coronel Barros	430587	4305871	2517.8	555.8	343.1	421.4	257	2083

Rio Grande

4709RS	43do Sul	Coronel Bicaco	430590	4305900	7694.2	1698.6	1048.4	1287.9	785.5	1261

Rio Grande

4710RS	43do Sul	Coronel Pilar	430593	4305934	1711.2	377.8	233.2	286.4	174.7	814

Rio Grande

6

1

1

1

1

2

1

1

5

5

1

1

2

3

4711RS	43do Sul	Cotiporã	430595	4305959	3947.8	871.5	537.9	660.8	403	2256

Rio Grande

4712RS	43do Sul	Coxilha	430597	4305975	2839.3	626.8	386.9	475.2	289.9	1237

Rio Grande

4713RS	43do Sul	Crissiumal	430600	4306007	14037.1	3098.8	1912.7	2349.5	1433	6350

Rio Grande

4714RS	43do Sul	Cristal	430605	4306056	7668.5	1692.9	1044.9	1283.6	782.8	2494

Rio Grande

4715RS	43do Sul	Cristal do Sul	430607	4306072	2874.9	634.7	391.7	481.2	293.5	1311

Rio Grande

4716RS	43do Sul	Cruz Alta	430610	4306106	62735.1	13849.4	8548.3	10500.6	6404.4	16202

Rio Grande

4717RS	43do Sul	Cruzaltense	430613	4306130	2051.7	452.9	279.6	343.4	209.4	1237

Rio Grande

4718RS	43do Sul	Cruzeiro do Sul	430620	4306205	12338.8	2723.9	1681.3	2065.3	1259.6	2995

Rio Grande

4719RS	43do Sul	David Canabarro	430630	4306304	4771.8	1053.4	650.2	798.7	487.1	2899

Rio Grande

4720RS	43do Sul	Derrubadas	430632	4306320	3085.5	681.2	420.4	516.5	315	1103

Rio Grande Dezesseis de

4721RS	43do Sul	Novembro	430635	4306353	2735.6	603.9	372.8	457.9	279.3	1448

Rio Grande Dilermando de

4722RS	43do Sul	Aguiar	430637	4306379	3088.5	681.8	420.8	517	315.3	744

Rio Grande

4723RS	43do Sul	Dois Irmãos	430640	4306403	29955.2	6612.9	4081.7	5013.9	3058	13606

Rio Grande Dois Irmãos das

4724RS	43do Sul	Missões	430642	4306429	2136.7	471.7	291.1	357.6	218.1	691

Rio Grande

4725RS	43do Sul	Dois Lajeados	430645	4306452	3372.5	744.5	459.5	564.5	344.3	1927

Rio Grande

4726RS	43do Sul	Dom Feliciano	430650	4306502	14988.6	3308.9	2042.3	2508.8	1530.1	1578

Rio Grande Dom Pedro de

4727RS	43do Sul	Alcântara	430655	4306551	2580.4	569.6	351.6	431.9	263.4	1683

Rio Grande

4728RS	43do Sul	Dom Pedrito	430660	4306601	39283.8	8672.3	5352.8	6575.3	4010.3	4838

Rio Grande

4729RS	43do Sul	Dona Francisca	430670	4306700	3309.2	730.5	450.9	553.9	337.8	1505

Rio Grande Doutor Maurício

4730RS	43do Sul	Cardoso	430673	4306734	5091	1123.9	693.7	852.1	519.7	2684

Rio Grande

4731RS	Doutor Ricardo	430675	4306759	2042.7	450.9	278.3	341.9	208.5	903

4732RS	Eldorado do Sul	430676	4306767	37542	8287.8	5115.5	6283.8	3832.5	3971

4733RS	Encantado	430680	4306809	21658.7	4781.4	2951.2	3625.2	2211	7798

Encruzilhada do

4734RS	Sul	430690	4306908	25408.5	5609.2	3462.2	4252.9	2593.9	9247

4735RS	Engenho Velho	430692	4306924	1355.9	299.3	184.8	227	138.4	794

4736RS	Entre-Ijuís	430693	4306932	8885.2	1961.5	1210.7	1487.2	907.1	2823

4737RS	Entre Rios do Sul	430695	4306957	3011.9	664.9	410.4	504.1	307.5	1718

4738RS	Erebango	430697	4306973	3020.6	666.8	411.6	505.6	308.4	1708

4739RS	Erechim	430700	4307005	101273	22357	13799.5	16951.1	10338.6	29683	10

4740RS	Ernestina	430705	4307054	3163.5	698.4	431.1	529.5	322.9	1418

4741RS	Herval	430710	4307104	6876.1	1518	936.9	1150.9	702	691

4742RS	Erval Grande	430720	4307203	5118	1129.8	697.4	856.7	522.5	2758

4743RS	Erval Seco	430730	4307302	7617.1	1681.6	1037.9	1275	777.6	4193

4744RS	43	Esmeralda	430740	4307401	3257.7	719.2	443.9	545.3	332.6	849

Rio Grande

4745RS	43do Sul	Esperança do Sul	430745	4307450	3183.4	702.8	433.8	532.8	325	1502

Rio Grande

4746RS	43do Sul	Espumoso	430750	4307500	15593.1	3442.3	2124.7	2610	1591.8	3932

Rio Grande

4747RS	43do Sul	Estação	430755	4307559	6076.4	1341.4	828	1017.1	620.3	4287

Rio Grande

4748RS	43do Sul	Estância Velha	430760	4307609	46087.8	10174.3	6279.9	7714.2	4704.9	11270

Rio Grande

4749RS	43do Sul	Esteio	430770	4307708	82863	18292.8	11290.9	13869.6	8459.2	28414

Rio Grande

4750RS	43do Sul	Estrela	430780	4307807	32420.8	7157.2	4417.7	5426.6	3309.7	6653

Rio Grande

4751RS	43do Sul	Estrela Velha	430781	4307815	3690.1	814.6	502.8	617.6	376.7	1927

Rio Grande

4752RS	43do Sul	Eugênio de Castro	430783	4307831	2685.9	592.9	366	449.6	274.2	1228

Rio Grande

4753RS	43do Sul	Fagundes Varela	430786	4307864	2675	590.5	364.5	447.7	273.1	1799

Rio Grande

4754RS	43do Sul	Farroupilha	430790	4307906	67936.8	14997.7	9257.1	11371.3	6935.4	17963

Rio Grande

4755RS	43do Sul	Faxinal do Soturno	430800	4308003	6769	1494.3	922.3	1133	691	4051

Rio Grande

4756RS	43do Sul	Faxinalzinho	430805	4308052	2505.3	553.1	341.4	419.3	255.8	1940

Rio Grande

4757RS	43do Sul	Fazenda Vilanova	430807	4308078	4075.8	899.8	555.4	682.2	416.1	942

Rio Grande

4758RS	43do Sul	Feliz	430810	4308102	12999.4	2869.7	1771.3	2175.8	1327.1	2730

Rio Grande

4759RS	43do Sul	Flores da Cunha	430820	4308201	28925.6	6385.6	3941.4	4841.6	2952.9	7336

Rio Grande

4760RS	43do Sul	Floriano Peixoto	430825	4308250	1948.2	430.1	265.5	326.1	198.9	1390

Rio Grande

4761RS	43do Sul	Fontoura Xavier	430830	4308300	10710.6	2364.5	1459.4	1792.7	1093.4	1569

Rio Grande

4762RS	43do Sul	Formigueiro	430840	4308409	6977.4	1540.3	950.7	1167.9	712.3	2697

Rio Grande

4763RS	43do Sul	Forquetinha	430843	4308433	2488	549.3	339	416.4	254	860

Rio Grande Fortaleza dos

4764RS	43do Sul	Valos	430845	4308458	4540.7	1002.4	618.7	760	463.5	1549

Rio Grande Frederico

4765RS	43do Sul	Westphalen	430850	4308508	30218.6	6671.1	4117.6	5058	3084.9	13508

Rio Grande

4766RS	43do Sul	Garibaldi	430860	4308607	32836.4	7249	4474.3	5496.2	3352.1	7968

Rio Grande

4767RS	43do Sul	Garruchos	430865	4308656	3158.9	697.4	430.4	528.7	322.5	1483

Rio Grande

4768RS	43do Sul	Gaurama	430870	4308706	5817.6	1284.3	792.7	973.8	593.9	2655

Rio Grande

4769RS	43do Sul	General Câmara	430880	4308805	8543.8	1886.1	1164.2	1430.1	872.2	2204

Rio Grande

4770RS	43do Sul	Gentil	430885	4308854	1684.1	371.8	229.5	281.9	171.9	841

Rio Grande

4771RS	43do Sul	Getúlio Vargas	430890	4308904	16407.1	3622	2235.6	2746.2	1674.9	13467

Rio Grande

4772RS	43do Sul	Giruá	430900	4309001	16901.1	3731.1	2302.9	2828.9	1725.4	10542

1

1

1

4

7

2

6

1

2

3

2

3

3

2

4

4

2

1

1

1

1

1

Rio Grande

4773RS	43do Sul	Glorinha	430905	4309050	7460.2	1646.9	1016.5	1248.7	761.6	8758

Rio Grande

4774RS	43do Sul	Gramado	430910	4309100	34272.3	7566	4669.9	5736.5	3498.7	11045

Rio Grande Gramado dos

4775RS	43do Sul	Loureiros	430912	4309126	2228.5	492	303.7	373	227.5	1069

Rio Grande

4776RS	43do Sul	Gramado Xavier	430915	4309159	4165.4	919.6	567.6	697.2	425.2	818

Rio Grande

4777RS	43do Sul	Gravataí	430920	4309209	269399.9	59472.7	36708.4	45092.2	27502	87224	23

Rio Grande

4778RS	43do Sul	Guabiju	430925	4309258	1584	349.7	215.8	265.1	161.7	803

Rio Grande

4779RS	43do Sul	Guaíba	430930	4309308	97710.4	21570.5	13314	16354.8	9974.9	16817

Rio Grande

4780RS	43do Sul	Guaporé	430940	4309407	24276.6	5359.3	3307.9	4063.4	2478.3	8985

Rio Grande Guarani das

4781RS	43do Sul	Missões	430950	4309506	8005.5	1767.3	1090.8	1340	817.2	2664

Rio Grande

4782RS	43do Sul	Harmonia	430955	4309555	4553.9	1005.3	620.5	762.2	464.9	1650

Rio Grande do Sul

4783RS	43	Herveiras	430957	4309571	3021.4	667	411.7	505.7	308.4	514

Rio Grande

4784RS	43do Sul	Horizontina	430960	4309605	18993	4192.9	2588	3179.1	1938.9	5371

Rio Grande

4785RS	43do Sul	Hulha Negra	430965	4309654	6415.6	1416.3	874.2	1073.8	654.9	889

Rio Grande

4786RS	43do Sul	Humaitá	430970	4309704	4928	1087.9	671.5	824.8	503.1	1491

Rio Grande

4787RS	43do Sul	Ibarama	430975	4309753	4451	982.6	606.5	745	454.4	1480

Rio Grande

4788RS	43do Sul	Ibiaçá	430980	4309803	4776.9	1054.5	650.9	799.6	487.7	2621

Rio Grande

4789RS	43do Sul	Ibiraiaras	430990	4309902	7331.8	1618.6	999	1227.2	748.5	4524

Rio Grande

4790RS	43do Sul	Ibirapuitã	430995	4309951	4099.9	905.1	558.7	686.2	418.5	1405

Rio Grande

4791RS	43do Sul	Ibirubá	431000	4310009	19991.6	4413.3	2724.1	3346.2	2040.9	3156

Rio Grande

4792RS	Igrejinha	431010	4310108	34106.4	7529.3	4647.3	5708.7	3481.8	5170

4793RS	Ijuí	431020	4310207	81820.9	18062.8	11148.9	13695.2	8352.8	34788	10

4794RS	Ilópolis	431030	4310306	4148.2	915.8	565.2	694.3	423.5	1490

4795RS	Imbé	431033	4310330	19923.5	4398.3	2714.8	3334.8	2033.9	6692

4796RS	Imigrante	431036	4310363	3104.5	685.3	423	519.6	316.9	1028

4797RS	Independência	431040	4310405	6536.4	1443	890.6	1094.1	667.3	2217

4798RS	Inhacorá	431041	4310413	2282.7	503.9	311	382.1	233	1026

4799RS	Ipê	431043	4310439	6345.4	1400.8	864.6	1062.1	647.8	1548

4800RS	Ipiranga do Sul	431046	4310462	1950.4	430.6	265.8	326.5	199.1	1730

4801RS	Iraí	431050	4310504	7866.9	1736.7	1071.9	1316.8	803.1	3472

4802RS	Itaara	431053	4310538	5272.4	1163.9	718.4	882.5	538.2	1383

4803RS	Itacurubi	431055	4310553	3499.8	772.6	476.9	585.8	357.3	1587

4804RS	Itapuca	431057	4310579	2280	503.3	310.7	381.6	232.8	949

4805RS	Itaqui	431060	4310603	38493.8	8497.9	5245.2	6443.1	3929.7	9584

4806RS	Itati	431065	4310652	2556.9	564.5	348.4	428	261	1155

4807RS	Itatiba do Sul	431070	4310702	3900.1	861	531.4	652.8	398.1	1791

4808RS	Ivorá	431075	4310751	2091.1	461.6	284.9	350	213.5	652

4809RS	Ivoti	431080	4310801	21881.8	4830.6	2981.6	3662.6	2233.8	10120

4810RS	Jaboticaba	431085	4310850	4044.2	892.8	551.1	676.9	412.9	2247

4811RS	Jacuizinho	431087	4310876	2615	577.3	356.3	437.7	267	894

4812RS	Jacutinga	431090	4310900	3656.5	807.2	498.2	612	373.3	1871

Rio Grande

4813RS	43do Sul	Jaguarão	431100	4311007	27842.1	6146.4	3793.8	4660.2	2842.3	4765

Rio Grande

4814RS	43do Sul	Jaguari	431110	4311106	11393.4	2515.2	1552.5	1907	1163.1	3088

Rio Grande

4815RS	43do Sul	Jaquirana	431112	4311122	4057.2	895.7	552.8	679.1	414.2	669

Rio Grande

4816RS	43do Sul	Jari	431113	4311130	3598.1	794.3	490.3	602.3	367.3	1210

Rio Grande

4817RS	43do Sul	Jóia	431115	4311155	8539.4	1885.2	1163.6	1429.3	871.8	4071

Rio Grande

4818RS	43do Sul	Júlio de Castilhos	431120	4311205	19747.1	4359.4	2690.7	3305.3	2015.9	6734

Rio Grande Lagoa Bonita do

4819RS	43do Sul	Sul	431123	4311239	2794.2	616.8	380.7	467.7	285.2	1170

Rio Grande

4820RS	43do Sul	Lagoão	431125	4311254	6397.7	1412.4	871.8	1070.8	653.1	771

Rio Grande Lagoa dos Três

4821RS	43do Sul	Cantos	431127	4311270	1625.3	358.8	221.5	272	165.9	932

Rio Grande do Sul

4822RS	43	Lagoa Vermelha	431130	4311304	28028	6187.5	3819.1	4691.3	2861.3	7385

Rio Grande

4823RS	43do Sul	Lajeado	431140	4311403	77537.3	17117.1	10565.2	12978.2	7915.5	19375

Rio Grande

4824RS	43do Sul	Lajeado do Bugre	431142	4311429	2553	563.6	347.9	427.3	260.6	406

Rio Grande

4825RS	43do Sul	Lavras do Sul	431150	4311502	7710.5	1702.2	1050.6	1290.6	787.1	982

Rio Grande

4826RS	43do Sul	Liberato Salzano	431160	4311601	5644	1246	769.1	944.7	576.2	3274

Rio Grande

4827RS	43do Sul	Lindolfo Collor	431162	4311627	5624.3	1241.6	766.4	941.4	574.2	2317

Rio Grande

4828RS	43do Sul	Linha Nova	431164	4311643	1682	371.3	229.2	281.5	171.7	732

Rio Grande

4829RS	43do Sul	Machadinho	431170	4311700	5563.8	1228.3	758.1	931.3	568	1930

Rio Grande

4830RS	43do Sul	Mampituba	431173	4311734	3037.2	670.5	413.8	508.4	310.1	1093

Rio Grande

4831RS	43do Sul	Manoel Viana	431175	4311759	7261.8	1603.1	989.5	1215.5	741.3	3001

Rio Grande

4832RS	43do Sul	Maquiné	431177	4311775	6929.2	1529.7	944.2	1159.8	707.4	2292

Rio Grande

4833RS	43do Sul	Maratá	431179	4311791	2627.2	580	358	439.7	268.2	966

3

3

1

1

2

2

6

1

3

1

1

2

1

Rio Grande

4834RS	43do Sul	Marau	431180	4311809	39924.8	8813.8	5440.2	6682.6	4075.8	10264

Rio Grande

4835RS	43do Sul	Marcelino Ramos	431190	4311908	4921.7	1086.5	670.6	823.8	502.4	1706

Rio Grande

4836RS	43do Sul	Mariana Pimentel	431198	4311981	3867.6	853.8	527	647.4	394.8	1107

Rio Grande

4837RS	43do Sul	Mariano Moro	431200	4312005	2171.5	479.4	295.9	363.5	221.7	1122

Rio Grande

4838RS	43do Sul	Marques de Souza	431205	4312054	4103.4	905.9	559.1	686.8	418.9	1098

Rio Grande

4839RS	43do Sul	Mata	431210	4312104	5071.4	1119.6	691	848.9	517.7	2056

Rio Grande

4840RS	43do Sul	Mato Castelhano	431213	4312138	2532.6	559.1	345.1	423.9	258.5	1252

Rio Grande

4841RS	43do Sul	Mato Leitão	431215	4312153	4171.9	921	568.5	698.3	425.9	2054

Rio Grande

4842RS	43do Sul	Mato Queimado	431217	4312179	1765.4	389.7	240.6	295.5	180.2	2266

Rio Grande Maximiliano de

4843RS	43do Sul	Almeida	431220	4312203	4772.9	1053.7	650.4	798.9	487.2	1519

Rio Grande

4844RS	43do Sul	Minas do Leão	431225	4312252	7912.9	1746.9	1078.2	1324.5	807.8	1290

Rio Grande

4845RS	43do Sul	Miraguaí	431230	4312302	4918.3	1085.8	670.2	823.2	502.1	1594

Rio Grande

4846RS	43do Sul	Montauri	431235	4312351	1529.5	337.7	208.4	256	156.1	1429

Rio Grande Monte Alegre dos

4847RS	43do Sul	Campos	431237	4312377	3194.5	705.2	435.3	534.7	326.1	736

Rio Grande

4848RS	43do Sul	Monte Belo do Sul	431238	4312385	2659.4	587.1	362.4	445.1	271.5	1120

Rio Grande

4849RS	43do Sul	Montenegro	431240	4312401	62545.5	13807.5	8522.4	10468.9	6385	2533

Rio Grande

4850RS	43do Sul	Mormaço	431242	4312427	2919.6	644.5	397.8	488.7	298.1	1087

Rio Grande

4851RS	43do Sul	Morrinhos do Sul	431244	4312443	3136.6	692.4	427.4	525	320.2	1735

Rio Grande

4852RS	43do Sul	Morro Redondo	431245	4312450	6448.8	1423.6	878.7	1079.4	658.3	1098

Rio Grande

4853RS	43do Sul	Morro Reuter	431247	4312476	6042.7	1334	823.4	1011.4	616.9	4174

Rio Grande

4854RS	43do Sul	Mostardas	431250	4312500	12563.1	2773.4	1711.8	2102.8	1282.5	5158

Rio Grande

4855RS	43do Sul	Muçum	431260	4312609	4923.3	1086.9	670.8	824.1	502.6	1612

Rio Grande

4856RS	43do Sul	Muitos Capões	431261	4312617	3098.8	684.1	422.2	518.7	316.3	718

Rio Grande

4857RS	43do Sul	Muliterno	431262	4312625	1870.1	412.8	254.8	313	190.9	1475

Rio Grande

4858RS	43do Sul	Não-Me-Toque	431265	4312658	16821.8	3713.6	2292.1	2815.6	1717.3	6123

Rio Grande

4859RS	43do Sul	Nicolau Vergueiro	431267	4312674	1730	381.9	235.7	289.6	176.6	975

Rio Grande

4860RS	43do Sul	Nonoai	431270	4312708	12098.9	2671	1648.6	2025.1	1235.1	6138

Rio Grande

do Sul

4861RS	43	Nova Alvorada	431275	4312757	3400.4	750.7	463.3	569.2	347.1	1963

Rio Grande

4862RS	43do Sul	Nova Araçá	431280	4312807	4354	961.2	593.3	728.8	444.5	1488

Rio Grande

4863RS	43do Sul	Nova Bassano	431290	4312906	9386.1	2072.1	1278.9	1571	958.2	3920 4864RS	Nova Boa Vista	431295	4312955	1916.4	423.1	261.1	320.8	195.6	1049

4865RS	Nova Bréscia	431300	4313003	3286.3	725.5	447.8	550.1	335.5	1387

4866RS	Nova Candelária	431301	4313011	2770.2	611.5	377.5	463.7	282.8	2051

Nova Esperança

4867RS	do Sul	431303	4313037	5003.6	1104.6	681.8	837.5	510.8	2746

4868RS	Nova Hartz	431306	4313060	19883.5	4389.5	2709.3	3328.1	2029.8	7304

4869RS	Nova Pádua	431308	4313086	2524.8	557.4	344	422.6	257.7	354

4870RS	Nova Palma	431310	4313102	6499.1	1434.7	885.6	1087.8	663.5	3544

4871RS	Nova Petrópolis	431320	4313201	20217.6	4463.2	2754.9	3384	2063.9	4843

4872RS	Nova Prata	431330	4313300	24882.9	5493.1	3390.5	4164.9	2540.2	7658

4873RS	Nova Ramada	431333	4313334	2396	528.9	326.5	401	244.6	2133

Rio Grande

4874RS	43do Sul	Nova Roma do Sul	431335	4313359	3526.4	778.5	480.5	590.2	360	760

Rio Grande

4875RS	43do Sul	Nova Santa Rita	431337	4313375	25609.3	5653.5	3489.5	4286.5	2614.4	4006

Rio Grande

4876RS	43do Sul	Novo Cabrais	431339	4313391	4042.7	892.5	550.9	676.7	412.7	1343

Rio Grande

4877RS	43do Sul	Novo Hamburgo	431340	4313409	245399.3	54174.3	33438.1	41075	25051.8	51190	16

Rio Grande

4878RS	43do Sul	Novo Machado	431342	4313425	3746.1	827	510.4	627	382.4	2101

Rio Grande

4879RS	43do Sul	Novo Tiradentes	431344	4313441	2283.8	504.2	311.2	382.3	233.1	1823

Rio Grande

4880RS	43do Sul	Novo Xingu	431346	4313466	1767.4	390.2	240.8	295.8	180.4	1530

Rio Grande

4881RS	43do Sul	Novo Barreiro	431349	4313490	4107.8	906.8	559.7	687.6	419.3	1706

Rio Grande

4882RS	43do Sul	Osório	431350	4313508	43475.5	9597.7	5924	7276.9	4438.2	14461

Rio Grande

4883RS	43do Sul	Paim Filho	431360	4313607	4141.7	914.3	564.3	693.2	422.8	1450

Rio Grande

4884RS	43do Sul	Palmares do Sul	431365	4313656	11261.6	2486.1	1534.5	1885	1149.7	3606

Rio Grande Palmeira das

4885RS	43do Sul	Missões	431370	4313706	34418.7	7598.3	4689.9	5761	3513.7	8293

Rio Grande

4886RS	43do Sul	Palmitinho	431380	4313805	7072	1561.2	963.6	1183.7	722	3581

Rio Grande

4887RS	43do Sul	Panambi	431390	4313904	40789.7	9004.7	5558	6827.4	4164.1	10517

Rio Grande

4888RS	43do Sul	Pantano Grande	431395	4313953	9755.4	2153.6	1329.3	1632.9	995.9	1841

Rio Grande

4889RS	43do Sul	Paraí	431400	4314001	7238.9	1598.1	986.4	1211.6	739	2456

Rio Grande

4890RS	43do Sul	Paraíso do Sul	431402	4314027	7548.1	1666.3	1028.5	1263.4	770.6	2499

Rio Grande

4891RS	43do Sul	Pareci Novo	431403	4314035	3686.4	813.8	502.3	617	376.3	784

Rio Grande

4892RS	43do Sul	Parobé	431405	4314050	54928.1	12125.9	7484.5	9193.9	5607.4	10423

Rio Grande

4893RS	43do Sul	Passa Sete	431406	4314068	5437.1	1200.3	740.9	910.1	555.1	1632

Rio Grande

4894RS	43do Sul	Passo do Sobrado	431407	4314076	6301.4	1391.1	858.6	1054.7	643.3	1694

Rio Grande

3

1

1

1

1

4

2

1

4

3

1

1

4

1

2

4895RS	43do Sul	Passo Fundo	431410	4314100	194663.6	42973.9	26524.9	32582.8	19872.4	43931	14

Rio Grande

4896RS	43do Sul	Paulo Bento	431413	4314134	2266.5	500.4	308.8	379.4	231.4	2148

Rio Grande

4897RS	43do Sul	Paverama	431415	4314159	8332.2	1839.4	1135.3	1394.6	850.6	2229

Rio Grande

4898RS	43do Sul	Pedras Altas	431417	4314175	2153.5	475.4	293.4	360.5	219.8	670

Rio Grande

4899RS	43do Sul	Pedro Osório	431420	4314209	7890.7	1742	1075.2	1320.7	805.5	1602

Rio Grande

do Sul

4900RS	43	Pejuçara	431430	4314308	3991.6	881.2	543.9	668.1	407.5	2174

Rio Grande

4901RS	43do Sul	Pelotas	431440	4314407	338476.9	74722.2	46120.9	56654.3	34553.8	55105	15

Rio Grande

4902RS	43do Sul	Picada Café	431442	4314423	5475.1	1208.7	746	916.4	558.9	1935

Rio Grande

4903RS	43do Sul	Pinhal	431445	4314456	2574.4	568.3	350.8	430.9	262.8	2875

Rio Grande

4904RS	43do Sul	Pinhal da Serra	431446	4314464	2086.8	460.7	284.3	349.3	213	373

Rio Grande

4905RS	43do Sul	Pinhal Grande	431447	4314472	4487.9	990.7	611.5	751.2	458.2	2577

Rio Grande Pinheirinho do

4906RS	43do Sul	Vale	431449	4314498	4707.7	1039.3	641.5	788	480.6	2800

Rio Grande

4907RS	43do Sul	Pinheiro Machado	431450	4314506	12764.5	2817.9	1739.3	2136.5	1303.1	1607

Rio Grande

4908RS	43do Sul	Pirapó	431455	4314555	2619.7	578.3	357	438.5	267.4	1904

Rio Grande

4909RS	43do Sul	Piratini	431460	4314605	20445.1	4513.5	2785.8	3422.1	2087.2	3520

Rio Grande

4910RS	43do Sul	Planalto	431470	4314704	10502.8	2318.6	1431.1	1758	1072.2	5075

Rio Grande

4911RS	43do Sul	Poço das Antas	431475	4314753	2076.9	458.5	283	347.6	212	774

Rio Grande

4912RS	43do Sul	Pontão	431477	4314779	3930.2	867.6	535.5	657.8	401.2	1752

Rio Grande

4913RS	43do Sul	Ponte Preta	431478	4314787	1696	374.4	231.1	283.9	173.1	1421

Rio Grande

4914RS	43do Sul	Portão	431480	4314803	33764.2	7453.8	4600.7	5651.5	3446.9	13053

Rio Grande

4915RS	Porto Alegre	431490	4314902	1458515.6	321981.9	198737.3	244126.7	148894	371616	100

4916RS	Porto Lucena	431500	4315008	5203.7	1148.8	709.1	871	531.2	2921

4917RS	Porto Mauá	431505	4315057	2512.4	554.6	342.3	420.5	256.5	1708

4918RS	Porto Vera Cruz	431507	4315073	1686.6	372.3	229.8	282.3	172.2	1718

4919RS	Porto Xavier	431510	4315107	10587.1	2337.2	1442.6	1772.1	1080.8	4456

4920RS	Pouso Novo	431513	4315131	1809.7	399.5	246.6	302.9	184.7	555

4921RS	Presidente Lucena	431514	4315149	2682.8	592.3	365.6	449	273.9	1661

4922RS	Progresso	431515	4315156	6282.6	1386.9	856.1	1051.6	641.4	1990

4923RS	Protásio Alves	431517	4315172	2008.3	443.4	273.7	336.1	205	1208

4924RS	Putinga	431520	4315206	4114.2	908.3	560.6	688.6	420	1453

4925RS	Quaraí	431530	4315305	23219.9	5126	3163.9	3886.6	2370.4	5956

4926RS	Quatro Irmãos	431531	4315313	1827.1	403.4	249	305.8	186.5	913

4927RS	Quevedos	431532	4315321	2778.7	613.4	378.6	465.1	283.7	1239

Quinze de

4928RS	Novembro	431535	4315354	3761.3	830.3	512.5	629.6	384	745

4929RS	Redentora	431540	4315404	10915.9	2409.8	1487.4	1827.1	1114.4	2462

4930RS	Relvado	431545	4315453	2160.5	477	294.4	361.6	220.6	1342

4931RS	Restinga Seca	431550	4315503	16078.4	3549.5	2190.8	2691.2	1641.4	4714

4932RS	Rio dos Índios	431555	4315552	3330.8	735.3	453.9	557.5	340	2420

4933RS	Rio Grande	431560	4315602	205420.4	45348.6	27990.6	34383.3	20970.5	14257

4934RS	Rio Pardo	431570	4315701	38395.1	8476.1	5231.7	6426.6	3919.6	2910

4935RS	Riozinho	431575	4315750	4518.3	997.5	615.7	756.3	461.3	1520

Rio Grande

4936RS	43do Sul	Roca Sales	431580	4315800	10863.7	2398.3	1480.3	1818.4	1109	2952

Rio Grande

4937RS	43do Sul	Rodeio Bonito	431590	4315909	5873.5	1296.6	800.3	983.1	599.6	4121

Rio Grande

4938RS	43do Sul	Rolador	431595	4315958	2495.3	550.9	340	417.7	254.7	1893

Rio Grande

do Sul

4939RS	43	Rolante	431600	4316006	20490.4	4523.5	2792	3429.7	2091.8	7019

Rio Grande

4940RS	43do Sul	Ronda Alta	431610	4316105	10515.4	2321.4	1432.8	1760.1	1073.5	4130

Rio Grande

4941RS	43do Sul	Rondinha	431620	4316204	5445.9	1202.2	742.1	911.5	556	3691

Rio Grande

4942RS	43do Sul	Roque Gonzales	431630	4316303	7167.2	1582.2	976.6	1199.6	731.7	4424

Rio Grande

4943RS	43do Sul	Rosário do Sul	431640	4316402	40164.3	8866.7	5472.8	6722.7	4100.2	9437

Rio Grande

4944RS	43do Sul	Sagrada Família	431642	4316428	2637.3	582.2	359.4	441.4	269.2	913

Rio Grande

4945RS	43do Sul	Saldanha Marinho	431643	4316436	2824.4	623.5	384.9	472.7	288.3	1231

Rio Grande

4946RS	43do Sul	Salto do Jacuí	431645	4316451	12271.8	2709.1	1672.2	2054.1	1252.8	2871

Rio Grande Salvador das

4947RS	43do Sul	Missões	431647	4316477	2731.9	603.1	372.2	457.3	278.9	2038

Rio Grande

4948RS	43do Sul	Salvador do Sul	431650	4316501	7253.9	1601.4	988.4	1214.2	740.5	1982

Rio Grande

4949RS	43do Sul	Sananduva	431660	4316600	15942.3	3519.4	2172.3	2668.4	1627.5	6309

Rio Grande Santa Bárbara do

4950RS	43do Sul	Sul	431670	4316709	8629.1	1905	1175.8	1444.3	880.9	2804

Rio Grande Santa Cecília do

4951RS	43do Sul	Sul	431673	4316733	1672.2	369.2	227.9	279.9	170.7	1314

Rio Grande

4952RS	43do Sul	Santa Clara do Sul	431675	4316758	6131.7	1353.6	835.5	1026.3	626	1718

Rio Grande

4953RS	43do Sul	Santa Cruz do Sul	431680	4316808	124763.1	27542.7	17000.2	20882.9	12736.6	28029

Rio Grande

4954RS	43do Sul	Santa Maria	431690	4316907	272987.8	60264.8	37197.3	45692.8	27868.2	43618	13

Rio Grande Santa Maria do

4955RS	43do Sul	Herval	431695	4316956	6247.9	1379.3	851.3	1045.8	637.8	2755

Rio Grande Santa Margarida

4956RS	43do Sul	do Sul	431697	4316972	2467.8	544.8	336.3	413.1	251.9	854

1

4

1

2

1

1

3

1

1

1

5

3

6

3

1

1

2

7

1

1

7

1

Rio Grande Santana da Boa

4957RS	43do Sul	Vista	431700	4317004	8303.9	1833.2	1131.5	1389.9	847.7	1842

Rio Grande

4958RS	43do Sul	Santa Rosa	431720	4317202	71387.1	15759.4	9727.2	11948.8	7287.6	29251	10

Rio Grande

4959RS	43do Sul	Santa Tereza	431725	4317251	1752.8	386.9	238.8	293.4	178.9	946

Rio Grande Santa Vitória do

4960RS	43do Sul	Palmar	431730	4317301	30920	6825.9	4213.2	5175.4	3156.5	2045

Rio Grande

4961RS	43do Sul	Santiago	431740	4317400	49910.3	11018.2	6800.8	8354	5095.1	11668

Rio Grande

4962RS	43do Sul	Santo Ângelo	431750	4317509	77879.3	17192.6	10611.8	13035.5	7950.4	19190

| 4963RS | Rio Grande Santo Antônio do  43do Sul Palma | 431755 | 4317558 | 2165.4 | 478 | 295.1 | 362.4 | 221.1 | 1538 |
| --- | --- | --- | --- | --- | --- | --- | --- | --- | --- |
| 4964RS | Rio Grande Santo Antônio da  43do Sul Patrulha | 431760 | 4317608 | 41503.4 | 9162.3 | 5655.3 | 6946.8 | 4236.9 | 12745 |
| 4965RS | Rio Grande Santo Antônio das  43do Sul Missões | 431770 | 4317707 | 10966.2 | 2420.9 | 1494.3 | 1835.5 | 1119.5 | 4329 |
| 4966RS | Rio Grande Santo Antônio do  43do Sul Planalto  Rio Grande | 431775 | 4317756 | 2027.8 | 447.7 | 276.3 | 339.4 | 207 | 1199 |

4967RS	43do Sul	Santo Augusto	431780	4317806	14135.4	3120.5	1926.1	2366	1443	4852

Rio Grande

4968RS	43do Sul	Santo Cristo	431790	4317905	14536.2	3209	1980.7	2433.1	1483.9	8732

Rio Grande Santo Expedito do

4969RS	43do Sul	Sul	431795	4317954	2442.7	539.3	332.8	408.9	249.4	1577

Rio Grande

4970RS	43do Sul	São Borja	431800	4318002	62010.3	13689.4	8449.5	10379.3	6330.4	21964

| 4971RS | Rio Grande São Domingos do  43do Sul Sul | 431805 | 4318051 | 3025.8 | 668 | 412.3 | 506.5 | 308.9 | 1710 |
| --- | --- | --- | --- | --- | --- | --- | --- | --- | --- |
| 4972RS | Rio Grande São Francisco de  43do Sul Assis | 431810 | 4318101 | 19170.6 | 4232.1 | 2612.2 | 3208.8 | 1957 | 5655 |
| 4973RS | Rio Grande São Francisco de  43do Sul Paula  Rio Grande | 431820 | 4318200 | 21287.6 | 4699.5 | 2900.6 | 3563.1 | 2173.2 | 3460 |

4974RS	43do Sul	São Gabriel	431830	4318309	61942.3	13674.4	8440.3	10367.9	6323.4	21327

Rio Grande

4975RS	43do Sul	São Jerônimo	431840	4318408	23275.1	5138.2	3171.5	3895.8	2376.1	5597

Rio Grande São João da

4976RS	43do Sul	Urtiga	431842	4318424	4766.7	1052.3	649.5	797.9	486.6	2309

Rio Grande São João do

4977RS	43do Sul	Polêsine	431843	4318432	2620.4	578.5	357.1	438.6	267.5	1215

Rio Grande

do Sul

4978RS	43	São Jorge	431844	4318440	2811	620.6	383	470.5	287	1743

Rio Grande São José das

4979RS	43do Sul	Missões	431845	4318457	2688	593.4	366.3	449.9	274.4	1381

Rio Grande São José do

4980RS	43do Sul	Herval	431846	4318465	2144.3	473.4	292.2	358.9	218.9	640

Rio Grande São José do

4981RS	43do Sul	Hortêncio	431848	4318481	4428.8	977.7	603.5	741.3	452.1	2219

Rio Grande São José do

4982RS	43do Sul	Inhacorá	431849	4318499	2182	481.7	297.3	365.2	222.8	1895

Rio Grande

4983RS	43do Sul	São José do Norte	431850	4318507	26672.8	5888.3	3634.4	4464.5	2722.9	1775

Rio Grande

4984RS	43do Sul	São José do Ouro	431860	4318606	7013.9	1548.4	955.7	1174	716	3254

Rio Grande

4985RS	43do Sul	São José do Sul	431861	4318614	2240.2	494.5	305.2	375	228.7	712

Rio Grande São José dos

4986RS	43do Sul	Ausentes	431862	4318622	3429.5	757.1	467.3	574	350.1	710

4987RS	São Leopoldo	431870	4318705	226019.2	49896	30797.4	37831.1	23073.4	54256	16

São Lourenço do

4988RS	Sul	431880	4318804	43910.8	9693.7	5983.3	7349.8	4482.7	13808

4989RS	São Luiz Gonzaga	431890	4318903	34630.3	7645	4718.7	5796.4	3535.3	19784

4990RS	São Marcos	431900	4319000	20957	4626.5	2855.6	3507.8	2139.4	2292

4991RS	São Martinho	431910	4319109	5720.6	1262.9	779.5	957.5	584	2639

São Martinho da

4992RS	Serra	431912	4319125	3259.7	719.6	444.2	545.6	332.8	1504

São Miguel das

4993RS	Missões	431915	4319158	7625.7	1683.4	1039.1	1276.4	778.5	3237

4994RS	São Nicolau	431920	4319208	5628.6	1242.6	767	942.1	574.6	2113

São Paulo das

4995RS	Missões	431930	4319307	6230.9	1375.5	849	1042.9	636.1	2939

São Pedro da

4996RS	Serra	431935	4319356	3552.3	784.2	484	594.6	362.6	881

Rio Grande São Pedro das

4997RS	43do Sul	Missões	431936	4319364	1960.7	432.8	267.2	328.2	200.2	541

Rio Grande São Pedro do

4998RS	43do Sul	Butiá	431937	4319372	2943	649.7	401	492.6	300.4	2573

Rio Grande

4999RS	43do Sul	São Pedro do Sul	431940	4319406	16535.6	3650.4	2253.1	2767.7	1688.1	4817

Rio Grande São Sebastião do

5000RS	43do Sul	Caí	431950	4319505	23957	5288.7	3264.4	4009.9	2445.7	4812

Rio Grande

5001RS	43do Sul	São Sepé	431960	4319604	24068.4	5313.3	3279.6	4028.6	2457	6366

Rio Grande

5002RS	43do Sul	São Valentim	431970	4319703	3553.6	784.5	484.2	594.8	362.8	1304

Rio Grande São Valentim do

5003RS	43do Sul	Sul	431971	4319711	2227.7	491.8	303.5	372.9	227.4	1140

Rio Grande

5004RS	43do Sul	São Valério do Sul	431973	4319737	2715.7	599.5	370	454.6	277.2	1679

Rio Grande

5005RS	43do Sul	São Vendelino	431975	4319752	2094.1	462.3	285.3	350.5	213.8	734

Rio Grande São Vicente do

5006RS	43do Sul	Sul	431980	4319802	8670.6	1914.1	1181.5	1451.3	885.1	2098

Rio Grande

5007RS	43do Sul	Sapiranga	431990	4319901	78689.5	17371.5	10722.2	13171.1	8033.1	25136

Rio Grande

5008RS	43do Sul	Sapucaia do Sul	432000	4320008	136774.7	30194.4	18636.9	22893.4	13962.8	35858

Rio Grande

5009RS	43do Sul	Sarandi	432010	4320107	22838.9	5041.9	3112	3822.8	2331.5	7066

Rio Grande

5010RS	43do Sul	Seberi	432020	4320206	10995.2	2427.3	1498.2	1840.4	1122.5	4953

Rio Grande

5011RS	43do Sul	Sede Nova	432023	4320230	3013.8	665.3	410.7	504.5	307.7	1192

Rio Grande

5012RS	43do Sul	Segredo	432026	4320263	7317.6	1615.4	997.1	1224.8	747	2208

Rio Grande

5013RS	43do Sul	Selbach	432030	4320305	5066.3	1118.4	690.3	848	517.2	1594

Rio Grande Senador Salgado

5014RS	43do Sul	Filho	432032	4320321	2840.7	627.1	387.1	475.5	290	1567

Rio Grande

5015RS	43do Sul	Sentinela do Sul	432035	4320354	5422	1197	738.8	907.5	553.5	1559

Rio Grande

5016RS	43do Sul	Serafina Corrêa	432040	4320404	15724.2	3471.3	2142.6	2631.9	1605.2	9442

Rio Grande

do Sul

5017RS	43	Sério	432045	4320453	2189	483.2	298.3	366.4	223.5	917

4

6

1

1

2

9

9

2

1

3

1

3

1

4

2

1

2

4

2

2

1

3

Rio Grande

5018RS	43do Sul	Sertão	432050	4320503	6041	1333.6	823.1	1011.1	616.7	2350

Rio Grande

5019RS	43do Sul	Sertão Santana	432055	4320552	6181.9	1364.7	842.3	1034.7	631.1	1319

Rio Grande

5020RS	43do Sul	Sete de Setembro	432057	4320578	2094.3	462.3	285.4	350.5	213.8	1598

Rio Grande Severiano de

5021RS	43do Sul	Almeida	432060	4320602	3825.2	844.5	521.2	640.3	390.5	1618

Rio Grande

5022RS	43do Sul	Silveira Martins	432065	4320651	2452.1	541.3	334.1	410.4	250.3	1076

Rio Grande

5023RS	43do Sul	Sinimbu	432067	4320677	10252.4	2263.3	1397	1716	1046.6	1891

Rio Grande

5024RS	43do Sul	Sobradinho	432070	4320701	14754.8	3257.3	2010.5	2469.7	1506.3	4037

Rio Grande

5025RS	43do Sul	Soledade	432080	4320800	30846.5	6809.7	4203.1	5163.1	3149	3715

Rio Grande

5026RS	43do Sul	Tabaí	432085	4320859	4419.8	975.7	602.2	739.8	451.2	1242

Rio Grande

5027RS	43do Sul	Tapejara	432090	4320909	21420.9	4728.9	2918.8	3585.4	2186.8	11981

Rio Grande

5028RS	43do Sul	Tapera	432100	4321006	10650.6	2351.2	1451.3	1782.7	1087.3	2800

Rio Grande

5029RS	43do Sul	Tapes	432110	4321105	17128.8	3781.4	2334	2867	1748.6	3449

Rio Grande

5030RS	43do Sul	Taquara	432120	4321204	56524.8	12478.4	7702.1	9461.1	5770.4	12487

Rio Grande

5031RS	43do Sul	Taquari	432130	4321303	26764.9	5908.6	3647	4479.9	2732.3	6180

Rio Grande

5032RS	43do Sul	Taquaruçu do Sul	432132	4321329	3049.7	673.3	415.6	510.5	311.3	2687

Rio Grande

5033RS	43do Sul	Tavares	432135	4321352	5478.4	1209.4	746.5	917	559.3	1551

Rio Grande

5034RS	43do Sul	Tenente Portela	432140	4321402	13823.9	3051.8	1883.6	2313.8	1411.2	6015

Rio Grande

5035RS	43do Sul	Terra de Areia	432143	4321436	10536.3	2326	1435.7	1763.6	1075.6	2826

Rio Grande

5036RS	43do Sul	Teutônia	432145	4321451	29986.8	6619.9	4086	5019.2	3061.2	7941

Rio Grande

5037RS	43do Sul	Tio Hugo	432146	4321469	2882.6	636.4	392.8	482.5	294.3	1210

Rio Grande

5038RS	Tiradentes do Sul	432147	4321477	6257.9	1381.5	852.7	1047.4	638.8	1900

5039RS	Toropi	432149	4321493	2937.4	648.5	400.3	491.7	299.9	1098

5040RS	Torres	432150	4321501	36741.4	8111	5006.4	6149.8	3750.8	14262

5041RS	Tramandaí	432160	4321600	46130.7	10183.8	6285.8	7721.4	4709.3	10409

5042RS	Travesseiro	432162	4321626	2355.4	520	320.9	394.2	240.5	1000

5043RS	Três Arroios	432163	4321634	2823	623.2	384.7	472.5	288.2	1130

5044RS	Três Cachoeiras	432166	4321667	10689.3	2359.8	1456.5	1789.2	1091.2	5767

5045RS	Três Coroas	432170	4321709	25899.4	5717.6	3529.1	4335	2644	4319

5046RS	Três de Maio	432180	4321808	24135.2	5328.1	3288.7	4039.8	2463.9	9976

5047RS	Três Forquilhas	432183	4321832	2871.3	633.9	391.2	480.6	293.1	1726

5048RS	Três Palmeiras	432185	4321857	4400.8	971.5	599.7	736.6	449.3	1692

5049RS	Três Passos	432190	4321907	24284.7	5361.1	3309	4064.8	2479.1	8098

5050RS	Trindade do Sul	432195	4321956	5875.3	1297	800.6	983.4	599.8	2448

5051RS	Triunfo	432200	4322004	27622.2	6097.9	3763.8	4623.4	2819.8	4263

5052RS	Tucunduva	432210	4322103	5896.2	1301.6	803.4	986.9	601.9	2822

5053RS	Tunas	432215	4322152	4526	999.2	616.7	757.6	462	1448

5054RS	Tupanci do Sul	432218	4322186	1556.6	343.6	212.1	260.5	158.9	997

5055RS	Tupanciretã	432220	4322202	23249.5	5132.6	3168	3891.5	2373.4	4250

5056RS	Tupandi	432225	4322251	4343	958.8	591.8	726.9	443.4	574

5057RS	Tuparendi	432230	4322301	8420.1	1858.8	1147.3	1409.4	859.6	4650

5058RS	Turuçu	432232	4322327	3539.7	781.4	482.3	592.5	361.4	1129

Rio Grande

5059RS	43do Sul	Ubiretama	432234	4322343	2219.6	490	302.4	371.5	226.6	1139

Rio Grande

5060RS	43do Sul	União da Serra	432235	4322350	1378.3	304.3	187.8	230.7	140.7	841

Rio Grande

5061RS	43do Sul	Unistalda	432237	4322376	2440.6	538.8	332.6	408.5	249.2	906

Rio Grande

5062RS	43do Sul	Uruguaiana	432240	4322400	127825.3	28218.7	17417.5	21395.4	13049.2	29005	11

Rio Grande

5063RS	43do Sul	Vacaria	432250	4322509	64120.7	14155.3	8737.1	10732.5	6545.8	15514

Rio Grande

5064RS	43do Sul	Vale Verde	432252	4322525	3394.7	749.4	462.6	568.2	346.6	844

Rio Grande

5065RS	43do Sul	Vale do Sol	432253	4322533	11510	2540.9	1568.4	1926.5	1175	2584

Rio Grande

5066RS	43do Sul	Vale Real	432254	4322541	5501.3	1214.5	749.6	920.8	561.6	1288

Rio Grande

5067RS	43do Sul	Vanini	432255	4322558	2062.4	455.3	281	345.2	210.5	1094

Rio Grande

5068RS	43do Sul	Venâncio Aires	432260	4322608	69077.7	15249.6	9412.5	11562.2	7051.9	14166

Rio Grande

5069RS	43do Sul	Vera Cruz	432270	4322707	25449.5	5618.2	3467.7	4259.7	2598	6149

Rio Grande

5070RS	43do Sul	Veranópolis	432280	4322806	24474.1	5402.9	3334.8	4096.5	2498.5	7276

Rio Grande Vespasiano

5071RS	43do Sul	Correa	432285	4322855	1940	428.3	264.3	324.7	198	851

Rio Grande

5072RS	43do Sul	Viadutos	432290	4322905	5170	1141.3	704.5	865.4	527.8	1963

Rio Grande

5073RS	43do Sul	Viamão	432300	4323002	248982.9	54965.5	33926.4	41674.8	25417.7	46671	14

Rio Grande

5074RS	43do Sul	Vicente Dutra	432310	4323101	5121	1130.5	697.8	857.2	522.8	1540

Rio Grande

5075RS	43do Sul	Victor Graeff	432320	4323200	3018.8	666.4	411.3	505.3	308.2	2233

Rio Grande

5076RS	43do Sul	Vila Flores	432330	4323309	3322	733.4	452.7	556	339.1	1395

Rio Grande

5077RS	43do Sul	Vila Lângaro	432335	4323358	2158.7	476.6	294.1	361.3	220.4	1946

Rio Grande

5078RS	43do Sul	Vila Maria	432340	4323408	4334.8	957	590.7	725.6	442.5	1235

Rio Grande

5079RS	43do Sul	Vila Nova do Sul	432345	4323457	4304.1	950.2	586.5	720.4	439.4	704

Rio Grande

2

1

1

1

5

1

4

2

2

1

1

1

1

4

5080RS	43do Sul	Vista Alegre	432350	4323507	2841.5	627.3	387.2	475.6	290.1	2302

Rio Grande Vista Alegre do

5081RS	43do Sul	Prata	432360	4323606	1588.5	350.7	216.4	265.9	162.2	717

Rio Grande

5082RS	43do Sul	Vista Gaúcha	432370	4323705	2834.5	625.7	386.2	474.4	289.4	2028

Rio Grande Vitória das

5083RS	43do Sul	Missões	432375	4323754	3397.6	750.1	463	568.7	346.8	1663

Rio Grande

5084RS	43do Sul	Westfalia	432377	4323770	2919.3	644.5	397.8	488.6	298	945

Rio Grande

5085RS	43do Sul	Xangri-lá	432380	4323804	14161.7	3126.3	1929.7	2370.4	1445.7	4577

Mato Grosso

5086MS	50do Sul	Água Clara	500020	5000203	14717.6	2514.2	1981.9	1782.6	1472.7	792

Mato Grosso

5087MS	50do Sul	Alcinópolis	500025	5000252	4970.5	849.1	669.3	602	497.4	456

Mato Grosso

5088MS	50do Sul	Anastácio	500070	5000708	24580.8	4199.1	3310.1	2977.2	2459.7	1355

Mato Grosso

5089MS	50do Sul	Anaurilândia	500080	5000807	8784.5	1500.7	1182.9	1064	879	1366

Mato Grosso

5090MS	50do Sul	Angélica	500085	5000856	10010.3	1710.1	1348	1212.4	1001.7	1530

Mato Grosso

5091MS	50do Sul	Antônio João	500090	5000906	8604.1	1469.8	1158.6	1042.1	861	593

Mato GrossoAparecida do

5092MS	50do Sul	Taboado	500100	5001003	24107.1	4118.2	3246.3	2919.8	2412.3	3660

Mato Grosso

5093MS	50do Sul	Aquidauana	500110	5001102	46878.8	8008.3	6312.7	5677.9	4691	3387

Mato Grosso

5094MS	50do Sul	Aral Moreira	500124	5001243	11234.5	1919.2	1512.8	1360.7	1124.2	701

Mato Grosso do Sul

5095MS	50	Bandeirantes	500150	5001508	6734.1	1150.4	906.8	815.6	673.9	536

Mato Grosso

5096MS	50do Sul	Bataguassu	500190	5001904	21494.1	3671.8	2894.4	2603.3	2150.8	712

Mato Grosso

5097MS	50do Sul	Batayporã	500200	5002001	11155.2	1905.6	1502.2	1351.1	1116.3	2337

Mato Grosso

5098MS	50do Sul	Bela Vista	500210	5002100	23956	4092.4	3225.9	2901.5	2397.2	884

Mato Grosso

5099MS	50do Sul	Bodoquena	500215	5002159	7905.5	1350.5	1064.6	957.5	791.1	547

Mato Grosso

5100MS	50do Sul	Bonito	500220	5002209	20829.4	3558.3	2804.9	2522.8	2084.3	1132

Mato Grosso

5101MS	50do Sul	Brasilândia	500230	5002308	11872.9	2028.2	1598.8	1438	1188.1	1204

Mato Grosso

5102MS	50do Sul	Caarapó	500240	5002407	28052.2	4792.2	3777.5	3397.6	2807.1	2095

Mato Grosso

5103MS	50do Sul	Camapuã	500260	5002605	13694.6	2339.4	1844.1	1658.7	1370.4	902

Mato Grosso

5104MS	50do Sul	Campo Grande	500270	5002704	843742.5	144136.5	113618.4	102192.8	84429.8	194603	76

Mato Grosso

5105MS	50do Sul	Caracol	500280	5002803	5772.7	986.2	777.4	699.2	577.7	196

Mato Grosso

5106MS	50do Sul	Cassilândia	500290	5002902	21501.6	3673.1	2895.4	2604.2	2151.6	3038

Mato Grosso

5107MS	50do Sul	Chapadão do Sul	500295	5002951	22359	3819.6	3010.9	2708.1	2237.4	2579

Mato Grosso

5108MS	50do Sul	Corguinho	500310	5003108	5421.8	926.2	730.1	656.7	542.5	401

Mato Grosso

5109MS	50do Sul	Coronel Sapucaia	500315	5003157	14694.9	2510.3	1978.8	1779.8	1470.5	818

Mato Grosso

5110MS	50do Sul	Corumbá	500320	5003207	107837.9	18421.9	14521.5	13061.2	10790.9	10944

| 5111MS | Mato Grosso  50do Sul Costa Rica | 500325 | 5003256 | 19890.1 | 3397.8 | 2678.4 | 2409.1 | 1990.3 | 4293 |
| --- | --- | --- | --- | --- | --- | --- | --- | --- | --- |
| 5112MS | Mato Grosso  50do Sul Coxim | 500330 | 5003306 | 32951.7 | 5629.1 | 4437.3 | 3991.1 | 3297.3 | 1600 |
| 5113MS | Mato Grosso  50do Sul Deodápolis | 500345 | 5003454 | 12563.7 | 2146.3 | 1691.8 | 1521.7 | 1257.2 | 1487 |
| 5114MS | Mato GrossoDois Irmãos do  50do Sul Buriti | 500348 | 5003488 | 10876.7 | 1858.1 | 1464.7 | 1317.4 | 1088.4 | 1089 |
| 5115MS | Mato Grosso  50do Sul Douradina | 500350 | 5003504 | 5666.1 | 967.9 | 763 | 686.3 | 567 | 562 |
| 5116MS | Mato Grosso  50do Sul Dourados | 500370 | 5003702 | 210324 | 35929.6 | 28322.2 | 25474.1 | 21046.2 | 19038 |
| 5117MS | Mato Grosso  50do Sul Eldorado | 500375 | 5003751 | 12052.1 | 2058.9 | 1622.9 | 1459.7 | 1206 | 2386 |
| 5118MS | Mato Grosso  50do Sul Fátima do Sul | 500380 | 5003801 | 19161.2 | 3273.3 | 2580.2 | 2320.8 | 1917.4 | 6398 |
| 5119MS | Mato Grosso  50do Sul Figueirão | 500390 | 5003900 | 2996.5 | 511.9 | 403.5 | 362.9 | 299.8 | 514 |
| 5120MS | Mato GrossoGlória de  50do Sul Dourados | 500400 | 5004007 | 9966.4 | 1702.6 | 1342.1 | 1207.1 | 997.3 | 1895 |
| 5121MS | Mato GrossoGuia Lopes da  50do Sul Laguna | 500410 | 5004106 | 10147.9 | 1733.6 | 1366.5 | 1229.1 | 1015.5 | 526 |
| 5122MS | Mato Grosso  50do Sul Iguatemi | 500430 | 5004304 | 15514.3 | 2650.3 | 2089.2 | 1879.1 | 1552.5 | 2331 |
| 5123MS | Mato Grosso  50do Sul Inocência | 500440 | 5004403 | 7651.7 | 1307.1 | 1030.4 | 926.8 | 765.7 | 1080 |
| 5124MS | Mato Grosso  50do Sul Itaporã | 500450 | 5004502 | 22664.4 | 3871.8 | 3052 | 2745.1 | 2267.9 | 1940 |
| 5125MS | Mato Grosso 50do Sul Itaquiraí | 500460 | 5004601 | 19932.9 | 3405.1 | 2684.2 | 2414.2 | 1994.6 | 1917 |
| 5126MS | Mato Grosso  50do Sul Ivinhema | 500470 | 5004700 | 22814.7 | 3897.4 | 3072.2 | 2763.3 | 2283 | 4210 |
| 5127MS | Mato Grosso  50do Sul Japorã | 500480 | 5004809 | 8446.4 | 1442.9 | 1137.4 | 1023 | 845.2 | 1615 |
| 5128MS | Mato Grosso  50do Sul Jaraguari | 500490 | 5004908 | 6782.9 | 1158.7 | 913.4 | 821.5 | 678.7 | 407 |
| 5129MS | Mato Grosso  50do Sul Jardim | 500500 | 5005004 | 25286.9 | 4319.8 | 3405.1 | 3062.7 | 2530.4 | 1498 |
| 5130MS | Mato Grosso  50do Sul Jateí | 500510 | 5005103 | 4027.4 | 688 | 542.3 | 487.8 | 403 | 882 |
| 5131MS | Mato Grosso  50do Sul Juti | 500515 | 5005152 | 6325 | 1080.5 | 851.7 | 766.1 | 632.9 | 866 |
| 5132MS | Mato Grosso  50do Sul Ladário | 500520 | 5005202 | 21540.2 | 3679.7 | 2900.6 | 2608.9 | 2155.4 | 1391 |
| 5133MS | Mato Grosso  50do Sul Laguna Carapã | 500525 | 5005251 | 6937.7 | 1185.2 | 934.2 | 840.3 | 694.2 | 532 |
| 5134MS | Mato Grosso do Sul  50 Maracaju | 500540 | 5005400 | 42309.3 | 7227.7 | 5697.4 | 5124.4 | 4233.7 | 1811 |
| 5135MS | Mato Grosso  50do Sul Miranda | 500560 | 5005608 | 26868.8 | 4590 | 3618.2 | 3254.3 | 2688.6 | 1537 |
| 5136MS | Mato Grosso  50do Sul Mundo Novo | 500568 | 5005681 | 17747.6 | 3031.8 | 2389.9 | 2149.6 | 1775.9 | 3317 |
| 5137MS | Mato Grosso  50do Sul Naviraí | 500570 | 5005707 | 50803.4 | 8678.7 | 6841.2 | 6153.2 | 5083.7 | 6430 |
| 5138MS | Mato Grosso  50do Sul Nioaque | 500580 | 5005806 | 14220.2 | 2429.2 | 1914.9 | 1722.3 | 1423 | 592 |
| 5139MS | Mato GrossoNova Alvorada do  50do Sul Sul | 500600 | 5006002 | 19218.7 | 3283.1 | 2588 | 2327.7 | 1923.1 | 949 |
| 5140MS | Mato Grosso  50do Sul Nova Andradina | 500620 | 5006200 | 50136.6 | 8564.8 | 6751.4 | 6072.5 | 5017 | 14550 |
| 5141MS | Mato GrossoNovo Horizonte do  50do Sul Sul | 500625 | 5006259 | 4378.9 | 748 | 589.7 | 530.4 | 438.2 | 1229 |
| 5142MS | Mato Grosso  50do Sul Paranaíba | 500630 | 5006309 | 41260.7 | 7048.6 | 5556.2 | 4997.4 | 4128.8 | 12209 |
| 5143MS | Mato Grosso  50do Sul Paranhos | 500635 | 5006358 | 13326.7 | 2276.6 | 1794.6 | 1614.1 | 1333.5 | 1020 |
| 5144MS | Mato Grosso  50do Sul Pedro Gomes | 500640 | 5006408 | 7808.5 | 1333.9 | 1051.5 | 945.8 | 781.4 | 363 |
| 5145MS | Mato Grosso  50do Sul Ponta Porã | 500660 | 5006606 | 85455.7 | 14598.4 | 11507.5 | 10350.3 | 8551.2 | 4657 |
| 5146MS | Mato Grosso  50do Sul Porto Murtinho | 500690 | 5006903 | 16337.5 | 2790.9 | 2200 | 1978.8 | 1634.8 | 241 |
| 5147MS | Mato GrossoRibas do Rio  50do Sul Pardo | 500710 | 5007109 | 22847.9 | 3903.1 | 3076.7 | 2767.3 | 2286.3 | 1510 |
| 5148MS | Mato Grosso  50do Sul Rio Brilhante | 500720 | 5007208 | 34198.6 | 5842.1 | 4605.2 | 4142.1 | 3422.1 | 2458 |
| 5149MS | Mato Grosso  50do Sul Rio Negro | 500730 | 5007307 | 4922.2 | 840.9 | 662.8 | 596.2 | 492.5 | 329 |
| 5150MS | Mato GrossoRio Verde de Mato  50do Sul Grosso | 500740 | 5007406 | 19357.4 | 3306.8 | 2606.7 | 2344.5 | 1937 | 479 |
| 5151MS | Mato Grosso  50do Sul Rochedo | 500750 | 5007505 | 5207.6 | 889.6 | 701.3 | 630.7 | 521.1 | 867 |
| 5152MS | Mato GrossoSanta Rita do  50do Sul Pardo | 500755 | 5007554 | 7573 | 1293.7 | 1019.8 | 917.2 | 757.8 | 661 |
| 5153MS | Mato GrossoSão Gabriel do  50do Sul Oeste | 500769 | 5007695 | 24584.2 | 4199.7 | 3310.5 | 2977.6 | 2460 | 1828 |
| 5154MS | Mato Grosso  50do Sul Sete Quedas | 500770 | 5007703 | 10807.1 | 1846.2 | 1455.3 | 1308.9 | 1081.4 | 2136 |
| 5155MS | Mato Grosso  50do Sul Selvíria | 500780 | 5007802 | 6422.6 | 1097.2 | 864.9 | 777.9 | 642.7 | 1328 |
| 5156MS | Mato Grosso  50do Sul Sidrolândia | 500790 | 5007901 | 50103.4 | 8559.2 | 6746.9 | 6068.4 | 5013.6 | 2600 |
| 5157MS | Mato Grosso  50do Sul Sonora | 500793 | 5007935 | 17118.5 | 2924.4 | 2305.2 | 2073.4 | 1713 | 476 |
| 5158MS | Mato Grosso  50do Sul Tacuru | 500795 | 5007950 | 10912.9 | 1864.3 | 1469.5 | 1321.8 | 1092 | 2021 |
| 5159MS | Mato Grosso  50do Sul Taquarussu | 500797 | 5007976 | 3558.1 | 607.8 | 479.1 | 431 | 356 | 901 |
| 5160MS | Mato Grosso  50do Sul Terenos | 500800 | 5008008 | 19533.7 | 3336.9 | 2630.4 | 2365.9 | 1954.7 | 1504 |
| 5161MS | Mato Grosso  50do Sul Três Lagoas | 500830 | 5008305 | 111934.6 | 19121.8 | 15073.1 | 13557.3 | 11200.8 | 17495 |
| 5162MS | Mato Grosso  50do Sul Vicentina | 500840 | 5008404 | 6003.5 | 1025.6 | 808.4 | 727.1 | 600.7 | 1345 |
| 5163MT | 51Mato GrossoAcorizal | 510010 | 5100102 | 5406.2 | 818.3 | 732.1 | 660.5 | 598.7 | 617 |
| 5164MT | 51Mato GrossoÁgua Boa | 510020 | 5100201 | 23259.9 | 3520.6 | 3149.6 | 2841.8 | 2576.1 | 2962 |
| 5165MT | 51Mato GrossoAlta Floresta | 510025 | 5100250 | 50128.6 | 7587.5 | 6787.9 | 6124.6 | 5551.8 | 2583 |
| 5166MT | 51Mato GrossoAlto Araguaia | 510030 | 5100300 | 17314.4 | 2620.7 | 2344.5 | 2115.4 | 1917.6 | 538 |
| 5167MT | 51Mato GrossoAlto Boa Vista | 510035 | 5100359 | 6039.2 | 914.1 | 817.8 | 737.9 | 668.9 | 83 |
| 5168MT | 51Mato GrossoAlto Garças | 510040 | 5100409 | 11153.2 | 1688.1 | 1510.3 | 1362.7 | 1235.2 | 260 |
| 5169MT | 51Mato GrossoAlto Paraguai | 510050 | 5100508 | 10663.7 | 1614.1 | 1444 | 1302.9 | 1181 | 348 |
| 5170MT | 51Mato GrossoAlto Taquari | 510060 | 5100607 | 9476.8 | 1434.4 | 1283.3 | 1157.9 | 1049.6 | 459 |
| 5171MT | 51Mato GrossoApiacás | 510080 | 5100805 | 9323.5 | 1411.2 | 1262.5 | 1139.1 | 1032.6 | 374 |
| 5172MT | 51Mato GrossoAraguaiana | 510100 | 5101001 | 3117.7 | 471.9 | 422.2 | 380.9 | 345.3 | 305 |
| 5173MT | 51Mato GrossoAraguainha | 510120 | 5101209 | 1000.5 | 151.4 | 135.5 | 122.2 | 110.8 | 52 |
| 5174MT | 51Mato GrossoAraputanga | 510125 | 5101258 | 16002.6 | 2422.2 | 2166.9 | 1955.2 | 1772.3 | 150 |
| 5175MT | 51Mato GrossoArenápolis | 510130 | 5101308 | 9848.6 | 1490.7 | 1333.6 | 1203.3 | 1090.8 | 893 |
| 5176MT | 51Mato GrossoAripuanã | 510140 | 5101407 | 20458.4 | 3096.6 | 2770.3 | 2499.6 | 2265.8 | 240 |
| 5177MT | 51Mato GrossoBarão de Melgaço | 510160 | 5101605 | 7827.4 | 1184.8 | 1059.9 | 956.3 | 866.9 | 402 |
| 5178MT | 51Mato GrossoBarra do Bugres | 510170 | 5101704 | 33304.1 | 5040.9 | 4509.7 | 4069 | 3688.5 | 1653 |
| 5179MT | 51Mato GrossoBarra do Garças | 510180 | 5101803 | 58432.3 | 8844.3 | 7912.3 | 7139.1 | 6471.5 | 6439 |

1

4

2

1

1

2

6

4

2

1

6

1

2

| 5180MT | Bom Jesus do  51Mato GrossoAraguaia | 510185 | 5101852 | 5941 | 899.2 | 804.5 | 725.9 | 658 | 251 |  |
| --- | --- | --- | --- | --- | --- | --- | --- | --- | --- | --- |
| 5181MT | 51Mato GrossoBrasnorte | 510190 | 5101902 | 17529.3 | 2653.2 | 2373.6 | 2141.7 | 1941.4 | 702 |  |
| 5182MT | 51Mato GrossoCáceres | 510250 | 5102504 | 90598.3 | 13713 | 12267.9 | 11069.1 | 10033.9 | 890 |  |
| 5183MT | 51Mato GrossoCampinápolis | 510260 | 5102603 | 15070.3 | 2281 | 2040.7 | 1841.3 | 1669.1 | 334 |  |
| 5184MT | Campo Novo do  51Mato GrossoParecis | 510263 | 5102637 | 31473.3 | 4763.8 | 4261.8 | 3845.3 | 3485.7 | 2334 |  |
| 5185MT | 51Mato GrossoCampo Verde | 510267 | 5102678 | 37406.6 | 5661.9 | 5065.2 | 4570.3 | 4142.8 | 2691 |  |
| 5186MT | 51Mato GrossoCampos de Júlio | 510268 | 5102686 | 6032.6 | 913.1 | 816.9 | 737 | 668.1 | 208 |  |
| 5187MT | Canabrava do  51Mato GrossoNorte | 510269 | 5102694 | 4720.9 | 714.6 | 639.3 | 576.8 | 522.8 | 14 |  |
| 5188MT | 51Mato GrossoCanarana | 510270 | 5102702 | 20092 | 3041.1 | 2720.7 | 2454.8 | 2225.2 | 962 |  |
| 5189MT | 51Mato GrossoCarlinda | 510279 | 5102793 | 10526.8 | 1593.3 | 1425.4 | 1286.1 | 1165.9 | 458 |  |
| 5190MT | 51Mato GrossoCastanheira | 510285 | 5102850 | 8423.8 | 1275 | 1140.7 | 1029.2 | 933 | 42 |  |
| 5191MT | Chapada dos  51Mato GrossoGuimarães | 510300 | 5103007 | 18686.9 | 2828.4 | 2530.4 | 2283.1 | 2069.6 | 1792 |  |
| 5192MT | 51Mato GrossoCláudia | 510305 | 5103056 | 11528.1 | 1744.9 | 1561 | 1408.5 | 1276.8 | 721 |  |
| 5193MT | 51Mato GrossoCocalinho | 510310 | 5103106 | 5554.1 | 840.7 | 752.1 | 678.6 | 615.1 | 308 |  |
| 5194MT | 51Mato GrossoColíder | 510320 | 5103205 | 31911.3 | 4830.1 | 4321.1 | 3898.9 | 3534.2 | 1709 |  |
| 5195MT | 51Mato GrossoColniza | 510325 | 5103254 | 32636.8 | 4939.9 | 4419.3 | 3987.5 | 3614.6 | 106 |  |
| 5196MT | 51Mato GrossoComodoro | 510330 | 5103304 | 19403.2 | 2936.9 | 2627.4 | 2370.6 | 2148.9 | 239 |  |
| 5197MT | 51Mato GrossoConfresa | 510335 | 5103353 | 27992.7 | 4237 | 3790.5 | 3420.1 | 3100.2 | 188 |  |
| 5198MT | 51Mato GrossoConquista D'Oeste | 510336 | 5103361 | 3702.4 | 560.4 | 501.3 | 452.4 | 410 | 115 |  |
| 5199MT | 51Mato GrossoCotriguaçu | 510337 | 5103379 | 17386.6 | 2631.6 | 2354.3 | 2124.3 | 1925.6 | 114 |  |
| 5200MT | 51Mato GrossoCuiabá | 510340 | 5103403 | 578970.9 | 87633 | 78398.4 | 70737.4 | 64122.1 | 102072 | 39 |
| 5201MT | 51Mato GrossoCurvelândia | 510343 | 5103437 | 5011.5 | 758.5 | 678.6 | 612.3 | 555 | 49 |  |
| 5202MT | 51Mato GrossoDenise | 510345 | 5103452 | 8949.3 | 1354.6 | 1211.8 | 1093.4 | 991.2 | 922 |  |
| 5203MT | 51Mato GrossoDiamantino | 510350 | 5103502 | 21067.1 | 3188.7 | 2852.7 | 2573.9 | 2333.2 | 790 |  |
| 5204MT | 51Mato GrossoDom Aquino | 510360 | 5103601 | 8101.9 | 1226.3 | 1097.1 | 989.9 | 897.3 | 259 |  |
| 5205MT | 51Mato GrossoFeliz Natal | 510370 | 5103700 | 12568.5 | 1902.4 | 1701.9 | 1535.6 | 1392 | 662 |  |
| 5206MT | Figueirópolis  51Mato GrossoD'Oeste | 510380 | 5103809 | 3603.6 | 545.4 | 488 | 440.3 | 399.1 | 56 |  |
| 5207MT | 51Mato GrossoGaúcha do Norte | 510385 | 5103858 | 6958.5 | 1053.2 | 942.3 | 850.2 | 770.7 | 433 |  |
| 5208MT | 51Mato GrossoGeneral Carneiro | 510390 | 5103908 | 5268.2 | 797.4 | 713.4 | 643.7 | 583.5 | 163 |  |
| 5209MT | 51Mato GrossoGlória D'Oeste | 510395 | 5103957 | 3051.7 | 461.9 | 413.2 | 372.8 | 338 | 69 |  |
| 5210MT | 51Mato GrossoGuarantã do Norte | 510410 | 5104104 | 33848.1 | 5123.2 | 4583.4 | 4135.5 | 3748.7 | 729 |  |
| 5211MT | 51Mato GrossoGuiratinga | 510420 | 5104203 | 14462.5 | 2189 | 1958.4 | 1767 | 1601.7 | 386 |  |
| 5212MT | 51Mato GrossoIndiavaí | 510450 | 5104500 | 2557 | 387 | 346.2 | 312.4 | 283.2 | 18 |  |
| 5213MT | 51Mato GrossoIpiranga do Norte | 510452 | 5104526 | 6430.4 | 973.3 | 870.7 | 785.7 | 712.2 | 671 |  |
| 5214MT | 51Mato GrossoItanhangá | 510454 | 5104542 | 6007.2 | 909.2 | 813.4 | 733.9 | 665.3 | 337 |  |
| 5215MT | 51Mato GrossoItaúba | 510455 | 5104559 | 4123.3 | 624.1 | 558.3 | 503.8 | 456.7 | 186 |  |
| 5216MT | 51Mato GrossoItiquira | 510460 | 5104609 | 12374.4 | 1873 | 1675.6 | 1511.9 | 1370.5 | 622 |  |
| 5217MT | 51Mato GrossoJaciara | 510480 | 5104807 | 26475.8 | 4007.4 | 3585.1 | 3234.8 | 2932.2 | 1118 |  |
| 5218MT | 51Mato GrossoJangada | 510490 | 5104906 | 7961.3 | 1205 | 1078 | 972.7 | 881.7 | 525 |  |
| 5219MT | 51Mato GrossoJauru | 510500 | 5105002 | 9480.1 | 1434.9 | 1283.7 | 1158.3 | 1049.9 | 86 |  |
| 5220MT | 51Mato GrossoJuara | 510510 | 5105101 | 33665.6 | 5095.6 | 4558.7 | 4113.2 | 3728.5 | 486 |  |
| 5221MT | 51Mato GrossoJuína | 510515 | 5105150 | 39838.2 | 6029.9 | 5394.5 | 4867.3 | 4412.2 | 701 |  |
| 5222MT | 51Mato GrossoJuruena | 510517 | 5105176 | 13583.1 | 2055.9 | 1839.3 | 1659.6 | 1504.4 | 102 |  |
| 5223MT | 51Mato GrossoJuscimeira | 510520 | 5105200 | 11219 | 1698.1 | 1519.2 | 1370.7 | 1242.5 | 472 |  |
| 5224MT | 51Mato GrossoLambari D'Oeste | 510523 | 5105234 | 5749.2 | 870.2 | 778.5 | 702.4 | 636.7 | 44 |  |
| 5225MT | Lucas do Rio  51Mato GrossoVerde | 510525 | 5105259 | 55776.3 | 8442.3 | 7552.7 | 6814.6 | 6177.3 | 2297 |  |
| 5226MT | Vila Bela da  Santíssima  51Mato GrossoTrindade | 510550 | 5105507 | 15235.8 | 2306.1 | 2063.1 | 1861.5 | 1687.4 | 157 |  |
| 5227MT | 51Mato GrossoMarcelândia | 510558 | 5105580 | 11099.8 | 1680.1 | 1503 | 1356.1 | 1229.3 | 257 |  |
| 5228MT | 51Mato GrossoMatupá | 510560 | 5105606 | 15321.5 | 2319.1 | 2074.7 | 1871.9 | 1696.9 | 346 |  |
| 5229MT | 51Mato GrossoMirassol d'Oeste | 510562 | 5105622 | 26367.5 | 3991 | 3570.4 | 3221.5 | 2920.2 | 241 |  |
| 5230MT | 51Mato GrossoNobres | 510590 | 5105903 | 15047.9 | 2277.7 | 2037.6 | 1838.5 | 1666.6 | 1691 |  |
| 5231MT | 51Mato GrossoNortelândia | 510600 | 5106000 | 6142 | 929.7 | 831.7 | 750.4 | 680.2 | 294 |  |
| 5232MT | Nossa Senhora do  51Mato GrossoLivramento | 510610 | 5106109 | 12052.1 | 1824.2 | 1632 | 1472.5 | 1334.8 | 1089 |  |
| 5233MT | Nova 51Mato GrossoBandeirantes | 510615 | 5106158 | 13479.2 | 2040.2 | 1825.2 | 1646.9 | 1492.8 | 371 |  |
| 5234MT | 51Mato GrossoNova Nazaré | 510617 | 5106174 | 3438.3 | 520.4 | 465.6 | 420.1 | 380.8 | 271 |  |
| 5235MT | 51Mato GrossoNova Lacerda | 510618 | 5106182 | 6017.8 | 910.9 | 814.9 | 735.2 | 666.5 | 223 |  |
| 5236MT | Nova Santa  51Mato GrossoHelena | 510619 | 5106190 | 3570.4 | 540.4 | 483.5 | 436.2 | 395.4 | 292 |  |
| 5237MT | 51Mato GrossoNova Brasilândia | 510620 | 5106208 | 4142.3 | 627 | 560.9 | 506.1 | 458.8 | 633 |  |
| 5238MT | Nova Canaã do  51Mato GrossoNorte | 510621 | 5106216 | 12378 | 1873.5 | 1676.1 | 1512.3 | 1370.9 | 436 |  |
| 5239MT | 51Mato GrossoNova Mutum | 510622 | 5106224 | 38671.6 | 5853.3 | 5236.5 | 4724.8 | 4283 | 901 |  |
| 5240MT | 51Mato GrossoNova Olímpia | 510623 | 5106232 | 18842.8 | 2852 | 2551.5 | 2302.2 | 2086.9 | 1741 |  |
| 5241MT | 51Mato GrossoNova Ubiratã | 510624 | 5106240 | 10608.5 | 1605.7 | 1436.5 | 1296.1 | 1174.9 | 755 |  |
| 5242MT | 51Mato GrossoNova Xavantina | 510625 | 5106257 | 20392 | 3086.5 | 2761.3 | 2491.4 | 2258.5 | 513 |  |
| 5243MT | 51Mato GrossoNovo Mundo | 510626 | 5106265 | 8249.2 | 1248.6 | 1117 | 1007.9 | 913.6 | 168 |  |
| 5244MT | Novo Horizonte do  51Mato GrossoNorte | 510627 | 5106273 | 3851.8 | 583 | 521.6 | 470.6 | 426.6 | 232 |  |
| 5245MT | Novo São  51Mato GrossoJoaquim | 510628 | 5106281 | 5471.4 | 828.2 | 740.9 | 668.5 | 606 | 188 |  |
| 5246MT | 51Mato GrossoParanaíta | 510629 | 5106299 | 10878.4 | 1646.6 | 1473 | 1329.1 | 1204.8 | 459 |  |
| 5247MT | 51Mato GrossoParanatinga | 510630 | 5106307 | 20860.8 | 3157.5 | 2824.8 | 2548.7 | 2310.4 | 383 |  |
| 5248MT | Novo Santo  51Mato GrossoAntônio | 510631 | 5106315 | 2325 | 351.9 | 314.8 | 284.1 | 257.5 | 148 |  |
| 5249MT | 51Mato GrossoPedra Preta | 510637 | 5106372 | 16614.7 | 2514.8 | 2249.8 | 2029.9 | 1840.1 | 398 |  |
| 5250MT | Peixoto de  51Mato GrossoAzevedo | 510642 | 5106422 | 32738.8 | 4955.3 | 4433.2 | 4000 | 3625.9 | 450 |  |
| 5251MT | 51Mato GrossoPlanalto da Serra | 510645 | 5106455 | 2670 | 404.1 | 361.5 | 326.2 | 295.7 | 616 |  |
| 5252MT | 51Mato GrossoPoconé | 510650 | 5106505 | 32189.9 | 4872.3 | 4358.8 | 3932.9 | 3565.1 | 1933 |  |
| 5253MT | Pontal do  51Mato GrossoAraguaia | 510665 | 5106653 | 6047.4 | 915.3 | 818.9 | 738.9 | 669.8 | 282 |  |
| 5254MT | 51Mato GrossoPonte Branca | 510670 | 5106703 | 1650.3 | 249.8 | 223.5 | 201.6 | 182.8 | 98 |  |
| 5255MT | 51Mato GrossoPontes e Lacerda | 510675 | 5106752 | 43187.5 | 6536.9 | 5848 | 5276.6 | 4783.1 | 677 |  |
| 5256MT | Porto Alegre do  51Mato GrossoNorte | 510677 | 5106778 | 11594.2 | 1754.9 | 1570 | 1416.6 | 1284.1 | 42 |  |
| 5257MT | Porto dos  51Mato GrossoGaúchos | 510680 | 5106802 | 5381.2 | 814.5 | 728.7 | 657.5 | 596 | 137 |  |
| 5258MT | 51Mato GrossoPorto Esperidião | 510682 | 5106828 | 11459.2 | 1734.5 | 1551.7 | 1400.1 | 1269.1 | 74 |  |
| 5259MT | 51Mato GrossoPorto Estrela | 510685 | 5106851 | 3252.4 | 492.3 | 440.4 | 397.4 | 360.2 | 526 |  |
| 5260MT | 51Mato GrossoPoxoréo | 510700 | 5107008 | 16711.4 | 2529.4 | 2262.9 | 2041.8 | 1850.8 | 600 |  |
| 5261MT | Primavera do  51Mato GrossoLeste | 510704 | 5107040 | 56902.3 | 8612.7 | 7705.1 | 6952.2 | 6302 | 13804 |  |
| 5262MT | 51Mato GrossoQuerência | 510706 | 5107065 | 15282.6 | 2313.2 | 2069.4 | 1867.2 | 1692.6 | 277 |  |
| 5263MT | São José dos 51Mato GrossoQuatro Marcos | 510710 | 5107107 | 18783.1 | 2843 | 2543.4 | 2294.9 | 2080.3 | 228 |  |
| 5264MT | Reserva do  51Mato GrossoCabaçal | 510715 | 5107156 | 2635 | 398.8 | 356.8 | 321.9 | 291.8 | 20 |  |
| 5265MT | Ribeirão 51Mato GrossoCascalheira | 510718 | 5107180 | 9508.2 | 1439.2 | 1287.5 | 1161.7 | 1053.1 | 516 |  |
| 5266MT | 51Mato GrossoRibeirãozinho | 510719 | 5107198 | 2289.1 | 346.5 | 310 | 279.7 | 253.5 | 153 |  |
| 5267MT | 51Mato GrossoRio Branco | 510720 | 5107206 | 5072.8 | 767.8 | 686.9 | 619.8 | 561.8 | 54 |  |
| 5268MT | 51Mato GrossoSanta Carmem | 510724 | 5107248 | 4283 | 648.3 | 580 | 523.3 | 474.4 | 512 |  |
| 5269MT | 51Mato GrossoSanto Afonso | 510726 | 5107263 | 3047.3 | 461.2 | 412.6 | 372.3 | 337.5 | 353 |  |
| 5270MT | 51Mato GrossoSão José do Povo | 510729 | 5107297 | 3810.5 | 576.8 | 516 | 465.6 | 422 | 285 |  |
| 5271MT | São José do Rio  51Mato GrossoClaro | 510730 | 5107305 | 18856.1 | 2854.1 | 2553.3 | 2303.8 | 2088.3 | 808 |  |

1

5

| 5272MT | 51Mato GrossoSão José do Xingu | | 510735 | 5107354 | 5383.5 | 814.8 | 729 | 657.7 | 596.2 | 42 |  |
| --- | --- | --- | --- | --- | --- | --- | --- | --- | --- | --- | --- |
| 5273MT | São Pedro da  51Mato GrossoCipa | | 510740 | 5107404 | 4423.9 | 669.6 | 599 | 540.5 | 490 | 178 |  |
| 5274MT | 51Mato GrossoRondolândia | | 510757 | 5107578 | 3783.4 | 572.7 | 512.3 | 462.2 | 419 | 15 |  |
| 5275MT | 51Mato GrossoRondonópolis | | 510760 | 5107602 | 213435.4 | 32305.6 | 28901.3 | 26077.1 | 23638.4 | 15942 |  |
| 5276MT | 51Mato GrossoRosário Oeste | | 510770 | 5107701 | 17294.7 | 2617.7 | 2341.9 | 2113 | 1915.4 | 1353 |  |
| 5277MT | Santa Cruz do  51Mato GrossoXingu | | 510774 | 5107743 | 2236.6 | 338.5 | 302.9 | 273.3 | 247.7 | 7 |  |
| 5278MT | 51Mato GrossoSalto do Céu | | 510775 | 5107750 | 3584.9 | 542.6 | 485.4 | 438 | 397 | 32 |  |
| 5279MT | Santa Rita do  51Mato GrossoTrivelato | | 510776 | 5107768 | 2967.9 | 449.2 | 401.9 | 362.6 | 328.7 | 206 |  |
| 5280MT | 51Mato GrossoSanta Terezinha | | 510777 | 5107776 | 7850.7 | 1188.3 | 1063.1 | 959.2 | 869.5 | 32 |  |
| 5281MT | Santo Antônio do  51Mato GrossoLeste | | 510779 | 5107792 | 4479.1 | 678 | 606.5 | 547.2 | 496.1 | 92 |  |
| 5282MT | Santo Antônio do  51Mato GrossoLeverger | | 510780 | 5107800 | 18330.7 | 2774.5 | 2482.2 | 2239.6 | 2030.2 | 1116 |  |
| 5283MT | São Félix do  51Mato GrossoAraguaia | | 510785 | 5107859 | 11108.2 | 1681.3 | 1504.2 | 1357.2 | 1230.3 | 210 |  |
| 5284MT | 51Mato GrossoSapezal | | 510787 | 5107875 | 22075.8 | 3341.4 | 2989.3 | 2697.2 | 2444.9 | 64 |  |
| 5285MT | Serra Nova  51Mato GrossoDourada | | 510788 | 5107883 | 1504.6 | 227.7 | 203.7 | 183.8 | 166.6 | 34 |  |
| 5286MT | 51Mato GrossoSinop | | 510790 | 5107909 | 128005.1 | 19374.9 | 17333.2 | 15639.4 | 14176.8 | 8476 |  |
| 5287MT | 51Mato GrossoSorriso | | 510792 | 5107925 | 78588.5 | 11895.2 | 10641.7 | 9601.8 | 8703.8 | 12175 |  |
| 5288MT | 51Mato GrossoTabaporã | | 510794 | 5107941 | 9609.9 | 1454.6 | 1301.3 | 1174.1 | 1064.3 | 256 |  |
| 5289MT | 51Mato GrossoTangará da Serra | | 510795 | 5107958 | 93400.8 | 14137.1 | 12647.4 | 11411.5 | 10344.3 | 8081 |  |
| 5290MT | 51Mato GrossoTapurah | | 510800 | 5108006 | 12066 | 1826.3 | 1633.9 | 1474.2 | 1336.3 | 1006 |  |
| 5291MT | Terra Nova do  51Mato GrossoNorte | | 510805 | 5108055 | 10345.7 | 1565.9 | 1400.9 | 1264 | 1145.8 | 318 |  |
| 5292MT | 51Mato GrossoTesouro | | 510810 | 5108105 | 3574 | 541 | 484 | 436.7 | 395.8 | 105 |  |
| 5293MT | 51Mato GrossoTorixoréu | | 510820 | 5108204 | 3789.9 | 573.6 | 513.2 | 463 | 419.7 | 399 |  |
| 5294MT | 51Mato GrossoUnião do Sul | | 510830 | 5108303 | 3603 | 545.4 | 487.9 | 440.2 | 399 | 316 |  |
| 5295MT | Vale de São  51Mato GrossoDomingos | | 510835 | 5108352 | 3063.4 | 463.7 | 414.8 | 374.3 | 339.3 | 119 |  |
| 5296MT | 51Mato GrossoVárzea Grande | | 510840 | 5108402 | 267583.3 | 40501.4 | 36233.5 | 32692.7 | 29635.3 | 28939 | 12 |
| 5297MT | 51Mato GrossoVera | | 510850 | 5108501 | 10716.8 | 1622.1 | 1451.2 | 1309.4 | 1186.9 | 886 |  |
| 5298MT | 51Mato GrossoVila Rica | | 510860 | 5108600 | 23669.7 | 3582.6 | 3205.1 | 2891.9 | 2621.5 | 78 |  |
| 5299MT | 51Mato GrossoNova Guarita | | 510880 | 5108808 | 4669.1 | 706.7 | 632.2 | 570.5 | 517.1 | 280 |  |
| 5300MT | 51Mato GrossoNova Marilândia | | 510885 | 5108857 | 3099.5 | 469.1 | 419.7 | 378.7 | 343.3 | 403 |  |
| 5301MT | 51Mato GrossoNova Maringá | | 510890 | 5108907 | 7623.1 | 1153.8 | 1032.2 | 931.4 | 844.3 | 113 |  |
| 5302MT | 51Mato GrossoNova Monte Verde | | 510895 | 5108956 | 8600.3 | 1301.7 | 1164.6 | 1050.8 | 952.5 | 316 |  |
| 5303GO | 52Goiás | Abadia de Goiás | 520005 | 5200050 | 7787.3 | 1296.8 | 1092.6 | 1087.5 | 908.4 | 1123 |  |
| 5304GO | 52Goiás | Abadiânia | 520010 | 5200100 | 17820.6 | 2967.7 | 2500.4 | 2488.7 | 2078.8 | 905 |  |
| 5305GO | 52Goiás | Acreúna | 520013 | 5200134 | 21446.2 | 3571.4 | 3009.1 | 2995 | 2501.8 | 1272 |  |
| 5306GO | 52Goiás | Adelândia | 520015 | 5200159 | 2525.4 | 420.6 | 354.3 | 352.7 | 294.6 | 552 |  |
| 5307GO | 52Goiás | Água Fria de  Goiás | 520017 | 5200175 | 5434.9 | 905.1 | 762.6 | 759 | 634 | 58 |  |
| 5308GO | 52Goiás | Água Limpa | 520020 | 5200209 | 1968.7 | 327.8 | 276.2 | 274.9 | 229.7 | 47 |  |
| 5309GO | 52Goiás | Águas Lindas de  Goiás | 520025 | 5200258 | 184465.7 | 30719.1 | 25882.4 | 25761 | 21518.6 | 2008 |  |
| 5310GO | 52Goiás | Alexânia | 520030 | 5200308 | 25787.6 | 4294.4 | 3618.3 | 3601.3 | 3008.2 | 1546 |  |
| 5311GO | 52Goiás | Aloândia | 520050 | 5200506 | 2053.4 | 342 | 288.1 | 286.8 | 239.5 | 131 |  |
| 5312GO | 52Goiás | Alto Horizonte | 520055 | 5200555 | 5392 | 897.9 | 756.6 | 753 | 629 | 27 |  |
| 5313GO | 52Goiás | Alto Paraíso de  Goiás | 520060 | 5200605 | 7294.7 | 1214.8 | 1023.5 | 1018.7 | 851 | 26 |  |
| 5314GO | 52Goiás | Alvorada do Norte | 520080 | 5200803 | 8438.5 | 1405.3 | 1184 | 1178.5 | 984.4 | 1111 |  |
| 5315GO | 52Goiás | Amaralina | 520082 | 5200829 | 3642.6 | 606.6 | 511.1 | 508.7 | 424.9 | 132 |  |
| 5316GO | 52Goiás | Americano do  Brasil | 520085 | 5200852 | 5840.7 | 972.7 | 819.5 | 815.7 | 681.3 | 501 |  |
| 5317GO | 52Goiás | Amorinópolis | 520090 | 5200902 | 3445.7 | 573.8 | 483.5 | 481.2 | 402 | 115 |  |
| 5318GO | 52Goiás | Anápolis | 520110 | 5201108 | 361629.8 | 60222.2 | 50740.3 | 50502.3 | 42185.5 | 73743 | 28 |
| 5319GO | 52Goiás | Anhanguera | 520120 | 5201207 | 1089.5 | 181.4 | 152.9 | 152.2 | 127.1 | 82 |  |
| 5320GO | 52Goiás | Anicuns | 520130 | 5201306 | 21199.4 | 3530.3 | 2974.5 | 2960.5 | 2473 | 2410 |  |
| 5321GO | 52Goiás | Aparecida de  Goiânia | 520140 | 5201405 | 514718.1 | 85716 | 72220.1 | 71881.4 | 60043.8 | 80381 | 33 |
| 5322GO | 52Goiás | Aparecida do Rio  Doce | 520145 | 5201454 | 2478.2 | 412.7 | 347.7 | 346.1 | 289.1 | 129 |  |
| 5323GO | 52Goiás | Aporé | 520150 | 5201504 | 4023.7 | 670.1 | 564.6 | 561.9 | 469.4 | 149 |  |
| 5324GO | 52Goiás | Araçu | 520160 | 5201603 | 3729.7 | 621.1 | 523.3 | 520.9 | 435.1 | 338 |  |
| 5325GO | 52Goiás | Aragarças | 520170 | 5201702 | 19327.8 | 3218.7 | 2711.9 | 2699.2 | 2254.7 | 229 |  |
| 5326GO | 52Goiás | Aragoiânia | 520180 | 5201801 | 9316.4 | 1551.5 | 1307.2 | 1301.1 | 1086.8 | 838 |  |
| 5327GO | 52Goiás | Araguapaz | 520215 | 5202155 | 7719 | 1285.4 | 1083.1 | 1078 | 900.5 | 394 |  |
| 5328GO | 52Goiás | Arenópolis | 520235 | 5202353 | 3030.4 | 504.7 | 425.2 | 423.2 | 353.5 | 78 |  |
| 5329GO | 52Goiás | Aruanã | 520250 | 5202502 | 8625.1 | 1436.3 | 1210.2 | 1204.5 | 1006.2 | 642 |  |
| 5330GO | 52Goiás | Aurilândia | 520260 | 5202601 | 3466.8 | 577.3 | 486.4 | 484.1 | 404.4 | 540 |  |
| 5331GO | 52Goiás | Avelinópolis | 520280 | 5202809 | 2467.4 | 410.9 | 346.2 | 344.6 | 287.8 | 311 |  |
| 5332GO | 52Goiás | Baliza | 520310 | 5203104 | 4380.2 | 729.4 | 614.6 | 611.7 | 511 | 46 |  |
| 5333GO | 52Goiás | Barro Alto | 520320 | 5203203 | 9892.6 | 1647.4 | 1388 | 1381.5 | 1154 | 356 |  |
| 5334GO | 52Goiás | Bela Vista de  Goiás | 520330 | 5203302 | 27227.5 | 4534.2 | 3820.3 | 3802.4 | 3176.2 | 3679 |  |
| 5335GO | 52Goiás | Bom Jardim de  Goiás | 520340 | 5203401 | 8713 | 1451 | 1222.5 | 1216.8 | 1016.4 | 123 |  |
| 5336GO | 52Goiás | Bom Jesus de  Goiás | 520350 | 5203500 | 22942.9 | 3820.7 | 3219.1 | 3204 | 2676.4 | 408 |  |
| 5337GO | 52Goiás | Bonfinópolis | 520355 | 5203559 | 8574.9 | 1428 | 1203.1 | 1197.5 | 1000.3 | 717 |  |
| 5338GO | 52Goiás | Bonópolis | 520357 | 5203575 | 3939.6 | 656.1 | 552.8 | 550.2 | 459.6 | 9 |  |
| 5339GO | 52Goiás | Brazabrantes | 520360 | 5203609 | 3478.7 | 579.3 | 488.1 | 485.8 | 405.8 | 457 |  |
| 5340GO | 52Goiás | Britânia | 520380 | 5203807 | 5697.6 | 948.8 | 799.4 | 795.7 | 664.6 | 156 |  |
| 5341GO | 52Goiás | Buriti Alegre | 520390 | 5203906 | 9346 | 1556.4 | 1311.3 | 1305.2 | 1090.2 | 196 |  |
| 5342GO | 52Goiás | Buriti de Goiás | 520393 | 5203939 | 2562 | 426.6 | 359.5 | 357.8 | 298.9 | 275 |  |
| 5343GO | 52Goiás | Buritinópolis | 520396 | 5203962 | 3351.1 | 558.1 | 470.2 | 468 | 390.9 | 725 |  |
| 5344GO | 52Goiás | Cabeceiras | 520400 | 5204003 | 7726.6 | 1286.7 | 1084.1 | 1079 | 901.3 | 237 |  |
| 5345GO | 52Goiás | Cachoeira Alta | 520410 | 5204102 | 11526.8 | 1919.6 | 1617.3 | 1609.7 | 1344.6 | 761 |  |
| 5346GO | 52Goiás | Cachoeira de  Goiás | 520420 | 5204201 | 1406.9 | 234.3 | 197.4 | 196.5 | 164.1 | 252 |  |
| 5347GO | 52Goiás | Cachoeira  Dourada | 520425 | 5204250 | 8279.1 | 1378.7 | 1161.6 | 1156.2 | 965.8 | 277 |  |
| 5348GO | 52Goiás | Caçu | 520430 | 5204300 | 14637.4 | 2437.6 | 2053.8 | 2044.1 | 1707.5 | 1291 |  |
| 5349GO | 52Goiás | Caiapônia | 520440 | 5204409 | 17909.1 | 2982.4 | 2512.8 | 2501 | 2089.2 | 606 |  |
| 5350GO | 52Goiás | Caldas Novas | 520450 | 5204508 | 80361 | 13382.5 | 11275.5 | 11222.6 | 9374.4 | 1550 |  |
| 5351GO | 52Goiás | Caldazinha | 520455 | 5204557 | 3575.8 | 595.5 | 501.7 | 499.4 | 417.1 | 263 |  |
| 5352GO | 52Goiás | Campestre de  Goiás | 520460 | 5204607 | 3535.6 | 588.8 | 496.1 | 493.8 | 412.4 | 585 |  |
| 5353GO | 52Goiás | Campinaçu | 520465 | 5204656 | 3695.9 | 615.5 | 518.6 | 516.1 | 431.1 | 55 |  |
| 5354GO | 52Goiás | Campinorte | 520470 | 5204706 | 11911.7 | 1983.7 | 1671.3 | 1663.5 | 1389.5 | 687 |  |
| 5355GO | 52Goiás | Campo Alegre de  Goiás | 520480 | 5204805 | 6802.1 | 1132.8 | 954.4 | 949.9 | 793.5 | 283 |  |
| 5356GO | 52Goiás | Campo Limpo de  Goiás | 520485 | 5204854 | 6993.4 | 1164.6 | 981.2 | 976.6 | 815.8 | 343 |  |
| 5357GO | 52Goiás | Campos Belos | 520490 | 5204904 | 19287.7 | 3212 | 2706.3 | 2693.6 | 2250 | 90 |  |
| 5358GO | 52Goiás | Campos Verdes | 520495 | 5204953 | 3834.9 | 638.6 | 538.1 | 535.6 | 447.4 | 177 |  |
| 5359GO | 52Goiás | Carmo do Rio  Verde | 520500 | 5205000 | 9543.8 | 1589.3 | 1339.1 | 1332.8 | 1113.3 | 1008 |  |
| 5360GO | 52Goiás | Castelândia | 520505 | 5205059 | 3597.1 | 599 | 504.7 | 502.3 | 419.6 | 223 |  |
| 5361GO | 52Goiás | Catalão | 520510 | 5205109 | 97389.7 | 16218.3 | 13664.7 | 13600.7 | 11360.9 | 19050 |  |
| 5362GO | 52Goiás | Caturaí | 520520 | 5205208 | 4912.9 | 818.1 | 689.3 | 686.1 | 573.1 | 618 |  |
| 5363GO | 52Goiás | Cavalcante | 520530 | 5205307 | 9650.7 | 1607.1 | 1354.1 | 1347.7 | 1125.8 | 48 |  |

6

1

4

2

1

8

| 5364GO | 52Goiás | Ceres | 520540 | 5205406 | 21626.7 | 3601.5 | 3034.4 | 3020.2 | 2522.8 | 5494 |  |
| --- | --- | --- | --- | --- | --- | --- | --- | --- | --- | --- | --- |
| 5365GO | 52Goiás | Cezarina | 520545 | 5205455 | 8102.3 | 1349.3 | 1136.8 | 1131.5 | 945.2 | 522 |  |
| 5366GO | 52Goiás | Chapadão do Céu | 520547 | 5205471 | 8465.8 | 1409.8 | 1187.8 | 1182.3 | 987.6 | 350 |  |
| 5367GO | 52Goiás | Cidade Ocidental | 520549 | 5205497 | 63347.1 | 10549.2 | 8888.2 | 8846.6 | 7389.7 | 689 |  |
| 5368GO | 52Goiás | Cocalzinho de  Goiás | 520551 | 5205513 | 18860.9 | 3140.9 | 2646.4 | 2634 | 2200.2 | 594 |  |
| 5369GO | 52Goiás | Colinas do Sul | 520552 | 5205521 | 3507.2 | 584.1 | 492.1 | 489.8 | 409.1 | 41 |  |
| 5370GO | 52Goiás | Córrego do Ouro | 520570 | 5205703 | 2533.3 | 421.9 | 355.4 | 353.8 | 295.5 | 378 |  |
| 5371GO | 52Goiás | Corumbá de Goiás | 520580 | 5205802 | 10819.4 | 1801.8 | 1518.1 | 1511 | 1262.1 | 507 |  |
| 5372GO | 52Goiás | Corumbaíba | 520590 | 5205901 | 8955.7 | 1491.4 | 1256.6 | 1250.7 | 1044.7 | 372 |  |
| 5373GO | 52Goiás | Cristalina | 520620 | 5206206 | 52570.4 | 8754.5 | 7376.2 | 7341.6 | 6132.5 | 742 |  |
| 5374GO | 52Goiás | Cristianópolis | 520630 | 5206305 | 2984.7 | 497 | 418.8 | 416.8 | 348.2 | 220 |  |
| 5375GO | 52Goiás | Crixás | 520640 | 5206404 | 16479.1 | 2744.3 | 2312.2 | 2301.3 | 1922.3 | 464 |  |
| 5376GO | 52Goiás | Cromínia | 520650 | 5206503 | 3571 | 594.7 | 501 | 498.7 | 416.6 | 259 |  |
| 5377GO | 52Goiás | Cumari | 520660 | 5206602 | 2954.6 | 492 | 414.6 | 412.6 | 344.7 | 293 |  |
| 5378GO | 52Goiás | Damianópolis | 520670 | 5206701 | 3342.5 | 556.6 | 469 | 466.8 | 389.9 | 158 |  |
| 5379GO | 52Goiás | Damolândia | 520680 | 5206800 | 2865.4 | 477.2 | 402 | 400.2 | 334.3 | 595 |  |
| 5380GO | 52Goiás | Davinópolis | 520690 | 5206909 | 2099.3 | 349.6 | 294.6 | 293.2 | 244.9 | 204 |  |
| 5381GO | 52Goiás | Diorama | 520710 | 5207105 | 2513.3 | 418.5 | 352.6 | 351 | 293.2 | 195 |  |
| 5382GO | 52Goiás | Doverlândia | 520725 | 5207253 | 7746 | 1289.9 | 1086.8 | 1081.7 | 903.6 | 225 |  |
| 5383GO | 52Goiás | Edealina | 520735 | 5207352 | 3766.2 | 627.2 | 528.4 | 526 | 439.3 | 528 |  |
| 5384GO | 52Goiás | Edéia | 520740 | 5207402 | 11890.8 | 1980.2 | 1668.4 | 1660.6 | 1387.1 | 459 |  |
| 5385GO | 52Goiás | Estrela do Norte | 520750 | 5207501 | 3343.3 | 556.8 | 469.1 | 466.9 | 390 | 32 |  |
| 5386GO | 52Goiás | Faina | 520753 | 5207535 | 6918 | 1152.1 | 970.7 | 966.1 | 807 | 379 |  |
| 5387GO | 52Goiás | Fazenda Nova | 520760 | 5207600 | 6106.3 | 1016.9 | 856.8 | 852.8 | 712.3 | 128 |  |
| 5388GO | 52Goiás | Firminópolis | 520780 | 5207808 | 12472.4 | 2077 | 1750 | 1741.8 | 1455 | 837 |  |
| 5389GO | 52Goiás | Flores de Goiás | 520790 | 5207907 | 14170.6 | 2359.8 | 1988.3 | 1979 | 1653.1 | 223 |  |
| 5390GO | 52Goiás | Formosa | 520800 | 5208004 | 110721.2 | 18438.4 | 15535.3 | 15462.4 | 12916 | 4778 |  |
| 5391GO | 52Goiás | Formoso | 520810 | 5208103 | 4670 | 777.7 | 655.2 | 652.2 | 544.8 | 76 |  |
| 5392GO | 52Goiás | Gameleira de  Goiás | 520815 | 5208152 | 3586.3 | 597.2 | 503.2 | 500.8 | 418.4 | 358 |  |
| 5393GO | 52Goiás | Divinópolis de  Goiás | 520830 | 5208301 | 4957.3 | 825.5 | 695.6 | 692.3 | 578.3 | 22 |  |
| 5394GO | 52Goiás | Goianápolis | 520840 | 5208400 | 11053 | 1840.7 | 1550.8 | 1543.6 | 1289.4 | 1310 |  |
| 5395GO | 52Goiás | Goiandira | 520850 | 5208509 | 5478 | 912.3 | 768.6 | 765 | 639 | 382 |  |
| 5396GO | 52Goiás | Goianésia | 520860 | 5208608 | 64889.1 | 10806 | 9104.6 | 9061.9 | 7569.6 | 7700 |  |
| 5397GO | 52Goiás | Goiânia | 520870 | 5208707 | 1411711.4 | 235092.3 | 198077.2 | 197148.4 | 164681.4 | 283665 | 114 |
| 5398GO | 52Goiás | Goianira | 520880 | 5208806 | 38938.8 | 6484.5 | 5463.5 | 5437.9 | 4542.4 | 5475 |  |
| 5399GO | 52Goiás | Goiás | 520890 | 5208905 | 24141.6 | 4020.3 | 3387.3 | 3371.4 | 2816.2 | 3726 |  |
| 5400GO | 52Goiás | Goiatuba | 520910 | 5209101 | 33606.3 | 5596.5 | 4715.3 | 4693.2 | 3920.3 | 7472 |  |
| 5401GO | 52Goiás | Gouvelândia | 520915 | 5209150 | 5424.9 | 903.4 | 761.2 | 757.6 | 632.8 | 165 |  |
| 5402GO | 52Goiás | Guapó | 520920 | 5209200 | 14257.6 | 2374.3 | 2000.5 | 1991.1 | 1663.2 | 1347 |  |
| 5403GO | 52Goiás | Guaraíta | 520929 | 5209291 | 2241.4 | 373.3 | 314.5 | 313 | 261.5 | 470 |  |
| 5404GO | 52Goiás | Guarani de Goiás | 520940 | 5209408 | 4153.7 | 691.7 | 582.8 | 580.1 | 484.5 | 124 |  |
| 5405GO | 52Goiás | Guarinos | 520945 | 5209457 | 2109.6 | 351.3 | 296 | 294.6 | 246.1 | 98 |  |
| 5406GO | 52Goiás | Heitoraí | 520960 | 5209606 | 3683.5 | 613.4 | 516.8 | 514.4 | 429.7 | 355 |  |
| 5407GO | 52Goiás | Hidrolândia | 520970 | 5209705 | 19492.2 | 3246 | 2735 | 2722.1 | 2273.8 | 2523 |  |
| 5408GO | 52Goiás | Hidrolina | 520980 | 5209804 | 3879.5 | 646.1 | 544.3 | 541.8 | 452.6 | 94 |  |
| 5409GO | 52Goiás | Iaciara | 520990 | 5209903 | 13246.9 | 2206 | 1858.7 | 1850 | 1545.3 | 413 |  |
| 5410GO | 52Goiás | Inaciolândia | 520993 | 5209937 | 5987.1 | 997 | 840.1 | 836.1 | 698.4 | 243 |  |
| 5411GO | 52Goiás | Indiara | 520995 | 5209952 | 14698.3 | 2447.7 | 2062.3 | 2052.6 | 1714.6 | 488 |  |
| 5412GO | 52Goiás | Inhumas | 521000 | 5210000 | 50872.6 | 8471.8 | 7137.9 | 7104.5 | 5934.5 | 2383 |  |
| 5413GO | 52Goiás | Ipameri | 521010 | 5210109 | 26030.4 | 4334.8 | 3652.3 | 3635.2 | 3036.5 | 624 |  |
| 5414GO | 52Goiás | Ipiranga de Goiás | 521015 | 5210158 | 2902.1 | 483.3 | 407.2 | 405.3 | 338.5 | 394 |  |
| 5415GO | 52Goiás | Iporá | 521020 | 5210208 | 31787.7 | 5293.6 | 4460.1 | 4439.2 | 3708.2 | 1334 |  |
| 5416GO | 52Goiás | Israelândia | 521030 | 5210307 | 2886.7 | 480.7 | 405 | 403.1 | 336.7 | 101 |  |
| 5417GO | 52Goiás | Itaberaí | 521040 | 5210406 | 39095 | 6510.5 | 5485.4 | 5459.7 | 4560.6 | 996 |  |
| 5418GO | 52Goiás | Itaguari | 521056 | 5210562 | 4642.4 | 773.1 | 651.4 | 648.3 | 541.6 | 902 |  |
| 5419GO | 52Goiás | Itaguaru | 521060 | 5210604 | 5419.9 | 902.6 | 760.5 | 756.9 | 632.3 | 452 |  |
| 5420GO | 52Goiás | Itajá | 521080 | 5210802 | 4900.6 | 816.1 | 687.6 | 684.4 | 571.7 | 415 |  |
| 5421GO | 52Goiás | Itapaci | 521090 | 5210901 | 20659.6 | 3440.4 | 2898.7 | 2885.2 | 2410 | 1064 |  |
| 5422GO | 52Goiás | Itapirapuã | 521100 | 5211008 | 6704 | 1116.4 | 940.6 | 936.2 | 782 | 479 |  |
| 5423GO | 52Goiás | Itapuranga | 521120 | 5211206 | 26305.9 | 4380.7 | 3691 | 3673.7 | 3068.7 | 1325 |  |
| 5424GO | 52Goiás | Itarumã | 521130 | 5211305 | 6762.5 | 1126.2 | 948.8 | 944.4 | 788.9 | 407 |  |
| 5425GO | 52Goiás | Itauçu | 521140 | 5211404 | 8842.9 | 1472.6 | 1240.7 | 1234.9 | 1031.6 | 583 |  |
| 5426GO | 52Goiás | Itumbiara | 521150 | 5211503 | 99226.7 | 16524.2 | 13922.5 | 13857.2 | 11575.2 | 5472 |  |
| 5427GO | 52Goiás | Ivolândia | 521160 | 5211602 | 2570 | 428 | 360.6 | 358.9 | 299.8 | 106 |  |
| 5428GO | 52Goiás | Jandaia | 521170 | 5211701 | 6193.4 | 1031.4 | 869 | 864.9 | 722.5 | 157 |  |
| 5429GO | 52Goiás | Jaraguá | 521180 | 5211800 | 46160.2 | 7687.1 | 6476.7 | 6446.4 | 5384.8 | 1081 |  |
| 5430GO | 52Goiás | Jataí | 521190 | 5211909 | 94728.6 | 15775.2 | 13291.4 | 13229 | 11050.4 | 8501 |  |
| 5431GO | 52Goiás | Jaupaci | 521200 | 5212006 | 2986.2 | 497.3 | 419 | 417 | 348.4 | 115 |  |
| 5432GO | 52Goiás | Jesúpolis | 521205 | 5212055 | 2412.4 | 401.7 | 338.5 | 336.9 | 281.4 | 796 |  |
| 5433GO | 52Goiás | Joviânia | 521210 | 5212105 | 7326.9 | 1220.1 | 1028 | 1023.2 | 854.7 | 343 |  |
| 5434GO | 52Goiás | Jussara | 521220 | 5212204 | 19106 | 3181.7 | 2680.8 | 2668.2 | 2228.8 | 249 |  |
| 5435GO | 52Goiás | Lagoa Santa | 521225 | 5212253 | 1415.3 | 235.7 | 198.6 | 197.6 | 165.1 | 107 |  |
| 5436GO | 52Goiás | Leopoldo de  Bulhões | 521230 | 5212303 | 7894.4 | 1314.7 | 1107.7 | 1102.5 | 920.9 | 429 |  |
| 5437GO | 52Goiás | Luziânia | 521250 | 5212501 | 191437.1 | 31880 | 26860.5 | 26734.6 | 22331.9 | 495 |  |
| 5438GO | 52Goiás | Mairipotaba | 521260 | 5212600 | 2401.9 | 400 | 337 | 335.4 | 280.2 | 289 |  |
| 5439GO | 52Goiás | Mambaí | 521270 | 5212709 | 7832.8 | 1304.4 | 1099 | 1093.9 | 913.7 | 337 |  |
| 5440GO | 52Goiás | Mara Rosa | 521280 | 5212808 | 10288.6 | 1713.4 | 1443.6 | 1436.8 | 1200.2 | 559 |  |
| 5441GO | 52Goiás | Marzagão | 521290 | 5212907 | 2169.9 | 361.4 | 304.5 | 303 | 253.1 | 67 |  |
| 5442GO | 52Goiás | Matrinchã | 521295 | 5212956 | 4443.5 | 740 | 623.5 | 620.5 | 518.4 | 183 |  |
| 5443GO | 52Goiás | Maurilândia | 521300 | 5213004 | 12780.9 | 2128.4 | 1793.3 | 1784.9 | 1490.9 | 560 |  |
| 5444GO | 52Goiás | Mimoso de Goiás | 521305 | 5213053 | 2681.6 | 446.6 | 376.3 | 374.5 | 312.8 | 30 |  |
| 5445GO | 52Goiás | Minaçu | 521308 | 5213087 | 30650.6 | 5104.2 | 4300.6 | 4280.4 | 3575.5 | 316 |  |
| 5446GO | 52Goiás | Mineiros | 521310 | 5213103 | 59636.9 | 9931.3 | 8367.7 | 8328.4 | 6956.9 | 2972 |  |
| 5447GO | 52Goiás | Moiporá | 521340 | 5213400 | 1683.8 | 280.4 | 236.3 | 235.1 | 196.4 | 38 |  |
| 5448GO | 52Goiás | Monte Alegre de  Goiás | 521350 | 5213509 | 8209.8 | 1367.2 | 1151.9 | 1146.5 | 957.7 | 23 |  |
| 5449GO | 52Goiás | Montes Claros de  Goiás | 521370 | 5213707 | 8119.6 | 1352.2 | 1139.3 | 1133.9 | 947.2 | 103 |  |
| 5450GO | 52Goiás | Montividiu | 521375 | 5213756 | 11934.7 | 1987.5 | 1674.6 | 1666.7 | 1392.2 | 494 |  |
| 5451GO | 52Goiás | Montividiu do  Norte | 521377 | 5213772 | 4330.3 | 721.1 | 607.6 | 604.7 | 505.1 | 39 |  |
| 5452GO | 52Goiás | Morrinhos | 521380 | 5213806 | 44023.5 | 7331.2 | 6176.9 | 6148 | 5135.5 | 4840 |  |
| 5453GO | 52Goiás | Morro Agudo de  Goiás | 521385 | 5213855 | 2339.5 | 389.6 | 328.3 | 326.7 | 272.9 | 71 |  |
| 5454GO | 52Goiás | Mossâmedes | 521390 | 5213905 | 4760.1 | 792.7 | 667.9 | 664.8 | 555.3 | 298 |  |
| 5455GO | 52Goiás | Mozarlândia | 521400 | 5214002 | 14555.6 | 2423.9 | 2042.3 | 2032.7 | 1698 | 246 |  |
| 5456GO | 52Goiás | Mundo Novo | 521405 | 5214051 | 5847.4 | 973.8 | 820.4 | 816.6 | 682.1 | 139 |  |
| 5457GO | 52Goiás | Mutunópolis | 521410 | 5214101 | 3867.8 | 644.1 | 542.7 | 540.1 | 451.2 | 88 |  |
| 5458GO | 52Goiás | Nazário | 521440 | 5214408 | 8526 | 1419.8 | 1196.3 | 1190.7 | 994.6 | 743 |  |
| 5459GO | 52Goiás | Nerópolis | 521450 | 5214507 | 26970.1 | 4491.3 | 3784.2 | 3766.4 | 3146.2 | 2617 |  |
| 5460GO | 52Goiás | Niquelândia | 521460 | 5214606 | 44654.6 | 7436.3 | 6265.5 | 6236.1 | 5209.1 | 151 |  |
| 5461GO | 52Goiás | Nova América | 521470 | 5214705 | 2328.2 | 387.7 | 326.7 | 325.1 | 271.6 | 356 |  |
| 5462GO | 52Goiás | Nova Aurora | 521480 | 5214804 | 2153 | 358.5 | 302.1 | 300.7 | 251.2 | 75 |  |
| 5463GO | 52Goiás | Nova Crixás | 521483 | 5214838 | 12489.2 | 2079.8 | 1752.4 | 1744.1 | 1456.9 | 107 |  |
| 5464GO | 52Goiás | Nova Glória | 521486 | 5214861 | 8468.9 | 1410.3 | 1188.3 | 1182.7 | 987.9 | 709 |  |
| 5465GO | 52Goiás | Nova Iguaçu de  Goiás | 521487 | 5214879 | 2906.8 | 484.1 | 407.9 | 405.9 | 339.1 | 251 |  |
| 5466GO | 52Goiás | Nova Roma | 521490 | 5214903 | 3426.5 | 570.6 | 480.8 | 478.5 | 399.7 | 394 |  |
| 5467GO | 52Goiás | Nova Veneza | 521500 | 5215009 | 8982.1 | 1495.8 | 1260.3 | 1254.4 | 1047.8 | 843 |  |
| 5468GO | 52Goiás | Novo Brasil | 521520 | 5215207 | 3301.6 | 549.8 | 463.2 | 461.1 | 385.1 | 171 |  |

1

1

3

2

1

2

1

2

1

1

| 5469GO | 52Goiás | Novo Gama | 521523 | 5215231 | 105231.2 | 17524.2 | 14765 | 14695.8 | 12275.6 | 309 |  |
| --- | --- | --- | --- | --- | --- | --- | --- | --- | --- | --- | --- |
| 5470GO | 52Goiás | Novo Planalto | 521525 | 5215256 | 4241.2 | 706.3 | 595.1 | 592.3 | 494.8 | 93 |  |
| 5471GO | 52Goiás | Orizona | 521530 | 5215306 | 15055.3 | 2507.2 | 2112.4 | 2102.5 | 1756.3 | 1365 |  |
| 5472GO | 52Goiás | Ouro Verde de  Goiás | 521540 | 5215405 | 3966.6 | 660.6 | 556.6 | 553.9 | 462.7 | 1173 |  |
| 5473GO | 52Goiás | Ouvidor | 521550 | 5215504 | 6058.5 | 1008.9 | 850.1 | 846.1 | 706.7 | 378 |  |
| 5474GO | 52Goiás | Padre Bernardo | 521560 | 5215603 | 30706 | 5113.5 | 4308.4 | 4288.2 | 3582 | 1058 |  |
| 5475GO | 52Goiás | Palestina de Goiás | 521565 | 5215652 | 3454.4 | 575.3 | 484.7 | 482.4 | 403 | 307 |  |
| 5476GO | 52Goiás | Palmeiras de  Goiás | 521570 | 5215702 | 26035 | 4335.6 | 3653 | 3635.8 | 3037.1 | 326 |  |
| 5477GO | 52Goiás | Palmelo | 521580 | 5215801 | 2385.1 | 397.2 | 334.7 | 333.1 | 278.2 | 148 |  |
| 5478GO | 52Goiás | Palminópolis | 521590 | 5215900 | 3615.2 | 602 | 507.2 | 504.9 | 421.7 | 326 |  |
| 5479GO | 52Goiás | Panamá | 521600 | 5216007 | 2687.9 | 447.6 | 377.1 | 375.4 | 313.6 | 129 |  |
| 5480GO | 52Goiás | Paranaiguara | 521630 | 5216304 | 9632.8 | 1604.2 | 1351.6 | 1345.2 | 1123.7 | 646 |  |
| 5481GO | 52Goiás | Paraúna | 521640 | 5216403 | 11057.1 | 1841.3 | 1551.4 | 1544.1 | 1289.9 | 832 |  |
| 5482GO | 52Goiás | Perolândia | 521645 | 5216452 | 3065.7 | 510.5 | 430.1 | 428.1 | 357.6 | 145 |  |
| 5483GO | 52Goiás | Petrolina de Goiás | 521680 | 5216809 | 10414.3 | 1734.3 | 1461.2 | 1454.4 | 1214.9 | 1010 |  |
| 5484GO | 52Goiás | Pilar de Goiás | 521690 | 5216908 | 2583.3 | 430.2 | 362.5 | 360.8 | 301.4 | 224 |  |
| 5485GO | 52Goiás | Piracanjuba | 521710 | 5217104 | 24506.1 | 4081 | 3438.5 | 3422.3 | 2858.7 | 2989 |  |
| 5486GO | 52Goiás | Piranhas | 521720 | 5217203 | 11028.2 | 1836.5 | 1547.4 | 1540.1 | 1286.5 | 531 |  |
| 5487GO | 52Goiás | Pirenópolis | 521730 | 5217302 | 24127.5 | 4018 | 3385.3 | 3369.5 | 2814.6 | 1272 |  |
| 5488GO | 52Goiás | Pires do Rio | 521740 | 5217401 | 30303.1 | 5046.4 | 4251.8 | 4231.9 | 3535 | 1568 |  |
| 5489GO | 52Goiás | Planaltina | 521760 | 5217609 | 86313.3 | 14373.8 | 12110.6 | 12053.8 | 10068.8 | 519 |  |
| 5490GO | 52Goiás | Pontalina | 521770 | 5217708 | 17644.5 | 2938.3 | 2475.7 | 2464.1 | 2058.3 | 1164 |  |
| 5491GO | 52Goiás | Porangatu | 521800 | 5218003 | 44219.7 | 7363.9 | 6204.5 | 6175.4 | 5158.4 | 858 |  |
| 5492GO | 52Goiás | Porteirão | 521805 | 5218052 | 3621.6 | 603.1 | 508.1 | 505.8 | 422.5 | 204 |  |
| 5493GO | 52Goiás | Portelândia | 521810 | 5218102 | 3963.4 | 660 | 556.1 | 553.5 | 462.3 | 55 |  |
| 5494GO | 52Goiás | Posse | 521830 | 5218300 | 34203 | 5695.8 | 4799 | 4776.5 | 3989.9 | 783 |  |
| 5495GO | 52Goiás | Professor Jamil | 521839 | 5218391 | 3315.6 | 552.1 | 465.2 | 463 | 386.8 | 282 |  |
| 5496GO | 52Goiás | Quirinópolis | 521850 | 5218508 | 46747.9 | 7784.9 | 6559.2 | 6528.4 | 5453.3 | 2650 |  |
| 5497GO | 52Goiás | Rialma | 521860 | 5218607 | 10829.7 | 1803.5 | 1519.5 | 1512.4 | 1263.3 | 1309 |  |
| 5498GO | 52Goiás | Rianápolis | 521870 | 5218706 | 4726.9 | 787.2 | 663.2 | 660.1 | 551.4 | 333 |  |
| 5499GO | 52Goiás | Rio Quente | 521878 | 5218789 | 3868.6 | 644.2 | 542.8 | 540.3 | 451.3 | 175 |  |
| 5500GO | 52Goiás | Rio Verde | 521880 | 5218805 | 204405.7 | 34039.7 | 28680.2 | 28545.7 | 23844.7 | 29099 |  |
| 5501GO | 52Goiás | Rubiataba | 521890 | 5218904 | 19578 | 3260.3 | 2747 | 2734.1 | 2283.8 | 1430 |  |
| 5502GO | 52Goiás | Sanclerlândia | 521900 | 5219001 | 7684.7 | 1279.7 | 1078.2 | 1073.2 | 896.4 | 862 |  |
| 5503GO | 52Goiás | Santa Bárbara de  Goiás | 521910 | 5219100 | 6176.3 | 1028.5 | 866.6 | 862.5 | 720.5 | 979 |  |
| 5504GO | 52Goiás | Santa Cruz de  Goiás | 521920 | 5219209 | 3057.6 | 509.2 | 429 | 427 | 356.7 | 262 |  |
| 5505GO | 52Goiás | Santa Fé de Goiás | 521925 | 5219258 | 5125.6 | 853.6 | 719.2 | 715.8 | 597.9 | 137 |  |
| 5506GO | 52Goiás | Santa Helena de  Goiás | 521930 | 5219308 | 37884.5 | 6308.9 | 5315.6 | 5290.6 | 4419.4 | 3407 |  |
| 5507GO | 52Goiás | Santa Isabel | 521935 | 5219357 | 3787.1 | 630.7 | 531.4 | 528.9 | 441.8 | 631 |  |
| 5508GO | 52Goiás | Santa Rita do  Araguaia | 521940 | 5219407 | 7808.1 | 1300.3 | 1095.6 | 1090.4 | 910.8 | 139 |  |
| 5509GO | 52Goiás | Santa Rita do  Novo Destino | 521945 | 5219456 | 3287.9 | 547.5 | 461.3 | 459.2 | 383.5 | 145 |  |
| 5510GO | 52Goiás | Santa Rosa de  Goiás | 521950 | 5219506 | 2690.5 | 448 | 377.5 | 375.7 | 313.9 | 421 |  |
| 5511GO | 52Goiás | Santa Tereza de  Goiás | 521960 | 5219605 | 3768.7 | 627.6 | 528.8 | 526.3 | 439.6 | 39 |  |
| 5512GO | 52Goiás | Santa Terezinha de Goiás | 521970 | 5219704 | 9758.7 | 1625.1 | 1369.2 | 1362.8 | 1138.4 | 314 |  |
| 5513GO | 52Goiás | Santo Antônio da  Barra | 521971 | 5219712 | 4652.6 | 774.8 | 652.8 | 649.7 | 542.7 | 363 |  |
| 5514GO | 52Goiás | Santo Antônio de  Goiás | 521973 | 5219738 | 5449.7 | 907.5 | 764.6 | 761.1 | 635.7 | 683 |  |
| 5515GO | 52Goiás | Santo Antônio do  Descoberto | 521975 | 5219753 | 69052.8 | 11499.4 | 9688.8 | 9643.4 | 8055.3 | 282 |  |
| 5516GO | 52Goiás | São Domingos | 521980 | 5219803 | 12144.9 | 2022.5 | 1704.1 | 1696.1 | 1416.7 | 122 |  |
| 5517GO | 52Goiás | São Francisco de  Goiás | 521990 | 5219902 | 6260.9 | 1042.6 | 878.5 | 874.3 | 730.4 | 301 |  |
| 5518GO | 52Goiás | São João d'Aliança | 522000 | 5220009 | 11901.1 | 1981.9 | 1669.8 | 1662 | 1388.3 | 20 |  |
| 5519GO | 52Goiás | São João da  Paraúna | 522005 | 5220058 | 1577.6 | 262.7 | 221.4 | 220.3 | 184 | 407 |  |
| 5520GO | 52Goiás | São Luís de  Montes Belos | 522010 | 5220108 | 32063.4 | 5339.5 | 4498.8 | 4477.7 | 3740.3 | 1590 |  |
| 5521GO | 52Goiás | São Luíz do Norte | 522015 | 5220157 | 4914.9 | 818.5 | 689.6 | 686.4 | 573.3 | 186 |  |
| 5522GO | 52Goiás | São Miguel do  Araguaia | 522020 | 5220207 | 22443.4 | 3737.5 | 3149 | 3134.3 | 2618.1 | 631 |  |
| 5523GO | 52Goiás | São Miguel do  Passa Quatro | 522026 | 5220264 | 3935.6 | 655.4 | 552.2 | 549.6 | 459.1 | 503 |  |
| 5524GO | 52Goiás | São Patrício | 522028 | 5220280 | 2036.1 | 339.1 | 285.7 | 284.3 | 237.5 | 288 |  |
| 5525GO | 52Goiás | São Simão | 522040 | 5220405 | 18852.2 | 3139.5 | 2645.2 | 2632.7 | 2199.2 | 2073 |  |
| 5526GO | 52Goiás | Senador Canedo | 522045 | 5220454 | 98957.2 | 16479.3 | 13884.7 | 13819.6 | 11543.7 | 21711 |  |
| 5527GO | 52Goiás | Serranópolis | 522050 | 5220504 | 8039.1 | 1338.8 | 1128 | 1122.7 | 937.8 | 399 |  |
| 5528GO | 52Goiás | Silvânia | 522060 | 5220603 | 19999.2 | 3330.5 | 2806.1 | 2792.9 | 2333 | 1142 |  |
| 5529GO | 52Goiás | Simolândia | 522068 | 5220686 | 6746.3 | 1123.5 | 946.6 | 942.1 | 787 | 364 |  |
| 5530GO | 52Goiás | Sítio d'Abadia | 522070 | 5220702 | 2932.3 | 488.3 | 411.4 | 409.5 | 342.1 | 59 |  |
| 5531GO | 52Goiás | Taquaral de Goiás | 522100 | 5221007 | 3581 | 596.3 | 502.5 | 500.1 | 417.7 | 399 |  |
| 5532GO | 52Goiás | Teresina de Goiás | 522108 | 5221080 | 3246.6 | 540.7 | 455.5 | 453.4 | 378.7 | 33 |  |
| 5533GO | 52Goiás | Terezópolis de  Goiás | 522119 | 5221197 | 7289 | 1213.8 | 1022.7 | 1017.9 | 850.3 | 703 |  |
| 5534GO | 52Goiás | Três Ranchos | 522130 | 5221304 | 2861.3 | 476.5 | 401.5 | 399.6 | 333.8 | 417 |  |
| 5535GO | 52Goiás | Trindade | 522140 | 5221403 | 115865.9 | 19295.1 | 16257.1 | 16180.9 | 13516.2 | 9336 |  |
| 5536GO | 52Goiás | Trombas | 522145 | 5221452 | 3517.2 | 585.7 | 493.5 | 491.2 | 410.3 | 29 |  |
| 5537GO | 52Goiás | Turvânia | 522150 | 5221502 | 4796.7 | 798.8 | 673 | 669.9 | 559.6 | 429 |  |
| 5538GO | 52Goiás | Turvelândia | 522155 | 5221551 | 4838.5 | 805.8 | 678.9 | 675.7 | 564.4 | 257 |  |
| 5539GO | 52Goiás | Uirapuru | 522157 | 5221577 | 2936.4 | 489 | 412 | 410.1 | 342.5 | 138 |  |
| 5540GO | 52Goiás | Uruaçu | 522160 | 5221601 | 38968.9 | 6489.5 | 5467.7 | 5442.1 | 4545.9 | 3070 |  |
| 5541GO | 52Goiás | Uruana | 522170 | 5221700 | 14011.3 | 2333.3 | 1965.9 | 1956.7 | 1634.5 | 1307 |  |
| 5542GO | 52Goiás | Urutaí | 522180 | 5221809 | 3113.3 | 518.5 | 436.8 | 434.8 | 363.2 | 251 |  |
| 5543GO | 52Goiás | Valparaíso de  Goiás | 522185 | 5221858 | 150669.4 | 25091 | 21140.4 | 21041.3 | 17576.1 | 8356 |  |
| 5544GO | 52Goiás | Varjão | 522190 | 5221908 | 3778.9 | 629.3 | 530.2 | 527.7 | 440.8 | 302 |  |
| 5545GO | 52Goiás | Vianópolis | 522200 | 5222005 | 13280.6 | 2211.6 | 1863.4 | 1854.7 | 1549.2 | 598 |  |
| 5546GO | 52Goiás | Vicentinópolis | 522205 | 5222054 | 8061.6 | 1342.5 | 1131.1 | 1125.8 | 940.4 | 241 |  |
| 5547GO | 52Goiás | Vila Boa | 522220 | 5222203 | 5419 | 902.4 | 760.3 | 756.8 | 632.1 | 229 |  |
| 5548GO | 52Goiás | Vila Propício | 522230 | 5222302 | 5504.6 | 916.7 | 772.4 | 768.7 | 642.1 | 291 |  |
| 5549DF | Distrito  53Federal | Brasília | 530010 | 5300108 | 2839228.3 | 454361.7 | 410524 | 249944.4 | 261093.3 | 118043 | 36 |

1

9

9

3

1

3
