# Supplementary material for: 10-year opportunistic mammographic screening scenario in Brazil and its impact on breast cancer early detection: a nationwide population-based study
Source: J Glob Health. 2022 Oct 14;12:04061. doi: 10.7189/jogh.12.04061 (PMC9564571; doi:10.7189/jogh.12.04061)
Supplement: Online Supplementary Document [file jogh-12-04061-s001.zip › Supplemental_table_states_data.doc]

uf ibge_uf_idavg_populationavg_pop_50_69avg_pop_50_69_ans_adjavg_pop_40_49avg_pop_40_49_ans_adjtotal_number_mmgavg_number_mmgsd_number_mmgtotal_number_mmg_0_39total_number_mmg_40_49total_

| 1AC | 12 | 824261.5 | 44565.3 | 38351.3609 | 43380.1 | 39272.4239 | 51655 | 5165.5 | 1841 | 1481 | 21607 |
| --- | --- | --- | --- | --- | --- | --- | --- | --- | --- | --- | --- |
| 2AL | 27 | 3269092.7 | 252421 | 211601.9705 | 209397.1 | 177701.1214 | 663789 | 66378.9 | 9778.2 | 51793 | 243619 |
| 3AM | 13 | 3850737.4 | 212149.3 | 174385.203 | 207572.1 | 164840.707 | 396544 | 39654.4 | 8615.6 | 15372 | 161088 |
| 4AP | 16 | 770483.8 | 38011.6 | 29887.2177 | 41743.7 | 35017.0757 | 8696 | 869.6 | 847.5 | 368 | 3728 |
| 5BA | 29 | 14594198.1 | 1210253.6 | 1051276.1261 | 959925.8 | 822924.8402 | 3029798 | 302979.8 | 53177 | 115390 | 869621 |
| 6CE | 23 | 8877918.9 | 734336.9 | 618671.171 | 578335.4 | 486741.6967 | 1007503 | 100750.3 | 19211.3 | 24320 | 354093 |
| 7DF | 53 | 2826656.5 | 236178.3 | 128498.4295 | 213015.2 | 134710.5665 | 118668 | 11866.8 | 10179.5 | 3990 | 36725 |
| 8ES | 32 | 3807732.7 | 353563.9 | 238158.3452 | 261394.6 | 179621.65 | 817661 | 81766.1 | 10355 | 27970 | 252216 |
| 9GO | 52 | 6570150.4 | 552801.8 | 462120.2669 | 464687.2 | 385598.2788 | 713231 | 71323.1 | 6723.6 | 28295 | 278890 |
| 10MA | 21 | 6895421.5 | 463571.5 | 425563.875 | 381117.7 | 348000.9264 | 450743 | 45074.3 | 6922.4 | 27750 | 166493 |
| 11MG | 31 | 20571609.3 | 2031704.3 | 1462480.7782 | 1433533.1 | 1037521.4658 | 5171171 | 517117.1 | 90896.8 | 167295 | 1498341 |
| 12MS | 50 | 2637115.7 | 227056.5 | 159953.3277 | 178540.9 | 132415.2633 | 386870 | 38687 | 6806.7 | 9922 | 146289 |
| 13MT | 51 | 3292974.3 | 245633.1 | 197457.1755 | 219244.9 | 178963.1629 | 265008 | 26500.8 | 3248 | 8920 | 101750 |
| 14PA | 15 | 8190778.4 | 498991.5 | 426810.3861 | 460281.8 | 397680.587 | 421052 | 42105.2 | 13316.7 | 19861 | 150600 |
| 15PB | 25 | 3925182.5 | 343511.3 | 294625.6162 | 262261.2 | 232263.2104 | 598444 | 59844.4 | 15829.2 | 16107 | 202873 |
| 16PE | 26 | 9281568.5 | 808774.2 | 659712.983 | 633493.1 | 517377.5246 | 1873766 | 187376.6 | 35956.1 | 52574 | 566917 |
| 17PI | 22 | 3233210.6 | 268968.7 | 243967.4374 | 203455.2 | 182265.2151 | 462946 | 46294.6 | 12008.8 | 7709 | 148253 |
| 18PR | 41 | 11042986.6 | 1091900 | 786773.5949 | 796202.1 | 578037.7854 | 3144024 | 314402.4 | 18409.3 | 141597 | 984621 |
| 19RJ | 33 | 16782709.5 | 1859751.5 | 1100493.3634 | 1231191.6 | 779333.0193 | 2363376 | 236337.6 | 26179.8 | 67667 | 656229 |
| 20RN | 24 | 3376329.9 | 286443.9 | 228622.8969 | 227948.6 | 187496.1601 | 494992 | 49499.2 | 7448.9 | 14225 | 178016 |
| 21RO | 11 | 1687868 | 116915.9 | 95871.6334 | 109188.8 | 94370.7694 | 116330 | 11633 | 3024.9 | 2856 | 49769 |
| 22RR | 14 | 517019.8 | 27559.1 | 23911.4742 | 27799.7 | 25153.3146 | 39563 | 3956.3 | 1745.3 | 1020 | 17250 |
| 23RS | 43 | 11147166.5 | 1263856.1 | 957140.2956 | 779041.8 | 583661.3202 | 3104685 | 310468.5 | 17955 | 111324 | 952634 |
| 24SC | 42 | 6757720.1 | 665332 | 501499.8895 | 482310.8 | 363459.8408 | 1828202 | 182820.2 | 24036.9 | 67540 | 593307 |
| 25SE | 28 | 2204797.8 | 173152.3 | 142221.5684 | 148188.6 | 123893.9196 | 327516 | 32751.6 | 9857.2 | 11649 | 128648 |
| 26SP | 35 | 44131679 | 4452773.1 | 2485121.147 | 3182680.2 | 1724179.0461 | 11577747 | 1157774.7 | 115272.4 | 401560 | 3854950 |
| 27TO | 17 | 1493641.3 | 100302.4 | 92209.726 | 88861.8 | 80837.7904 | 121656 | 12165.6 | 6693.7 | 4521 | 45214 |
